# Cell‐Specific Expression and Cellular Compartmental Regulation in Camptothecin Biosynthesis

**DOI:** 10.1111/pbi.70687

**Published:** 2026-05-26

**Authors:** Xiaolong Hao, Yinkai Yang, Tiantian Chen, Xiaoxuan Fan, Yiqing Peng, Qingyan Ruan, Qin Zhou, Fanghao Liu, Jiayi He, Yongpeng Li, Yue Feng, Jiyan Qi, Guoyin Kai

**Affiliations:** ^1^ Zhejiang Provincial TCM Key Laboratory of Chinese Medicine Resource Innovation and Transformation, Zhejiang International Science and Technology Cooperation Base for Active Ingredients of Medicinal and Edible Plants and Health, Jinhua Academy, School of Pharmaceutical Sciences Zhejiang Chinese Medical University Hangzhou China; ^2^ Shaanxi Key Laboratory of Qinling Ecological Intelligent Monitoring and Protection, School of Ecology and Environment Northwestern Polytechnical University Xi'an China

**Keywords:** amino acid vacuolar transporter, camptothecin, *Ophiorrhiza pumila*, single‐cell RNA sequencing, *STR‐TDC* gene cluster

## Abstract

Plant secondary metabolites such as monoterpenoid indole alkaloids (MIAs) show tightly regulated biosynthesis and accumulation in specific organelles of distinct cell types. However, the cell‐specific expression patterns of anticancer MIA camptothecin biosynthetic genes in *Ophiorrhiza pumila* and the regulatory mechanisms of camptothecin biosynthesis via cellular compartmentalisation remain poorly understood. Here, single‐cell RNA sequencing of *O. pumila* leaves generated 9181 cells, classified into four types using marker gene annotation and in situ hybridisation. Gene expression profiling combined with pseudotime analyses revealed that camptothecin biosynthetic genes are preferentially expressed in epidermal and young cells. Notably, the genes encoding camptothecin key enzymes, strictosidine synthase (STR) and tryptophan decarboxylase (TDC), have expanded within the *O. pumila* *STR‐TDC* gene cluster, supporting cell‐specific specialisation. Concurrently, this gene cluster has evolved a novel functional gene, *OpAVT1*, which annotated as a vacuolar transporter, mediates the transport of tryptophan from vacuole to the cytosol, thereby regulating camptothecin and other MIAs accumulation. Collectively, these findings establish a cellular‐level framework for camptothecin biosynthesis and regulation.

## 1 Introduction

**FIGURE 1:**
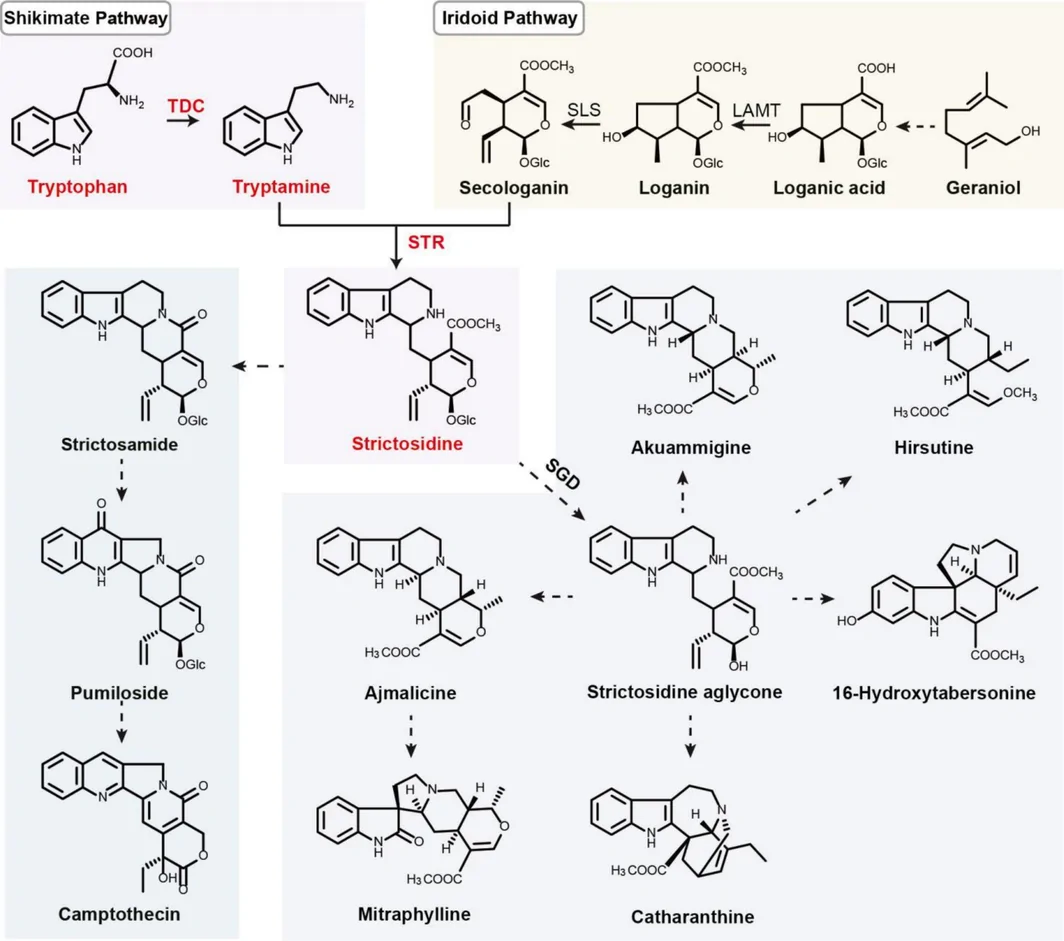
Putative biosynthetic pathways of camptothecin and other MIAs in *O. pumila* . In other MIA pathways, strictosidine undergoes early deglycosylation. In contrast, the camptothecin pathway in *O. pumila* uniquely retains glycosylated intermediates until late stages. STR, strictosidine synthase; TDC, tryptophan decarboxylase; LAMT, loganic acid *O*‐methyltransferase; SLS, secologanin synthase; SGD, strictosidine *β*‐glucosidase.

Monoterpenoid indole alkaloids (MIAs) constitute a notable class of natural products, encompassing over 3000 structurally diverse members with significant biological activities (Besseau et al. 2025; Caputi et al. 2018; De Luca et al. 2014; Hong et al. 2022; Kamileen et al. 2025; Lezin et al. 2024; Lombe et al. 2025; Zhang et al. 2022). Among these, camptothecin‐based drugs represent the only class of topoisomerase I‐specific inhibitors among anti‐cancer agents, exhibiting remarkable clinical efficacy and substantial market demand (Fan et al. 2022; Hevener et al. 2018; Kang et al. 2021; Ruan et al. 2021). The biosynthetic pathway of camptothecin in its source plants remains incompletely delineated, including uncharacterised cytochrome P450 (CYP450) enzymes specific to this biosynthetic cascade (Pu et al. 2023; Zhang et al. 2024). As a monoterpenoid indole alkaloid, its core structure was primarily synthesised via the strictosidine synthase (STR)‐catalysed Pictet‐Spengler reaction (Yang et al. 2021). Unlike most other MIAs, including catharanthine, hirsutine, mitraphylline, ajmalicine and akuammigine, which undergo immediate deglycosylation upon strictosidine formation, the camptothecin biosynthetic pathway follows a distinct route by maintaining glycosylated intermediates across nearly all biosynthetic stages. Accordingly, strictosidine‐derived intermediates such as strictosamide and pumiloside persist in camptothecin biosynthesis, with deglycosylation occurring exclusively at relatively late biosynthetic steps (Figure 1). The terpenoid moiety of camptothecin biosynthesis derived from an 11‐step catalytic cascade in iridoid pathway, while tryptamine generated from tryptophan via the shikimate pathway is produced through the action of tryptophan decarboxylase (TDC) (Hao et al. 2023; Li et al. 2025; Yang et al. 2021). In the well‐characterised MIA‐producing plant *Catharanthus roseus* , *STR* and *TDC* were clustered with *MATE* transporter, forming the *STR‐TDC* gene cluster (Franke et al. 2019). Genomic analysis of *Ophiorrhiza pumila*, the ideal model plant for the study of camptothecin biosynthesis and regulation, has also revealed an expanded *STR‐TDC* gene cluster (Hao et al. 2023; Rai et al. 2021). However, the expression profiles and functional roles of this cluster remain unknown.

Regulation of plant secondary metabolism is central to metabolic engineering of medicinal plants. In *O. pumila* , several transcription factors regulating camptothecin biosynthesis and accumulation have been identified (Hao et al. 2023, 2021; Wang, Hao, et al. 2022; Zhou et al. 2025). For example, OpNAC1 negatively regulated camptothecin production by directly binding to and repressing the biosynthetic gene *OpLAMT1*, whereas OpWRKY2 enhanced camptothecin biosynthesis by directly activating *OpTDC1* (Hao et al. 2023, 2021). Amino acid vacuolar transporters (AVTs), which mediate transmembrane partitioning of amino acids between vacuoles and the cytosol, act as critical hubs linking amino acid homeostasis to secondary metabolic flux by controlling substrate availability (Cai and Aharoni 2022; Dhatterwal et al. 2021; Heinemann and Hildebrandt 2021). As dynamic reservoirs for amino acids, vacuoles exert precise control over cytosolic amino acid pools via AVTs, whose substrate specificity enables targeted modulation of secondary metabolism (Martinoia et al. 2012). For instance, in tomato, SlAVT6A functioned as a core amino acid efflux transporter, promoting terpenoid accumulation and gibberellin biosynthesis by regulating vacuolar‐to‐cytosolic amino acid distribution, thereby enhancing insect resistance (Hao et al. 2025). Notably, within the *STR‐TDC* gene cluster of *O. pumila* , an AVT used for amino acid compartmentalisation has evolved, but the function remains unknown.

Single‐cell RNA sequencing (scRNA‐seq) has transcended the limitations of bulk sequencing, and emerged as a valuable technology for dissecting cell‐type‐specific functions in complex tissues (Mo and Jiao 2022; Rhaman et al. 2024). The technology has advanced rapidly in model plants like *Arabidopsis thaliana* (Xia et al. 2022), *Oryza sativa* (Wu et al. 2025), *Zea mays* (Nelms and Walbot 2019), *Solanum lycopersicum* (Omary et al. 2022) and *Populus* (Li, Wang, et al. 2023), enabling the construction of cellular atlases for diverse plant tissues, including root tips, xylem, stem, leaves and floral organs, and offering unprecedented insights into processes such as plant development and stress responses. In plants, secondary metabolites exhibit characteristic cell‐specific expression and accumulation in specialised organelles, thereby supporting defence and detoxification. Notably, scRNA‐seq has driven breakthroughs in understanding plant secondary metabolism, such as MIAs in *C. roseus* , paclitaxel in *Taxus wallichiana* and catechins in tea (Sun et al. 2023; Wang, Wu, et al. 2022; Yu et al. 2023; Zhan et al. 2023). It has pinpointed cell populations responsible for synthesising specific secondary metabolites, such as paclitaxel in mesophyll cells and catechins in leaf epidermal cells (ECs) and uncovered cell‐type‐specific expression patterns of key MIAs biosynthetic pathway genes in *C. roseus* (Li, Wood, et al. 2023; Sun et al. 2023). In addition, scRNA‐seq has identified cell‐specific transcription factors and regulatory networks that govern metabolite production, providing direct evidence for the cellular basis of metabolic diversity (Yu et al. 2023; Zhan et al. 2023). In *O. pumila* , camptothecin accumulates in all tested tissues, yet the cell‐type‐specific biosynthesis and regulation of camptothecin remain unknown. Thus, scRNA‐seq offered an unprecedented tool for unravelling the cell‐type‐specific biosynthesis and regulatory mechanisms of camptothecin in *O. pumila* .

*O. pumila* serves as an ideal model plant for investigating the biosynthesis and transcriptional regulation of camptothecin. Here, we performed high‐throughput scRNA‐seq on protoplasts isolated from *O. pumila* leaves, generating transcriptomic profiles for 9181 cells. These cells were classified into four major types using a dual validation strategy, namely marker gene annotation combined with in situ hybridisation (ISH), ensuring robust cell‐type assignment. Gene expression and pseudotime analyses revealed that camptothecin biosynthetic genes were enriched in ECs, particularly in younger cells, with their expression declining as cells differentiate along developmental trajectories. This spatial and temporal expression pattern enabled the identification of candidate potential regulatory factors and CYP450 enzymes involved in camptothecin biosynthesis. Phylogenetic analysis of the conserved *STR‐TDC* gene cluster across species uncovered significant expansion of both *STR* and *TDC* in *O. pumila* , underlying their functional diversification. *OpTDC3*, *OpTDC5* and *OpSTRs* all exhibited specific high expression in ECs, whereas *OpTDC4* showed a broad expression pattern. Furthermore, *OpAVT1*, encoding a vacuolar membrane amino acid transporter, was identified within this cluster. It shared a conserved genomic context with *OpTDCs* and *OpSTRs* within the *STR‐TDC* gene cluster, and this physical proximity likely facilitated coordinated regulation. *OpAVT1* localised to the tonoplast and mediated the efflux of L‐tryptophan from the vacuolar into the cytosol. As vacuoles served as the primary storage compartment for amino acids in plant cells, *OpAVT1* supplied stored tryptophan as a critical precursor for cytosolic camptothecin biosynthesis. ISH assays revealed that *OpAVT1* exhibited specific and high expression in ECs, and this cell‐type colocalisation provided a spatial basis for its functional coordination with *OpTDCs* and *OpSTRs*. By regulating vacuolar tryptophan export, *OpAVT1* directly influenced the cytosolic availability of tryptophan, thereby modulating the metabolic flux through the *TDC* and *STR* catalysed reactions, as well as the accumulation of camptothecin and other MIAs. Together, our findings provided novel insights into the cellular basis of camptothecin biosynthesis and compartmental regulation in *O. pumila* .

## 2 Results

### 2.1 Single‐Cell RNA Sequencing Reveals a Leaf Cell Atlas of *O. pumila*

**FIGURE 2:**
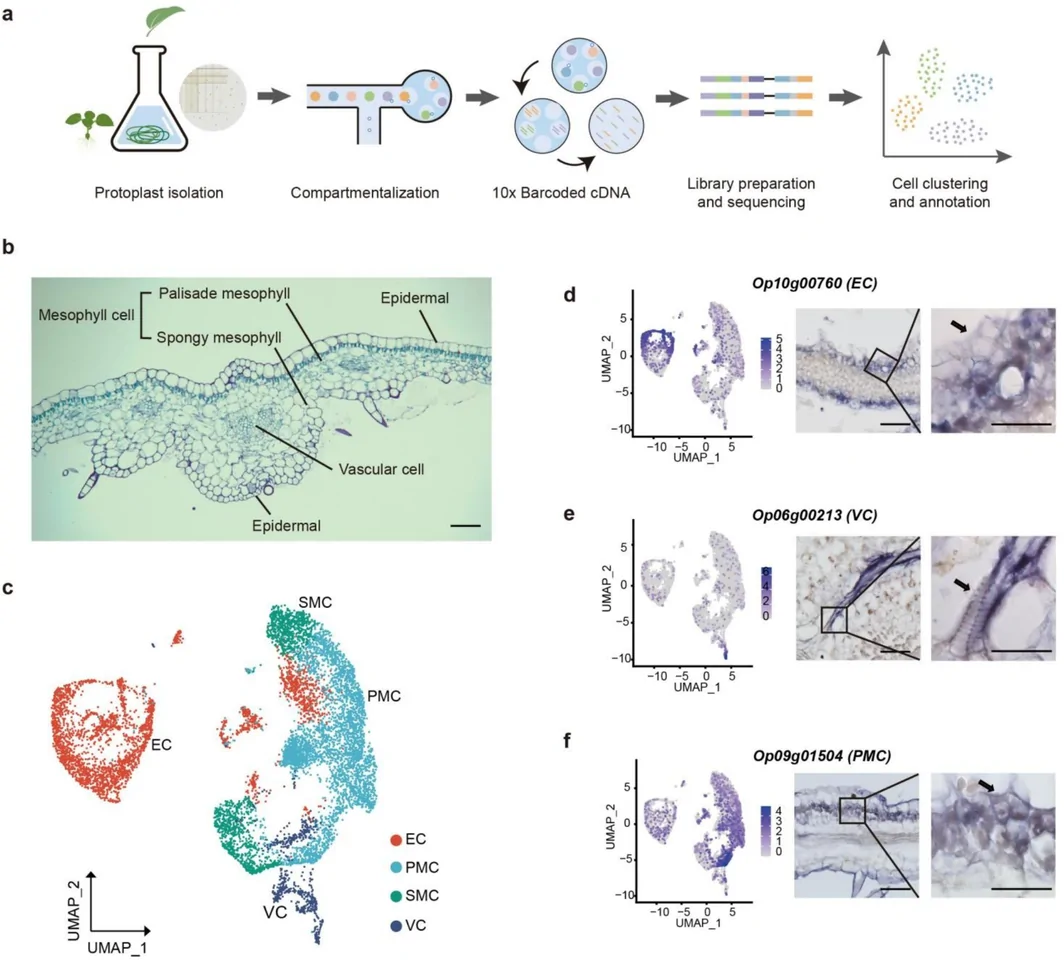
Single‐cell RNA sequencing (scRNA‐seq) and cell type annotation. (a) Schematic workflow of scRNA‐seq for *O. pumila* leaves. Protoplasts were isolated from two‐month‐old *O. pumila* leaves, barcoded with unique sequence tags using the 10 × Genomics Chromium system and processed for cDNA library construction. Sequencing was performed on the NovaSeq X Plus high‐throughput platform. (b) Bright‐field microscopic images of cell clusters in longitudinal sections of *O. pumila* leaves. Sections were stained with toluidine blue and imaged via light microscopy at 10 × magnification. Scale bars represent 100 μm. (c) UMAP visualisation of the four identified cell types, with distinct colours denoting different cell types. (d–f) UMAP visualisation of transcript abundance for three cell type‐specific marker genes and validation via in situ hybridisation. *Op10g00760* (epidermal cells, EC), *Op09g01504* (palisade mesophyll cells, PMC) and *Op06g00213* (vascular cells, VC). In situ hybridisation signals (purple) were imaged at 20 × and 100 × magnification, with positive signals marked by black arrows. Scale bars represent 50 μm and 10 μm, respectively.

Two month old *O. pumila* leaves were used for protoplast isolation, yielding a concentration of 650 cells μL^−1^ and 82% viability. Purified protoplasts were subjected to single‐cell RNA sequencing using the 10 × Genomics platform, generating 115.21 Gb of raw data (Figure 2a; Table S1). Barcode and unique molecular identifier (UMI) counting were used to assess viable cells (Figure S1, Figure S2), capturing 11 835 cells with a median of 916 genes per cell and 97.4% effective barcodes (Table S1). After stringent filtering, a gene expression matrix comprising 21 448 genes across 9181 cells were generated, with 21 094 genes functionally annotated. Principal component analysis (PCA) was performed on 2000 highly variable genes, and subsequent clustering divided these 9181 cells into 19 clusters (Table S2, Table S3). Uniform manifold approximation and projection (UMAP) and t‐distributed stochastic neighbour‐embedding (t‐SNE) were used to visualise cluster distribution and inter‐cluster correlations. Finally, 2710 cluster‐specific genes (CSGs) across the 19 clusters were identified (Figure S3, Table S4), with three representative differentially‐expressed genes (DEGs) for each cluster shown in Figure S4. These analyses yielded a high‐quality single‐cell transcriptomic dataset of *O. pumila* leaves, encompassing 19 distinct cell clusters with well‐characterised cluster‐specific genes.

Longitudinal sections of *O. pumila* leaves (perpendicular to veins), stained with toluidine blue and imaged at 10 × magnification via a Carl Zeiss microscope, revealed four major cell types including ECs, palisade mesophyll cells (PMCs), spongy mesophyll cells (SMCs) and vascular cells (VCs) (Figure 2b). In the absence of validated *O. pumila* marker genes, homologous alignments against published leaf single‐cell transcriptomes of *A. thaliana* and *C. roseus* were performed to identify candidate markers (Table S5, Table S6). Initial cell‐type annotation using SCINA software indicated that clusters 4, 5, 12, 14, 15 and 16 corresponded to ECs, clusters 2, 8, 9, 10, 11 and 18 to PMCs, and clusters 3 and 6 to SMCs. Clusters 7 and 17 were enriched for the phloem‐specific callose synthase gene *CALLOSE SYNTHASE 7* (*CALS7*, Op10g00722) and the early xylem differentiation marker *TRACHEARY ELEMENT DIFFERENTIATION‐RELATED 4* (*TED4*, Op06g01554), leading to their classification as VCs (Figure S5) (Endo et al. 2018; Xie et al. 2011). ISH for cluster‐specific genes confirmed cluster 0 as PMCs and cluster 1 as ECs (Figure S6). Thus, all 19 clusters were mapped to four major cell types, and cell counts revealed PMCs as the most abundant type (Figure 2c, Figure S7). Further ISH validation of cell‐type‐specific genes confirmed the accuracy of our annotations (Figure 2d–f, Figure S8). In general, these analyses enabled robust annotation of the 19 cell clusters into four major leaf cell types with their identities validated by histological and molecular evidence.

### 2.2 Cell‐Type‐Specific Distribution of Camptothecin Biosynthesis in *O. pumila* Leaves

**FIGURE 3:**
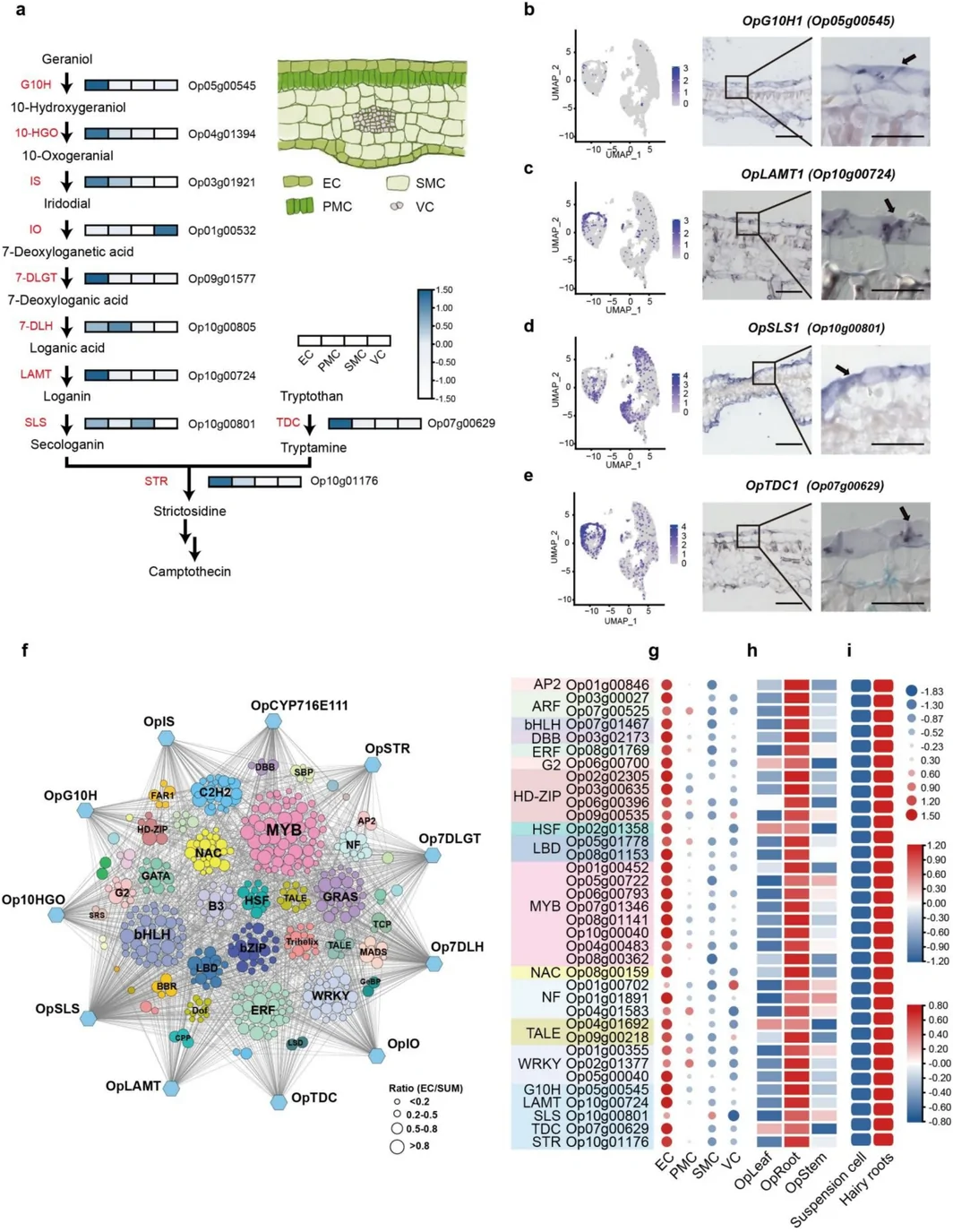
Expression patterns of the camptothecin biosynthetic pathway genes and transcription factors in different *O. pumila* cell types. (a) Transcript levels of camptothecin biosynthetic pathway genes in four cell types of *O. pumila* leaves, presented as a heatmap. (b–e) UMAP visualisation of transcript abundance for camptothecin biosynthetic genes *OpG10H1*, *OpLAMT1*, *OpSLS1*, *OpTDC1* in *O. pumila* leaves, with expression validated via in situ hybridisation. In situ hybridisation signals (purple) were imaged at 20 × and 100 × magnification, with positive signals marked by black arrows. Scale bars represent 50 μm and 10 μm, respectively. (f) Co‐expression network depicting interactions between transcription factors and camptothecin biosynthetic genes. Hexagonal nodes represent camptothecin biosynthetic genes, and coloured solid circles denote transcription factors. (g–i) Gene expression heatmaps for candidate transcription factors across four cell types (g) three tissue types (h) two cultures (i) The colour scale indicates the average scaled expression level of each gene.

Single‐cell transcriptome analysis of *O. pumila* leaves revealed that most camptothecin biosynthetic genes were specifically distributed in ECs (Figure 3a, Table S7). ISH validation of five functionally characterised genes, *OpG10H1* (*Op05g00545*), *OpLAMT1* (*Op10g00724*), *OpSLS1* (*Op10g00801*), *OpTDC1* (*Op07g00629*) and *OpSTR1* (*Op10g01176*), confirmed their epidermal cell localisation, consistent with scRNA‐seq results (Figure 3b–e, Figure 5e). The cell‐type‐specific expression patterns of camptothecin biosynthesis might be governed by cell‐type‐specifically expressed transcription factors. Using available transcriptomic datasets covering four cell types (ECs, PMCs, SMCs and VCs), three diverse tissues (roots, stems and leaves), two cultures (suspension cell and hairy roots), the co‐expression network analyses of camptothecin biosynthetic genes with all transcription factors were performed. After quality control and normalisation, Pearson correlation analysis identified 446 transcription factors showed co‐expression with 11 camptothecin biosynthetic genes and a Cytoscape‐generated network visualised these correlations with circle size indicating expression levels in ECs, implying potential regulatory roles in the pathway (Figure 3f, Table S8). Ultimately, 31 transcription factors, primarily from the MYB, HD‐ZIP and WRKY families, were identified as significantly associated with camptothecin biosynthetic genes and highly expressed in ECs, roots and hairy roots, matching accumulation patterns of camptothecin (Figure 3g–i). Among these transcription factors, OpHD‐ZIP7 (Op02g02305) is a previously reported regulator proposed to positively regulate camptothecin biosynthesis (Wang et al. 2023). Thus, these findings uncover the epidermal cell‐specific localisation of camptothecin biosynthetic genes and identify candidate transcription factors that likely govern this cell‐type‐specific expression, shedding light on the regulatory basis of camptothecin biosynthesis.

### 2.3 Pseudotime Analyses Screen the Candidate CYP450s for Camptothecin Biosynthesis

**FIGURE 4:**
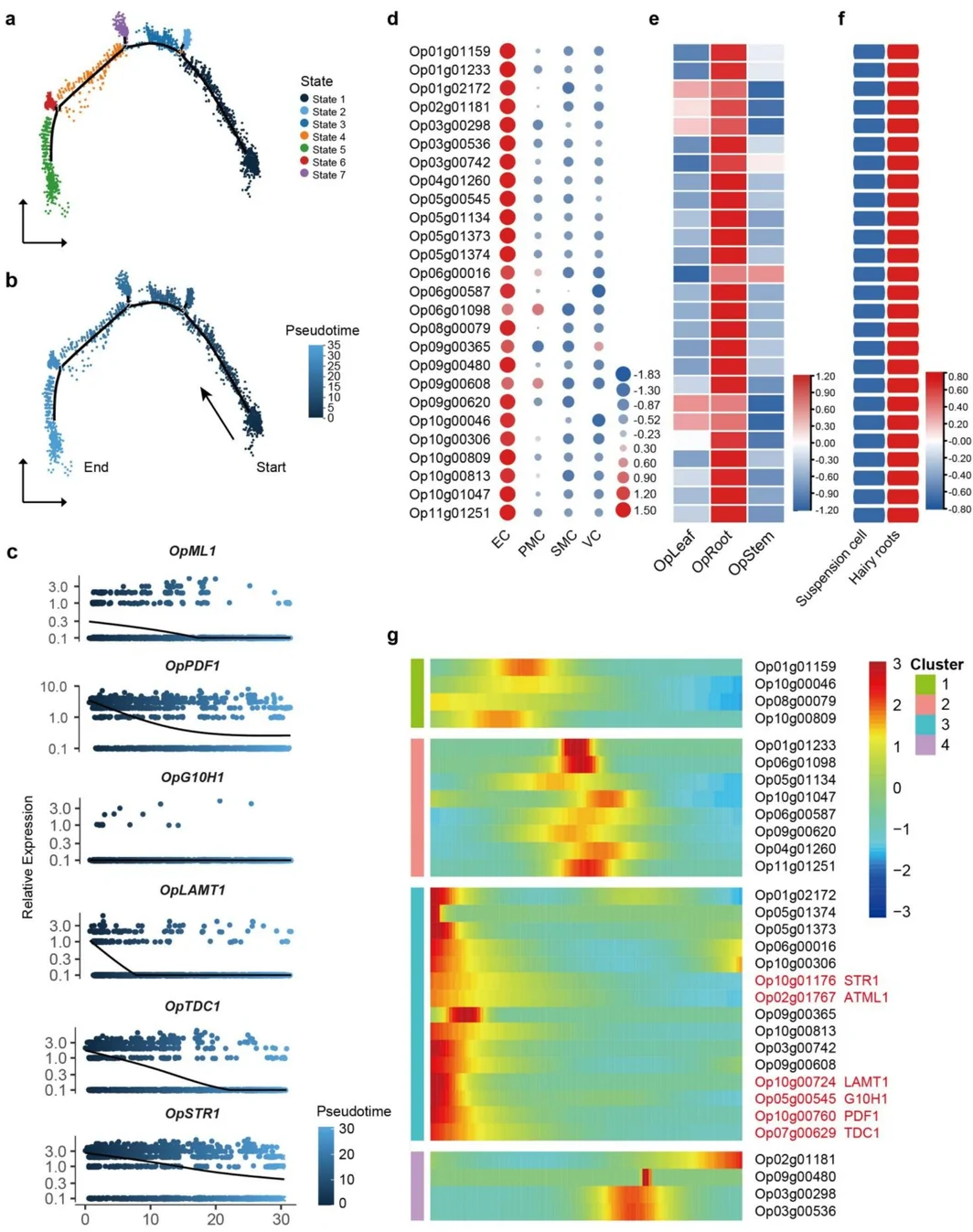
Developmental trajectory reconstruction of epidermal cells (ECs) and candidate CYP450 genes in *O. pumila* leaves. (a, b) UMAP visualisation of EC subgroups along pseudotime trajectories, coloured by pseudotime state (a) and differentiation status (b), respectively. (c) Expression dynamics of young EC marker genes (*OpML1* and *OpPDF1*) and camptothecin biosynthetic genes (*OpG10H1*, *OpLAMT1*, *OpTDC1* and *OpSTR1*) along pseudotime. d–f, Gene expression heatmap of the 26 CYP450 genes across four cell types (d), three tissue types (e), two cultures (f). The colour scale indicates the average scaled expression level of each gene. (g) Heatmap of 26 CYP450 gene expression during epidermal development. X‐axes represent the proposed chronological order, each row denotes a gene, each column represents the average expression value of the current cell state and colours gradient from red (high expression) to blue (low expression).

To characterise expression dynamics of camptothecin biosynthetic genes, pseudotime analysis was used to simulate epidermal cell development from young to mature stages, with three branch points resolving ECs into seven states (Figure 4a,b). The origin of the differentiation trajectory was validated via expression patterns of *ML1* (MERISTEM LAYER 1) and *PDF1* (PROTODERMAL FACTOR 1), two genes exclusive to the L1 layer (Abe et al. 1999; Sessions et al. 1999) (Figure 4c). Colour intensity of darker to lighter indicates differentiation progression from early to late stages, with the darkest shade set as the origin (Figure 4b). Visualisation of the 100 most differentially expressed genes across the pseudotime trajectory revealed clustering into four modules based on expression trends (Figure S9). Among them, module 1 genes, highly expressed at the trajectory end, were enriched primarily in photosynthesis and oxidative phosphorylation pathways and module 2 genes, upregulated at the trajectory start, were enriched in phenylalanine metabolism and phenylpropanoid biosynthesis (Figure S10, S11). Pseudotime trajectory analysis further defined dynamic expression of camptothecin biosynthetic genes during epidermal development, and most functionally characterised EC‐specific genes, including *OpG10H1*, *OpLAMT1*, *OpTDC1* and *OpSTR1*, exhibited high expression in young ECs, with levels declining as cells matured (Figure 4c, Figure S12). While the biosynthesis of camptothecin is tissue‐specific, key CYP450s responsible for synthesising certain intermediates remain poorly characterised. Using transcriptomic datasets, the expression patterns of all 192 *O. pumila* CYP450 genes were analysed, identifying 26 candidates with expression profiles similar to those of camptothecin biosynthetic genes. These 26 CYP450s were highly expressed in leaf ECs, more abundant in roots, and more highly expressed in hairy roots than suspension cells (Figure 4d–f). Further pseudotime analysis revealed 10 of these CYP450s were upregulated in young ECs, suggesting roles in early camptothecin biosynthesis (Figure 4g). Together, these findings map the developmental dynamics of camptothecin biosynthetic genes in ECs and identify candidate CYP450s, advancing cellular‐level understanding of camptothecin biosynthesis.

### 2.4 Evolutionary Relationships and Functional Validation of *STR‐TDC* Gene Cluster

**FIGURE 5:**
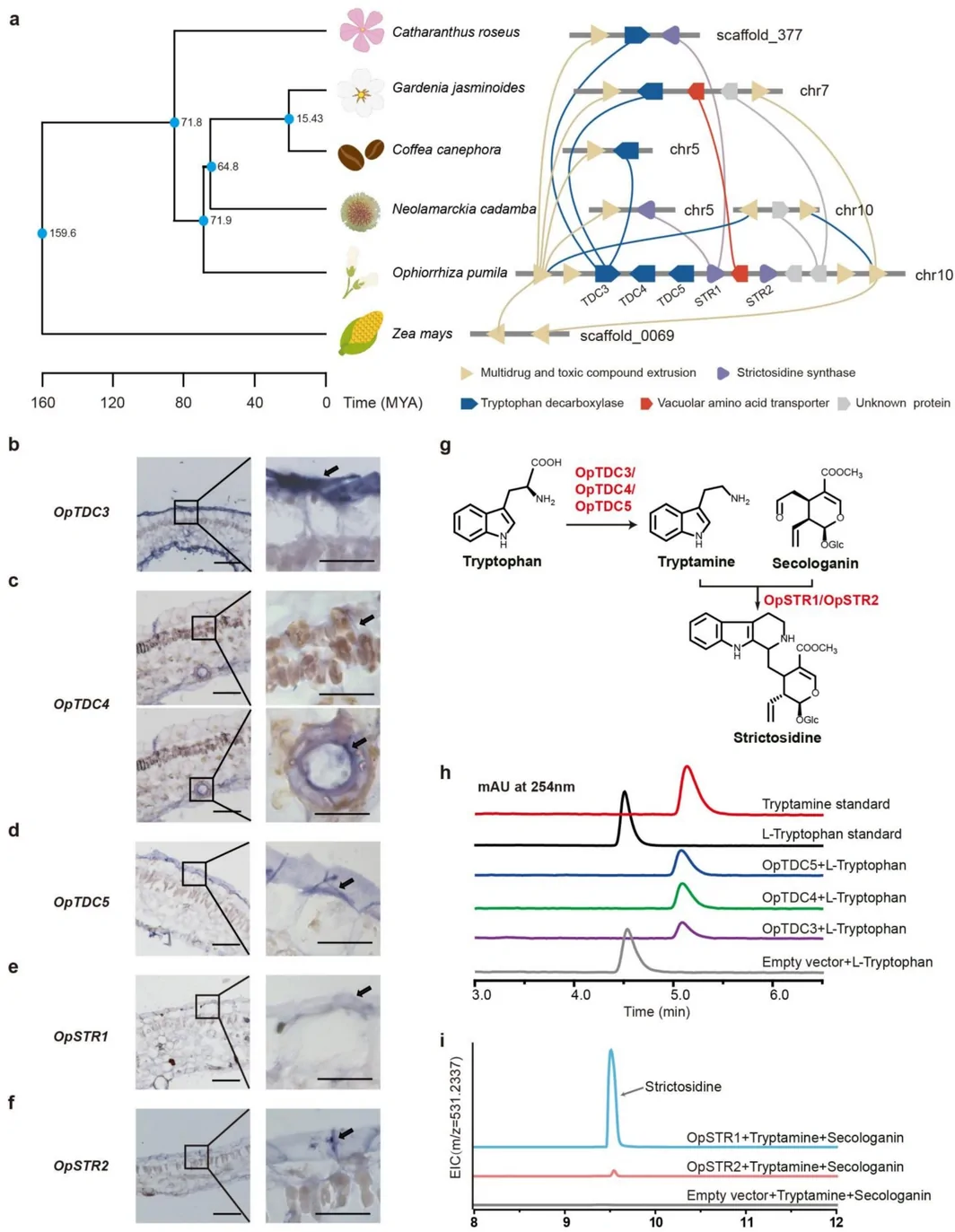
Evolutionary relationships and functional characterisation of the *O. pumila STR‐TDC* gene cluster. (a) Phylogenetic dating of *O. pumila* relative to *C. roseus* , *C. canephora* , *G. jasminoides* , *N. cadamba* and *Z. mays* , plus interspecific collinearity analysis of the *STR‐TDC* gene cluster. Genes with distinct annotations are represented by coloured symbols, and connected lines indicate covariance between gene pairs. (b–f) In Situ hybridisation validation of expression patterns for camptothecin biosynthetic genes within the *STR‐TDC* gene cluster. In situ hybridisation signals (purple) were imaged at 20 × and 100 × magnification, with positive signals marked by black arrows. Scale bars represent 50 μm and 10 μm, respectively. (g) Catalytic roles of *OpTDC* and *OpSTR* family members in the camptothecin biosynthetic pathway. (h) The catalytic activity of *Op*TDC3, *Op*TDC4 and *Op*TDC5 were detected by HPLC and detected at 254 nm. (i) The catalytic activity of *Op*STR2 was detected by UPLC. Shown is the extracted ion chromatogram (EIC) of strictosidine (m/z 531.2337). Control (CK), the pCold‐TF empty vector with substrate added.

The physical colocalisation of genes encoding secondary metabolic enzymes, known as metabolic gene clusters, is a conserved feature across plant species. Using our reported *O. pumila* genome, a metabolic gene cluster on chromosome 10 containing three *TDC*‐ and two *STR*‐encoding genes was identified. Phylogenetic timing analysis across *O. pumila* , *C. roseus* (Franke et al. 2019), *Coffea canephora* (Salojärvi et al. 2024), *Gardenia jasminoides* (Xu et al. 2020), *Neolamarckia cadamba* (Zhao et al. 2022) and *Z. mays* (Chen, Wang, et al. 2023) revealed *O. pumila* diverged relatively early, second only to *Z. mays* and *C. roseus* . Collinearity analyses showed MATE proteins were conserved in all species and flanked the cluster, with only *O. pumila* exhibiting *OpMATE* duplication. By contrast, *STR* and *TDC* are conserved only in specific species, with gene duplication observed exclusively in *O. pumila* , these duplicated genes were named *OpTDC3* (*Op10g01173*), *OpTDC4* (*Op10g01174*), *OpTDC5* (*Op10g01175*), *OpSTR1* (*Op10g01176*) and *OpSTR2* (*Op10g01178*) based on their gene IDs. Meanwhile, two uncharacterised genes (*Op10g01177* and *Op10g01180*) were conserved in some species, *Op10g01177* had a collinear homologue in *G. jasminoides* and *Op10g01180* had collinear homologues in both *G. jasminoides* and *N. cadamba* (Figure 5a). Previous studies have shown that in the early evolution of Gentianales plants, the *STR* gene was recruited into a genomic region that already contained *TDC* and *MATE* genes, thereby forming the complete gene cluster (Hwang et al. 2025). Conserved gene clusters with lineage‐specific internal expansion represent a recurrent evolutionary strategy for optimising specialised metabolism (Qiu et al. 2025). Consistent with this notion, our data indicated that although the gene cluster and the *STR* gene are broadly conserved across all MIA‐producing species, lineage‐specific gene duplication within this cluster occurred exclusively in *O. pumila* .

Gene duplication often drives functional diversification. To explore whether duplication altered expression specificity, cluster gene expression was analysed via single‐cell transcriptomics (Figure S13) and validated patterns using ISH assays. ISH showed *OpTDC3* and *OpTDC5* was expressed in ECs and *OpTDC4* in PMCs, ECs and stomata, *OpSTR1* and *OpSTR2* were both ECs‐expressed (Figure 5b–f). Functional validation via heterologous expression in *Escherichia coli* confirmed catalytic activity, and all three *OpTDC*s converted L‐tryptophan to tryptamine but not D‐tryptophan (Figure 5g,h, Figure S14–15). Using previously validated *Op*STR1 as a positive control, *Op*STR2 was shown to catalyse strictosidine formation from secologanin and tryptamine, consistent with known STR function, albeit with lower catalytic activity than *Op*STR1 (Figure 5g, Figure 5i, Figure S16). These results demonstrated that duplicated biosynthetic genes retained ancestral catalytic functions while acquiring cell‐type‐specific expression, enabling functional diversification.

### 2.5 OpAVT1 In *STR‐TDC* Gene Clusters Transport of Tryptophan from the Vacuole to the Cytoplasm

**FIGURE 6:**
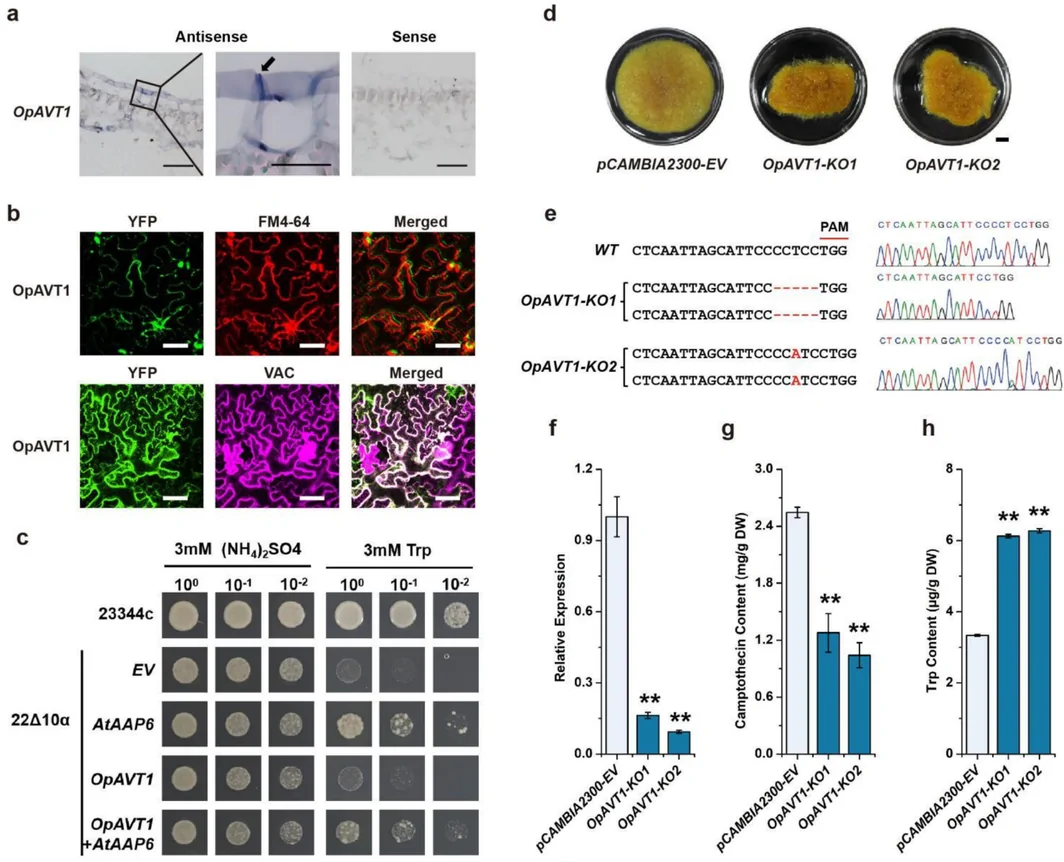
Functional validation of the *OpAVT1* gene. (a) In situ hybridisation validation of expression patterns for *OpAVT1*. In situ hybridisation signals (purple) were imaged at 20 × and 100 × magnification, with positive signals marked by black arrows. Scale bars represent 50 μm and 10 μm, respectively. (b) Subcellular localisation of OpAVT1 in *Nicotiana benthamiana*. The *Op*AVT1 fusion protein tagged with YFP was co‐localised with the tonoplast‐specific red fluorescent marker protein (mCherry‐VAC). YFP was excited at 488 nm, while mCherry and FM4‐64 were excited at 561 nm. Scale bars: 20 μm. (c) Yeast growth complementation assay validating the tryptophan transport function of *Op*AVT1. The *At*AAP6 and *Op*AVT1 genes were individually or co‐transformed into the *Saccharomyces cerevisiae* strain 22Δ10 α. Transformants were serially diluted (10, 10^−1^ and 10^−2^) and spotted onto selective media with ammonium sulfate or Trp as the sole nitrogen source. Wild‐type strain 23344c was used as control. (d) The phenotype of the *OpAVT1‐KO* transgenic hairy root lines (scale bars: 1 cm). (e) DNA sequencing of genomic DNA from *OpAVT1‐KO* transgenic hairy root lines confirmed successful gene editing at the *OpAVT1* locus. The wild‐type sequence was shown at the top, with the PAM sequence (TGG) was highlighted by the red line. The right panel details the specific insertions and point mutations identified in each independent line. (f) qRT‐PCR analysis revealed a significant reduction in *OpAVT1* transcript levels in the *OpAVT1‐KO* transgenic hairy root lines. For normalisation, *OpUBQ* served as a reference gene. The average expression level in control hairy root line was designated as the baseline (value = 1) for calculating relative transcript abundance. Error bars represent the SD of three technical replicates. (g–h) The concentration of camptothecin (g) and free tryptophan levels (h) in *OpAVT1‐KO* transgenic hairy root lines. Error bars represent the SD of three biological replicates. Asterisks indicate statistically significant differences (**p < 0.01) compared to the control.

During evolution, the *STR‐TDC* gene cluster continued to recruit new genes to acquire new functions. A conserved amino acid vacuolar transporter was identified in both *O. pumila* and *G. jasminoides* within the *STR‐TDC* gene cluster and designated as OpAVT1 (Figure 5a). Phylogenetic analysis with *Arabidopsis* AVT proteins revealed that it belongs to the AVT1 family and contains the family‐specific motif 9 (Figure S17–18). To validate its expression at the cellular level, ISH assays revealed that *OpAVT1* was specifically expressed in ECs of *O. pumila* leaves, consistent with the expression sites of known camptothecin biosynthetic genes (Figure 6a). Moreover, subcellular localisation assays in tobacco leaves demonstrated that *Op*AVT1 colocalised with the tonoplast marker VAC‐mCherry but not the plasma membrane dye FM4‐64, confirming its exclusive localisation to the vacuolar membrane (Figure 6b). To assess whether *Op*AVT1 functions as an amino acid transporter, complementation assays using the 22Δ10α yeast mutant were performed. Yeast expressing *Op*AVT1 alone failed to grow on medium containing 3 mM tryptophan (Trp), whereas expression of *At*AAP6, a known tryptophan transporter (Fischer et al. 2002), restored growth (Figure 6c). Notably, co‐expression of *Op*AVT1 with *At*AAP6 inhibited yeast growth compared to *At*AAP6 alone, indicating that *Op*AVT1 mediates tryptophan efflux in yeast (Figure 6c). Furthermore, after testing 19 other common amino acids, *Op*AVT1 was also shown to mediate the efflux of other aromatic amino acids when co‐expressed with *At*AAP1 or *At*AAP3 (Okumoto et al. 2004; Sanders et al. 2009), including phenylalanine and tyrosine (Tyr), as well as valine (Val), proline (Pro), serine (Ser), asparagine (Asn) and glutamic acid (Glu) (Figure S19).

We functionally characterised *OpAVT1* in *O. pumila* using CRISPR‐Cas9‐mediated knockout and obtained two homozygous knockout (KO) hairy root lines (*OpAVT1‐KO1* and *OpAVT1‐KO2*) from 44 transgenic positive lines (Figure 6d,e). These lines carry a 5‐bp deletion and a 1‐bp insertion in the *OpAVT1* coding sequence, respectively, resulting in premature translation termination at the 19th or 21st amino acid residue (Figure 6e, Figure S20). When cultured in shake flasks for 35 days, *OpAVT1‐KOs* hairy roots showed no significant change in biomass accumulation but displayed striking differences in root colour and growth phenotype (Figure 6d, Figure S21). qRT‐PCR analysis revealed that the relative transcript level of *OpAVT1* in the *OpAVT1‐KOs* was about 4‐fold lower than in controls. HPLC and LC–MS/MS analyses revealed that camptothecin levels were significantly reduced in *OpAVT1‐KOs*, whereas free tryptophan accumulated markedly (Figure 6f–h). Meanwhile, the levels of the other 19 amino acids also showed significant changes in the knockout lines (Figure S22). These results suggested that loss of *OpAVT1* impaired intracellular tryptophan transport, disrupting camptothecin biosynthesis.

Consistent with the above findings, prolonged cultivation revealed stable phenotypic divergence between *OpAVT1‐KO* and control lines. To systematically characterise these differences, *OpAVT1‐KO* and control plants were cultivated for 38 days, with phenotypic assessments performed every 7 days starting from Day 10. Notably, *OpAVT1‐KO* lines showed yellowish‐brown colour, suppressed root elongation and centralised growth, whereas controls were bright yellow with elongated roots and disc‐like structures. These phenotypic differences persisted throughout the cultivation period until the *OpAVT1‐KO* lines underwent senescence at Day 38. Although knockout lines exhibited faster early growth, their dry biomass ultimately converged to the level of control lines by Day 38. Biochemically, camptothecin content was consistently lower in knockout lines throughout, with a significant reduction in camptothecin yield detected from Day 24 onward. Collectively, these results demonstrated that knockout of the *OpAVT1* gene transiently promoted plant growth but persistently reduced camptothecin biosynthetic capacity (Figure S23).

### 2.6 OpAVT1 Modulated Camptothecin and Other MIAs Biosynthesis

**FIGURE 7:**
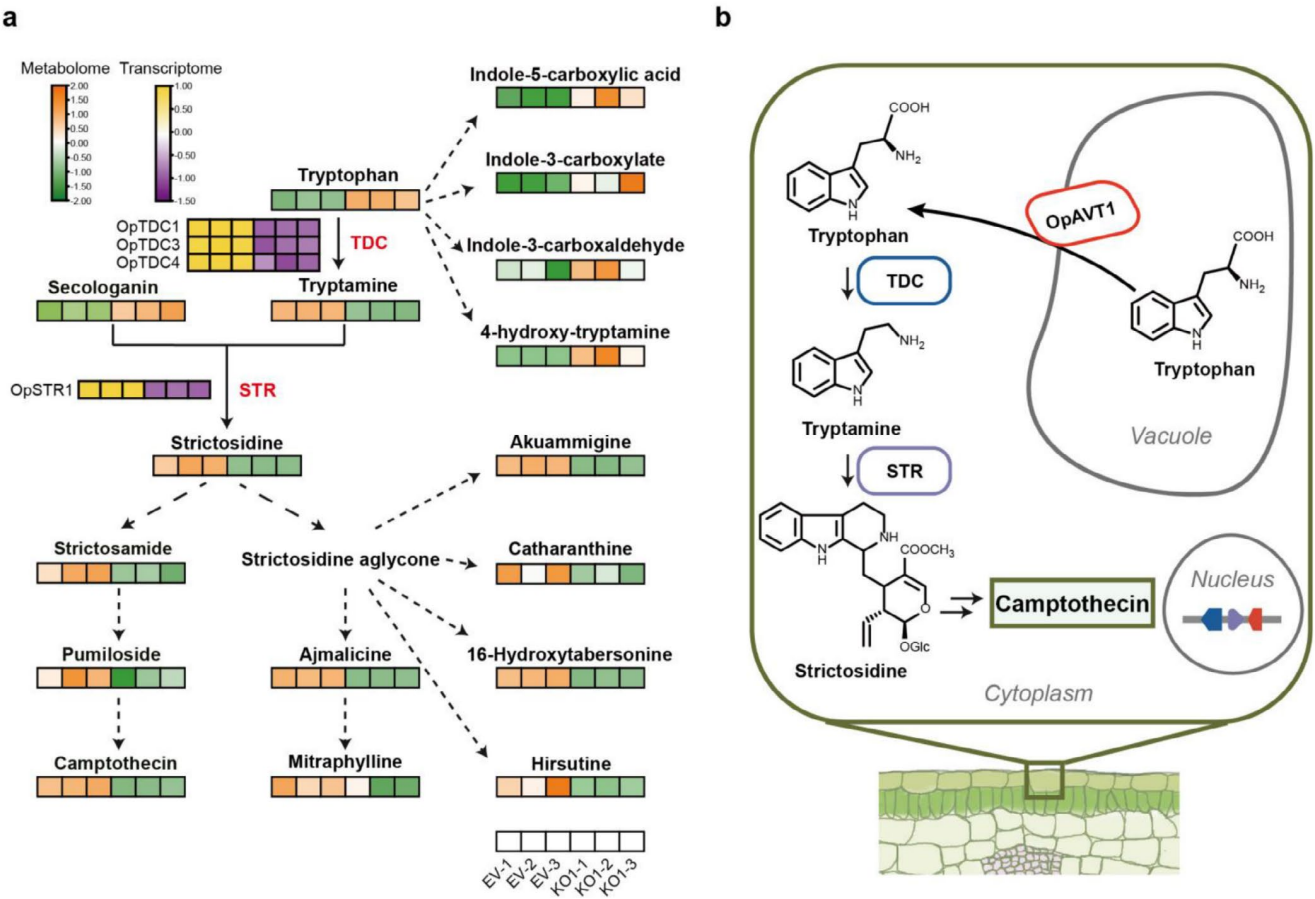
*OpAVT1* in *STR‐TDC* gene cluster regulates camptothecin biosynthesis. (a) Integrated metabolomic and transcriptomic analyses of *OpAVT1‐KO1* hairy root line. These analyses revealed the impact of *Op*AVT1 on genes and metabolites involved in the camptothecin and other MIAs biosynthetic pathway. Datasets from each omic approach are visualised as heatmaps, with distinct colour schemes for metabolomic and transcriptomic data. (b) Proposed working model for the *STR‐TDC* gene cluster in *O. pumila* epidermal cells. *Op*AVT1 transports free tryptophan from the vacuole into the cytoplasm, where it is converted into tryptamine catalysed by *Op*TDC. Tryptamine is subsequently catalysed by *Op*STR to form strictosidine, the central precursor for the biosynthesis of camptothecin. *EV*, *pCAMBIA2300* empty vector. *KO1*, *OpAVT1‐KO1*.

By mediating the efflux of vacuolar L‐tryptophan, *Op*AVT1 ensures a continuous substrate supply for the upstream camptothecin pathway operating in ECs. To further explore the regulatory mechanism of *OpAVT1*, metabolomic profiling of *OpAVT1‐KO1* revealed a marked accumulation of tryptophan and a concomitant reduction in tryptamine (Figure 7a, Figure S24). Consistent with this, strictosidine, the central and universal biosynthetic intermediate in the camptothecin pathway, exhibited significantly reduced accumulation. Concurrently, the CPT‐specific biosynthetic pathway, encompassing strictosamide, pumiloside and camptothecin, were markedly downregulated. Furthermore, other classical MIAs, such as hirsutine, 16‐hydroxytabersonine, mitraphylline, ajmalicine, akuammigine and catharanthine, were also detected with substantially decreased accumulation levels (Table S9). Depletion of tryptamine also led to accumulation of secologanin, the terpenoid skeletons of camptothecin. As L‐tryptophan serves as a common precursor for protein synthesis and multiple secondary metabolic pathways, *Op*AVT1 may participate in a broader metabolic regulatory network. In addition to the camptothecin and other MIA pathways, the levels of several tryptophan derivatives also exhibited significant changes. For instance, the contents of indole‐5‐carboxylic acid, indole‐3‐carboxylate, indole‐3‐carboxaldehyde and 4‐hydroxy‐tryptamine were all markedly increased (Figure 7a). Meanwhile, as a broad‐spectrum amino acid transporter, *OpAVT1* knockout lines also showed significant alterations in multiple amino acids and their corresponding alkaloid derivatives, including phenylalanine/tyrosine‐derived isoquinoline alkaloids and lysine‐derived pyridine alkaloids (Figure S24). Transcriptome analysis of *OpAVT1‐KO1* hairy root line showed reduced expression of *OpTDC1*, *OpTDC3*, *OpTDC4* and *OpSTR1* genes in the camptothecin biosynthetic pathway that were expressed in ECs and contributed to camptothecin biosynthesis (Figure 7a). Meanwhile, the differentially expressed genes were significantly enriched in amino acid‐related pathways, including phenylpropanoid biosynthesis, tyrosine metabolism and glutathione metabolism, as well as in plant‐pathogen interaction, monoterpenoid biosynthesis and isoquinoline alkaloid biosynthesis pathways (Figure S25). Together, these data indicated that *Op*AVT1 regulated camptothecin biosynthesis through two mechanisms, such as modulating tryptophan transport and controlling the expression of key biosynthetic genes. Thus, *Op*AVT1 mediated vacuole‐to‐cytoplasm transport of multiple amino acids and its loss disrupted biosynthesis of diverse metabolites.

## 3 Discussion

### 3.1 scRNA‐Seq Dissects Camptothecin Biosynthesis and Transcriptional Regulation

scRNA‐seq has emerged as a transformative tool in plant biology, enabling dissection of complex physiological and biochemical processes at unprecedented cellular resolution (Ali et al. 2024). Medicinal plants, rich in bioactive natural metabolites, are of particular interest; for example, studies in *C. roseus* revealed the multicellular compartmentalisation of vinblastine biosynthesis, illuminating mechanisms underlying metabolite production in medicinal species (Li, Wood, et al. 2023; Sun et al. 2023). Here, scRNA‐seq was applied to *O. pumila* to resolve the cellular distribution and developmental dynamics of camptothecin biosynthetic genes and to identify regulatory components. Protoplast isolation is critical for scRNA‐seq, as it captures cytoplasmic transcripts with higher UMI counts than single‐nucleus RNA sequencing (Chen, Ge, and Lu 2023; Mo and Jiao 2022). Given the enzyme dependency and plant material sensitivity of protoplast preparation, two‐months‐old fresh sterile seedlings were used to optimise cell concentration and viability, generating the first scRNA‐seq dataset for *O. pumila* leaves. Cell clustering, validated by microscopic tissue observation, combined with marker gene homology and ISH experiments, identified four major cell types including ECs, PMCs, SMCs and VCs. This classification was robust, as ISH confirmed cell‐type specificity of marker genes and camptothecin biosynthetic genes.

Notably, scRNA‐seq and ISH consistently showed that known camptothecin biosynthetic genes in *O. pumila* , including *OpG10H1*, *OpLAMT1*, *OpSLS1*, *OpTDC1* and *OpSTR1*, were specifically expressed in ECs. This contrasts with the multicellular distribution of MIA‐biosynthetic genes in *C. roseus* , highlighting ECs as a focal point for camptothecin production in *O. pumila* and enabling precise identification of cell‐type‐specific transcription factors (Li, Wood, et al. 2023; Sun et al. 2023). Pseudotime analysis, which models cellular dynamics by ordering transient expression patterns, revealed that camptothecin biosynthetic genes are highly expressed in young ECs of *O. pumila* and downregulated as cells mature. Integrating this developmental trajectory with scRNA‐seq and bulk transcriptome data, 10 candidate CYP450 genes were identified with expression patterns congruent with camptothecin accumulation. Together, the scRNA‐seq dataset for *O. pumila* provides a cellular framework for understanding camptothecin biosynthesis. By resolving cell‐type‐specific gene expression and developmental dynamics and complementing bulk sequencing, this resource enhances the efficiency of candidate gene screening, advancing to decipher the regulation of this clinically important metabolite. However, several limitations should be acknowledged. Our current dissection of camptothecin biosynthesis is primarily restricted to the upstream pathway. The identification of genes involved in the downstream route specifically, the conversion of strictosidine to camptothecin remains unresolved. Future integration of cell‐type‐resolved maps covering downstream genes will be essential to establish a complete spatial regulatory model of the entire biosynthetic pathway.

### 3.2 *STR‐TDC* Gene Cluster Function in Metabolite Biosynthesis Across Distinct Cell Types

Plant genomes frequently harbour metabolic gene clusters, physical groupings of genes encoding enzymes for secondary metabolite biosynthesis, yet their single‐cell transcript dynamics remain poorly characterised (Zhan et al. 2022). Here, we characterised the conserved *STR‐TDC* gene cluster in *O. pumila* , which has undergone species‐specific duplication events and comprises three *OpTDC*, two *OpSTR* and one *OpAVT1* gene. Phylogenetic and comparative genomic analyses suggest that during early evolution of the Gentianales order, *TDC* and *MATE* genes likely existed as a ‘core scaffold’ within an ancestral genomic region, followed by the subsequent recruitment of *STR* genes via local duplication or transposition events, ultimately leading to the stepwise assembly of the complete *STR‐TDC* gene cluster. Notably, while *STR* and *TDC* remain as single‐copy genes in other monoterpene indole alkaloid‐producing species such as *C. roseus* , they have undergone pronounced lineage‐specific duplications in *O. pumila* . Gene duplication events have been proposed as a key driver for the expansion of metabolic gene clusters (Zhang et al. 2023). Three *OpTDC* genes, tandemly arranged in the *STR‐TDC* gene cluster and validated to catalyse L‐tryptophan conversion to tryptamine, exhibit striking cell‐type‐specific transcript accumulation, as shown by scRNA‐seq and ISH assays. Tryptamine, a key metabolite linking primary and secondary metabolism, feeds into pathways for auxin, serotonin and melatonin biosynthesis (Negri et al. 2021), and the cell‐type partitioning of *OpTDC* expression likely enables this metabolite to participate in diverse metabolic processes across distinct cellular compartments. In contrast, two *OpSTR* genes, both expressed in ECs, catalyse strictosidine formation, a critical precursor for camptothecin. Despite shared epidermal localisation, *OpSTR1* and *OpSTR2* differ in expression levels and catalytic efficiency. This suggests camptothecin, which functions in plant defence, predominantly accumulates in ECs, with *OpSTR2* potentially compensating for *OpSTR1* to accelerate defence compound biosynthesis under inducing conditions.

Notably, the *STR‐TDC* gene cluster contains *OpAVT1*, an amino acid vacuolar transporter, positioned between the two *OpSTR* genes. Functional analyses demonstrated OpAVT1 transported tryptophan and other secondary metabolite precursors from vacuoles, where amino acids were stored, to the cytoplasm. Knockout of *OpAVT1* disrupted amino acid compartmentalisation, impairing biosynthesis of camptothecin and other secondary metabolites. Thus, scRNA‐seq of *O. pumila* leaves led to the identification of a gene cluster, comprising three *TDC*, two *STR* and one *AVT* gene, specifically associated with ECs, where camptothecin biosynthesis occurs. Free tryptophan, which supplies the indole skeleton for camptothecin, is stored in vacuoles and transported to the cytoplasm by the tonoplast‐localised OpAVT1 protein. In the cytosol, tryptophan is converted to tryptamine by *OpTDC*‐encoded enzymes and subsequently catalysed by *OpSTR* into strictosidine, the central precursor for camptothecin biosynthesis (Figure 7b). Precursor competition frequently underlies the trade‐off between growth and specialised metabolism (Liu et al. 2024). Consistent with this notion, *OpAVT1* knockout lines showed a transient growth advantage while suffering an irreversible impairment of camptothecin biosynthetic capacity (Figure S23). In addition, hormonal dysregulation may also be a contributing factor to the developmental abnormalities observed in *OpAVT1* knockout hairy roots (Table S10). Together, these findings revealed that the *STR‐TDC* gene cluster in *O. pumila* has evolved both duplicated biosynthetic genes with cell‐type‐specific expression enabling metabolic diversification and a conserved *Op*AVT1 transporter that coordinates amino acid homeostasis to link plant growth and secondary metabolism.

## 4 Methods

### 4.1 Protoplast Isolation

The samples were washed and chopped with a razor blade and pectinase and cellulase were added to digest the cell walls. Afterwards, the digestion was terminated by adding the corresponding concentration of mannitol. Single cell suspensions were quality checked and counted, generally requiring cell viability of 80% or more. The tested cells were washed, resuspended and prepared to a suitable cell concentration of 700~1200 cells/μL, ready for 10 × Genomics Chromium system on‐board operation.

### 4.2 Single‐Cell Library Construction and Data Preprocessing

Using the 10 × Genomics Chromium system, sequence labelled Gel Bead, sample, reagent premixes and oils were loaded into their respective injection channels. The single‐cell microreaction systems GEMs encapsulated by oil droplets through a network of microfluidic channels forming a ‘double‐cross’ system. After normal formation of GEMs, GEMs were collected to achieve labelling by reverse transcription. The GEMs were treated with oil‐breaking, purified and enriched with one‐stranded cDNA using magnetic beads, and then subjected to cDNA amplification and quality control. The qualified cDNAs were subjected to second‐generation sequencing library construction and quantitative analysis. The constructed libraries were sequenced using the Novaseq Xplus high‐throughput sequencing platform.

### 4.3 Cell Type Identification

To identify the cell type, using ‘LogNormalize’ of the Seurat software to normalise the expression matrix after filtering. Before clustering, dimensionality reduction was performed using PCA (Pearson 1901) dimensionality reduction analysis. Clustering is performed using the ‘SNN’ method, which identifies different cell subtypes by clustering cells of the same type together based on gene expression patterns in each cell. Afterwards, the nonlinear dimensionality reduction approach UMAP algorithm is used for visualisation purposes (McInnes and Healy 2018). Marker genes of different cell types were collected using reported single‐cell transcriptomes of *A. thaliana* , *C. roseus* . Homology comparison was performed using the blast tool of TBTOOLS (e‐value: 1‐e5) (Chen et al. 2020) and *O. pumila* proteins with high similarity were obtained. The marker genes were collated into a list and the semi‐supervised modelling software SCINA was used for cell type determination (Zhang et al. 2019), and combined with the manual determination method to classify all the cell types, finally all the cell taxa were classified into four cell types, namely ECs, PMCs, SMCs and VCs.

### 4.4 In situ Hybridisation Experiments

The protocol for the ISH of gene expression was performed as described previously (Javelle et al. 2011). The Open Reading Frame sequence of the target gene was constructed into the pKW89 vector containing the T7 and SP6 promoters. The recombinant plasmid was linearised using enzymatic digestion before using as a template for transcription. In vitro transcription was performed with a DIG RNA Labeling Kit (Sp6/T7) (Roche, Basel, Switzerland). The transcribed RNA probe was base‐digested into a fragment length of 150 bp. The quality of the probes was determined by spot hybridisation. *O. pumila* leaves were taken for paraffin embedding and sliced with a slicer to control the thickness at 10 μm. Selected paraffin sections were dewaxed and rehydrated with gradient ethanol. Unsolved proteins with protease buffer to expose the target RNA requiring hybridization. The probe was diluted in 50% formamide solution according to the results of spot hybridisation. Each section was spiked with 100 μL of the mixture, covered with a coverslip to avoid air bubbles and hybridised at 50°C overnight. After repeated washing with 0.2 × SSC solution at 55°C and NTE solution at 37°C, the antibody was applied to the sample with a 1250‐fold dilution in TBST solution. After moisturising at room temperature for 2 h, the excess antibody was washed out, and NBT/BCIP was added for colour development in dark. After 1–3 days, the slices were washed with TE buffer and observed under the microscope. Primers used for cloning are listed in Table S11.

### 4.5 Pseudotime Analysis

Dimensionality reduction clustering analysis of ECs allowed further subdivision of cell subpopulations. The secondary subdivided cell subpopulations were screened for all differentially expressed genes in the cell class using the DDRTree algorithm in the Monocle2 (Trapnell et al. 2014). And dimensionality reduction was performed and constructed into a minimum spanning tree. Single‐cell expression data are then projected into a low‐dimensional space and searched for optimal sorting. Finally, the optimal cell development or differentiation proposed time trajectory was fitted. The starting position of the differentiation trajectory is determined based on the expression pattern of the specifically expressed gene. Function genes of camptothecin biosynthetic pathway were visualised using the plot genes in pseudotime function.

### 4.6 Collinearity and Evolutionary Analysis Among Species

The whole genome sequence file and gene structure annotation file were obtained from EnsemblPlants database. The synonymous relationships of *O. pumila* with *C. roseus* , *C. canephora* , *G. jasminoides* , *N. cadamba* and *Z. mays* were conducted with MCScanX program and TBtools software to perform synteny analysis (Wang et al. 2012). Divergence times and evolutionary relationship trees for each species were obtained through an online website (http://www.timetree.org/).

### 4.7 Extraction and Quantification of Camptothecin and Free Tryptophan

Transgenic hairy root lines were cultured in 100 mL of B5 liquid medium in Erlenmeyer flasks at 25°C for 35 days. The samples were subsequently dried and ground into fine powder. Approximately 50 mg of dried powder was accurately weighed and extracted with 1 mL of methanol for camptothecin analysis. The mixture was vortexed thoroughly and subjected to ultrasonic extraction for 30 min, followed by centrifugation (12 000 × g, 10 min) to collect the supernatant. Camptothecin content was quantified according to the method described before (Yang et al. 2021). The 0.5 g dried powder was extracted with 2 mL of 0.1% (v/v) formic acid aqueous solution by vortexing and sonication for 30 min for free tryptophan analysis. The supernatant was collected after centrifugation, and the residue was re‐extracted with an additional 2 mL of 0.1% formic acid. The combined supernatants were adjusted to a final volume of 5 mL for subsequent analysis. Chromatographic separation was performed on an Agilent Poroshell 120 HILIC‐Z column (2.7 μm, 3.0 × 150 mm) using a QTRAP 5500 LC–MS/MS system (AB SCIEX, USA). The mobile phase consisted of (A) 0.1% formic acid in water and (B) acetonitrile, delivered at a flow rate of 0.5 mL/min. Detection was carried out in positive electrospray ionisation (ESI+) mode.

### 4.8 Validation of the Transport Function of OpAVT1 for Tryptophan

Functional characterisation of OpAVT1 in tryptophan transport was performed using yeast complementation assays. The full‐length cDNA sequences of OpAVT1 and AtAAP6 from *A. thaliana* were amplified by PCR and cloned into pESC‐Ura vector, which could tag two genes in yeast. The *S. cerevisiae* strain 22Δ10α (Besnard et al. 2016) was transformed with these recombinant constructs or empty vector (negative control) followed by selection on SD‐Ura solid medium and wild‐type strain 23344c was used as positive control. Selected transformants were cultured in liquid medium containing 1.7 g/L yeast nitrogen base without amino acids or ammonium sulfate (BD, USA), 20 mg/L uracil and 20 g/L galactose, with either 3 mM (NH4)_2_SO_4_ or tryptophan as the sole nitrogen source. After growing to OD600 = 1.0 at 28°C with 200 rpm shaking, yeast cells were harvested, serially diluted (10‐ and 100‐fold) and 5 μL aliquots of each dilution were spotted onto selective plates containing (NH4)_2_SO_4_ or tryptophan as nitrogen sources, followed by 3–5 days incubation at 30°C before growth observation and documentation. Primers used for cloning are listed in Table S11.

### 4.9 Metabolome Analysis

The transgenic lines were cultured in shaking flasks and rapidly frozen in liquid nitrogen. Biological samples used for metabolite detection were lyophilised (Scientz‐100F, 63 h), pulverised (MM 400 grinder, 30 Hz, 1.5 min) and 30 mg precisely aliquoted for extraction with 1500 μL of −20°C pre‐chilled 70% methanol containing 250 μg/mL internal standard. After 6 cycles of vortexing (30 s every 30 min), supernatants were collected (12 000 rpm, 3 min), filtered (0.22 μm) and stored in vials for UPLC–MS/MS. Hormone detection: Frozen biological samples (fresh samples by default) were ground into powder in liquid nitrogen using a ball mill (30 Hz, 1 min). Approximately 50 mg of the homogenised sample was weighed, mixed with 10 μL of internal standard solution (100 ng/mL) and 1 mL of extraction solvent (methanol/water/formic acid, 15:4:1, v/v/v) and vortexed for 10 min. After centrifugation (12 000 rpm, 5 min, 4°C), the supernatant was collected, concentrated and reconstituted in 100 μL of 80% methanol/water. The solution was filtered through a 0.22 μm membrane and analysed using LC–MS/MS (QTRAP 6500+, SCIEX).

## Author Contributions

G.K., J.Q. and X.H. conceived and designed the project. X.H., Y.Y., T.C. and X.F. prepared materials and performed the experiments. X.H., Y.Y., T.C., Y.P., Q.R., Q.Z., F.L. and J.H. performed the bioinformatics analysis and analysed the data. X.H., Y.Y., T.C. and X.F. wrote the manuscript. G.K., Q.T., X.H., Y.L. and Y.F. revised the manuscript. All authors read and approved the final manuscript.

## Funding

This work was supported by National Key Research and Development Program of China, 2023YFC3503900. National Natural Science Foundation of China, 82474030, 32570405, 32300673. National “Ten‐thousand Talents Program” for Leading Talents of Science and Technology Innovation in China, National Young Qihuang Scholars Training Program. Young Talent Support Project of China Association of Chinese Medicine, CACM‐2024‐QNRC2‐B33. Zhejiang Chinese Medical University Postgraduate Scientific Research Fund Project, 2023YKJ09. Research Project of Zhejiang Chinese Medical University, 2025JKZKTS21. Zhejiang Provincial Natural Science Foundation of China, LZ26H280003.

## Ethics Statement

All experiments in this article do not include human or animal related research.

## Conflicts of Interest

The authors declare no conflicts of interest.

## Supporting information


**Figure S1:** Barcode Rank Plot.
**Figure S2:** Cluster analysis of scRNA‐seq.
**Figure S3:** UMAP visualisation of 19 cell clusters.
**Figure S4:** Three specifically expressed genes in each of 19 clusters.
**Figure S5:** Expression levels of the VC marker genes in 19 clusters.
**Figure S6:** Localisation of specifically expressed genes in cluster 0 and cluster 1.
**Figure S7:** Proportion of four celltype in *O. pumila leaves*.
**Figure S8:** UMAP visualisation of transcript accumulation of cell type‐specific expressed genes and experimental validation by ISH.
**Figure S9:** 100 genes with the most significant differences in expression over the pseudotime trajectory of EC subclusters.
**Figure S10:** KEGG pathway analysis of genes in module1.
**Figure S11:** KEGG pathway analysis of genes in module 2.
**Figure S12:** Candidate CPT biosynthesis function genes expression changed with the pseudotime time.
**Figure S13:** UMAP visualisation of genes on gene cluster.
**Figure S14:** SDS‐PAGE of purfied OpTDCs and OpSTRs on gene cluster.
**Figure S15:** Catalytic activity of OpTDC3, OpTDC4 and OpTDC5 were detected by HPLC.
**Figure S16:** MS spectrums of OpSTR2 catalytic products.
**Figure S17:** Phylogenetic analysis of OpAVT1.
**Figure S18:** Protein motifs of AtAVTs and OpAVTs.
**Figure S19:** Yeast growth complementation assay validating amino acid transport function of OpAVT1.
**Figure S20:** Identification of positive *OpAVT1‐KOs* transgenic hairy root lines.
**Figure S21:** Biomass accumulation and camptothecin yield in *OpAVT1‐KOs* transgenic hairy root lines.
**Figure S22:** Detection of other free amino acids in the *OpAVT1‐KOs* transgenic hairy root lines.
**Figure S23:** Time‐course analysis of *OpAVT1‐KOs* transgenic hairy root lines during hairy root suspension culture.
**Figure S24:** Metabolome detection in the *OpAVT1‐KO1* transgenic hairy root line.
**Figure S25:** Transcriptomic analysis of the *OpAVT1‐KO1* transgenic hairy root line.

**Table S1:** Statistical data of scRNA‐seq.
**Table S2:** Cell quality assessment and filtration.
**Table S3:** Number of cells in each cluster.
**Table S4:** Top 10 maker genes of each cluster.
**Table S5:** Marker genes from *Arabidopsis thaliana* and *Catharanthus roseus* leaves.
**Table S6:** Marker genes of two types of Mesophyll cell from *Arabidopsis thaliana* leaves.
**Table S7:** Transcript accumulation of cell type‐specific expressed genes.
**Table S8:** Correlation analysis of transcription factors with functional genes for camptothecin biosynthesis.
**Table S9:** Metabolomic data of the camptothecin pathway in *OpAVT1‐KO1* hairy root line.
**Table S10:** Hormone profiling data of *OpAVT1‐KO1* hairy root line.
**Table S11:** Primer used in this study.

## Data Availability

The single‐cell and bulk RNA sequencing data generated in this study have been deposited to NCBI with the accession number PRJNA1206684 (http://www.ncbi.nlm.nih.gov/bioproject/1206684). The raw transcriptome data for the *OpAVT1‐KO1* hairy root line is now available under NCBI accession number PRJNA1329249 (http://www.ncbi.nlm.nih.gov/bioproject/1329249). The genome of *O. pumila* used in this study is openly available in China National GenBank Database (CNGB) Nucleotide Sequence Archive (https://db.cngb.org/cnsa) with project accession ID: CNP0002219.
