# Supplementary material for: Cell‐Specific Expression and Cellular Compartmental Regulation in Camptothecin Biosynthesis
Source: Plant Biotechnol J. 2026 May 26:10.1111/pbi.70687. Online ahead of print. doi: 10.1111/pbi.70687 (PMC13398565; doi:10.1111/pbi.70687)
Supplement: Supplementary file 1 — Figure S1: Barcode Rank Plot. Figure S2: Cluster analysis of scRNA‐seq. Figure S3: UMAP visualisation of 19 cell clusters. Figure S4: Three specifically expressed genes in each of 19 clusters. Figure S5: Expression levels of the VC marker genes in 19 clusters. Figure S6: Localisation of specifically expressed genes in cluster 0 and cluster 1. Figure S7: Proportion of four celltype in O. pumila leaves. Figure S8: UMAP visualisation of transcript accumulation of cell type‐specific expressed genes and experimental validation by ISH. Figure S9: 100 genes with the most significant differences in expression over the pseudotime trajectory of EC subclusters. Figure S10: KEGG pathway analysis of genes in module1. Figure S11: KEGG pathway analysis of genes in module 2. Figure S12: Candidate CPT biosynthesis function genes expression changed with the pseudotime time. Figure S13: UMAP visualisation of genes on gene cluster. Figure S14: SDS‐PAGE of purfied OpTDCs and OpSTRs on gene cluster. Figure S15: Catalytic activity of OpTDC3, OpTDC4 and OpTDC5 were detected by HPLC. Figure S16: MS spectrums of OpSTR2 catalytic products. Figure S17: Phylogenetic analysis of OpAVT1. Figure S18: Protein motifs of AtAVTs and OpAVTs. Figure S19: Yeast growth complementation assay validating amino acid transport function of OpAVT1. Figure S20: Identification of positive OpAVT1‐KOs transgenic hairy root lines. Figure S21: Biomass accumulation and camptothecin yield in OpAVT1‐KOs transgenic hairy root lines. Figure S22: Detection of other free amino acids in the OpAVT1‐KOs transgenic hairy root lines. Figure S23: Time‐course analysis of OpAVT1‐KOs transgenic hairy root lines during hairy root suspension culture. Figure S24: Metabolome detection in the OpAVT1‐KO1 transgenic hairy root line. Figure S25: Transcriptomic analysis of the OpAVT1‐KO1 transgenic hairy root line. [file PBI-9999-0-s001.docx]

Supplementary Materials for

**Cell-specific expression and cellular compartmental regulation in camptothecin biosynthesis**

Xiaolong Hao^1#^, Yinkai Yang^1#^, Tiantian Chen^2#^, Xiaoxuan Fan^1#^, Yiqing Peng^1^, Qingyan Ruan^1^, Qin Zhou^1^, Fanghao Liu^1^, Jiayi He^1^, Yongpeng Li^1^, Yue Feng^1^, Jiyan Qi^2*^, Guoyin Kai^1*^

^1^ Zhejiang Provincial TCM Key Laboratory of Chinese Medicine Resource Innovation and Transformation, Zhejiang International Science and Technology Cooperation Base for Active Ingredients of Medicinal and Edible Plants and Health, Jinhua Academy, School of Pharmaceutical Sciences, Zhejiang Chinese Medical University, Hangzhou, 310053, China

^2^ Shaanxi Key Laboratory of Qinling Ecological Intelligent Monitoring and Protection, School of Ecology and Environment, Northwestern Polytechnical University, Xi’an 710129, China

# These authors contributed equally to this work

* Correspondence author:

Prof. Guoyin Kai: kaiguoyin@163.com; Prof. Jiyan Qi: jyqi@nwpu.edu.cn

**Running title**

Cell-Specific Biosynthesis Regulation of Camptothecin

**Supplementary Methods**

**Plant materials**

1. *pumila* was collected from Fujian Province (Fujian, China) in 2006^1^. It was cultured in growth chambers at Zhejiang Chinese Medical University (Zhejiang, China) at 26 degree Celsius under a 16 h light photoperiod^2^. The seeds were collected and sterilized with 75% alcohol and 5% sodium hypochlorite and then spread flat on B5 medium. Two-months seedlings after germination were taken for subsequent experiments. The stem segments of *O. pumila* were infected by *Agrobacterium tumefaciens* C58C1 to obtain transgenic hairy root lines.

**RNA extraction and gene cloning**

*O. pumila* plants were collected, quick-frozen in liquid nitrogen and ground into powder. Total RNA was extracted and reverse-transcribed into cDNA using the RNApure Plant Kit and FastKing RT Kit, respectively (Tiangen, China). The target genes were cloned by nested PCR using high-Fidelity DNA polymerase KOD-Plus (Toyobo, Japan). The purified target dna fragments were ligated into the pLB intermediate vector, and positive single clones were selected after transformation with TOP10 *E*. *coli*. After successful sequencing alignment, the strains were saved for subsequent construction of other recombinant plasmids. The specific experimental procedure was performed as previously reported^3,4^.

**Correlation analysis of transcription factors with camptothecin biosynthetic pathway genes**

To analyze the co-expression relationships, we extracted transcriptome data for all *Ophiorrhiza pumila* transcription factors along with eleven camptothecin biosynthetic pathway genes. Pearson correlation coefficients were calculated to assess the pairwise relationships between each TFs and the camptothecin pathway genes, generating both correlation coefficients (R) and corresponding p-values. Candidate genes were selected using stringent thresholds: absolute R values >0.8 and statistical significance (p<0.05). The resulting co-expression network between qualifying TFs and the eleven structural genes was constructed and visualized using Cytoscape (v3.7.2), where various layout parameters were adjusted to optimize network representation.

**Protein expression and detection of catalytic products**

The CDS sequences of the target genes were cloned using *O. pumila* cDNA as a template. The target genes were constructed into the prokaryotic expression vector *His-pCold-TF* and transferred into BL21 (DE3) *E. coli* strain. Positive strains were inoculated into LB liquid medium containing 100 mM AMP antibiotic and incubated with shaking until OD600=0.6 and induced for 12 h at 16 °C after addition of 0.5 mM isopropyl-β-D-thiogalactoside (IPTG) to to produce protein^5^. The cells were resuspended in PBS buffer (pH=8), broken by sonication and centrifuged to remove the supernatant. The recombinant proteins were purified by His-Tag Prepacked Chromatographic Column (Sangon Biotech, Shanghai). The purified proteins were desalted with an ultrafiltration tube（Merck Millipore, Germany). 200 µl of dialysed protein was taken and 1 µl of 20 mM PLP (Pyridoxal5-phosphatemonohydrate) and 2 µl of 50 mM of the corresponding catalytic substrate were added to the system. The mixed system was incubated at 37 °C for 20 h with 200 rpm. The catalytic reaction was terminated by adding 100 µl of methanol. Catalytic sample of the OpTDCs were used for HPLC-diode array detection (HPLC-DAD) analysis after filtering through a 0.2 μm membrane filter. Agilent Eclipse Plus C18 column (3.5 μm, 4.6×150 mm ) was used on Agilent 1260 Infinity II system at 30 °C. Mobile phases A (H_2_O +0.1% formic acid) and B (acetonitrile) were used for chromatography and the gradient program of the mobile phase was as follows:10% B at 0 min; 100% B at 26 min; 10% B at 26.01 min; and 10% B at 31 min. The chromatographic peaks of catalytic substrates and products were obtained at 254 nm. Analysis of OpSTR catalytic samples by UPLC-Q-TOF/MS (Waters, SYNAPT XS model) with a ACQUITY Premier HSS T3, VanGuard FIT Colu VanGuard FIT Colu (1.8 µm, 2.1 x 150 mm). Positive ion mode was used for product detection, the flow phase and gradient program were consistent with the HPLC.

**RNA preparation and sequencing**

The wild-type and *OpAVT1-KO* hairy root lines cultured in shake flasks were stored at -80 °C. Total RNA was extracted from frozen tissue samples using TRIzol reagent according to the manufacturer's protocol. The RNA concentration and purity were determined by Nanodrop 2000 spectrophotometry, while RNA integrity was verified through both agarose gel electrophoresis and an Agilent 5300 system, ensuring all samples met the quality thresholds (RQN > 6.5, OD260/280 = 1.8-2.2). Poly(A)+ mRNA was enriched from total RNA using Oligo(dT) magnetic beads through polyA-selection. The enriched mRNA was then fragmented using divalent cations under elevated temperature to generate fragments of approximately 300 bp. Using random primers, the fragmented mRNA was reverse-transcribed into double-stranded cDNA. After end repair and dA-tailing, Illumina adapters were ligated. The library was then size-selected, PCR-amplified, and purified. Final qualified libraries were sequenced on the Illumina NovaSeq X Plus platform with a paired-end strategy.

**Supplementary Figures**


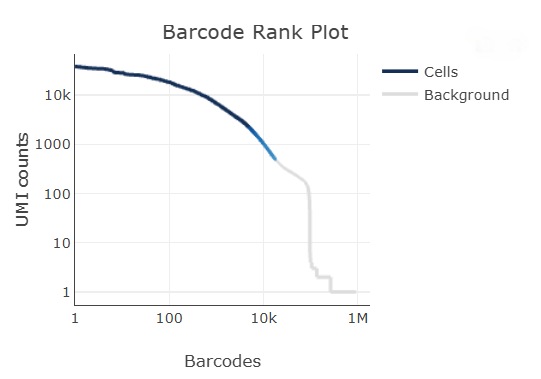


**Figure S1. Barcode Rank Plot.** The number of effective cells was quantified by barcodes and UMI counts.


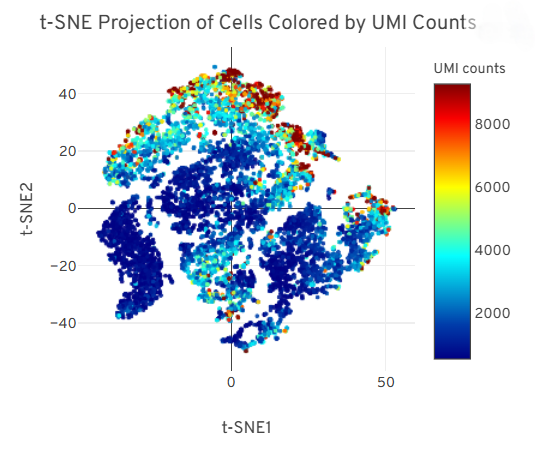


**Figure S2. Cluster analysis of scRNA-seq.** t-SNE projection of cells colored by UMI counts.


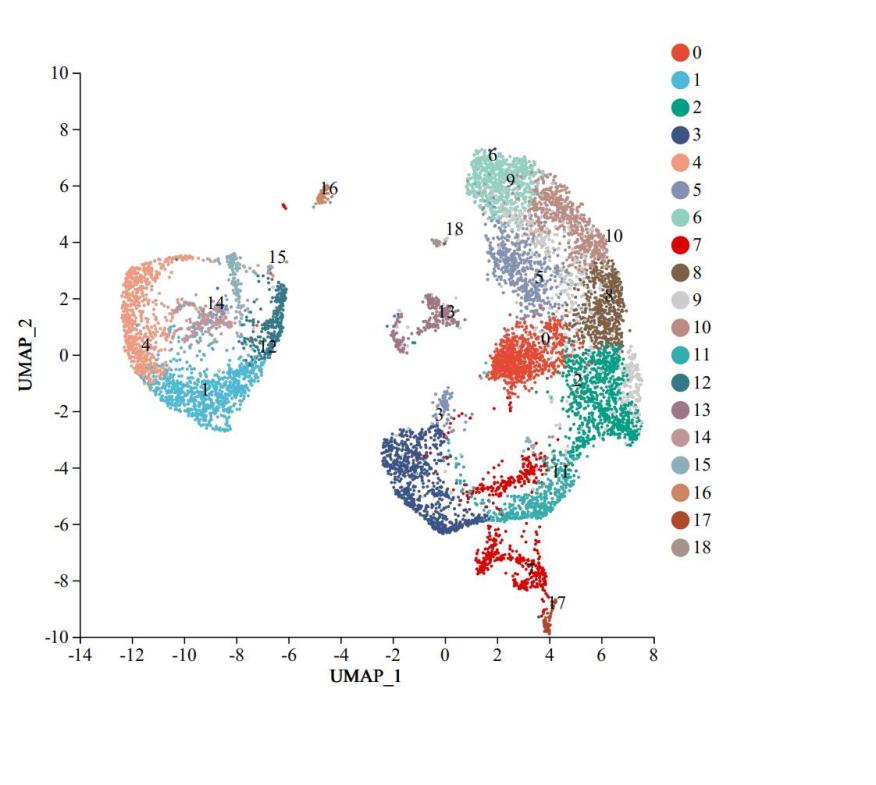


**Figure S3. UMAP visualisation of 19 cell clusters.** Dots indicate individual cells, colours indicate different cell clusters.


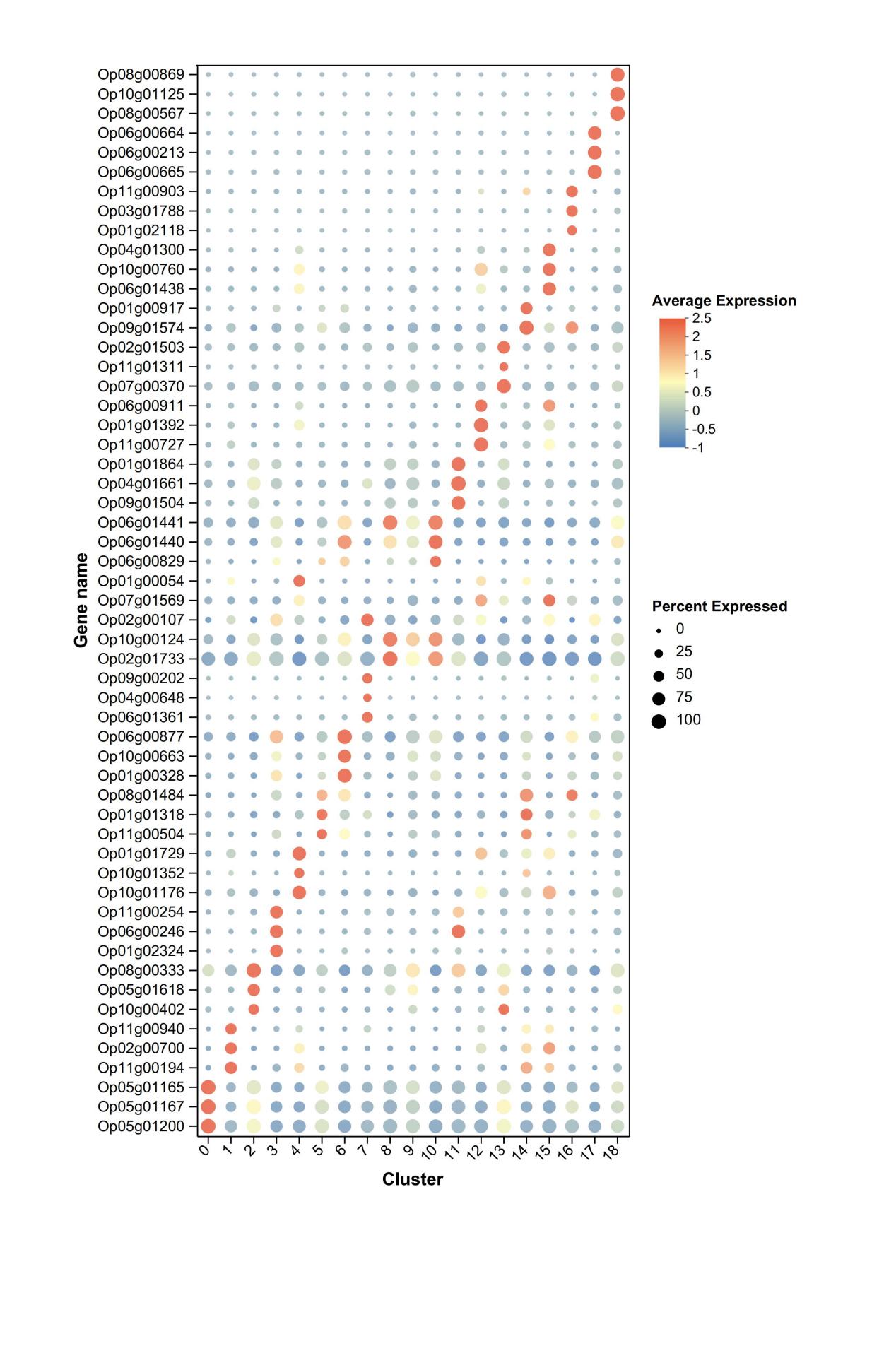


**Figure S4. Three specifically expressed genes in each of 19 clusters.** Red colour indicates high transcription levels of genes and blue colour indicates low transcription levels of genes. The larger the bubble the higher the level of transcript expression.


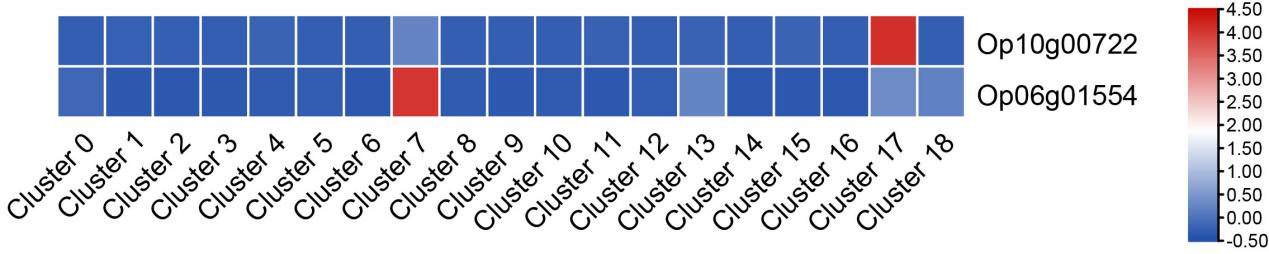


**Figure S5. Expression levels of the VC marker genes in 19 clusters.** *Op10g00722* (*CALS7*) and *Op06g01554* (*TED4*), the marker genes of the VC cell types, were highly expressed in cluster17 and cluster7. Red colour indicates high transcription levels of genes and blue colour indicates low transcription levels of genes.


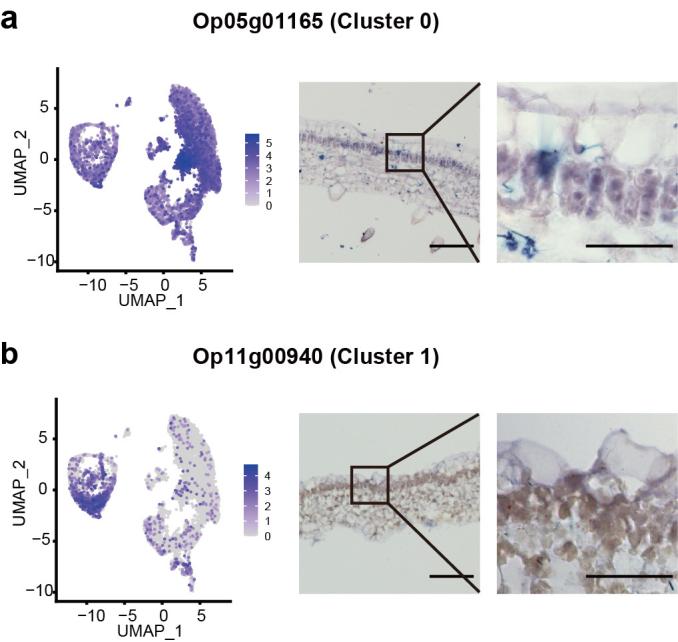


**Figure S6. Localisation of specifically expressed genes in cluster 0 and cluster 1. a-b**, UMAP visualisation of transcript accumulation and experimental validation by ISH of specifically expressed genes in (**a**), cluster 0 and (**b)**, cluster 1. The results of the ISH experiments were observed at 20x magnification, and the purplish signals are marked with black arrows.


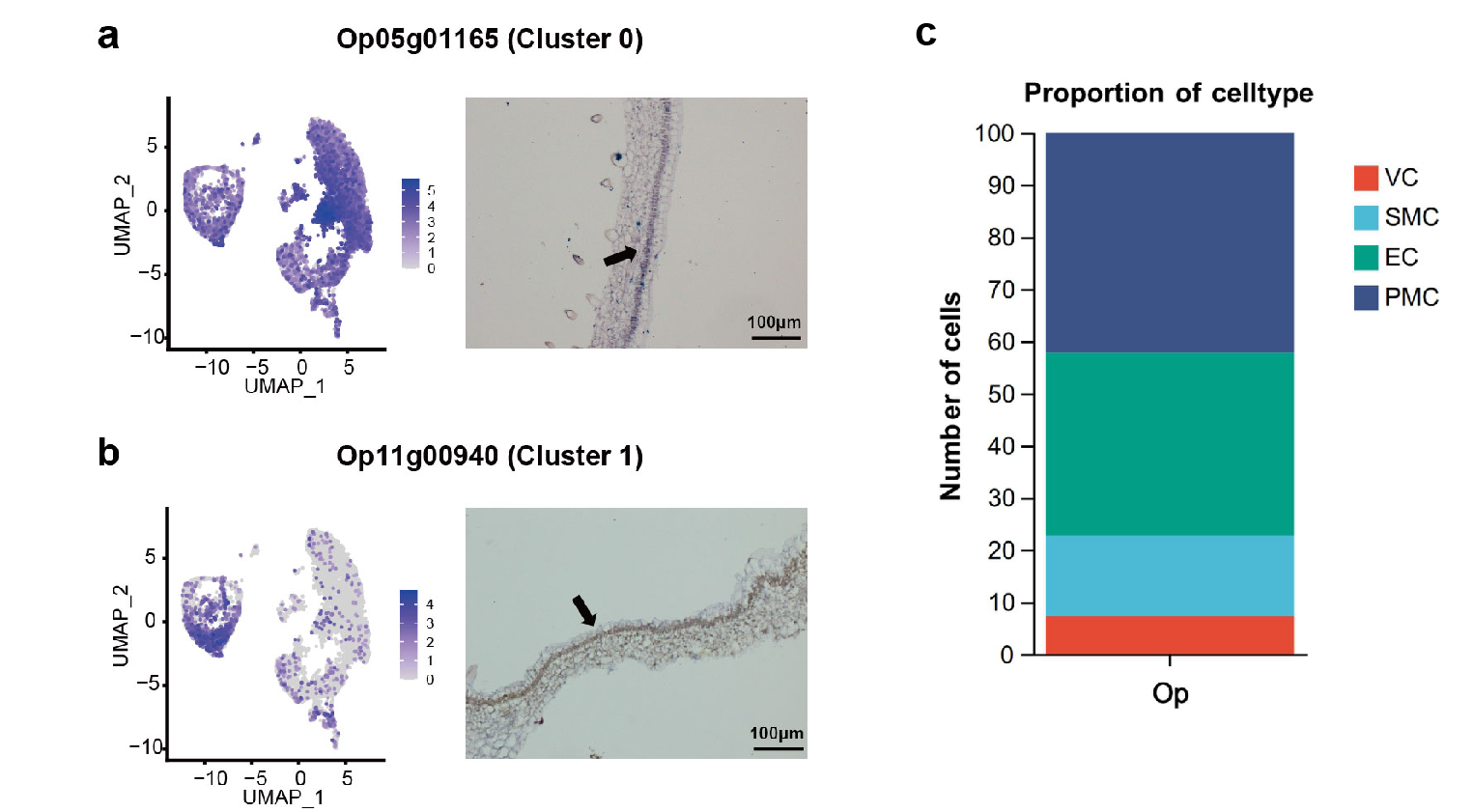


**Figure S7. Proportion of four celltype in *O. pumila leaves.*** The four colors of the histograms indicate different cell types.

**
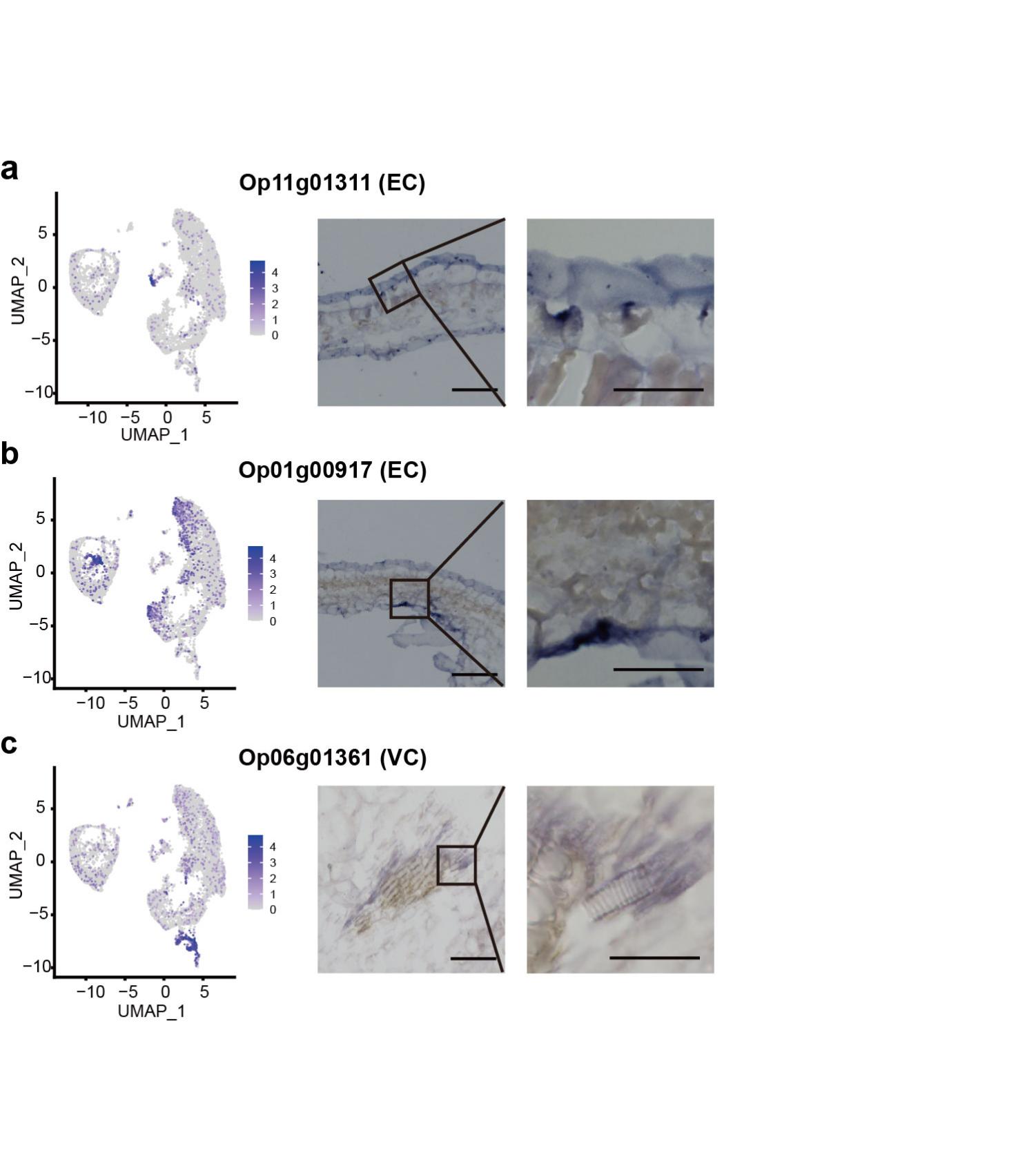
**

**Figure S8. UMAP visualisation of transcript accumulation of cell type-specific expressed genes and experimental validation by ISH.** They were *Op11g01311* and *Op01g00917* specifically expressed in EC, and *Op06g01361* in VC.


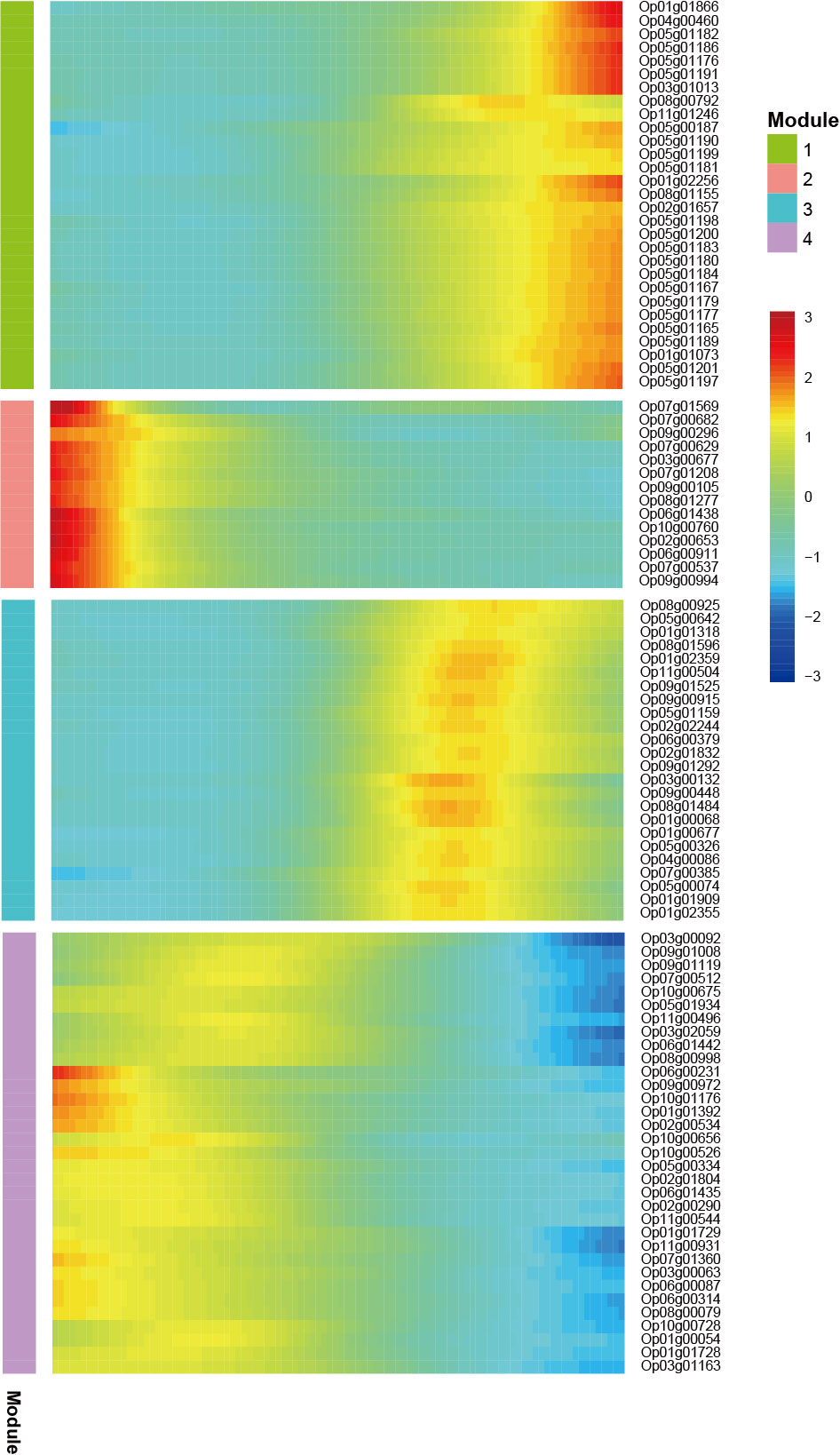


**Figure S9. 100 genes with the most significant differences in expression over the pseudotime trajectory of EC subclusters.** Each module represents a class of gene sets with consistent expression trends. Each row of the graph represents a gene, and each column represents the average expression value in the current cell state, and the color is gradually reduced from red to blue.


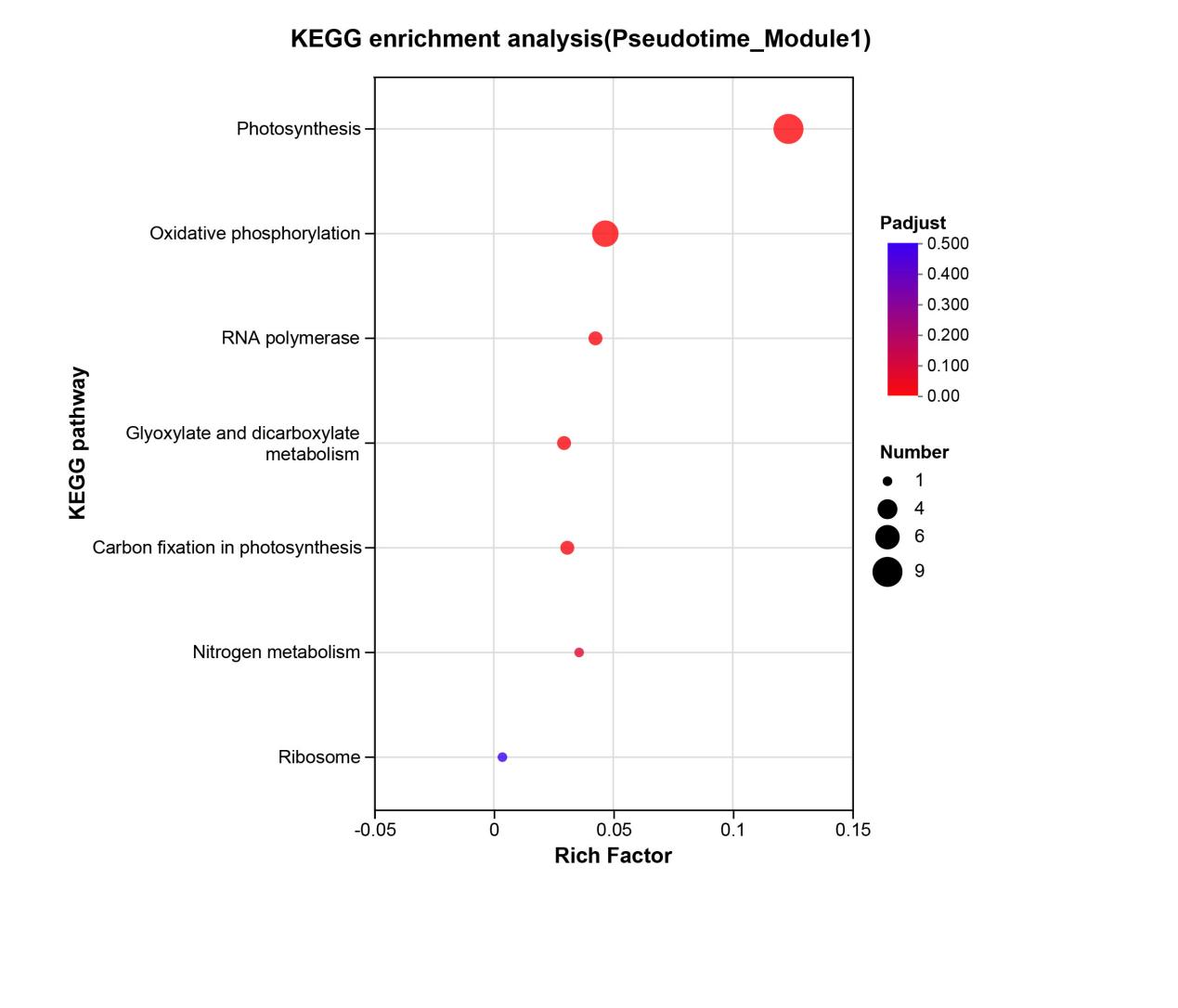


**Figure S10. KEGG pathway analysis of genes in module1.** The rich factor represents the ratio of sample number enriched in the KEGG pathway to background number. Larger rich factor indicates a greater degree of enrichment. The size of the points indicates the number of genes in this KEGG pathway, while the colour of the points corresponds to different padjust ranges.


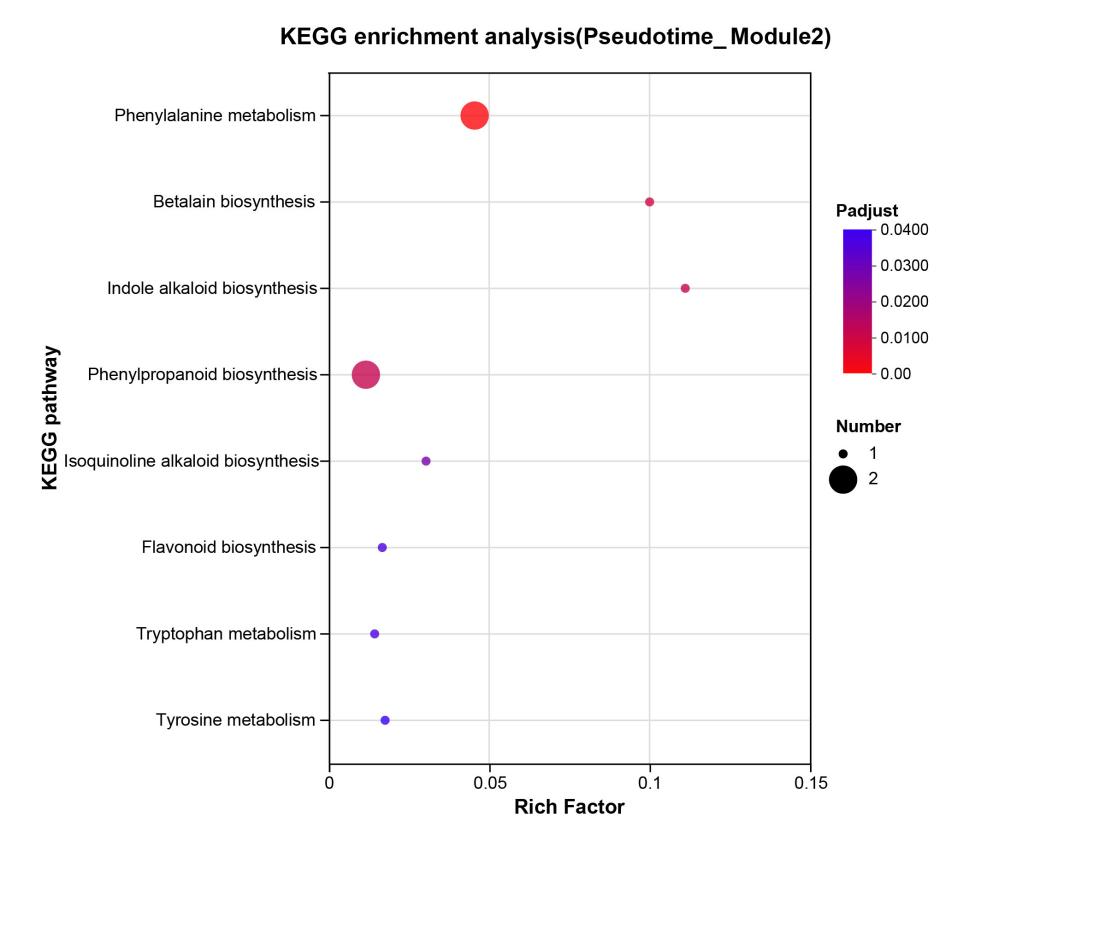


**Figure S11.** **KEGG pathway analysis of genes in module 2.** The rich factor represents the ratio of sample number enriched in the KEGG pathway to background number. Larger rich factor indicates a greater degree of enrichment. The size of the points indicates the number of genes in this KEGG pathway, while the colour of the points corresponds to different padjust ranges.


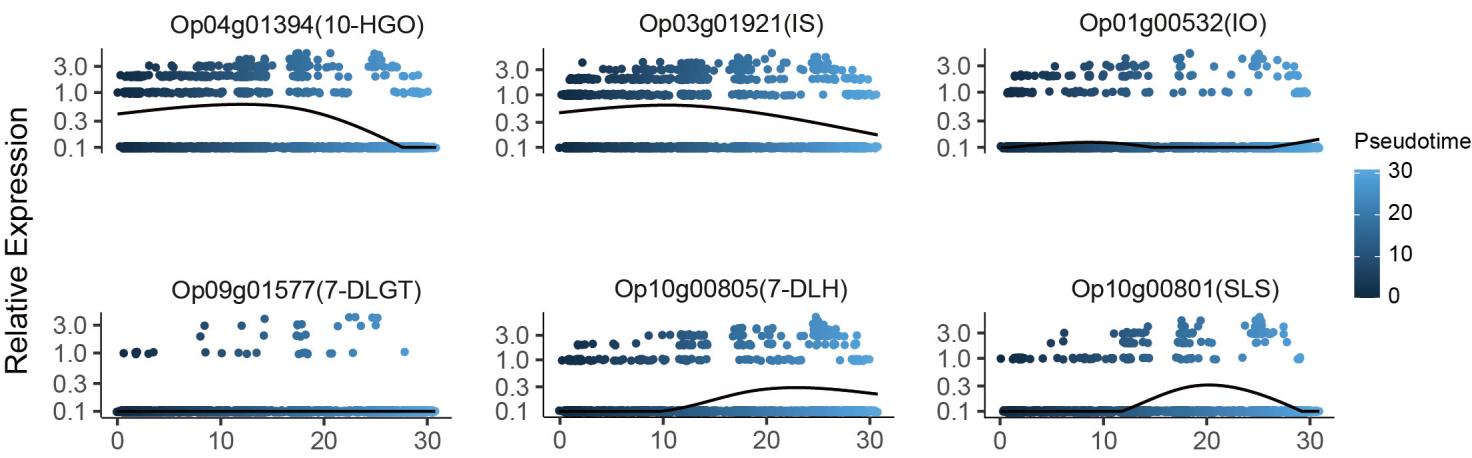


**Figure S12. Candidate CPT biosynthesis function genes expression changed with the pseudotime time.** The deepest colour were the starting position.


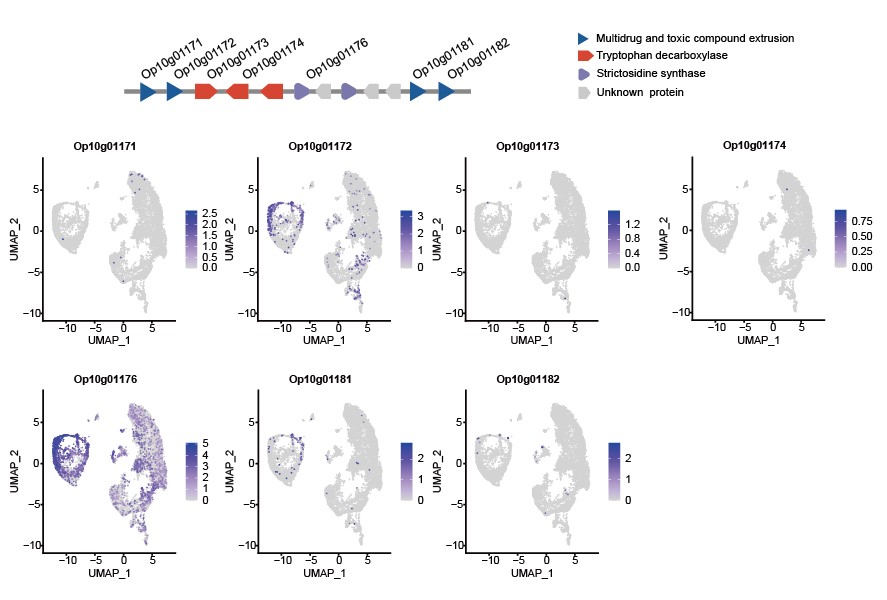


**Figure S13.** **UMAP visualization of genes on gene cluster.** Schematic representation of the arrangement of genes on gene clusters and UMAP plots showing the transcript accumulation of genes in gene cluster. Color intensity indicates the relative transcript level for the indicated gene in each cell.


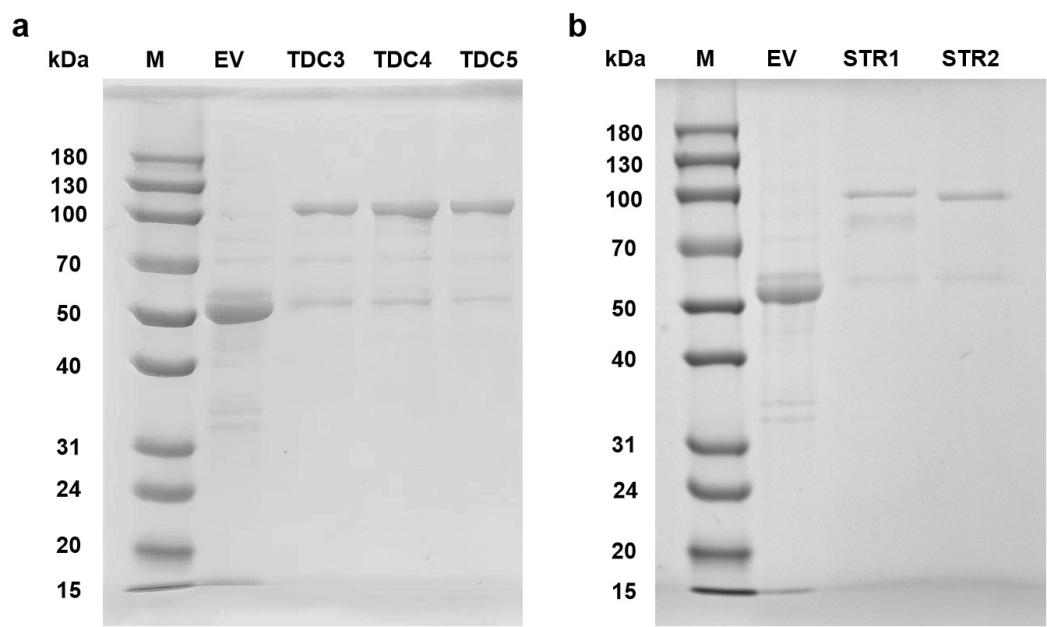


**Figure S14. SDS-PAGE of purfied OpTDCs and OpSTRs on gene cluster. a**, Purified His-tagged OpTDC3, OpTDC4, OpTDC5 from *O. pumila*. **b**, Purified His-tagged OpSTR1 and OpSTR2 from *O. pumila*. The recombinant plasmid was transferred into BL21 *E. coli* and crude protein was induced by adding 0.5 mM IPTG. After purification, the expression and purification of the target protein was detected using SDS-PAGE protein electrophoresis assay. M: Protein marker. EV, empty vector.


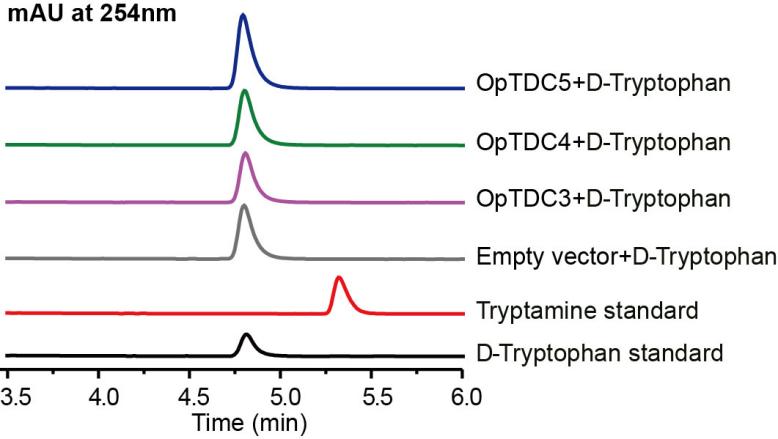


**Figure S15. The catalytic activity of OpTDC3, OpTDC4 and OpTDC5 were detected by HPLC.** D-tryptophan was used as the catalytic substrate and the catalytic product was tryptamine. The detection wavelength was 254 nm.


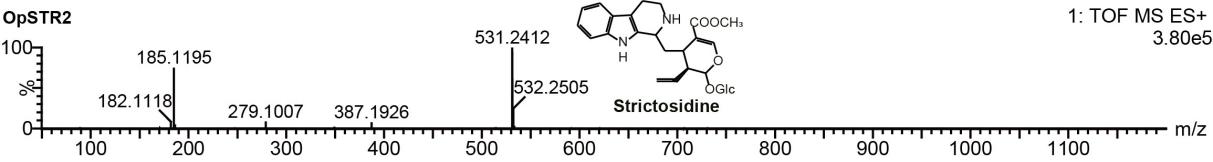


**Figure S16. MS spectrums of OpSTR2 catalytic products.** The catalytic product is strictosidine.


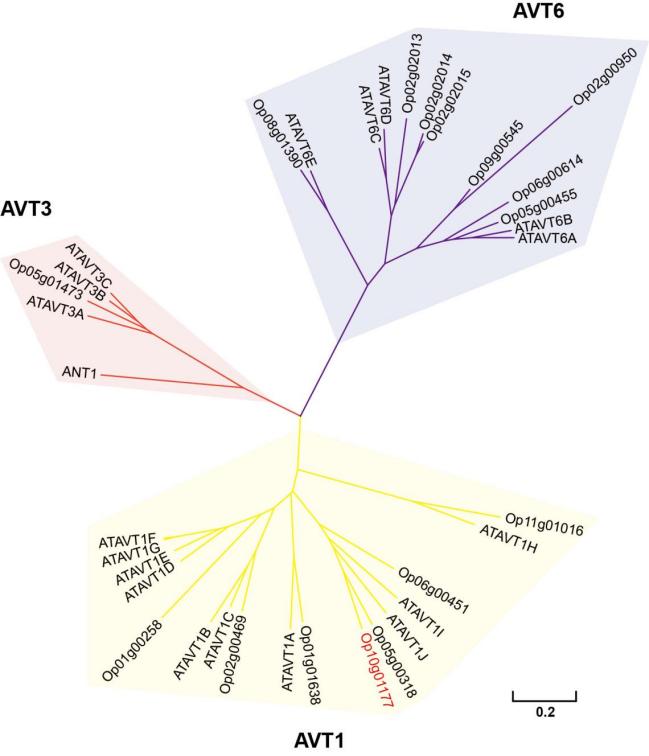


**Figure S17. Phylogenetic analysis of *OpAVT1*.** Based on the classification of the Arabidopsis thaliana *AVT* family, the results revealed that *OpAVT* can also be divided into three families, namely *AVT1*, *AVT3*, and *AVT6*. Among them, *Op10g01177* (*OpAVT1*) belongs to the *AVT1* family.


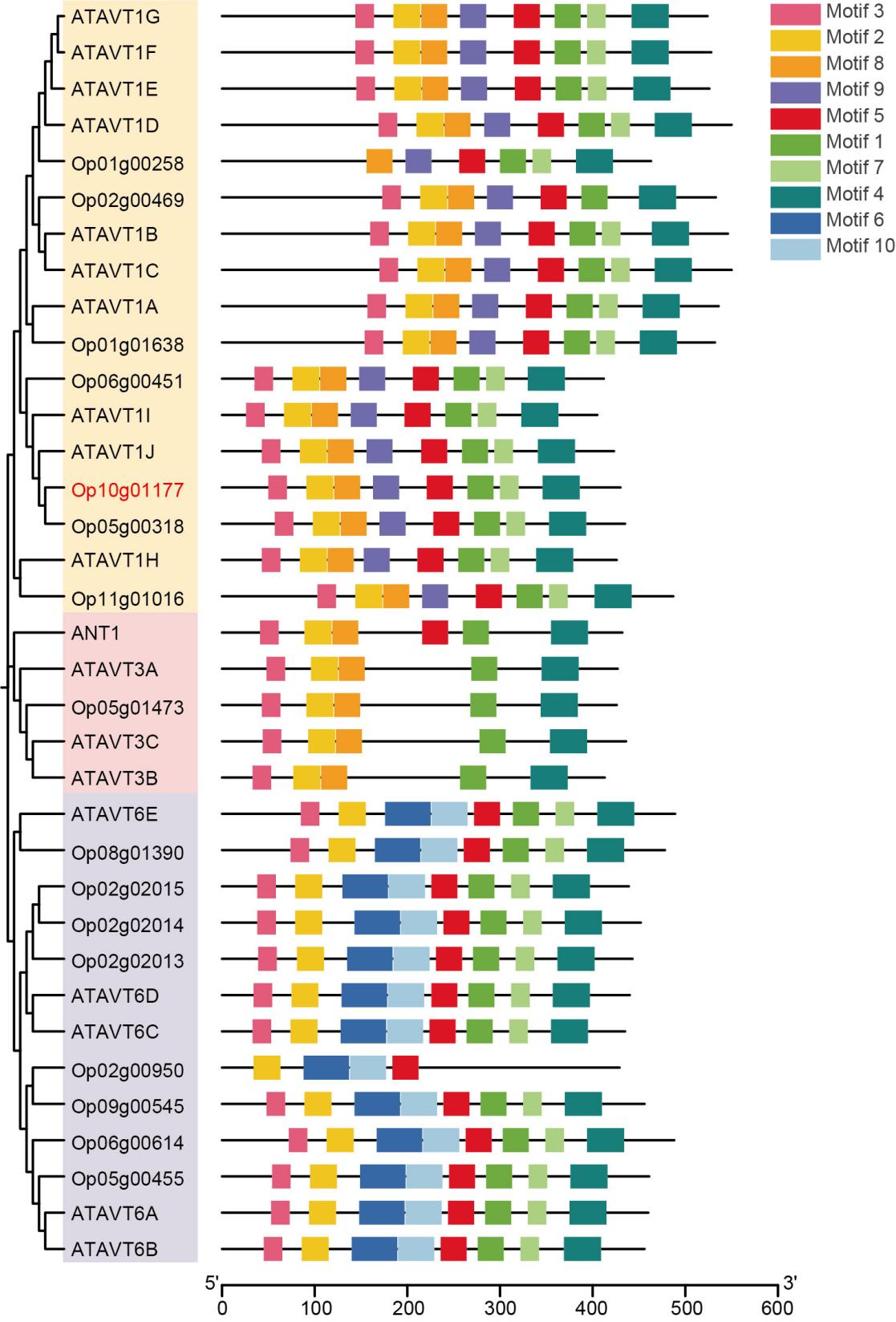


**Figure S18. The protein motifs of AtAVTs and OpAVTs.** The specific motifs in proteins were identified using the MEME motif search tool. There are differences in the conserved domains among the three AVT gene families. The family to which OpAVT1 belongs contains one additional motif (Motif 9) compared to the other two familie.


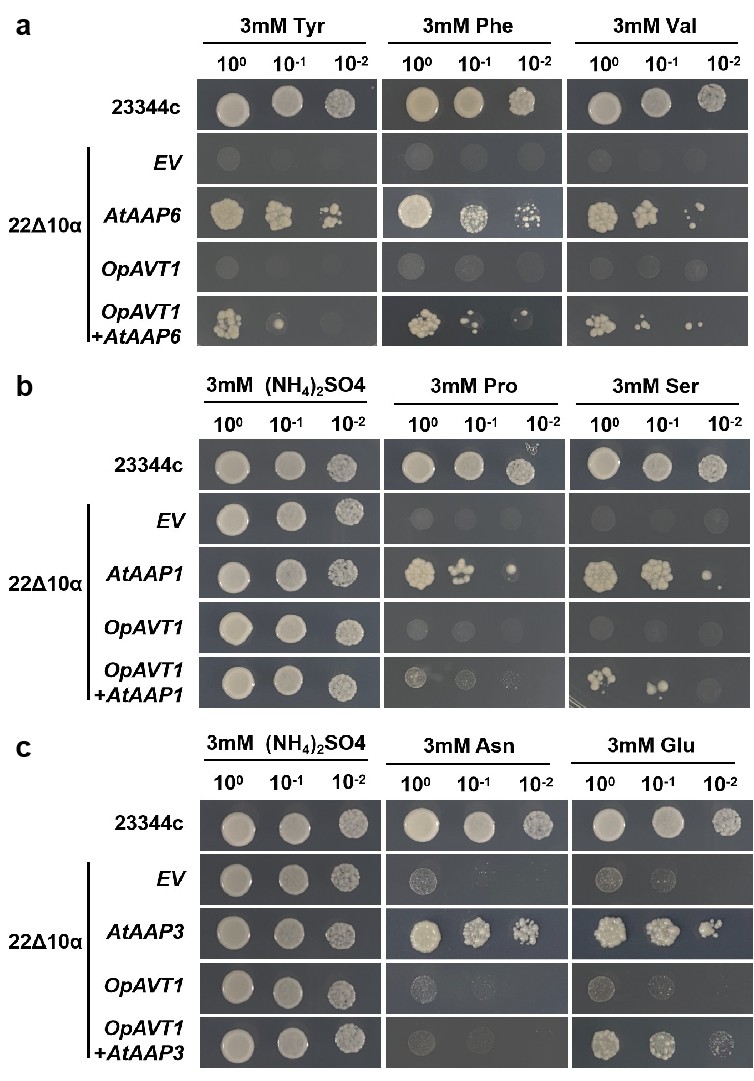


**Figure S19. Yeast growth complementation assay validating amino acid transport function of OpAVT1.** The AtAAP1/AtAAP3 and OpAVT1 genes were individually or co-transformed into the *Saccharomyces cerevisiae* strain 22Δ10α. Transformants were serially diluted (10^0^, 10^-1^, 10^-2^) and spotted onto selective media with ammonium sulfate or amino acid as the sole nitrogen source. Co-expression of OpAVT1 and AtAAP1 demonstrated its transport function for proline and serine, while co-expression of OpAVT1 and AtAAP3 confirmed its transport capability for asparagine and glutamic acid.

**
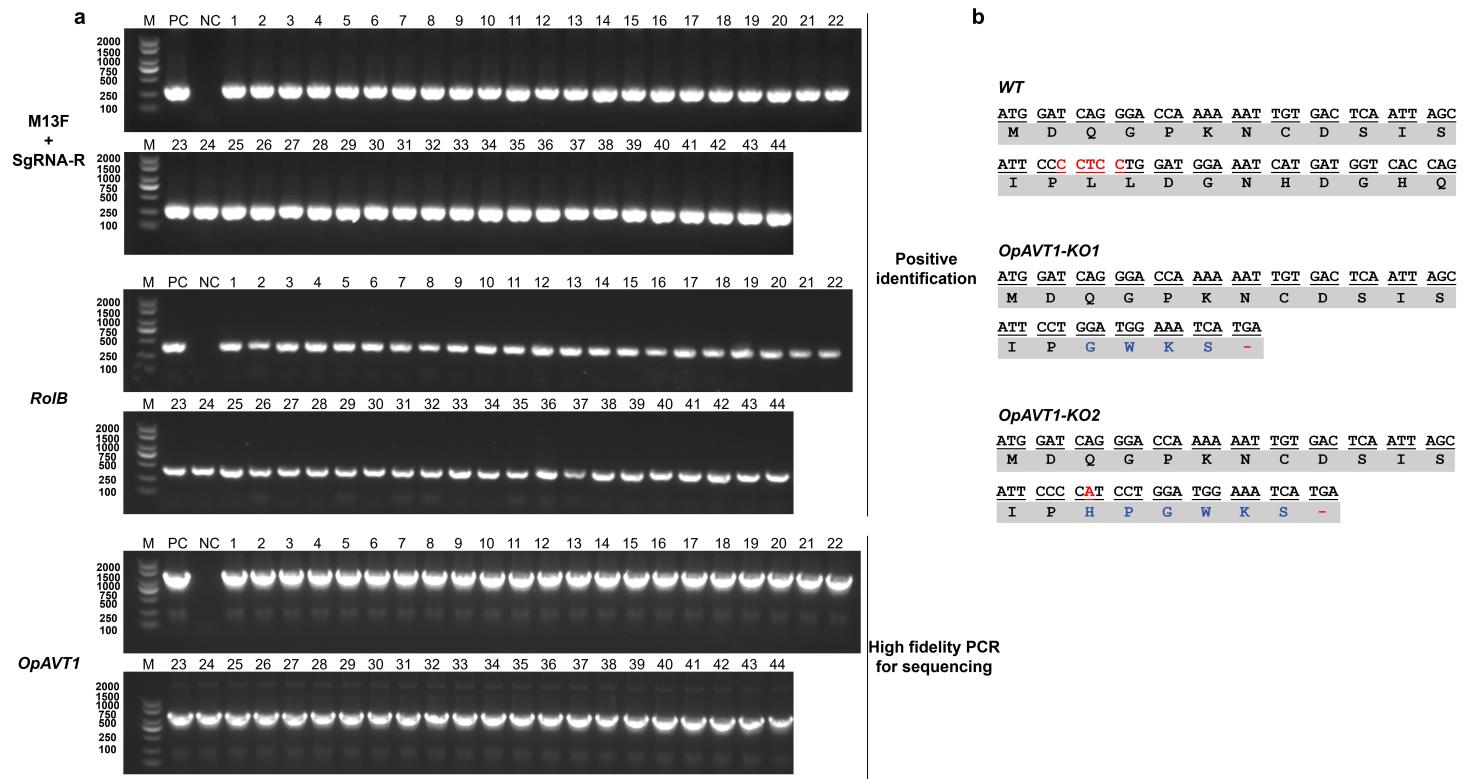
**

**Figure S20. Identification of positive *OpAVT1-KO* transgenic hairy root lines.** **a**, The presence of transgenic clones was confirmed by PCR amplification using the *M13-F* and *SgRNA-R* primer pair. Integration of the T-DNA was verified by detecting the rolB gene, serving as a marker for successful Agrobacterium rhizogenes-mediated transformation. To validate the genetic modifications in knockout lines, the OpAVT1 locus was amplified from genomic DNA using high-fidelity PCR and subsequently subjected to sequencing. M, Marker. PC, Positive Control. NC, Negative Control. b, Two homozygous knockout lines *OpAVT-KO-1/2* exhibited premature termination of the amino acid sequence at the 19th and 21st residues, respectively.

**
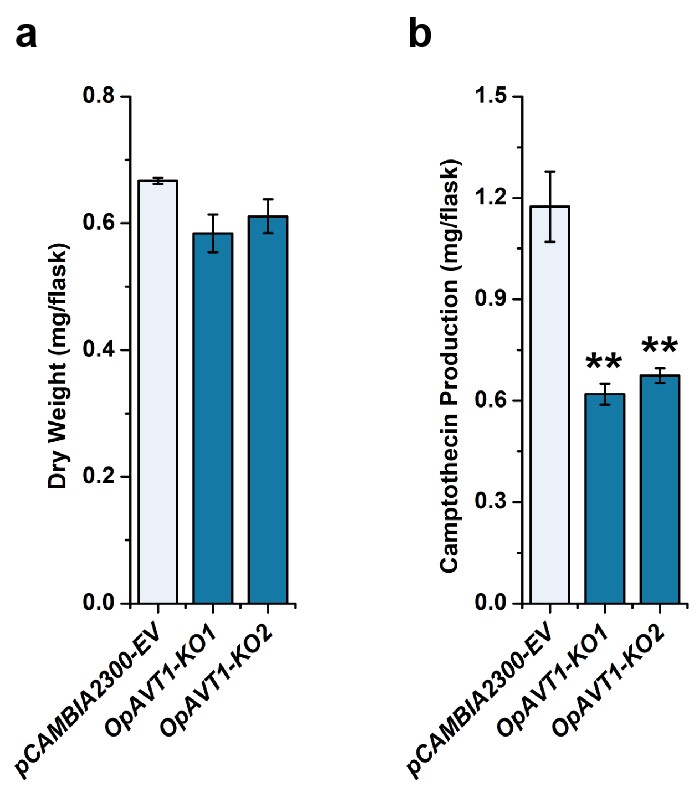
**

**Figure S21. Biomass accumulation and camptothecin yield in *OpAVT1-KO* transgenic hairy root lines.** **a**, Dry weight of hairy root lines after 35 days of culture. **b**, Camptothecin (CPT) content measured by HPLC. Data are presented as mean ± SD (n = 3). Statistical significance between the WT and each KO line was determined by Student's t-test (***p* < 0.01).


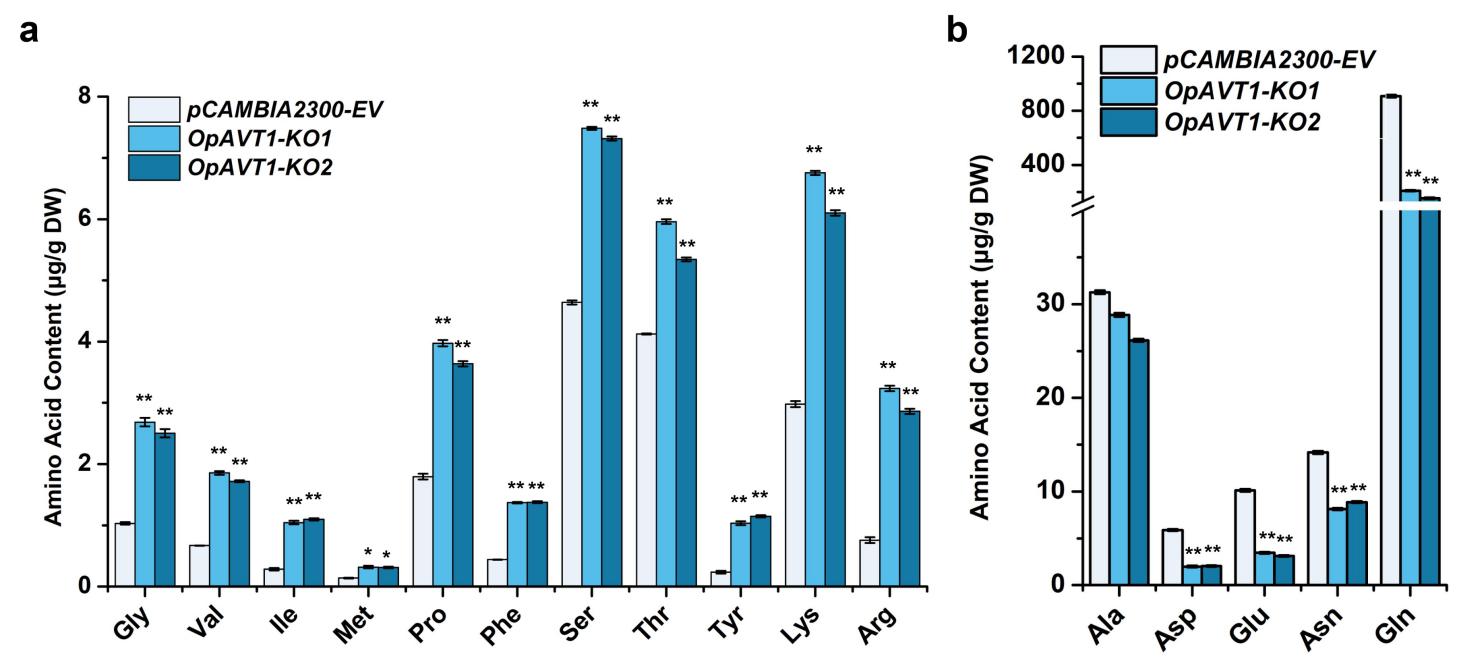


**Figure S22. Detection of other free amino acids in the *OpAVT1-KOs* transgenic hairy root lines. a-b**, The free amino acids that exhibited a significant increase (**a**) or decrease (**b**) in content are shown in separate bar graphs. Error bars represent the SD of three biological replicates. Asterisks indicate statistically significant differences (**p* < 0.05, ***p* < 0.01) compared to the wild-type control.


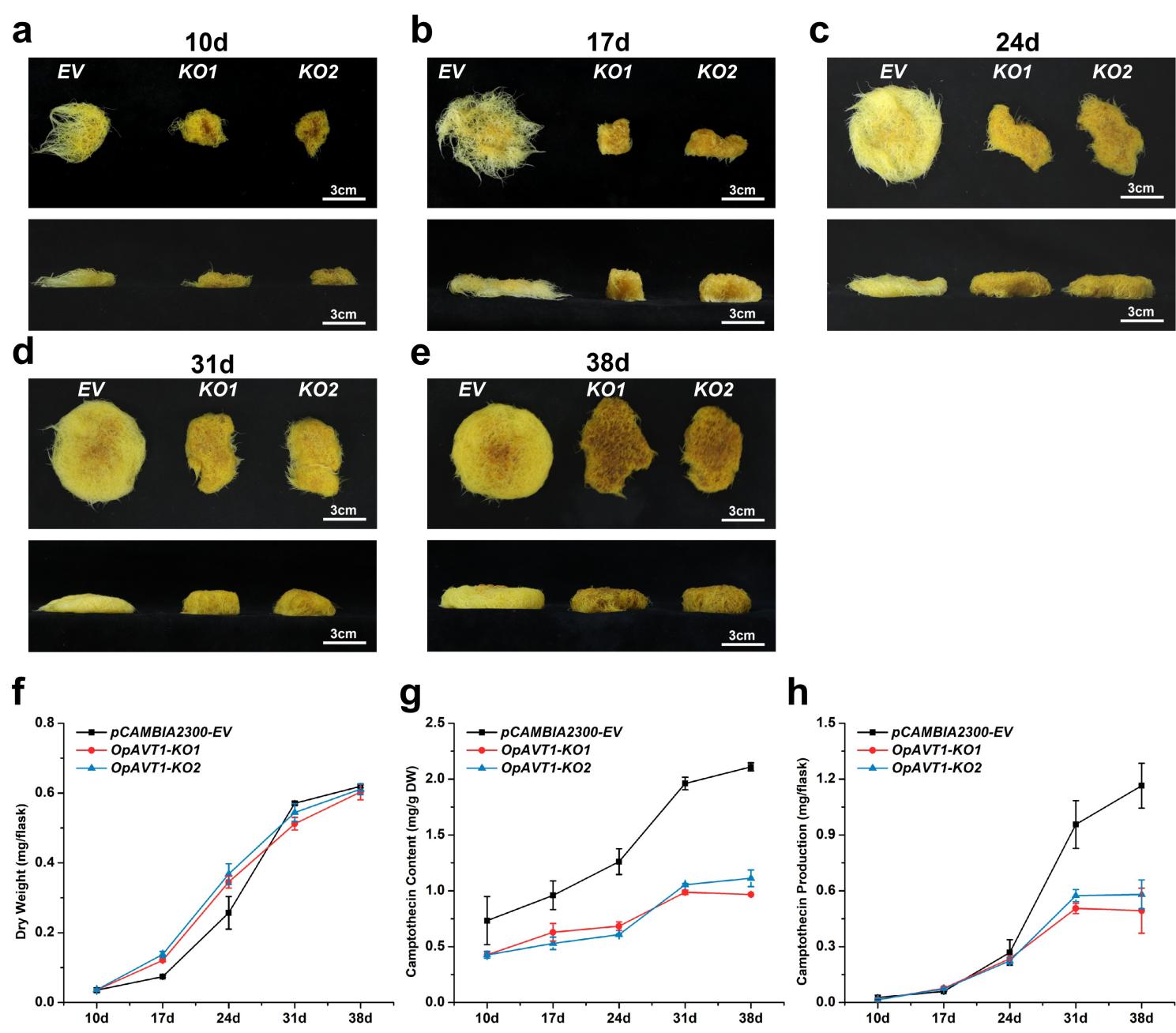


**Figure S23. Time-course analysis of *OpAVT1-KOs* transgenic hairy root lines during hairy root suspension culture. a-e**, Phenotypic records of empty vector control and *OpAVT1-KOs* lines cultured in shake flasks at 10, 17, 24, 31, and 38 days, respectively. Scale bars: 3 cm. **f-h**, Dry weight, camptothecin content, and camptothecin production were measured in empty vector control and *OpAVT1-KOs* lines at 10, 17, 24, 31, and 38 days of shake flask culture.


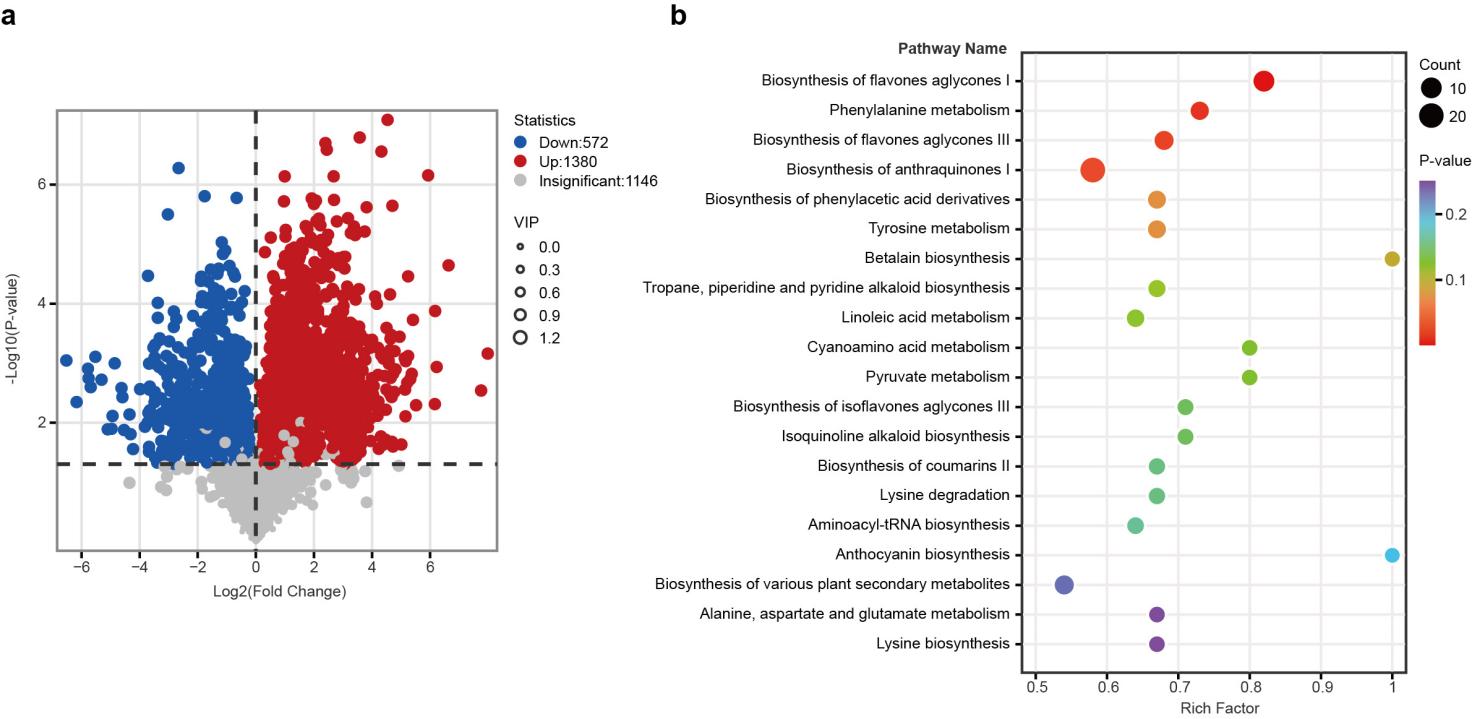


**Figure S24. Metabolome detection in the *OpAVT1-KO1* transgenic hairy root line.** **a**, A total of 3,098 metabolites were detected in the metabolomic analysis. Among them, 1,380 metabolites showed significantly increased abundance, while 572 metabolites exhibited significantly decreased abundance. Each point represents a metabolite. Significantly upregulated and downregulated metabolites are highlighted in red and blue, respectively. **b**, The rich factor represents the degree of enrichment, the size of the dots corresponds to the number of DEGs mapped to the pathway, and the color indicates the range of the adjusted P-value.


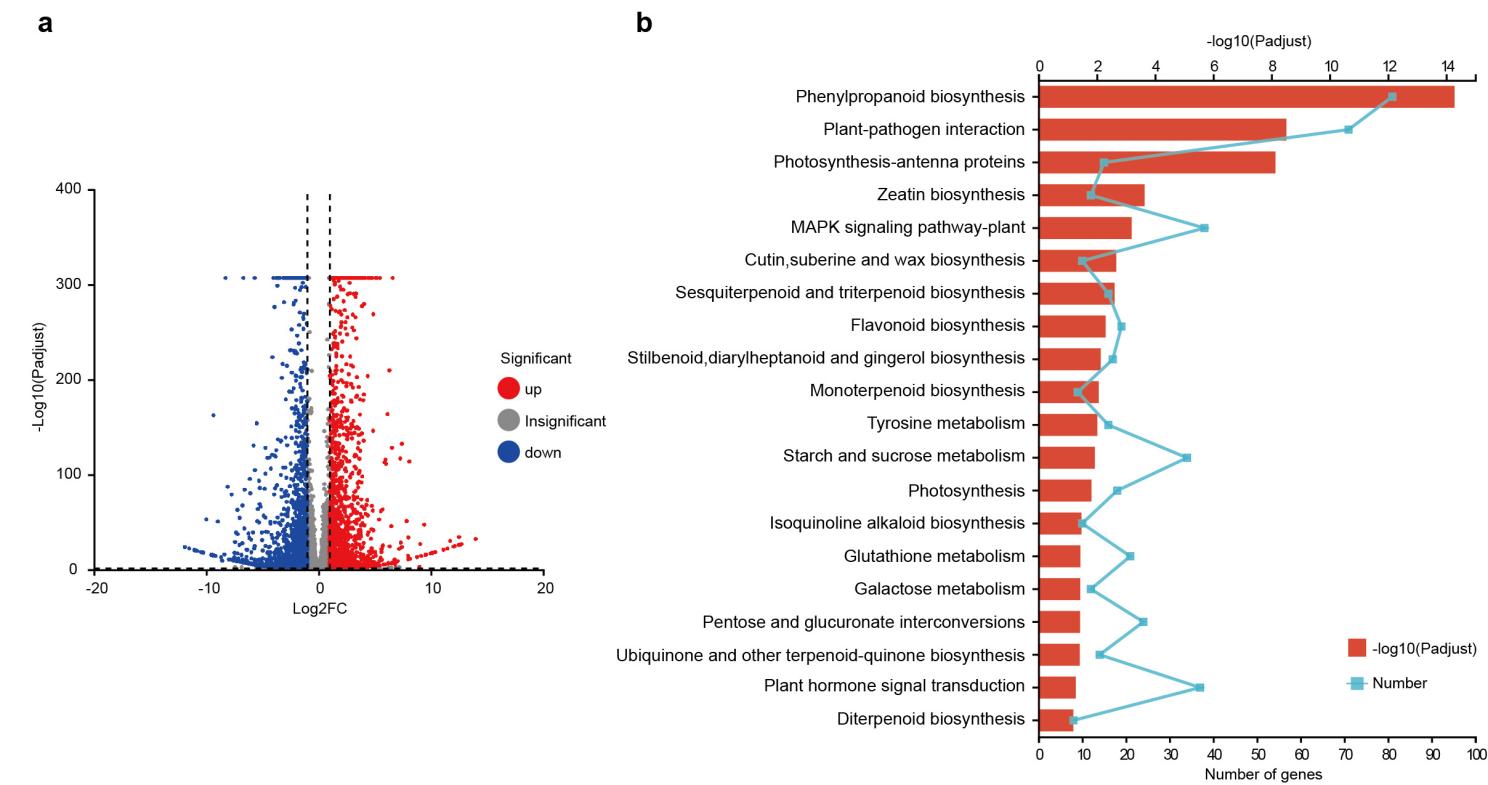


**Figure S25. Transcriptomic analysis of the *OpAVT1-KO1* transgenic hairy root line. a**, A total of 3,244 differentially expressed genes were detected, including 1,676 significantly upregulated genes and 1,568 significantly downregulated genes. Each point represents a gene. Significantly upregulated and downregulated genes are highlighted in red and blue, respectively. **b**, The y-axis represents the enriched KEGG pathways. The lower x-axis corresponds to the number of differentially expressed genes mapped to a given pathway (gene number), which is associated with the data points on the curve. The upper x-axis indicates the enrichment significance, expressed as -log_10_(Padjust), which corresponds to the height of the bars.

**References**

1 Hao, X. et al. The transcription factor OpWRKY2 positively regulates the biosynthesis of the anticancer drug camptothecin in *Ophiorrhiza pumila*. *Hortic. Res.* **8**, 7 (2021).

2 Hao, X. et al. OpNAC1 transcription factor regulates the biosynthesis of the anticancer drug camptothecin by targeting loganic acid O-methyltransferase in *Ophiorrhiza pumila*. *J. Integr. Plant Biol.* **65**, 133-149(2023).

3 Hao, X. et al. Tanshinone and salvianolic acid biosynthesis are regulated by SmMYB98 in Salvia miltiorrhiza hairy roots. *J. Adv. Res.* **23**, 1-12(2020).

4 Yang, Y. et al. Jasmonic acid responsive AaJRM1 transcription factor positively regulates the biosynthesis of anti-malarial drug artemisinin in Artemisia annua. *Ind. crop prod.* **199**, (2023).

5 Zhou, W. et al. A chromosome-level genome assembly of anesthetic drug-producing Anisodus acutangulus provides insights into its evolution and the biosynthesis of tropane alkaloids. *Plant commun.* **5**, (2024).
