# Supplementary material for: Cell‐Specific Expression and Cellular Compartmental Regulation in Camptothecin Biosynthesis
Source: Plant Biotechnol J. 2026 May 26:10.1111/pbi.70687. Online ahead of print. doi: 10.1111/pbi.70687 (PMC13398565; doi:10.1111/pbi.70687)
Supplement: Supplementary file 2 — Table S1: Statistical data of scRNA‐seq. Table S2: Cell quality assessment and filtration. Table S3: Number of cells in each cluster. Table S4: Top 10 maker genes of each cluster. Table S5: Marker genes from Arabidopsis thaliana and Catharanthus roseus leaves. Table S6: Marker genes of two types of Mesophyll cell from Arabidopsis thaliana leaves. Table S7: Transcript accumulation of cell type‐specific expressed genes. Table S8: Correlation analysis of transcription factors with functional genes for camptothecin biosynthesis. Table S9: Metabolomic data of the camptothecin pathway in OpAVT1‐KO1 hairy root line. Table S10: Hormone profiling data of OpAVT1‐KO1 hairy root line. Table S11: Primer used in this study. [file PBI-9999-0-s002.pdf]

Table S1. Statistical data of scRNA-seq.

Sample	Op_W1
Estimated Number of Cells	11,835
Mean Reads per Cell	32,234
Median Genes per Cell	916
Valid Barcodes	97.40%
Fraction Reads in Cells	58.40%
Sequencing Saturation	47.60%
Reads Mapped to Genome	93.50%
Reads Mapped Confidently to Exonic Regions	28.20%
Number of Reads	381,487,534
Q30 Bases in Barcode	95.80%
Q30 Bases in RNA Read	89.00%
Q30 Bases in UMI	95.30%
Reads Mapped Confidently to Genome	75.30%
Reads Mapped Confidently to Transcriptome	29.60%
Reads Mapped Antisense to Gene	1.10%
Total Genes Detected	19,290
Median UMI Counts per Cell	1,740

Table S2. Cell quality assessment and filtration.

Sample	nCount_RNA	nFeature_RNA	Percent_double_cell	Num_Orig	Num_filterd
Op_W1	7186	200-2812	7.92%	10924	9181

Table S3. Number of cells in each cluster.

cluster	Op_W1
0	885
1	872
2	853
3	796
4	685
5	650
6	625
7	597
8	566
9	549
10	549
11	441
12	279
13	245
14	222
15	187
16	75
17	72
18	33
sum	9181

Table S4. Top 10 maker genes of each cluster.

Clusters	Gene id	avg_Log2FC	Pvalue	Padjust	pct.1(%)	pct.2(%)	Significant	Regulate
0	Op05g01200	2.921141383	0	0	99.3	83.6	yes	up
0	Op05g01165	2.859905004	0	0	98.1	65.2	yes	up
0	Op05g01167	2.859629323	0	0	99.5	74.1	yes	up
0	Op05g01197	2.852764399	0	0	97.6	59.8	yes	up
0	Op05g01198	2.84924807	0	0	99.4	80.9	yes	up
0	Op05g01186	2.818641208	0	0	82.3	35.3	yes	up
0	Op05g01199	2.770980352	0	0	99.9	96.4	yes	up
0	Op05g01201	2.7609898	0	0	94.2	55.5	yes	up
0	Op05g01190	2.747696602	0	0	99.7	89.1	yes	up
0	Op05g01184	2.739122707	0	0	98.9	78.2	yes	up
0	Op05g01180	2.701271964	0	0	99.5	79.1	yes	up
0	Op05g01177	2.666253794	0	0	98.5	71.8	yes	up
0	Op05g01179	2.595045207	0	0	96.9	60.4	yes	up
0	Op05g01181	2.565921474	0	0	99.7	95.4	yes	up
0	Op05g01176	2.456979081	7.06E-267	1.29E-262	75.6	33.8	yes	up
0	Op05g01183	2.446908294	0	0	97.3	69.4	yes	up
0	Op05g01185	2.442768034	8.32E-260	1.52E-255	79.1	40.6	yes	up
0	Op05g01182	2.384970726	1.25E-290	2.29E-286	79.1	35.4	yes	up
0	Op05g01163	2.375685447	3.92E-180	7.17E-176	61.8	27	yes	up
0	Op05g01164	2.356232617	1.52E-184	2.78E-180	58.8	23.5	yes	up
0	Op05g01189	2.32221563	8.65E-246	1.58E-241	73.9	33.8	yes	up
0	Op01g01073	2.319954742	9.65E-215	1.77E-210	63.8	25.2	yes	up
0	Op03g01013	2.213569436	2.34E-186	4.29E-182	68.1	33.9	yes	up
0	Op05g01196	2.202126489	2.42E-141	4.43E-137	48.8	18.8	yes	up
0	Op05g01191	2.181851708	2.11E-191	3.86E-187	64.6	29.4	yes	up
0	Op05g01166	1.975319666	4.02E-122	7.36E-118	47.5	19.6	yes	up
0	Op03g01003	1.834437895	2.72E-94	4.97E-90	37.2	14.1	yes	up
0	Op05g01187	1.685802999	2.77E-76	5.08E-72	30.6	11.2	yes	up
0	Op05g01188	1.667756661	9.27E-79	1.70E-74	30.3	10.6	yes	up
0	Op11g00679	1.626672169	1.69E-196	3.08E-192	99.5	98.3	yes	up
0	Op05g01172	1.550876236	6.07E-51	1.11E-46	24.7	9.8	yes	up
0	Op06g01400	1.533988994	5.68E-27	1.04E-22	46.2	39.1	yes	up
0	Op-scaf17g0000	1.491177416	1.69E-67	3.09E-63	21.1	6	yes	up
0	Op-scaf06g0000	1.421369566	4.16E-14	7.61E-10	31.5	25.2	yes	up
0	Op-scaf26g0000	1.417948323	3.11E-18	5.70E-14	33.1	25	yes	up
0	Op-scaf07g0000	1.304104701	6.28E-12	1.15E-07	34.9	30.7	yes	up
0	Op05g01161	1.284556631	2.60E-35	4.76E-31	20	8.4	yes	up
0	Op05g01173	1.276398466	3.19E-38	5.83E-34	19.4	7.5	yes	up
0	Op-scaf16g0000	1.17500536	2.36E-10	4.33E-06	27.1	22	yes	up
0	Op05g01178	1.128413173	4.44E-34	8.13E-30	12.7	3.9	yes	up
0	Op05g01202	1.105377351	1.47E-37	2.69E-33	13.7	4.1	yes	up
0	Op05g01225	1.073276733	1.52E-34	2.79E-30	14.4	4.8	yes	up
0	Op06g00865	1.000439208	3.27E-07	0.005981563	16.8	12.2	yes	up
1	Op11g00940	2.837385458	0	0	54.7	8.1	yes	up
1	Op11g00194	2.791387333	2.05E-305	3.76E-301	65.7	17.2	yes	up
1	Op02g00700	2.779929141	0	0	61.8	13.5	yes	up
1	Op03g01197	2.677449452	2.65E-229	4.85E-225	42.2	7.9	yes	up
1	Op05g00843	2.648473724	0	0	60.9	12.7	yes	up
1	Op09g01326	2.603894938	1.00E-305	1.84E-301	68	18.7	yes	up
1	Op02g01215	2.507436513	4.71E-278	8.61E-274	41.9	6.1	yes	up
1	Op03g02217	2.46906889	7.98E-273	1.46E-268	78.8	30.5	yes	up
1	Op03g00156	2.392905981	2.41E-131	4.41E-127	48.7	18.8	yes	up
1	Op11g00461	2.264439781	9.62E-213	1.76E-208	36.6	6.3	yes	up
1	Op03g01758	2.263183101	5.50E-191	1.01E-186	76.4	43.3	yes	up
1	Op05g00764	2.256015351	9.23E-216	1.69E-211	74	36.4	yes	up
1	Op08g01000	2.229575069	8.71E-190	1.59E-185	54.4	16.5	yes	up
1	Op05g01464	2.204019087	2.72E-186	4.97E-182	70.2	32.7	yes	up
1	Op11g01294	2.135975328	8.74E-136	1.60E-131	45.1	14.3	yes	up
1	Op03g02310	2.040239071	1.67E-255	3.06E-251	45.6	8	yes	up
1	Op08g01715	2.004400288	1.17E-101	2.14E-97	45.4	19.8	yes	up

1	Op11g00475	2.003736358	2.10E-174	3.84E-170	53.2	17.7	yes	up
1	Op04g00216	1.939219869	1.03E-112	1.88E-108	55.2	26.7	yes	up
1	Op03g00169	1.909595462	5.50E-117	1.01E-112	57.3	30.7	yes	up
1	Op09g01008	1.902498704	1.79E-184	3.27E-180	57.8	18.3	yes	up
1	Op06g01482	1.868948697	1.98E-209	3.63E-205	23.9	2.1	yes	up
1	Op03g00092	1.868768004	3.03E-182	5.54E-178	76.7	38.8	yes	up
1	Op11g00447	1.826926502	1.19E-132	2.18E-128	39.6	11.3	yes	up
1	Op11g00851	1.768749445	1.07E-112	1.95E-108	55.8	29	yes	up
1	Op01g00899	1.76829965	2.00E-161	3.67E-157	76.3	52	yes	up
1	Op03g01424	1.739763066	3.80E-157	6.95E-153	21	2.3	yes	up
1	Op02g01422	1.735033092	1.76E-139	3.22E-135	24.7	4	yes	up
1	Op05g00652	1.683567485	7.03E-95	1.29E-90	47.7	22.3	yes	up
1	Op02g01454	1.675929355	5.36E-19	9.82E-15	15.3	7.4	yes	up
1	Op10g00684	1.664279117	2.43E-71	4.46E-67	16.1	3.2	yes	up
1	Op11g01296	1.63685036	2.53E-125	4.63E-121	13.6	1.1	yes	up
1	Op07g00857	1.636321519	3.02E-115	5.53E-111	35.4	10.1	yes	up
1	Op01g00446	1.610492134	8.20E-56	1.50E-51	33.8	15.8	yes	up
1	Op06g00396	1.592560431	9.33E-49	1.71E-44	40.8	24.8	yes	up
1	Op11g00476	1.587769026	1.66E-78	3.03E-74	28.4	9.1	yes	up
1	Op05g01964	1.576877682	5.99E-111	1.10E-106	24.8	5.2	yes	up
1	Op03g00397	1.55872545	5.36E-132	9.81E-128	19.7	2.6	yes	up
1	Op04g00252	1.555923825	3.58E-102	6.55E-98	50.8	22.6	yes	up
1	Op11g00904	1.545676495	1.54E-90	2.82E-86	20.9	4.3	yes	up
1	Op06g00054	1.540693266	1.17E-101	2.13E-97	17.4	2.7	yes	up
1	Op11g00495	1.536907522	2.23E-59	4.09E-55	28.8	11.2	yes	up
1	Op05g00784	1.524381808	4.28E-87	7.83E-83	26.6	7.2	yes	up
1	Op03g01323	1.52141472	2.55E-77	4.67E-73	58.4	40.1	yes	up
1	Op04g00186	1.51710278	5.78E-87	1.06E-82	15.7	2.5	yes	up
1	Op09g00958	1.51423165	1.65E-94	3.02E-90	20.3	4	yes	up
1	Op11g00854	1.505920931	8.15E-77	1.49E-72	46.9	23.6	yes	up
1	Op09g01534	1.503762599	3.56E-98	6.51E-94	31.8	9.1	yes	up
1	Op01g02215	1.490981038	3.06E-32	5.60E-28	24.3	12.2	yes	up
1	Op02g00368	1.487304836	6.33E-74	1.16E-69	45.2	22.9	yes	up
1	Op01g01702	1.485237618	9.74E-99	1.78E-94	38	12.7	yes	up
1	Op03g00176	1.471412351	8.16E-89	1.49E-84	36.2	12.5	yes	up
1	Op06g01470	1.470907584	4.71E-56	8.62E-52	34.9	16.7	yes	up
1	Op06g01442	1.457266392	3.29E-115	6.02E-111	50.9	19.3	yes	up
1	Op09g01119	1.447508994	4.91E-74	8.99E-70	36.8	14.2	yes	up
1	Op04g00368	1.442020691	5.22E-50	9.55E-46	26.8	10.6	yes	up
1	Op06g00644	1.421309956	1.30E-122	2.39E-118	20.2	2.9	yes	up
1	Op06g01432	1.417661615	1.70E-88	3.12E-84	25.1	6.3	yes	up
1	Op07g01230	1.40610689	3.91E-69	7.15E-65	62.7	47.9	yes	up
1	Op01g00911	1.38886434	1.19E-54	2.18E-50	23.7	8.5	yes	up
1	Op11g01422	1.386578292	5.02E-58	9.18E-54	21.7	6.8	yes	up
1	Op03g02125	1.360689081	8.27E-145	1.51E-140	83	60.9	yes	up
1	Op07g00395	1.35481396	3.05E-32	5.58E-28	11.8	3.5	yes	up
1	Op08g00998	1.338020227	4.27E-86	7.82E-82	49.3	22.2	yes	up
1	Op10g00728	1.330144043	2.07E-81	3.80E-77	36.9	13.6	yes	up
1	Op10g00675	1.329242532	2.34E-143	4.28E-139	65.6	26.8	yes	up
1	Op09g01029	1.329215757	1.18E-113	2.17E-109	18.5	2.6	yes	up
1	Op06g00944	1.327897575	1.18E-37	2.16E-33	20.4	8	yes	up
1	Op04g01040	1.323036316	9.53E-44	1.74E-39	34.3	18.4	yes	up
1	Op08g01732	1.322418906	2.50E-34	4.57E-30	38	24.7	yes	up
1	Op05g00953	1.321603836	3.83E-53	7.01E-49	52.4	37.5	yes	up
1	Op07g01425	1.314287327	4.93E-42	9.03E-38	33.4	17.7	yes	up
1	Op07g00512	1.312013452	7.71E-97	1.41E-92	63	36.1	yes	up
1	Op05g00156	1.30192353	5.59E-60	1.02E-55	60	39.7	yes	up
1	Op05g01775	1.298423214	3.32E-91	6.08E-87	17.5	3	yes	up
1	Op01g02073	1.295896548	2.75E-47	5.03E-43	61.4	50.1	yes	up
1	Op05g00896	1.293256806	1.34E-44	2.46E-40	33.9	17.6	yes	up
1	Op01g02357	1.292535994	5.57E-41	1.02E-36	33.1	18	yes	up
1	Op03g01163	1.290949096	4.31E-66	7.89E-62	34.9	13.9	yes	up

1	Op09g00404	1.286398425	1.14E-54	2.08E-50	30.2	12.5	yes	up
1	Op06g00670	1.28628161	1.06E-81	1.94E-77	16.6	3	yes	up
1	Op03g01350	1.281426271	6.18E-68	1.13E-63	61.7	43.7	yes	up
1	Op09g01210	1.281086961	4.04E-60	7.40E-56	22.7	7.2	yes	up
1	Op07g00177	1.279316895	1.90E-45	3.48E-41	24	9.5	yes	up
1	Op03g02131	1.270738705	9.61E-33	1.76E-28	39	26.5	yes	up
1	Op09g00812	1.268905136	2.41E-44	4.41E-40	32.8	16.6	yes	up
1	Op01g01063	1.25444478	4.56E-45	8.34E-41	52.6	39.7	yes	up
1	Op09g00096	1.241630855	2.09E-50	3.82E-46	17.1	4.9	yes	up
1	Op02g01687	1.240714476	1.83E-40	3.35E-36	24.4	10.6	yes	up
1	Op05g00924	1.233407472	2.87E-95	5.26E-91	70.4	47.6	yes	up
1	Op04g01394	1.224748667	7.05E-27	1.29E-22	26.7	15.4	yes	up
1	Op03g01757	1.223859598	3.10E-31	5.68E-27	30.4	17.6	yes	up
1	Op06g00520	1.221677596	8.17E-99	1.50E-94	77.5	55.7	yes	up
1	Op11g00145	1.211746524	1.16E-30	2.13E-26	25.5	13.3	yes	up
1	Op02g01529	1.177529513	1.04E-31	1.91E-27	30.4	17	yes	up
1	Op10g01591	1.171008923	8.63E-39	1.58E-34	22.8	9.6	yes	up
1	Op11g00243	1.168978312	7.75E-32	1.42E-27	36.6	23.6	yes	up
1	Op11g00496	1.168155952	6.22E-45	1.14E-40	32.1	15	yes	up
1	Op04g01504	1.16741031	1.10E-30	2.01E-26	44	33.5	yes	up
1	Op11g00144	1.158693448	1.06E-48	1.95E-44	17.5	5.3	yes	up
1	Op05g01399	1.152821377	3.16E-18	5.79E-14	17.7	9.5	yes	up
1	Op01g01751	1.143662004	3.19E-66	5.83E-62	69.7	58.4	yes	up
1	Op01g00162	1.14192316	1.26E-27	2.32E-23	24.8	13.2	yes	up
1	Op02g00260	1.128021866	5.87E-28	1.07E-23	20.5	9.7	yes	up
1	Op07g00653	1.126503629	6.26E-50	1.15E-45	17.8	5.2	yes	up
1	Op05g00197	1.12650248	3.06E-43	5.61E-39	51.5	38.8	yes	up
1	Op07g00679	1.124485359	1.52E-25	2.77E-21	32.6	20.6	yes	up
1	Op10g01545	1.102432083	3.03E-38	5.54E-34	26.6	12.4	yes	up
1	Op07g00789	1.096987497	5.26E-50	9.62E-46	16.7	4.7	yes	up
1	Op01g00075	1.090216793	2.03E-26	3.72E-22	30.7	18.3	yes	up
1	Op02g00139	1.089880321	2.62E-31	4.79E-27	20.3	9	yes	up
1	Op01g02039	1.08563134	1.37E-25	2.51E-21	24.2	13.2	yes	up
1	Op03g01677	1.08369693	1.05E-24	1.93E-20	40.8	31.9	yes	up
1	Op05g00170	1.083001236	4.30E-30	7.86E-26	14.1	5	yes	up
1	Op09g00407	1.080737528	2.91E-51	5.33E-47	15.6	4.1	yes	up
1	Op05g00484	1.078267425	8.44E-31	1.54E-26	16.5	6.4	yes	up
1	Op-scaf01g0007	1.074864951	3.14E-48	5.76E-44	61.5	52.7	yes	up
1	Op09g00077	1.067895023	8.02E-44	1.47E-39	11.2	2.5	yes	up
1	Op09g00519	1.064058133	1.62E-96	2.97E-92	82.9	68.3	yes	up
1	Op08g00107	1.057214412	1.78E-10	3.25E-06	14.6	8.9	yes	up
1	Op09g01251	1.05166154	2.13E-17	3.89E-13	33.6	25.7	yes	up
1	Op03g00470	1.050373629	2.60E-34	4.75E-30	51.8	42.3	yes	up
1	Op06g00404	1.047001874	1.17E-16	2.14E-12	26.1	17.8	yes	up
1	Op07g01737	1.044771745	2.85E-18	5.22E-14	20	11.2	yes	up
1	Op05g01466	1.042878024	2.65E-57	4.85E-53	12.6	2.5	yes	up
1	Op03g00702	1.042794178	7.35E-27	1.35E-22	18.9	8.6	yes	up
1	Op01g01231	1.03608452	3.82E-26	7.00E-22	15	6.1	yes	up
1	Op01g00658	1.036001708	3.35E-49	6.13E-45	61.8	52.8	yes	up
1	Op04g00235	1.030947515	2.84E-151	5.19E-147	94.2	79.3	yes	up
1	Op02g02300	1.028178307	3.69E-23	6.76E-19	43.3	36.3	yes	up
1	Op05g01757	1.027402448	2.81E-15	5.15E-11	26.3	18.3	yes	up
1	Op02g02187	1.024450397	4.79E-65	8.77E-61	61.8	40.3	yes	up
1	Op10g00383	1.0242774	8.35E-31	1.53E-26	51.8	44.8	yes	up
1	Op01g01228	1.022579613	2.58E-45	4.72E-41	13.2	3.3	yes	up
1	Op03g02265	1.020564349	4.90E-23	8.97E-19	30.8	20.1	yes	up
1	Op09g01536	1.019089669	3.42E-77	6.25E-73	11.6	1.5	yes	up
1	Op05g00656	1.009224776	2.16E-14	3.96E-10	18.9	11.4	yes	up
1	Op06g00587	1.005503236	4.58E-29	8.38E-25	13.8	4.9	yes	up
1	Op05g01934	1.004528679	9.60E-62	1.76E-57	54	30.1	yes	up
1	Op06g00012	1.002933396	1.87E-28	3.42E-24	49.9	42.5	yes	up
1	Op11g01423	1.000831713	3.61E-35	6.62E-31	16.7	5.9	yes	up

2	Op10g00402	2.286867382	5.49E-188	1.00E-183	47	11.7	yes	up
2	Op05g01618	2.198550062	1.33E-273	2.43E-269	67.3	18.3	yes	up
2	Op08g00333	2.002285748	1.27E-300	2.33E-296	96	64.2	yes	up
2	Op07g01754	1.962304216	9.13E-223	1.67E-218	42.8	7.7	yes	up
2	Op03g01476	1.784718832	1.10E-255	2.01E-251	39	5.3	yes	up
2	Op06g00264	1.771129309	4.58E-290	8.38E-286	72.6	19	yes	up
2	Op11g00001	1.736442198	1.08E-142	1.98E-138	34.9	7.8	yes	up
2	Op01g01848	1.553324161	7.11E-103	1.30E-98	41.4	13.8	yes	up
2	Op08g01504	1.5283164	3.47E-220	6.35E-216	53.9	12.5	yes	up
2	Op05g00437	1.475848689	1.28E-229	2.34E-225	52.5	11.5	yes	up
2	Op01g02256	1.464119552	5.03E-262	9.20E-258	97.8	57.1	yes	up
2	Op07g01445	1.353673755	5.12E-180	9.37E-176	74.3	29.5	yes	up
2	Op04g00576	1.346449318	1.17E-44	2.15E-40	12.9	3.1	yes	up
2	Op03g00377	1.321372595	3.20E-111	5.86E-107	75	42.8	yes	up
2	Op03g01658	1.302784805	1.65E-91	3.01E-87	36.5	11.6	yes	up
2	Op01g01866	1.226297904	4.94E-200	9.04E-196	94.4	52.1	yes	up
2	Op08g00261	1.214870867	4.52E-78	8.27E-74	21.9	5.2	yes	up
2	Op06g00453	1.210633408	2.13E-166	3.90E-162	55.6	16.3	yes	up
2	Op02g02185	1.201566999	4.74E-161	8.68E-157	53.9	16	yes	up
2	Op09g01230	1.190056996	4.17E-169	7.64E-165	30.6	4.9	yes	up
2	Op01g00892	1.164262153	1.95E-111	3.57E-107	36	9.8	yes	up
2	Op02g01583	1.158348561	6.33E-119	1.16E-114	20.5	3	yes	up
2	Op05g01044	1.155546983	7.90E-147	1.45E-142	65.3	24.6	yes	up
2	Op05g00182	1.127093417	8.33E-137	1.52E-132	81.7	46.8	yes	up
2	Op07g00616	1.122271526	2.14E-131	3.91E-127	59.9	22.5	yes	up
2	Op03g01708	1.120066809	6.11E-153	1.12E-148	44.5	11.2	yes	up
2	Op04g01530	1.108979684	2.07E-131	3.80E-127	77.7	38.6	yes	up
2	Op06g00282	1.101128099	4.81E-124	8.80E-120	53.8	19.2	yes	up
2	Op06g00265	1.086427153	5.42E-121	9.91E-117	44.1	13.6	yes	up
2	Op03g01212	1.079099844	8.42E-155	1.54E-150	37.3	7.9	yes	up
2	Op01g00602	1.066909121	8.71E-71	1.60E-66	26.5	7.9	yes	up
2	Op02g01239	1.053128645	3.55E-129	6.50E-125	93.4	71.5	yes	up
2	Op03g00239	1.017377073	8.59E-91	1.57E-86	53.3	22.8	yes	up
2	Op02g01228	1.006362348	8.81E-116	1.61E-111	94.8	75.3	yes	up
3	Op01g02324	3.364842851	0	0	70.5	4.3	yes	up
3	Op06g00246	3.142069954	0	0	76.1	11.6	yes	up
3	Op11g00254	2.666881424	0	0	73.4	13.3	yes	up
3	Op03g02101	2.646625421	0	0	89.1	28	yes	up
3	Op04g01662	2.544274636	3.33E-256	6.09E-252	90.6	55.4	yes	up
3	Op03g00570	2.424669565	0	0	65.7	9.4	yes	up
3	Op11g00881	2.422182582	0	0	89.2	22.7	yes	up
3	Op09g00430	2.303865122	0	0	72.5	17	yes	up
3	Op03g00265	2.197182338	0	0	61.2	5.9	yes	up
3	Op05g00229	2.166060189	3.12E-160	5.71E-156	66.7	27	yes	up
3	Op11g00333	2.061040736	4.37E-116	7.99E-112	37.2	10	yes	up
3	Op04g00596	2.049619345	3.15E-284	5.76E-280	78.6	25.4	yes	up
3	Op08g00497	2.043851214	0	0	49	3.6	yes	up
3	Op09g00651	2.039737002	0	0	78.3	20	yes	up
3	Op11g00072	1.966630305	0	0	43	2.6	yes	up
3	Op10g00482	1.912760838	2.90E-210	5.31E-206	96.2	71.8	yes	up
3	Op10g00288	1.90324487	9.59E-210	1.76E-205	84	39.1	yes	up
3	Op10g00800	1.899690392	0	0	47.7	5	yes	up
3	Op11g00334	1.862909968	8.70E-136	1.59E-131	27.5	4.8	yes	up
3	Op03g01946	1.845914762	0	0	38.6	1.1	yes	up
3	Op05g01314	1.792027263	3.66E-219	6.69E-215	53.4	11.7	yes	up
3	Op05g00074	1.752328904	3.62E-144	6.63E-140	82.8	50.8	yes	up
3	Op09g00032	1.747558436	1.20E-283	2.19E-279	99.6	77.6	yes	up
3	Op01g00352	1.683674218	2.44E-166	4.47E-162	62.4	20.7	yes	up
3	Op01g00485	1.682684008	1.11E-237	2.04E-233	86.7	37.9	yes	up
3	Op06g00847	1.655290947	9.97E-175	1.83E-170	76	32.2	yes	up
3	Op11g00146	1.620407754	7.08E-266	1.30E-261	56.8	10.9	yes	up
3	Op04g00590	1.615810189	2.77E-249	5.06E-245	47.2	8.2	yes	up

3	Op06g00783	1.581882102	1.85E-160	3.39E-156	53.3	15.3	yes	up
3	Op10g01113	1.580300281	2.92E-252	5.34E-248	57.9	12.2	yes	up
3	Op05g00891	1.574826601	1.99E-307	3.64E-303	37.2	3.4	yes	up
3	Op11g00297	1.572194783	4.74E-193	8.67E-189	78.3	32	yes	up
3	Op01g00093	1.567331811	5.72E-137	1.05E-132	89.6	54.6	yes	up
3	Op02g01796	1.556474116	0	0	42	4.3	yes	up
3	Op04g01464	1.554806084	0	0	32.5	1.6	yes	up
3	Op09g00100	1.553653539	3.37E-204	6.17E-200	69.7	22.7	yes	up
3	Op09g00270	1.531881438	6.97E-113	1.28E-108	38.9	10.9	yes	up
3	Op07g01041	1.517356707	1.27E-213	2.32E-209	95.6	65.5	yes	up
3	Op01g00219	1.511303135	1.49E-274	2.73E-270	45.4	6.2	yes	up
3	Op02g00896	1.496847252	7.94E-215	1.45E-210	44.7	8.4	yes	up
3	Op06g00376	1.486221052	1.24E-91	2.27E-87	53.5	22.8	yes	up
3	Op01g00358	1.478597546	1.27E-140	2.33E-136	90.7	63.6	yes	up
3	Op01g01307	1.476574612	7.16E-124	1.31E-119	21.6	3.1	yes	up
3	Op06g00877	1.466095159	5.60E-107	1.02E-102	81.5	52.9	yes	up
3	Op07g01294	1.456649377	1.34E-135	2.45E-131	81.9	46.2	yes	up
3	Op05g01823	1.452441182	9.18E-93	1.68E-88	67.5	34.7	yes	up
3	Op09g00658	1.438819611	3.93E-186	7.20E-182	60.7	18.1	yes	up
3	Op09g00771	1.435518945	1.13E-262	2.06E-258	34.2	3.5	yes	up
3	Op10g00439	1.424305375	5.31E-115	9.72E-111	70.6	35.5	yes	up
3	Op04g00632	1.41324626	3.39E-165	6.20E-161	79.8	36.1	yes	up
3	Op02g01508	1.407725391	1.73E-143	3.17E-139	25.6	3.8	yes	up
3	Op02g02321	1.407391374	3.47E-132	6.35E-128	92.6	75	yes	up
3	Op07g01371	1.406314476	4.86E-118	8.90E-114	93	73	yes	up
3	Op09g00209	1.402146563	3.27E-145	5.99E-141	95.4	71.2	yes	up
3	Op10g00801	1.391546529	1.62E-255	2.96E-251	44.8	6.7	yes	up
3	Op07g01906	1.3910505	1.49E-139	2.73E-135	51.5	15.8	yes	up
3	Op07g01671	1.390398183	1.59E-265	2.90E-261	52.3	8.8	yes	up
3	Op07g00353	1.386459174	2.01E-133	3.68E-129	88.7	57.5	yes	up
3	Op06g00836	1.382330845	2.92E-276	5.35E-272	33	2.9	yes	up
3	Op10g00392	1.378872148	4.81E-82	8.80E-78	81.3	61.9	yes	up
3	Op09g01545	1.37792093	3.43E-247	6.29E-243	48.5	8.3	yes	up
3	Op02g02357	1.37272223	5.13E-117	9.39E-113	73.2	36.1	yes	up
3	Op09g00331	1.366888734	5.48E-227	1.00E-222	39.9	6	yes	up
3	Op07g00791	1.361765892	4.55E-125	8.33E-121	64.4	27.2	yes	up
3	Op04g01032	1.355103698	7.25E-271	1.33E-266	32.4	2.9	yes	up
3	Op08g00440	1.355091015	6.42E-172	1.18E-167	53.1	15	yes	up
3	Op08g01281	1.339630621	1.39E-201	2.55E-197	44.2	8.3	yes	up
3	Op04g00442	1.334344103	1.13E-114	2.07E-110	80.2	43.3	yes	up
3	Op07g01910	1.320278669	1.44E-116	2.63E-112	46.5	14.8	yes	up
3	Op10g00783	1.313234358	1.22E-127	2.24E-123	54.1	18.8	yes	up
3	Op05g01917	1.307015342	2.79E-103	5.11E-99	65.3	30.5	yes	up
3	Op10g00220	1.300208868	1.01E-68	1.86E-64	24.5	6.7	yes	up
3	Op07g00888	1.300028836	4.13E-117	7.55E-113	57.2	21.7	yes	up
3	Op03g02053	1.297953649	1.09E-121	2.00E-117	78.9	42.7	yes	up
3	Op07g00513	1.281930854	2.39E-80	4.38E-76	56	26.4	yes	up
3	Op02g01798	1.274501469	2.34E-255	4.29E-251	30.5	2.7	yes	up
3	Op07g00264	1.270719535	3.89E-58	7.13E-54	74	52.4	yes	up
3	Op07g00359	1.249837507	2.90E-114	5.30E-110	84	51.1	yes	up
3	Op08g01454	1.248146053	8.61E-173	1.58E-168	29.8	4.3	yes	up
3	Op07g01545	1.24379561	4.56E-113	8.34E-109	63.7	26.8	yes	up
3	Op11g00884	1.239143193	5.50E-55	1.01E-50	30	11.4	yes	up
3	Op10g01447	1.221428918	2.40E-74	4.39E-70	65.5	36.1	yes	up
3	Op02g00363	1.210916914	4.62E-143	8.45E-139	98.4	83	yes	up
3	Op07g01923	1.208841195	6.85E-147	1.25E-142	45.2	11.5	yes	up
3	Op08g00149	1.208741053	5.47E-229	1.00E-224	28.9	2.8	yes	up
3	Op03g00777	1.204315766	2.14E-112	3.91E-108	59.3	23.5	yes	up
3	Op05g01464	1.202229242	2.47E-151	4.52E-147	78	32.2	yes	up
3	Op03g00100	1.182894248	6.98E-88	1.28E-83	52	21.6	yes	up
3	Op11g00864	1.18104003	0	0	25.1	1	yes	up
3	Op09g01292	1.172187848	6.52E-89	1.19E-84	75	43	yes	up

3	Op05g00593	1.150699347	7.44E-134	1.36E-129	55.2	17.9	yes	up
3	Op10g01478	1.148567341	1.26E-192	2.30E-188	31.9	4.4	yes	up
3	Op10g00879	1.146762682	5.44E-98	9.96E-94	54.5	21.8	yes	up
3	Op09g01507	1.145317212	3.40E-63	6.23E-59	51.9	25.6	yes	up
3	Op05g00786	1.140555814	3.17E-77	5.80E-73	30.5	9.1	yes	up
3	Op03g01872	1.138456467	2.32E-69	4.24E-65	44	18.7	yes	up
3	Op03g00616	1.138373651	4.51E-69	8.25E-65	54.6	26	yes	up
3	Op10g01287	1.133422071	1.61E-125	2.94E-121	40.7	11	yes	up
3	Op01g00291	1.131243842	5.88E-56	1.08E-51	17.1	4.2	yes	up
3	Op05g00185	1.129755545	7.65E-80	1.40E-75	48.7	19.9	yes	up
3	Op01g02433	1.128431981	2.21E-78	4.05E-74	36.9	12.6	yes	up
3	Op07g00067	1.12425616	2.61E-172	4.77E-168	23.9	2.6	yes	up
3	Op06g01138	1.121668095	9.73E-100	1.78E-95	42	13.6	yes	up
3	Op07g01195	1.12153775	1.51E-80	2.77E-76	40.8	14.7	yes	up
3	Op11g00206	1.116485752	3.29E-102	6.02E-98	84.9	61.1	yes	up
3	Op01g01718	1.114896771	1.70E-93	3.11E-89	25.6	5.7	yes	up
3	Op10g00659	1.113528121	4.31E-91	7.90E-87	59.8	28.2	yes	up
3	Op08g01716	1.111295935	6.46E-90	1.18E-85	80	48.5	yes	up
3	Op11g00416	1.106225819	6.74E-76	1.23E-71	41.7	15.6	yes	up
3	Op11g01192	1.104672637	1.67E-128	3.06E-124	95	72.5	yes	up
3	Op08g01334	1.100889897	9.29E-56	1.70E-51	50.9	26.6	yes	up
3	Op07g01948	1.097457662	3.08E-93	5.64E-89	74	40.3	yes	up
3	Op01g01727	1.097420431	8.28E-97	1.52E-92	75.1	39.7	yes	up
3	Op04g00423	1.094960213	3.19E-130	5.85E-126	24.5	3.9	yes	up
3	Op03g00135	1.089223289	1.57E-126	2.88E-122	39.4	10	yes	up
3	Op07g01874	1.087609025	3.65E-59	6.69E-55	26.6	8.6	yes	up
3	Op09g01372	1.087096572	2.92E-111	5.34E-107	83.2	51.5	yes	up
3	Op01g00328	1.081329122	1.17E-84	2.14E-80	55.8	23.9	yes	up
3	Op02g02072	1.078662628	5.46E-142	9.99E-138	37.4	8.2	yes	up
3	Op01g01903	1.063713039	2.17E-116	3.98E-112	39.1	10.9	yes	up
3	Op10g01109	1.060187931	9.00E-122	1.65E-117	41	11.1	yes	up
3	Op01g02281	1.050602534	3.82E-89	7.00E-85	26.9	6.5	yes	up
3	Op06g00698	1.0492409	6.52E-97	1.19E-92	41.2	13.2	yes	up
3	Op05g01337	1.047630954	1.27E-85	2.32E-81	30.4	8.3	yes	up
3	Op07g01186	1.034918199	7.26E-97	1.33E-92	92.7	75.1	yes	up
3	Op10g00796	1.023418765	1.64E-106	3.00E-102	30.5	7.1	yes	up
3	Op08g01205	1.023410689	6.50E-91	1.19E-86	68.5	33.8	yes	up
3	Op09g01309	1.0204644	8.99E-79	1.65E-74	28.6	8.1	yes	up
3	Op05g01400	1.019021059	2.15E-119	3.93E-115	36.9	9.4	yes	up
3	Op01g01213	1.009453676	9.10E-111	1.67E-106	83.9	48.8	yes	up
3	Op01g02037	1.008055092	2.29E-70	4.19E-66	50	22.2	yes	up
4	Op01g01729	3.645101227	0	0	83.8	19.2	yes	up
4	Op10g01176	3.260201318	0	0	83.8	22	yes	up
4	Op10g01352	3.119543579	0	0	43.2	2.2	yes	up
4	Op09g01008	2.994282907	0	0	86	16.9	yes	up
4	Op06g01442	2.983600135	0	0	88.2	17	yes	up
4	Op06g00314	2.856781347	0	0	85.8	18.7	yes	up
4	Op09g01119	2.823958148	0	0	70.9	11.9	yes	up
4	Op01g01728	2.812264199	0	0	76.8	10.3	yes	up
4	Op11g00496	2.786702689	0	0	78.2	11.7	yes	up
4	Op08g00998	2.763061978	0	0	87.4	19.7	yes	up
4	Op10g00675	2.714566972	0	0	93.3	25.4	yes	up
4	Op07g01360	2.69814864	1.92E-248	3.51E-244	76.1	26.1	yes	up
4	Op01g00054	2.635983755	0	0	59.9	7.1	yes	up
4	Op07g00629	2.622140854	6.28E-271	1.15E-266	46.1	6.5	yes	up
4	Op02g01804	2.616800762	0	0	87.4	25.4	yes	up
4	Op03g00092	2.573468914	3.20E-303	5.86E-299	93.1	38.4	yes	up
4	Op07g01326	2.558052784	0	0	99.7	74.2	yes	up
4	Op05g01934	2.53921283	0	0	92.4	27.5	yes	up
4	Op02g00290	2.514773585	0	0	62	6.7	yes	up
4	Op06g01438	2.511843355	1.21E-172	2.22E-168	47.4	11.2	yes	up
4	Op10g00728	2.458045814	0	0	70.1	11.5	yes	up

4	Op01g00423	2.42994547	5.42E-188	9.92E-184	40.9	7.6	yes	up
4	Op03g02059	2.323539388	0	0	95.3	50.9	yes	up
4	Op07g01208	2.310062328	3.31E-288	6.05E-284	65.4	13.5	yes	up
4	Op09g00105	2.292663143	3.76E-300	6.88E-296	58.2	10.1	yes	up
4	Op07g01569	2.288761031	3.54E-84	6.48E-80	52.8	25.9	yes	up
4	Op06g01435	2.278937929	0	0	59.7	7.4	yes	up
4	Op08g01000	2.237316809	3.88E-279	7.10E-275	69.8	16.1	yes	up
4	Op01g00075	2.226591154	0	0	71.7	15.3	yes	up
4	Op10g00760	2.211851516	8.11E-222	1.48E-217	54	11.4	yes	up
4	Op08g01277	2.198315001	1.09E-191	2.00E-187	61	18.8	yes	up
4	Op11g00898	2.1853746	3.56E-268	6.52E-264	99.4	92.1	yes	up
4	Op06g00087	2.170757413	0	0	61.5	9.7	yes	up
4	Op08g01173	2.165205014	3.51E-265	6.43E-261	76.5	23.2	yes	up
4	Op04g01534	2.14643777	1.61E-234	2.94E-230	60.6	14.8	yes	up
4	Op09g00429	2.111719586	8.08E-301	1.48E-296	97.4	60.4	yes	up
4	Op11g00495	2.092421784	2.71E-276	4.97E-272	55.3	9.4	yes	up
4	Op03g01163	2.091073354	0	0	66.1	11.8	yes	up
4	Op11g01294	2.056997094	7.33E-128	1.34E-123	48.3	14.7	yes	up
4	Op10g00526	2.048087703	1.71E-275	3.14E-271	45.4	6.1	yes	up
4	Op03g02226	2.039983767	4.59E-206	8.40E-202	33.6	4.2	yes	up
4	Op09g00972	2.034095567	5.37E-304	9.84E-300	50.9	7	yes	up
4	Op07g00512	2.031869652	3.21E-262	5.87E-258	87.6	34.7	yes	up
4	Op09g00296	2.024057624	1.09E-210	2.00E-206	57.4	14.6	yes	up
4	Op02g00534	2.005774983	4.23E-226	7.75E-222	51.8	10	yes	up
4	Op11g00438	2.00477564	0	0	46.6	4.2	yes	up
4	Op07g00537	2.000017125	2.22E-172	4.06E-168	36.5	6.2	yes	up
4	Op02g00653	1.994277319	3.83E-275	7.01E-271	38	3.9	yes	up
4	Op11g01254	1.929575059	3.06E-224	5.60E-220	46.6	8	yes	up
4	Op02g00606	1.928107173	0	0	41.9	2.5	yes	up
4	Op03g00654	1.9225539	4.30E-254	7.87E-250	52.4	9	yes	up
4	Op05g00334	1.90195703	2.07E-220	3.79E-216	54.3	11.8	yes	up
4	Op10g00656	1.878057714	7.57E-245	1.39E-240	69.2	17.8	yes	up
4	Op03g02217	1.870817178	3.43E-192	6.27E-188	80	31.4	yes	up
4	Op03g01051	1.854693841	1.03E-288	1.88E-284	37.1	3.4	yes	up
4	Op11g00931	1.847743774	1.45E-214	2.65E-210	73.6	23.8	yes	up
4	Op01g01545	1.845470513	1.59E-144	2.91E-140	51.1	15.5	yes	up
4	Op02g01461	1.821562325	8.89E-220	1.63E-215	41.5	6.2	yes	up
4	Op06g00154	1.800166655	3.22E-63	5.90E-59	22.8	6	yes	up
4	Op07g00448	1.750137646	2.79E-235	5.11E-231	40.6	5.5	yes	up
4	Op11g00544	1.731907171	1.38E-172	2.52E-168	73.7	30.4	yes	up
4	Op10g00503	1.730354168	8.16E-177	1.49E-172	52.8	13.6	yes	up
4	Op10g00229	1.725547032	1.85E-187	3.38E-183	39.1	6.6	yes	up
4	Op11g00242	1.722681414	2.91E-220	5.32E-216	63.2	15.9	yes	up
4	Op04g00368	1.715168478	4.72E-178	8.64E-174	45.8	9.4	yes	up
4	Op07g00682	1.703529622	1.57E-131	2.88E-127	49.2	15	yes	up
4	Op09g00519	1.698328693	3.88E-198	7.09E-194	96.5	67.5	yes	up
4	Op01g01392	1.693979454	2.24E-147	4.11E-143	48.9	12.8	yes	up
4	Op05g01949	1.682123636	3.71E-295	6.80E-291	33.6	2.5	yes	up
4	Op06g00231	1.670935071	5.68E-138	1.04E-133	61.2	23.3	yes	up
4	Op09g01534	1.669265101	2.23E-205	4.08E-201	46.7	8.4	yes	up
4	Op07g01278	1.645808163	4.72E-135	8.64E-131	48.2	14.3	yes	up
4	Op01g01726	1.621353316	1.34E-165	2.44E-161	41.8	8.6	yes	up
4	Op03g00156	1.602692333	5.39E-99	9.87E-95	51.1	19.3	yes	up
4	Op11g01301	1.597311292	4.02E-203	7.36E-199	25	2.1	yes	up
4	Op04g00252	1.568741699	1.94E-133	3.54E-129	61.5	22.3	yes	up
4	Op11g01423	1.561820085	5.09E-186	9.32E-182	34	4.8	yes	up
4	Op03g01350	1.556848834	9.54E-160	1.75E-155	81.2	42.5	yes	up
4	Op04g00235	1.541407029	2.39E-231	4.38E-227	99.4	79.2	yes	up
4	Op07g00857	1.538113155	8.23E-156	1.51E-151	44.8	9.9	yes	up
4	Op01g00162	1.524311825	7.70E-144	1.41E-139	45.4	11.8	yes	up
4	Op02g00740	1.515763787	6.34E-143	1.16E-138	69.6	29.8	yes	up
4	Op01g01702	1.51476758	1.38E-186	2.54E-182	53.3	12	yes	up

4	Op07g00517	1.509716867	8.82E-190	1.61E-185	37.1	5.8	yes	up
4	Op03g00063	1.505467019	1.17E-190	2.14E-186	41.6	7.1	yes	up
4	Op01g00163	1.494871002	4.14E-175	7.57E-171	31.8	4.4	yes	up
4	Op06g00216	1.494230501	2.89E-148	5.30E-144	36.9	7.3	yes	up
4	Op01g01063	1.48582418	9.75E-137	1.78E-132	75.5	38.2	yes	up
4	Op02g00368	1.482503894	1.80E-134	3.29E-130	60.6	22.1	yes	up
4	Op07g01723	1.467999296	7.40E-133	1.35E-128	24.4	3.5	yes	up
4	Op11g00194	1.458249866	1.57E-46	2.87E-42	41.3	20.2	yes	up
4	Op05g00313	1.44515364	3.55E-130	6.50E-126	35.3	7.5	yes	up
4	Op08g01617	1.430327175	5.02E-144	9.19E-140	39	8.2	yes	up
4	Op01g01723	1.418089366	1.63E-160	2.98E-156	27.6	3.6	yes	up
4	Op10g00691	1.408153533	4.81E-142	8.81E-138	32.1	5.6	yes	up
4	Op09g01326	1.383669618	1.29E-109	2.36E-105	56.5	20.7	yes	up
4	Op03g00232	1.373562613	1.50E-111	2.74E-107	65.8	32	yes	up
4	Op07g00653	1.370098815	3.88E-172	7.11E-168	31.7	4.4	yes	up
4	Op03g00176	1.367523221	9.21E-135	1.69E-130	47.2	12.2	yes	up
4	Op11g00447	1.362010625	1.72E-141	3.14E-137	46.3	11.4	yes	up
4	Op11g00475	1.349992517	7.99E-127	1.46E-122	55.5	18.3	yes	up
4	Op07g01994	1.348118255	1.59E-229	2.91E-225	23.8	1.5	yes	up
4	Op03g01758	1.34159194	2.18E-84	3.99E-80	72.8	44.3	yes	up
4	Op01g00609	1.338856508	1.62E-129	2.96E-125	30.4	5.6	yes	up
4	Op07g00789	1.331769972	5.82E-175	1.07E-170	30.2	3.9	yes	up
4	Op01g00625	1.331211147	9.94E-196	1.82E-191	25.3	2.2	yes	up
4	Op03g00742	1.327877311	3.68E-101	6.74E-97	25.3	4.8	yes	up
4	Op04g01502	1.326670802	6.99E-105	1.28E-100	84.2	57.5	yes	up
4	Op08g00209	1.316705425	1.26E-187	2.31E-183	23.5	2	yes	up
4	Op09g01569	1.312895125	6.77E-84	1.24E-79	29.2	7.8	yes	up
4	Op10g00576	1.311457336	1.67E-137	3.06E-133	27.6	4.2	yes	up
4	Op01g00204	1.308596803	5.73E-105	1.05E-100	29.1	6.3	yes	up
4	Op01g02338	1.307501306	1.54E-100	2.81E-96	41.3	12.6	yes	up
4	Op07g00477	1.299536651	1.21E-147	2.22E-143	33.7	6	yes	up
4	Op05g00156	1.295873121	9.02E-132	1.65E-127	79	38.6	yes	up
4	Op09g00994	1.291283784	1.12E-166	2.04E-162	29.9	4.1	yes	up
4	Op07g01575	1.288182044	1.63E-115	2.98E-111	23.4	3.6	yes	up
4	Op09g00878	1.287085693	3.96E-113	7.25E-109	30.1	6	yes	up
4	Op07g01425	1.287053344	8.65E-72	1.58E-67	42.8	17.3	yes	up
4	Op03g01421	1.285423584	2.48E-90	4.54E-86	28.8	6.7	yes	up
4	Op01g02146	1.2808711	1.18E-136	2.15E-132	30.9	5.5	yes	up
4	Op11g00285	1.279084689	2.17E-116	3.97E-112	39.6	10.2	yes	up
4	Op02g02255	1.278976279	8.01E-98	1.47E-93	38.4	11.3	yes	up
4	Op03g01496	1.277122385	6.50E-170	1.19E-165	26	2.9	yes	up
4	Op03g02014	1.277027618	2.66E-86	4.87E-82	52.6	23.3	yes	up
4	Op10g00724	1.276850534	2.32E-127	4.25E-123	20.1	2.3	yes	up
4	Op03g00308	1.262224407	1.63E-100	2.98E-96	29.2	6.6	yes	up
4	Op03g02310	1.254337084	1.02E-79	1.86E-75	33.9	9.8	yes	up
4	Op02g01214	1.253780593	9.10E-115	1.67E-110	21.9	3.2	yes	up
4	Op02g01491	1.251256816	6.28E-150	1.15E-145	29.5	4.4	yes	up
4	Op05g01847	1.244296538	5.36E-109	9.81E-105	22.6	3.6	yes	up
4	Op03g01232	1.233151925	2.59E-97	4.75E-93	43.5	14.1	yes	up
4	Op10g01545	1.232708638	5.69E-73	1.04E-68	35.8	12	yes	up
4	Op11g00930	1.231336588	1.54E-79	2.81E-75	43.5	16.5	yes	up
4	Op02g02109	1.228827787	7.18E-76	1.31E-71	58.2	31.1	yes	up
4	Op07g00431	1.228416582	2.45E-71	4.49E-67	42.5	17.4	yes	up
4	Op03g00864	1.227294023	1.20E-137	2.20E-133	30.5	5.1	yes	up
4	Op08g00107	1.224674331	5.49E-73	1.00E-68	28	7.9	yes	up
4	Op09g01210	1.224037863	8.12E-124	1.49E-119	33	6.7	yes	up
4	Op08g01657	1.219169938	5.25E-109	9.61E-105	29.5	6	yes	up
4	Op07g01536	1.217303864	4.13E-85	7.56E-81	44.2	16.5	yes	up
4	Op11g00854	1.216640868	1.42E-84	2.60E-80	55.3	23.5	yes	up
4	Op01g02562	1.203458769	8.53E-82	1.56E-77	38.4	12.9	yes	up
4	Op04g00186	1.202770052	5.84E-101	1.07E-96	18.7	2.6	yes	up
4	Op06g00229	1.195958548	7.94E-98	1.45E-93	22.2	3.9	yes	up

4	Op07g01059	1.195896415	4.28E-91	7.84E-87	62	30.8	yes	up
4	Op06g00442	1.194218919	1.39E-110	2.54E-106	20	2.8	yes	up
4	Op01g00326	1.189497401	1.57E-141	2.88E-137	32.7	5.8	yes	up
4	Op02g00700	1.187781306	3.17E-84	5.80E-80	45.5	15.9	yes	up
4	Op05g01464	1.185199441	4.27E-115	7.82E-111	72.4	33.3	yes	up
4	Op02g01729	1.18312836	1.05E-152	1.91E-148	24.2	2.8	yes	up
4	Op07g01737	1.169251676	9.30E-77	1.70E-72	33.4	10.3	yes	up
4	Op06g00227	1.168662316	6.55E-70	1.20E-65	38.1	14.3	yes	up
4	Op11g00243	1.164053755	1.78E-63	3.26E-59	48.2	22.9	yes	up
4	Op10g01172	1.161375487	2.27E-138	4.15E-134	24.7	3.3	yes	up
4	Op08g01645	1.158350458	4.14E-69	7.59E-65	58.4	32.6	yes	up
4	Op11g00439	1.15572758	2.46E-100	4.50E-96	33.3	8.2	yes	up
4	Op06g00176	1.148863471	2.82E-59	5.17E-55	18.4	4.2	yes	up
4	Op05g01328	1.135462337	3.18E-109	5.82E-105	20.6	3	yes	up
4	Op08g01437	1.131690042	1.03E-73	1.89E-69	48	20.8	yes	up
4	Op03g00677	1.131063169	4.19E-71	7.67E-67	31.5	9.8	yes	up
4	Op01g00520	1.128447851	3.65E-67	6.68E-63	41.5	17.1	yes	up
4	Op11g01256	1.125718206	3.91E-101	7.15E-97	24.7	4.6	yes	up
4	Op03g02334	1.125028438	9.74E-70	1.78E-65	31.2	9.9	yes	up
4	Op11g00141	1.123260933	1.04E-159	1.91E-155	96.8	78	yes	up
4	Op03g02306	1.122402559	3.45E-172	6.31E-168	20.6	1.6	yes	up
4	Op01g00652	1.120470184	3.54E-54	6.49E-50	36.6	15.3	yes	up
4	Op09g00412	1.114144745	1.45E-57	2.65E-53	34.9	13.6	yes	up
4	Op03g01274	1.110479204	2.02E-107	3.69E-103	23.8	4	yes	up
4	Op02g02015	1.107687781	8.81E-54	1.61E-49	34.9	14.3	yes	up
4	Op01g02073	1.107392733	9.76E-115	1.79E-110	82	48.7	yes	up
4	Op08g00012	1.106585303	1.59E-104	2.91E-100	32	7.2	yes	up
4	Op03g01778	1.095067034	9.03E-95	1.65E-90	27.2	6	yes	up
4	Op08g00079	1.093581081	6.18E-69	1.13E-64	35.3	11.7	yes	up
4	Op02g02152	1.086626718	2.60E-69	4.77E-65	25.7	6.9	yes	up
4	Op07g01011	1.082722266	1.70E-116	3.11E-112	19.7	2.5	yes	up
4	Op05g01698	1.08079111	2.61E-92	4.78E-88	17.2	2.4	yes	up
4	Op09g01228	1.077356941	8.96E-118	1.64E-113	19.4	2.4	yes	up
4	Op02g01741	1.075439252	3.20E-84	5.86E-80	28.3	7	yes	up
4	Op01g00388	1.071065873	1.95E-124	3.56E-120	18.8	2.1	yes	up
4	Op07g01950	1.06352174	7.35E-75	1.35E-70	44.8	17.4	yes	up
4	Op09g00428	1.055278303	4.83E-89	8.83E-85	13.4	1.4	yes	up
4	Op04g00121	1.052523295	7.71E-69	1.41E-64	33.9	11.3	yes	up
4	Op09g01352	1.03772228	2.48E-73	4.55E-69	80.9	61.1	yes	up
4	Op03g00054	1.036634328	7.49E-82	1.37E-77	26.6	6.4	yes	up
4	Op06g01391	1.03316129	3.91E-58	7.16E-54	43.2	19.9	yes	up
4	Op08g01304	1.026836615	5.15E-44	9.42E-40	38.2	18.8	yes	up
4	Op05g00484	1.020161788	6.70E-70	1.23E-65	24.1	6	yes	up
4	Op06g00292	1.014884904	1.07E-98	1.96E-94	21.5	3.5	yes	up
4	Op05g00359	1.011261456	1.73E-44	3.18E-40	16.9	4.5	yes	up
4	Op04g01300	1.010998117	8.52E-88	1.56E-83	24.5	5.1	yes	up
4	Op05g00652	1.004330664	1.07E-55	1.96E-51	47.3	22.8	yes	up
5	Op11g00504	2.247078247	1.58E-93	2.89E-89	44.5	16.7	yes	up
5	Op08g01484	2.226313251	2.21E-96	4.04E-92	52.3	22.2	yes	up
5	Op01g01318	2.172884986	1.98E-88	3.62E-84	51.7	23.3	yes	up
5	Op08g01596	2.146510261	2.46E-120	4.51E-116	55.5	22.5	yes	up
5	Op01g00766	1.855081178	5.07E-75	9.27E-71	52.8	27.9	yes	up
5	Op08g00925	1.834801549	6.47E-55	1.18E-50	39.7	19.2	yes	up
5	Op05g00326	1.826752309	1.79E-101	3.28E-97	69.7	40.2	yes	up
5	Op09g01525	1.802975675	6.78E-95	1.24E-90	57.2	27.6	yes	up
5	Op05g00583	1.755888604	9.01E-39	1.65E-34	27.8	12.1	yes	up
5	Op02g00798	1.754361029	4.41E-55	8.08E-51	35.5	15.5	yes	up
5	Op08g00792	1.741034233	7.42E-38	1.36E-33	34.5	17.9	yes	up
5	Op09g00915	1.723550843	1.85E-45	3.39E-41	45.7	28.4	yes	up
5	Op09g00448	1.682818681	1.12E-126	2.05E-122	76.9	44.2	yes	up
5	Op02g02244	1.679958408	8.37E-67	1.53E-62	56.9	34.7	yes	up
5	Op05g00786	1.664230038	5.13E-28	9.40E-24	22.8	10.1	yes	up

5	Op06g01084	1.633687719	2.00E-37	3.67E-33	24.3	9.8	yes	up
5	Op11g00577	1.62952487	1.91E-30	3.49E-26	23.7	10.6	yes	up
5	Op06g00383	1.624171363	6.35E-36	1.16E-31	25.4	10.7	yes	up
5	Op03g00212	1.623930565	1.08E-40	1.98E-36	54.6	41.2	yes	up
5	Op03g02151	1.595923562	2.30E-13	4.22E-09	25.7	17.5	yes	up
5	Op03g00062	1.594803435	2.82E-35	5.16E-31	42.5	27.7	yes	up
5	Op01g02359	1.588214833	2.22E-41	4.07E-37	37.1	19.7	yes	up
5	Op02g01657	1.585566844	7.08E-39	1.30E-34	40.3	23.8	yes	up
5	Op01g00068	1.573935676	1.15E-39	2.11E-35	37.8	20.5	yes	up
5	Op01g00545	1.568514857	5.48E-26	1.00E-21	21.2	9.4	yes	up
5	Op09g01568	1.566052325	3.18E-42	5.81E-38	24.5	9.2	yes	up
5	Op05g00642	1.53753404	1.25E-52	2.29E-48	54.8	33.6	yes	up
5	Op03g00132	1.529406472	4.03E-27	7.38E-23	30.9	16.9	yes	up
5	Op02g02330	1.526437955	4.49E-38	8.21E-34	29.5	13.6	yes	up
5	Op02g01936	1.517082469	2.04E-38	3.74E-34	37.5	21	yes	up
5	Op05g01954	1.516301703	6.26E-40	1.15E-35	41.1	24.3	yes	up
5	Op05g00089	1.500927936	1.90E-29	3.47E-25	19.5	7.8	yes	up
5	Op07g00093	1.498486424	1.58E-35	2.89E-31	32.6	16.9	yes	up
5	Op02g02242	1.496759055	2.82E-29	5.16E-25	34	20.2	yes	up
5	Op01g00677	1.490069666	1.03E-75	1.89E-71	70.9	49.5	yes	up
5	Op02g01507	1.465763938	1.81E-30	3.30E-26	16.9	6	yes	up
5	Op11g01390	1.424819298	1.35E-20	2.46E-16	23.2	12.5	yes	up
5	Op07g01253	1.393040065	1.40E-15	2.57E-11	20.3	11.2	yes	up
5	Op05g01181	1.385537561	8.29E-157	1.52E-152	98.5	95.6	yes	up
5	Op01g01973	1.384088066	2.64E-24	4.83E-20	24.9	13	yes	up
5	Op05g01076	1.376037626	2.43E-20	4.45E-16	10	3.2	yes	up
5	Op08g01295	1.374225971	2.00E-28	3.67E-24	14.6	4.9	yes	up
5	Op02g01832	1.360723964	6.77E-54	1.24E-49	53.5	31.9	yes	up
5	Op01g01917	1.353361631	5.61E-10	1.03E-05	15.1	8.8	yes	up
5	Op11g01246	1.349294934	5.01E-18	9.16E-14	25.8	15.1	yes	up
5	Op07g01606	1.336256469	2.67E-22	4.88E-18	27.2	15.5	yes	up
5	Op08g00160	1.323807593	7.88E-09	0.0001443	29.1	24.1	yes	up
5	Op02g01646	1.293441877	5.76E-22	1.05E-17	21.1	10.2	yes	up
5	Op02g00017	1.284061687	2.45E-25	4.49E-21	20.2	9	yes	up
5	Op07g01315	1.264501773	5.42E-22	9.92E-18	16.5	6.9	yes	up
5	Op08g00014	1.260059453	1.80E-11	3.30E-07	37.4	31.5	yes	up
5	Op10g01078	1.25861729	3.34E-12	6.11E-08	20.2	12.6	yes	up
5	Op09g00135	1.257341739	7.33E-12	1.34E-07	23.4	15.9	yes	up
5	Op01g01786	1.249669714	2.41E-13	4.41E-09	27.7	19.8	yes	up
5	Op03g01853	1.241851668	1.05E-24	1.92E-20	16.6	6.6	yes	up
5	Op03g02251	1.236270417	3.28E-18	6.00E-14	31.7	20.7	yes	up
5	Op10g01131	1.225266145	4.41E-14	8.07E-10	16.9	9	yes	up
5	Op03g01725	1.219607758	7.60E-10	1.39E-05	17.5	10.9	yes	up
5	Op01g02379	1.216482788	2.95E-15	5.39E-11	25.1	15.9	yes	up
5	Op08g01726	1.208155139	6.85E-07	0.012540684	21.1	15.6	yes	up
5	Op09g01574	1.201081289	1.23E-24	2.25E-20	44.5	31.3	yes	up
5	Op02g01953	1.196270621	2.29E-14	4.19E-10	17.1	9	yes	up
5	Op05g00721	1.182174758	1.02E-12	1.86E-08	12.9	6.2	yes	up
5	Op02g01541	1.179904216	3.02E-20	5.53E-16	18.6	8.8	yes	up
5	Op05g00500	1.168603042	7.75E-10	1.42E-05	16.8	10.1	yes	up
5	Op10g01113	1.164339656	1.59E-08	0.000290364	21.8	15.7	yes	up
5	Op04g00086	1.154107304	4.50E-59	8.24E-55	68	47.4	yes	up
5	Op09g01567	1.15229634	2.37E-14	4.34E-10	10.2	4	yes	up
5	Op05g01442	1.148297393	2.55E-14	4.67E-10	21.2	12.5	yes	up
5	Op03g01729	1.146346293	1.87E-09	3.43E-05	18	11.7	yes	up
5	Op10g01408	1.145617904	5.36E-13	9.80E-09	25.4	16.6	yes	up
5	Op07g00310	1.144045124	8.60E-11	1.57E-06	18.3	11.2	yes	up
5	Op09g00210	1.139380884	1.07E-14	1.96E-10	44.3	39.2	yes	up
5	Op03g00100	1.132611259	1.21E-15	2.22E-11	32.9	23.5	yes	up
5	Op05g01183	1.118697044	2.11E-109	3.87E-105	88.8	70.8	yes	up
5	Op11g00573	1.117808645	5.36E-09	9.81E-05	21.1	14.5	yes	up
5	Op01g01544	1.111784613	6.23E-14	1.14E-09	12.5	5.7	yes	up

5	Op04g00383	1.108910791	3.64E-10	6.66E-06	16.6	10	yes	up
5	Op04g01089	1.105815324	1.71E-11	3.13E-07	31.8	25.4	yes	up
5	Op09g00028	1.104106949	1.12E-17	2.05E-13	20.9	11	yes	up
5	Op05g00264	1.10371522	5.57E-08	0.001018731	13.7	8.3	yes	up
5	Op07g01649	1.096451278	5.42E-09	9.92E-05	18.3	12.1	yes	up
5	Op04g01647	1.094187107	2.91E-11	5.32E-07	22.8	15.2	yes	up
5	Op02g02211	1.092672656	3.56E-10	6.52E-06	15.7	9.3	yes	up
5	Op02g01122	1.092643429	3.04E-07	0.005566552	22.3	17.1	yes	up
5	Op05g01917	1.084113693	1.54E-17	2.82E-13	42.6	32.8	yes	up
5	Op03g00093	1.081955347	1.35E-09	2.47E-05	15.2	9	yes	up
5	Op08g01129	1.080436596	1.72E-09	3.15E-05	11.8	6.2	yes	up
5	Op05g01190	1.070571169	4.38E-134	8.01E-130	97.2	89.6	yes	up
5	Op08g00935	1.068260942	8.29E-16	1.52E-11	14	6.3	yes	up
5	Op05g01180	1.067177396	6.37E-119	1.17E-114	94.5	80	yes	up
5	Op02g00572	1.063867435	2.01E-11	3.68E-07	13.2	6.8	yes	up
5	Op08g01307	1.060451878	9.21E-07	0.016860063	10.9	6.5	yes	up
5	Op07g00099	1.058700053	6.16E-23	1.13E-18	50.6	42	yes	up
5	Op04g00446	1.056726514	1.35E-11	2.47E-07	16	8.9	yes	up
5	Op08g00508	1.053118037	5.74E-12	1.05E-07	21.1	13	yes	up
5	Op11g00044	1.050687836	2.68E-07	0.004909236	18.2	12.8	yes	up
5	Op10g00799	1.045568727	7.31E-09	0.00013387	25.5	19.6	yes	up
5	Op05g01159	1.043506995	2.20E-39	4.02E-35	60.3	44	yes	up
5	Op01g01118	1.038250185	5.25E-07	0.009614923	15.4	10.3	yes	up
5	Op-scaf01g0011	1.038184481	6.69E-08	0.001223726	16.5	10.9	yes	up
5	Op05g01310	1.028930857	4.95E-11	9.06E-07	16.8	9.7	yes	up
5	Op01g00281	1.021437926	2.85E-10	5.21E-06	19.7	12.7	yes	up
5	Op04g01029	1.014333317	4.03E-15	7.38E-11	32.6	23.1	yes	up
5	Op08g01806	1.006430654	3.37E-15	6.16E-11	10.6	4.2	yes	up
6	Op01g00328	2.50072825	0	0	90.7	21.9	yes	up
6	Op10g00663	2.351022312	8.96E-230	1.64E-225	78.1	25.8	yes	up
6	Op06g00877	2.327840624	1.16E-257	2.12E-253	96.8	52.4	yes	up
6	Op05g00593	2.296472033	0	0	78.2	17	yes	up
6	Op02g02321	2.221223255	5.25E-281	9.61E-277	99.8	74.8	yes	up
6	Op10g00651	2.16442781	4.45E-246	8.14E-242	49.3	7.7	yes	up
6	Op05g00642	2.159850179	8.88E-234	1.63E-229	85.1	31.4	yes	up
6	Op07g01948	2.1537647	2.88E-267	5.27E-263	94.6	39.4	yes	up
6	Op04g00320	2.151395326	1.11E-271	2.04E-267	87.8	28.2	yes	up
6	Op06g00847	2.137081435	9.15E-261	1.68E-256	89.6	32.1	yes	up
6	Op05g01823	2.120615384	4.63E-228	8.47E-224	88.5	33.8	yes	up
6	Op02g01646	2.108412989	0	0	57.4	7.5	yes	up
6	Op01g02110	2.107956725	6.99E-209	1.28E-204	70.1	19.8	yes	up
6	Op01g02314	2.066643056	4.05E-224	7.42E-220	83.7	29.5	yes	up
6	Op05g00074	2.057876299	1.67E-234	3.06E-230	96	50.4	yes	up
6	Op01g02037	2.047597908	8.30E-259	1.52E-254	77.9	20.7	yes	up
6	Op05g01159	2.047591632	3.74E-245	6.84E-241	92.2	41.7	yes	up
6	Op03g00685	2.037113603	5.20E-242	9.52E-238	93.4	42.7	yes	up
6	Op01g01909	2.015612431	1.31E-279	2.40E-275	99.4	59.2	yes	up
6	Op04g00231	1.98274939	2.24E-233	4.10E-229	73	19.2	yes	up
6	Op09g00742	1.937160857	3.72E-213	6.81E-209	69.8	19.2	yes	up
6	Op10g00439	1.933946045	2.96E-224	5.42E-220	88.6	34.9	yes	up
6	Op03g01997	1.93193993	1.19E-270	2.18E-266	87	26.6	yes	up
6	Op06g00379	1.915330459	1.04E-217	1.91E-213	87.5	34.2	yes	up
6	Op02g01954	1.905917233	3.34E-242	6.12E-238	98.1	60.2	yes	up
6	Op09g01292	1.896247578	1.12E-249	2.05E-245	96.5	42	yes	up
6	Op01g02355	1.887588581	3.15E-209	5.77E-205	94.7	54.5	yes	up
6	Op07g01109	1.886641752	1.29E-236	2.36E-232	81.6	25.2	yes	up
6	Op10g00879	1.879095995	1.04E-230	1.90E-226	75.5	20.9	yes	up
6	Op02g01921	1.832789018	1.39E-256	2.55E-252	85.3	25.7	yes	up
6	Op02g02357	1.824833061	4.62E-220	8.46E-216	89.8	35.6	yes	up
6	Op04g00086	1.81336495	6.83E-223	1.25E-218	95	45.5	yes	up
6	Op07g00385	1.807083189	1.07E-266	1.96E-262	99.7	72.8	yes	up
6	Op10g01447	1.76961997	2.18E-182	3.99E-178	84	35.4	yes	up

6	Op02g01832	1.76808661	1.33E-221	2.44E-217	85.3	29.7	yes	up
6	Op05g00799	1.767416687	1.71E-155	3.12E-151	79.2	33.7	yes	up
6	Op09g00344	1.760008787	5.08E-243	9.30E-239	73.6	18.4	yes	up
6	Op09g00581	1.75253966	3.54E-264	6.48E-260	99.4	85.9	yes	up
6	Op05g00229	1.740024714	2.49E-170	4.56E-166	75.5	27.2	yes	up
6	Op06g00273	1.731593715	1.05E-233	1.92E-229	71	17.9	yes	up
6	Op09g01525	1.723713233	1.56E-212	2.85E-208	81.9	25.9	yes	up
6	Op10g00783	1.702663341	5.31E-176	9.72E-172	65.1	18.7	yes	up
6	Op08g01484	1.678652785	1.10E-194	2.02E-190	72.5	20.8	yes	up
6	Op03g00100	1.674424152	3.69E-188	6.76E-184	70.7	20.8	yes	up
6	Op07g01186	1.64487309	2.66E-222	4.87E-218	99.8	74.9	yes	up
6	Op01g02170	1.644575165	2.11E-178	3.86E-174	88.6	43	yes	up
6	Op01g01727	1.618829482	9.37E-177	1.72E-172	87	39.5	yes	up
6	Op03g00242	1.598625031	7.22E-142	1.32E-137	72.6	28	yes	up
6	Op07g00099	1.589522241	3.76E-195	6.89E-191	88.2	39.3	yes	up
6	Op06g00376	1.588796163	1.09E-127	2.00E-123	63.4	22.7	yes	up
6	Op09g01293	1.577476138	1.28E-163	2.35E-159	36.5	6	yes	up
6	Op01g02354	1.563680785	1.27E-202	2.33E-198	56	11.9	yes	up
6	Op06g01440	1.562588623	1.28E-183	2.33E-179	89.6	37.6	yes	up
6	Op09g00448	1.551980066	5.42E-189	9.93E-185	93.3	43.1	yes	up
6	Op03g01725	1.534844483	4.75E-150	8.69E-146	43	9	yes	up
6	Op04g01029	1.533897358	2.50E-203	4.57E-199	72.5	20.2	yes	up
6	Op08g01349	1.524359336	2.81E-217	5.14E-213	60.3	12.7	yes	up
6	Op01g00068	1.522867796	7.64E-227	1.40E-222	72.3	18.1	yes	up
6	Op07g01910	1.522619821	5.54E-205	1.01E-200	62.1	14.2	yes	up
6	Op11g00573	1.509532899	2.02E-123	3.69E-119	47.4	12.6	yes	up
6	Op07g01874	1.476538381	8.14E-89	1.49E-84	33.1	8.4	yes	up
6	Op08g01205	1.470315943	5.59E-189	1.02E-184	86.7	33.2	yes	up
6	Op07g01294	1.464326735	4.47E-142	8.18E-138	89.3	46.4	yes	up
6	Op02g00188	1.463189886	4.38E-167	8.03E-163	46.7	9.6	yes	up
6	Op04g00125	1.458160218	9.16E-136	1.68E-131	60.6	19	yes	up
6	Op10g00392	1.45101652	1.24E-124	2.26E-120	92.3	61.5	yes	up
6	Op05g00185	1.450155701	1.87E-133	3.43E-129	60.5	19.7	yes	up
6	Op11g01246	1.444439377	2.19E-127	4.01E-123	49.6	13.4	yes	up
6	Op01g00694	1.435562669	3.03E-151	5.54E-147	61.3	17.7	yes	up
6	Op06g01396	1.432038405	6.64E-139	1.22E-134	72.6	27.4	yes	up
6	Op07g01195	1.431652234	2.68E-134	4.91E-130	52.5	14.4	yes	up
6	Op07g01253	1.42694179	1.10E-140	2.02E-136	43.2	9.5	yes	up
6	Op07g01906	1.424368726	1.62E-181	2.96E-177	62.1	15.7	yes	up
6	Op03g00616	1.421440526	2.62E-126	4.80E-122	69	25.6	yes	up
6	Op09g00209	1.418330709	5.95E-126	1.09E-121	96	71.6	yes	up
6	Op01g02234	1.41062198	5.15E-118	9.43E-114	38.7	8.9	yes	up
6	Op09g01032	1.40289395	2.07E-143	3.79E-139	42.7	9.2	yes	up
6	Op02g02330	1.399812645	8.67E-198	1.59E-193	56	11.7	yes	up
6	Op08g00792	1.397388643	1.13E-167	2.07E-163	61	16.1	yes	up
6	Op03g00777	1.38732428	2.02E-152	3.70E-148	71	23.4	yes	up
6	Op07g00299	1.382498978	2.85E-159	5.22E-155	91	48.9	yes	up
6	Op07g01371	1.38136448	2.43E-152	4.45E-148	98.6	73	yes	up
6	Op02g01535	1.381207479	1.49E-162	2.73E-158	56.6	14.6	yes	up
6	Op03g01899	1.377723646	1.90E-186	3.48E-182	35.2	4.7	yes	up
6	Op08g00014	1.371716527	3.09E-62	5.65E-58	58.7	30	yes	up
6	Op06g00731	1.357326676	1.75E-143	3.21E-139	64.5	20.4	yes	up
6	Op11g00333	1.335703703	6.17E-74	1.13E-69	35.2	10.7	yes	up
6	Op04g00442	1.33255534	8.87E-83	1.62E-78	80.5	44	yes	up
6	Op09g00904	1.323174078	1.38E-128	2.52E-124	83.4	41.6	yes	up
6	Op02g02244	1.320542679	2.48E-170	4.54E-166	84.6	32.7	yes	up
6	Op05g00326	1.318432724	1.30E-173	2.38E-169	90.6	38.8	yes	up
6	Op05g00583	1.314130613	7.63E-131	1.40E-126	45.1	10.9	yes	up
6	Op06g01084	1.309047838	2.33E-155	4.27E-151	42.9	8.4	yes	up
6	Op10g00288	1.30324812	1.25E-117	2.29E-113	82.4	40.1	yes	up
6	Op07g00406	1.302427009	4.43E-148	8.11E-144	58.6	16.3	yes	up
6	Op11g00923	1.298284016	3.89E-114	7.12E-110	35.5	7.8	yes	up

6	Op01g01303	1.298164848	8.27E-120	1.51E-115	97.4	82.6	yes	up
6	Op07g00513	1.295320254	7.46E-81	1.37E-76	60.8	26.6	yes	up
6	Op07g00888	1.280865791	1.43E-164	2.61E-160	70.6	21.4	yes	up
6	Op09g01168	1.280081523	8.61E-138	1.58E-133	70.6	25.7	yes	up
6	Op02g01936	1.276622883	4.43E-170	8.10E-166	67.4	18.9	yes	up
6	Op10g00377	1.275580966	1.30E-176	2.38E-172	53.9	12.1	yes	up
6	Op02g02122	1.265277965	3.61E-122	6.61E-118	60.6	20.5	yes	up
6	Op08g01596	1.260719074	1.14E-160	2.08E-156	70.1	21.6	yes	up
6	Op10g00703	1.260242623	5.39E-72	9.86E-68	20.3	4	yes	up
6	Op06g00519	1.255819753	5.39E-157	9.86E-153	96.5	74.7	yes	up
6	Op01g00677	1.250699325	7.02E-123	1.29E-118	90.1	48.2	yes	up
6	Op02g01996	1.247126935	1.26E-164	2.31E-160	49.4	10.9	yes	up
6	Op04g00410	1.247114819	1.91E-152	3.49E-148	40.5	7.7	yes	up
6	Op07g00359	1.246530205	2.23E-138	4.08E-134	92.6	51.1	yes	up
6	Op02g02170	1.244277208	4.07E-12	7.45E-08	20.2	11.2	yes	up
6	Op07g00353	1.239485905	1.98E-126	3.63E-122	94.1	57.7	yes	up
6	Op06g00339	1.235863595	1.65E-108	3.03E-104	45.6	12.6	yes	up
6	Op08g00935	1.232039637	7.25E-164	1.33E-159	33.8	4.9	yes	up
6	Op01g01491	1.228830732	3.88E-118	7.10E-114	73.3	31.5	yes	up
6	Op10g00482	1.2281234	4.61E-158	8.43E-154	96.8	72.3	yes	up
6	Op11g01192	1.227477289	8.41E-130	1.54E-125	96.2	72.9	yes	up
6	Op01g00093	1.223461635	1.65E-120	3.02E-116	93.6	55	yes	up
6	Op03g02195	1.218422918	1.67E-123	3.07E-119	55.8	17.5	yes	up
6	Op07g00384	1.215775788	8.53E-108	1.56E-103	26.2	4.5	yes	up
6	Op04g01647	1.205716661	1.64E-121	3.00E-117	49	13.3	yes	up
6	Op07g01545	1.187007093	6.21E-112	1.14E-107	68.5	27.2	yes	up
6	Op06g00696	1.183393709	2.89E-97	5.29E-93	44.3	13.3	yes	up
6	Op02g01364	1.179299147	6.32E-134	1.16E-129	74.6	28.1	yes	up
6	Op05g00895	1.174706099	4.66E-123	8.53E-119	61.6	20.3	yes	up
6	Op10g00116	1.170826498	2.18E-89	3.99E-85	59.4	23.9	yes	up
6	Op07g00119	1.170327804	1.17E-135	2.14E-131	59.2	17.8	yes	up
6	Op08g00250	1.169565945	1.71E-149	3.13E-145	18.2	1.4	yes	up
6	Op06g00645	1.151566715	1.71E-86	3.13E-82	45.9	15.3	yes	up
6	Op01g00352	1.146081096	5.52E-85	1.01E-80	56.8	22	yes	up
6	Op05g01337	1.136764377	4.56E-75	8.34E-71	31.7	8.7	yes	up
6	Op06g00783	1.116370337	7.22E-57	1.32E-52	42.6	16.9	yes	up
6	Op03g00081	1.113708186	6.70E-69	1.23E-64	68.8	37	yes	up
6	Op06g00378	1.109656487	5.83E-79	1.07E-74	51.5	19.9	yes	up
6	Op01g02359	1.109417363	4.30E-148	7.86E-144	62.4	17.9	yes	up
6	Op10g01497	1.108888772	3.18E-82	5.81E-78	31.4	8.1	yes	up
6	Op02g01953	1.103566316	5.84E-130	1.07E-125	37.3	7.6	yes	up
6	Op10g01428	1.103362075	2.25E-120	4.12E-116	91.4	54.6	yes	up
6	Op09g00182	1.101733982	2.62E-129	4.80E-125	52.6	14.6	yes	up
6	Op08g01334	1.099493442	4.56E-98	8.34E-94	64.3	26.1	yes	up
6	Op05g00632	1.098894215	6.81E-103	1.25E-98	79	39.3	yes	up
6	Op02g02040	1.097484369	2.84E-101	5.21E-97	40.8	10.8	yes	up
6	Op06g00732	1.095989359	5.30E-75	9.70E-71	34.4	10.2	yes	up
6	Op03g01306	1.091270231	8.16E-86	1.49E-81	59.4	24.1	yes	up
6	Op02g02227	1.083431431	1.05E-147	1.93E-143	35.2	5.9	yes	up
6	Op02g01212	1.078661801	2.72E-127	4.98E-123	36.2	7.2	yes	up
6	Op02g02038	1.077441	9.09E-83	1.66E-78	70.4	34.1	yes	up
6	Op08g00925	1.076108757	1.18E-124	2.17E-120	58.1	17.9	yes	up
6	Op11g00206	1.072892465	2.00E-111	3.66E-107	92.6	61	yes	up
6	Op02g00378	1.071610561	7.45E-104	1.36E-99	37.9	9.5	yes	up
6	Op10g01544	1.071472537	5.15E-66	9.42E-62	22.7	5.3	yes	up
6	Op02g00118	1.070591573	1.60E-159	2.93E-155	99.2	81.6	yes	up
6	Op07g01608	1.066898425	2.98E-101	5.46E-97	56.6	20.4	yes	up
6	Op05g00906	1.064254321	5.98E-114	1.10E-109	49	13.6	yes	up
6	Op11g00881	1.063095973	1.14E-106	2.09E-102	67.7	25.6	yes	up
6	Op10g01127	1.061071472	4.30E-109	7.86E-105	61.4	22.1	yes	up
6	Op03g02053	1.057505047	1.85E-90	3.39E-86	81.9	43.2	yes	up
6	Op04g00446	1.052240825	3.33E-127	6.10E-123	36.8	7.4	yes	up

6	Op01g00485	1.050400811	7.41E-96	1.36E-91	79.8	39.4	yes	up
6	Op07g00310	1.049313817	1.50E-50	2.75E-46	30.1	10.3	yes	up
6	Op03g02182	1.04852241	6.87E-114	1.26E-109	52.5	16	yes	up
6	Op02g02192	1.046834957	3.45E-136	6.32E-132	26.1	3.5	yes	up
6	Op02g00164	1.043023524	2.49E-73	4.55E-69	93.3	65.5	yes	up
6	Op07g01674	1.042308023	2.16E-149	3.95E-145	22.4	2.2	yes	up
6	Op06g01586	1.039914013	2.30E-130	4.22E-126	47.4	11.8	yes	up
6	Op03g01965	1.038234635	1.67E-98	3.05E-94	55.7	19.2	yes	up
6	Op07g01230	1.037824366	2.94E-31	5.39E-27	70.4	47.8	yes	up
6	Op01g00212	1.031893125	1.17E-52	2.15E-48	21	5.4	yes	up
6	Op10g01408	1.02465874	2.04E-45	3.74E-41	37.3	15.8	yes	up
6	Op09g00271	1.020577718	3.60E-81	6.59E-77	31.2	7.9	yes	up
6	Op06g00639	1.01327662	1.20E-96	2.19E-92	89	53.1	yes	up
6	Op04g00632	1.011695362	1.38E-88	2.53E-84	75.7	37.2	yes	up
6	Op07g01148	1.011573649	2.66E-97	4.88E-93	88	50.1	yes	up
6	Op09g01507	1.009370552	3.45E-29	6.31E-25	46.6	26.5	yes	up
6	Op01g00358	1.007705343	1.38E-63	2.52E-59	90.1	64.2	yes	up
6	Op09g01490	1.001298557	5.51E-105	1.01E-100	27.8	5.2	yes	up
6	Op08g01155	1.001130358	1.91E-76	3.50E-72	84.6	51.2	yes	up
7	Op06g01361	5.199557718	1.71E-254	3.14E-250	49.2	7.9	yes	up
7	Op04g00648	4.111082928	0	0	25.3	0.8	yes	up
7	Op09g00202	3.521781208	0	0	43.2	1.4	yes	up
7	Op02g01257	3.174643831	3.13E-199	5.74E-195	46.2	8.7	yes	up
7	Op10g00279	2.971387545	0	0	47.6	3.2	yes	up
7	Op07g00450	2.955380353	0	0	30.5	1.1	yes	up
7	Op01g02497	2.944602579	2.64E-266	4.83E-262	27.1	1.5	yes	up
7	Op06g00332	2.936875329	9.56E-97	1.75E-92	27.6	5.8	yes	up
7	Op04g01262	2.749710817	3.31E-272	6.06E-268	31.2	2.2	yes	up
7	Op01g00532	2.717147952	6.01E-262	1.10E-257	49.6	7.5	yes	up
7	Op09g00974	2.714165623	1.41E-106	2.59E-102	13.1	1	yes	up
7	Op09g01389	2.667561817	0	0	44.7	1.4	yes	up
7	Op06g01554	2.660454531	1.79E-155	3.27E-151	11.7	0.3	yes	up
7	Op02g00068	2.591033432	0	0	46.2	4.2	yes	up
7	Op05g01907	2.565664168	2.63E-292	4.81E-288	22.9	0.7	yes	up
7	Op08g00591	2.485672672	0	0	36.5	1.1	yes	up
7	Op10g00482	2.479008277	1.66E-25	3.03E-21	79.7	73.5	yes	up
7	Op04g01590	2.466216303	1.33E-157	2.43E-153	21.6	1.9	yes	up
7	Op09g00066	2.114434162	1.15E-85	2.10E-81	38.7	12	yes	up
7	Op08g01720	2.006466957	2.09E-209	3.83E-205	18.3	0.7	yes	up
7	Op05g00415	1.998894261	8.19E-229	1.50E-224	35.8	4	yes	up
7	Op02g00107	1.967747433	8.68E-108	1.59E-103	68	33.8	yes	up
7	Op03g00614	1.963921559	1.14E-127	2.09E-123	60.8	23.3	yes	up
7	Op04g01502	1.951547612	3.47E-82	6.35E-78	79.6	58.1	yes	up
7	Op03g01949	1.943518742	8.51E-258	1.56E-253	21.4	0.7	yes	up
7	Op05g01279	1.936554094	5.34E-243	9.77E-239	32.5	2.9	yes	up
7	Op04g01457	1.9356563	4.27E-295	7.81E-291	26.5	1.1	yes	up
7	Op01g01580	1.919516737	1.06E-108	1.94E-104	13.2	1	yes	up
7	Op09g01367	1.892337589	3.64E-118	6.67E-114	32	6.3	yes	up
7	Op09g00651	1.890645307	6.16E-81	1.13E-76	52.3	23.2	yes	up
7	Op06g00056	1.889350176	7.35E-19	1.35E-14	21.4	10.4	yes	up
7	Op06g01216	1.873858237	1.15E-276	2.10E-272	41.4	4.3	yes	up
7	Op05g01942	1.865207798	3.75E-66	6.87E-62	24.1	6.1	yes	up
7	Op09g00794	1.860624816	1.66E-219	3.03E-215	20.6	0.9	yes	up
7	Op03g01486	1.821709663	0	0	28.6	1	yes	up
7	Op03g01327	1.777540024	2.31E-61	4.22E-57	10.2	1.2	yes	up
7	Op07g01671	1.75428839	3.94E-130	7.21E-126	43.2	10.5	yes	up
7	Op01g01625	1.74916734	1.33E-90	2.43E-86	46.9	17.1	yes	up
7	Op07g00348	1.690168733	3.62E-217	6.63E-213	28.5	2.4	yes	up
7	Op02g02307	1.679566203	1.75E-103	3.20E-99	36.7	9.2	yes	up
7	Op10g00639	1.672938927	5.37E-127	9.83E-123	38.4	8.5	yes	up
7	Op08g01207	1.671603043	1.26E-94	2.30E-90	25.6	4.9	yes	up
7	Op07g00458	1.611502217	6.55E-196	1.20E-191	20.9	1.2	yes	up

7	Op09g00150	1.583120755	4.18E-184	7.66E-180	31	3.6	yes	up
7	Op04g00044	1.57019367	4.86E-79	8.89E-75	38.2	12.3	yes	up
7	Op08g01574	1.569160426	6.44E-99	1.18E-94	72.2	42.4	yes	up
7	Op01g00884	1.55422547	9.39E-235	1.72E-230	26.6	1.8	yes	up
7	Op11g00888	1.539841583	2.52E-21	4.61E-17	48.9	36.2	yes	up
7	Op07g01756	1.499563687	1.19E-81	2.18E-77	14.2	1.7	yes	up
7	Op05g00301	1.476094397	2.47E-67	4.53E-63	35.7	11.9	yes	up
7	Op07g00885	1.464784538	1.80E-219	3.29E-215	21.1	1	yes	up
7	Op01g02034	1.463888636	5.50E-169	1.01E-164	14.4	0.5	yes	up
7	Op06g01456	1.462312329	3.79E-152	6.94E-148	19.8	1.6	yes	up
7	Op05g00090	1.455515531	3.72E-213	6.80E-209	29	2.5	yes	up
7	Op01g02227	1.444641478	8.81E-64	1.61E-59	27	7.5	yes	up
7	Op04g00368	1.432219927	2.30E-33	4.21E-29	27	11.1	yes	up
7	Op01g00279	1.430541043	7.61E-43	1.39E-38	33.5	14.6	yes	up
7	Op09g00995	1.426369007	2.45E-250	4.49E-246	21.8	0.8	yes	up
7	Op01g01626	1.407216767	5.13E-67	9.40E-63	32.3	10.1	yes	up
7	Op07g00395	1.395645853	4.45E-36	8.15E-32	14.2	3.6	yes	up
7	Op02g02121	1.393998433	2.92E-46	5.35E-42	38.4	17	yes	up
7	Op07g00025	1.370199503	1.10E-124	2.01E-120	27.1	4.2	yes	up
7	Op06g01221	1.363208525	2.86E-94	5.23E-90	36.7	9.6	yes	up
7	Op01g00793	1.360988196	3.77E-53	6.91E-49	32	11.6	yes	up
7	Op03g01412	1.355715249	3.43E-90	6.27E-86	34.7	9.1	yes	up
7	Op07g01177	1.354971392	9.92E-217	1.82E-212	22.8	1.3	yes	up
7	Op03g02142	1.346959526	1.36E-141	2.49E-137	20.4	1.9	yes	up
7	Op09g00288	1.346206997	1.91E-232	3.50E-228	20.6	0.8	yes	up
7	Op03g02167	1.341000363	2.64E-108	4.84E-104	19.8	2.5	yes	up
7	Op02g01907	1.335625869	1.10E-61	2.02E-57	39	15.1	yes	up
7	Op05g01788	1.334247163	1.83E-168	3.35E-164	26.5	2.8	yes	up
7	Op10g01480	1.332501782	3.16E-38	5.79E-34	37.4	18.7	yes	up
7	Op10g01449	1.331772108	1.11E-35	2.03E-31	32.2	15	yes	up
7	Op03g00333	1.328496927	2.00E-251	3.66E-247	20.8	0.7	yes	up
7	Op09g00756	1.326166552	1.09E-138	1.99E-134	15.7	1	yes	up
7	Op01g02469	1.305416266	1.57E-62	2.87E-58	30.2	9.5	yes	up
7	Op07g01759	1.30190149	1.07E-26	1.97E-22	37	21.3	yes	up
7	Op09g00598	1.298242117	3.36E-79	6.16E-75	14.9	1.9	yes	up
7	Op03g00280	1.289223738	3.15E-158	5.77E-154	10.7	0.2	yes	up
7	Op07g01563	1.280773757	1.83E-84	3.34E-80	24.1	4.8	yes	up
7	Op02g00725	1.27163953	1.46E-197	2.68E-193	20.8	1.2	yes	up
7	Op05g01314	1.270316668	6.51E-55	1.19E-50	36.7	13.9	yes	up
7	Op11g00354	1.264730548	7.91E-30	1.45E-25	21.4	8.4	yes	up
7	Op02g00363	1.257875822	1.33E-89	2.44E-85	93.6	83.7	yes	up
7	Op06g00235	1.246717373	2.91E-51	5.32E-47	35	13.8	yes	up
7	Op04g00235	1.24016721	4.22E-101	7.73E-97	94	79.8	yes	up
7	Op06g00216	1.238121554	1.55E-59	2.84E-55	28	8.2	yes	up
7	Op02g02331	1.231285053	3.97E-152	7.26E-148	18.9	1.4	yes	up
7	Op06g00467	1.226723403	2.81E-40	5.14E-36	23.6	8.1	yes	up
7	Op03g02271	1.225312749	1.20E-51	2.19E-47	58.1	36.1	yes	up
7	Op01g01321	1.219881059	2.70E-199	4.93E-195	14.6	0.3	yes	up
7	Op01g00754	1.213052484	1.79E-49	3.27E-45	32	11.8	yes	up
7	Op02g00935	1.202624367	1.06E-63	1.93E-59	34.7	11.7	yes	up
7	Op06g00319	1.200266017	1.02E-116	1.87E-112	15.1	1.2	yes	up
7	Op07g01014	1.191747368	9.04E-167	1.65E-162	18.9	1.2	yes	up
7	Op10g00391	1.175696811	3.68E-56	6.73E-52	30	9.9	yes	up
7	Op10g00272	1.172571046	2.89E-132	5.29E-128	13.6	0.7	yes	up
7	Op05g00651	1.170427661	4.15E-67	7.59E-63	16.2	2.7	yes	up
7	Op08g01085	1.168234105	1.29E-54	2.35E-50	16.6	3.4	yes	up
7	Op06g00164	1.165669613	2.80E-95	5.12E-91	19.9	2.9	yes	up
7	Op04g00063	1.163563062	4.74E-211	8.68E-207	18.3	0.7	yes	up
7	Op03g00153	1.156988712	2.61E-180	4.79E-176	16.8	0.7	yes	up
7	Op01g00750	1.153913201	6.50E-10	1.19E-05	54.1	49	yes	up
7	Op06g00688	1.141275519	2.77E-155	5.07E-151	11.7	0.3	yes	up
7	Op08g01331	1.135788945	1.02E-40	1.86E-36	10.4	1.8	yes	up

7	Op03g01482	1.13164198	2.87E-69	5.25E-65	32.8	9.9	yes	up
7	Op05g01923	1.129553222	1.80E-46	3.29E-42	56.1	33.3	yes	up
7	Op03g01716	1.128777628	6.98E-39	1.28E-34	20.3	6.4	yes	up
7	Op03g00177	1.125101398	2.05E-157	3.75E-153	11.7	0.3	yes	up
7	Op02g01555	1.122711058	7.56E-35	1.38E-30	24.3	9.2	yes	up
7	Op11g00395	1.118935918	4.19E-42	7.67E-38	42.7	21.5	yes	up
7	Op05g01664	1.115050279	2.36E-210	4.31E-206	12.7	0.1	yes	up
7	Op08g01330	1.11465601	7.95E-26	1.46E-21	11.7	3.3	yes	up
7	Op03g00746	1.099387285	8.67E-51	1.59E-46	27.8	9.3	yes	up
7	Op02g02337	1.095229955	1.32E-82	2.41E-78	17.9	2.7	yes	up
7	Op06g01565	1.088958256	1.61E-73	2.96E-69	25	5.7	yes	up
7	Op08g01248	1.081160998	9.19E-45	1.68E-40	30	11.1	yes	up
7	Op02g00035	1.077980761	1.15E-38	2.11E-34	22.4	7.6	yes	up
7	Op05g01576	1.077286382	3.55E-71	6.49E-67	12.6	1.5	yes	up
7	Op11g01283	1.076547479	2.61E-123	4.78E-119	19.8	2.1	yes	up
7	Op03g01600	1.076165366	9.07E-151	1.66E-146	14.7	0.7	yes	up
7	Op03g01301	1.07566177	8.83E-45	1.62E-40	23.8	7.6	yes	up
7	Op07g00545	1.062598025	1.42E-61	2.60E-57	18.3	3.7	yes	up
7	Op07g00264	1.059127811	1.37E-08	0.000249988	57.6	54.1	yes	up
7	Op02g01831	1.058065319	1.72E-41	3.14E-37	24.6	8.5	yes	up
7	Op10g00882	1.044136382	1.59E-32	2.91E-28	37.7	19.3	yes	up
7	Op07g01836	1.043609426	1.02E-102	1.87E-98	19.3	2.5	yes	up
7	Op10g01246	1.026575691	2.30E-119	4.21E-115	13.7	0.9	yes	up
7	Op04g01429	1.026415166	6.68E-36	1.22E-31	44.4	23.9	yes	up
7	Op02g02286	1.020614295	9.81E-171	1.80E-166	16.6	0.8	yes	up
7	Op06g00574	1.014751666	4.75E-88	8.69E-84	19.6	3	yes	up
7	Op05g00757	1.0052554	4.03E-36	7.39E-32	48.9	29.1	yes	up
7	Op08g01252	1.004677316	2.34E-43	4.29E-39	18.8	5.1	yes	up
7	Op05g00205	1.004509335	1.20E-95	2.20E-91	16.6	1.9	yes	up
7	Op09g01397	1.003034412	6.63E-48	1.21E-43	19.8	5.3	yes	up
7	Op04g00147	1.002907881	2.74E-49	5.01E-45	80.1	68.7	yes	up
7	Op04g01458	1.001209813	6.53E-144	1.19E-139	13.2	0.6	yes	up
8	Op06g01441	1.67736845	1.23E-179	2.25E-175	96.3	54.8	yes	up
8	Op02g01733	1.523783399	1.95E-226	3.57E-222	99.8	94.1	yes	up
8	Op10g00124	1.454356217	8.97E-157	1.64E-152	94.9	56.4	yes	up
8	Op05g00187	1.449882182	1.30E-185	2.38E-181	97.3	60.6	yes	up
8	Op07g01580	1.400471021	2.36E-229	4.31E-225	100	93.2	yes	up
8	Op05g01779	1.280978283	1.91E-219	3.49E-215	100	95.2	yes	up
8	Op11g00376	1.242225686	4.05E-223	7.42E-219	99.8	90.3	yes	up
8	Op02g00520	1.240106854	2.14E-174	3.91E-170	98.6	86	yes	up
8	Op01g00153	1.216425404	1.85E-227	3.39E-223	99.8	93.2	yes	up
8	Op10g00972	1.17018262	2.12E-188	3.88E-184	100	98.7	yes	up
8	Op09g00086	1.167843904	8.49E-216	1.55E-211	100	99	yes	up
8	Op07g00102	1.167800689	7.28E-120	1.33E-115	84.5	38.4	yes	up
8	Op02g01245	1.160375826	1.97E-144	3.61E-140	99.6	95.8	yes	up
8	Op07g01717	1.15903555	2.31E-236	4.23E-232	100	99.8	yes	up
8	Op06g00093	1.154568023	5.22E-170	9.56E-166	98.9	75.9	yes	up
8	Op07g00328	1.14980219	1.09E-91	2.00E-87	51.8	17.1	yes	up
8	Op02g02174	1.140297868	1.83E-115	3.35E-111	64.1	21.8	yes	up
8	Op03g01720	1.131586945	8.29E-173	1.52E-168	99.6	94.2	yes	up
8	Op07g01194	1.124635508	4.96E-180	9.08E-176	99.8	91.7	yes	up
8	Op05g01780	1.124446377	6.49E-229	1.19E-224	100	98.9	yes	up
8	Op07g01420	1.123300777	2.21E-210	4.04E-206	99.6	93.1	yes	up
8	Op04g00607	1.122664743	2.87E-137	5.25E-133	94.9	62.1	yes	up
8	Op02g00102	1.119066283	5.68E-132	1.04E-127	97.2	60.5	yes	up
8	Op01g00256	1.112779633	1.71E-107	3.12E-103	88.5	49.7	yes	up
8	Op03g01090	1.10843451	2.47E-88	4.51E-84	60.8	22.4	yes	up
8	Op06g00026	1.10503893	4.91E-106	8.98E-102	85.7	44	yes	up
8	Op02g00043	1.096964868	6.35E-108	1.16E-103	84.5	40.2	yes	up
8	Op05g00519	1.086936149	4.91E-199	8.99E-195	99.8	93.2	yes	up
8	Op01g00584	1.086006893	2.80E-164	5.12E-160	98.1	78.7	yes	up
8	Op10g00096	1.082121013	2.33E-206	4.27E-202	99.8	94.8	yes	up

8	Op05g01520	1.078056285	5.22E-112	9.55E-108	88.3	45.2	yes	up
8	Op10g01551	1.065193292	5.96E-183	1.09E-178	99.5	91.2	yes	up
8	Op10g00061	1.06426829	1.62E-208	2.96E-204	100	97.1	yes	up
8	Op10g00695	1.061473902	2.04E-186	3.74E-182	100	93.1	yes	up
8	Op10g00452	1.05840779	1.20E-92	2.19E-88	91.3	62.6	yes	up
8	Op08g00265	1.05119724	8.20E-109	1.50E-104	93.6	60.4	yes	up
8	Op08g01142	1.050333427	1.74E-107	3.19E-103	91.5	59	yes	up
8	Op08g01303	1.03056836	1.38E-93	2.52E-89	33.6	7.6	yes	up
8	Op07g02010	1.029485032	3.91E-145	7.16E-141	97.9	82.3	yes	up
8	Op09g01427	1.029003385	5.47E-135	1.00E-130	98.4	83.7	yes	up
8	Op07g00891	1.017413455	3.45E-139	6.31E-135	98.6	81.8	yes	up
8	Op01g01941	1.015820768	5.90E-113	1.08E-108	70.8	25.2	yes	up
8	Op04g01589	1.012365915	3.54E-189	6.47E-185	100	96	yes	up
8	Op07g01604	1.008071333	1.30E-103	2.38E-99	91.5	53.7	yes	up
8	Op08g01155	1.005889063	2.01E-99	3.68E-95	91.7	50.9	yes	up
8	Op01g02104	1.003915397	3.72E-115	6.80E-111	52.1	14.3	yes	up
8	Op09g00493	1.003181366	7.89E-101	1.44E-96	83.7	40.7	yes	up
10	Op06g00829	1.818278295	9.79E-155	1.79E-150	50.1	11.1	yes	up
10	Op06g01440	1.793157852	4.22E-184	7.73E-180	89.1	38	yes	up
10	Op06g01441	1.660611554	6.47E-172	1.18E-167	94.5	55	yes	up
10	Op10g00116	1.610946167	7.53E-134	1.38E-129	67.9	23.7	yes	up
10	Op11g01246	1.510546309	6.88E-84	1.26E-79	44.8	14	yes	up
10	Op08g01155	1.334433903	3.65E-126	6.67E-122	90.7	51.1	yes	up
10	Op10g00124	1.325393943	3.01E-104	5.50E-100	89.1	56.8	yes	up
10	Op02g02038	1.293260475	9.15E-79	1.67E-74	70.9	34.4	yes	up
10	Op02g01733	1.192793279	1.30E-157	2.38E-153	99.8	94.2	yes	up
10	Op03g01939	1.171703852	3.17E-121	5.80E-117	54.8	15.7	yes	up
10	Op01g00093	1.152279816	1.06E-95	1.93E-91	91.1	55.5	yes	up
10	Op09g00403	1.129662629	8.52E-101	1.56E-96	48.8	13.8	yes	up
10	Op02g00118	1.124524041	8.98E-147	1.64E-142	98.2	81.8	yes	up
10	Op02g00520	1.051128518	4.76E-117	8.72E-113	97.4	86.1	yes	up
10	Op10g00845	1.047832421	4.37E-116	8.00E-112	31	5.4	yes	up
10	Op03g01725	1.040143495	3.77E-90	6.90E-86	38.1	9.7	yes	up
10	Op10g00972	1.031474228	4.81E-123	8.81E-119	100	98.7	yes	up
10	Op04g00442	1.027475372	1.47E-55	2.69E-51	77.4	44.5	yes	up
10	Op07g01717	1.019143564	2.08E-172	3.80E-168	100	99.8	yes	up
10	Op06g00268	1.012008182	3.63E-67	6.64E-63	69.9	34.2	yes	up
11	Op09g01504	3.578769649	0	0	87.1	21	yes	up
11	Op04g01661	3.311392023	7.82E-304	1.43E-299	99.5	39.5	yes	up
11	Op01g01864	3.254833102	1.79E-242	3.28E-238	88.4	29.3	yes	up
11	Op04g01662	3.178996066	1.49E-242	2.73E-238	100	56.4	yes	up
11	Op06g00246	3.079424747	0	0	83	13.9	yes	up
11	Op02g01325	2.933109716	0	0	70.7	4.5	yes	up
11	Op02g01004	2.739267088	0	0	65.8	4.7	yes	up
11	Op04g00207	2.695836837	1.15E-230	2.10E-226	67.8	13.8	yes	up
11	Op01g01213	2.676711015	1.36E-262	2.49E-258	99.3	49.5	yes	up
11	Op01g01866	2.658999669	3.56E-175	6.52E-171	94.6	54.1	yes	up
11	Op11g01298	2.527425159	0	0	39.7	2.3	yes	up
11	Op01g00473	2.305557486	1.06E-199	1.94E-195	98	52.1	yes	up
11	Op01g00892	2.186389386	4.33E-131	7.93E-127	47.2	10.4	yes	up
11	Op01g02256	2.124222264	2.29E-222	4.19E-218	99.8	58.9	yes	up
11	Op09g00262	2.113336815	6.74E-242	1.23E-237	58.3	8.7	yes	up
11	Op06g00264	2.049545595	6.92E-123	1.27E-118	66	21.8	yes	up
11	Op05g01314	2.029988552	2.18E-128	3.99E-124	54.2	13.4	yes	up
11	Op04g01429	1.986058811	1.37E-228	2.52E-224	85.5	22.2	yes	up
11	Op05g01044	1.974824635	6.92E-135	1.27E-130	73.7	26.1	yes	up
11	Op11g00146	1.900979331	4.06E-206	7.43E-202	64.4	12.4	yes	up
11	Op07g01445	1.867588909	7.22E-126	1.32E-121	77.8	31.4	yes	up
11	Op05g00422	1.83189666	1.42E-278	2.60E-274	44.2	3.8	yes	up
11	Op08g01716	1.786853765	5.53E-115	1.01E-110	88.2	49.4	yes	up
11	Op09g00032	1.752639374	8.14E-178	1.49E-173	100	78.5	yes	up
11	Op03g01708	1.736051067	1.58E-154	2.89E-150	55.8	12.2	yes	up

11	Op11g00881	1.712388166	9.45E-166	1.73E-161	83.2	25.7	yes	up
11	Op06g00453	1.697761362	3.27E-122	5.99E-118	60.5	17.9	yes	up
11	Op07g01759	1.652948398	1.05E-173	1.92E-169	74.4	19.7	yes	up
11	Op11g01023	1.626258997	1.73E-170	3.16E-166	67.6	16.8	yes	up
11	Op02g01796	1.604793484	3.92E-154	7.18E-150	39.7	5.9	yes	up
11	Op04g01423	1.51003278	7.51E-159	1.37E-154	51.9	9.7	yes	up
11	Op09g01545	1.477391041	1.40E-166	2.57E-162	52.8	9.7	yes	up
11	Op08g01025	1.435149447	1.02E-126	1.86E-122	47.4	10.4	yes	up
11	Op11g00162	1.359965841	1.88E-104	3.45E-100	70.1	26.1	yes	up
11	Op05g00656	1.342363314	7.33E-138	1.34E-133	49.9	10.3	yes	up
11	Op03g02101	1.336587194	1.68E-112	3.07E-108	78.9	31	yes	up
11	Op02g02185	1.332681039	5.16E-101	9.45E-97	58	17.6	yes	up
11	Op07g00679	1.323799778	1.13E-67	2.06E-63	53.7	20.1	yes	up
11	Op06g00205	1.321935307	1.25E-208	2.28E-204	35.1	3.2	yes	up
11	Op06g00503	1.320085877	2.40E-110	4.40E-106	44.9	10.3	yes	up
11	Op08g01574	1.307689732	5.24E-113	9.59E-109	88.9	42.1	yes	up
11	Op09g00658	1.306608001	1.93E-92	3.53E-88	59.2	19.9	yes	up
11	Op10g01553	1.299755022	1.34E-115	2.46E-111	18.6	1.6	yes	up
11	Op02g00036	1.286432077	2.05E-105	3.75E-101	94.3	57.8	yes	up
11	Op06g00033	1.258961388	4.12E-91	7.54E-87	71.4	29.2	yes	up
11	Op05g01633	1.252468829	2.14E-99	3.92E-95	48.5	12.7	yes	up
11	Op04g01502	1.248786365	1.10E-83	2.02E-79	90.9	57.9	yes	up
11	Op01g01082	1.246284103	7.80E-181	1.43E-176	38.5	4.6	yes	up
11	Op02g02262	1.242844599	6.76E-105	1.24E-100	59.4	17.6	yes	up
11	Op10g01263	1.2361782	6.42E-76	1.18E-71	70.7	32.8	yes	up
11	Op01g00485	1.225407118	4.35E-82	7.96E-78	81.4	40.2	yes	up
11	Op09g00651	1.208572479	1.56E-104	2.85E-100	68.5	22.9	yes	up
11	Op11g00254	1.203620581	2.68E-88	4.91E-84	54	16.7	yes	up
11	Op10g00184	1.189684995	2.70E-69	4.94E-65	67.8	31.3	yes	up
11	Op01g01558	1.171064122	2.15E-163	3.94E-159	23.8	1.8	yes	up
11	Op10g00706	1.127635984	8.37E-72	1.53E-67	88.4	54.2	yes	up
11	Op01g02347	1.125756723	8.74E-56	1.60E-51	30.4	8.3	yes	up
11	Op07g01136	1.104012912	2.11E-110	3.86E-106	46	10.2	yes	up
11	Op01g02021	1.097832815	1.22E-90	2.24E-86	31.7	6.2	yes	up
11	Op01g00472	1.09065169	1.56E-61	2.86E-57	43.8	14.7	yes	up
11	Op07g00549	1.083363014	2.11E-63	3.86E-59	33.1	8.7	yes	up
11	Op01g00275	1.082374661	1.55E-71	2.84E-67	59.9	23.8	yes	up
11	Op05g01084	1.080994361	1.34E-86	2.45E-82	45.6	12.6	yes	up
11	Op08g01322	1.065558412	1.05E-79	1.92E-75	29.3	6	yes	up
11	Op08g01027	1.044246374	3.11E-228	5.69E-224	23.1	1	yes	up
11	Op03g01071	1.042656112	1.16E-79	2.13E-75	93.2	62.4	yes	up
11	Op06g00467	1.037511365	3.08E-87	5.64E-83	35.6	7.7	yes	up
11	Op07g01923	1.032251175	1.76E-60	3.23E-56	41.3	13.1	yes	up
11	Op07g01875	1.031374743	3.30E-72	6.05E-68	26.8	5.4	yes	up
11	Op10g00639	1.027512418	2.56E-106	4.69E-102	42.2	8.9	yes	up
11	Op02g01624	1.025030311	3.03E-155	5.55E-151	22.2	1.6	yes	up
11	Op10g01449	1.011405681	2.73E-65	5.00E-61	44.9	14.7	yes	up
11	Op05g00668	1.010943777	1.97E-151	3.60E-147	26.5	2.5	yes	up
11	Op09g00337	1.000959485	6.19E-104	1.13E-99	32	5.4	yes	up
12	Op11g00727	4.066304252	0	0	91	12.2	yes	up
12	Op01g01392	3.946128946	0	0	94.3	13	yes	up
12	Op06g00911	3.182824078	1.24E-256	2.27E-252	67	7.8	yes	up
12	Op08g00079	2.93381219	6.20E-306	1.14E-301	84.9	11.2	yes	up
12	Op07g01569	2.853039539	2.63E-63	4.82E-59	64.2	26.8	yes	up
12	Op05g01342	2.771939911	0	0	62.7	3	yes	up
12	Op03g00092	2.669385144	1.77E-147	3.24E-143	97.5	40.7	yes	up
12	Op06g00154	2.660983003	2.76E-113	5.05E-109	40.9	6.2	yes	up
12	Op07g00537	2.587025258	1.32E-280	2.42E-276	65.9	6.6	yes	up
12	Op07g00401	2.533602244	6.66E-275	1.22E-270	40.9	2.2	yes	up
12	Op10g00760	2.460416195	1.05E-221	1.93E-217	78.9	12.6	yes	up
12	Op07g01208	2.453936802	5.18E-141	9.48E-137	70.3	15.7	yes	up
12	Op05g00359	2.396324056	1.63E-249	2.99E-245	49.8	4	yes	up

12	Op11g00898	2.359535426	7.55E-121	1.38E-116	99.3	92.5	yes	up
12	Op06g00944	2.32802768	2.87E-186	5.26E-182	57.7	7.6	yes	up
12	Op08g01657	2.326273085	1.74E-211	3.19E-207	55.9	6.3	yes	up
12	Op02g02051	2.318844782	1.37E-284	2.51E-280	50.9	3.5	yes	up
12	Op10g00656	2.306975229	9.18E-185	1.68E-180	86	19.6	yes	up
12	Op10g00675	2.210263618	1.37E-144	2.51E-140	90.3	28.6	yes	up
12	Op03g01163	2.194447082	1.25E-201	2.29E-197	79.9	13.9	yes	up
12	Op11g01294	2.177327346	5.29E-41	9.68E-37	44.4	16.3	yes	up
12	Op06g01431	2.157112298	5.36E-140	9.80E-136	28.3	2.3	yes	up
12	Op04g00143	2.154013774	1.13E-38	2.07E-34	15.4	2.4	yes	up
12	Op11g00931	2.146289598	2.22E-131	4.07E-127	84.6	25.7	yes	up
12	Op05g00843	2.109074044	2.09E-142	3.82E-138	71.7	15.6	yes	up
12	Op08g01617	2.040179843	6.71E-179	1.23E-174	61.6	8.9	yes	up
12	Op01g01729	1.954423124	4.51E-72	8.26E-68	65.6	22.7	yes	up
12	Op11g00544	1.939514151	9.25E-111	1.69E-106	84.6	32.1	yes	up
12	Op09g00105	1.93270669	1.81E-139	3.32E-135	63.8	12.1	yes	up
12	Op07g00629	1.930006422	1.34E-105	2.45E-101	47	8.3	yes	up
12	Op04g01267	1.9115279	1.24E-147	2.27E-143	25.4	1.7	yes	up
12	Op06g00314	1.91002971	1.99E-138	3.64E-134	83.2	21.8	yes	up
12	Op09g00994	1.854722034	3.94E-173	7.21E-169	44.8	4.8	yes	up
12	Op06g01438	1.811911045	2.39E-55	4.37E-51	44.4	13	yes	up
12	Op02g01804	1.772976779	1.07E-116	1.95E-112	85.3	28.3	yes	up
12	Op01g02227	1.748016084	4.15E-137	7.60E-133	49.8	7.5	yes	up
12	Op02g01461	1.746249384	1.85E-103	3.39E-99	45.2	7.7	yes	up
12	Op10g00576	1.743752663	2.23E-134	4.09E-130	40.1	4.9	yes	up
12	Op07g00857	1.742674993	1.01E-121	1.85E-117	58.1	11.1	yes	up
12	Op08g01616	1.735403388	2.46E-143	4.51E-139	43.7	5.4	yes	up
12	Op05g01934	1.733942455	6.25E-115	1.14E-110	87.8	30.6	yes	up
12	Op04g01423	1.716015663	7.51E-80	1.37E-75	46.6	10.7	yes	up
12	Op02g00290	1.670126291	1.54E-115	2.83E-111	52.7	9.5	yes	up
12	Op03g00654	1.655251534	5.61E-130	1.03E-125	59.9	10.8	yes	up
12	Op08g01173	1.654874524	9.77E-97	1.79E-92	77.4	25.6	yes	up
12	Op01g01728	1.632180487	6.24E-113	1.14E-108	63.4	13.7	yes	up
12	Op01g00652	1.632004281	1.57E-53	2.87E-49	48.4	15.9	yes	up
12	Op05g01586	1.620836267	8.98E-149	1.64E-144	38.4	4	yes	up
12	Op05g01261	1.618403999	3.08E-83	5.64E-79	29.7	4.3	yes	up
12	Op06g00176	1.570824306	6.06E-95	1.11E-90	32.3	4.4	yes	up
12	Op02g01662	1.562952108	1.01E-240	1.85E-236	34.1	1.7	yes	up
12	Op06g00231	1.560271957	2.41E-94	4.42E-90	76.3	24.5	yes	up
12	Op01g01726	1.52663437	3.38E-76	6.18E-72	44.8	10	yes	up
12	Op02g00382	1.475975575	2.05E-137	3.76E-133	43.4	5.5	yes	up
12	Op06g00087	1.467081515	3.14E-111	5.75E-107	59.5	12.1	yes	up
12	Op07g00682	1.467070468	8.90E-99	1.63E-94	64.2	16.1	yes	up
12	Op09g00519	1.454304388	1.09E-72	2.00E-68	95.3	68.9	yes	up
12	Op01g01702	1.437012021	1.61E-92	2.95E-88	58.8	13.7	yes	up
12	Op07g01136	1.430693208	1.21E-71	2.22E-67	45.2	10.9	yes	up
12	Op05g00313	1.417524904	1.07E-94	1.96E-90	45.5	8.4	yes	up
12	Op07g00647	1.415455068	1.14E-85	2.08E-81	24	2.7	yes	up
12	Op08g00163	1.410203984	7.90E-89	1.45E-84	19.7	1.7	yes	up
12	Op08g01716	1.40776387	3.11E-35	5.69E-31	77.8	50.4	yes	up
12	Op08g01421	1.403265323	2.33E-82	4.27E-78	15.1	1	yes	up
12	Op05g00334	1.398468016	6.72E-75	1.23E-70	52.7	13.8	yes	up
12	Op07g01425	1.393212412	1.99E-55	3.65E-51	53.8	18.1	yes	up
12	Op03g02059	1.392722663	2.21E-74	4.04E-70	90.3	53.1	yes	up
12	Op09g00032	1.389363927	1.22E-83	2.23E-79	99.3	78.9	yes	up
12	Op10g00728	1.38374161	1.40E-97	2.56E-93	62	14.4	yes	up
12	Op09g01072	1.38020982	2.83E-63	5.18E-59	31.5	5.9	yes	up
12	Op01g02338	1.354839114	4.08E-64	7.46E-60	49.5	13.6	yes	up
12	Op02g01491	1.342788613	8.31E-103	1.52E-98	37.3	5.3	yes	up
12	Op02g02262	1.334470827	8.33E-53	1.53E-48	53	18.6	yes	up
12	Op04g00252	1.327472296	1.13E-43	2.07E-39	58.4	24.2	yes	up
12	Op04g01268	1.326321148	1.28E-123	2.35E-119	21.9	1.4	yes	up

12	Op03g00742	1.32543706	2.01E-72	3.68E-68	32.3	5.5	yes	up
12	Op09g01582	1.324386327	9.84E-60	1.80E-55	35.8	8.1	yes	up
12	Op10g00229	1.307929184	9.99E-77	1.83E-72	40.5	8	yes	up
12	Op07g01326	1.305430403	2.80E-86	5.12E-82	99.3	75.3	yes	up
12	Op11g01053	1.304183144	2.43E-77	4.45E-73	44.8	9.9	yes	up
12	Op11g00447	1.287612101	1.85E-78	3.39E-74	53	12.7	yes	up
12	Op01g00163	1.284238751	3.65E-94	6.67E-90	36.6	5.5	yes	up
12	Op03g00176	1.282355784	3.24E-82	5.93E-78	56.3	13.5	yes	up
12	Op11g00930	1.277720993	1.16E-56	2.13E-52	53.4	17.4	yes	up
12	Op04g00144	1.27072575	3.80E-41	6.96E-37	17.6	2.9	yes	up
12	Op04g01209	1.264862126	1.55E-57	2.84E-53	33	7.1	yes	up
12	Op03g00368	1.263246895	1.84E-182	3.38E-178	26.9	1.4	yes	up
12	Op07g00549	1.260423139	1.69E-36	3.09E-32	31.5	9.2	yes	up
12	Op03g00063	1.236638907	5.66E-77	1.04E-72	42.7	8.7	yes	up
12	Op07g01059	1.234819707	3.54E-49	6.49E-45	69.2	32	yes	up
12	Op03g00677	1.230833169	6.69E-64	1.23E-59	42.7	10.4	yes	up
12	Op09g00972	1.228937498	5.85E-82	1.07E-77	45.2	9.1	yes	up
12	Op01g01723	1.223919072	7.41E-97	1.36E-92	33.3	4.5	yes	up
12	Op09g01119	1.220777359	1.25E-47	2.29E-43	48.4	15.3	yes	up
12	Op09g00554	1.209713393	1.91E-77	3.49E-73	25.4	3.3	yes	up
12	Op05g01847	1.204633328	9.53E-57	1.75E-52	25.4	4.3	yes	up
12	Op04g01278	1.202033221	1.14E-72	2.09E-68	39.8	8	yes	up
12	Op01g00609	1.196834585	4.48E-59	8.21E-55	32.3	6.6	yes	up
12	Op02g00740	1.19380514	3.43E-42	6.27E-38	65.6	31.7	yes	up
12	Op10g01176	1.191097193	1.97E-57	3.60E-53	66.7	25.4	yes	up
12	Op08g01574	1.186911304	1.81E-37	3.31E-33	72.8	43.5	yes	up
12	Op11g00162	1.183374368	5.56E-37	1.02E-32	58.1	27.3	yes	up
12	Op07g00512	1.180604848	9.45E-50	1.73E-45	77.1	37.5	yes	up
12	Op08g00012	1.173743943	2.41E-69	4.41E-65	39.1	8.1	yes	up
12	Op04g00413	1.154082507	2.75E-77	5.03E-73	28.3	4.2	yes	up
12	Op06g00594	1.152542941	1.09E-92	2.00E-88	25.8	2.9	yes	up
12	Op09g00652	1.128461848	6.44E-34	1.18E-29	35.1	11.6	yes	up
12	Op09g01534	1.128411831	6.60E-52	1.21E-47	39.8	10.4	yes	up
12	Op11g00242	1.128046521	2.03E-65	3.71E-61	59.5	18.1	yes	up
12	Op02g00469	1.127998231	2.08E-45	3.81E-41	23.3	4.5	yes	up
12	Op09g00429	1.120258984	5.30E-59	9.70E-55	93.9	62.2	yes	up
12	Op03g00293	1.114555799	3.86E-90	7.06E-86	27.6	3.4	yes	up
12	Op01g01656	1.11217998	2.03E-47	3.71E-43	26.9	5.6	yes	up
12	Op10g00526	1.109963782	2.02E-54	3.70E-50	35.5	8.2	yes	up
12	Op01g01724	1.109752145	9.17E-64	1.68E-59	25.8	4.1	yes	up
12	Op02g00534	1.109387396	1.36E-57	2.49E-53	45.5	12.1	yes	up
12	Op03g02226	1.108440893	7.82E-33	1.43E-28	23.7	5.8	yes	up
12	Op03g01051	1.09975756	7.41E-63	1.36E-58	29.4	5.2	yes	up
12	Op06g01442	1.09333053	9.32E-71	1.71E-66	67.4	20.9	yes	up
12	Op09g01412	1.089621191	3.58E-91	6.55E-87	29	3.6	yes	up
12	Op01g00054	1.085056564	5.26E-61	9.63E-57	42.3	10	yes	up
12	Op11g00141	1.082845904	3.55E-69	6.49E-65	97.8	78.8	yes	up
12	Op07g01723	1.079903465	9.87E-59	1.81E-54	25.8	4.4	yes	up
12	Op02g01627	1.076409055	2.16E-74	3.95E-70	28	4.1	yes	up
12	Op07g00460	1.068251751	1.88E-24	3.44E-20	34.1	13.7	yes	up
12	Op06g00706	1.06776011	1.11E-31	2.03E-27	30.8	9.6	yes	up
12	Op11g01049	1.061119239	1.73E-37	3.17E-33	50.2	20.7	yes	up
12	Op07g00653	1.06018829	5.47E-69	1.00E-64	32.3	5.6	yes	up
12	Op04g00235	1.057363694	1.18E-56	2.16E-52	97.5	80.2	yes	up
12	Op03g01589	1.056314077	1.56E-64	2.86E-60	24	3.5	yes	up
12	Op02g02152	1.055655642	1.73E-43	3.16E-39	30.5	7.6	yes	up
12	Op09g00296	1.053206083	2.70E-39	4.94E-35	46.6	16.9	yes	up
12	Op04g01040	1.052793927	7.20E-48	1.32E-43	53	18.9	yes	up
12	Op04g00044	1.043329746	1.09E-55	2.00E-51	46.2	13	yes	up
12	Op05g01648	1.042799837	2.63E-54	4.82E-50	24.4	4.2	yes	up
12	Op02g00894	1.04256486	6.65E-28	1.22E-23	29.4	9.7	yes	up
12	Op03g02131	1.038154839	3.12E-27	5.72E-23	53	26.9	yes	up

12	Op07g01360	1.036098009	2.16E-41	3.95E-37	63.8	28.7	yes	up
12	Op06g00227	1.034770106	1.15E-38	2.11E-34	43.4	15.2	yes	up
12	Op08g01255	1.034355191	1.62E-37	2.96E-33	35.8	11.4	yes	up
12	Op06g01435	1.026637279	1.64E-52	3.00E-48	40.5	10.4	yes	up
12	Op03g00475	1.021828386	1.34E-35	2.45E-31	39.1	13.2	yes	up
12	Op03g00579	1.021770865	1.42E-27	2.60E-23	25.8	7.8	yes	up
12	Op07g00407	1.021323554	5.31E-40	9.71E-36	19.4	3.5	yes	up
12	Op04g00216	1.018383771	5.09E-19	9.32E-15	51.3	28.8	yes	up
12	Op11g00476	1.012703406	3.45E-30	6.32E-26	31.9	10.3	yes	up
12	Op07g00217	1.004544424	1.00E-43	1.84E-39	37.6	10.6	yes	up
12	Op01g00887	1.003578674	3.24E-44	5.94E-40	24.4	5	yes	up
12	Op07g00448	1.003457108	6.02E-57	1.10E-52	34.1	7.3	yes	up
12	Op09g01399	1.002758656	5.46E-37	9.99E-33	57.3	25.5	yes	up
13	Op07g00370	4.519624851	1.96E-119	3.60E-115	90.2	38	yes	up
13	Op11g01311	4.31782835	1.79E-96	3.27E-92	29	3.3	yes	up
13	Op02g01503	4.265966548	1.29E-88	2.37E-84	73.5	26.5	yes	up
13	Op05g00302	4.094784873	2.30E-246	4.21E-242	92.7	17.2	yes	up
13	Op10g00171	3.718060152	2.70E-129	4.93E-125	27.8	2.1	yes	up
13	Op04g00133	3.62331428	7.05E-236	1.29E-231	47.3	3.4	yes	up
13	Op05g00993	3.552527969	2.55E-198	4.66E-194	73.9	11.9	yes	up
13	Op07g01854	3.513595161	1.74E-176	3.18E-172	45.3	4.5	yes	up
13	Op03g01293	3.49914581	4.77E-200	8.73E-196	66.5	9.2	yes	up
13	Op03g00664	3.454948329	1.92E-146	3.51E-142	49.8	6.7	yes	up
13	Op07g00253	3.224853393	2.07E-279	3.78E-275	68.6	6.6	yes	up
13	Op07g00377	3.133471129	4.93E-63	9.03E-59	93.5	62.1	yes	up
13	Op07g00380	3.133397613	8.62E-73	1.58E-68	72.2	28.5	yes	up
13	Op07g00374	2.919827945	3.47E-77	6.34E-73	62.9	20.5	yes	up
13	Op09g00413	2.736540886	0	0	66.5	2	yes	up
13	Op02g00425	2.72838867	7.18E-81	1.31E-76	71.8	25.8	yes	up
13	Op09g00371	2.708971646	2.92E-157	5.35E-153	49	5.8	yes	up
13	Op05g01588	2.633178231	3.04E-128	5.56E-124	21.6	1.2	yes	up
13	Op01g01860	2.598259368	6.88E-233	1.26E-228	29.8	1.1	yes	up
13	Op07g01708	2.563192126	6.39E-99	1.17E-94	78	25.7	yes	up
13	Op02g02121	2.54330951	5.30E-183	9.70E-179	82.9	16.6	yes	up
13	Op07g00416	2.432296238	8.64E-45	1.58E-40	63.3	29	yes	up
13	Op10g01120	2.265441947	3.43E-273	6.28E-269	32.2	1.1	yes	up
13	Op11g00506	2.258879319	3.49E-90	6.39E-86	53.9	12.4	yes	up
13	Op10g00369	2.143556385	0	0	50.2	1.8	yes	up
13	Op07g00540	2.128506585	4.01E-248	7.33E-244	64.1	6.4	yes	up
13	Op02g01555	2.102648452	1.69E-153	3.09E-149	60	8.8	yes	up
13	Op01g00297	2.099449673	1.00E-236	1.84E-232	43.3	2.8	yes	up
13	Op04g01545	2.046925703	0	0	55.5	3.3	yes	up
13	Op11g00135	2.019924107	5.52E-168	1.01E-163	20	0.7	yes	up
13	Op01g01575	1.968881532	2.55E-200	4.66E-196	23.3	0.7	yes	up
13	Op10g00045	1.956169394	3.60E-163	6.58E-159	20	0.7	yes	up
13	Op10g00372	1.953924947	9.82E-61	1.80E-56	80	37.9	yes	up
13	Op09g01492	1.953741448	0	0	60	4	yes	up
13	Op05g00873	1.910851103	1.79E-106	3.28E-102	75.9	22.1	yes	up
13	Op05g01494	1.895081761	6.89E-149	1.26E-144	23.3	1.2	yes	up
13	Op10g00402	1.881935191	9.58E-60	1.75E-55	49.8	14	yes	up
13	Op03g00733	1.878037524	5.71E-250	1.05E-245	37.1	1.8	yes	up
13	Op07g00486	1.842252742	0	0	41.6	1	yes	up
13	Op07g00610	1.827280042	5.46E-115	1.00E-110	66.9	15.1	yes	up
13	Op05g00874	1.816548671	5.58E-220	1.02E-215	44.1	3.1	yes	up
13	Op07g01106	1.762794345	0	0	40.4	1.2	yes	up
13	Op02g00705	1.707117061	6.91E-122	1.27E-117	16.3	0.7	yes	up
13	Op04g01396	1.685841631	1.71E-132	3.13E-128	40.4	4.6	yes	up
13	Op01g02135	1.672145819	0	0	37.1	1	yes	up
13	Op04g00362	1.658478213	4.39E-302	8.04E-298	35.5	1.2	yes	up
13	Op08g01481	1.643482328	1.41E-139	2.57E-135	19.2	0.8	yes	up
13	Op11g00433	1.615878743	3.51E-215	6.42E-211	45.7	3.4	yes	up
13	Op01g00071	1.613776642	1.47E-204	2.68E-200	42	3	yes	up

13	Op02g00973	1.593007483	8.47E-142	1.55E-137	18.4	0.7	yes	up
13	Op03g00514	1.569614161	1.64E-73	3.01E-69	48.2	11.1	yes	up
13	Op07g01795	1.526194146	1.17E-182	2.14E-178	19.2	0.5	yes	up
13	Op03g01903	1.525824823	4.36E-141	7.97E-137	35.5	3.2	yes	up
13	Op03g01875	1.5223381	8.93E-238	1.63E-233	36.7	1.8	yes	up
13	Op03g01647	1.511247793	0	0	32.2	0.7	yes	up
13	Op11g00603	1.509559848	0	0	42	1.5	yes	up
13	Op05g00954	1.486645745	0	0	29	0.6	yes	up
13	Op10g00216	1.480499214	0	0	25.7	0.3	yes	up
13	Op09g01205	1.468170817	0	0	35.5	0.8	yes	up
13	Op11g00001	1.466692947	8.18E-68	1.50E-63	43.3	9.4	yes	up
13	Op07g01735	1.435847396	1.08E-130	1.97E-126	55.5	8.8	yes	up
13	Op-scaf01g0010	1.424107649	0	0	41.2	1.6	yes	up
13	Op06g00147	1.412172197	4.31E-125	7.89E-121	15.9	0.6	yes	up
13	Op04g01238	1.401100326	1.51E-98	2.76E-94	10.2	0.3	yes	up
13	Op08g01583	1.398430171	4.68E-80	8.57E-76	40	6.8	yes	up
13	Op06g00751	1.384666605	1.07E-135	1.95E-131	24.1	1.4	yes	up
13	Op04g00452	1.368217557	1.27E-104	2.33E-100	15.5	0.7	yes	up
13	Op06g00285	1.367367284	1.09E-202	1.99E-198	20.8	0.5	yes	up
13	Op02g01880	1.355974202	3.48E-285	6.36E-281	26.5	0.6	yes	up
13	Op05g01562	1.348595644	1.46E-131	2.67E-127	15.9	0.5	yes	up
13	Op01g00078	1.335120221	1.40E-56	2.57E-52	15.1	1.5	yes	up
13	Op05g00053	1.329558288	6.15E-164	1.13E-159	23.7	1.1	yes	up
13	Op10g01185	1.326670115	3.70E-157	6.76E-153	14.7	0.3	yes	up
13	Op02g02071	1.317851023	0	0	35.5	0.5	yes	up
13	Op03g00186	1.305924893	5.24E-210	9.60E-206	20.4	0.5	yes	up
13	Op09g00035	1.298947768	3.26E-109	5.97E-105	18.8	1.1	yes	up
13	Op07g00361	1.290017097	1.87E-65	3.42E-61	39.6	8.1	yes	up
13	Op05g00409	1.278375109	8.25E-39	1.51E-34	77.1	41.5	yes	up
13	Op07g01509	1.268201516	4.65E-111	8.51E-107	20.4	1.3	yes	up
13	Op01g02546	1.268193326	0	0	34.3	0.7	yes	up
13	Op10g00108	1.259979925	4.28E-32	7.84E-28	13.5	2	yes	up
13	Op07g01876	1.250514829	7.78E-115	1.42E-110	33.9	3.6	yes	up
13	Op05g00317	1.234448355	0	0	32.2	0.6	yes	up
13	Op05g01294	1.233013009	1.32E-135	2.41E-131	35.1	3.2	yes	up
13	Op01g01426	1.21948328	3.03E-105	5.54E-101	18.8	1.1	yes	up
13	Op07g01333	1.211418683	2.27E-167	4.16E-163	20	0.7	yes	up
13	Op06g00792	1.201295462	4.09E-83	7.48E-79	37.1	5.9	yes	up
13	Op07g01623	1.200730612	4.72E-113	8.64E-109	15.5	0.6	yes	up
13	Op08g00979	1.198645015	7.51E-103	1.37E-98	28.2	2.8	yes	up
13	Op04g00017	1.194843574	1.07E-220	1.96E-216	36.3	2	yes	up
13	Op03g01260	1.191386975	5.04E-139	9.23E-135	14.3	0.4	yes	up
13	Op02g00240	1.188491237	4.14E-31	7.57E-27	59.2	27.7	yes	up
13	Op11g00601	1.184237529	2.03E-128	3.72E-124	18	0.8	yes	up
13	Op07g01978	1.181801429	8.58E-160	1.57E-155	14.7	0.3	yes	up
13	Op08g00277	1.166804173	1.98E-231	3.62E-227	22.4	0.5	yes	up
13	Op06g00890	1.164743977	9.78E-64	1.79E-59	39.6	8.4	yes	up
13	Op01g01848	1.163788613	6.98E-37	1.28E-32	45.7	15.5	yes	up
13	Op01g01482	1.147912094	8.94E-178	1.64E-173	14.3	0.2	yes	up
13	Op01g00652	1.140937693	1.42E-17	2.60E-13	37.1	16.3	yes	up
13	Op08g01121	1.13084854	2.48E-223	4.54E-219	18.8	0.3	yes	up
13	Op03g00706	1.127818084	1.37E-52	2.52E-48	16.7	1.9	yes	up
13	Op07g00420	1.125034096	1.08E-128	1.97E-124	21.6	1.2	yes	up
13	Op03g01671	1.123873066	2.11E-285	3.86E-281	30.2	0.8	yes	up
13	Op02g01353	1.122825645	7.69E-186	1.41E-181	13.9	0.2	yes	up
13	Op01g02347	1.11392114	2.20E-73	4.03E-69	43.3	8.5	yes	up
13	Op04g00576	1.099901846	1.17E-38	2.15E-34	20	3.6	yes	up
13	Op03g01716	1.098525934	1.75E-60	3.20E-56	34.3	6.5	yes	up
13	Op09g00038	1.090606651	6.01E-142	1.10E-137	16.3	0.5	yes	up
13	Op08g01579	1.083546859	3.35E-56	6.14E-52	16.3	1.7	yes	up
13	Op05g00760	1.079411773	1.19E-39	2.17E-35	87.3	53.9	yes	up
13	Op04g01285	1.073692195	4.04E-282	7.40E-278	25.7	0.5	yes	up

13	Op11g01183	1.072950231	3.02E-95	5.54E-91	23.7	2.1	yes	up
13	Op03g02041	1.065994683	3.93E-231	7.19E-227	24.9	0.7	yes	up
13	Op05g01610	1.064553827	1.25E-21	2.29E-17	47.8	22.4	yes	up
13	Op05g00625	1.044595021	1.63E-276	2.98E-272	27.3	0.6	yes	up
13	Op01g00728	1.038890583	5.07E-95	9.29E-91	33.1	4.1	yes	up
13	Op06g00097	1.038466707	1.73E-78	3.17E-74	40	7.1	yes	up
13	Op01g02015	1.031160148	0	0	28.2	0.5	yes	up
13	Op07g00437	1.024270339	3.08E-243	5.63E-239	26.5	0.8	yes	up
13	Op02g02332	1.01779646	2.22E-40	4.07E-36	67.8	30	yes	up
13	Op01g01577	1.017503064	2.21E-112	4.04E-108	27.3	2.4	yes	up
13	Op06g00207	1.01669468	5.98E-40	1.10E-35	19.2	3.2	yes	up
13	Op05g00398	1.007602509	6.16E-38	1.13E-33	14.3	1.9	yes	up
13	Op08g01069	1.006411179	1.22E-158	2.24E-154	19.6	0.7	yes	up
13	Op01g00620	1.00557674	2.09E-231	3.83E-227	26.9	0.9	yes	up
13	Op07g02062	1.005216549	1.12E-164	2.06E-160	22	0.9	yes	up
13	Op02g00958	1.001298567	8.21E-42	1.50E-37	17.1	2.5	yes	up
14	Op01g00917	3.451967086	1.54E-166	2.81E-162	64.4	9.6	yes	up
14	Op11g00903	3.324119922	3.02E-23	5.54E-19	19.4	4.9	yes	up
14	Op09g01574	3.124378848	6.72E-130	1.23E-125	91	30.8	yes	up
14	Op11g01253	3.067838657	1.41E-165	2.58E-161	55.4	6.8	yes	up
14	Op07g01742	3.015334993	6.50E-247	1.19E-242	50.9	3.5	yes	up
14	Op02g02189	2.982070124	4.02E-270	7.36E-266	61.3	4.9	yes	up
14	Op04g01386	2.952511505	1.41E-45	2.57E-41	29.7	6.2	yes	up
14	Op02g02188	2.798264685	3.69E-105	6.76E-101	54.1	10	yes	up
14	Op09g00028	2.787722224	3.49E-171	6.40E-167	68	10.3	yes	up
14	Op09g00878	2.754870032	1.64E-250	3.00E-246	66.7	6.4	yes	up
14	Op03g01880	2.749251747	5.71E-107	1.05E-102	39.6	5.1	yes	up
14	Op05g01310	2.6783812	9.12E-144	1.67E-139	59	9	yes	up
14	Op03g02310	2.656996463	1.03E-156	1.89E-152	66.7	10.2	yes	up
14	Op06g01247	2.645747161	3.01E-156	5.51E-152	41.4	3.8	yes	up
14	Op03g01775	2.616899271	2.80E-121	5.13E-117	56.3	9.7	yes	up
14	Op09g01507	2.603532123	1.74E-93	3.18E-89	77.5	26.6	yes	up
14	Op05g01441	2.599958796	2.09E-136	3.82E-132	49.1	6.4	yes	up
14	Op02g02186	2.590508719	6.30E-113	1.15E-108	69.4	16.4	yes	up
14	Op02g01124	2.562236598	0	0	47.3	1.9	yes	up
14	Op04g00199	2.551561095	3.96E-237	7.25E-233	50.9	3.7	yes	up
14	Op05g00906	2.525343886	4.31E-102	7.89E-98	63.5	14.9	yes	up
14	Op05g00721	2.51597545	3.57E-118	6.53E-114	44.1	5.7	yes	up
14	Op06g01567	2.487111776	1.31E-159	2.40E-155	58.6	7.8	yes	up
14	Op04g01580	2.475469696	2.15E-156	3.93E-152	51.4	6	yes	up
14	Op11g00416	2.463458878	2.85E-85	5.21E-81	62.6	16.8	yes	up
14	Op11g00904	2.458206519	8.74E-107	1.60E-102	39.6	5	yes	up
14	Op03g00132	2.384932755	3.32E-79	6.07E-75	61.7	16.8	yes	up
14	Op05g01668	2.357253024	6.83E-119	1.25E-114	57.7	10.1	yes	up
14	Op08g00917	2.34974535	4.02E-166	7.36E-162	39.2	3.1	yes	up
14	Op01g00960	2.337366701	1.29E-97	2.37E-93	19.8	1.3	yes	up
14	Op02g00289	2.303946997	3.69E-223	6.75E-219	36.9	1.9	yes	up
14	Op04g00143	2.290224117	6.54E-61	1.20E-56	20.7	2.4	yes	up
14	Op03g00480	2.275020747	1.12E-98	2.04E-94	52.7	10.3	yes	up
14	Op07g01151	2.263085823	1.76E-129	3.23E-125	41	4.5	yes	up
14	Op05g00799	2.25243605	8.37E-90	1.53E-85	85.6	35.6	yes	up
14	Op08g01484	2.249776577	7.26E-98	1.33E-93	79.3	23	yes	up
14	Op01g02234	2.248929129	1.45E-82	2.65E-78	49.1	10	yes	up
14	Op02g02190	2.248607157	1.93E-188	3.53E-184	40.5	2.9	yes	up
14	Op09g00641	2.239615698	1.54E-185	2.81E-181	49.1	4.5	yes	up
14	Op10g01146	2.225726743	2.00E-229	3.67E-225	37.8	1.9	yes	up
14	Op05g00924	2.219817677	3.10E-86	5.67E-82	92.3	48.8	yes	up
14	Op05g01862	2.185913585	1.06E-89	1.95E-85	46.8	8.5	yes	up
14	Op02g00446	2.179114941	1.35E-99	2.48E-95	52.3	9.5	yes	up
14	Op05g01507	2.166696565	4.10E-104	7.50E-100	42.3	6	yes	up
14	Op05g00326	2.152914275	5.50E-79	1.01E-74	88.7	41.2	yes	up
14	Op07g01382	2.152541717	1.19E-148	2.18E-144	36.5	3	yes	up

14	Op01g00694	2.138951077	2.34E-68	4.29E-64	62.2	19.6	yes	up
14	Op07g00406	2.134691476	1.85E-79	3.38E-75	62.6	18.1	yes	up
14	Op01g01318	2.117329759	6.71E-50	1.23E-45	62.6	24.4	yes	up
14	Op01g01135	2.112126187	7.47E-158	1.37E-153	29.7	1.8	yes	up
14	Op08g01129	2.112080526	5.14E-95	9.40E-91	40.1	5.8	yes	up
14	Op05g01671	2.105981652	1.90E-202	3.47E-198	32.4	1.6	yes	up
14	Op03g00616	2.102862414	2.33E-82	4.26E-78	77	27.3	yes	up
14	Op08g01726	2.062429915	6.34E-70	1.16E-65	55.9	15	yes	up
14	Op09g00448	2.056088211	7.67E-66	1.40E-61	86	45.6	yes	up
14	Op03g00242	2.05479107	8.92E-34	1.63E-29	60.4	30.3	yes	up
14	Op10g01143	2.05130708	1.50E-229	2.74E-225	19.8	0.3	yes	up
14	Op04g01579	2.049948982	5.00E-155	9.15E-151	38.3	3.2	yes	up
14	Op03g01943	2.026732072	2.86E-150	5.23E-146	17.6	0.5	yes	up
14	Op01g00750	2.024627646	7.51E-50	1.37E-45	82.9	48.5	yes	up
14	Op01g00920	2.016806227	1.09E-192	2.00E-188	23.4	0.7	yes	up
14	Op06g00340	2.013812469	2.98E-97	5.46E-93	43.7	6.7	yes	up
14	Op04g00193	1.996191148	5.84E-84	1.07E-79	43.7	7.8	yes	up
14	Op03g00587	1.993787216	7.65E-80	1.40E-75	35.1	5.3	yes	up
14	Op02g00840	1.988073512	8.91E-251	1.63E-246	38.3	1.7	yes	up
14	Op05g00264	1.986563678	7.38E-100	1.35E-95	47.3	7.7	yes	up
14	Op01g00199	1.955541553	3.08E-166	5.65E-162	23	0.9	yes	up
14	Op03g02251	1.953799886	2.17E-46	3.97E-42	56.3	20.6	yes	up
14	Op10g00703	1.953132618	9.36E-14	1.71E-09	15.8	4.9	yes	up
14	Op01g01136	1.939900512	1.06E-211	1.93E-207	30.2	1.2	yes	up
14	Op02g01075	1.936891231	5.31E-66	9.71E-62	32	5.2	yes	up
14	Op05g01012	1.899432354	1.63E-121	2.98E-117	23	1.4	yes	up
14	Op01g01702	1.88870036	1.20E-44	2.19E-40	47.3	14.3	yes	up
14	Op06g00645	1.881043462	1.08E-32	1.98E-28	44.1	16.7	yes	up
14	Op03g00115	1.873234416	1.56E-73	2.85E-69	50.5	11.6	yes	up
14	Op07g01462	1.86866888	2.06E-73	3.77E-69	50.9	11.7	yes	up
14	Op02g01458	1.866378892	2.29E-184	4.19E-180	24.3	0.9	yes	up
14	Op06g01084	1.855800188	3.60E-74	6.59E-70	47.3	9.9	yes	up
14	Op06g01490	1.850229496	3.03E-40	5.54E-36	40.5	12.2	yes	up
14	Op01g00677	1.848130865	2.76E-57	5.05E-53	84.7	50.2	yes	up
14	Op04g00781	1.831151753	7.93E-123	1.45E-118	31.5	2.7	yes	up
14	Op11g00192	1.825879413	5.92E-67	1.08E-62	40.1	8	yes	up
14	Op09g01529	1.805153143	5.37E-58	9.84E-54	53.6	15.7	yes	up
14	Op08g00200	1.804006863	5.70E-62	1.04E-57	47.7	12.1	yes	up
14	Op01g02314	1.802657565	5.38E-31	9.85E-27	62.6	32.4	yes	up
14	Op07g01268	1.802065319	2.32E-109	4.24E-105	31.1	3	yes	up
14	Op09g01209	1.792709009	3.61E-44	6.61E-40	34.2	8.3	yes	up
14	Op11g00478	1.791366745	1.44E-91	2.63E-87	17.6	1.1	yes	up
14	Op04g00504	1.786972507	8.33E-32	1.52E-27	35.1	11.1	yes	up
14	Op08g00098	1.777586742	1.88E-59	3.43E-55	40.5	9.1	yes	up
14	Op01g00766	1.777396745	3.87E-41	7.08E-37	64.4	28.8	yes	up
14	Op09g00305	1.776645998	4.48E-62	8.19E-58	16.7	1.5	yes	up
14	Op11g00479	1.77117483	3.88E-73	7.09E-69	14	0.8	yes	up
14	Op08g01785	1.768090383	4.38E-109	8.03E-105	37.8	4.5	yes	up
14	Op07g01709	1.767556096	3.52E-59	6.44E-55	43.2	10.5	yes	up
14	Op01g00668	1.766833443	8.95E-184	1.64E-179	27	1.1	yes	up
14	Op03g00831	1.766048056	7.68E-131	1.41E-126	21.6	1.1	yes	up
14	Op06g00341	1.763285095	2.13E-98	3.90E-94	21.6	1.6	yes	up
14	Op03g00586	1.762226829	1.30E-99	2.38E-95	31.5	3.4	yes	up
14	Op01g00773	1.750186725	6.96E-20	1.27E-15	49.1	26.3	yes	up
14	Op11g00194	1.745874802	5.27E-49	9.65E-45	59.9	20.9	yes	up
14	Op03g01979	1.7442006	4.54E-37	8.31E-33	15.3	2.1	yes	up
14	Op06g00383	1.740626354	1.61E-74	2.94E-70	50	10.8	yes	up
14	Op10g01144	1.739721561	1.92E-210	3.51E-206	15.3	0.2	yes	up
14	Op02g00891	1.734066862	3.96E-49	7.26E-45	52.7	17.5	yes	up
14	Op02g01829	1.731036626	3.13E-86	5.73E-82	27.9	3.1	yes	up
14	Op11g00362	1.725715173	3.58E-47	6.55E-43	38.7	9.8	yes	up
14	Op04g01575	1.714376013	3.06E-36	5.60E-32	23.9	5	yes	up

14	Op02g00504	1.710200205	4.57E-69	8.36E-65	24.8	3	yes	up
14	Op07g00175	1.705957825	1.01E-46	1.84E-42	57.7	21.6	yes	up
14	Op04g00125	1.700807393	2.47E-53	4.52E-49	59.9	20.9	yes	up
14	Op01g00850	1.699637167	6.21E-51	1.14E-46	70.7	31.7	yes	up
14	Op03g01384	1.694034562	2.68E-166	4.90E-162	20.3	0.6	yes	up
14	Op09g01134	1.692244312	1.83E-86	3.35E-82	11.3	0.4	yes	up
14	Op08g00935	1.689800317	1.20E-56	2.19E-52	32.9	6.2	yes	up
14	Op07g01023	1.683937614	6.67E-182	1.22E-177	14.9	0.2	yes	up
14	Op04g00231	1.675628592	9.35E-50	1.71E-45	60.4	22	yes	up
14	Op01g01718	1.670060997	9.89E-29	1.81E-24	26.1	7	yes	up
14	Op09g00344	1.659248026	2.40E-41	4.39E-37	55.4	21.4	yes	up
14	Op09g00696	1.657762307	4.50E-129	8.24E-125	11.3	0.2	yes	up
14	Op02g00795	1.651252082	2.42E-79	4.44E-75	26.6	3	yes	up
14	Op05g00786	1.65051074	1.06E-17	1.95E-13	27.9	10.5	yes	up
14	Op01g00068	1.648836997	2.08E-57	3.81E-53	62.2	20.8	yes	up
14	Op10g00439	1.647671318	3.62E-36	6.62E-32	69.8	37.8	yes	up
14	Op11g00584	1.632313545	2.16E-40	3.95E-36	58.6	23.9	yes	up
14	Op11g00504	1.628450847	1.16E-33	2.12E-29	47.3	17.9	yes	up
14	Op06g01541	1.624430572	9.35E-168	1.71E-163	24.8	1.1	yes	up
14	Op09g00628	1.623231267	1.34E-87	2.46E-83	35.1	4.7	yes	up
14	Op02g00380	1.621677919	6.12E-58	1.12E-53	34.7	6.8	yes	up
14	Op09g00738	1.612995569	4.66E-68	8.53E-64	36	6.2	yes	up
14	Op07g01874	1.607597757	3.71E-24	6.79E-20	29.7	9.6	yes	up
14	Op10g01473	1.603919143	4.40E-116	8.05E-112	22.5	1.4	yes	up
14	Op08g01596	1.59905951	1.71E-38	3.13E-34	59	24	yes	up
14	Op07g01195	1.598602978	5.92E-50	1.08E-45	51.8	16.1	yes	up
14	Op09g01072	1.589999261	5.94E-45	1.09E-40	29.7	6.1	yes	up
14	Op01g00213	1.589266702	2.63E-49	4.81E-45	22.5	3.4	yes	up
14	Op09g00091	1.58887079	7.89E-136	1.44E-131	38.7	3.7	yes	up
14	Op03g01421	1.585187264	4.94E-65	9.05E-61	39.6	7.6	yes	up
14	Op07g00888	1.583399242	1.54E-35	2.82E-31	55	24	yes	up
14	Op01g02379	1.583191506	1.11E-44	2.04E-40	48.6	15.8	yes	up
14	Op04g01647	1.580573923	1.18E-61	2.16E-57	54.1	14.8	yes	up
14	Op09g01313	1.574657872	6.91E-72	1.27E-67	32.4	4.9	yes	up
14	Op03g02217	1.572298274	9.38E-45	1.72E-40	73.4	34.1	yes	up
14	Op03g01197	1.56978423	1.71E-68	3.14E-64	47.7	10.2	yes	up
14	Op04g00233	1.568676353	8.27E-96	1.51E-91	22.5	1.7	yes	up
14	Op08g01786	1.563613492	1.55E-104	2.83E-100	28.4	2.6	yes	up
14	Op06g00339	1.557769549	4.27E-35	7.82E-31	42.3	14.2	yes	up
14	Op02g02187	1.556171936	1.83E-46	3.36E-42	77.9	41.5	yes	up
14	Op07g00487	1.533499287	4.98E-49	9.12E-45	25.7	4.4	yes	up
14	Op01g01522	1.53211971	5.22E-114	9.55E-110	15.3	0.6	yes	up
14	Op03g02165	1.522582651	5.04E-84	9.22E-80	27	2.9	yes	up
14	Op01g02321	1.513851382	3.02E-23	5.53E-19	17.6	4	yes	up
14	Op02g00188	1.512803433	3.43E-42	6.29E-38	40.5	11.4	yes	up
14	Op10g00479	1.510398492	6.20E-72	1.13E-67	33.3	5.1	yes	up
14	Op07g00359	1.507331156	2.00E-42	3.65E-38	83.8	53.2	yes	up
14	Op04g01029	1.506748525	1.77E-39	3.23E-35	57.2	22.9	yes	up
14	Op10g01352	1.503214844	5.47E-30	1.00E-25	22.1	4.9	yes	up
14	Op04g00086	1.499344078	6.84E-41	1.25E-36	79.3	48.1	yes	up
14	Op07g01948	1.491757196	1.01E-40	1.85E-36	76.1	42.4	yes	up
14	Op06g00722	1.490573058	9.23E-22	1.69E-17	23	6.8	yes	up
14	Op08g01295	1.47737392	6.92E-36	1.27E-31	24.3	5.1	yes	up
14	Op09g00941	1.471931247	1.47E-93	2.69E-89	18.5	1.2	yes	up
14	Op10g00932	1.466639489	3.42E-56	6.27E-52	22.5	3	yes	up
14	Op02g00839	1.458847099	7.95E-103	1.46E-98	18.5	1	yes	up
14	Op11g01256	1.451655944	2.44E-39	4.46E-35	26.6	5.6	yes	up
14	Op08g00322	1.448444041	3.98E-50	7.28E-46	27.9	5	yes	up
14	Op07g00725	1.447552738	1.91E-28	3.49E-24	18	3.6	yes	up
14	Op05g01917	1.441253344	6.25E-33	1.14E-28	66.2	32.7	yes	up
14	Op08g00508	1.433262101	2.61E-42	4.79E-38	43.7	12.9	yes	up
14	Op03g00747	1.432576087	4.32E-49	7.91E-45	18.5	2.3	yes	up

14	Op03g02151	1.422112047	1.74E-39	3.19E-35	49.1	17.3	yes	up
14	Op02g01529	1.419598589	9.07E-13	1.66E-08	34.2	17.8	yes	up
14	Op01g00049	1.418498455	7.47E-49	1.37E-44	43.2	11.7	yes	up
14	Op10g00392	1.406948902	1.97E-47	3.60E-43	89.6	63	yes	up
14	Op03g01306	1.401365426	2.84E-27	5.21E-23	53.2	25.9	yes	up
14	Op02g02227	1.401185883	8.78E-51	1.61E-46	34.2	7.3	yes	up
14	Op06g01396	1.397498419	5.00E-36	9.15E-32	62.6	29.7	yes	up
14	Op03g00594	1.396774621	1.45E-67	2.66E-63	13.5	0.9	yes	up
14	Op02g02070	1.396084793	2.30E-42	4.21E-38	26.6	5.3	yes	up
14	Op07g01186	1.388826299	8.20E-41	1.50E-36	91.4	76.2	yes	up
14	Op06g00039	1.382744311	7.88E-42	1.44E-37	24.3	4.5	yes	up
14	Op02g02244	1.381846019	3.64E-41	6.66E-37	72.1	35.4	yes	up
14	Op07g00485	1.380327106	1.01E-64	1.86E-60	18	1.7	yes	up
14	Op01g02219	1.377469458	1.00E-61	1.83E-57	31.1	5.1	yes	up
14	Op01g00989	1.376129147	1.80E-37	3.29E-33	32.9	8.4	yes	up
14	Op09g01568	1.375490808	2.11E-26	3.86E-22	31.1	9.8	yes	up
14	Op09g01326	1.374785051	7.14E-43	1.31E-38	59.9	22.5	yes	up
14	Op03g00749	1.373930916	1.05E-27	1.93E-23	15.3	2.7	yes	up
14	Op10g01316	1.369655206	4.45E-47	8.15E-43	12.6	1.1	yes	up
14	Op01g00109	1.367672996	1.13E-74	2.06E-70	26.6	3.1	yes	up
14	Op02g00412	1.366261616	5.41E-49	9.91E-45	39.6	9.8	yes	up
14	Op09g01575	1.363578777	4.89E-17	8.96E-13	58.6	37.3	yes	up
14	Op05g01954	1.361713723	7.29E-30	1.33E-25	55	24.8	yes	up
14	Op01g00406	1.361487648	2.30E-88	4.21E-84	23.4	2.1	yes	up
14	Op10g01447	1.357472589	3.67E-26	6.72E-22	64.9	38	yes	up
14	Op02g00700	1.354991606	6.42E-29	1.18E-24	45.9	17.4	yes	up
14	Op11g00206	1.353761679	2.11E-26	3.87E-22	77	62.8	yes	up
14	Op07g00183	1.349568575	1.22E-51	2.24E-47	25.2	4	yes	up
14	Op02g02321	1.349306658	9.44E-42	1.73E-37	91	76.2	yes	up
14	Op04g00029	1.349109656	2.10E-23	3.84E-19	27	8.4	yes	up
14	Op09g00271	1.345434001	2.88E-30	5.27E-26	31.1	9	yes	up
14	Op07g00299	1.344818679	2.25E-40	4.12E-36	80.6	51	yes	up
14	Op09g00135	1.340867726	2.79E-25	5.12E-21	39.6	15.8	yes	up
14	Op09g00812	1.34076158	1.18E-42	2.16E-38	51.4	17.3	yes	up
14	Op06g00658	1.334623067	1.83E-52	3.36E-48	23.4	3.5	yes	up
14	Op08g01349	1.325890517	1.78E-38	3.26E-34	46.4	15.2	yes	up
14	Op03g00654	1.325279633	1.12E-54	2.05E-50	46.4	11.4	yes	up
14	Op08g01332	1.3214967	4.45E-61	8.14E-57	18.5	1.9	yes	up
14	Op10g01133	1.312374941	8.79E-63	1.61E-58	18.5	1.8	yes	up
14	Op11g00285	1.302380983	5.88E-30	1.08E-25	36.5	11.8	yes	up
14	Op10g01405	1.294012353	8.27E-30	1.51E-25	38.7	13.5	yes	up
14	Op04g01546	1.293821979	3.43E-46	6.29E-42	37.8	9.2	yes	up
14	Op06g00836	1.285186324	3.82E-23	6.99E-19	20.3	5.1	yes	up
14	Op02g00983	1.283572642	3.42E-83	6.25E-79	16.7	1.1	yes	up
14	Op04g00649	1.282971321	3.55E-26	6.50E-22	49.5	22.9	yes	up
14	Op02g01921	1.282261043	4.25E-27	7.79E-23	56.8	29	yes	up
14	Op02g02245	1.279202499	2.82E-52	5.16E-48	31.5	6.1	yes	up
14	Op06g01542	1.277737204	8.59E-152	1.57E-147	13.1	0.2	yes	up
14	Op05g01802	1.274590859	1.48E-35	2.71E-31	34.2	9.5	yes	up
14	Op11g01390	1.273969879	2.40E-19	4.39E-15	32.4	12.8	yes	up
14	Op03g00176	1.266581573	1.02E-30	1.88E-26	41.4	14.1	yes	up
14	Op11g00927	1.265852689	5.90E-21	1.08E-16	26.1	8.5	yes	up
14	Op10g01095	1.265441981	2.32E-63	4.25E-59	19.4	2	yes	up
14	Op01g00212	1.260705377	1.35E-32	2.47E-28	25.7	6	yes	up
14	Op03g01226	1.259029933	4.71E-32	8.61E-28	36	11.1	yes	up
14	Op04g01089	1.256621745	2.27E-18	4.16E-14	47.3	25.3	yes	up
14	Op10g00522	1.253177628	1.16E-28	2.13E-24	13.1	2	yes	up
14	Op03g00777	1.251795153	5.67E-21	1.04E-16	50.5	26	yes	up
14	Op08g01328	1.249103499	6.84E-32	1.25E-27	32.4	9.3	yes	up
14	Op10g01428	1.241730687	4.71E-34	8.62E-30	83.3	56.5	yes	up
14	Op06g01595	1.237278116	3.13E-27	5.72E-23	74.3	47.9	yes	up
14	Op07g01109	1.236535358	1.71E-22	3.13E-18	54.5	28.4	yes	up

14	Op06g01290	1.234872293	3.70E-80	6.77E-76	14.9	0.9	yes	up
14	Op05g01442	1.23394575	3.18E-25	5.81E-21	35.6	12.6	yes	up
14	Op05g01716	1.231156572	4.77E-31	8.73E-27	25.2	6.1	yes	up
14	Op10g00377	1.228327306	6.04E-29	1.11E-24	39.6	14.3	yes	up
14	Op07g01294	1.225299772	2.59E-23	4.74E-19	70.7	48.8	yes	up
14	Op08g01806	1.224140109	1.91E-47	3.49E-43	24.8	4.2	yes	up
14	Op08g01670	1.22256574	1.45E-24	2.65E-20	23.9	6.5	yes	up
14	Op05g00089	1.222349889	1.16E-33	2.13E-29	30.6	8.1	yes	up
14	Op10g01131	1.221787601	3.36E-34	6.14E-30	32.9	9	yes	up
14	Op10g00199	1.209730859	7.82E-31	1.43E-26	19.8	4	yes	up
14	Op07g01545	1.206846536	1.61E-26	2.94E-22	58.6	29.3	yes	up
14	Op06g01390	1.205347242	3.03E-37	5.55E-33	22.1	4.2	yes	up
14	Op09g01573	1.204781652	4.03E-51	7.37E-47	23	3.4	yes	up
14	Op02g02040	1.198937643	1.26E-34	2.32E-30	39.2	12.2	yes	up
14	Op08g00925	1.194723235	2.94E-24	5.39E-20	45.9	20	yes	up
14	Op06g00587	1.190679962	1.17E-35	2.14E-31	24.8	5.2	yes	up
14	Op05g01815	1.189768206	2.79E-18	5.10E-14	18	4.9	yes	up
14	Op10g00984	1.186627109	1.66E-60	3.05E-56	16.7	1.5	yes	up
14	Op07g00533	1.183706782	3.48E-75	6.37E-71	19.8	1.7	yes	up
14	Op02g00842	1.181895877	1.85E-32	3.38E-28	22.5	4.8	yes	up
14	Op04g00108	1.181226248	1.27E-40	2.33E-36	23.9	4.5	yes	up
14	Op01g02096	1.180793768	6.02E-16	1.10E-11	28.4	11.4	yes	up
14	Op04g00446	1.174861585	1.50E-22	2.75E-18	27.9	9	yes	up
14	Op09g00398	1.174685029	3.52E-87	6.44E-83	12.2	0.5	yes	up
14	Op08g01781	1.17192931	4.19E-35	7.67E-31	26.6	6.1	yes	up
14	Op01g01909	1.170036771	3.79E-30	6.93E-26	82.9	61.4	yes	up
14	Op01g00718	1.167972552	7.40E-72	1.35E-67	18.9	1.7	yes	up
14	Op07g01069	1.164607185	6.19E-46	1.13E-41	24.8	4.3	yes	up
14	Op04g00661	1.161799836	1.29E-20	2.36E-16	18.5	4.7	yes	up
14	Op02g02125	1.15913155	7.19E-25	1.32E-20	16.7	3.4	yes	up
14	Op05g01585	1.158009682	2.29E-22	4.20E-18	34.2	12.9	yes	up
14	Op07g01950	1.157427121	8.19E-34	1.50E-29	50	18.7	yes	up
14	Op07g01814	1.157366203	3.11E-34	5.69E-30	31.1	8.1	yes	up
14	Op09g01572	1.156081387	1.33E-14	2.44E-10	15.8	4.6	yes	up
14	Op02g00572	1.155026274	4.48E-38	8.20E-34	29.3	6.7	yes	up
14	Op02g02242	1.151189922	1.58E-19	2.88E-15	43.7	20.6	yes	up
14	Op03g00001	1.146419289	5.66E-151	1.04E-146	20.3	0.7	yes	up
14	Op08g00004	1.145762945	1.98E-66	3.63E-62	14.9	1.1	yes	up
14	Op02g01741	1.144978056	6.28E-36	1.15E-31	31.5	8	yes	up
14	Op09g00656	1.143851165	1.08E-62	1.97E-58	17.1	1.6	yes	up
14	Op04g00383	1.143139159	7.25E-24	1.33E-19	30.6	10	yes	up
14	Op11g00573	1.143058215	5.13E-19	9.39E-15	34.7	14.5	yes	up
14	Op03g00092	1.142236776	8.51E-43	1.56E-38	81.5	41.5	yes	up
14	Op01g02355	1.141864351	9.22E-23	1.69E-18	76.1	56.7	yes	up
14	Op01g00916	1.138932322	7.25E-19	1.33E-14	43.7	21.6	yes	up
14	Op05g01823	1.138678401	1.56E-17	2.86E-13	60.4	37	yes	up
14	Op10g00851	1.138191339	3.55E-17	6.50E-13	38.7	18.2	yes	up
14	Op03g00685	1.134190309	1.13E-29	2.06E-25	74.3	45.5	yes	up
14	Op07g00353	1.13251003	6.91E-28	1.26E-23	82.9	59.7	yes	up
14	Op06g00731	1.130842055	8.57E-22	1.57E-17	47.7	22.8	yes	up
14	Op09g01525	1.129695622	1.57E-26	2.88E-22	59.5	28.9	yes	up
14	Op05g00843	1.128475874	1.11E-30	2.03E-26	45.9	16.6	yes	up
14	Op09g00972	1.127712033	1.48E-33	2.70E-29	34.7	9.6	yes	up
14	Op04g01619	1.126713114	1.66E-92	3.03E-88	10.8	0.3	yes	up
14	Op04g01572	1.124928419	7.98E-24	1.46E-19	26.1	7.6	yes	up
14	Op01g01025	1.121644573	5.82E-14	1.07E-09	44.6	26.3	yes	up
14	Op01g00698	1.121030235	2.66E-28	4.87E-24	36.9	12.4	yes	up
14	Op02g01212	1.120779193	3.27E-27	5.99E-23	29.3	8.7	yes	up
14	Op01g01911	1.120435654	6.48E-23	1.19E-18	20.3	5.2	yes	up
14	Op09g00581	1.118004822	3.38E-31	6.19E-27	93.2	86.7	yes	up
14	Op05g00313	1.112539873	5.34E-34	9.77E-30	33.3	9	yes	up
14	Op09g00622	1.1077122	6.49E-17	1.19E-12	13.1	3	yes	up

14	Op06g00421	1.105993053	3.18E-22	5.82E-18	29.7	10.2	yes	up
14	Op02g01364	1.105043498	3.89E-24	7.12E-20	57.2	30.7	yes	up
14	Op02g01459	1.101312177	1.17E-111	2.15E-107	10.4	0.2	yes	up
14	Op03g01997	1.100855612	5.44E-17	9.96E-13	51.8	30.2	yes	up
14	Op05g00623	1.100011651	5.29E-32	9.69E-28	28.8	7.5	yes	up
14	Op11g00286	1.099627634	4.51E-96	8.26E-92	18.5	1.1	yes	up
14	Op11g00371	1.099559816	5.64E-14	1.03E-09	15.3	4.6	yes	up
14	Op05g00074	1.097429596	4.23E-23	7.74E-19	76.1	53	yes	up
14	Op06g01138	1.097013034	8.34E-08	0.001526534	27.9	15.8	yes	up
14	Op03g00126	1.09647498	3.81E-13	6.98E-09	35.1	17.3	yes	up
14	Op02g02296	1.094803147	4.14E-33	7.57E-29	22.5	4.7	yes	up
14	Op08g01447	1.087289857	1.47E-44	2.69E-40	17.6	2.3	yes	up
14	Op04g00201	1.083713547	5.86E-49	1.07E-44	25.7	4.4	yes	up
14	Op04g00533	1.080937989	1.14E-98	2.08E-94	11.3	0.3	yes	up
14	Op01g00545	1.080567654	1.18E-13	2.16E-09	24.8	9.9	yes	up
14	Op07g01148	1.080311751	3.79E-24	6.95E-20	73.9	52.2	yes	up
14	Op06g01519	1.078179607	6.48E-23	1.19E-18	25.2	7.5	yes	up
14	Op08g01657	1.076289	2.13E-14	3.90E-10	21.2	7.4	yes	up
14	Op11g00496	1.075071056	1.73E-24	3.17E-20	41.4	16	yes	up
14	Op11g00773	1.073507349	3.87E-25	7.08E-21	13.1	2.2	yes	up
14	Op01g02110	1.071996783	7.80E-12	1.43E-07	41	22.8	yes	up
14	Op10g01544	1.06855877	1.18E-10	2.16E-06	16.7	6.2	yes	up
14	Op09g00355	1.065299546	3.41E-14	6.24E-10	38.3	19.9	yes	up
14	Op11g01289	1.064212558	1.05E-28	1.93E-24	15.8	2.8	yes	up
14	Op01g00625	1.063273528	2.88E-40	5.27E-36	21.2	3.5	yes	up
14	Op08g01246	1.056457832	6.49E-21	1.19E-16	21.2	6	yes	up
14	Op09g01315	1.054563875	7.88E-46	1.44E-41	15.8	1.8	yes	up
14	Op01g01656	1.044573193	1.80E-29	3.29E-25	24.3	5.8	yes	up
14	Op07g01606	1.039151759	2.28E-20	4.17E-16	37.8	15.8	yes	up
14	Op07g01278	1.038494903	1.04E-17	1.91E-13	36.5	16.3	yes	up
14	Op11g01294	1.035620663	2.63E-23	4.82E-19	41.9	16.6	yes	up
14	Op03g01758	1.034355916	7.14E-17	1.31E-12	66.7	45.9	yes	up
14	Op01g00243	1.027935827	2.94E-62	5.38E-58	16.2	1.4	yes	up
14	Op01g02162	1.027876773	1.04E-24	1.90E-20	22.5	5.8	yes	up
14	Op09g01275	1.026898171	7.21E-46	1.32E-41	14.9	1.6	yes	up
14	Op01g01797	1.025180685	1.70E-68	3.11E-64	17.6	1.5	yes	up
14	Op06g00348	1.022482667	6.45E-55	1.18E-50	15.3	1.4	yes	up
14	Op07g00044	1.016992507	4.65E-49	8.51E-45	18.5	2.3	yes	up
14	Op07g01575	1.015586272	5.90E-30	1.08E-25	21.6	4.7	yes	up
14	Op03g02084	1.015458791	1.87E-28	3.42E-24	24.3	6	yes	up
14	Op10g00352	1.014200777	2.66E-16	4.87E-12	28.8	11.5	yes	up
14	Op01g00008	1.013709221	8.11E-32	1.49E-27	21.6	4.5	yes	up
14	Op04g00320	1.01354282	2.72E-17	4.99E-13	55.4	31.7	yes	up
14	Op03g01491	1.013238988	2.16E-19	3.96E-15	30.2	11.1	yes	up
14	Op09g00958	1.012238794	2.44E-10	4.47E-06	14.9	5.3	yes	up
14	Op02g02191	1.012166475	8.90E-40	1.63E-35	18.9	2.9	yes	up
14	Op07g01226	1.009907716	8.30E-22	1.52E-17	33.3	12.2	yes	up
14	Op05g00889	1.009191612	7.05E-57	1.29E-52	15.3	1.4	yes	up
14	Op06g01482	1.009072389	1.82E-40	3.33E-36	22.1	3.7	yes	up
14	Op03g01965	1.005587724	5.51E-15	1.01E-10	40.5	21.2	yes	up
14	Op05g01008	1.005551319	1.65E-24	3.02E-20	17.6	3.8	yes	up
14	Op01g02036	1.004879414	1.17E-17	2.14E-13	40.5	18.4	yes	up
14	Op09g00915	1.004366371	5.03E-12	9.21E-08	46.8	29.2	yes	up
14	Op10g00545	1.00295873	3.59E-11	6.57E-07	23	10	yes	up
14	Op01g01729	1.001374808	1.49E-15	2.73E-11	45.9	23.5	yes	up
15	Op06g01438	4.226558065	4.82E-189	8.83E-185	80.7	12.5	yes	up
15	Op10g00760	3.753401744	2.34E-164	4.28E-160	77.5	13.3	yes	up
15	Op04g01300	3.728892454	0	0	75.9	5.1	yes	up
15	Op07g01569	3.548961997	1.49E-41	2.73E-37	63.6	27.2	yes	up
15	Op09g00404	3.423316048	1.08E-184	1.97E-180	84	12.7	yes	up
15	Op05g00316	3.414781812	0	0	66.3	4.3	yes	up
15	Op07g00682	3.09865018	1.67E-171	3.06E-167	86.6	16.1	yes	up

15	Op02g00410	2.998097128	3.41E-175	6.24E-171	59.4	6.3	yes	up
15	Op05g01587	2.853771805	2.85E-199	5.22E-195	64.7	6.5	yes	up
15	Op03g00677	2.822307912	1.22E-211	2.24E-207	79.1	10	yes	up
15	Op03g02104	2.622939805	0	0	50.8	1.4	yes	up
15	Op02g00534	2.552406556	3.90E-151	7.14E-147	75.4	11.8	yes	up
15	Op06g00176	2.535899518	4.41E-146	8.08E-142	46.5	4.4	yes	up
15	Op11g00931	2.476642455	1.51E-112	2.76E-108	90.4	26.2	yes	up
15	Op06g00911	2.409757115	9.46E-154	1.73E-149	65.2	8.5	yes	up
15	Op07g01208	2.383802456	4.72E-96	8.64E-92	70.6	16.3	yes	up
15	Op09g01072	2.318616666	1.92E-184	3.52E-180	58.8	5.6	yes	up
15	Op11g00898	2.30850808	3.48E-81	6.38E-77	100	92.5	yes	up
15	Op07g01059	2.278204127	2.32E-80	4.24E-76	85	32	yes	up
15	Op01g00652	2.271124187	4.67E-118	8.54E-114	75.4	15.7	yes	up
15	Op03g00742	2.25959807	4.97E-217	9.10E-213	61.5	5.2	yes	up
15	Op04g01212	2.235239823	2.36E-307	4.33E-303	56.1	2.8	yes	up
15	Op06g00314	2.196321445	5.24E-85	9.59E-81	77.5	22.6	yes	up
15	Op10g00576	2.190114484	5.32E-143	9.74E-139	49.2	5	yes	up
15	Op11g00482	2.174566776	1.77E-105	3.23E-101	40.1	4.5	yes	up
15	Op03g00063	2.114313303	5.62E-153	1.03E-148	65.8	8.5	yes	up
15	Op04g01386	2.096054466	2.67E-67	4.88E-63	38	6.1	yes	up
15	Op11g01149	2.083136523	2.57E-88	4.70E-84	84.5	29.7	yes	up
15	Op08g01277	2.06764484	1.43E-73	2.62E-69	71.1	20.9	yes	up
15	Op03g00176	2.022268996	7.02E-112	1.29E-107	71.1	13.6	yes	up
15	Op07g00629	1.994088428	7.07E-104	1.29E-99	55.1	8.5	yes	up
15	Op02g00382	1.951693873	3.65E-178	6.68E-174	57.8	5.5	yes	up
15	Op04g01278	1.944902635	1.15E-128	2.10E-124	58.8	7.9	yes	up
15	Op09g01377	1.928034787	2.27E-117	4.15E-113	67.4	12.1	yes	up
15	Op09g00427	1.914822853	9.70E-110	1.78E-105	48.1	6.3	yes	up
15	Op09g00105	1.897534022	1.93E-73	3.53E-69	57.8	12.8	yes	up
15	Op09g00405	1.886093402	6.23E-33	1.14E-28	71.1	33.8	yes	up
15	Op05g00313	1.879222624	2.09E-148	3.82E-144	64.7	8.4	yes	up
15	Op10g01176	1.878210847	5.11E-83	9.35E-79	82.9	25.5	yes	up
15	Op01g00609	1.871967981	1.68E-103	3.08E-99	48.1	6.6	yes	up
15	Op08g00841	1.846514862	0	0	35.3	0.4	yes	up
15	Op08g01583	1.846476748	2.92E-133	5.34E-129	55.1	6.7	yes	up
15	Op02g00700	1.809717632	4.62E-70	8.46E-66	66.3	17.1	yes	up
15	Op11g00727	1.803914853	1.04E-67	1.91E-63	57.8	13.7	yes	up
15	Op11g01208	1.803335681	1.46E-233	2.67E-229	48.7	2.9	yes	up
15	Op08g01684	1.791929502	7.69E-98	1.41E-93	70.1	15.1	yes	up
15	Op02g00469	1.784547605	1.55E-143	2.84E-139	45.5	4.2	yes	up
15	Op11g00773	1.784045373	1.35E-280	2.47E-276	42.8	1.6	yes	up
15	Op02g01918	1.778510185	2.42E-143	4.42E-139	31	1.9	yes	up
15	Op01g01702	1.776399202	1.58E-99	2.89E-95	70.6	13.9	yes	up
15	Op05g00409	1.767841848	6.18E-77	1.13E-72	91.4	41.4	yes	up
15	Op02g02152	1.761116803	3.26E-62	5.97E-58	41.2	7.6	yes	up
15	Op07g00217	1.755358198	3.31E-99	6.06E-95	59.9	10.4	yes	up
15	Op04g00252	1.743050403	3.00E-74	5.49E-70	79.7	24.1	yes	up
15	Op02g02051	1.737978824	1.98E-54	3.62E-50	29.4	4.5	yes	up
15	Op05g01373	1.736145329	1.84E-174	3.37E-170	21.4	0.6	yes	up
15	Op02g01804	1.734593867	1.07E-85	1.96E-81	89.3	28.8	yes	up
15	Op10g00675	1.727094104	1.28E-75	2.34E-71	88.2	29.3	yes	up
15	Op06g01432	1.72247369	6.52E-99	1.19E-94	50.3	7.2	yes	up
15	Op05g01923	1.713018324	1.12E-67	2.05E-63	83.4	33.8	yes	up
15	Op11g00930	1.712794869	4.92E-89	9.00E-85	73.3	17.4	yes	up
15	Op08g01617	1.706947041	7.90E-74	1.45E-69	50.8	9.7	yes	up
15	Op03g01421	1.696156964	5.49E-70	1.00E-65	43.9	7.6	yes	up
15	Op09g00519	1.680282959	3.76E-59	6.88E-55	96.8	69.1	yes	up
15	Op02g01635	1.676805772	2.47E-175	4.53E-171	49.2	4	yes	up
15	Op07g01352	1.67203229	2.22E-187	4.06E-183	24.1	0.7	yes	up
15	Op04g01213	1.654708711	1.23E-290	2.26E-286	31	0.7	yes	up
15	Op01g02347	1.653202321	2.39E-98	4.37E-94	53.5	8.5	yes	up
15	Op05g00648	1.65177843	2.13E-58	3.90E-54	67.4	21.1	yes	up

15	Op10g00526	1.64893268	8.54E-108	1.56E-103	55.1	8	yes	up
15	Op02g00653	1.63704982	6.66E-81	1.22E-76	40.6	5.7	yes	up
15	Op06g00332	1.635004306	1.61E-29	2.95E-25	27.8	6.8	yes	up
15	Op02g01776	1.631208049	1.38E-102	2.53E-98	18.7	0.9	yes	up
15	Op08g01657	1.621406936	1.13E-88	2.07E-84	46.5	7	yes	up
15	Op01g02338	1.609390719	4.65E-106	8.52E-102	71.1	13.6	yes	up
15	Op11g00365	1.60901834	2.39E-151	4.38E-147	34.8	2.3	yes	up
15	Op03g01458	1.60409433	3.10E-60	5.67E-56	93.6	51.1	yes	up
15	Op05g00359	1.593402889	1.36E-82	2.49E-78	36.9	4.7	yes	up
15	Op09g00972	1.586287297	1.02E-81	1.87E-77	52.4	9.4	yes	up
15	Op05g01934	1.577327589	5.11E-74	9.36E-70	89.3	31.2	yes	up
15	Op08g00998	1.574317902	2.38E-76	4.35E-72	81.8	23.5	yes	up
15	Op01g00944	1.570890554	6.15E-130	1.13E-125	36.4	2.9	yes	up
15	Op05g01874	1.56841088	1.29E-58	2.36E-54	61	16.7	yes	up
15	Op02g01588	1.539514912	3.25E-43	5.94E-39	40.1	10	yes	up
15	Op01g02073	1.537413891	4.77E-60	8.74E-56	92.5	50.3	yes	up
15	Op03g00744	1.536927106	1.48E-126	2.72E-122	34.2	2.7	yes	up
15	Op08g01207	1.53186312	8.79E-62	1.61E-57	35.3	5.7	yes	up
15	Op03g01658	1.525412648	9.24E-57	1.69E-52	53.5	13.1	yes	up
15	Op08g00079	1.514122267	1.40E-68	2.57E-64	57.2	12.5	yes	up
15	Op11g00194	1.498936204	7.96E-09	0.000145723	38.5	21.4	yes	up
15	Op08g00012	1.497038878	4.94E-105	9.04E-101	55.1	8.1	yes	up
15	Op03g00293	1.495168332	6.02E-147	1.10E-142	41.2	3.3	yes	up
15	Op03g01589	1.493547939	5.83E-145	1.07E-140	41.2	3.4	yes	up
15	Op06g00594	1.493500273	3.01E-162	5.50E-158	40.1	2.8	yes	up
15	Op10g00724	1.492418232	1.46E-147	2.68E-143	39	2.9	yes	up
15	Op03g02103	1.491082687	0	0	34.8	0.7	yes	up
15	Op08g01437	1.490749388	4.41E-63	8.07E-59	71.7	21.9	yes	up
15	Op08g01612	1.472162665	1.78E-45	3.25E-41	35.8	7.6	yes	up
15	Op06g01436	1.461802539	2.86E-189	5.24E-185	19.8	0.4	yes	up
15	Op09g00068	1.452700057	5.44E-84	9.96E-80	44.9	6.8	yes	up
15	Op01g01729	1.439222756	7.63E-39	1.40E-34	63.1	23.2	yes	up
15	Op03g01232	1.434976754	1.25E-51	2.29E-47	56.1	15.4	yes	up
15	Op01g01724	1.425928638	8.98E-47	1.64E-42	26.7	4.3	yes	up
15	Op06g00706	1.423992507	4.94E-84	9.04E-80	52.9	9.4	yes	up
15	Op11g00630	1.423583077	9.67E-151	1.77E-146	28.9	1.5	yes	up
15	Op09g01399	1.423379016	2.88E-57	5.28E-53	74.3	25.5	yes	up
15	Op07g00537	1.421914226	1.22E-58	2.24E-54	41.2	7.8	yes	up
15	Op06g00231	1.415374128	5.11E-49	9.36E-45	69.5	25.2	yes	up
15	Op06g01435	1.414844844	2.37E-81	4.34E-77	56.1	10.4	yes	up
15	Op09g00429	1.412797932	2.03E-52	3.71E-48	95.2	62.5	yes	up
15	Op02g00227	1.41091884	5.49E-105	1.01E-100	20.9	1.1	yes	up
15	Op07g00858	1.398962045	6.70E-166	1.23E-161	29.9	1.4	yes	up
15	Op11g00141	1.387305826	2.52E-73	4.61E-69	98.9	79	yes	up
15	Op02g01491	1.376969061	3.48E-67	6.37E-63	36.9	5.6	yes	up
15	Op11g01183	1.374818132	1.74E-145	3.19E-141	32.6	2	yes	up
15	Op02g02085	1.371759717	1.05E-102	1.92E-98	16.6	0.7	yes	up
15	Op03g02206	1.368167551	1.16E-84	2.12E-80	15	0.7	yes	up
15	Op05g00592	1.364717152	8.47E-173	1.55E-168	43.3	3.1	yes	up
15	Op06g00351	1.343881663	5.75E-62	1.05E-57	37.4	6.3	yes	up
15	Op06g01469	1.34242784	6.18E-134	1.13E-129	34.8	2.6	yes	up
15	Op11g01302	1.34016639	3.18E-70	5.82E-66	15	0.9	yes	up
15	Op07g01536	1.340065413	1.38E-59	2.53E-55	63.6	17.6	yes	up
15	Op06g01431	1.339636411	4.49E-46	8.22E-42	20.9	2.7	yes	up
15	Op08g01815	1.337282592	1.93E-61	3.53E-57	54	12.7	yes	up
15	Op08g01173	1.336069545	1.12E-48	2.04E-44	72.7	26.2	yes	up
15	Op05g00301	1.3355052	1.38E-69	2.52E-65	57.2	12.5	yes	up
15	Op03g01969	1.330183932	1.13E-68	2.07E-64	43.3	7.6	yes	up
15	Op06g00087	1.323144435	1.75E-54	3.20E-50	52.9	12.8	yes	up
15	Op07g00348	1.314399104	1.72E-94	3.14E-90	33.7	3.5	yes	up
15	Op08g01004	1.302864012	1.35E-107	2.47E-103	39	4.1	yes	up
15	Op07g00545	1.291971076	1.11E-98	2.04E-94	36.9	3.9	yes	up

15	Op02g00532	1.287526917	2.82E-65	5.17E-61	44.9	8.6	yes	up
15	Op10g00427	1.284327011	4.34E-55	7.95E-51	49.7	11.7	yes	up
15	Op05g00217	1.283413216	7.09E-66	1.30E-61	39.6	6.6	yes	up
15	Op04g01614	1.281875299	8.87E-38	1.62E-33	91.4	59.3	yes	up
15	Op01g02538	1.281156421	5.37E-140	9.82E-136	33.2	2.2	yes	up
15	Op05g00173	1.277924248	3.13E-39	5.72E-35	86.6	46.3	yes	up
15	Op09g01210	1.266097842	4.56E-80	8.35E-76	48.1	7.8	yes	up
15	Op03g01163	1.265906551	2.78E-67	5.09E-63	63.6	14.9	yes	up
15	Op05g00225	1.259634092	7.43E-198	1.36E-193	26.2	0.8	yes	up
15	Op11g01303	1.256641946	3.61E-93	6.60E-89	28.9	2.6	yes	up
15	Op02g01461	1.254213667	3.66E-59	6.70E-55	42.8	8.1	yes	up
15	Op07g01326	1.247374216	1.74E-57	3.18E-53	99.5	75.6	yes	up
15	Op07g00647	1.245840579	5.25E-37	9.60E-33	19.8	3	yes	up
15	Op02g00036	1.244310371	1.49E-43	2.72E-39	91.4	58.9	yes	up
15	Op03g02125	1.242624091	2.19E-46	4.02E-42	93.6	62.4	yes	up
15	Op02g01529	1.240631677	2.04E-25	3.73E-21	46.5	17.6	yes	up
15	Op03g00054	1.230965494	9.51E-58	1.74E-53	39.6	7.2	yes	up
15	Op10g00168	1.229514871	1.73E-171	3.17E-167	34.2	1.9	yes	up
15	Op11g00144	1.219132309	4.04E-62	7.40E-58	36.4	5.8	yes	up
15	Op04g01209	1.218900137	1.01E-55	1.84E-51	39	7.3	yes	up
15	Op03g00654	1.214881812	2.17E-38	3.96E-34	43.9	11.6	yes	up
15	Op01g00279	1.211784948	1.29E-38	2.37E-34	49.7	15.1	yes	up
15	Op10g01263	1.210942603	6.72E-16	1.23E-11	57.2	34.2	yes	up
15	Op03g02093	1.209083659	9.56E-36	1.75E-31	52.9	18.5	yes	up
15	Op01g00326	1.208355284	3.57E-76	6.54E-72	44.4	7.1	yes	up
15	Op04g00030	1.202692417	2.89E-63	5.29E-59	40.6	7.2	yes	up
15	Op01g01545	1.200323682	5.02E-58	9.19E-54	63.6	17.2	yes	up
15	Op10g00449	1.199414562	1.86E-76	3.40E-72	46	7.6	yes	up
15	Op05g01261	1.198762143	2.99E-76	5.47E-72	34.8	4.5	yes	up
15	Op11g00717	1.197422459	1.77E-53	3.24E-49	42.2	9	yes	up
15	Op03g02129	1.194170366	9.23E-97	1.69E-92	17.1	0.8	yes	up
15	Op11g00259	1.188300922	4.82E-64	8.83E-60	54	11.7	yes	up
15	Op02g00035	1.188197848	1.24E-44	2.27E-40	36.9	7.9	yes	up
15	Op05g01244	1.178563536	3.01E-76	5.51E-72	10.2	0.3	yes	up
15	Op02g00865	1.175968721	1.33E-48	2.44E-44	14.4	1.2	yes	up
15	Op09g00296	1.174779767	4.30E-55	7.86E-51	61.5	16.9	yes	up
15	Op01g01392	1.167622729	1.95E-52	3.57E-48	56.1	14.7	yes	up
15	Op03g00579	1.166788381	1.60E-59	2.93E-55	41.2	7.6	yes	up
15	Op10g00229	1.165182652	5.38E-35	9.85E-31	34.8	8.5	yes	up
15	Op09g01352	1.164401002	5.64E-35	1.03E-30	90.4	62	yes	up
15	Op01g01656	1.16270282	1.46E-44	2.67E-40	31	5.7	yes	up
15	Op04g01502	1.153840869	1.45E-24	2.66E-20	85.6	59	yes	up
15	Op03g02334	1.153772119	6.47E-42	1.19E-37	42.8	10.9	yes	up
15	Op04g00927	1.153733754	8.71E-30	1.59E-25	55.1	20.9	yes	up
15	Op10g00656	1.152492492	3.90E-54	7.14E-50	70.1	20.6	yes	up
15	Op10g01133	1.150137359	6.82E-107	1.25E-102	25.7	1.7	yes	up
15	Op03g01775	1.141182418	8.81E-54	1.61E-49	45.5	10.1	yes	up
15	Op03g01412	1.132189942	8.90E-43	1.63E-38	41.7	10.1	yes	up
15	Op11g01423	1.130869128	4.32E-69	7.91E-65	40.1	6.3	yes	up
15	Op07g01575	1.130515879	2.03E-60	3.72E-56	31.6	4.5	yes	up
15	Op10g00099	1.123391256	3.43E-58	6.28E-54	37.4	6.6	yes	up
15	Op04g01534	1.113017253	7.51E-45	1.38E-40	58.3	17.3	yes	up
15	Op04g01423	1.112064856	4.80E-40	8.79E-36	42.8	11.1	yes	up
15	Op05g01648	1.110422617	5.21E-56	9.55E-52	29.4	4.3	yes	up
15	Op05g01586	1.108758013	6.16E-57	1.13E-52	30.5	4.5	yes	up
15	Op05g00613	1.108135718	1.27E-72	2.33E-68	36.9	5.3	yes	up
15	Op06g00292	1.106864427	6.81E-77	1.25E-72	34.2	4.3	yes	up
15	Op02g00826	1.101841472	2.54E-86	4.65E-82	21.4	1.5	yes	up
15	Op11g00439	1.090869619	3.41E-69	6.24E-65	49.7	9.3	yes	up
15	Op11g00544	1.087900119	3.20E-28	5.86E-24	69	32.9	yes	up
15	Op05g01678	1.087492546	5.64E-90	1.03E-85	29.4	2.8	yes	up
15	Op09g00262	1.0856333	9.14E-16	1.67E-11	28.9	10.8	yes	up

15	Op03g01441	1.082722834	7.52E-40	1.38E-35	70.6	27.2	yes	up
15	Op07g01175	1.080627921	1.19E-42	2.18E-38	29.4	5.5	yes	up
15	Op08g00930	1.080095869	1.06E-37	1.94E-33	89.3	56.1	yes	up
15	Op07g01360	1.076446889	3.78E-33	6.92E-29	66.3	29	yes	up
15	Op03g00475	1.067361113	1.70E-40	3.11E-36	47.6	13.3	yes	up
15	Op03g00656	1.06730031	3.21E-60	5.87E-56	33.2	5.1	yes	up
15	Op02g02255	1.062654036	6.90E-48	1.26E-43	49.7	12.6	yes	up
15	Op06g00081	1.061804123	4.71E-39	8.62E-35	75.9	33.2	yes	up
15	Op03g02060	1.060717885	7.61E-67	1.39E-62	40.6	6.7	yes	up
15	Op07g00725	1.05733375	9.99E-77	1.83E-72	30.5	3.4	yes	up
15	Op08g00322	1.05672351	4.65E-78	8.52E-74	36.9	4.9	yes	up
15	Op09g00179	1.055796615	4.60E-42	8.42E-38	67.4	24.7	yes	up
15	Op01g01102	1.052807594	2.11E-150	3.86E-146	27.8	1.4	yes	up
15	Op02g02015	1.052608475	1.18E-43	2.16E-39	52.9	15.1	yes	up
15	Op09g00139	1.047701994	1.30E-51	2.37E-47	34.8	6.4	yes	up
15	Op07g00477	1.043500451	8.19E-62	1.50E-57	41.7	7.4	yes	up
15	Op06g01442	1.040789993	1.04E-40	1.91E-36	64.2	21.4	yes	up
15	Op06g00227	1.039197024	1.55E-41	2.84E-37	52.4	15.3	yes	up
15	Op10g01545	1.037907974	6.65E-49	1.22E-44	51.9	13	yes	up
15	Op04g01602	1.037643614	5.87E-40	1.08E-35	45.5	12.6	yes	up
15	Op02g00358	1.036360878	6.84E-34	1.25E-29	39	10.6	yes	up
15	Op01g00719	1.03483886	1.14E-100	2.09E-96	30.5	2.6	yes	up
15	Op01g00293	1.033579182	2.36E-47	4.31E-43	98.4	74	yes	up
15	Op02g02048	1.032982763	6.21E-17	1.14E-12	24.1	7.4	yes	up
15	Op11g00919	1.03022321	1.91E-54	3.50E-50	40.1	7.7	yes	up
15	Op10g00691	1.025033173	2.63E-50	4.82E-46	36.9	7	yes	up
15	Op04g00401	1.018774071	7.74E-37	1.42E-32	50.3	15.6	yes	up
15	Op07g01790	1.017585139	1.56E-90	2.86E-86	22.5	1.6	yes	up
15	Op06g00944	1.016656444	1.13E-59	2.07E-55	44.4	8.4	yes	up
15	Op11g00308	1.015798301	5.13E-30	9.39E-26	70.1	34.5	yes	up
15	Op07g01737	1.011808712	1.35E-43	2.47E-39	45.5	11.3	yes	up
15	Op01g01723	1.011124193	1.34E-53	2.45E-49	31	4.9	yes	up
15	Op03g02111	1.0099035	8.01E-61	1.47E-56	39	6.9	yes	up
15	Op02g00368	1.003855795	5.85E-45	1.07E-40	70.6	24	yes	up
15	Op10g00807	1.00096401	2.41E-173	4.41E-169	23	0.7	yes	up
15	Op07g01890	1.000649588	1.32E-115	2.42E-111	27.8	1.9	yes	up
16	Op01g02118	5.841726413	1.41E-238	2.58E-234	40	0.8	yes	up
16	Op03g01788	5.242506143	8.34E-219	1.53E-214	57.3	2	yes	up
16	Op11g00903	4.622469926	6.07E-104	1.11E-99	58.7	4.8	yes	up
16	Op05g00863	4.480357971	7.41E-96	1.36E-91	89.3	14.3	yes	up
16	Op11g01075	4.42954793	3.91E-180	7.15E-176	41.3	1.2	yes	up
16	Op11g00362	4.109821107	2.06E-58	3.76E-54	62.7	10	yes	up
16	Op11g00482	4.097765999	1.12E-120	2.05E-116	62.7	4.7	yes	up
16	Op03g01775	3.756911936	6.97E-59	1.28E-54	64	10.3	yes	up
16	Op08g01585	3.741487066	2.54E-227	4.64E-223	42.7	0.9	yes	up
16	Op02g01352	3.565242934	3.32E-100	6.07E-96	60	5.2	yes	up
16	Op03g02211	3.437218441	5.64E-238	1.03E-233	49.3	1.3	yes	up
16	Op05g01815	3.381596521	1.30E-52	2.38E-48	42.7	5	yes	up
16	Op11g00242	3.363955988	6.42E-59	1.18E-54	84	18.9	yes	up
16	Op05g01923	3.318087546	6.96E-51	1.27E-46	92	34.3	yes	up
16	Op09g01299	3.299511722	0	0	30.7	0.2	yes	up
16	Op03g01880	3.277610236	3.49E-91	6.39E-87	58.7	5.5	yes	up
16	Op07g01462	3.267803643	8.52E-58	1.56E-53	68	12.2	yes	up
16	Op01g00850	3.228367475	7.85E-41	1.44E-36	85.3	32.2	yes	up
16	Op01g02036	3.227844946	3.45E-20	6.32E-16	53.3	18.7	yes	up
16	Op03g00212	3.16814883	4.99E-13	9.13E-09	64	41.9	yes	up
16	Op06g00836	3.162606308	2.20E-19	4.03E-15	28	5.3	yes	up
16	Op07g01059	3.146825433	2.87E-23	5.26E-19	72	32.8	yes	up
16	Op11g00604	3.118610887	1.10E-144	2.01E-140	50.7	2.4	yes	up
16	Op02g01074	3.059992083	3.52E-27	6.45E-23	65.3	22.5	yes	up
16	Op03g02213	3.046873015	3.45E-52	6.31E-48	40	4.2	yes	up
16	Op08g00508	3.043451159	8.93E-47	1.64E-42	65.3	13.2	yes	up

16	Op01g00545	3.037517664	2.15E-55	3.93E-51	61.3	9.8	yes	up
16	Op05g01076	3.036060329	8.25E-141	1.51E-136	57.3	3.2	yes	up
16	Op11g00904	3.034971457	4.50E-21	8.24E-17	30.7	5.7	yes	up
16	Op02g01676	3.006129201	1.46E-106	2.67E-102	41.3	2.2	yes	up
16	Op01g01135	2.992439017	7.51E-116	1.37E-111	42.7	2.1	yes	up
16	Op03g00132	2.959248582	1.58E-47	2.89E-43	74.7	17.4	yes	up
16	Op07g01699	2.955581128	7.03E-104	1.29E-99	34.7	1.5	yes	up
16	Op05g00549	2.941536682	3.04E-38	5.57E-34	34.7	4.4	yes	up
16	Op10g01133	2.914145294	1.22E-129	2.24E-125	42.7	1.9	yes	up
16	Op02g01671	2.884213857	9.75E-92	1.78E-87	34.7	1.8	yes	up
16	Op02g01214	2.86789046	1.17E-34	2.14E-30	33.3	4.3	yes	up
16	Op03g01959	2.826952282	7.29E-98	1.33E-93	32	1.4	yes	up
16	Op05g00437	2.821900532	1.53E-13	2.80E-09	41.3	15.1	yes	up
16	Op05g01310	2.813088074	3.75E-37	6.86E-33	52	9.8	yes	up
16	Op01g00726	2.78974442	8.56E-69	1.57E-64	60	7.5	yes	up
16	Op07g01382	2.784713995	6.79E-110	1.24E-105	52	3.4	yes	up
16	Op04g00162	2.774828685	0	0	53.3	0.6	yes	up
16	Op08g01617	2.768829691	3.48E-35	6.38E-31	52	10.1	yes	up
16	Op06g01519	2.762463595	2.24E-55	4.10E-51	54.7	7.6	yes	up
16	Op04g01560	2.742540653	7.83E-38	1.43E-33	49.3	9	yes	up
16	Op11g00371	2.727291884	3.01E-41	5.51E-37	37.3	4.6	yes	up
16	Op02g01831	2.717914766	2.03E-38	3.72E-34	50.7	9.2	yes	up
16	Op04g00130	2.675386717	1.05E-216	1.92E-212	30.7	0.5	yes	up
16	Op01g00750	2.649178572	3.08E-16	5.63E-12	77.3	49.1	yes	up
16	Op06g00374	2.631672237	3.49E-45	6.39E-41	41.3	5.3	yes	up
16	Op09g00182	2.618984287	5.70E-24	1.04E-19	54.7	16.9	yes	up
16	Op11g00573	2.567852531	4.91E-22	8.99E-18	50.7	14.7	yes	up
16	Op01g00773	2.557226923	8.13E-17	1.49E-12	60	26.6	yes	up
16	Op06g00397	2.552426335	1.44E-106	2.63E-102	49.3	3.2	yes	up
16	Op03g01320	2.547550575	1.60E-74	2.92E-70	36	2.4	yes	up
16	Op03g00126	2.547028552	5.89E-18	1.08E-13	50.7	17.4	yes	up
16	Op09g00421	2.519402212	8.64E-143	1.58E-138	34.7	1.1	yes	up
16	Op07g00791	2.482337434	1.02E-32	1.87E-28	80	30	yes	up
16	Op06g00314	2.461157766	1.83E-08	0.000335728	48	23.5	yes	up
16	Op09g00448	2.45096771	3.51E-33	6.42E-29	93.3	46.2	yes	up
16	Op09g01574	2.441259591	5.75E-18	1.05E-13	69.3	31.9	yes	up
16	Op04g00562	2.411590429	2.40E-33	4.39E-29	44	7.8	yes	up
16	Op03g01177	2.375112186	1.50E-136	2.75E-132	30.7	0.8	yes	up
16	Op04g00661	2.36580271	5.07E-68	9.28E-64	48	4.7	yes	up
16	Op01g02401	2.353906075	0	0	41.3	0.2	yes	up
16	Op03g02226	2.351408749	9.84E-43	1.80E-38	44	6.1	yes	up
16	Op03g00522	2.33757189	0	0	25.3	0.1	yes	up
16	Op04g00035	2.336824103	1.32E-116	2.41E-112	34.7	1.4	yes	up
16	Op03g00253	2.328847312	1.07E-09	1.95E-05	66.7	54.5	yes	up
16	Op01g01718	2.308204426	5.50E-36	1.01E-31	44	7.1	yes	up
16	Op08g01484	2.304616695	9.54E-13	1.75E-08	54.7	24.1	yes	up
16	Op06g00520	2.30438563	1.17E-11	2.15E-07	74.7	57.6	yes	up
16	Op05g01682	2.290417711	5.21E-26	9.54E-22	41.3	8.5	yes	up
16	Op10g01131	2.266809438	9.49E-33	1.74E-28	48	9.2	yes	up
16	Op05g01411	2.258585927	1.55E-53	2.84E-49	44	5	yes	up
16	Op10g01146	2.254103683	2.89E-50	5.29E-46	30.7	2.6	yes	up
16	Op09g00174	2.250161919	1.05E-12	1.92E-08	58.7	33.1	yes	up
16	Op01g00205	2.249847188	1.02E-42	1.87E-38	61.3	12.2	yes	up
16	Op02g01822	2.218624018	5.56E-09	0.000101758	29.3	10.4	yes	up
16	Op02g01461	2.20821354	2.34E-28	4.28E-24	44	8.5	yes	up
16	Op03g02165	2.195035812	3.94E-43	7.22E-39	32	3.3	yes	up
16	Op11g00765	2.194752854	1.90E-25	3.49E-21	42.7	9.4	yes	up
16	Op10g00545	2.185923109	2.22E-16	4.06E-12	37.3	10.1	yes	up
16	Op10g00753	2.183210076	1.45E-23	2.65E-19	13.3	1.1	yes	up
16	Op02g01860	2.153167318	6.48E-50	1.19E-45	37.3	3.9	yes	up
16	Op02g02170	2.141110425	4.31E-33	7.89E-29	54.7	11.5	yes	up
16	Op03g01288	2.137543454	9.89E-22	1.81E-17	41.3	9.9	yes	up

16	Op07g00875	2.137139346	1.11E-222	2.04E-218	29.3	0.4	yes	up
16	Op09g01525	2.13420246	2.03E-16	3.71E-12	64	29.4	yes	up
16	Op05g00239	2.128421301	1.48E-46	2.71E-42	52	8.1	yes	up
16	Op05g00816	2.120402112	1.73E-37	3.16E-33	36	4.7	yes	up
16	Op02g01893	2.113333493	2.20E-24	4.03E-20	42.7	9.2	yes	up
16	Op10g01164	2.096121276	7.03E-23	1.29E-18	50.7	13.9	yes	up
16	Op11g00333	2.09591199	3.12E-12	5.72E-08	37.3	12.2	yes	up
16	Op07g01268	2.084484379	3.03E-60	5.55E-56	38.7	3.4	yes	up
16	Op08g00160	2.083156097	4.05E-25	7.42E-21	68	24.1	yes	up
16	Op05g01418	2.080153778	7.64E-32	1.40E-27	50.7	10.6	yes	up
16	Op03g02060	2.063006831	7.47E-39	1.37E-34	45.3	7.1	yes	up
16	Op04g00527	2.061221404	6.10E-29	1.12E-24	57.3	15.1	yes	up
16	Op11g00888	2.058821326	6.30E-07	0.011535159	57.3	36.9	yes	up
16	Op03g00176	2.0578823	1.11E-32	2.04E-28	61.3	14.4	yes	up
16	Op04g00614	2.054304879	0	0	30.7	0.1	yes	up
16	Op05g01775	2.035362335	2.21E-41	4.04E-37	36	4.2	yes	up
16	Op11g01131	2.024090896	2.04E-250	3.74E-246	21.3	0.1	yes	up
16	Op07g00183	2.020011534	4.85E-41	8.87E-37	36	4.3	yes	up
16	Op09g01471	2.019525878	9.72E-160	1.78E-155	30.7	0.7	yes	up
16	Op06g00114	2.009823194	8.20E-35	1.50E-30	48	8.9	yes	up
16	Op08g00322	1.987235796	1.71E-35	3.12E-31	37.3	5.3	yes	up
16	Op06g00348	1.983182867	2.01E-98	3.68E-94	33.3	1.5	yes	up
16	Op09g00915	1.964580214	1.44E-24	2.63E-20	74.7	29.3	yes	up
16	Op07g00407	1.949994013	1.35E-19	2.47E-15	24	3.9	yes	up
16	Op05g00264	1.94488833	2.56E-29	4.69E-25	44	8.4	yes	up
16	Op03g02251	1.931052587	4.41E-10	8.07E-06	46.7	21.3	yes	up
16	Op04g00122	1.92885117	1.48E-24	2.70E-20	64	20.9	yes	up
16	Op06g00351	1.903222738	1.24E-20	2.28E-16	33.3	6.8	yes	up
16	Op04g01205	1.899859876	7.15E-07	0.013095558	65.3	51.9	yes	up
16	Op05g00965	1.890207266	2.69E-20	4.93E-16	34.7	7.3	yes	up
16	Op06g01043	1.8874187	1.49E-89	2.72E-85	25.3	0.9	yes	up
16	Op09g00209	1.886095574	1.18E-08	0.000215493	77.3	73.3	yes	up
16	Op06g00456	1.880499513	5.71E-29	1.05E-24	14.7	1	yes	up
16	Op09g00862	1.874759655	1.90E-82	3.47E-78	25.3	1	yes	up
16	Op02g01166	1.87102086	2.46E-47	4.50E-43	21.3	1.3	yes	up
16	Op01g00299	1.866174508	6.97E-46	1.28E-41	29.3	2.6	yes	up
16	Op01g00766	1.860908289	5.52E-17	1.01E-12	65.3	29.3	yes	up
16	Op04g00369	1.847085511	5.55E-20	1.02E-15	37.3	8.6	yes	up
16	Op11g00334	1.842630067	9.57E-09	0.000175218	22.7	6.6	yes	up
16	Op02g00137	1.840846642	2.13E-155	3.90E-151	26.7	0.5	yes	up
16	Op04g01154	1.830080353	4.86E-223	8.89E-219	30.7	0.4	yes	up
16	Op10g00220	1.802059298	6.44E-23	1.18E-18	38.7	8	yes	up
16	Op06g00379	1.799726464	3.78E-08	0.000692702	57.3	37.6	yes	up
16	Op09g00671	1.794320961	1.18E-26	2.16E-22	28	3.9	yes	up
16	Op02g01122	1.788320819	1.06E-13	1.93E-09	45.3	17.3	yes	up
16	Op11g00923	1.770483786	6.85E-10	1.25E-05	29.3	9.5	yes	up
16	Op02g00382	1.749437643	3.69E-10	6.75E-06	24	6.5	yes	up
16	Op05g01553	1.746828626	9.34E-21	1.71E-16	24	3.7	yes	up
16	Op04g00933	1.744367256	2.65E-08	0.000485628	68	59.1	yes	up
16	Op08g01447	1.738441143	3.26E-43	5.96E-39	28	2.5	yes	up
16	Op04g01278	1.735218677	1.48E-21	2.70E-17	40	8.7	yes	up
16	Op02g02242	1.732017978	2.56E-13	4.68E-09	52	20.9	yes	up
16	Op02g02152	1.728813595	6.94E-20	1.27E-15	36	8.1	yes	up
16	Op01g00944	1.72766356	4.00E-15	7.32E-11	20	3.5	yes	up
16	Op09g00405	1.722614852	1.30E-06	0.023783488	52	34.4	yes	up
16	Op07g00200	1.710465888	1.64E-119	3.00E-115	25.3	0.6	yes	up
16	Op04g00113	1.704354926	5.81E-16	1.06E-11	32	7.8	yes	up
16	Op07g01948	1.689410927	2.79E-10	5.11E-06	65.3	43	yes	up
16	Op09g00091	1.689084187	1.62E-40	2.96E-36	36	4.3	yes	up
16	Op02g02189	1.684994934	1.79E-12	3.28E-08	25.3	6.1	yes	up
16	Op09g00993	1.68050389	1.06E-159	1.94E-155	20	0.2	yes	up
16	Op03g01883	1.678720968	6.52E-19	1.19E-14	21.3	3.2	yes	up

16	Op02g02186	1.675222141	3.76E-14	6.88E-10	49.3	17.5	yes	up
16	Op07g00725	1.675185972	1.39E-27	2.54E-23	28	3.8	yes	up
16	Op04g00231	1.673763163	5.26E-13	9.62E-09	53.3	22.7	yes	up
16	Op04g00401	1.651503068	1.39E-12	2.55E-08	42.7	16	yes	up
16	Op04g00811	1.638474423	1.00E-25	1.83E-21	28	4.1	yes	up
16	Op03g00673	1.638125025	2.81E-23	5.14E-19	26.7	4	yes	up
16	Op02g02190	1.63783245	4.68E-16	8.57E-12	21.3	3.7	yes	up
16	Op11g00934	1.629908858	1.11E-11	2.03E-07	30.7	9.2	yes	up
16	Op07g00341	1.628513885	3.68E-12	6.74E-08	26.7	7	yes	up
16	Op04g00781	1.620648884	7.91E-16	1.45E-11	20	3.3	yes	up
16	Op03g00145	1.617741025	2.61E-18	4.78E-14	48	14.8	yes	up
16	Op11g00080	1.615851378	5.37E-44	9.82E-40	16	0.8	yes	up
16	Op04g01638	1.60983774	3.29E-28	6.01E-24	24	2.8	yes	up
16	Op03g00115	1.609360643	2.41E-10	4.42E-06	34.7	12.4	yes	up
16	Op10g01012	1.607230402	1.87E-53	3.43E-49	26.7	1.8	yes	up
16	Op01g00409	1.606465183	9.32E-54	1.71E-49	17.3	0.7	yes	up
16	Op07g00557	1.60142173	9.68E-38	1.77E-33	18.7	1.3	yes	up
16	Op08g00029	1.593667927	2.94E-43	5.39E-39	29.3	2.7	yes	up
16	Op07g00573	1.592378194	3.05E-83	5.58E-79	14.7	0.3	yes	up
16	Op05g00566	1.588819291	2.33E-17	4.27E-13	24	4.3	yes	up
16	Op05g00632	1.588278708	2.41E-07	0.004407842	58.7	41.9	yes	up
16	Op04g00439	1.587687249	2.94E-13	5.37E-09	33.3	9.7	yes	up
16	Op05g01255	1.586846144	8.00E-12	1.46E-07	14.7	2.4	yes	up
16	Op01g02511	1.575748415	7.05E-13	1.29E-08	37.3	12.1	yes	up
16	Op06g00039	1.571039973	6.08E-31	1.11E-26	33.3	4.7	yes	up
16	Op06g00087	1.569255801	8.52E-22	1.56E-17	50.7	13.3	yes	up
16	Op09g00641	1.569098024	3.18E-20	5.81E-16	29.3	5.4	yes	up
16	Op02g01980	1.566089549	1.36E-19	2.49E-15	38.7	9.1	yes	up
16	Op01g00358	1.565182635	3.61E-14	6.60E-10	85.3	65.8	yes	up
16	Op01g02266	1.560203095	6.92E-15	1.27E-10	41.3	13.1	yes	up
16	Op07g01935	1.555346303	2.39E-139	4.38E-135	13.3	0.1	yes	up
16	Op07g00359	1.551931935	2.52E-16	4.62E-12	82.7	53.7	yes	up
16	Op02g00491	1.550683686	1.61E-10	2.95E-06	28	8.3	yes	up
16	Op11g01053	1.549268092	3.57E-20	6.54E-16	42.7	10.7	yes	up
16	Op09g01050	1.545398121	1.09E-26	1.99E-22	28	3.9	yes	up
16	Op03g01421	1.543844562	5.45E-07	0.009975444	24	8.3	yes	up
16	Op01g00341	1.541016751	5.72E-15	1.05E-10	30.7	7.4	yes	up
16	Op01g02321	1.540939111	2.27E-17	4.15E-13	24	4.2	yes	up
16	Op10g01473	1.538791404	1.25E-35	2.28E-31	21.3	1.7	yes	up
16	Op07g01716	1.534297527	1.02E-19	1.88E-15	25.3	4.2	yes	up
16	Op01g02162	1.53373561	2.70E-21	4.94E-17	32	6	yes	up
16	Op01g00281	1.526263296	9.44E-13	1.73E-08	38.7	13	yes	up
16	Op02g00702	1.525579575	2.01E-204	3.69E-200	20	0.2	yes	up
16	Op06g01221	1.516315233	3.45E-13	6.32E-09	37.3	11.1	yes	up
16	Op06g00658	1.503901143	1.99E-33	3.64E-29	30.7	3.7	yes	up
16	Op06g00806	1.502266069	7.53E-13	1.38E-08	30.7	8.3	yes	up
16	Op01g02322	1.501444589	2.11E-71	3.86E-67	17.3	0.5	yes	up
16	Op05g01154	1.49902825	1.39E-34	2.55E-30	26.7	2.7	yes	up
16	Op07g01302	1.498752327	8.11E-99	1.48E-94	20	0.5	yes	up
16	Op03g01769	1.49197901	2.47E-06	0.045175563	44	26.1	yes	up
16	Op01g01307	1.49187062	6.89E-12	1.26E-07	21.3	4.6	yes	up
16	Op08g01187	1.484130783	1.61E-10	2.94E-06	37.3	13.9	yes	up
16	Op06g00685	1.475590025	0	0	28	0.1	yes	up
16	Op11g00829	1.474562126	6.13E-08	0.00112131	21.3	6.4	yes	up
16	Op09g01292	1.47097908	1.51E-07	0.002761089	64	45.6	yes	up
16	Op01g00706	1.467774708	7.60E-216	1.39E-211	18.7	0.1	yes	up
16	Op03g00580	1.467138002	9.49E-18	1.74E-13	18.7	2.6	yes	up
16	Op10g00184	1.467061805	2.45E-08	0.000447761	57.3	32.8	yes	up
16	Op02g02070	1.466476464	1.64E-09	3.01E-05	21.3	5.7	yes	up
16	Op09g01443	1.46279445	3.24E-13	5.94E-09	21.3	4.3	yes	up
16	Op08g01205	1.462766432	4.84E-10	8.85E-06	58.7	36.6	yes	up
16	Op07g00120	1.459680424	1.80E-38	3.29E-34	18.7	1.2	yes	up

16	Op09g01571	1.457231135	2.34E-119	4.28E-115	20	0.4	yes	up
16	Op10g01549	1.436149922	1.57E-09	2.87E-05	45.3	21.5	yes	up
16	Op07g01742	1.433360418	2.38E-29	4.36E-25	32	4.5	yes	up
16	Op-scaf01g0008	1.433158708	5.12E-28	9.37E-24	22.7	2.5	yes	up
16	Op01g01999	1.431451244	1.48E-56	2.71E-52	29.3	2.1	yes	up
16	Op08g01423	1.4188516	1.97E-41	3.60E-37	21.3	1.5	yes	up
16	Op07g01814	1.418000749	3.84E-07	0.007038134	24	8.6	yes	up
16	Op11g00776	1.396616782	4.55E-176	8.32E-172	22.7	0.3	yes	up
16	Op02g01635	1.395014691	7.47E-19	1.37E-14	26.7	4.7	yes	up
16	Op01g02120	1.393069133	9.07E-10	1.66E-05	38.7	15.3	yes	up
16	Op02g00167	1.391622086	1.63E-12	2.98E-08	37.3	12	yes	up
16	Op04g01274	1.391310785	3.86E-37	7.06E-33	21.3	1.7	yes	up
16	Op05g01822	1.389464152	6.73E-12	1.23E-07	28	7.6	yes	up
16	Op03g01306	1.38936741	1.50E-08	0.00027481	50.7	26.3	yes	up
16	Op07g00322	1.386960683	8.57E-10	1.57E-05	44	19.3	yes	up
16	Op04g01511	1.383800151	1.20E-15	2.20E-11	22.7	4.2	yes	up
16	Op08g01657	1.381896221	1.77E-11	3.24E-07	28	7.6	yes	up
16	Op02g02255	1.369843684	1.24E-06	0.022788148	30.7	13.2	yes	up
16	Op07g01195	1.36979099	1.19E-06	0.02187278	36	16.8	yes	up
16	Op10g00245	1.369783791	2.19E-113	4.01E-109	10.7	0.1	yes	up
16	Op03g01444	1.369369951	1.48E-24	2.71E-20	20	2.2	yes	up
16	Op05g01159	1.367751043	5.46E-10	1.00E-05	69.3	45	yes	up
16	Op02g02244	1.364634678	7.27E-10	1.33E-05	62.7	36	yes	up
16	Op05g00526	1.363816149	6.71E-21	1.23E-16	22.7	3.2	yes	up
16	Op05g00906	1.359070407	7.88E-09	0.000144154	38.7	15.8	yes	up
16	Op05g01541	1.353300847	4.00E-12	7.32E-08	21.3	4.6	yes	up
16	Op04g00417	1.347061967	1.65E-180	3.03E-176	24	0.3	yes	up
16	Op03g01919	1.344649986	8.99E-13	1.65E-08	25.3	6.1	yes	up
16	Op02g01936	1.340067343	2.68E-12	4.90E-08	52	21.9	yes	up
16	Op04g00118	1.338671993	1.14E-13	2.08E-09	26.7	6.1	yes	up
16	Op06g00727	1.335206592	4.52E-62	8.28E-58	20	0.8	yes	up
16	Op02g00051	1.332844607	1.40E-09	2.56E-05	48	22.7	yes	up
16	Op07g01874	1.331298629	9.16E-09	0.000167645	29.3	10	yes	up
16	Op03g01368	1.32785429	2.69E-08	0.000493177	37.3	15.9	yes	up
16	Op01g02379	1.327290323	2.80E-12	5.13E-08	44	16.4	yes	up
16	Op05g00889	1.322221828	4.75E-35	8.70E-31	20	1.5	yes	up
16	Op06g00449	1.321703476	5.55E-13	1.02E-08	44	15.9	yes	up
16	Op02g00453	1.317160789	2.97E-08	0.000544529	34.7	14.5	yes	up
16	Op06g00696	1.314555076	1.94E-07	0.003554478	34.7	15.3	yes	up
16	Op05g01461	1.314521049	1.42E-06	0.025908581	20	6.6	yes	up
16	Op01g00134	1.313659705	8.89E-23	1.63E-18	14.7	1.3	yes	up
16	Op01g02219	1.298595619	1.59E-15	2.91E-11	26.7	5.5	yes	up
16	Op09g00344	1.295402837	3.10E-11	5.68E-07	50.7	21.9	yes	up
16	Op08g01547	1.294461802	2.15E-09	3.93E-05	36	13.9	yes	up
16	Op08g00163	1.292814125	2.43E-25	4.45E-21	20	2.1	yes	up
16	Op02g01358	1.290965364	1.31E-22	2.39E-18	26.7	4.1	yes	up
16	Op02g02149	1.290615123	4.06E-08	0.000743059	21.3	6.3	yes	up
16	Op06g01474	1.287953488	1.68E-07	0.003082284	25.3	8.9	yes	up
16	Op05g01473	1.287865351	2.01E-09	3.67E-05	28	9	yes	up
16	Op04g00074	1.286459548	7.41E-35	1.36E-30	18.7	1.4	yes	up
16	Op07g00533	1.282721088	3.58E-39	6.55E-35	24	2	yes	up
16	Op03g00093	1.281559982	4.39E-07	0.008041755	25.3	9.3	yes	up
16	Op09g00412	1.275670026	1.48E-07	0.002702607	34.7	15	yes	up
16	Op10g00324	1.271622109	6.97E-11	1.28E-06	30.7	9.4	yes	up
16	Op05g00173	1.27062085	1.66E-07	0.003044362	62.7	47	yes	up
16	Op07g00603	1.26868271	6.67E-07	0.012205769	37.3	18	yes	up
16	Op02g01106	1.254590551	4.15E-19	7.60E-15	17.3	2.1	yes	up
16	Op10g01007	1.247435607	3.07E-10	5.62E-06	26.7	7.8	yes	up
16	Op02g02296	1.24592302	1.80E-09	3.30E-05	20	5.1	yes	up
16	Op07g00353	1.244912612	1.38E-06	0.025215814	70.7	60.1	yes	up
16	Op09g00092	1.243971309	3.63E-11	6.65E-07	22.7	5.6	yes	up
16	Op04g00361	1.240050101	1.10E-237	2.02E-233	20	0.1	yes	up

16	Op07g01910	1.235283573	2.41E-06	0.044029424	36	17.4	yes	up
16	Op10g00836	1.23412748	6.29E-21	1.15E-16	20	2.5	yes	up
16	Op03g01226	1.234054749	2.43E-06	0.044496463	28	11.5	yes	up
16	Op02g00891	1.228943848	1.60E-06	0.029352129	37.3	18.2	yes	up
16	Op02g00962	1.227362655	1.16E-06	0.021309773	21.3	7.3	yes	up
16	Op01g00421	1.220740685	2.54E-294	4.65E-290	21.3	0.1	yes	up
16	Op02g01376	1.21970132	1.60E-17	2.93E-13	18.7	2.6	yes	up
16	Op04g01386	1.21343617	1.76E-06	0.032259033	20	6.6	yes	up
16	Op09g01096	1.212692358	6.00E-18	1.10E-13	22.7	3.7	yes	up
16	Op09g01173	1.209969023	1.70E-21	3.10E-17	21.3	2.8	yes	up
16	Op03g00097	1.206733161	5.72E-57	1.05E-52	17.3	0.7	yes	up
16	Op04g00424	1.202261943	8.97E-11	1.64E-06	13.3	2.2	yes	up
16	Op01g01342	1.196042805	2.58E-118	4.72E-114	21.3	0.4	yes	up
16	Op11g00192	1.194784634	8.81E-11	1.61E-06	29.3	8.6	yes	up
16	Op03g01157	1.194047863	7.82E-14	1.43E-09	14.7	2.1	yes	up
16	Op02g02145	1.19037228	1.83E-91	3.36E-87	17.3	0.4	yes	up
16	Op09g01102	1.182208221	1.01E-08	0.0001841	18.7	4.7	yes	up
16	Op03g02167	1.179829074	1.07E-14	1.95E-10	20	3.5	yes	up
16	Op01g00304	1.176878708	3.38E-15	6.19E-11	28	6.2	yes	up
16	Op07g00857	1.170151803	5.58E-09	0.000102189	34.7	12.3	yes	up
16	Op02g02220	1.168618524	2.09E-27	3.82E-23	10.7	0.6	yes	up
16	Op07g01906	1.167272425	8.02E-09	0.000146751	42.7	18.7	yes	up
16	Op07g01849	1.16666375	6.79E-43	1.24E-38	13.3	0.5	yes	up
16	Op08g01785	1.165613498	3.73E-15	6.83E-11	25.3	5.2	yes	up
16	Op06g01086	1.163307442	4.34E-08	0.000794729	21.3	6.4	yes	up
16	Op10g00691	1.160462326	3.96E-08	0.000725771	24	7.5	yes	up
16	Op01g02359	1.159583085	2.06E-07	0.003764927	42.7	20.7	yes	up
16	Op02g00248	1.159038323	1.23E-17	2.26E-13	14.7	1.7	yes	up
16	Op06g00747	1.154725574	1.20E-23	2.20E-19	24	3.2	yes	up
16	Op01g00481	1.149150683	1.47E-15	2.69E-11	16	2.2	yes	up
16	Op06g00383	1.148456199	1.43E-08	0.000260968	32	11.6	yes	up
16	Op02g02000	1.146256496	1.22E-19	2.24E-15	25.3	4.2	yes	up
16	Op09g01200	1.145690713	5.07E-12	9.28E-08	21.3	4.7	yes	up
16	Op06g00342	1.144017653	1.18E-25	2.17E-21	17.3	1.6	yes	up
16	Op10g00595	1.143106199	4.30E-26	7.87E-22	20	2.1	yes	up
16	Op03g02179	1.140711227	8.03E-07	0.014696407	37.3	17.4	yes	up
16	Op10g00199	1.138508527	4.31E-13	7.89E-09	21.3	4.2	yes	up
16	Op09g00240	1.13325158	2.17E-16	3.98E-12	13.3	1.5	yes	up
16	Op05g00607	1.132922451	4.01E-11	7.34E-07	18.7	3.9	yes	up
16	Op08g00815	1.129452788	2.07E-07	0.003782947	41.3	20.3	yes	up
16	Op06g01368	1.127113727	1.06E-23	1.94E-19	18.7	2	yes	up
16	Op08g01330	1.125230513	2.14E-15	3.92E-11	21.3	3.7	yes	up
16	Op11g00579	1.123849786	4.40E-20	8.05E-16	17.3	2	yes	up
16	Op10g01078	1.118047052	1.97E-08	0.000360022	33.3	12.9	yes	up
16	Op05g00089	1.115257044	6.35E-07	0.011628299	24	8.5	yes	up
16	Op01g01641	1.111737418	1.91E-06	0.034990269	26.7	10.6	yes	up
16	Op05g00835	1.111667889	1.84E-06	0.033600625	18.7	6	yes	up
16	Op10g00034	1.111024817	4.78E-07	0.008754978	44	23.9	yes	up
16	Op08g01682	1.105608205	1.11E-23	2.03E-19	17.3	1.7	yes	up
16	Op07g01899	1.102731904	6.56E-07	0.012012463	18.7	5.8	yes	up
16	Op06g00700	1.102329147	1.18E-20	2.15E-16	17.3	2	yes	up
16	Op09g00398	1.102169363	3.82E-23	6.99E-19	10.7	0.7	yes	up
16	Op03g00296	1.10145256	6.40E-10	1.17E-05	13.3	2.4	yes	up
16	Op08g01478	1.09916671	1.59E-06	0.02915797	18.7	6	yes	up
16	Op03g01232	1.094289233	1.15E-07	0.002114145	37.3	16.1	yes	up
16	Op01g00677	1.093452646	5.34E-07	0.009779511	70.7	50.9	yes	up
16	Op02g00412	1.093008134	2.70E-09	4.95E-05	30.7	10.3	yes	up
16	Op01g00707	1.091610141	7.95E-07	0.014560442	44	24	yes	up
16	Op02g02294	1.089582233	4.52E-26	8.28E-22	20	2.1	yes	up
16	Op02g00164	1.087765648	6.76E-07	0.012372228	76	67.3	yes	up
16	Op11g00773	1.087101541	8.92E-20	1.63E-15	18.7	2.3	yes	up
16	Op10g00060	1.086550047	3.95E-15	7.24E-11	24	4.7	yes	up

16	Op09g00912	1.084322455	4.51E-10	8.25E-06	20	4.8	yes	up
16	Op02g02034	1.077324327	7.27E-15	1.33E-10	16	2.3	yes	up
16	Op04g01621	1.063281423	1.73E-06	0.031656263	33.3	15.1	yes	up
16	Op03g00190	1.060304366	2.16E-20	3.95E-16	14.7	1.5	yes	up
16	Op10g00288	1.057127054	2.16E-08	0.000395791	66.7	42.8	yes	up
16	Op08g01438	1.057040075	2.30E-07	0.004203859	21.3	6.7	yes	up
16	Op01g01294	1.056600774	1.07E-28	1.97E-24	20	1.9	yes	up
16	Op10g01519	1.055113283	1.54E-20	2.82E-16	12	1	yes	up
16	Op05g01644	1.049094988	7.56E-15	1.38E-10	13.3	1.6	yes	up
16	Op07g01724	1.047176558	1.37E-07	0.002509138	29.3	11.1	yes	up
16	Op03g02181	1.045580649	9.67E-26	1.77E-21	16	1.4	yes	up
16	Op02g01832	1.04230007	6.24E-07	0.011421902	54.7	33.3	yes	up
16	Op09g00872	1.042200461	5.33E-22	9.76E-18	12	0.9	yes	up
16	Op03g00538	1.042100329	7.64E-126	1.40E-121	13.3	0.1	yes	up
16	Op01g00298	1.041103909	2.90E-11	5.31E-07	22.7	5.4	yes	up
16	Op08g01616	1.040935091	1.55E-06	0.028337544	20	6.5	yes	up
16	Op04g01572	1.040035853	8.57E-12	1.57E-07	29.3	7.9	yes	up
16	Op03g02057	1.034161407	1.52E-06	0.027905302	12	2.8	yes	up
16	Op01g00348	1.033328558	4.64E-30	8.50E-26	21.3	2	yes	up
16	Op09g00661	1.031508571	4.06E-08	0.00074228	12	2.4	yes	up
16	Op08g00918	1.031332688	1.37E-07	0.002505787	28	10.3	yes	up
16	Op06g01026	1.028340966	3.26E-31	5.96E-27	13.3	0.8	yes	up
16	Op01g02378	1.023369229	8.96E-09	0.000164091	14.7	3.1	yes	up
16	Op10g01326	1.023299262	9.69E-19	1.77E-14	12	1.1	yes	up
16	Op02g00482	1.021179484	2.11E-17	3.85E-13	16	2	yes	up
16	Op01g00312	1.019080907	3.02E-16	5.52E-12	25.3	4.9	yes	up
16	Op07g01956	1.01048997	1.25E-09	2.28E-05	20	4.9	yes	up
16	Op10g00631	1.006507915	4.52E-120	8.27E-116	13.3	0.1	yes	up
16	Op08g00012	1.003816014	8.35E-08	0.001528311	26.7	8.9	yes	up
16	Op01g00437	1.003077768	2.18E-07	0.003991062	44	20.3	yes	up
16	Op01g01970	1.000999763	6.00E-142	1.10E-137	10.7	0	yes	up
17	Op06g00665	8.334403522	8.28E-228	1.52E-223	91.7	5.4	yes	up
17	Op06g00213	7.48227767	1.50E-301	2.74E-297	87.5	3.4	yes	up
17	Op06g00664	6.827146043	0	0	83.3	1.4	yes	up
17	Op09g01028	5.342547005	0	0	73.6	0.5	yes	up
17	Op09g00713	4.950453335	0	0	61.1	0.4	yes	up
17	Op11g00027	4.795683182	4.93E-156	9.03E-152	26.4	0.5	yes	up
17	Op09g00714	4.661975916	0	0	54.2	0.5	yes	up
17	Op06g00211	4.517713594	0	0	73.6	0.5	yes	up
17	Op06g00165	4.445339554	0	0	79.2	0.3	yes	up
17	Op03g02142	4.444177396	0	0	80.6	2.5	yes	up
17	Op08g01626	4.375167617	0	0	72.2	0.3	yes	up
17	Op09g01323	4.341347848	0	0	77.8	0.3	yes	up
17	Op08g00877	4.246006925	0	0	58.3	0.4	yes	up
17	Op03g00614	4.225970832	8.99E-51	1.64E-46	86.1	25.2	yes	up
17	Op07g01885	4.216770414	0	0	54.2	0.3	yes	up
17	Op06g00386	3.828731025	0	0	40.3	0.2	yes	up
17	Op01g00326	3.682108446	2.66E-107	4.87E-103	73.6	7.3	yes	up
17	Op08g01720	3.641468877	1.11E-161	2.04E-157	44.4	1.5	yes	up
17	Op08g01640	3.602993219	2.90E-09	5.32E-05	26.4	8.5	yes	up
17	Op04g00011	3.531219538	0	0	54.2	0.8	yes	up
17	Op02g01760	3.490145984	0	0	47.2	0.2	yes	up
17	Op02g01868	3.457853475	3.03E-148	5.55E-144	34.7	1	yes	up
17	Op07g01671	3.367226687	5.59E-100	1.02E-95	88.9	12	yes	up
17	Op03g00444	3.359562506	4.25E-65	7.79E-61	27.8	1.6	yes	up
17	Op02g01290	3.312134261	4.49E-227	8.21E-223	22.2	0.2	yes	up
17	Op09g00862	3.299755041	1.09E-300	2.00E-296	48.6	0.8	yes	up
17	Op07g01139	3.271991826	1.09E-36	1.99E-32	61.1	14.5	yes	up
17	Op11g00303	3.228229757	3.90E-60	7.13E-56	22.2	1.1	yes	up
17	Op10g00340	3.214013402	4.66E-128	8.53E-124	15.3	0.2	yes	up
17	Op06g01403	3.193337481	0	0	37.5	0.2	yes	up
17	Op10g00012	3.16471734	0	0	66.7	1.1	yes	up

17	Op10g01246	3.131766425	0	0	61.1	1.3	yes	up
17	Op03g00052	3.118089678	0	0	48.6	0.6	yes	up
17	Op03g01152	3.087012473	0	0	44.4	0.1	yes	up
17	Op06g00319	3.038311994	0	0	69.4	1.6	yes	up
17	Op01g02032	3.033910344	0	0	66.7	0.8	yes	up
17	Op02g01831	3.004998729	1.30E-47	2.39E-43	56.9	9.2	yes	up
17	Op09g01442	2.987946487	9.16E-136	1.68E-131	51.4	2.5	yes	up
17	Op07g01666	2.932425109	3.66E-278	6.70E-274	48.6	0.9	yes	up
17	Op11g00387	2.892742857	6.60E-49	1.21E-44	44.4	5.5	yes	up
17	Op06g00188	2.814259343	1.05E-300	1.93E-296	19.4	0	yes	up
17	Op07g01926	2.76164833	4.22E-17	7.73E-13	52.8	18.3	yes	up
17	Op03g02002	2.746303104	1.08E-223	1.98E-219	54.2	1.6	yes	up
17	Op06g01216	2.734164531	2.68E-102	4.90E-98	68.1	6.2	yes	up
17	Op10g01552	2.734131835	6.13E-301	1.12E-296	19.4	0	yes	up
17	Op01g02155	2.702535919	0	0	40.3	0.5	yes	up
17	Op07g02011	2.691534015	1.37E-92	2.50E-88	23.6	0.7	yes	up
17	Op03g01691	2.630288678	5.23E-233	9.58E-229	27.8	0.3	yes	up
17	Op03g02065	2.61594559	0	0	34.7	0.1	yes	up
17	Op01g02227	2.611493842	2.31E-33	4.24E-29	47.2	8.4	yes	up
17	Op01g01551	2.594244931	4.79E-37	8.77E-33	15.3	0.8	yes	up
17	Op03g00747	2.577241596	1.90E-101	3.48E-97	43.1	2.4	yes	up
17	Op08g01118	2.571518632	8.86E-275	1.62E-270	18.1	0	yes	up
17	Op03g01747	2.552151218	0	0	43.1	0.2	yes	up
17	Op07g01175	2.548016424	7.65E-25	1.40E-20	33.3	5.8	yes	up
17	Op01g00600	2.539865276	0	0	41.7	0.2	yes	up
17	Op11g00430	2.52563871	3.95E-27	7.23E-23	16.7	1.4	yes	up
17	Op06g00638	2.522362801	0	0	56.9	0.6	yes	up
17	Op03g01723	2.476837047	0	0	25	0.1	yes	up
17	Op02g00660	2.474722694	0	0	41.7	0.1	yes	up
17	Op06g00235	2.454074967	1.47E-33	2.70E-29	61.1	14.9	yes	up
17	Op02g02154	2.453151367	9.69E-29	1.77E-24	72.2	24	yes	up
17	Op07g01886	2.443244588	0	0	25	0.1	yes	up
17	Op08g00514	2.439119882	1.08E-195	1.98E-191	43.1	1.1	yes	up
17	Op05g01555	2.431740672	0	0	38.9	0.3	yes	up
17	Op05g01643	2.412515629	1.49E-119	2.73E-115	29.2	0.9	yes	up
17	Op02g00363	2.40758822	2.23E-13	4.09E-09	93.1	84.2	yes	up
17	Op07g01316	2.370178683	1.28E-158	2.34E-154	16.7	0.1	yes	up
17	Op03g01827	2.352492549	0	0	40.3	0.1	yes	up
17	Op01g00986	2.349865125	1.66E-162	3.03E-158	13.9	0.1	yes	up
17	Op08g00622	2.348605629	0	0	27.8	0	yes	up
17	Op03g01746	2.347765917	0	0	48.6	0.1	yes	up
17	Op02g01573	2.333047467	2.49E-122	4.55E-118	36.1	1.3	yes	up
17	Op08g00106	2.319420485	3.04E-293	5.57E-289	40.3	0.5	yes	up
17	Op05g00643	2.308815592	0	0	45.8	0.5	yes	up
17	Op07g01601	2.289812183	2.33E-91	4.26E-87	23.6	0.7	yes	up
17	Op09g00214	2.279072933	0	0	34.7	0.3	yes	up
17	Op05g00577	2.274000961	1.49E-213	2.72E-209	37.5	0.7	yes	up
17	Op01g00279	2.261439466	2.00E-08	0.000366216	37.5	15.7	yes	up
17	Op07g01190	2.254160874	1.98E-68	3.62E-64	23.6	1	yes	up
17	Op05g01561	2.239949612	6.27E-20	1.15E-15	25	4	yes	up
17	Op05g00045	2.217344073	1.64E-28	3.01E-24	41.7	7.7	yes	up
17	Op01g00395	2.213705218	4.80E-22	8.79E-18	58.3	19.4	yes	up
17	Op01g02536	2.19597486	8.68E-176	1.59E-171	34.7	0.8	yes	up
17	Op06g01224	2.177746202	5.10E-221	9.33E-217	16.7	0.1	yes	up
17	Op10g00895	2.175408266	2.73E-259	4.99E-255	18.1	0.1	yes	up
17	Op06g00164	2.108106264	2.85E-79	5.21E-75	47.2	3.7	yes	up
17	Op09g00097	2.098871931	2.41E-139	4.42E-135	22.2	0.4	yes	up
17	Op03g00944	2.093239734	1.16E-125	2.12E-121	29.2	0.8	yes	up
17	Op06g01456	2.088629294	1.92E-24	3.52E-20	22.2	2.6	yes	up
17	Op02g00771	2.087381456	1.75E-13	3.21E-09	26.4	6.2	yes	up
17	Op03g02149	2.075709052	4.40E-21	8.06E-17	52.8	15.9	yes	up
17	Op01g00340	2.073280235	4.85E-179	8.87E-175	26.4	0.4	yes	up

17	Op01g00764	2.068894631	9.29E-86	1.70E-81	54.2	4.5	yes	up
17	Op06g00277	2.064424708	8.14E-48	1.49E-43	38.9	4.1	yes	up
17	Op01g01303	2.061326176	2.79E-18	5.10E-14	94.4	83.6	yes	up
17	Op05g00923	2.045834586	4.04E-286	7.40E-282	40.3	0.6	yes	up
17	Op09g00066	2.040263468	9.39E-36	1.72E-31	62.5	13.4	yes	up
17	Op03g00613	2.029743792	3.96E-218	7.25E-214	31.9	0.5	yes	up
17	Op11g00114	2.013042226	5.25E-234	9.60E-230	16.7	0.1	yes	up
17	Op03g01288	1.998682958	8.29E-07	0.015180526	26.4	10	yes	up
17	Op09g00518	1.981177986	0	0	31.9	0.3	yes	up
17	Op03g01468	1.967903951	3.13E-160	5.73E-156	37.5	1	yes	up
17	Op08g01330	1.957767691	6.38E-19	1.17E-14	23.6	3.7	yes	up
17	Op05g01576	1.936987112	6.97E-40	1.28E-35	25	2	yes	up
17	Op11g00559	1.934389712	0	0	25	0.1	yes	up
17	Op07g00545	1.934125882	3.04E-13	5.56E-09	22.2	4.5	yes	up
17	Op11g00597	1.926188491	0	0	29.2	0	yes	up
17	Op05g00799	1.919292077	6.49E-13	1.19E-08	68.1	36.5	yes	up
17	Op01g00358	1.91849111	3.20E-21	5.87E-17	93.1	65.7	yes	up
17	Op01g00219	1.917801125	7.62E-23	1.39E-18	43.1	9.4	yes	up
17	Op11g01407	1.902596291	5.89E-271	1.08E-266	22.2	0.1	yes	up
17	Op03g00242	1.901455046	6.16E-07	0.011283955	52.8	30.8	yes	up
17	Op08g01331	1.877582659	2.26E-72	4.13E-68	34.7	2.1	yes	up
17	Op05g01952	1.869617282	1.64E-98	3.01E-94	26.4	0.9	yes	up
17	Op07g01075	1.869194554	6.68E-282	1.22E-277	22.2	0.1	yes	up
17	Op03g02224	1.864478949	3.33E-275	6.09E-271	26.4	0.2	yes	up
17	Op03g01121	1.860634921	0	0	27.8	0.1	yes	up
17	Op05g00205	1.850642299	4.29E-100	7.85E-96	44.4	2.6	yes	up
17	Op11g00452	1.847570292	3.23E-25	5.91E-21	68.1	22.3	yes	up
17	Op09g01319	1.841669639	5.40E-195	9.89E-191	33.3	0.6	yes	up
17	Op01g01639	1.837997718	2.06E-259	3.77E-255	18.1	0.1	yes	up
17	Op05g00046	1.824805116	3.44E-136	6.30E-132	34.7	1.1	yes	up
17	Op06g00461	1.820882967	1.44E-128	2.64E-124	31.9	1	yes	up
17	Op08g01116	1.811333899	1.47E-162	2.70E-158	22.2	0.3	yes	up
17	Op09g00427	1.808219207	2.35E-06	0.043012045	20.8	7	yes	up
17	Op09g00867	1.80720888	1.03E-128	1.89E-124	25	0.5	yes	up
17	Op10g00288	1.800829349	8.00E-16	1.47E-11	76.4	42.7	yes	up
17	Op02g00725	1.79413423	3.06E-111	5.60E-107	43.1	2.1	yes	up
17	Op11g00827	1.766963276	0	0	22.2	0	yes	up
17	Op10g00722	1.759122061	2.98E-123	5.45E-119	16.7	0.2	yes	up
17	Op07g01876	1.758581884	6.58E-48	1.20E-43	38.9	4.1	yes	up
17	Op03g01717	1.747703794	4.26E-17	7.79E-13	36.1	8.9	yes	up
17	Op05g00149	1.738038952	5.77E-189	1.06E-184	16.7	0.1	yes	up
17	Op04g00312	1.728247231	1.14E-103	2.09E-99	40.3	2	yes	up
17	Op09g01367	1.728197515	1.70E-09	3.12E-05	26.4	7.8	yes	up
17	Op07g01020	1.716137569	5.28E-22	9.67E-18	61.1	19.9	yes	up
17	Op07g01014	1.715577044	1.13E-99	2.07E-95	40.3	2.1	yes	up
17	Op06g00639	1.706495937	9.53E-26	1.74E-21	93.1	55.2	yes	up
17	Op08g00591	1.698298808	7.05E-66	1.29E-61	40.3	3.1	yes	up
17	Op10g00639	1.685483109	7.24E-23	1.33E-18	44.4	10.2	yes	up
17	Op10g00842	1.677384062	1.27E-177	2.33E-173	11.1	0	yes	up
17	Op09g01549	1.666680318	2.44E-40	4.47E-36	31.9	3.3	yes	up
17	Op06g00372	1.662565307	0	0	30.6	0.1	yes	up
17	Op02g00885	1.652566217	1.52E-291	2.77E-287	26.4	0.2	yes	up
17	Op02g01335	1.65183564	9.88E-35	1.81E-30	38.9	5.6	yes	up
17	Op07g01365	1.634880707	4.25E-13	7.78E-09	68.1	36.2	yes	up
17	Op10g00120	1.631610206	2.27E-36	4.16E-32	18.1	1.2	yes	up
17	Op06g00162	1.631514115	9.29E-61	1.70E-56	31.9	2.2	yes	up
17	Op06g01361	1.621831214	7.01E-07	0.012826884	27.8	10.5	yes	up
17	Op05g00641	1.61840286	2.28E-45	4.18E-41	15.3	0.7	yes	up
17	Op02g02170	1.616293618	4.36E-07	0.007987804	30.6	11.7	yes	up
17	Op04g00063	1.609298975	4.74E-114	8.68E-110	37.5	1.5	yes	up
17	Op03g00610	1.595816016	3.01E-41	5.52E-37	27.8	2.4	yes	up
17	Op09g00793	1.571565055	1.75E-40	3.20E-36	11.1	0.4	yes	up

17	Op09g00739	1.570917197	3.74E-175	6.85E-171	23.6	0.3	yes	up
17	Op05g01687	1.56783107	6.39E-100	1.17E-95	22.2	0.6	yes	up
17	Op04g01573	1.564698801	1.50E-18	2.74E-14	22.2	3.3	yes	up
17	Op11g00922	1.562028755	7.36E-17	1.35E-12	30.6	6.4	yes	up
17	Op09g00837	1.550544096	2.58E-23	4.72E-19	23.6	3.1	yes	up
17	Op01g00124	1.55024086	2.66E-46	4.88E-42	27.8	2.2	yes	up
17	Op10g01234	1.547846423	1.17E-148	2.14E-144	19.4	0.2	yes	up
17	Op01g00793	1.547259473	2.42E-21	4.43E-17	48.6	12.6	yes	up
17	Op08g01351	1.540575147	2.65E-30	4.86E-26	18.1	1.4	yes	up
17	Op03g01267	1.521301114	2.98E-21	5.45E-17	29.2	5	yes	up
17	Op02g01907	1.519576587	1.98E-13	3.63E-09	45.8	16.4	yes	up
17	Op03g01857	1.517351686	1.14E-110	2.09E-106	11.1	0.1	yes	up
17	Op08g01310	1.512897451	3.12E-22	5.71E-18	30.6	5.2	yes	up
17	Op02g00900	1.502557975	3.64E-21	6.66E-17	38.9	8.3	yes	up
17	Op07g00004	1.496783446	5.89E-12	1.08E-07	25	6	yes	up
17	Op07g00458	1.495362833	2.31E-52	4.24E-48	30.6	2.3	yes	up
17	Op08g01342	1.49370959	2.17E-94	3.98E-90	15.3	0.3	yes	up
17	Op01g02006	1.478882929	3.50E-256	6.41E-252	23.6	0.2	yes	up
17	Op08g00477	1.474974593	2.22E-33	4.06E-29	37.5	5.2	yes	up
17	Op01g00199	1.466835479	2.24E-19	4.09E-15	13.9	1.3	yes	up
17	Op06g00078	1.460635863	1.80E-21	3.30E-17	23.6	3.3	yes	up
17	Op11g00652	1.458318855	1.43E-157	2.62E-153	20.8	0.3	yes	up
17	Op08g00590	1.456111979	1.96E-173	3.60E-169	31.9	0.6	yes	up
17	Op09g00498	1.455601003	7.10E-07	0.013003514	47.2	25.9	yes	up
17	Op07g00345	1.447889535	2.81E-227	5.14E-223	25	0.2	yes	up
17	Op02g01501	1.438362223	7.59E-27	1.39E-22	44.4	8.7	yes	up
17	Op11g00165	1.437593683	1.35E-76	2.47E-72	12.5	0.2	yes	up
17	Op01g00549	1.43354854	2.51E-12	4.60E-08	56.9	24.5	yes	up
17	Op11g01283	1.420678974	1.16E-35	2.13E-31	29.2	3	yes	up
17	Op07g01545	1.413854387	2.85E-08	0.000521209	55.6	29.8	yes	up
17	Op04g01557	1.410319363	2.31E-307	4.23E-303	25	0.1	yes	up
17	Op08g01146	1.409017407	3.70E-27	6.78E-23	36.1	5.9	yes	up
17	Op03g01151	1.408844421	7.50E-148	1.37E-143	11.1	0	yes	up
17	Op01g01121	1.408844256	5.58E-79	1.02E-74	34.7	2	yes	up
17	Op09g00288	1.406222772	7.23E-70	1.32E-65	31.9	1.9	yes	up
17	Op03g01458	1.403551451	1.12E-10	2.05E-06	75	51.8	yes	up
17	Op09g01172	1.400858598	2.46E-10	4.51E-06	54.2	25.9	yes	up
17	Op03g00665	1.398999696	1.70E-60	3.11E-56	15.3	0.5	yes	up
17	Op02g00441	1.398535117	2.45E-22	4.49E-18	20.8	2.5	yes	up
17	Op07g00885	1.397352722	4.18E-42	7.65E-38	26.4	2.1	yes	up
17	Op06g00116	1.392073135	0	0	29.2	0.1	yes	up
17	Op04g00651	1.386818458	3.53E-117	6.47E-113	22.2	0.5	yes	up
17	Op07g01294	1.385599735	1.28E-13	2.34E-09	80.6	49	yes	up
17	Op10g01042	1.38508094	1.75E-11	3.21E-07	48.6	19.7	yes	up
17	Op04g01586	1.382461726	1.40E-24	2.57E-20	20.8	2.3	yes	up
17	Op10g01051	1.380918703	5.14E-140	9.41E-136	23.6	0.4	yes	up
17	Op02g00068	1.375910685	3.96E-17	7.24E-13	31.9	6.8	yes	up
17	Op01g00758	1.374309197	6.89E-19	1.26E-14	13.9	1.4	yes	up
17	Op06g00163	1.372478531	1.45E-123	2.65E-119	22.2	0.4	yes	up
17	Op05g01945	1.370947913	2.09E-34	3.82E-30	30.6	3.5	yes	up
17	Op03g01811	1.369315271	3.19E-12	5.85E-08	72.2	45.1	yes	up
17	Op03g02167	1.367505137	1.79E-38	3.28E-34	31.9	3.4	yes	up
17	Op01g00221	1.364127211	1.11E-38	2.03E-34	29.2	2.8	yes	up
17	Op01g00525	1.364088319	3.06E-50	5.61E-46	22.2	1.3	yes	up
17	Op09g00571	1.359683741	1.97E-36	3.60E-32	36.1	4.5	yes	up
17	Op01g01626	1.359573386	3.21E-15	5.88E-11	40.3	11.3	yes	up
17	Op02g01746	1.359263313	1.85E-197	3.38E-193	11.1	0	yes	up
17	Op01g00312	1.353418656	6.81E-29	1.25E-24	33.3	4.8	yes	up
17	Op04g00410	1.352147969	1.07E-06	0.019508877	26.4	9.8	yes	up
17	Op05g01112	1.345953005	3.98E-254	7.28E-250	30.6	0.3	yes	up
17	Op02g01745	1.338869691	2.88E-222	5.28E-218	11.1	0	yes	up
17	Op03g01748	1.337293268	8.08E-100	1.48E-95	22.2	0.6	yes	up

17	Op09g00522	1.33622646	7.60E-13	1.39E-08	31.9	8.5	yes	up
17	Op01g02487	1.335933644	7.75E-08	0.001417866	40.3	18.5	yes	up
17	Op02g00421	1.335624304	3.72E-118	6.80E-114	18.1	0.3	yes	up
17	Op02g01871	1.335106083	1.48E-96	2.70E-92	27.8	1	yes	up
17	Op09g00547	1.334218857	2.72E-22	4.98E-18	20.8	2.5	yes	up
17	Op09g00123	1.334067516	1.58E-185	2.89E-181	15.3	0.1	yes	up
17	Op05g00891	1.316852058	1.91E-19	3.50E-15	31.9	6.1	yes	up
17	Op04g00209	1.31494057	4.52E-160	8.28E-156	12.5	0.1	yes	up
17	Op01g00688	1.31341294	1.10E-19	2.01E-15	45.8	11.7	yes	up
17	Op02g02307	1.310567412	3.07E-21	5.62E-17	45.8	10.7	yes	up
17	Op07g01173	1.308286559	9.61E-18	1.76E-13	31.9	6.6	yes	up
17	Op05g00273	1.302565833	3.36E-10	6.15E-06	19.4	4.3	yes	up
17	Op03g00333	1.294235496	4.47E-55	8.19E-51	27.8	1.8	yes	up
17	Op06g00705	1.293260761	4.58E-23	8.38E-19	26.4	3.8	yes	up
17	Op11g00924	1.285991339	2.05E-18	3.75E-14	34.7	7.5	yes	up
17	Op05g00272	1.283662828	3.87E-16	7.08E-12	48.6	15.2	yes	up
17	Op02g00453	1.281661919	6.21E-17	1.14E-12	47.2	14.4	yes	up
17	Op09g00136	1.2780235	5.67E-10	1.04E-05	68.1	43.5	yes	up
17	Op07g01838	1.274784835	2.80E-43	5.13E-39	18.1	1	yes	up
17	Op07g01747	1.263592756	3.95E-158	7.23E-154	16.7	0.1	yes	up
17	Op02g01593	1.25772026	2.52E-09	4.61E-05	41.7	16.5	yes	up
17	Op06g00362	1.254463141	7.41E-141	1.36E-136	19.4	0.3	yes	up
17	Op10g00473	1.245594474	3.32E-23	6.08E-19	30.6	4.9	yes	up
17	Op07g01041	1.242566309	1.07E-11	1.96E-07	87.5	67.9	yes	up
17	Op05g00609	1.237936292	7.28E-18	1.33E-13	65.3	24.4	yes	up
17	Op05g01314	1.228684483	3.92E-07	0.007175398	36.1	15.2	yes	up
17	Op10g00620	1.228330165	2.64E-25	4.83E-21	22.2	2.5	yes	up
17	Op01g00474	1.221875218	5.33E-84	9.75E-80	11.1	0.1	yes	up
17	Op06g00351	1.217639798	2.01E-07	0.003672767	22.2	6.9	yes	up
17	Op05g00863	1.215360058	2.50E-10	4.58E-06	40.3	14.7	yes	up
17	Op08g01518	1.21506544	1.20E-49	2.20E-45	15.3	0.6	yes	up
17	Op04g00934	1.21026765	8.79E-17	1.61E-12	34.7	8	yes	up
17	Op11g00173	1.207114425	6.50E-305	1.19E-300	15.3	0	yes	up
17	Op10g00494	1.204680025	2.31E-15	4.22E-11	44.4	13.5	yes	up
17	Op04g01380	1.203025594	5.57E-14	1.02E-09	22.2	4.3	yes	up
17	Op11g00190	1.199852913	0	0	29.2	0.2	yes	up
17	Op10g00178	1.192813561	3.40E-13	6.22E-09	23.6	5	yes	up
17	Op07g01836	1.185709596	1.00E-25	1.84E-21	26.4	3.4	yes	up
17	Op07g01702	1.184272252	1.98E-27	3.63E-23	23.6	2.6	yes	up
17	Op01g00437	1.184225969	8.60E-09	0.000157454	45.8	20.2	yes	up
17	Op07g01177	1.182860885	9.76E-36	1.79E-31	26.4	2.5	yes	up
17	Op04g00571	1.178456339	1.96E-95	3.58E-91	16.7	0.3	yes	up
17	Op03g00834	1.17597573	1.05E-64	1.91E-60	22.2	1	yes	up
17	Op03g00570	1.166086625	2.92E-07	0.005353501	34.7	14.1	yes	up
17	Op07g01762	1.155008885	3.34E-16	6.12E-12	41.7	11.4	yes	up
17	Op01g01625	1.149889275	7.11E-12	1.30E-07	48.6	18.8	yes	up
17	Op03g01600	1.148388085	2.63E-55	4.82E-51	25	1.4	yes	up
17	Op04g00025	1.148371632	8.30E-110	1.52E-105	13.9	0.2	yes	up
17	Op01g00759	1.14069841	1.44E-44	2.63E-40	15.3	0.7	yes	up
17	Op04g01478	1.1362892	4.54E-07	0.008313147	33.3	14.1	yes	up
17	Op07g01148	1.129200931	8.54E-09	0.000156343	75	52.5	yes	up
17	Op02g01346	1.122016014	4.38E-178	8.01E-174	26.4	0.4	yes	up
17	Op01g00540	1.116540358	3.91E-07	0.007164109	44.4	21.1	yes	up
17	Op05g01788	1.114400664	1.13E-22	2.07E-18	27.8	4.1	yes	up
17	Op05g00877	1.112977508	2.72E-32	4.98E-28	16.7	1.1	yes	up
17	Op09g01389	1.109740302	1.71E-30	3.12E-26	31.9	4	yes	up
17	Op09g00074	1.104722139	1.50E-165	2.74E-161	18.1	0.2	yes	up
17	Op01g02034	1.104240982	7.18E-44	1.31E-39	20.8	1.3	yes	up
17	Op03g02344	1.104099607	1.23E-12	2.25E-08	25	5.8	yes	up
17	Op03g00608	1.102135377	3.64E-82	6.67E-78	12.5	0.2	yes	up
17	Op06g00067	1.09744489	9.81E-87	1.80E-82	15.3	0.3	yes	up
17	Op01g01886	1.096197582	2.36E-11	4.31E-07	44.4	15.9	yes	up

17	Op01g00171	1.09603939	6.42E-121	1.18E-116	19.4	0.3	yes	up
17	Op05g00494	1.095625339	1.93E-06	0.035305433	15.3	4.1	yes	up
17	Op07g01462	1.095163566	9.59E-12	1.76E-07	38.9	12.4	yes	up
17	Op01g00058	1.092279661	2.77E-16	5.07E-12	38.9	9.8	yes	up
17	Op10g00272	1.091688907	2.97E-45	5.44E-41	22.2	1.4	yes	up
17	Op11g00952	1.09163038	2.77E-13	5.07E-09	27.8	6.4	yes	up
17	Op04g00933	1.091294529	2.37E-11	4.34E-07	81.9	59	yes	up
17	Op02g00778	1.081236724	1.32E-15	2.42E-11	18.1	2.6	yes	up
17	Op01g00142	1.080406263	1.01E-36	1.85E-32	18.1	1.2	yes	up
17	Op08g01466	1.079641398	4.34E-08	0.000793922	12.5	2.5	yes	up
17	Op11g00925	1.072778666	6.35E-189	1.16E-184	16.7	0.1	yes	up
17	Op05g01768	1.07228534	4.64E-13	8.49E-09	30.6	8	yes	up
17	Op04g00335	1.071544893	2.86E-17	5.24E-13	26.4	4.8	yes	up
17	Op06g00918	1.065049691	1.46E-07	0.002669177	19.4	5.5	yes	up
17	Op09g01397	1.062231054	3.08E-11	5.63E-07	25	6.1	yes	up
17	Op07g01776	1.054287818	6.66E-08	0.001218401	36.1	14.2	yes	up
17	Op01g01211	1.045577857	2.32E-277	4.25E-273	13.9	0	yes	up
17	Op09g01507	1.043601575	1.33E-09	2.43E-05	58.3	27.6	yes	up
17	Op04g00407	1.043521488	2.15E-08	0.000394074	44.4	20	yes	up
17	Op05g00986	1.040689023	1.04E-54	1.91E-50	11.1	0.3	yes	up
17	Op01g00663	1.035672867	4.96E-15	9.09E-11	44.4	13.1	yes	up
17	Op11g01292	1.033576864	4.24E-18	7.75E-14	29.2	5.4	yes	up
17	Op07g00230	1.024630435	1.53E-07	0.002793411	56.9	32.2	yes	up
17	Op10g01490	1.02245271	5.78E-128	1.06E-123	18.1	0.3	yes	up
17	Op06g00661	1.021519078	2.24E-184	4.11E-180	13.9	0.1	yes	up
17	Op05g01745	1.019411207	1.64E-198	3.00E-194	16.7	0.1	yes	up
17	Op07g01434	1.018851489	1.15E-07	0.002096241	36.1	14.2	yes	up
17	Op02g02064	1.018469626	2.03E-10	3.72E-06	43.1	15.7	yes	up
17	Op05g00970	1.01566632	1.28E-18	2.34E-14	25	4.1	yes	up
17	Op04g01154	1.015246717	4.89E-52	8.96E-48	15.3	0.6	yes	up
17	Op01g01950	1.012502089	1.60E-15	2.93E-11	29.2	6.2	yes	up
17	Op03g00106	1.011445039	2.34E-10	4.28E-06	81.9	55	yes	up
17	Op02g01096	1.009786509	2.82E-16	5.16E-12	16.7	2.2	yes	up
17	Op07g01988	1.007971519	2.37E-154	4.34E-150	18.1	0.2	yes	up
17	Op03g00443	1.007429763	3.90E-16	7.14E-12	26.4	5.1	yes	up
17	Op01g02171	1.006927311	4.72E-09	8.64E-05	27.8	8.5	yes	up
17	Op04g00580	1.005581812	1.01E-29	1.85E-25	25	2.7	yes	up
17	Op07g00359	1.005298213	2.50E-08	0.000457398	79.2	53.8	yes	up
17	Op11g00659	1.005176813	1.39E-62	2.55E-58	19.4	0.8	yes	up
18	Op08g00567	6.910589528	0	0	100	1.6	yes	up
18	Op10g01125	6.029581639	0	0	97	1.3	yes	up
18	Op08g00869	5.209824727	0	0	90.9	0.9	yes	up
18	Op03g00701	5.023046625	0	0	81.8	0.3	yes	up
18	Op02g01327	4.489241746	0	0	78.8	0.4	yes	up
18	Op08g01052	4.432990197	0	0	81.8	0.5	yes	up
18	Op08g00568	4.189229059	0	0	90.9	0.6	yes	up
18	Op07g00255	4.010202772	0	0	78.8	0.3	yes	up
18	Op10g01528	3.934854894	0	0	72.7	0.4	yes	up
18	Op01g01713	3.838022136	0	0	78.8	0.4	yes	up
18	Op08g00442	3.826599528	0	0	84.8	0.4	yes	up
18	Op04g01184	3.592104511	0	0	60.6	0.3	yes	up
18	Op03g00704	3.568100017	0	0	75.8	0.6	yes	up
18	Op02g00858	3.55699869	0	0	63.6	0.3	yes	up
18	Op08g01051	3.437729614	0	0	57.6	0.1	yes	up
18	Op07g00729	3.383020441	0	0	60.6	0.2	yes	up
18	Op08g00447	3.258764168	1.38E-254	2.52E-250	39.4	0.3	yes	up
18	Op08g00462	3.241946148	0	0	63.6	0.3	yes	up
18	Op10g01124	3.227485355	0	0	63.6	0.2	yes	up
18	Op06g00418	3.20347673	0	0	51.5	0.2	yes	up
18	Op10g01122	2.967642833	0	0	57.6	0.1	yes	up
18	Op09g00318	2.95972516	0	0	33.3	0.1	yes	up
18	Op01g01794	2.896767649	0	0	51.5	0.1	yes	up

18	Op08g00039	2.642569153	1.36E-26	2.50E-22	42.4	4.6	yes	up
18	Op02g00377	2.587931358	0	0	39.4	0	yes	up
18	Op04g01345	2.388528301	0	0	36.4	0	yes	up
18	Op08g00121	2.36231733	0	0	24.2	0	yes	up
18	Op04g01185	2.3114413	0	0	45.5	0.1	yes	up
18	Op11g01196	2.284535043	2.58E-137	4.72E-133	33.3	0.5	yes	up
18	Op04g00322	2.194822552	3.38E-261	6.18E-257	39.4	0.3	yes	up
18	Op02g02117	2.187768576	1.83E-95	3.35E-91	21.2	0.3	yes	up
18	Op07g00939	2.166912859	0	0	36.4	0.1	yes	up
18	Op08g00120	2.127297509	0	0	33.3	0.1	yes	up
18	Op04g01018	2.080732359	0	0	42.4	0	yes	up
18	Op05g01504	2.069194844	1.45E-74	2.65E-70	27.3	0.6	yes	up
18	Op09g00089	1.939937928	6.78E-75	1.24E-70	30.3	0.8	yes	up
18	Op03g00209	1.877694893	1.54E-304	2.82E-300	33.3	0.1	yes	up
18	Op08g00507	1.868632209	0	0	30.3	0	yes	up
18	Op05g01086	1.782408379	9.50E-258	1.74E-253	27.3	0.1	yes	up
18	Op08g01727	1.780441222	2.06E-158	3.76E-154	30.3	0.3	yes	up
18	Op04g00545	1.740153337	5.61E-15	1.03E-10	30.3	4	yes	up
18	Op05g00635	1.647576978	8.05E-272	1.47E-267	27.3	0.1	yes	up
18	Op08g00044	1.632433816	1.13E-296	2.07E-292	21.2	0	yes	up
18	Op03g00651	1.596469184	2.47E-218	4.52E-214	18.2	0	yes	up
18	Op03g00921	1.584092822	1.37E-18	2.51E-14	18.2	1.2	yes	up
18	Op05g01421	1.58360198	2.36E-124	4.32E-120	24.2	0.3	yes	up
18	Op05g00922	1.55244707	1.65E-08	0.000302363	39.4	10.8	yes	up
18	Op04g00661	1.541283861	1.84E-07	0.003374621	24.2	5	yes	up
18	Op05g01754	1.51391471	2.21E-09	4.04E-05	30.3	6.2	yes	up
18	Op11g01167	1.436901678	4.11E-228	7.53E-224	21.2	0.1	yes	up
18	Op05g00374	1.415831976	2.34E-09	4.28E-05	30.3	6.1	yes	up
18	Op04g01628	1.400917366	4.75E-09	8.69E-05	18.2	2.5	yes	up
18	Op06g00419	1.370007958	0	0	24.2	0	yes	up
18	Op01g00778	1.340060279	2.66E-218	4.87E-214	18.2	0	yes	up
18	Op02g01831	1.338667111	1.32E-09	2.42E-05	39.4	9.5	yes	up
18	Op08g00144	1.301255122	0	0	18.2	0	yes	up
18	Op05g01345	1.280302023	1.02E-38	1.87E-34	24.2	1	yes	up
18	Op02g02115	1.25133871	6.81E-217	1.25E-212	15.2	0	yes	up
18	Op09g00810	1.194036862	3.24E-08	0.000593057	30.3	6.6	yes	up
18	Op04g00935	1.175668053	3.91E-14	7.15E-10	18.2	1.6	yes	up
18	Op08g00241	1.170426934	8.01E-12	1.47E-07	21.2	2.5	yes	up
18	Op01g00651	1.121628166	5.44E-253	9.96E-249	15.2	0	yes	up
18	Op05g01441	1.120515254	6.75E-09	0.000123587	33.3	7.3	yes	up
18	Op02g00792	1.113156989	4.17E-75	7.64E-71	18.2	0.3	yes	up
18	Op10g00488	1.110507535	4.69E-11	8.59E-07	21.2	2.6	yes	up
18	Op04g00294	1.058004108	8.08E-07	0.014792028	15.2	2.3	yes	up
18	Op03g01085	1.053502286	3.43E-92	6.28E-88	21.2	0.3	yes	up
18	Op10g01527	1.050780648	3.84E-28	7.03E-24	15.2	0.5	yes	up
18	Op01g01793	1.031291289	2.37E-162	4.34E-158	12.1	0	yes	up

Table S5. Marker genes from *Arabidopsis thaliana* and *Catharanthus roseus* leaves.

	Species name	Query_ID	Cell type	OpGene id
1	Arabidopsis thaliana	AT4G23670	Epidermal cell	Op06g01447
2	Arabidopsis thaliana	AT3G51600	Epidermal cell	Op03g00450
3	Arabidopsis thaliana	AT2G27385	Epidermal cell	Op07g01208
4	Arabidopsis thaliana	AT2G38540	Epidermal cell	Op08g00760
5	Arabidopsis thaliana	AT1G09310	Epidermal cell	Op03g01424
6	Arabidopsis thaliana	AT1G68530	Epidermal cell	Op05g01775
7	Arabidopsis thaliana	AT4G21960	Epidermal cell	Op02g01804
8	Arabidopsis thaliana	AT5G44020	Epidermal cell	Op11g00630
9	Arabidopsis thaliana	AT1G29670	Epidermal cell	Op02g01322
10	Arabidopsis thaliana	AT1G78850	Epidermal cell	Op02g01426
11	Arabidopsis thaliana	AT4G21620	Epidermal cell	Op02g01635
12	Arabidopsis thaliana	AT2G26250	Epidermal cell	Op04g01278
13	Arabidopsis thaliana	AT1G01120	Epidermal cell	Op03g00063
14	Arabidopsis thaliana	AT3G16370	Epidermal cell	Op08g01617
15	Arabidopsis thaliana	AT3G24420	Epidermal cell	Op01g02127
16	Arabidopsis thaliana	AT4G38770	Epidermal cell	Op03g02227
17	Arabidopsis thaliana	AT2G28630	Epidermal cell	Op07g00407
18	Arabidopsis thaliana	AT1G51500	Epidermal cell	Op04g01040
19	Arabidopsis thaliana	AT3G26450	Epidermal cell	Op06g01447
20	Arabidopsis thaliana	AT4G01950	Epidermal cell	Op03g01421
21	Arabidopsis thaliana	AT5G43760	Epidermal cell	Op11g00482
22	Arabidopsis thaliana	AT1G48750	Epidermal cell	Op05g01806
23	Arabidopsis thaliana	AT1G21130	Epidermal cell	Op02g02331
24	Arabidopsis thaliana	AT5G40450	Epidermal cell	Op05g00156
25	Arabidopsis thaliana	AT5G62470	Epidermal cell	Op08g00195
26	Arabidopsis thaliana	AT1G29930	Mesophyll cell	Op02g01246
27	Arabidopsis thaliana	ATCG00490	Mesophyll cell	Op03g01010
28	Arabidopsis thaliana	AT2G34430	Mesophyll cell	Op02g01229
29	Arabidopsis thaliana	AT2G39730	Mesophyll cell	Op02g00118
30	Arabidopsis thaliana	AT1G61520	Mesophyll cell	Op10g00695
31	Arabidopsis thaliana	AT5G54270	Mesophyll cell	Op10g00414
32	Arabidopsis thaliana	AT2G06520	Mesophyll cell	Op01g01882
33	Arabidopsis thaliana	AT1G29910	Mesophyll cell	Op02g01246
34	Arabidopsis thaliana	AT2G34420	Mesophyll cell	Op02g01246
35	Arabidopsis thaliana	AT1G08380	Mesophyll cell	Op07g01194
36	Arabidopsis thaliana	AT1G55670	Mesophyll cell	Op10g00096
37	Arabidopsis thaliana	AT3G61470	Mesophyll cell	Op06g00093
38	Arabidopsis thaliana	AT3G47470	Mesophyll cell	Op08g00184
39	Arabidopsis thaliana	AT3G08940	Mesophyll cell	Op01g00153
40	Arabidopsis thaliana	AT3G54890	Mesophyll cell	Op02g00520
41	Arabidopsis thaliana	AT5G66570	Mesophyll cell	Op05g00519
42	Arabidopsis thaliana	AT1G79040	Mesophyll cell	Op10g00061
43	Arabidopsis thaliana	AT1G67090	Mesophyll cell	Op05g01780
44	Arabidopsis thaliana	AT1G29920	Mesophyll cell	Op02g01246
45	Arabidopsis thaliana	AT4G05180	Mesophyll cell	Op02g01733
46	Arabidopsis thaliana	AT4G12800	Mesophyll cell	Op07g01420
47	Arabidopsis thaliana	AT4G10340	Mesophyll cell	Op09g01427
48	Arabidopsis thaliana	AT4G28750	Mesophyll cell	Op07g01580
49	Arabidopsis thaliana	AT3G28455	Vascular cell	Op06g00362
50	Arabidopsis thaliana	AT1G07640	Vascular cell	Op07g00352
51	Arabidopsis thaliana	AT4G15110	Vascular cell	Op10g01583
52	Arabidopsis thaliana	AT2G24762	Vascular cell	Op01g00903
53	Arabidopsis thaliana	AT1G80760	Vascular cell	Op08g01358

54	Arabidopsis thaliana	AT5G57130	Vascular cell	Op01g01321
55	Arabidopsis thaliana	AT1G77110	Vascular cell	Op08g01212
56	Arabidopsis thaliana	AT5G47720	Vascular cell	Op10g00950
57	Arabidopsis thaliana	AT3G18280	Vascular cell	Op06g01554
58	Arabidopsis thaliana	AT5G19260	Vascular cell	Op11g00354
59	Arabidopsis thaliana	AT1G06490	Vascular cell	Op10g00722
60	Arabidopsis thaliana	AT3G05330	Vascular cell	Op03g01260
61	Arabidopsis thaliana	AT1G61660	Vascular cell	Op02g01712
62	Arabidopsis thaliana	AT1G64625	Vascular cell	Op07g01163
63	Catharanthus roseus	EVM0005631	Mesophyll cell	Op05g01165
64	Catharanthus roseus	EVM0012653	Mesophyll cell	Op05g01199
65	Catharanthus roseus	EVM0011613	Mesophyll cell	Op05g01780
66	Catharanthus roseus	EVM0013845	Mesophyll cell	Op10g00972
67	Catharanthus roseus	EVM0017264	Mesophyll cell	Op10g01291
68	Catharanthus roseus	EVM0030212	Mesophyll cell	Op05g00187
69	Catharanthus roseus	EVM0006721	Mesophyll cell	Op02g00118
70	Catharanthus roseus	EVM0029598	Epidermal cell	Op02g01767
71	Catharanthus roseus	EVM0034768	Epidermal cell	Op04g00699
72	Catharanthus roseus	EVM0026652	Epidermal cell	Op03g00598
73	Catharanthus roseus	EVM0009371	Epidermal cell	Op01g01135
74	Catharanthus roseus	EVM0025066	Epidermal cell	Op01g00944
75	Catharanthus roseus	EVM0012121	Epidermal cell	Op02g00382
76	Catharanthus roseus	EVM0020471	Vascular cell	Op09g01082
77	Catharanthus roseus	EVM0033622	Vascular cell	Op07g00458
78	Catharanthus roseus	EVM0001919	Vascular cell	Op10g00012
79	Catharanthus roseus	EVM0034564	Vascular cell	Op11g00191

Table S6. Marker genes of two types of Mesophyll cell from *Arabidopsis thaliana* leaves.

	Query_ID	Cell type	OpGene_ID
1	AT1G05010	Spongy Mesophyll cell	Op02g00107
2	AT1G05560	Spongy Mesophyll cell	Op11g00881
3	AT1G07000	Spongy Mesophyll cell	Op07g00126
4	AT1G09070	Spongy Mesophyll cell	Op03g01288
5	AT1G12610	Spongy Mesophyll cell	Op04g00320
6	AT1G15670	Spongy Mesophyll cell	Op08g01500
7	AT1G19020	Spongy Mesophyll cell	Op06g00341
8	AT1G20440	Spongy Mesophyll cell	Op09g00066
9	AT1G20823	Spongy Mesophyll cell	Op05g01454
10	AT1G22710	Spongy Mesophyll cell	Op09g01082
11	AT1G53210	Spongy Mesophyll cell	Op10g00545
12	AT1G54100	Spongy Mesophyll cell	Op08g01731
13	AT1G59910	Spongy Mesophyll cell	Op06g00348
14	AT1G69490	Spongy Mesophyll cell	Op06g00476
15	AT1G72520	Spongy Mesophyll cell	Op08g01726
16	AT1G80840	Spongy Mesophyll cell	Op08g01349
17	AT2G17220	Spongy Mesophyll cell	Op03g02332
18	AT2G25460	Spongy Mesophyll cell	Op04g01647
19	AT2G26580	Spongy Mesophyll cell	Op01g02227
20	AT2G45190	Spongy Mesophyll cell	Op03g00368
21	AT3G11930	Spongy Mesophyll cell	Op01g00743
22	AT3G14990	Spongy Mesophyll cell	Op10g00564
23	AT3G48740	Spongy Mesophyll cell	Op08g00883
24	AT3G55430	Spongy Mesophyll cell	Op02g00147
25	AT4G00180	Spongy Mesophyll cell	Op03g00368
26	AT4G02380	Spongy Mesophyll cell	Op07g01948
27	AT4G02520	Spongy Mesophyll cell	Op07g01910
28	AT4G24380	Spongy Mesophyll cell	Op09g01525
29	AT4G30270	Spongy Mesophyll cell	Op01g00874
30	AT4G31800	Spongy Mesophyll cell	Op08g01349
31	AT4G34150	Spongy Mesophyll cell	Op03g01965
32	AT4G37870	Spongy Mesophyll cell	Op05g01886
33	AT4G38550	Spongy Mesophyll cell	Op05g01655
34	AT5G04480	Spongy Mesophyll cell	Op02g00419
35	AT5G09440	Spongy Mesophyll cell	Op03g01758
36	AT5G16560	Spongy Mesophyll cell	Op05g00916
37	AT5G18170	Spongy Mesophyll cell	Op01g02536
38	AT5G22250	Spongy Mesophyll cell	Op07g01186
39	AT5G56980	Spongy Mesophyll cell	Op04g01532
40	AT5G57660	Spongy Mesophyll cell	Op01g00899
41	AT5G62280	Spongy Mesophyll cell	Op03g00013
42	AT5G67450	Spongy Mesophyll cell	Op09g00581
43	AT1G05190	Palisade Mesophyll cell	Op09g01419
44	AT1G14150	Palisade Mesophyll cell	Op06g00474
45	AT1G32470	Palisade Mesophyll cell	Op04g00607
46	AT1G70760	Palisade Mesophyll cell	Op06g01419
47	AT3G15570	Palisade Mesophyll cell	Op08g01479
48	AT3G21055	Palisade Mesophyll cell	Op07g00804
49	AT3G47070	Palisade Mesophyll cell	Op07g00219
50	AT3G47650	Palisade Mesophyll cell	Op01g02212
51	AT3G62410	Palisade Mesophyll cell	Op07g02010
52	AT5G38410	Palisade Mesophyll cell	Op05g01780
53	AT5G63780	Palisade Mesophyll cell	Op05g01144

Table S7. Transcript accumulation of the camptothecin biosynthetic pathway genes in different cell types.

	gene_name	EC	PMC	SMC	VC
1	Op05g00545(G10H)	0.316901428	0.019714482	0.00354982	0.050188437
2	Op04g01394(10HGO)	11.62613083	4.096475409	2.555816467	0.713884447
4	Op03g01921(IS)	14.33131774	8.730681521	4.476974462	2.5009399
7	Op01g00532(IO)	2.884771243	3.429566468	0.953983662	8.912541444
8	Op09g01577(7DLGT)	1.181458003	0.174880525	0.116304007	0.045310511
10	Op10g00805(7DLH)	4.575930064	6.441979943	2.149076633	0.817567747
12	Op10g00724(LAMT)	6.044441211	0.456731597	0.042756649	0.320184911
13	Op10g00801(SLS)	3.963642667	2.517707539	4.466847891	0.441165992
18	Op07g00629(TDC)	27.63087085	2.423497295	0.277918174	0.646783353
22	Op10g01176(STR)	77.98345417	13.29721747	1.203537472	2.681185809

Table S8. Correlation analysis of transcription factors with functional genes for camptothecin biosynthesis.

Var1	Var2	cor	p_value
Op11g00943	OpTDC1	0.9994059	1.68187E-11
Op11g00943	OpG10H1	0.999115813	6.76188E-11
Op11g00943	OpSTR1	0.998856625	1.66232E-10
Op08g01712	OpSTR1	0.998292422	6.76313E-10
Op08g01712	OpTDC1	0.99792341	1.34087E-09
Op01g00882	OpLAMT1	0.997690554	1.94464E-09
Op07g01327	OpTDC1	0.99748452	2.62209E-09
Op10g01485	OpSTR1	0.997096147	4.33233E-09
Op08g01712	OpG10H1	0.996816427	5.97577E-09
Op07g01327	OpSTR1	0.996594613	7.56277E-09
Op10g01259	OpTDC1	0.996228113	1.08118E-08
Op04g00341	OpSTR1	0.995809688	1.56181E-08
Op07g01323	OpG10H1	0.99576658	1.61871E-08
Op10g01259	OpSTR1	0.995650881	1.7787E-08
Op10g01485	OpG10H1	0.99549041	2.01888E-08
Op07g01323	OpTDC1	0.995461051	2.0652E-08
Op07g01327	OpG10H1	0.995191658	2.52632E-08
Op10g01485	OpTDC1	0.995137635	2.62693E-08
Op02g02106	OpG10H1	0.994982805	2.9311E-08
Op10g01259	OpG10H1	0.994389867	4.33084E-08
Op04g00399	OpSTR1	0.994374273	4.37305E-08
Op08g00159	Op7DLGT1	0.994240062	4.74859E-08
Op05g00930	OpSLS1	0.994156038	4.99509E-08
Op03g00128	OpG10H1	0.994087131	5.20393E-08
Op10g00726	Op10HG01	0.993952555	5.62967E-08
Op08g01189	OpG10H1	0.993938413	5.6758E-08
Op01g02269	OpTDC1	0.993621073	6.78355E-08
Op04g00399	OpG10H1	0.993581931	6.9301E-08
Op04g00341	OpTDC1	0.993222024	8.38536E-08
Op07g01323	OpSTR1	0.992995121	9.40758E-08
Op03g00603	OpSTR1	0.992992023	9.42212E-08
Op03g00603	OpTDC1	0.99278132	1.04493E-07
Op03g00603	OpG10H1	0.992653263	1.11112E-07
Op08g01189	OpTDC1	0.992383228	1.26042E-07
Op04g00399	OpTDC1	0.992115895	1.42179E-07
Op01g02269	OpSTR1	0.991942076	1.5343E-07
Op04g01583	OpLAMT1	0.991788094	1.63915E-07
Op03g00057	OpLAMT1	0.991779839	1.64491E-07
Op04g01692	OpSLS1	0.991426583	1.90524E-07
Op03g00426	OpLAMT1	0.991381907	1.94013E-07
Op08g01189	OpSTR1	0.991284102	2.0181E-07
Op02g00585	OpSTR1	0.990848458	2.39275E-07
Op01g00101	OpLAMT1	0.990574796	2.65197E-07
Op09g01542	Op10HG01	0.990557561	2.66894E-07
Op02g01543	OpSTR1	0.990296107	2.93592E-07
Op03g00128	OpTDC1	0.989995478	3.26582E-07
Op04g00341	OpG10H1	0.989854773	3.42896E-07
Op06g00029	Op7DLH1	0.989663476	3.65995E-07
Op05g00909	OpLAMT1	0.989603698	3.73435E-07
Op03g00318	OpG10H1	0.989143755	4.34333E-07
Op05g00553	Op7DLGT1	0.988842856	4.77807E-07
Op03g00128	OpSTR1	0.988833071	4.79271E-07
Op02g02106	OpTDC1	0.988789724	4.85793E-07
Op03g00035	Op7DLGT1	0.988724433	4.95737E-07
Op02g01543	OpG10H1	0.988509602	5.29478E-07
Op07g00667	Op10HG01	0.988505864	5.3008E-07
Op02g00585	OpTDC1	0.988361912	5.53603E-07
Op07g01467	OpIO1	0.98802784	6.11047E-07
Op01g00702	OpLAMT1	0.987885572	6.36753E-07
Op05g01879	OpG10H1	0.987600383	6.90588E-07

Op03g00318	Op10HG01	0.987397453	7.30816E-07
Op01g02269	OpG10H1	0.987377355	7.34889E-07
Op02g02106	OpSTR1	0.986898666	8.36752E-07
Op02g01543	OpTDC1	0.986700383	8.81749E-07
Op05g00287	Op10HG01	0.986600378	9.05086E-07
Op05g01662	OpSLS1	0.986535337	9.20497E-07
Op10g01256	OpSLS1	0.985872634	1.08836E-06
Op03g00318	OpTDC1	0.985747801	1.12225E-06
Op01g00101	OpSTR1	0.985669959	1.14377E-06
Op06g00709	OpG10H1	0.985435769	1.21026E-06
Op01g00100	OpG10H1	0.985371506	1.22897E-06
Op05g01879	OpTDC1	0.985350052	1.23527E-06
Op04g00399	OpLAMT1	0.985120671	1.30401E-06
Op05g00428	Op7DLGT1	0.985107592	1.30801E-06
Op03g00318	OpSTR1	0.985094334	1.31207E-06
Op06g00709	OpSTR1	0.984945424	1.35832E-06
Op08g01598	OpSLS1	0.984732029	1.42662E-06
Op06g00448	OpG10H1	0.98471534	1.43206E-06
Op05g00707	Op7DLGT1	0.984713878	1.43254E-06
Op05g01879	OpSTR1	0.984592671	1.47251E-06
Op07g01436	Op7DLH1	0.984434729	1.52579E-06
Op09g01542	OpLAMT1	0.984344489	1.55684E-06
Op02g01996	OpCYP716E111	0.984077975	1.65116E-06
Op04g00332	Op7DLH1	0.983862136	1.73047E-06
Op10g01520	OpSLS1	0.983566564	1.84343E-06
Op04g01272	OpSLS1	0.98353929	1.85411E-06
Op01g00643	OpSLS1	0.983405876	1.907E-06
Op04g00036	OpSLS1	0.983347352	1.93053E-06
Op10g00726	OpLAMT1	0.983251735	1.96942E-06
Op04g01583	Op10HG01	0.983181368	1.9984E-06
Op02g02106	Op10HG01	0.98282717	2.14887E-06
Op02g00585	OpG10H1	0.98254284	2.27536E-06
Op03g00128	OpLAMT1	0.982538808	2.27719E-06
Op03g00057	Op10HG01	0.982458924	2.31368E-06
Op07g00667	OpLAMT1	0.982176721	2.44593E-06
Op06g00448	OpSTR1	0.982172411	2.44799E-06
Op06g00709	OpLAMT1	0.982162658	2.45266E-06
Op05g01593	OpSTR1	0.982032184	2.5157E-06
Op06g00448	OpTDC1	0.982012165	2.52548E-06
Op01g00101	OpG10H1	0.981864828	2.59825E-06
Op06g00709	OpTDC1	0.98159895	2.73332E-06
Op01g00101	OpTDC1	0.981597019	2.73432E-06
Op07g01668	OpLAMT1	0.98149166	2.78922E-06
Op07g01346	Op10HG01	0.981491389	2.78936E-06
Op05g00375	Op7DLGT1	0.981306319	2.88768E-06
Op06g00217	OpSTR1	0.980946759	3.08571E-06
Op10g01485	OpLAMT1	0.980847806	3.14186E-06
Op05g01593	OpG10H1	0.980026578	3.63628E-06
Op09g01109	OpSLS1	0.979756158	3.81051E-06
Op02g01429	Op7DLGT1	0.979565509	3.93687E-06
Op01g00100	OpTDC1	0.979022164	4.31329E-06
Op04g00341	OpLAMT1	0.97899961	4.32945E-06
Op01g00882	OpG10H1	0.978558952	4.65383E-06
Op05g01593	OpTDC1	0.978109663	5.00197E-06
Op10g00005	Op7DLH1	0.978018779	5.07458E-06
Op01g00100	OpSTR1	0.977946936	5.13251E-06
Op06g00396	OpSLS1	0.97781863	5.23713E-06
Op03g00128	Op10HG01	0.977561453	5.45138E-06
Op02g01543	OpLAMT1	0.977488881	5.51294E-06
Op04g01664	OpSTR1	0.977403864	5.58569E-06
Op09g00668	OpSLS1	0.977293165	5.68144E-06
Op05g00547	OpIS1	0.977054968	5.89141E-06

Op03g00720	OpIS1	0.977027678	5.91581E-06
Op01g00882	OpSTR1	0.977016061	5.92622E-06
Op07g00186	OpSLS1	0.976124801	6.76437E-06
Op04g01664	OpTDC1	0.975800858	7.08883E-06
Op06g00709	Op10HG01	0.97574142	7.14954E-06
Op04g01003	Op7DLH1	0.975524487	7.37426E-06
Op06g00217	OpTDC1	0.975380555	7.5261E-06
Op08g01141	Op7DLGT1	0.975336054	7.57349E-06
Op10g00726	OpG10H1	0.975072643	7.85835E-06
Op01g01804	Op7DLGT1	0.974932969	8.01245E-06
Op02g01436	OpSLS1	0.974872315	8.08003E-06
Op02g02106	OpLAMT1	0.974620156	8.36533E-06
Op01g00882	OpTDC1	0.974545488	8.45117E-06
Op07g01645	OpSTR1	0.974433857	8.58066E-06
Op04g01664	OpG10H1	0.974211773	8.84246E-06
Op10g00506	OpSTR1	0.973660717	9.51649E-06
Op03g00318	OpLAMT1	0.973581323	9.61652E-06
Op07g01645	OpTDC1	0.973479351	9.74608E-06
Op11g00564	OpSLS1	0.973316682	9.95533E-06
Op08g00836	OpTDC1	0.972618099	1.08903E-05
Op01g02524	OpCYP716E111	0.972516599	1.10312E-05
Op11g00347	Op10HG01	0.972361695	1.12486E-05
Op07g01321	OpCYP716E111	0.972090419	1.16367E-05
Op07g00105	Op7DLGT1	0.971545371	1.2445E-05
Op03g01308	OpCYP716E111	0.971332066	1.2772E-05
Op05g01593	OpLAMT1	0.971218706	1.29481E-05
Op07g00667	OpG10H1	0.97090751	1.34407E-05
Op01g00882	Op10HG01	0.970768792	1.36645E-05
Op10g00726	OpIO1	0.970534997	1.40476E-05
Op06g00217	OpG10H1	0.970383941	1.42992E-05
Op06g00046	OpSLS1	0.970199733	1.46102E-05
Op07g01645	OpG10H1	0.969877733	1.51655E-05
Op08g00836	OpSTR1	0.969807191	1.52891E-05
Op10g00040	Op7DLGT1	0.969633844	1.55959E-05
Op05g00287	OpLAMT1	0.969366834	1.60769E-05
Op05g01593	Op10HG01	0.969260879	1.62707E-05
Op11g00560	Op7DLH1	0.969190816	1.63997E-05
Op02g02305	Op7DLGT1	0.969116446	1.65375E-05
Op02g01543	Op10HG01	0.968833139	1.70698E-05
Op10g00726	OpSTR1	0.968666519	1.73885E-05
Op05g00457	OpLAMT1	0.968565526	1.75837E-05
Op10g01423	OpLAMT1	0.968493139	1.77246E-05
Op03g00635	Op7DLGT1	0.968299651	1.81051E-05
Op07g01346	OpIO1	0.967637963	1.94499E-05
Op08g00805	OpIO1	0.967331961	2.00952E-05
Op10g00506	OpTDC1	0.967328961	2.01016E-05
Op06g01598	Op7DLGT1	0.967320543	2.01196E-05
Op11g00943	OpLAMT1	0.967290174	2.01845E-05
Op11g00347	OpLAMT1	0.9670287	2.07496E-05
Op09g00537	OpLAMT1	0.966661844	2.15611E-05
Op10g00542	OpIO1	0.966559334	2.17919E-05
Op10g00726	OpTDC1	0.966361451	2.22422E-05
Op03g02189	OpCYP716E111	0.966243316	2.25142E-05
Op10g00506	OpG10H1	0.965840244	2.34599E-05
Op06g00709	OpIO1	0.965588095	2.40656E-05
Op03g01991	OpSLS1	0.965277534	2.48268E-05
Op07g01668	Op10HG01	0.965230265	2.49442E-05
Op01g00100	OpIO1	0.964617889	2.65E-05
Op05g00909	OpSTR1	0.964499076	2.68096E-05
Op01g00113	OpSTR1	0.964486124	2.68435E-05
Op07g01346	OpG10H1	0.964425783	2.70019E-05
Op04g00084	OpLAMT1	0.964346505	2.72109E-05

Op04g01583	OpG10H1	0.964286633	2.73696E-05
Op05g01962	OpCYP716E111	0.964265635	2.74254E-05
Op03g01579	OpSLS1	0.96421708	2.75547E-05
Op01g01993	OpIS1	0.964203225	2.75917E-05
Op04g00399	Op10HG01	0.964008981	2.81139E-05
Op03g00426	OpSTR1	0.964006792	2.81198E-05
Op07g01321	Op7DLGT1	0.963681029	2.90114E-05
Op09g01542	OpIO1	0.963566532	2.93295E-05
Op02g01358	Op7DLH1	0.963501079	2.95124E-05
Op05g00040	Op7DLGT1	0.963449308	2.96577E-05
Op04g00296	OpCYP716E111	0.963376891	2.98617E-05
Op08g01712	OpLAMT1	0.963312353	3.00444E-05
Op01g00100	Op10HG01	0.96328742	3.01152E-05
Op03g01395	OpIO1	0.963051141	3.07918E-05
Op03g00057	OpIO1	0.962312378	3.2977E-05
Op01g00702	Op10HG01	0.962167663	3.34176E-05
Op08g00836	OpG10H1	0.962166709	3.34205E-05
Op03g00057	OpG10H1	0.962104172	3.36122E-05
Op08g01141	OpCYP716E111	0.961935319	3.41336E-05
Op07g01668	OpSTR1	0.96139831	3.583E-05
Op05g00707	OpCYP716E111	0.961007326	3.7102E-05
Op09g00218	OpSLS1	0.960947218	3.73004E-05
Op10g01485	Op10HG01	0.960879778	3.75238E-05
Op02g02090	OpSLS1	0.960735926	3.80036E-05
Op03g00035	OpCYP716E111	0.960513466	3.87541E-05
Op05g00988	Op7DLH1	0.960373979	3.92299E-05
Op07g00667	OpTDC1	0.960271768	3.95812E-05
Op07g00667	OpSTR1	0.960150183	4.0002E-05
Op03g01309	OpCYP716E111	0.959590594	4.19797E-05
Op02g02305	OpCYP716E111	0.959088383	4.38125E-05
Op11g00110	Op10HG01	0.95907219	4.38726E-05
Op01g00399	OpCYP716E111	0.95893176	4.43955E-05
Op09g00537	Op10HG01	0.958930118	4.44017E-05
Op11g00110	OpLAMT1	0.958622645	4.55622E-05
Op03g00057	OpSTR1	0.958068336	4.77084E-05
Op07g00667	OpIO1	0.958053507	4.77668E-05
Op07g00071	OpIO1	0.958019478	4.7901E-05
Op03g00426	OpG10H1	0.957937621	4.82248E-05
Op01g00113	OpTDC1	0.957834098	4.86365E-05
Op07g01645	Op10HG01	0.957761741	4.89257E-05
Op03g01308	Op7DLGT1	0.957704637	4.91549E-05
Op03g01392	OpSLS1	0.957543558	4.98053E-05
Op07g00317	OpLAMT1	0.957252381	5.09964E-05
Op07g01467	Op10HG01	0.957200198	5.1212E-05
Op04g01583	OpSTR1	0.957192103	5.12455E-05
Op03g00426	OpTDC1	0.957163176	5.13653E-05
Op11g00943	Op10HG01	0.95712553	5.15216E-05
Op09g00421	OpSLS1	0.95702635	5.19349E-05
Op07g01645	OpLAMT1	0.956398995	5.46034E-05
Op03g00603	OpLAMT1	0.956131614	5.57697E-05
Op05g00909	OpTDC1	0.95597157	5.64761E-05
Op05g00018	OpCYP716E111	0.955690635	5.77315E-05
Op07g01668	OpG10H1	0.955644441	5.79397E-05
Op01g01085	OpIO1	0.955572075	5.82671E-05
Op02g01676	OpCYP716E111	0.955568238	5.82845E-05
Op09g01542	OpG10H1	0.955548222	5.83753E-05
Op04g01583	OpTDC1	0.955242169	5.97761E-05
Op08g01349	Op7DLH1	0.9548995	6.13725E-05
Op02g01996	Op7DLGT1	0.954706191	6.22862E-05
Op01g02419	OpSLS1	0.954666786	6.24736E-05
Op08g00096	OpSTR1	0.954488696	6.33257E-05
Op01g01992	Op7DLGT1	0.954440276	6.35588E-05

Op03g02182	Op7DLH1	0.953859567	6.64015E-05
Op05g00828	OpCYP716E111	0.953849029	6.6454E-05
Op03g00057	OpTDC1	0.953664348	6.73771E-05
Op07g01668	OpTDC1	0.953199159	6.97424E-05
Op02g01989	OpIS1	0.953012488	7.07078E-05
Op05g00040	OpCYP716E111	0.95279703	7.18338E-05
Op10g01526	Op7DLGT1	0.952778008	7.19338E-05
Op01g00846	OpSLS1	0.952573275	7.30165E-05
Op06g00445	Op10HG01	0.952044588	7.58655E-05
Op07g00296	OpSLS1	0.951904192	7.66351E-05
Op09g00150	OpLAMT1	0.951653092	7.80253E-05
Op06g00448	OpIO1	0.951547639	7.86143E-05
Op08g01189	Op10HG01	0.951531328	7.87057E-05
Op05g00909	OpG10H1	0.951452049	7.9151E-05
Op07g00317	Op10HG01	0.951384799	7.95301E-05
Op04g00296	OpSLS1	0.951384675	7.95308E-05
Op04g01664	Op10HG01	0.951154759	8.08367E-05
Op07g01346	OpTDC1	0.950889871	8.23598E-05
Op06g00071	OpCYP716E111	0.950868909	8.24812E-05
Op04g01664	OpIO1	0.950824747	8.27374E-05
Op09g01542	OpSTR1	0.950338303	8.55963E-05
Op02g00585	OpLAMT1	0.950337091	8.56035E-05
Op01g00101	Op10HG01	0.950325306	8.56736E-05
Op09g00763	OpLAMT1	0.950279047	8.59493E-05
Op08g00096	OpLAMT1	0.95024424	8.61571E-05
Op07g01967	OpCYP716E111	0.950192349	8.64675E-05
Op01g00113	OpG10H1	0.950148814	8.67286E-05
Op08g01717	OpLAMT1	0.950037269	8.74001E-05
Op03g00603	Op10HG01	0.949990252	8.76842E-05
Op07g01327	OpLAMT1	0.949895014	8.82617E-05
Op07g02009	OpSLS1	0.949856712	8.84947E-05
Op09g00192	Op7DLGT1	0.949765235	8.9053E-05
Op06g00071	OpSLS1	0.949713673	8.93688E-05
Op02g01603	OpCYP716E111	0.949707653	8.94057E-05
Op08g01189	OpLAMT1	0.949441021	9.10517E-05
Op07g00319	OpSLS1	0.949409937	9.12449E-05
Op07g01323	OpLAMT1	0.949225998	9.23945E-05
Op01g00100	OpLAMT1	0.94902137	9.36854E-05
Op06g01598	OpCYP716E111	0.948960769	9.40701E-05
Op07g01346	OpLAMT1	0.948710056	9.56736E-05
Op06g00448	OpLAMT1	0.948591478	9.64387E-05
Op03g00317	Op7DLH1	0.948507436	9.69836E-05
Op10g01259	OpLAMT1	0.948330687	9.81365E-05
Op09g00904	Op7DLH1	0.948155917	9.92861E-05
Op10g00506	OpLAMT1	0.948017082	0.000100206
Op02g01571	Op7DLH1	0.947996237	0.000100345
Op06g00476	OpSLS1	0.947701301	0.000102321
Op08g01712	Op10HG01	0.947672007	0.000102518
Op06g00031	Op7DLH1	0.947549306	0.00010335
Op07g01346	OpSTR1	0.947468402	0.0001039
Op02g01358	OpSLS1	0.947415304	0.000104263
Op02g01676	OpSLS1	0.947377942	0.000104519
Op03g02033	OpSLS1	0.947374014	0.000104546
Op05g00428	OpCYP716E111	0.947281821	0.000105178
Op01g02524	Op7DLGT1	0.947230769	0.00010553
Op11g00917	OpCYP716E111	0.94675357	0.000108857
Op06g00071	Op7DLGT1	0.946716926	0.000109115
Op09g00904	OpSLS1	0.946632787	0.00010971
Op05g00018	Op7DLGT1	0.94656093	0.00011022
Op10g00506	OpIO1	0.946367425	0.000111602
Op07g01967	Op7DLGT1	0.94614513	0.000113204
Op09g01542	OpTDC1	0.945957261	0.000114571

Op04g01583	OpIO1	0.945832882	0.000115483
Op05g01879	OpLAMT1	0.945577211	0.000117372
Op02g01543	OpIO1	0.945548864	0.000117583
Op05g01332	OpIS1	0.945159992	0.000120502
Op06g00217	OpLAMT1	0.944753116	0.00012361
Op08g00096	OpTDC1	0.9446566	0.000124356
Op06g00445	OpLAMT1	0.944639581	0.000124487
Op09g00150	OpSTR1	0.944592545	0.000124852
Op06g00074	OpSLS1	0.94450258	0.000125552
Op06g00448	Op10HG01	0.944266028	0.000127405
Op07g01668	OpIO1	0.944259878	0.000127453
Op07g01323	Op10HG01	0.944006326	0.000129462
Op02g00436	OpSTR1	0.943976386	0.0001297
Op02g00265	Op7DLGT1	0.943560057	0.00013305
Op04g00341	Op10HG01	0.943542188	0.000133195
Op04g01664	OpLAMT1	0.943539743	0.000133215
Op05g00583	Op7DLH1	0.943438019	0.000134044
Op08g01256	OpSLS1	0.943295821	0.000135208
Op02g02106	OpIO1	0.943277846	0.000135355
Op04g01490	Op10HG01	0.943264687	0.000135463
Op05g01879	OpIO1	0.943180485	0.000136157
Op04g00146	OpIO1	0.942824142	0.00013912
Op04g01489	Op7DLGT1	0.942781442	0.000139478
Op05g00598	Op7DLGT1	0.942741718	0.000139812
Op01g00399	Op7DLGT1	0.942422953	0.00014251
Op05g01879	Op10HG01	0.942405176	0.000142661
Op10g00506	Op10HG01	0.941661967	0.000149099
Op03g00914	OpTDC1	0.941207701	0.000153133
Op01g00545	OpCYP716E111	0.941018859	0.000154833
Op01g00434	OpCYP716E111	0.940897603	0.000155931
Op05g00287	OpG10H1	0.94006328	0.000163636
Op05g00255	OpCYP716E111	0.940017114	0.00016407
Op09g00218	Op7DLGT1	0.939805523	0.00016607
Op04g00296	Op7DLGT1	0.939663164	0.000167425
Op03g00024	OpSLS1	0.939661918	0.000167437
Op02g01495	Op7DLGT1	0.939354223	0.000170392
Op10g01259	Op10HG01	0.939350858	0.000170425
Op07g01327	Op10HG01	0.939069726	0.000173158
Op09g00730	OpCYP716E111	0.939008869	0.000173753
Op09g00167	OpSLS1	0.938915545	0.000174669
Op08g00096	OpG10H1	0.93864001	0.000177394
Op03g00603	OpIO1	0.938425677	0.000179534
Op04g00399	OpIO1	0.938341762	0.000180377
Op05g01962	Op7DLGT1	0.938325417	0.000180541
Op05g00909	Op10HG01	0.938189517	0.000181913
Op03g00914	OpSTR1	0.938095893	0.000182862
Op05g01151	OpSLS1	0.937970995	0.000184134
Op01g00452	Op10HG01	0.937945021	0.000184399
Op03g00426	Op10HG01	0.937851749	0.000185354
Op05g00375	OpCYP716E111	0.937683391	0.000187086
Op10g01443	OpLAMT1	0.937668471	0.00018724
Op08g01189	OpIO1	0.937423689	0.00018978
Op07g01652	OpLAMT1	0.937355137	0.000190495
Op02g01896	OpIS1	0.937347067	0.00019058
Op09g00165	OpIS1	0.937274831	0.000191336
Op08g01717	Op10HG01	0.937065669	0.000193538
Op10g00615	OpSLS1	0.937062062	0.000193576
Op04g01490	OpLAMT1	0.936985005	0.000194392
Op03g02182	OpSLS1	0.936848381	0.000195845
Op01g00434	OpSLS1	0.93664048	0.00019807
Op05g01778	OpCYP716E111	0.936618875	0.000198302
Op07g00762	OpLAMT1	0.935933697	0.000205767

Op05g00333	Op7DLGT1	0.935927696	0.000205833
Op01g00113	OpLAMT1	0.935542344	0.000210118
Op03g02189	Op7DLGT1	0.93525731	0.000213327
Op02g00436	OpTDC1	0.935036678	0.000215835
Op05g00553	OpCYP716E111	0.934979136	0.000216493
Op09g00165	OpIO1	0.934974816	0.000216542
Op01g02269	OpLAMT1	0.934820604	0.000218311
Op04g01003	OpSLS1	0.934740665	0.000219232
Op08g00159	OpCYP716E111	0.934395653	0.000223239
Op09g00167	Op7DLH1	0.93439349	0.000223265
Op09g01495	OpSLS1	0.934158412	0.000226024
Op02g01575	Op7DLH1	0.934153176	0.000226086
Op01g01475	Op7DLGT1	0.934085141	0.000226889
Op10g00933	Op10HG01	0.933977134	0.000228169
Op06g00964	Op7DLGT1	0.933581673	0.000232896
Op04g00084	OpIO1	0.933526084	0.000233566
Op11g00347	OpG10H1	0.93306744	0.000239146
Op05g00628	Op7DLH1	0.932865464	0.000241633
Op09g00150	OpTDC1	0.9328598	0.000241703
Op03g01309	Op7DLGT1	0.932731946	0.000243286
Op03g02254	OpLAMT1	0.9325702	0.0002453
Op11g00563	OpSLS1	0.932404336	0.000247378
Op01g01992	OpCYP716E111	0.932085596	0.000251405
Op03g01991	OpCYP716E111	0.931886197	0.000253947
Op09g00730	OpSLS1	0.931798412	0.000255072
Op07g01436	OpSLS1	0.931758238	0.000255589
Op05g00287	OpSTR1	0.931174579	0.000263169
Op01g00702	OpG10H1	0.931090952	0.000264267
Op02g01429	OpCYP716E111	0.931081418	0.000264393
Op02g00329	Op7DLH1	0.930785318	0.000268311
Op10g01485	OpIO1	0.930767992	0.000268541
Op03g00318	OpIO1	0.930702327	0.000269416
Op02g00265	Op7DLH1	0.930670691	0.000269838
Op01g00434	Op7DLGT1	0.930532989	0.000271682
Op05g01593	OpIO1	0.9302735	0.000275179
Op09g00763	Op10HG01	0.929283723	0.00028881
Op05g00287	OpTDC1	0.92919906	0.000289997
Op02g01603	Op7DLGT1	0.929155093	0.000290615
Op10g00249	OpSLS1	0.929144613	0.000290763
Op05g01852	OpLAMT1	0.92860684	0.0002984
Op09g00150	OpG10H1	0.928266821	0.000303301
Op10g00933	OpLAMT1	0.928254683	0.000303477
Op05g00333	OpCYP716E111	0.928188561	0.000304437
Op09g00470	Op7DLH1	0.92816312	0.000304807
Op03g01674	Op7DLGT1	0.92809402	0.000305813
Op05g01746	OpSLS1	0.927768457	0.000310586
Op09g00198	OpIS1	0.927660029	0.000312187
Op05g00457	OpSTR1	0.92750226	0.000314527
Op02g00436	OpLAMT1	0.927398965	0.000316066
Op10g00542	OpSTR1	0.927298299	0.00031757
Op05g00828	Op7DLGT1	0.927089206	0.000320711
Op01g00702	OpSTR1	0.92690031	0.000323568
Op04g00077	OpSLS1	0.92688189	0.000323847
Op01g00882	OpIO1	0.926869029	0.000324043
Op09g01461	Op7DLGT1	0.926776996	0.000325442
Op06g00445	OpG10H1	0.926542857	0.000329022
Op01g00101	OpIO1	0.926341511	0.000332123
Op07g01430	OpIO1	0.926341268	0.000332127
Op04g00084	Op10HG01	0.925949308	0.000338221
Op02g00933	OpIO1	0.925876918	0.000339355
Op05g01332	OpIO1	0.925307843	0.000348364
Op09g01380	Op7DLH1	0.925196417	0.000350147

Op03g01684	OpSLS1	0.924358577	0.000363762
Op10g01423	Op10HG01	0.923913342	0.000371146
Op04g01455	OpIO1	0.92386657	0.000371928
Op04g00567	Op7DLH1	0.923754207	0.000373811
Op02g00436	OpG10H1	0.923695806	0.000374792
Op09g00535	OpCYP716E111	0.923626288	0.000375962
Op08g00362	OpSLS1	0.923593915	0.000376508
Op10g00542	OpG10H1	0.923557376	0.000377125
Op03g00914	OpG10H1	0.923277155	0.000381879
Op10g00040	OpCYP716E111	0.923024111	0.000386208
Op05g01962	OpSLS1	0.922939014	0.000387671
Op05g00668	OpTDC1	0.9227249	0.00039137
Op01g00702	OpTDC1	0.922377936	0.000397417
Op05g00668	OpSTR1	0.921890504	0.000406023
Op08g00071	OpCYP716E111	0.92181938	0.000407289
Op11g00347	OpTDC1	0.92177604	0.000408062
Op01g02117	OpSLS1	0.921644641	0.000410413
Op01g02269	Op10HG01	0.921566464	0.000411815
Op03g00128	OpIO1	0.921440409	0.000414084
Op08g00190	OpSLS1	0.921379035	0.000415192
Op05g00325	OpSLS1	0.921310075	0.000416439
Op03g00426	OpIO1	0.92096738	0.000422677
Op11g00347	OpSTR1	0.920830502	0.000425186
Op02g01377	Op10HG01	0.920778763	0.000426138
Op05g01662	Op7DLH1	0.920570746	0.000429977
Op05g01778	Op7DLGT1	0.920551935	0.000430326
Op10g00542	OpTDC1	0.92054725	0.000430412
Op03g01078	Op7DLH1	0.92024053	0.000436122
Op08g00362	Op7DLH1	0.92000568	0.00044053
Op05g00287	OpIO1	0.919786424	0.000444673
Op06g00159	Op10HG01	0.919733228	0.000445682
Op07g00296	OpCYP716E111	0.919704174	0.000446234
Op06g00046	OpCYP716E111	0.919562529	0.000448931
Op07g01467	OpG10H1	0.919542645	0.000449311
Op02g01976	Op7DLGT1	0.919422672	0.000451606
Op04g01489	OpCYP716E111	0.919375902	0.000452503
Op02g01495	OpCYP716E111	0.918928942	0.000461138
Op04g01490	OpSTR1	0.918921001	0.000461292
Op01g01804	OpCYP716E111	0.918500163	0.000469529
Op08g00071	Op7DLGT1	0.917484821	0.000489824
Op04g00241	OpIS1	0.91732858	0.000493001
Op06g00624	Op7DLGT1	0.917285274	0.000493884
Op08g01016	Op10HG01	0.91727284	0.000494138
Op04g00084	OpSTR1	0.917219326	0.000495231
Op05g00668	OpG10H1	0.917157674	0.000496492
Op07g00618	Op7DLH1	0.916885771	0.000502082
Op09g00730	Op7DLGT1	0.916655269	0.000506856
Op06g00688	OpSLS1	0.916538017	0.000509296
Op03g00098	OpSLS1	0.916121064	0.00051804
Op06g00445	OpTDC1	0.916097832	0.000518531
Op01g01833	Op7DLH1	0.915978562	0.000521053
Op03g01392	OpCYP716E111	0.915972579	0.00052118
Op08g00805	Op10HG01	0.915792195	0.000525011
Op02g00030	Op7DLH1	0.915745967	0.000525996
Op01g00702	OpIO1	0.915727971	0.00052638
Op08g01712	OpIO1	0.915713294	0.000526693
Op11g00917	Op7DLGT1	0.91569973	0.000526983
Op05g00457	OpTDC1	0.915694777	0.000527089
Op11g00943	OpIO1	0.915643301	0.000528189
Op01g01085	OpLAMT1	0.91563307	0.000528407
Op05g00457	OpG10H1	0.915445755	0.000532425
Op06g00009	Op10HG01	0.915414561	0.000533096

Op01g00452	OpLAMT1	0.914924351	0.000543721
Op07g01674	Op7DLGT1	0.91479876	0.000546466
Op09g00470	OpSLS1	0.91476605	0.000547183
Op02g00585	Op10HG01	0.914760489	0.000547305
Op07g01673	OpSLS1	0.914756418	0.000547394
Op09g00904	Op7DLGT1	0.914483624	0.000553398
Op07g00319	Op7DLH1	0.914326563	0.000556876
Op10g00450	OpSLS1	0.914024349	0.000563611
Op10g01443	OpSTR1	0.913988799	0.000564407
Op03g00694	OpSLS1	0.913897447	0.000566456
Op05g00988	OpSLS1	0.913778629	0.000569129
Op09g01437	OpSLS1	0.913059281	0.000585499
Op06g00217	Op10HG01	0.912919875	0.000588709
Op07g00838	Op7DLH1	0.912852638	0.000590262
Op05g00457	OpIO1	0.912775512	0.000592046
Op04g00341	OpIO1	0.912745932	0.000592732
Op06g00445	OpSTR1	0.912712914	0.000593498
Op09g00660	Op7DLH1	0.912520272	0.000597979
Op01g00201	OpIS1	0.91247503	0.000599035
Op09g00667	OpSLS1	0.912466989	0.000599223
Op08g00805	OpLAMT1	0.912299031	0.000603155
Op01g00524	OpCYP716E111	0.912043572	0.00060917
Op10g01445	OpIS1	0.911704802	0.000617212
Op10g00542	OpLAMT1	0.911676885	0.000617878
Op04g00241	Op10HG01	0.911462014	0.000623021
Op04g01490	OpG10H1	0.911160904	0.000630278
Op09g00218	OpCYP716E111	0.910603017	0.000643879
Op07g00317	OpIO1	0.910516264	0.000646012
Op07g01467	OpLAMT1	0.910351269	0.000650083
Op04g00241	OpLAMT1	0.910296632	0.000651435
Op07g00525	OpSLS1	0.910197734	0.000653887
Op07g00071	OpLAMT1	0.910163679	0.000654733
Op04g01272	Op7DLH1	0.90996257	0.000659744
Op02g01377	OpLAMT1	0.909530428	0.000670602
Op04g01490	OpTDC1	0.909520783	0.000670846
Op09g00150	OpIO1	0.909313396	0.000676102
Op09g01334	OpIS1	0.909219171	0.0006785
Op05g00909	OpIO1	0.90919162	0.000679202
Op07g01056	OpSLS1	0.908794091	0.00068939
Op05g00050	OpSLS1	0.908435522	0.000698671
Op07g00843	OpSLS1	0.908408702	0.000699369
Op05g00616	OpSLS1	0.908390891	0.000699833
Op03g00638	OpSLS1	0.908301752	0.000702156
Op01g02078	OpSLS1	0.907951036	0.000711349
Op03g00826	OpSLS1	0.90791281	0.000712357
Op04g00084	OpG10H1	0.90752841	0.000722539
Op11g00704	OpSLS1	0.907020634	0.000736145
Op07g00525	OpCYP716E111	0.906436417	0.00075202
Op06g00029	OpSLS1	0.906400496	0.000753004
Op02g00329	OpSLS1	0.906228248	0.000757734
Op04g01489	OpSLS1	0.906105552	0.000761115
Op05g00345	OpIS1	0.905895106	0.00076694
Op06g00618	OpSLS1	0.905753444	0.000770879
Op10g01259	OpIO1	0.905548959	0.000776589
Op08g00250	OpSLS1	0.905545927	0.000776674
Op07g01323	OpIO1	0.9054974	0.000778033
Op07g00119	Op7DLH1	0.905443024	0.000779559
Op07g01431	OpSLS1	0.905065393	0.00079021
Op05g01960	Op7DLH1	0.904920495	0.000794323
Op03g02222	OpSLS1	0.904809076	0.000797496
Op09g00904	OpCYP716E111	0.904719176	0.000800063
Op04g00084	OpTDC1	0.904432215	0.000808295

Op01g01085	Op10HG01	0.903990466	0.000821082
Op07g01327	OpIO1	0.903563518	0.000833575
Op01g01085	OpIS1	0.903544411	0.000834137
Op09g00763	OpIS1	0.903372679	0.000839201
Op03g02033	Op7DLH1	0.903311944	0.000840997
Op10g00542	Op10HG01	0.903226429	0.000843531
Op02g01575	OpSLS1	0.903077687	0.00084795
Op06g00217	OpIO1	0.902813484	0.00085584
Op05g01662	OpCYP716E111	0.90273772	0.000858112
Op02g00432	OpSLS1	0.902664912	0.0008603
Op06g00009	OpLAMT1	0.902654014	0.000860627
Op09g00535	Op7DLGT1	0.902643869	0.000860932
Op05g00457	Op10HG01	0.90262636	0.000861459
Op11g00798	Op7DLGT1	0.902310598	0.000870999
Op07g01467	OpTDC1	0.902273195	0.000872134
Op06g00046	Op7DLGT1	0.902233697	0.000873334
Op09g00218	Op7DLH1	0.902021609	0.000879795
Op06g00505	OpSLS1	0.901972116	0.000881307
Op09g00537	OpIO1	0.901711138	0.000889313
Op07g00320	OpSLS1	0.901643726	0.000891389
Op08g01667	OpSLS1	0.901423244	0.000898202
Op07g01467	OpSTR1	0.901207614	0.000904901
Op01g02383	OpIS1	0.901165694	0.000906208
Op08g01153	OpSLS1	0.900865569	0.000915599
Op06g00046	Op7DLH1	0.900692922	0.000921031
Op04g00293	OpIO1	0.900663295	0.000921966
Op10g01443	OpTDC1	0.900488706	0.000927487
Op09g00537	OpG10H1	0.900410389	0.000929971
Op05g01159	Op7DLH1	0.900399601	0.000930313
Op08g01769	OpSLS1	0.900106908	0.00093964
Op06g00700	OpSLS1	0.899975929	0.000943834
Op04g01490	OpIO1	0.899934261	0.000945171
Op08g00836	OpLAMT1	0.899268575	0.000966713
Op02g00585	OpIO1	0.899180192	0.000969598
Op10g01443	OpG10H1	0.899024188	0.000974706
Op05g01662	Op7DLGT1	0.89878242	0.000982659
Op11g00110	OpSTR1	0.898733756	0.000984266
Op04g01417	OpIS1	0.898733562	0.000984272
Op03g00067	OpIS1	0.898136447	0.00100413
Op06g00505	Op7DLH1	0.898028559	0.001007748
Op07g02009	OpCYP716E111	0.897847777	0.00101383
Op10g01423	OpIO1	0.897809832	0.001015109
Op07g01407	Op7DLH1	0.897707447	0.001018568
Op09g00537	OpSTR1	0.897571069	0.001023188
Op05g00628	Op7DLGT1	0.897539656	0.001024255
Op03g00635	OpIS1	0.897502439	0.001025519
Op06g00964	OpSLS1	0.897484834	0.001026117
Op07g01674	OpCYP716E111	0.89714708	0.001037645
Op11g00110	OpG10H1	0.896857228	0.001047609
Op08g00096	OpIO1	0.89675598	0.001051105
Op08g01016	OpLAMT1	0.896698435	0.001053096
Op01g00355	OpSLS1	0.89643357	0.001062293
Op04g00334	OpSLS1	0.895971185	0.001078482
Op11g00564	OpCYP716E111	0.895819587	0.001083826
Op02g01654	OpSLS1	0.895690333	0.001088398
Op08g01758	OpSLS1	0.895595707	0.001091753
Op08g01686	OpSLS1	0.895214877	0.00110533
Op07g01038	OpSLS1	0.895208441	0.00110556
Op11g01129	Op7DLH1	0.89504565	0.001111401
Op07g00317	OpIS1	0.89465872	0.001125367
Op02g01676	Op7DLGT1	0.894494516	0.001131331
Op10g00005	Op7DLGT1	0.894224736	0.001141178

Op04g00483	OpSLS1	0.894069858	0.001146857
Op06g00359	OpIS1	0.893812611	0.001156334
Op01g00545	Op7DLGT1	0.893738119	0.001159088
Op10g01443	OpIO1	0.893732817	0.001159285
Op11g00682	OpSLS1	0.893657515	0.001162074
Op04g01413	OpSLS1	0.893636381	0.001162858
Op11g00955	OpSLS1	0.893266634	0.001176628
Op09g00150	Op10HG01	0.893217904	0.001178451
Op03g01907	OpIS1	0.893013097	0.001186135
Op09g00763	OpIO1	0.892717866	0.001197274
Op06g00396	OpCYP716E111	0.892675627	0.001198873
Op07g01652	OpSTR1	0.892658743	0.001199513
Op11g00110	OpTDC1	0.892316977	0.001212514
Op10g01443	Op10HG01	0.892199936	0.001216989
Op01g00452	OpIS1	0.89166384	0.001237632
Op03g01739	OpSLS1	0.891127742	0.001258518
Op06g00159	OpLAMT1	0.890974044	0.001264551
Op04g00026	Op7DLGT1	0.890780175	0.001272189
Op09g00537	OpTDC1	0.890551774	0.001281229
Op03g01395	OpG10H1	0.890228536	0.001294098
Op01g00474	OpLAMT1	0.890021105	0.001302404
Op11g00347	OpIO1	0.889896097	0.001307427
Op06g01599	OpSLS1	0.88979741	0.001311402
Op06g00700	Op7DLH1	0.889795527	0.001311478
Op01g00524	Op7DLGT1	0.889718752	0.001314576
Op10g01423	OpSTR1	0.889622672	0.001318461
Op03g02189	OpSLS1	0.889592307	0.00131969
Op04g00567	OpSLS1	0.889274515	0.001332604
Op05g00333	Op7DLH1	0.889214683	0.001335046
Op03g00035	OpSLS1	0.8892084	0.001335302
Op03g02173	OpIS1	0.889190958	0.001336015
Op02g02090	OpCYP716E111	0.889140244	0.001338087
Op01g00643	OpCYP716E111	0.888870826	0.001349135
Op05g00910	OpIS1	0.888620209	0.001359469
Op09g00674	OpSLS1	0.888607454	0.001359997
Op03g00809	OpSLS1	0.8883822	0.001369334
Op03g00559	OpSLS1	0.888212193	0.001376411
Op08g01349	OpSLS1	0.887793705	0.001393939
Op03g00720	Op7DLGT1	0.88769668	0.001398025
Op07g00141	OpIS1	0.887463542	0.001407877
Op03g00635	Op7DLH1	0.887437438	0.001408983
Op02g02156	Op7DLGT1	0.8872152	0.001418424
Op08g01016	OpIO1	0.887187044	0.001419623
Op07g00071	Op10HG01	0.887021755	0.001426678
Op07g00317	OpG10H1	0.886967057	0.001429017
Op03g00951	Op7DLH1	0.886685395	0.001441108
Op10g01205	OpSLS1	0.886085207	0.001467109
Op03g00027	OpIS1	0.885989414	0.001471288
Op03g00004	OpSLS1	0.885988611	0.001471323
Op03g00035	Op7DLH1	0.885761359	0.001481272
Op04g00483	Op7DLH1	0.885444641	0.001495216
Op06g00793	OpCYP716E111	0.885067372	0.001511943
Op03g01579	OpCYP716E111	0.885066142	0.001511998
Op05g00930	Op7DLH1	0.884959684	0.001516742
Op08g01717	OpIS1	0.884886944	0.001519989
Op08g00096	Op10HG01	0.884279103	0.001547312
Op10g01423	OpG10H1	0.88420641	0.001550602
Op01g02383	Op7DLGT1	0.883739108	0.00157187
Op07g00105	OpCYP716E111	0.883668024	0.001575123
Op05g00425	OpIS1	0.883408585	0.001587034
Op08g00836	Op10HG01	0.882939214	0.001608742
Op02g01495	Op7DLH1	0.882617112	0.001623758

Op02g02156	Op7DLH1	0.882616783	0.001623773
Op11g00565	Op7DLH1	0.882575621	0.001625699
Op07g01673	Op7DLGT1	0.882432037	0.001632429
Op05g01778	OpSLS1	0.882298172	0.001638721
Op07g01235	OpIS1	0.882123285	0.001646967
Op07g00071	OpSTR1	0.882071982	0.001649391
Op04g00026	OpCYP716E111	0.881803972	0.001662096
Op03g01395	Op10HG01	0.881557905	0.00167382
Op07g00762	OpIO1	0.881368933	0.001682863
Op11g00110	OpIO1	0.881230348	0.001689516
Op03g02254	Op10HG01	0.881126099	0.001694533
Op03g01674	OpIS1	0.881054952	0.001697962
Op01g02269	OpIO1	0.880494774	0.001725134
Op03g00094	Op7DLH1	0.8804642	0.001726626
Op03g00079	Op7DLH1	0.880100407	0.001744443
Op07g01652	OpTDC1	0.879861125	0.001756231
Op07g00141	OpIO1	0.879354464	0.001781374
Op01g00368	Op7DLH1	0.879261491	0.001786015
Op05g00598	OpIS1	0.879234576	0.00178736
Op05g01332	OpLAMT1	0.879182921	0.001789943
Op03g01395	OpSTR1	0.879093044	0.001794444
Op03g01395	OpTDC1	0.878708674	0.001813781
Op06g00793	Op7DLGT1	0.878653936	0.001816547
Op03g01809	OpSLS1	0.878492857	0.001824702
Op08g01717	OpIO1	0.878165738	0.001841341
Op10g01423	OpTDC1	0.878152594	0.001842011
Op01g00113	OpIO1	0.87811644	0.001843857
Op08g01153	OpCYP716E111	0.877311908	0.001885266
Op05g01609	Op7DLH1	0.877009624	0.001900989
Op09g00165	Op10HG01	0.876988144	0.00190211
Op10g00366	Op7DLH1	0.8769815	0.001902457
Op06g00964	OpCYP716E111	0.876915009	0.001905929
Op11g00798	OpIS1	0.876914413	0.00190596
Op03g01991	Op7DLGT1	0.876594448	0.001922732
Op07g01652	OpG10H1	0.876519454	0.001926678
Op03g01479	OpIS1	0.876431549	0.00193131
Op01g00137	OpCYP716E111	0.876333819	0.001936469
Op03g01078	OpSLS1	0.876264861	0.001940115
Op04g01618	OpSLS1	0.876192	0.001943972
Op02g02090	Op7DLH1	0.876150918	0.001946149
Op01g00474	OpIS1	0.875834496	0.001962976
Op07g00317	OpSTR1	0.875534071	0.001979044
Op09g00152	OpIO1	0.875232541	0.001995264
Op07g00071	OpG10H1	0.875232194	0.001995282
Op09g01245	OpSLS1	0.875155923	0.001999399
Op01g01304	OpSLS1	0.874723654	0.002022845
Op06g00071	Op7DLH1	0.874709087	0.002023638
Op05g01852	OpIO1	0.874551237	0.002032249
Op02g01976	OpIS1	0.874509777	0.002034515
Op04g01417	OpIO1	0.874499843	0.002035058
Op07g01645	OpIO1	0.874409488	0.002040003
Op05g00669	OpIS1	0.874000873	0.00206247
Op10g01256	OpCYP716E111	0.873723474	0.002077819
Op10g01091	Op7DLH1	0.873702319	0.002078993
Op03g02254	OpIO1	0.873472695	0.002091764
Op03g01907	Op7DLGT1	0.873415972	0.002094927
Op07g00762	OpSTR1	0.873392236	0.002096251
Op02g01429	OpSLS1	0.87338502	0.002096654
Op04g01543	OpCYP716E111	0.873160243	0.002109227
Op05g01332	Op10HG01	0.873108262	0.002112143
Op11g00563	OpCYP716E111	0.873013958	0.002117438
Op07g00316	OpSLS1	0.872645186	0.002138235

Op07g00838	OpSLS1	0.872604227	0.002140554
Op01g00417	OpSLS1	0.87250904	0.002145949
Op05g01609	OpSLS1	0.872313344	0.00215707
Op04g00679	OpSLS1	0.872243313	0.002161059
Op06g00793	OpSLS1	0.872222248	0.00216226
Op01g00777	OpSLS1	0.872099207	0.002169284
Op07g00317	OpTDC1	0.872026761	0.002173428
Op03g00004	Op7DLH1	0.871935449	0.002178658
Op07g01436	Op7DLGT1	0.871839693	0.002184151
Op01g00113	Op10HG01	0.87158795	0.00219864
Op07g01673	Op7DLH1	0.871586715	0.002198712
Op07g02038	OpSLS1	0.871555233	0.002200528
Op07g00762	Op10HG01	0.871228866	0.002219422
Op08g00805	OpSTR1	0.871057857	0.002229367
Op07g00296	Op7DLGT1	0.87082713	0.002242833
Op05g00255	Op7DLGT1	0.870788309	0.002245104
Op03g00635	OpCYP716E111	0.870635199	0.002254077
Op03g01599	OpSLS1	0.870549187	0.002259129
Op05g01931	OpIS1	0.870395542	0.002268172
Op10g00377	OpSLS1	0.870284215	0.00227474
Op07g00321	OpSLS1	0.870118717	0.002284529
Op04g00528	OpIO1	0.869868822	0.002299364
Op07g00071	OpTDC1	0.869379157	0.002328627
Op04g00296	Op7DLH1	0.869008854	0.002350926
Op02g02182	OpCYP716E111	0.868683621	0.002370633
Op10g00723	OpIO1	0.868608025	0.002375229
Op07g00141	Op10HG01	0.86854188	0.002379257
Op05g01639	OpIS1	0.868139736	0.002403842
Op10g01520	Op7DLH1	0.867880121	0.002419807
Op01g00583	Op7DLH1	0.867704019	0.002430678
Op11g01129	OpIS1	0.867684444	0.002431888
Op02g00565	OpSLS1	0.867681863	0.002432048
Op07g01431	Op7DLH1	0.867525757	0.002441716
Op02g02156	OpIS1	0.867276762	0.002457192
Op05g01852	OpIS1	0.867026408	0.002472821
Op07g01652	OpIO1	0.866846671	0.002484083
Op08g00805	OpG10H1	0.866836519	0.002484721
Op07g01056	Op7DLH1	0.866794443	0.002487363
Op04g01692	Op7DLH1	0.866627929	0.002497838
Op01g00846	OpCYP716E111	0.866414146	0.002511331
Op02g00265	OpCYP716E111	0.866056122	0.002534041
Op05g01861	OpSLS1	0.865786963	0.002551207
Op01g01891	OpSLS1	0.865180124	0.002590203
Op03g02173	Op10HG01	0.865012024	0.002601078
Op03g01392	Op7DLGT1	0.864784966	0.002615816
Op03g02254	OpSTR1	0.864777596	0.002616296
Op01g01627	OpSLS1	0.864626328	0.002626148
Op09g00192	OpIS1	0.864593083	0.002628316
Op08g01016	OpIS1	0.864387256	0.002641771
Op06g00359	OpIO1	0.864317882	0.002646316
Op06g00009	OpG10H1	0.864110708	0.002659923
Op08g01717	OpG10H1	0.864052553	0.002663751
Op10g00933	OpG10H1	0.863841443	0.002677679
Op05g00511	OpSLS1	0.863617008	0.002692541
Op10g01526	OpCYP716E111	0.863359715	0.002709649
Op05g01852	Op10HG01	0.863078016	0.002728466
Op06g00074	Op7DLH1	0.862948724	0.002737132
Op02g00436	OpIO1	0.862930883	0.002738329
Op01g02158	OpIO1	0.862668505	0.00275598
Op01g00452	OpIO1	0.862525933	0.002765604
Op06g00531	OpSLS1	0.862348048	0.002777644
Op01g01833	OpSLS1	0.861808961	0.002814352

Op03g02198	OpSLS1	0.861751868	0.002818259
Op09g00547	OpIS1	0.861683059	0.002822973
Op06g00009	OpTDC1	0.86153968	0.002832813
Op09g00763	OpG10H1	0.861439438	0.002839707
Op10g01500	OpSLS1	0.861108789	0.002862527
Op06g00159	OpIO1	0.860969017	0.002872211
Op08g00190	OpCYP716E111	0.860951611	0.002873419
Op02g00786	OpSLS1	0.860693922	0.002891337
Op11g00704	Op7DLH1	0.860617948	0.002896635
Op07g00458	OpSLS1	0.860286791	0.002919805
Op01g02250	OpSLS1	0.86018374	0.00292704
Op08g00805	OpTDC1	0.859987194	0.002940876
Op03g01115	OpSLS1	0.859748513	0.002957737
Op03g00914	OpLAMT1	0.859606765	0.002967782
Op08g01717	OpSTR1	0.859564177	0.002970805
Op04g01029	Op7DLH1	0.85940605	0.002982047
Op05g01753	Op7DLH1	0.85926238	0.002992286
Op06g00700	OpIS1	0.859095047	0.003004242
Op07g01674	Op7DLH1	0.858832401	0.003023074
Op06g00009	OpSTR1	0.858753976	0.003028713
Op05g00255	OpSLS1	0.858701346	0.003032501
Op02g00053	OpSLS1	0.858498962	0.003047099
Op02g01377	OpIS1	0.858114009	0.003074999
Op03g02197	OpSLS1	0.858078135	0.003077608
Op11g01151	OpSLS1	0.85797784	0.00308491
Op03g00720	Op7DLH1	0.857788439	0.003098733
Op01g00643	Op7DLH1	0.857539907	0.003116934
Op07g00762	OpG10H1	0.857258669	0.00313762
Op07g00762	OpTDC1	0.856962301	0.003159521
Op02g01603	OpSLS1	0.856595238	0.003186791
Op09g00537	OpIS1	0.856362623	0.003204156
Op04g01272	OpCYP716E111	0.856256801	0.003212078
Op03g01448	OpSLS1	0.856237875	0.003213496
Op11g01093	OpSLS1	0.855984106	0.003232553
Op08g00805	OpIS1	0.855867743	0.003241317
Op10g00249	Op7DLH1	0.855864811	0.003241538
Op05g00575	OpSLS1	0.855401727	0.00327658
Op01g01475	OpCYP716E111	0.855277558	0.00328602
Op01g00474	Op10HGO1	0.85526471	0.003286998
Op05g01954	Op7DLH1	0.854728982	0.00332795
Op07g01235	Op7DLGT1	0.854422319	0.003351549
Op02g01676	Op7DLH1	0.854349022	0.003357207
Op11g00249	OpSLS1	0.854249826	0.003364874
Op09g00763	OpSTR1	0.85412605	0.003374457
Op09g00730	Op7DLH1	0.853982125	0.003385625
Op02g01947	OpSLS1	0.853858237	0.003395258
Op06g01421	OpIO1	0.853274052	0.003440936
Op01g00524	OpSLS1	0.853170523	0.003449074
Op06g00618	OpCYP716E111	0.85299137	0.00346319
Op07g00526	OpSLS1	0.852681966	0.00348766
Op08g01706	OpSLS1	0.852615294	0.003492949
Op06g00624	OpCYP716E111	0.852523067	0.003500274
Op09g00800	OpSLS1	0.852390466	0.003510824
Op11g00177	Op7DLH1	0.852159927	0.003529217
Op08g01717	OpTDC1	0.852155728	0.003529553
Op02g02182	OpSLS1	0.852027616	0.003539804
Op04g01543	Op7DLGT1	0.852015272	0.003540793
Op10g00849	OpSLS1	0.851933342	0.00354736
Op06g00688	Op7DLH1	0.8518857	0.003551183
Op07g00525	Op7DLGT1	0.851652781	0.003569913
Op08g00362	OpIS1	0.851607904	0.00357353
Op02g02182	Op7DLGT1	0.851569433	0.003576632

Op04g00438	OpSLS1	0.851535645	0.003579359
Op01g00545	OpSLS1	0.851337606	0.003595366
Op07g01748	OpCYP716E111	0.850742288	0.003643783
Op03g00317	OpSLS1	0.850463136	0.00366664
Op03g01395	OpLAMT1	0.850391363	0.003672532
Op08g01598	Op7DLH1	0.850323492	0.00367811
Op04g00146	Op10HG01	0.850299597	0.003680076
Op08g00330	Op7DLH1	0.850174313	0.003690391
Op06g00445	OpIO1	0.850141261	0.003693116
Op08g00339	OpIS1	0.850082793	0.00369794
Op04g00241	OpIO1	0.850069356	0.003699049
Op01g02104	OpCYP716E111	0.850053311	0.003700374
Op08g00362	Op7DLGT1	0.849932214	0.003710382
Op08g01153	Op7DLGT1	0.849797356	0.00372155
Op02g00329	OpCYP716E111	0.849763647	0.003724345
Op10g00933	OpTDC1	0.849756989	0.003724897
Op10g00933	OpSTR1	0.849421869	0.003752766
Op07g00296	Op7DLH1	0.849215433	0.003770004
Op03g00098	OpCYP716E111	0.849027261	0.003785764
Op02g00329	Op7DLGT1	0.84882769	0.003802529
Op01g01085	OpG10H1	0.848805965	0.003804357
Op11g01157	Op7DLH1	0.848687195	0.003814361
Op04g00332	OpSLS1	0.848623249	0.003819755
Op09g01109	Op7DLH1	0.848510371	0.003829289
Op02g00436	Op10HG01	0.84806061	0.00386744
Op03g02254	OpTDC1	0.848003063	0.00387234
Op09g00763	OpTDC1	0.847815209	0.003888366
Op03g02254	OpG10H1	0.847592711	0.003907405
Op10g01423	OpIS1	0.847079229	0.003951589
Op07g01430	OpIS1	0.847065591	0.003952767
Op11g00862	Op7DLH1	0.84683908	0.00397237
Op08g00966	OpSLS1	0.846782245	0.003977299
Op06g00396	Op7DLGT1	0.846741247	0.003980858
Op01g01115	OpSLS1	0.846629471	0.00399057
Op02g00433	OpSLS1	0.846573094	0.003995475
Op05g00668	OpLAMT1	0.846551654	0.003997341
Op03g01579	Op7DLH1	0.846338173	0.004015957
Op05g00553	OpSLS1	0.846199742	0.004028061
Op02g02090	Op7DLGT1	0.846176325	0.00403011
Op01g01266	OpSLS1	0.845864627	0.004057464
Op04g00528	OpIS1	0.845541241	0.004085977
Op05g00345	Op10HG01	0.845316384	0.004105884
Op03g00024	OpCYP716E111	0.845193445	0.004116796
Op03g02173	OpLAMT1	0.845165695	0.004119262
Op08g00813	OpIS1	0.845100578	0.004125052
Op10g00005	OpIS1	0.845050796	0.004129483
Op03g00559	Op7DLGT1	0.844870387	0.004145565
Op04g00036	Op7DLH1	0.844592783	0.004170397
Op07g01430	Op10HG01	0.844558288	0.004173489
Op11g00453	OpSLS1	0.843771477	0.004244457
Op10g01256	Op7DLH1	0.843576178	0.0042622
Op06g01421	OpLAMT1	0.843442051	0.004274415
Op07g01652	Op10HG01	0.842946287	0.004319771
Op03g00138	OpSLS1	0.842841131	0.004329434
Op10g00005	OpSLS1	0.842775424	0.004335479
Op03g00635	OpSLS1	0.842718347	0.004340735
Op04g01455	Op10HG01	0.842629174	0.004348955
Op01g01085	OpSTR1	0.842560691	0.004355276
Op06g01072	OpSLS1	0.842442346	0.004366213
Op09g01475	Op7DLH1	0.842204648	0.004388236
Op02g01896	OpIO1	0.841690345	0.004436148
Op03g00914	Op10HG01	0.841555019	0.004448814

Op10g00933	OpIS1	0.84154956	0.004449326
Op05g01954	OpSLS1	0.84101548	0.00449956
Op09g00668	Op7DLH1	0.84098176	0.004502745
Op02g01436	Op7DLH1	0.840915778	0.004508981
Op10g01526	OpIS1	0.840902079	0.004510276
Op03g00400	OpLAMT1	0.840886115	0.004511786
Op05g00428	OpSLS1	0.840703068	0.004529124
Op10g00518	OpSTR1	0.840655479	0.004533639
Op03g00098	Op7DLH1	0.84059374	0.004539501
Op01g00452	OpG10H1	0.840500689	0.004548346
Op06g00159	OpG10H1	0.840342785	0.004563383
Op08g01579	OpIO1	0.84021428	0.004575645
Op02g00432	Op7DLH1	0.840120583	0.0045846
Op02g02141	OpSLS1	0.839972539	0.004598774
Op11g00560	OpSLS1	0.839889787	0.004606709
Op02g01423	OpSLS1	0.839829383	0.004612508
Op06g00159	OpSTR1	0.839476605	0.004646472
Op10g00110	Op7DLH1	0.839360537	0.004657684
Op05g01705	OpLAMT1	0.838999315	0.004692696
Op02g01996	OpSLS1	0.838957512	0.004696759
Op08g01598	OpCYP716E111	0.838863468	0.00470591
Op11g00564	Op7DLGT1	0.838570771	0.004734466
Op01g02315	OpSLS1	0.838536332	0.004737834
Op05g00333	OpSLS1	0.838356297	0.004755466
Op06g00396	Op7DLH1	0.838323066	0.004758726
Op06g00606	OpSLS1	0.838138323	0.004776874
Op01g01913	Op7DLH1	0.838090056	0.004781623
Op10g00933	OpIO1	0.838050503	0.004785518
Op01g00542	Op7DLH1	0.837760555	0.004814132
Op02g02172	OpSLS1	0.837443458	0.004845559
Op02g00053	OpCYP716E111	0.837437148	0.004846185
Op07g01673	OpCYP716E111	0.837424001	0.004847492
Op02g01377	OpG10H1	0.837073311	0.00488242
Op10g00723	OpIS1	0.836520251	0.004937855
Op06g00734	OpSLS1	0.836469602	0.004942953
Op07g00458	OpCYP716E111	0.836420579	0.00494789
Op07g01436	OpIS1	0.836377984	0.004952184
Op03g01932	OpSLS1	0.83624488	0.004965615
Op07g02009	Op7DLGT1	0.835964661	0.004993974
Op07g02038	Op7DLH1	0.835706992	0.005020148
Op03g00710	OpLAMT1	0.835688923	0.005021987
Op03g01308	OpSLS1	0.835594211	0.005031634
Op08g00159	OpSLS1	0.835550553	0.005036085
Op05g01631	OpSLS1	0.835344117	0.005057168
Op04g01285	OpSLS1	0.835043415	0.005087986
Op01g01804	Op7DLH1	0.834991955	0.005093273
Op07g00655	OpSLS1	0.83490735	0.005101973
Op01g00643	Op7DLGT1	0.834784505	0.005114624
Op07g01620	OpSLS1	0.834756578	0.005117503
Op09g01231	OpIS1	0.834397971	0.005154569
Op02g00030	OpSLS1	0.833803804	0.005216386
Op01g01085	OpTDC1	0.833729111	0.005224192
Op01g00702	OpIS1	0.833683204	0.005228994
Op06g00700	Op7DLGT1	0.833221652	0.005277442
Op08g00460	OpCYP716E111	0.832543416	0.005349189
Op05g00722	Op7DLGT1	0.832485991	0.005355294
Op09g01542	OpIS1	0.832059559	0.005400778
Op01g01052	OpIO1	0.83203396	0.005403517
Op09g00547	OpLAMT1	0.831931479	0.005414491
Op07g00186	OpCYP716E111	0.831881278	0.005419872
Op11g00110	OpIS1	0.831668796	0.005442689
Op05g00958	OpSLS1	0.831339841	0.005478143

Op10g00441	Op7DLH1	0.831310019	0.005481365
Op03g01542	OpSLS1	0.830871858	0.005528851
Op01g00137	Op7DLGT1	0.830847664	0.005531481
Op10g01445	OpLAMT1	0.830831809	0.005533206
Op02g01989	Op7DLGT1	0.830821664	0.005534309
Op02g01571	OpSLS1	0.830795003	0.005537209
Op06g00159	OpTDC1	0.830416052	0.005578547
Op09g01475	OpSLS1	0.83038501	0.005581943
Op03g02033	OpCYP716E111	0.83013379	0.005609474
Op08g01016	OpSTR1	0.830048301	0.005618864
Op03g01601	Op7DLH1	0.829872431	0.005638215
Op05g00668	Op10HG01	0.829752901	0.005651393
Op03g02033	Op7DLGT1	0.829533202	0.005675669
Op01g00434	Op7DLH1	0.829512007	0.005678015
Op11g00564	Op7DLH1	0.829363043	0.005694519
Op04g01707	OpSLS1	0.829327363	0.005698478
Op08g00931	OpSLS1	0.829280667	0.005703661
Op11g00909	OpIS1	0.829140819	0.005719202
Op06g01360	OpSLS1	0.828947077	0.005740781
Op09g00667	Op7DLH1	0.828862735	0.005750192
Op09g01472	OpSLS1	0.82874445	0.005763409
Op01g00452	OpSTR1	0.828599458	0.005779637
Op09g00192	OpCYP716E111	0.828588301	0.005780887
Op02g00785	Op7DLH1	0.828363276	0.005806139
Op11g00453	OpCYP716E111	0.8281306	0.005832329
Op05g00553	Op7DLH1	0.827802071	0.005869445
Op09g01461	OpIS1	0.827616235	0.005890511
Op08g01016	OpG10H1	0.827397305	0.005915395
Op03g01991	Op7DLH1	0.827246798	0.005932543
Op07g01498	OpSLS1	0.827181357	0.005940009
Op05g01852	OpSTR1	0.826873235	0.005975251
Op06g00752	OpSLS1	0.826813052	0.005982151
Op07g01673	OpIS1	0.826537959	0.006013761
Op07g01430	OpLAMT1	0.825847751	0.006093569
Op05g01159	OpSLS1	0.825791467	0.006100108
Op06g00463	OpSLS1	0.825741535	0.006105914
Op07g00672	OpSLS1	0.82571383	0.006109137
Op06g00031	OpSLS1	0.825648709	0.006116717
Op08g00095	OpSLS1	0.825167196	0.006172964
Op10g00377	Op7DLH1	0.824618876	0.006237443
Op04g01692	OpCYP716E111	0.824514291	0.006249793
Op01g00452	OpTDC1	0.824316547	0.00627319
Op08g00836	OpIO1	0.824070525	0.006302382
Op09g01109	OpCYP716E111	0.823751282	0.006340401
Op02g01377	OpIO1	0.823554135	0.006363956
Op10g00086	Op7DLH1	0.823524174	0.006367541
Op10g00518	OpTDC1	0.823355891	0.006387703
Op07g01467	OpIS1	0.823151248	0.00641228
Op03g01392	Op7DLH1	0.823145136	0.006413015
Op09g00341	OpSLS1	0.822887076	0.006444101
Op04g01003	Op7DLGT1	0.822850963	0.006448459
Op10g00518	OpLAMT1	0.82278343	0.006456615
Op05g01705	OpSTR1	0.822208913	0.00652628
Op04g00146	OpG10H1	0.821920197	0.006561482
Op07g01982	OpIO1	0.821799521	0.006576234
Op05g01852	OpG10H1	0.821797631	0.006576465
Op09g00668	OpCYP716E111	0.821720227	0.006585939
Op02g01495	OpSLS1	0.821150005	0.00665602
Op03g01579	Op7DLGT1	0.821113142	0.006660568
Op06g00029	Op7DLGT1	0.820997129	0.006674894
Op06g00964	Op7DLH1	0.820936851	0.006682347
Op10g01256	Op7DLGT1	0.820884333	0.006688844

Op02g01377	OpSTR1	0.820791344	0.006700359
Op02g01377	OpTDC1	0.820779844	0.006701784
Op07g00458	Op7DLH1	0.820758845	0.006704386
Op11g00563	Op7DLGT1	0.820395038	0.006749584
Op05g01705	OpIO1	0.820221028	0.006771276
Op01g01634	OpSLS1	0.819996852	0.00679929
Op04g01489	Op7DLH1	0.819972732	0.006802309
Op05g00628	OpIS1	0.81966257	0.006841211
Op03g01235	Op7DLH1	0.819554921	0.006854748
Op03g00027	OpLAMT1	0.819354633	0.006879983
Op10g00518	OpIO1	0.819331733	0.006882872
Op02g02286	OpCYP716E111	0.818707737	0.006961919
Op11g00563	Op7DLH1	0.818634052	0.006971294
Op03g00694	Op7DLH1	0.818603027	0.006975244
Op11g01129	OpSLS1	0.818420611	0.006998498
Op10g01445	Op10HG01	0.818317655	0.007011646
Op06g00079	OpIO1	0.818247092	0.007020667
Op05g00094	OpSLS1	0.818215232	0.007024743
Op11g01151	Op7DLH1	0.818198243	0.007026917
Op08g01016	OpTDC1	0.817792643	0.007078954
Op11g00067	Op7DLH1	0.817672622	0.007094403
Op03g00057	OpIS1	0.817555923	0.007109445
Op08g01769	OpCYP716E111	0.817360311	0.007134709
Op02g01976	OpCYP716E111	0.817078599	0.007171199
Op05g00930	OpCYP716E111	0.817065505	0.007172898
Op04g00332	OpIS1	0.816920774	0.007191698
Op05g00287	OpIS1	0.816568592	0.007237583
Op07g00186	Op7DLH1	0.816208043	0.007284764
Op07g01072	OpSLS1	0.81616112	0.00729092
Op05g00707	OpSLS1	0.816146984	0.007292775
Op01g01475	Op7DLH1	0.816048514	0.007305706
Op05g01962	Op7DLH1	0.815856531	0.007330963
Op09g00645	Op7DLH1	0.815837684	0.007333446
Op07g01748	Op7DLGT1	0.815551636	0.007371196
Op03g00098	Op7DLGT1	0.814758864	0.007476509
Op03g00132	OpSLS1	0.814484333	0.007513215
Op10g00518	OpG10H1	0.814343454	0.007532098
Op01g00526	Op7DLH1	0.814077235	0.00756787
Op05g00598	OpCYP716E111	0.814049506	0.007571603
Op05g01852	OpTDC1	0.81398547	0.007580227
Op05g00910	OpLAMT1	0.813912867	0.007590014
Op05g01151	Op7DLH1	0.813894672	0.007592468
Op03g02262	OpSLS1	0.813704165	0.007618193
Op11g00862	OpSLS1	0.813602247	0.007631981
Op04g01272	Op7DLGT1	0.813522622	0.007642764
Op11g01144	OpSLS1	0.813518728	0.007643291
Op08g00159	Op7DLH1	0.813471207	0.007649732
Op02g00265	OpSLS1	0.812999198	0.007713905
Op03g01599	Op7DLH1	0.812939022	0.007722113
Op01g01993	Op7DLGT1	0.812891881	0.007728546
Op08g01638	OpSLS1	0.812354981	0.007802079
Op06g00531	Op7DLH1	0.812294584	0.007810381
Op11g00917	OpSLS1	0.812161264	0.007828726
Op02g00045	OpSLS1	0.812081475	0.00783972
Op06g00159	OpIS1	0.81168744	0.007894164
Op03g00027	OpIO1	0.811554865	0.00791254
Op08g00190	Op7DLGT1	0.811487679	0.007921863
Op02g01429	Op7DLH1	0.81112624	0.007972148
Op09g00535	OpSLS1	0.811047087	0.007983189
Op06g00023	Op7DLH1	0.810819969	0.008014926
Op03g01479	Op7DLGT1	0.810598304	0.008045984
Op04g00077	Op7DLH1	0.81037624	0.00807718

Op08g01686	Op7DLH1	0.810343096	0.008081843
Op10g00417	OpSLS1	0.810254883	0.008094263
Op05g00692	OpSLS1	0.81006839	0.008120562
Op05g01741	OpSLS1	0.810066884	0.008120775
Op03g00710	OpIO1	0.810040263	0.008124534
Op08g00071	OpSLS1	0.810011333	0.00812862
Op06g00964	OpIS1	0.809960928	0.008135743
Op01g00846	Op7DLH1	0.809732652	0.008168054
Op03g00559	Op7DLH1	0.809644698	0.008180527
Op01g01065	OpIS1	0.809462669	0.008206381
Op06g00984	Op7DLH1	0.809050268	0.00826516
Op01g00846	Op7DLGT1	0.808763228	0.00830624
Op04g00241	OpG10H1	0.808441246	0.008352484
Op08g00190	Op7DLH1	0.808048765	0.00840909
Op09g00547	OpIO1	0.808008946	0.008414847
Op02g01358	Op7DLGT1	0.807929801	0.008426299
Op04g01583	OpIS1	0.807640968	0.008468179
Op03g00640	OpSLS1	0.807555356	0.00848062
Op09g00165	OpLAMT1	0.807529565	0.00848437
Op03g02189	Op7DLH1	0.80712159	0.008543842
Op07g00105	OpIS1	0.807041252	0.008555587
Op07g00618	OpIS1	0.806727377	0.008601576
Op02g01654	Op7DLH1	0.806701693	0.008605347
Op06g00011	OpIS1	0.806670295	0.008609958
Op10g00450	Op7DLH1	0.806638064	0.008614693
Op11g00276	OpSLS1	0.806559518	0.008626239
Op05g00828	OpSLS1	0.806364427	0.008654964
Op07g00465	OpIO1	0.806274884	0.00866817
Op01g01052	OpIS1	0.806176432	0.008682705
Op02g00590	OpSLS1	0.806127276	0.008689969
Op06g00476	Op7DLH1	0.806108424	0.008692755
Op09g01461	OpCYP716E111	0.806032801	0.00870394
Op02g01970	OpIS1	0.805883413	0.008726064
Op10g00052	OpSLS1	0.805642724	0.008761788
Op09g01334	OpIO1	0.805535323	0.008777761
Op03g02009	Op7DLH1	0.805468775	0.008787669
Op09g00167	Op7DLGT1	0.805376505	0.008801417
Op03g00710	OpSTR1	0.804975564	0.00886133
Op02g00785	OpSLS1	0.804962114	0.008863344
Op02g00265	OpIS1	0.804812558	0.008885766
Op05g00628	OpCYP716E111	0.804603728	0.008917139
Op04g00483	Op7DLGT1	0.804459093	0.008938911
Op06g01204	OpSLS1	0.804436505	0.008942315
Op04g00084	OpIS1	0.804403754	0.008947251
Op09g00800	Op7DLH1	0.804327349	0.008958774
Op01g00355	Op7DLH1	0.804275635	0.00896658
Op02g02062	OpSLS1	0.804101569	0.008992885
Op10g01500	Op7DLH1	0.80402088	0.009005097
Op05g00814	OpIS1	0.803975083	0.009012033
Op08g00819	OpSLS1	0.803927571	0.009019232
Op07g02009	Op7DLH1	0.803851993	0.009030692
Op01g00082	Op7DLH1	0.803773626	0.009042586
Op07g01697	Op7DLH1	0.803709408	0.009052341
Op04g00146	OpTDC1	0.803585449	0.009071189
Op07g01153	OpSLS1	0.803333865	0.009109525
Op07g01407	OpSLS1	0.803249323	0.009122432
Op11g00347	OpIS1	0.803206444	0.009128983
Op05g00866	OpIS1	0.80311632	0.009142763
Op02g00786	Op7DLH1	0.802996235	0.009161145
Op09g01437	OpCYP716E111	0.802852562	0.009183171
Op05g01705	OpTDC1	0.802818838	0.009188346
Op04g00585	Op7DLH1	0.802790038	0.009192767

Op04g00241	OpSTR1	0.8024825	0.009240068
Op03g01308	Op7DLH1	0.802327201	0.009264016
Op04g00146	OpSTR1	0.802044792	0.009307673
Op01g01993	OpLAMT1	0.801976933	0.009318183
Op02g00933	Op10HG01	0.801883557	0.00933266
Op07g01364	Op7DLH1	0.801861881	0.009336022
Op01g01115	Op7DLH1	0.801728846	0.009356678
Op07g01207	Op7DLH1	0.801661384	0.009367164
Op01g01993	Op10HG01	0.801509874	0.009390744
Op05g00686	OpIO1	0.801486125	0.009394444
Op08g01598	Op7DLGT1	0.801245463	0.009431991
Op07g01726	OpSLS1	0.801113912	0.009452558
Op05g00553	OpIS1	0.801009076	0.00946897
Op03g01891	Op7DLH1	0.800945095	0.009478996
Op05g01530	Op7DLH1	0.800769444	0.009506556
Op05g00707	Op7DLH1	0.800703292	0.00951695
Op01g00474	OpIO1	0.800568872	0.009538093
Op05g00547	OpIO1	0.800560313	0.009539441
Op07g00352	OpSLS1	0.800302731	0.009580049
Op09g01011	OpLAMT1	0.800132455	0.009606958
Op08g01758	Op7DLH1	0.800092784	0.009613234

Table S9. Metabolomic Data from *OpAVT1-KO1* hairy root line.

Compounds	Class I	Class II	Formula	CAS	KO-1	KO-2	KO-3	EV-1	EV-2	EV-3	VIP	P-value	FDR	Fold Change
(3r,5r)-1,4-bis({[(2e)-3-(3,4-dihydroxyphenyl)prop-2-enoyl]oxy})-3,5-dihydroxycyclohexane-1-carboxylic acid*	Phenolic acids	Phenolic acids	C25H24O12	71275-40-6	8585460	8814504	9858585	1.2E+07	1.5E+07	1.3E+07	1.08788	0.0229	0.041077	0.669003437
1,3-Dicaffeoylquinic acid*	Phenolic acids	Phenolic acids	C25H24O12	30964-13-7	8267644	8891461	1E+07	1.3E+07	1.3E+07	1.2E+07	1.07454	0.02621	0.045315	0.736800282
7-hydroxyaloin	Quinones	Anthraquinone	C21H22O10	82461-12-9	716520	798016	651989	323576	230036	362929	1.10337	0.002	0.008824	2.363807035
Isofraxidin	Lignans and Coumarins	Coumarins	C11H10O5	486-21-5	1059007	826326	1055370	3271876	3184509	4121105	1.14599	0.00981	0.022547	0.278015266
Crocusatin C	Terpenoids	Monoterpenoids	C10H16O2	484033-27-4	572554	445462	755870	1994416	2390468	1597532	1.11213	0.01552	0.031045	0.296516543
Kaempferide 3,7-dirhamnoside	Flavonoids	Flavonols	C28H32O14	111128-11-1	1894109	2235473	2408586	379801	534398	551084	1.1449	0.00385	0.012754	4.462052126
Rhamnazin 3-O-β-D-glucopyranoside(Flavoyadorinin	Flavonoids	Flavonols	C23H24O12	20486-38-8	3224892	2947430	3387750	6152148	7022440	6461161	1.15117	0.00141	0.007228	0.48687076
(+)-Ajmalinol	Alkaloids	Plumerane	C20H26N2O3	73012-74-5	571767	675014	543419	3269505	3825412	3294038	1.15964	0.0028	0.010581	0.172317619
Hydroquinine	Alkaloids	Quinoline alkaloids	C20H26N2O2	522-66-7	125159	71307.8	90599.7	39543.4	73177.5	115256	0.45297	0.50961	0.556104	1.259191054
Mascaroside II	Terpenoids	Ditepenoids	C26H34O9	1583263-46-0	1122128	1302166	1195163	3466888	4007422	4791349	1.14847	0.01595	0.031612	0.295088719
Olivil Monoacetate	Lignans and Coumarins	Lignans	C22H26O8	1016974-78-9	388002	371061	398304	122980	152889	212496	1.09545	0.00891	0.021264	2.369878135
Scoparone	Lignans and Coumarins	Coumarins	C11H10O4	120-08-1	420610	307943	349608	202360	257698	178252	1.02745	0.02634	0.045442	1.689088711
5-Methoxysalicylic acid	Phenolic acids	Phenolic acids	C8H8O4	2612-02-4	2438998	2512688	2676098	7117804	6612008	7634778	1.15945	0.00276	0.01052	0.35702925
Isoscoparine*	Flavonoids	Flavones	C22H22O11	20013-23-4	156219	193648	263037	524374	592801	556037	1.11601	0.00147	0.007425	0.366303705
7,8-Dihydro-4,7-Megastigmadiene-3,6,9-Triol 9-O-β-D-Glucopyranoside	Terpenoids	Sesquiterpenoids	C19H34O8	177261-75-5	6920809	9704999	8915007	4604623	4754627	4617233	1.10035	0.0428	0.066985	1.827413647
Loganin*	Terpenoids	Monoterpenoids	C17H26O10	18524-94-2	3.3E+07	4.4E+07	3.4E+07	1.7E+07	2.2E+07	2E+07	1.09801	0.02834	0.047812	1.877507136
Oleoside	Terpenoids	Monoterpenoids	C16H22O11	178600-68-5	4947272	4512562	4580221	4922392	5346247	5249446	0.92796	0.05741	0.085215	0.904754326
Scyphiphin B1	Terpenoids	Monoterpenoids	C11H16O5	1029386-87-5	4152965	3979180	4357182	1523251	2131875	1521838	1.12727	0.00164	0.007846	2.412480728
N-Methylisoleucine	Amino acids and derivatives	Amino acids and derivatives	C7H15NO2	39554-61-5	37664.2	45792.1	23939.9	30724.6	31347	22544.7	0.51211	0.36233	0.413137	1.269213894
Methyl L-pyroglutamate	Amino acids and derivatives	Amino acids and derivatives	C6H9NO3	4931-66-2	453346	384294	350983	127027	155716	128608	1.14496	0.00847	0.020738	2.889555477
Obaberine	Alkaloids	Alkaloids	C38H42N2O6	1263-80-5	6974131	8012764	7403305	2.8E+07	3.4E+07	3.2E+07	1.15962	0.00428	0.01357	0.237183552
Jasminoside C	Terpenoids	Monoterpenoids	C16H24O7	214125-05-0	147179	171063	273963	153601	247682	193657	0.05893	0.98571	0.987939	0.995402863
Pedaliin*	Flavonoids	Flavones	C22H22O12	22860-72-6	702025	712130	683068	331910	327914	183345	1.07579	0.01152	0.025117	2.487308341
Tamarixetin-3-O-glucoside (Tamarixin)	Flavonoids	Flavonols	C22H22O12	27542-39-8	689212	752899	677630	293033	307361	265538	1.1563	0.00053	0.004495	2.44792896
Secoxyloganin	Terpenoids	Monoterpenoids	C17H24O11	58822-47-2	3699987	4017639	4225760	2814803	3519486	4266811	0.53834	0.40228	0.45237	1.126617606
Kingiside*	Terpenoids	Monoterpenoids	C17H24O11	25406-67-1	3512180	3588581	2779778	2320818	3211099	3297099	0.45578	0.43702	0.48649	1.119098596
N-Feruloyloctopamine	Alkaloids	Phenolamine	C18H19NO5	66648-44-0	272616	256704	296584	454551	518663	336468	0.99837	0.0881	0.123782	0.630614374
Quercetin 3-(6''-methylglucuronide):Quercetin 3-O-beta-D-glucuronide methyl ester	Flavonoids	Flavonols	C22H20O13	79543-28-5	3343376	3991034	3675122	5655656	8166625	7058484	1.09138	0.03837	0.061266	0.527257123
3',4',7-Trihydroxyisoflavone	Flavonoids	Isoflavones	C15H10O5	485-63-2	2.7E+07	2.2E+07	2.1E+07	1.3E+07	1.1E+07	1.2E+07	1.12362	0.01538	0.030806	1.992600294
10-Formyltetrahydrofolate	Alkaloids	Alkaloids	C20H23N7O7	2800-34-2	5917583	5073380	4471784	6301294	5673982	5439770	0.64601	0.26964	0.316899	0.887895921
N-Acetylindole-3-	Alkaloids	Plumerane	C11H9NO2	22948-94-3	1225787	1447883	1529040	645242	746880	661210	1.13539	0.00931	0.021748	2.046775367
L-Prolyl-L-Phenylalanine	Amino acids and derivatives	Amino acids and derivatives	C14H18N2O3	13589-02-1	179694	154846	180653	57927.6	56117.5	104708	1.05612	0.01145	0.024974	2.355131028
5-Methoxyindoleacetate	Alkaloids	Plumerane	C11H11NO3	3471-31-6	1129104	1158543	870989	474472	482727	542915	1.12468	0.02148	0.039219	2.105598091
Indole-4-Carboxaldehyde*	Alkaloids	Alkaloids	C9H7NO	1074-86-8	2056671	1966512	1987656	1184711	1364832	1216959	1.14333	0.0014	0.007186	1.595867491
3-Indoleacetonitrile	Alkaloids	Plumerane	C10H8N2	771-51-7	768382	679189	665345	319475	299700	288455	1.15578	0.00393	0.012894	2.32794876
3-(1H-Indol-3-yl)propanoate	Alkaloids	Plumerane	C11H11NO2	830-96-6	129874	150251	130148	37041.4	78442.6	58825	1.04209	0.00918	0.021584	2.353713684
cyclo-(Gly-Phe)	Amino acids and derivatives	Amino acids and derivatives	C11H12N2O2	10125-07-2	3.8E+07	4.2E+07	4E+07	1.7E+07	1.9E+07	1.9E+07	1.1577	0.00041	0.00388	2.186312166
Tryptophan	Amino acids and derivatives	Amino acids and derivatives	C11H12N2O2	54-12-6	4.3E+07	4.2E+07	3.8E+07	1.8E+07	2E+07	1.9E+07	1.15664	0.0015	0.007449	2.190196356
8-Hydroxyquinolin	Alkaloids	Quinoline alkaloids	C9H7NO	148-24-3	2795651	2592896	2639902	1602860	1766194	1498974	1.13984	0.00058	0.004708	1.649220009
Quinoline	Others	Others	C9H7N	91-22-5	713980	872423	810870	223136	344531	304393	1.12185	0.00125	0.006845	2.748976364
Gallacetophenon	Others	Ketone	C8H8O4	528-21-2	129216	110384	171348	177605	169498	169740	0.80336	0.18692	0.233167	0.795113739
3-alpha(S)-Strictosidine	Alkaloids	Plumerane	C27H34N2O9	20824-29-7	3794307	3651867	3712173	9411338	1.2E+07	1.1E+07	1.15231	0.01304	0.027255	0.336522223
Physcion 8-glucoside	Quinones	Anthraquinone	C22H22O10	23451-01-6	5767376	5794987	6752316	1.1E+07	1.2E+07	1.1E+07	1.14106	0.00129	0.006943	0.532107313
2,2-Dimethylchroman-6-carboxylic	Phenolic acids	Phenolic acids	C12H14O3	2039-47-6	1.3E+07	9311653	1E+07	2194109	2442573	2718042	1.1491	0.01902	0.035858	4.501573092
Leucylproline	Amino acids and derivatives	Amino acids and derivatives	C11H20N2O3	6403-35-6	1.1E+07	1.2E+07	1.3E+07	2975684	2868855	2898617	1.16134	0.00421	0.013452	4.098346295
7,8-Dihydroxy-4-methylcoumarin	Lignans and Coumarins	Coumarins	C10H8O4	2107-77-9	238886	293005	291994	189958	248970	220649	0.85385	0.09098	0.127024	1.249109549
Isoscopoletin	Lignans and Coumarins	Coumarins	C10H8O4	776-86-3	4583691	4188715	4381944	1.2E+07	1.5E+07	1.2E+07	1.15626	0.00805	0.020134	0.332116601

Kaempferol-3-O-(6"-galloyl)glucoside	Flavonoids	Flavonols	C28H24O15	56317-05-6	49916.2	18609.7	33105.7	409383	518470	393418	1.13567	0.00685	0.018154	0.076919565
Sinapoyl malate	Phenolic acids	Phenolic acids	C15H16O9	92344-58-6	27078.6	29945.1	28288.2	75444.7	61097.2	35346.3	0.95568	0.13234	0.174535	0.496322491
6-O-veratroyl-capalpol*	Terpenoids	Monoterpenoids	C24H30O13	56973-43-4	81240.7	67668.8	93410.6	102716	56825.6	64692.3	0.28626	0.73122	0.764459	1.080659535
Isopentenyl adenosine	Nucleotides and derivatives	Nucleotides and derivatives	C15H21N5O4	7724-76-7	165518	160717	153093	255336	255144	201865	1.07491	0.04384	0.068179	0.672887845
Phenethylamine	Alkaloids	Benzylphenylethylamine alkaloids	C8H11N	156-28-5	106410	127882	130319	340389	359724	365707	1.15597	2.7E-05	0.001133	0.342093603
Furaneol 4-glucoside*	Others	Others	C12H18O8	121063-56-7	286351	251569	259714	208426	208724	208720	1.10913	0.03203	0.052835	1.27443885
Curculigoside B	Phenolic acids	Phenolic acids	C21H24O11	143601-09-6	140152	115037	133012	52798.6	63635.4	57020.6	1.14226	0.00463	0.014164	2.238055337
4A,5-Dimethyl-3-((E)-1-((2R,3R,4S,5S,6R)-3,4,5-Trihydroxy-6-(Hydroxymethyl)Tetrahydro-2H-Pyran-2-Yloxy)Prop-1-En-2-Yl)-4,4A,5,6,7,8-Hexahydronaphthalen-2(3H)-	Terpenoids	Sesquiterpenoids	C21H32O7	1312185-70-8	1399835	1112911	1624259	130918	81333.4	108490	1.1539	0.01265	0.026584	12.89825508
9-Glucopyranosyloxysolanascone*	Terpenoids	Sesquiterpenoids	C21H32O7	125537-96-4	1155760	1075813	1051160	110613	86653	74137	1.15959	0.00033	0.003491	12.0954145
1-Ethoxycarbonyl-	Alkaloids	Plumerane	C14H12N2O2	72755-19-2	6965448	6265012	6714519	2614569	2729802	3053106	1.15536	0.00026	0.003032	2.375115605
D-Pantothenic Acid 4'-O-Beta-Glucoside	Others	Vitamin	C15H27NO10	29588-37-2	772363	701281	653165	1952646	1929204	1784932	1.15686	0.00013	0.00213	0.375311675
O-beta-D-Glucosylzeatin	Alkaloids	Alkaloids	C16H23N5O6	56329-06-7	737044	647095	587562	1780462	1640714	1817473	1.15153	0.00013	0.00213	0.376375983
Fortuneanoside E	Others	Others	C20H24O10	908608-56-0	124133	153220	156356	1281506	1088202	1156426	1.16087	0.00232	0.009609	0.122998548
Pantothenol	Others	Vitamin	C9H19NO4	16485-10-2	60650.7	83787	56290.1	68135.8	37886.1	72782	0.32718	0.62779	0.668576	1.122614523
N6-(2-Hydroxyethyl)adenosine*	Nucleotides and derivatives	Nucleotides and derivatives	C12H17N5O5	4338-48-1	924060	953627	979466	522627	673898	734357	1.03477	0.03263	0.053537	1.479713829
Nonaethylene glycol	Others	Alcohol compounds	C18H38O10	3386-18-3	208761	326866	194333	191415	131815	118819	0.87545	0.13545	0.177877	1.651309088
Jasminoside T	Terpenoids	Monoterpenoids	C21H34O11	1423752-90-2	2252700	2424158	2283697	3205118	3339115	3967386	1.10321	0.03204	0.052835	0.66217719
Swertiajaponin*	Flavonoids	Flavones	C22H22O11	6980-25-2	192207	247469	209767	286220	405967	328782	1.01646	0.05342	0.080224	0.63610465
Tributyl citrate	Organic acids	Organic acids	C18H32O7	77-94-1	1337875	1290958	1438616	1172726	1023256	1220742	0.95151	0.04709	0.072218	1.190452722
Chrysin-7-O-glucoside*	Flavonoids	Flavones	C21H20O9	31025-53-3	5.7E+07	6E+07	5.4E+07	2.2E+07	2.3E+07	2.5E+07	1.15652	0.00034	0.003531	2.457289902
Chrysin-5-O-glucoside (Toringin)*	Flavonoids	Flavones	C21H20O9	1329-10-8	7.2E+07	7.6E+07	7.6E+07	2.7E+07	2.9E+07	2.8E+07	1.16235	5.7E-05	0.00145	2.642536889
7,8-Dihydroxyflavon	Flavonoids	Flavones	C15H10O4	38183-03-8	1102810	963527	976948	226009	256862	278501	1.15827	0.00144	0.007331	3.997107825
2'-O-Methyladenosine	Nucleotides and derivatives	Nucleotides and derivatives	C11H15N5O4	2140-79-6	1936860	1535005	1897688	246702	249973	345451	1.15315	0.00483	0.014557	6.376186101
heliocurassavicin	Alkaloids	Pyrrole alkaloids	C15H27NO4	82354-34-5	175291	163272	154154	159901	172613	162281	0.06439	0.92932	0.940719	0.995799155
Pantothenate	Others	Vitamin	C9H17NO5	79-83-4	1.8E+07	1.5E+07	1.6E+07	9248373	1.1E+07	1.1E+07	1.11198	0.00884	0.021231	1.584271338
L-Adrenaline	Alkaloids	Alkaloids	C9H13NO3	51-43-4	569959	427011	437742	312908	376845	324779	0.98379	0.07623	0.109209	1.414161522
Vesperol	Terpenoids	Monoterpenoids	C10H14O2	204978-67-6	441905	453804	459059	554525	425015	335700	0.19931	0.85512	0.877039	1.030053669
Gliocladinin B	Others	Others	C26H28O10	956094-01-2	1.7E+07	1.8E+07	1.7E+07	1.1E+07	1.5E+07	1.1E+07	0.99186	0.06105	0.089804	1.367455486
Quercetin-7-O-(6"-malonyl)glucosid	Flavonoids	Flavonols	C24H22O15	98767-37-4	26845.7	57205.2	92876.8	42932.7	24233.4	21033.5	0.69087	0.25747	0.304911	2.005990175
4-	Terpenoids	Monoterpenoids	C10H14O5	1010795-27-3	4131243	4104546	4133661	585840	634648	721591	1.16226	6E-05	0.001457	6.3691789
11-Hydroxy-3-Eudesmen-2-one 11-O-β-D-Glucopyranoside	Terpenoids	Sesquiterpenoids	C21H34O7	643002-99-7	841024	853405	996287	926030	1236870	906923	0.56142	0.36687	0.417242	0.876505675
20-nor-cofaryloside I	Terpenoids	Ditepenoids	C20H30O4	2214215-17-3	234343	268194	348334	323869	441065	367227	0.82294	0.12301	0.164319	0.751546368
3-Hydroxy-1-methoxy-9,10-anthraquinone	Quinones	Anthraquinone	C15H10O4	28504-24-7	3420616	2821366	3606897	2548425	3052454	2862641	0.73437	0.18696	0.233167	1.163685988
P-Menth-5-Ene-1,2-diol 1-O-β-D-	Terpenoids	Monoterpenoids	C16H28O7	499155-84-9	527614	494478	346036	926533	873139	1013066	1.08907	0.00308	0.011188	0.486404461
L-Phenylalanine	Amino acids and derivatives	Amino acids and derivatives	C9H11NO2	63-91-2	1.4E+07	1.6E+07	1.6E+07	5374547	5628035	5349281	1.15927	0.00441	0.013769	2.851907598
3-Hydroxy-9,10-Dinor-6-Megastigmen-8,5-olide O-β-D-Glucopyranoside	Terpenoids	Monoterpenoids	C17H26O8	82395-89-9	1.7E+07	1.8E+07	1.7E+07	8817457	8517074	9871835	1.15339	0.00019	0.002553	1.939643022
Iridolinaroside	Terpenoids	Monoterpenoids	C16H22O9	168074-87-1	9139510	8499426	8586794	3815156	3822232	4751497	1.14196	0.00057	0.004659	2.11687575
Eupenicisirenin	Terpenoids	Monoterpenoids	C10H12O4	1636133-62-4	1007733	924432	728785	379810	347693	375151	1.1358	0.02306	0.04129	2.413222417
Brasoside*	Terpenoids	Monoterpenoids	C16H22O9	70980-41-5	2.4E+07	2.7E+07	2.6E+07	1.2E+07	1.2E+07	1E+07	1.15197	0.00032	0.003485	2.301025642
Tomenin*	Lignans and Coumarins	Coumarins	C17H20O10	28446-08-4	552895	595353	469926	657216	668551	562055	0.76614	0.14699	0.190386	0.857164805
Umckalin (7-hydroxy-5,6-dimethoxycoumarin)	Lignans and Coumarins	Coumarins	C11H10O5	43053-62-9	3041478	3348377	3239855	3688267	4012638	3909462	1.08177	0.00741	0.019178	0.829406124
7-Hydroxy-4-chromone	Others	Chromone	C9H6O3	59887-89-7	196370	166430	139490	1760556	1985537	1884263	1.16017	0.0008	0.005565	0.089211093
19-epi-Ajmalicine*	Alkaloids	Plumerane	C21H24N2O3	25532-45-0	101036	125294	123310	333925	344560	463311	1.13955	0.02078	0.038272	0.306219576
Akuammigine*	Alkaloids	Plumerane	C21H24N2O3	642-17-1	138301	131535	151704	433561	469816	446330	1.16108	9.7E-05	0.001835	0.312319177
Gelsevirine*	Alkaloids	Plumerane	C21H24N2O3	38990-03-3	88429.9	131150	153652	337965	458083	425553	1.1129	0.00576	0.016212	0.305526183
Coniferyl aldehyde	Others	Aldehyde compounds	C10H10O3	20649-42-7	31661.5	29854.4	54318.2	52898.9	43424.2	65151.3	0.72771	0.20892	0.25512	0.717352183
Grandidentatin	Phenolic acids	Phenolic acids	C21H28O9	15732-48-6	161968	120810	150817	76769.5	68895.1	82822.7	1.10837	0.02211	0.040003	1.897675281
L-Tyrosine methyl ester	Amino acids and derivatives	Amino acids and derivatives	C10H13NO3	1080-06-4	137868	162410	249116	169571	198761	201659	0.20807	0.86111	0.88218	0.963863286

Methyl Hydroxycinnamate	Phenolic acids	Phenolic acids	C10H10O3	80540-55-2	104977	159146	172205	230327	172149	166745	0.70117	0.20068	0.247101	0.766537117
3,6,7-Trihydroxy-12-Oleanen-27-Oic	Terpenoids	Triterpene	C30H48O5	1192706-45-8	2369027	2076107	2114852	925223	805529	1003785	1.14802	0.00076	0.005359	2.398938482
2-(2-Ethynyl-2,4-cyclopentadien-1-ylidene)ethenol	Others	Alcohol compounds	C9H6O	393561-91-6	3212398	3090717	2831502	897371	1048800	1081490	1.15601	0.00056	0.004585	3.017053959
Pumiloside	Alkaloids	Alkaloids	C26H28N2O9	126722-26-7	7.6E+07	8.2E+07	8.6E+07	9.8E+07	1.2E+08	1.1E+08	1.05333	0.03063	0.050916	0.743391138
2,6-Dımethyl-6-hydroxy-2,7-octadienyl-β-D-glucoside (Betulalbuside A; Betulabuside A)	Terpenoids	Monoterpenoids	C16H28O7	64776-96-1	301410	290754	291531	232959	340305	296808	0.14116	0.89786	0.91185	1.015658264
11-o-Galloylbergenin	Lignans and Coumarins	Coumarins	C21H20O13	82958-44-9	409954	476051	366434	119190	98419.9	148697	1.13598	0.00451	0.013912	3.419097445
5-Hydroxy-1-tetralone	Others	Ketone compounds	C10H10O2	28315-93-7	62409.1	48882.4	40342.8	62547.8	69616.5	56411.1	0.73747	0.19074	0.237152	0.804104592
Ailanindole	Alkaloids	Plumerane	C12H8N2O2	159903-51-2	49162.1	45278.3	40391	81956	53601.9	70547	0.96899	0.09126	0.127299	0.654188016
1-Methoxy-3-hydroxy-2-carbomethoxy-9,10-anthraquinone	Quinones	Anthraquinone	C17H12O6	470704-04-2	22232.6	35501.3	28319.4	30904.2	22405.4	34974	0.08226	0.89583	0.911126	0.974738313
4-Coumarate	Phenolic acids	Phenolic acids	C9H8O3	501-98-4	694142	627064	552644	506492	536928	413292	0.9047	0.06615	0.096352	1.286355214
Met-Gly-Met	Amino acids and derivatives	Amino acids and derivatives	C12H23N3O4S2	14486-10-3	332853	460025	401302	497545	542504	410155	0.72104	0.18599	0.232434	0.823457253
P-Menth-1-En-8-ol O-β-D-Glucopyranoside	Terpenoids	Monoterpenoids	C16H28O6	114673-99-3	569135	600351	505648	581908	714214	697671	0.84276	0.11125	0.150833	0.8401748
N,N-Dimethyl-5-methoxytryptamine	Alkaloids	Plumerane	C13H18N2O	1019-45-0	1650828	1895615	1455041	749585	622189	602038	1.13783	0.00893	0.021264	2.53392142
4,9-Dimethoxycanthin-6-one	Alkaloids	Plumerane	C16H12N2O3	1270001-72-3	390885	377511	365747	1518406	1756108	1729339	1.1621	0.00314	0.011297	0.22665383
3-O-Feruloylquinic acid*	Phenolic acids	Phenolic acids	C17H20O9	40242-06-6	2.6E+07	2.6E+07	2.5E+07	1.5E+07	1.5E+07	1.5E+07	1.16088	0.00033	0.003498	1.705299636
Gly-Pro-Arg	Amino acids and derivatives	Amino acids and derivatives	C13H24N6O4	47295-77-2	661357	657929	682032	2878165	3400624	3948746	1.15742	0.01243	0.026321	0.195679362
11-methyl-forsythide	Terpenoids	Monoterpenoids	C17H24O11	159598-00-2	744562	553511	622865	226558	179272	170579	1.14171	0.00986	0.022566	3.332595185
Uncarine A	Alkaloids	Plumerane	C21H24N2O4	6899-73-6	265762	358359	233516	430394	487464	492818	1.03162	0.02167	0.039449	0.607962366
N-(p-Coumaroyl)serotonin Glucoside	Alkaloids	Phenolamine	C25H28N2O8	76423-56-8	307536	320950	335033	180272	106290	156835	1.08176	0.00883	0.021231	2.173038036
N2-Malonyl-D-tryptophan	Amino acids and derivatives	Amino acids and derivatives	C14H14N2O5	3184-74-5	2089824	2122133	2020976	941453	967490	1128487	1.15005	0.00053	0.004514	2.052041556
Wistin	Flavonoids	Isoflavones	C23H24O10	19046-26-5	1.3E+07	1E+07	1.3E+07	2739511	2762407	3373791	1.15189	0.00779	0.01981	4.066832069
Yuanhuanin*	Flavonoids	Flavones	C22H22O11	83133-14-6	770319	535556	860318	188616	352301	210529	1.06327	0.02245	0.040524	2.882702747
Arillatose B	Phenolic acids	Phenolic acids	C22H30O14	137941-45-8	260427	306933	316319	104235	141150	121128	1.13165	0.00228	0.009524	2.411045997
Tectoridin*	Flavonoids	Isoflavones	C22H22O11	611-40-5	910047	1823107	636062	215531	329310	365135	1.01471	0.14771	0.191071	3.702533959
Hydroxy-o-tolyl-acetic acid	Organic acids	Organic acids	C9H10O3	85589-35-1	2519026	2378856	2342092	3658963	3443608	3843090	1.1442	0.00295	0.010926	0.66144697
Secologanin*	Terpenoids	Monoterpenoids	C17H24O10	19351-63-4	5946724	6181990	6505164	4438620	4682760	4573520	1.14204	0.00377	0.012637	1.360643609
Epivogeloside*	Terpenoids	Monoterpenoids	C17H24O10	118627-52-4	6744041	6439993	6775411	4247168	5066947	4532970	1.11712	0.00595	0.016555	1.441418482
Chrysoeriol-8-C-glucoside (Scoparin)*	Flavonoids	Flavones	C22H22O11	301-16-6	277063	129108	163273	107721	102361	111901	0.87748	0.20605	0.252618	1.76855316
Breyniaionoside	Terpenoids	Sesquiterpenoids	C19H32O8	823182-51-0	1.6E+07	1.7E+07	1.9E+07	1.3E+07	1.3E+07	1.4E+07	1.09234	0.0126	0.026544	1.313830094
Benzocyclobutyl-1-carboxylic acid	Organic acids	Organic acids	C9H8O2	14381-41-0	1692266	1125662	995079	1647486	1432741	1414747	0.5679	0.40348	0.453549	0.848282495
3-(4-Hydroxyphenyl)butanoic acid	Organic acids	Organic acids	C10H12O3	6739-21-5	439642	446273	403963	462502	523376	403971	0.46245	0.44292	0.492347	0.928070814
Aldosecologanin	Terpenoids	Monoterpenoids	C34H46O19	471271-55-3	4.2E+07	5.3E+07	5.3E+07	2.5E+07	2.9E+07	3E+07	1.10851	0.01813	0.034517	1.761607625
5-carboxystrictosidine	Alkaloids	Alkaloids	C28H34N2O11	34371-47-6	1.5E+07	1.5E+07	1.6E+07	1.3E+07	1.5E+07	1.2E+07	0.89377	0.11215	0.151724	1.182395889
Hydroprotopine	Alkaloids	Isoquinoline alkaloids	C20H20NO5+	128397-41-1	28622	45026.8	27062	23637.7	70685.3	64938.4	0.51234	0.31933	0.368447	0.632361585
Cycloartenol	Terpenoids	Triterpene	C30H50O	469-38-5	40780.5	41547	40600.1	27282.5	30248	34644.2	1.05199	0.03873	0.061692	1.333635202
Kingiside	Terpenoids	Monoterpenoids	C11H14O6	74848-77-4	1557588	1530066	1449951	982335	918874	1012571	1.15036	0.00025	0.002963	1.557291552
Helecin	Phenolic acids	Phenolic acids	C13H16O7	618-65-5	316530	312792	341592	196301	206294	135317	1.06239	0.01299	0.027188	1.804965872
Alpigenoside	Terpenoids	Monoterpenoids	C18H28O12	79916-78-2	1.2E+07	9801313	8965575	1.4E+07	1.1E+07	2E+07	0.81694	0.18914	0.235507	0.678048117
Apocynoside I	Terpenoids	Sesquiterpenoids	C19H30O8	358721-31-0	920854	870965	833215	576388	740579	1217577	0.21686	0.89027	0.906361	1.035702591
Tagalsin Q	Terpenoids	Ditepenoids	C18H26O2	1253641-44-9	1463275	1258900	1280080	548147	562220	521499	1.15711	0.00539	0.015602	2.452563315
Afzelechin	Flavonoids	Flavanols	C15H14O5	2545-00-8	7798490	8692133	9265358	7634693	9337896	7318590	0.37326	0.55924	0.605353	1.060301802
L-Leucyl-L-phenylalanine	Amino acids and derivatives	Amino acids and derivatives	C15H22N2O3	56217-82-4	486871	484113	535273	151632	173718	155931	1.15951	0.00066	0.004944	3.129680819
(2S)-2-{[(2E)-1-hydroxy-3-(4-hydroxy-3-methoxyphenyl)prop-2-en-1-ylidene]amino}butanedioic acid	Alkaloids	Phenolamine	C14H15NO7	177715-70-7	1.5E+07	1.7E+07	1.7E+07	749922	853366	983349	1.16254	0.00158	0.007674	18.45205008
catechin-3-O-α-L-	Flavonoids	Flavanols	C21H24O10	103630-03-1	1E+07	1.4E+07	1.4E+07	1E+07	1.4E+07	9197524	0.40988	0.50165	0.54857	1.124803241
Riboflavin	Others	Vitamin	C17H20N4O6	83-88-5	1196583	1163093	1239751	589851	646076	644374	1.15778	4.9E-05	0.001344	1.914282471
Tributyl acetylcitrate	Others	Others	C20H34O8	77-90-7	199036	206945	219190	261705	217063	249664	0.89467	0.10719	0.145961	0.858241793
Multinoside A*	Flavonoids	Flavonols	C27H30O16	59262-54-3	182047	215557	136558	119993	58764.4	130445	0.83636	0.07911	0.112785	1.727554654
Plantarenaloside*	Terpenoids	Monoterpenoids	C16H24O9	72396-01-1	306045	307291	234646	217350	377492	260554	0.03327	0.96612	0.971131	0.991331183
Quercetin-7-O-rutinoside*	Flavonoids	Flavonols	C27H30O16	147714-62-3	149477	102916	214439	89957.9	107114	72139.4	0.8523	0.17026	0.215914	1.734073034
Nicotinic Acid Methyl Ester(Methyl Nicotinate)	Alkaloids	Pyridine alkaloids	C7H7NO2	93-60-7	285917	210999	172512	123445	124502	165105	0.93626	0.10929	0.148437	1.620685802

8-Hydroxy-3,4-dihydro-2H-1-benzopyran-2-	Lignans and Coumarins	Coumarins	C9H8O3	20974-72-5	3721701	3959037	3923085	3299605	3293316	3028678	1.09366	0.00519	0.015188	1.206018201
Rutin Trihydrate	Flavonoids	Flavonols	C27H36O19	207671-50-9	112228	83045.3	114274	68546.8	92140.5	41263.6	0.78546	0.12363	0.164948	1.532786894
Eucomoside B	Terpenoids	Monoterpenoids	C25H31NO11	951672-66-5	864987	856780	702613	711463	743661	858499	0.2933	0.62239	0.663512	1.047872145
3-Hydroxy-3,7-dimethyl-2-benzofuran-1(3H)-one	Others	Others	C10H10O3	7335-82-2	594279	624947	767683	689272	594926	469595	0.50153	0.40407	0.454046	1.132921101
cis-Zeatin-O-glucoside	Alkaloids	Alkaloids	C21H31N5O10	125225-72-1	93398.7	94180.6	121406	529629	670954	489386	1.15225	0.01222	0.025994	0.18283522
3β-Isodihydrocadambine*	Alkaloids	Plumerane	C27H34N2O10	62014-69-1	8898486	9934701	1.2E+07	1764119	1759163	1577960	1.15744	0.01188	0.02558	6.073872574
γ-Glu-Trp	Amino acids and derivatives	Amino acids and derivatives	C16H19N3O5	66471-20-3	157861	194330	256380	96534.8	57008.8	83275.2	1.06387	0.0356	0.057466	2.569775541
2-Ethylpyrazine	Alkaloids	Alkaloids	C6H8N2	13925-00-3	1293228	1087047	911166	596727	563713	781227	1.02634	0.03435	0.055817	1.695163053
6-	Others	Chromone	C10H10O4	70901-60-9	4520993	4290725	4142596	2079187	1824547	2166338	1.15205	0.00011	0.00202	2.134128323
loganetin*	Terpenoids	Monoterpenoids	C11H16O5	29748-10-5	3.6E+07	3.9E+07	3.3E+07	1.8E+07	1.6E+07	2.4E+07	1.08396	0.00571	0.016158	1.863501006
1-Epidihydrocornin Aglycone*	Terpenoids	Monoterpenoids	C11H16O5	145512-33-0	3.7E+07	3.9E+07	3.7E+07	1.9E+07	1.9E+07	2.5E+07	1.10499	0.01018	0.023022	1.765392078
Chokolic Acid A	Terpenoids	Monoterpenoids	C12H20O4	125564-56-9	2.1E+07	2.3E+07	2.1E+07	1.1E+07	1.1E+07	1.5E+07	1.08933	0.01043	0.023308	1.760935053
Allyl salicylate	Phenolic acids	Phenolic acids	C10H10O3	10484-09-0	6022149	4309942	4543176	2982921	3523133	2548891	1.01451	0.0486	0.074097	1.642778336
Penstemonoside	Terpenoids	Monoterpenoids	C17H26O10	81203-56-7	434257	512880	698699	440219	404172	302941	0.81506	0.15646	0.200471	1.434490387
Cornolactone C*	Terpenoids	Monoterpenoids	C11H16O5	1622866-98-1	3.5E+07	3.5E+07	3.4E+07	1.5E+07	1.6E+07	1.1E+07	1.12653	0.00517	0.015188	2.53896164
6-Hydroxykaempferol-3,6-O-diglucoside	Flavonoids	Flavonols	C27H30O17	142674-16-6	78794.4	83712.9	82320.6	64095.3	38784	65629.1	0.88376	0.09596	0.132838	1.452912358
Wushanicariin	Flavonoids	Flavonols	C27H30O11	115516-53-5	2301074	2753100	2925379	3079884	3128436	2986241	0.8262	0.15603	0.200412	0.86785582
Shionoside A	Terpenoids	Monoterpenoids	C21H36O10	114892-58-9	1092426	1088215	1148924	1859249	2298511	2253349	1.13925	0.01645	0.032232	0.519343004
Picrocrocinic	Terpenoids	Monoterpenoids	C16H26O8	62218-53-5	2339355	2279582	2555009	3757633	4311391	3947862	1.14152	0.00309	0.011205	0.596988755
Jasminoside G	Terpenoids	Monoterpenoids	C16H26O8	871589-35-4	2329266	2113718	2121089	3496282	4000427	3993663	1.1428	0.0043	0.013597	0.571267237
Acetylpyrazine	Alkaloids	Alkaloids	C6H6N2O	22047-25-2	644830	536735	529461	277952	401385	498123	0.86019	0.08905	0.124889	1.453149749
Val-Trp	Amino acids and derivatives	Amino acids and derivatives	C16H21N3O3	24587-37-9	67150.9	60464.2	92750.2	43832.2	28175.1	45377.1	0.98491	0.05246	0.079087	1.877296199
Hedyotoside	Terpenoids	Monoterpenoids	C17H22O10	210109-25-4	2109371	1339291	844187	368845	500002	602485	1.0056	0.12068	0.161774	2.917660143
N-	Alkaloids	Plumerane	C12H14N2O2	1210-83-9	189163	164400	215357	213521	142509	244046	0.11049	0.77735	0.805697	0.94808013
Ile-Trp	Amino acids and derivatives	Amino acids and derivatives	C17H23N3O3	13589-06-5	221809	241141	213482	143700	111339	130644	1.1186	0.00159	0.007697	1.753857855
Lamiuamplexoside A	Terpenoids	Monoterpenoids	C17H24O11	1195409-53-0	3805532	4533217	3981127	3373999	3023525	3849156	0.85027	0.10077	0.138693	1.202328549
Ononin*	Flavonoids	Isoflavones	C22H22O9	486-62-4	9900216	1.1E+07	1.2E+07	1.5E+07	1.6E+07	1.7E+07	1.08121	0.00899	0.021308	0.703069332
Isofraxetin	Lignans and Coumarins	Coumarins	C10H8O5	50656-75-2	746631	969780	898150	559778	534382	524436	1.10281	0.03429	0.055763	1.615327659
Uncinoside B	Others	Others	C20H24O10	474104-96-6	1630439	1451794	1687482	2282796	2776746	2662155	1.11236	0.01083	0.023878	0.617702976
Apigenin 7,4'-diglucoside	Flavonoids	Flavones	C27H30O15	31737-50-5	150432	121580	189802	90229	174137	118359	0.47758	0.45318	0.502848	1.206649075
5-(2-Hydroxyethyl)-4-methylthiazole	Others	Others	C6H9NOS	137-00-8	482346	400788	323297	424652	414916	412924	0.24476	0.77024	0.799665	0.963224423
methyl 2-(4-methoxyphenyl)-2-oxoacetate	Others	Others	C10H10O4	32766-61-3	1485170	1475806	1544868	1.1E+07	1.3E+07	1.4E+07	1.16092	0.00867	0.021041	0.118899492
Lamiophlomiol C*	Terpenoids	Monoterpenoids	C11H14O7	143086-39-9	2484156	2576252	2343511	1.6E+07	1.9E+07	2.1E+07	1.16003	0.00928	0.021724	0.13193402
Tyr-Leu	Amino acids and derivatives	Amino acids and derivatives	C15H22N2O4	17355-10-1	312361	318897	455486	103550	122741	110651	1.13475	0.03158	0.052182	3.225313922
N-Acetyl-L-tyrosine	Amino acids and derivatives	Amino acids and derivatives	C11H13NO4	537-55-3	82372.1	76300.4	83975.8	182348	127557	162778	1.10457	0.03858	0.061546	0.513342276
Lamiolactone*	Terpenoids	Monoterpenoids	C11H14O7	1391997-21-9	2132851	2354491	2314087	1.2E+07	1.6E+07	1.7E+07	1.15722	0.01333	0.027657	0.149454184
eucomic acid	Others	Others	C11H12O6	42151-32-6	2713552	2955617	2808528	1.9E+07	1.9E+07	1.9E+07	1.16427	0.00018	0.002498	0.149971928
Sesamin	Lignans and Coumarins	Lignans	C20H18O6	607-80-7	1.9E+08	2E+08	2.1E+08	3.2E+08	3.3E+08	3.4E+08	1.15866	3.5E-05	0.001173	0.608265388
Neochlorogenic acid*	Phenolic acids	Phenolic acids	C16H18O9	906-33-2	2.1E+08	2.1E+08	2.1E+08	3.2E+08	3.3E+08	3.4E+08	1.16058	0.0027	0.010381	0.624414081
Isoscopoletin-β-D-glucoside	Lignans and Coumarins	Coumarins	C16H18O9	20186-29-2	1.9E+08	2E+08	1.9E+08	3.2E+08	3.3E+08	3.4E+08	1.16007	0.00121	0.00675	0.594246787
8-Epikingiside	Terpenoids	Monoterpenoids	C17H24O11	115729-53-8	1.7E+07	1.9E+07	1.8E+07	1.4E+07	1.3E+07	1.4E+07	1.115	0.00789	0.019937	1.288366721
5-Dehydro-8-Epimussaenoside	Terpenoids	Monoterpenoids	C17H24O10	913093-70-6	4230597	4668560	4783271	1415782	1586402	1550807	1.15942	0.00149	0.007445	3.005152251
Vogeloside	Terpenoids	Monoterpenoids	C17H24O10	60077-47-6	1569444	1796191	1752318	554861	868630	569407	1.11063	0.00187	0.008438	2.568095409
Verbenalin	Terpenoids	Monoterpenoids	C17H24O10	548-37-8	1740285	1724438	1820346	659040	848437	599863	1.13578	0.00186	0.008438	2.507933068
Quercetin 5,7,3',4'-tetramethyl ether 3-galactoside:2-(3,4-Dimethoxyphenyl)-3-(beta-D-galactopyranosyloxy)-5,7-dimethoxy-4H-1-benzopyran-4-one*	Flavonoids	Flavonols	C25H28O12	196942-96-8	181837	127129	122177	103856	120352	128490	0.6481	0.30437	0.353033	1.222412372
Prenantheside A	Terpenoids	Sesquiterpenoids	C21H30O9	112606-68-5	4834344	5238555	4357303	2710938	3535033	2675378	1.06881	0.00858	0.020877	1.617490969
6-Hydroxyswerosid	Terpenoids	Monoterpenoids	C16H22O10	423158-48-9	1.2E+07	1.1E+07	1.2E+07	5999002	7026742	5296432	1.12389	0.00131	0.007005	1.904337599
2,4,6,6-Tetramethyl-3(6H)-pyridinone	Alkaloids	Pyridine alkaloids	C9H13NO	203524-64-5	55607.8	36137.2	45268	93250.7	142440	93039.5	1.06867	0.0481	0.073405	0.416793936
Paeonin B	Terpenoids	Monoterpenoids	C16H22O9	1155280-58-2	2E+07	2.1E+07	2E+07	1.3E+07	1.2E+07	1.5E+07	1.11049	0.00519	0.015193	1.492221425
Massoniresinol; Vladinol A	Lignans and Coumarins	Lignans	C20H24O8	96087-10-4	516970	442877	467591	422220	179681	606106	0.42279	0.61564	0.656769	1.181646598
5-Hydroxyconiferyl alcohol	Others	Others	C10H12O4	1782-47-4	1.1E+07	1.1E+07	9346151	6970677	6420528	7551783	1.09875	0.00967	0.022368	1.493894272
Phe-Ala	Amino acids and derivatives	Amino acids and derivatives	C12H16N2O3	3918-87-4	74725	160122	181517	77608.3	138252	136457	0.21983	0.61206	0.654304	1.181790664
3-Succinoylpyridin	Alkaloids	Pyridine alkaloids	C9H9NO3	4192-31-8	448725	416655	356236	383037	313721	317287	0.82195	0.12357	0.16494	1.20469667

Daphnin	Lignans and Coumarins	Coumarins	C15H16O9	486-55-5	5321794	4446079	5692260	3160283	3349760	3638589	1.07955	0.02882	0.048455	1.523371065
Thr-Trp	Amino acids and derivatives	Amino acids and derivatives	C15H19N3O4	186761-42-2	250000	202555	212049	232291	198335	243543	0.09223	0.8803	0.897681	0.985810232
8-hydroxy-10-hydrosweroside*	Terpenoids	Monoterpenoids	C16H24O10	919355-73-0	5259114	5122830	6042281	2830190	4710403	3545991	0.92708	0.06321	0.092626	1.481450354
Asperulogenin	Terpenoids	Monoterpenoids	C10H12O4	1639418-29-3	1.4E+07	1.3E+07	1.2E+07	7145247	9603989	7925122	1.08501	0.00753	0.019403	1.601518795
Phenylalanylproline	Amino acids and derivatives	Amino acids and derivatives	C14H18N2O3	7669-65-0	422800	275149	288611	1847211	2248867	1875875	1.14885	0.00269	0.010381	0.165198948
Protocatechualdehyde	Others	Aldehyde compounds	C7H6O3	139-85-5	126995	111572	152905	432464	377077	321295	1.13389	0.00929	0.021724	0.34617935
3-Hydroxy-1-(4-Hydroxy-3-Methoxyphenyl) Propan-1-One	Others	Ketone compounds	C10H12O4	2196-18-1	2826783	2891858	3458463	2097026	3001549	2503426	0.72578	0.19115	0.237446	1.207195835
L-Aspartyl-L-Phenylalanine	Amino acids and derivatives	Amino acids and derivatives	C13H16N2O5	13433-09-5	536872	500294	549321	263832	286413	178217	1.09073	0.00557	0.015878	2.177858615
Ser-Trp	Amino acids and derivatives	Amino acids and derivatives	C14H17N3O4	94421-70-2	521257	493054	413211	227708	322706	308093	1.04054	0.01258	0.026544	1.662795729
5,6,7-Trimethoxycoumarin*	Lignans and Coumarins	Coumarins	C12H12O5	55085-47-7	46692.2	48105.1	42001.7	2265946	2514052	2611473	1.1642	0.0018	0.008306	0.018507677
5,6,7-trimethoxy-4H-chromen-4-one	Others	Chromone	C12H12O5	52099-25-9	58899.3	50016.6	47770.5	2433832	2736529	2886612	1.16367	0.00253	0.010036	0.019447314
6-Chloropurine	Nucleotides and derivatives	Nucleotides and derivatives	C5H3ClN4	87-42-3	4153857	3972119	3857064	3259919	4190924	3493121	0.60984	0.34146	0.391066	1.094945235
5-Methoxytryptamine	Alkaloids	Plumerane	C11H14N2O	608-07-1	933719	863906	764076	2372239	2413852	1553897	1.09937	0.0424	0.066503	0.404054538
Feruloylhistamin	Alkaloids	Phenolamine	C15H17N3O3	94848-18-7	15833.6	23792.9	7466.84	44714.4	39206.6	51119.3	0.99599	0.00928	0.021724	0.348735549
Shanzhiside	Terpenoids	Monoterpenoids	C17H26O11	29836-27-9	140831	207674	182617	314089	392625	406289	1.09158	0.0073	0.018974	0.477197005
3-Hydroxyhexadecanoic acid	Lipids	Free fatty acids	C16H32O3	2398-34-7	45449.2	41041.7	64255	108730	63826	57417.8	0.74741	0.23926	0.286521	0.655493102
Nobiletin	Flavonoids	Flavones	C21H22O8	478-01-3	40346.4	66637	46323.5	102487	50848.8	114909	0.77154	0.18033	0.226184	0.571517632
3,4-Methylenedioxycinnamaldehyde	Others	Aldehyde compounds	C10H8O3	58095-77-5	1080818	998960	1175567	1224155	1246808	1042620	0.5318	0.35776	0.408683	0.92650282
2-Linoleoylglycerol	Lipids	Glycerol ester	C21H38O4	3443-82-1	438029	418973	447354	476675	480718	477812	1.07971	0.03253	0.053507	0.908828745
1-Octadecanoyl-sn-glycero-3-phosphoethanolamine	Lipids	LPE	C23H48NO7P	69747-55-3	771143	623504	632424	416822	308051	322466	1.1002	0.00685	0.018154	1.935447699
5-Hydroxy-3,7,4'-trimethoxyflavon	Flavonoids	Flavonols	C18H16O6	15486-34-7	617030	671455	591519	312313	293156	438689	1.06924	0.01235	0.026208	1.80049889
9-Methoxycamptothecine	Alkaloids	Quinoline alkaloids	C21H18N2O5	39026-92-1	2089955	1836943	2267490	196905	193711	268591	1.15787	0.00348	0.01208	9.396736209
2,6,10,14-Phytatetraene-1,20-dial	Terpenoids	Ditepenoids	C20H30O2	79421-72-0	9231.11	24937.6	19166.6	10452.5	17446.8	16832.6	0.20095	0.61462	0.656132	1.192333856
3-Chloroaniline	Alkaloids	Alkaloids	C6H6ClN	108-42-9	367327	399922	356750	361712	353517	343030	0.72269	0.22809	0.275056	1.062120983
D-Allo-Isoleucine*	Amino acids and derivatives	Amino acids and derivatives	C6H13NO2	1509-35-9	1.2E+07	1.4E+07	1.4E+07	5390629	5624481	6054358	1.1541	0.00311	0.011248	2.341522512
Angustoline	Alkaloids	Plumerane	C20H17N3O2	40041-95-0	580300	647967	650695	454835	463499	379373	1.08157	0.0057	0.016149	1.447909447
Piperidine	Alkaloids	Piperidine alkaloids	C5H11N	110-89-4	1219197	1053335	963878	494552	569264	475868	1.13403	0.00951	0.022123	2.101997263
Cyclocolorenone	Terpenoids	Sesquiterpenoids	C15H22O	489-45-2	252961	371550	278121	215611	259883	277326	0.61827	0.30595	0.354593	1.199003249
6-Methylquinizarin	Quinones	Anthraquinone	C15H10O4	14569-42-7	2416915	3292478	3389993	944687	1040502	1088140	1.13785	0.02128	0.038914	2.960758861
Oxypeucedanin	Lignans and Coumarins	Coumarins	C16H14O5	737-52-0	2604808	2450614	2287958	42056.1	37867.6	113924	1.14503	0.00076	0.005359	37.88220313
NAD+	Nucleotides and derivatives	Nucleotides and derivatives	C21H27N7O14P2	53-84-9	3220485	3647786	2859837	1160816	1429279	1369801	1.13978	0.00774	0.019729	2.456657875
Glutamyl-glutamic acid	Amino acids and derivatives	Amino acids and derivatives	C10H16N2O7	3929-61-1	148861	124756	105220	92192.7	60917.7	81338.6	0.97694	0.04151	0.065379	1.615858628
Cytarabine	Nucleotides and derivatives	Nucleotides and derivatives	C9H13N3O5	147-94-4	372245	365686	367621	179590	194297	225933	1.13777	0.00572	0.016172	1.843142123
L-Methionine	Amino acids and derivatives	Amino acids and derivatives	C5H11NO2S	63-68-3	390677	318431	348542	302980	350085	303768	0.63598	0.27274	0.320173	1.105363562
Guanine*	Nucleotides and derivatives	Nucleotides and derivatives	C5H5N5O	73-40-5	5730440	5992061	5069517	2012968	2438170	2289068	1.14933	0.00216	0.009176	2.491321074
4-methoxybenzamide	Alkaloids	Alkaloids	C8H9NO2	3424-93-9	371745	365368	317344	158131	151964	117146	1.13432	0.0009	0.005901	2.468066122
Nicotinate	Others	Vitamin	C6H5NO2	59-67-6	968172	740676	814992	315170	290720	350005	1.14346	0.01194	0.025664	2.640290316
N-Benzylformamide*	Alkaloids	Alkaloids	C8H9NO	6343-54-0	7535611	6840455	6442341	1636755	1707014	1497305	1.16104	0.00271	0.010388	4.300369415
3-Hydroxy-L-phenylalanine*	Amino acids and derivatives	Amino acids and derivatives	C9H11NO3	587-33-7	2.7E+07	2.9E+07	2.5E+07	5907699	6265558	6175042	1.16271	0.00262	0.010195	4.419229295
Alertenone	Terpenoids	Sesquiterpenoids	C30H44O2	222735-88-8	53957.7	55585.5	55389.6	19468.1	25715.7	27879.6	1.12577	0.00518	0.015188	2.25739402
5-Hydroxyquinolin	Alkaloids	Quinoline alkaloids	C11H11NO2	578-67-6	55983.9	65797.8	66112.9	28763.8	27444.3	23252	1.14249	0.00251	0.010001	2.364642461
Uncargenin C; 3 β,6β,23-Trihydroxyolean-12-en-28-oic acid	Terpenoids	Triterpene	C30H48O5	152243-70-4	991020	944579	915843	200648	239841	261776	1.15706	2E-05	0.001026	4.060352043
2-Palmitoylglycerol*	Lipids	Glycerol ester	C19H38O4	23470-00-0	369548	183378	323644	232777	352174	184969	0.24464	0.66007	0.698394	1.138518545
7-Eperuen-15-Oic Acid	Terpenoids	Ditepenoids	C20H34O2	15770-53-3	11145.9	19827.8	11136.2	20805.7	13980.5	18385.6	0.57936	0.36058	0.411594	0.791958638
Nicotinamide	Others	Vitamin	C6H6N2O	98-92-0	964890	916106	959455	901122	865035	894816	0.98363	0.03958	0.062842	1.067448392
Teresantalol	Terpenoids	Monoterpenoids	C10H16O	29550-55-8	536314	493252	450886	1323490	1052537	637451	0.97083	0.12246	0.163666	0.491277198
Oxyanin A	Flavonoids	Flavonols	C18H16O8	549-17-7	126674	141204	146947	824845	616557	520435	1.1456	0.02857	0.048106	0.211447198
4,10,14-Aromadendranetriol 10,14-Isopropylidene	Terpenoids	Sesquiterpenoids	C18H30O3	939774-26-2	1.1E+07	9060763	8142912	2835483	2866801	2881098	1.1556	0.01192	0.025653	3.234886121
Lauramine oxide	Others	Others	C14H31NO	1643-20-5	55863	81240.4	83603.4	55510.4	76774.5	70107.8	0.2747	0.60764	0.650249	1.090488429

Andrograpanin	Terpenoids	Ditepenoids	C20H30O3	82209-74-3	127783	182430	174217	115132	150669	138358	0.62458	0.26433	0.311366	1.198615292
11,12-epoxy-13-hydroxy-3-Oxooleanane-28-oic acid gamma-lactone (Liquidambaric Lactone)	Terpenoids	Triterpene	C30H44O4	185051-75-6	89421.4	77917.3	78285.7	35154.5	31773	29301.1	1.15258	0.00177	0.008226	2.552510509
11,28-Dihydroxy-20(29)-Lupen-3-	Terpenoids	Triterpene	C30H48O3	1713280-15-9	408532	456660	453241	149535	139895	128511	1.15894	0.00084	0.005655	3.154589641
1-Monomyristin	Lipids	Glycerol ester	C17H34O4	589-68-4	945899	655825	833940	542102	677026	928103	0.40263	0.536	0.582645	1.134327997
Ehretioside B	Alkaloids	Alkaloids	C14H17NO7	156368-84-2	299945	261755	274591	64378.3	78828.6	57380.5	1.15332	0.00037	0.003678	4.169208043
Succinic anhydride	Organic acids	Organic acids	C4H4O3	108-30-5	664345	651509	599051	350745	298505	312962	1.14946	0.00032	0.003491	1.990105792
Phytosphingosin	Lipids	Sphingolipids	C18H39NO3	554-62-1	2188406	2122314	2214270	776505	664015	641884	1.15849	2.8E-05	0.001156	3.133392254
linolenoyl ethanolamine	Lipids	LPE	C20H35NO2	57086-93-8	113967	142979	117632	243392	253400	249079	1.1407	0.00282	0.010635	0.50220221
Phosphatidylcholine lyso 17:0*	Lipids	LPC	C25H52NO7P	68659-01-8	1348006	1396053	1382100	992764	921299	1063213	1.12741	0.00621	0.016997	1.38588404
Savinin	Lignans and Coumarins	Lignans	C20H16O6	493-95-8	61537.6	53904	65158.8	32064.7	43915.8	31299.8	1.05612	0.01069	0.023682	1.683444769
Batatasin I	Phenolic acids	Phenolic acids	C17H16O4	51415-00-0	74632.9	59623.5	64800.7	67807.1	66774.8	54374.7	0.30724	0.614	0.655691	1.053454154
4,7,22,24-Tetrahydroxy-3,4-Seco-11,13(18)-Oleanadien-3-	Terpenoids	Triterpene	C30H48O6	1004987-14-7	205196	282237	308066	1661521	1851772	2215140	1.15223	0.00786	0.019886	0.138868433
LPC(18:1/0:0)	Lipids	LPC	C26H53NO7P+	3542-29-8	407742	404100	412970	314846	276741	412906	0.77968	0.21148	0.257732	1.219333408
9,12-Octadecadien-6-Ynoic Acid	Lipids	Free fatty acids	C18H28O2	61481-29-6	2444985	2266381	2150679	644533	668801	590242	1.16103	0.00149	0.007449	3.604818924
2,3,23-Trihydroxy-12-Oleanen-28-Oic Acid 3-O-(4-Hydroxy-3-Methoxy-E-Cinnamoyl)	Terpenoids	Triterpene Saponin	C40H56O8	1616385-47-7	1178496	1078990	1020211	25160.5	30491.8	26695	1.16394	0.00186	0.008438	39.80339096
(9R,13R)-10,11-dihydro-12-oxo-15-phytoenoic acid	Lipids	Free fatty acids	C18H30O3	136845-14-2	622674	605921	558009	108447	97042.7	82382.7	1.15975	0.00041	0.003813	6.206223542
Acacetin*	Flavonoids	Flavones	C16H12O5	480-44-4	2278475	2443145	2334940	159036	179470	175834	1.16408	0.0004	0.003813	13.7196485
7-Oxodehydroabietic acid*	Terpenoids	Ditepenoids	C20H26O3	18684-55-4	401924	365983	372621	574864	626262	526946	1.12213	0.01212	0.025858	0.660000038
Oleamide	Lipids	Free fatty acids	C18H35NO	301-02-0	237081	266349	238357	309432	551214	344166	0.91073	0.17567	0.221348	0.615686282
Momilactone E*	Terpenoids	Ditepenoids	C19H28O2	62994-68-7	33798.8	24884.7	24461.1	53042.9	51444.2	54979.2	1.10743	0.00843	0.02067	0.521392935
12,13-Dihydroxy-7-Oxo-8(14)-Abieten-18-Oic	Terpenoids	Ditepenoids	C20H30O5	935874-69-4	1.2E+07	1.2E+07	1.1E+07	1.6E+07	1.7E+07	1.9E+07	1.12402	0.01996	0.037204	0.675257107
N-[2-[(1R,2S)-3-Oxo-2-(2Z)-2-penten-1-ylcyclopentyl]acetyl]-L-leucine	Amino acids and derivatives	Amino acids and derivatives	C18H29NO4	1272649-78-1	70486.6	93050	94857.2	85904	81029.6	69330.4	0.3986	0.47786	0.526648	1.093664679
1,6-Dihydroxy-2,7(19),10,14-Phytatetraen-13-one	Terpenoids	Ditepenoids	C20H32O3	79404-59-4	403851	467242	628428	53185.1	63679.8	80224.6	1.14702	0.02166	0.039449	7.608323177
Strictosamide*	Alkaloids	Plumerane	C26H30N2O8	23141-25-5	6.8E+07	6.9E+07	6.4E+07	8.1E+07	9E+07	9.1E+07	1.10738	0.01229	0.026136	0.768354002
Nudol	Quinones	PhenAnthraquinones	C16H14O4	86630-46-8	1923739	2252874	1987368	4638036	2809482	2161081	0.75999	0.26043	0.307688	0.641506728
LysoPC 17:0*	Lipids	LPC	C25H52NO7P	50930-23-9	1553359	1589940	1529306	1128670	1137003	1058627	1.15209	0.00024	0.002855	1.405591035
Worenine	Alkaloids	Isoquinoline alkaloids	C20H16NO4+	38763-29-0	1559293	1776646	1608372	425017	487760	478106	1.15973	0.00158	0.007674	3.554799632
3',7-dihydroxy-4'-	Flavonoids	Flavones	C16H12O5	54867-60-6	1399652	1393972	1509561	265890	245461	336975	1.15637	3.2E-05	0.001173	5.072564307
Calycosin*	Flavonoids	Isoflavones	C16H12O5	20575-57-9	1588289	1439634	1398136	238852	316108	229964	1.15592	0.00043	0.003919	5.638838595
7-Hydroxy-3-(4-hydroxybenzylidene)chroman-4-	Flavonoids	Other Flavonoids	C16H12O4	110064-50-1	664773	631090	743298	86718.5	127860	127903	1.15094	0.00106	0.00637	5.954075808
Hydroxygenkwanin	Flavonoids	Flavones	C16H12O6	20243-59-8	444458	449454	441812	99971.5	47231.1	54940.6	1.13401	0.0016	0.007733	6.60781169
delta-Guanidinovaleric acid	Organic acids	Organic acids	C6H13N3O2	462-93-1	59534.8	89880.5	83092.4	60540	42651.9	67474.7	0.74509	0.15885	0.20319	1.362350167
Adenine	Nucleotides and derivatives	Nucleotides and derivatives	C5H5N5	73-24-5	2.3E+07	2.3E+07	2.4E+07	1.9E+07	1.9E+07	1.7E+07	1.13945	0.00098	0.006139	1.289234066
Isomacedonic Acid	Terpenoids	Triterpene	C30H46O4	32772-00-2	87892.5	75819.7	58342.3	23978	24934	75999.6	0.78134	0.19228	0.238573	1.777693343
9-hydroxy-2,3-dimethoxy-1,4-anthraquinone*	Quinones	Anthraquinone	C16H12O5	184652-26-4	3443087	3515699	3654344	1725366	1766671	1944861	1.15615	4.9E-05	0.001345	1.952055719
3,5-di-tert-Butyl-4-hydroxyacetophenone	Others	Ketone compounds	C16H24O2	14035-33-7	394556	528694	423250	392705	375402	479261	0.35554	0.56083	0.606495	1.079472231
3,5-Di-tert-butylbenzoic acid	Phenolic acids	Phenolic acids	C15H22O2	16225-26-6	1985109	1994464	1959157	1591213	1774832	1692003	1.08214	0.02734	0.046608	1.174114926
4-Phenyl-1-butanol	Others	Alcohol compounds	C10H14O	3360-41-6	124429	121777	107566	1185067	933777	708789	1.15154	0.02657	0.045672	0.125112273
3β-Isodihydrocadambine 4-oxide	Alkaloids	Alkaloids	C27H34N2O11	1092371-18-0	2283945	2949380	2913544	1159519	1316944	1357586	1.12729	0.0162	0.031944	2.124873766
3-Hydroxy-3'-methoxyflavone	Flavonoids	Flavones	C16H12O4	76666-32-5	4328451	4180525	4502339	622197	681587	778839	1.16146	6.4E-05	0.001504	6.247561169
2,3,7,19-Tetrahydroxyurs-12-en-28-oic acid (Roxburic Acid)*	Terpenoids	Triterpene	C30H48O6	108657-25-6	129637	109319	109467	699390	771723	759464	1.16182	0.00054	0.004555	0.156203078

linoleoyl ethanolamine	Lipids	LPE	C20H37NO2	68171-52-8	2758882	2614366	2623564	5122980	4948282	4905166	1.16173	2.3E-05	0.001056	0.533959869
Glycidyl oleate	Lipids	Glycerol ester	C21H38O3	5431-33-4	32085.5	33360.8	35123.6	25178.9	30270.2	41091.4	0.23831	0.80332	0.829565	1.041737217
2',3',4'-Trimethoxyflavone	Flavonoids	Flavones	C18H16O5	7143-46-6	361522	363608	355469	79204.4	76708.1	75568	1.16456	4.8E-06	0.0006	4.668207996
6-Paradol	Phenolic acids	Phenolic acids	C17H26O3	27113-22-0	60368.6	22952.5	32682.9	98167.2	62182.7	64151.5	0.86993	0.08932	0.125126	0.516718086
LysoPE 18:1*	Lipids	LPE	C23H46NO7P	89576-29-4	2136978	2305128	2357103	1067159	1337093	1290052	1.12999	0.00079	0.005531	1.840457823
3,30-Dihydroxyurs-12-en-28-oic acid (Rubifolic acid)*	Terpenoids	Triterpene	C30H48O4	80489-65-2	184639	170177	175848	352927	341907	418017	1.14583	0.0126	0.026544	0.476850129
13-Methyl-8,11,13-Podocarpatriene-2,19-diol	Terpenoids	Ditepenoids	C18H26O2	2081951-14-4	185081	219675	192259	61232.6	96578.2	98874.8	1.0882	0.00231	0.009608	2.32586394
3-Hydroxyolean-12-ene-27,28-dioic acid (Cincholic acid)	Terpenoids	Triterpene	C30H46O5	5948-32-3	309709	333411	319785	143721	177441	160565	1.14485	0.00033	0.003509	1.998859103
1-Monolinolenoyl-	Lipids	Glycerol ester	C21H36O4	18465-99-1	504475	397614	519833	520061	411707	425220	0.23115	0.69548	0.730369	1.047852336
Monolinolenin*	Lipids	Glycerol ester	C21H36O4	75685-85-7	544227	400083	522019	545358	437607	417550	0.19955	0.7328	0.765672	1.046992084
Elemicin	Others	Others	C12H16O3	487-11-6	133187	95766.4	100341	94644.2	122582	115042	0.06136	0.94891	0.957567	0.99105027
Butyl isobutyl phthalate*	Phenolic acids	Phenolic acids	C16H22O4	17851-53-5	4.6E+07	3.9E+07	4.8E+07	5E+07	4E+07	4.3E+07	0.00794	0.98786	0.989453	1.001526669
Hexadecanamide	Alkaloids	Alkaloids	C16H33NO	629-54-9	1982051	1949425	1948288	2367516	2280399	2301687	1.15131	0.00183	0.008421	0.846057762
Trisporol C	Terpenoids	Sesquiterpenoids	C18H28O3	26094-65-5	3172292	2960355	3060967	1308323	1235880	1185611	1.16142	7.8E-05	0.001638	2.46489904
13-Methyl-8,11,13-Podocarpatriene-2,3-diol*	Terpenoids	Ditepenoids	C18H26O2	1801444-77-8	1190251	1132126	1179852	444365	490389	472844	1.16171	1.3E-05	0.000842	2.488087464
Aristolactam BII	Alkaloids	Alkaloids	C17H13NO3	53948-09-7	47844	44937.9	45341.1	225828	92213.2	92160.9	0.99694	0.17865	0.224441	0.336719187
Lys-Gly	Amino acids and derivatives	Amino acids and derivatives	C8H17N3O3	7563-03-3	3.8E+07	3.7E+07	3.7E+07	3.4E+07	3.3E+07	3.1E+07	1.09599	0.01234	0.026207	1.139333325
Gln-Gly	Amino acids and derivatives	Amino acids and derivatives	C7H13N3O4	2650-65-9	3.2E+07	2.9E+07	3.1E+07	2.9E+07	2.8E+07	2.8E+07	0.88464	0.12156	0.162822	1.080481688
L-Histidinol	Alkaloids	Alkaloids	C6H11N3O	501-28-0	5.8E+07	5.8E+07	5.4E+07	4.5E+07	5.4E+07	5E+07	0.89211	0.09353	0.129937	1.13910132
Vallesiachotamin	Alkaloids	Plumerane	C21H22N2O3	5523-37-5	209815	249416	238576	89373.5	69452.8	96346.6	1.13744	0.00093	0.005941	2.734645813
Tetradecyldiethanolamine	Alkaloids	Alkaloids	C18H39NO2	18924-66-8	722963	766685	811527	686580	1132873	733082	0.28072	0.6162	0.657136	0.901525189
Tomentosolic Acid	Terpenoids	Triterpene	C30H46O3	6812-98-2	147494	113270	130513	89412.8	73794.9	71031.6	1.07946	0.01684	0.032643	1.670414661
LysoPE 16:0	Lipids	LPE	C21H44NO7P	53862-35-4	2.4E+07	2.5E+07	2.7E+07	1.4E+07	1.4E+07	1.4E+07	1.15314	0.00658	0.017703	1.81365757
LysoPC 16:1*	Lipids	LPC	C24H48NO7P	76790-27-7	289140	229860	289668	220931	272259	245151	0.48161	0.4016	0.451759	1.095249612
Virgatic acid	Terpenoids	Triterpene	C30H46O4	14356-51-5	2299790	2247745	2296672	977323	1074255	1439017	1.10297	0.01443	0.029323	1.960756909
p-Methoxycinnamaldehyde	Others	Aldehyde compounds	C10H10O2	1963-36-6	611144	487737	540196	654260	542695	494279	0.15577	0.78517	0.812985	0.969160446
Geranyl acetate*	Terpenoids	Monoterpenoids	C12H20O2	105-87-3	157393	138083	166590	55615.2	52291.2	71955.7	1.1335	0.00125	0.006845	2.569000407
Schisanlactone E	Terpenoids	Triterpene	C30H44O4	136040-43-2	164654	154753	112698	33769.9	31706.7	45460.5	1.13389	0.01617	0.031918	3.895049398
N6,N6,N6-Trimethyl-L-lysine	Amino acids and derivatives	Amino acids and derivatives	C9H20N2O2	23284-33-5	554098	462464	585310	180489	210673	218468	1.14384	0.00756	0.019457	2.627610157
Aminoimidazole ribotide	Nucleotides and derivatives	Nucleotides and derivatives	C8H14N3O7P	25635-88-5	530104	502739	564289	239584	306825	356804	1.07538	0.00894	0.021264	1.768277592
Nicotinamide D-ribonucleotide	Nucleotides and derivatives	Nucleotides and derivatives	C11H15N2O8P	1094-61-7	476346	393800	450326	282171	281807	283669	1.12849	0.02302	0.041278	1.557810316
N-Acetyl-D-mannosamine	Others	Saccharides	C8H15NO6	7772-94-3	286404	192235	247608	190078	210176	172908	0.76785	0.19434	0.240535	1.267088231
Nicotinate D-ribonucleoside	Others	Vitamin	C11H14NO6+	17720-18-2	3921342	4343835	4181634	2233004	2603125	2751898	1.12169	0.00147	0.007425	1.64032253
Asn-Hyp	Amino acids and derivatives	Amino acids and derivatives	C9H15N3O5	844640-50-2	218439	218943	208601	134530	148506	143103	1.15057	0.00019	0.002573	1.515900109
Pro-Asn	Amino acids and derivatives	Amino acids and derivatives	C9H15N3O4	107856-82-6	516614	537970	540839	281119	265503	284501	1.16109	2.1E-05	0.001051	1.919598858
Cytidine	Nucleotides and derivatives	Nucleotides and derivatives	C9H13N3O5	65-46-3	314237	290426	330891	245074	266912	224442	1.03151	0.01746	0.033557	1.270395019
O-Acetyl-L-serine	Amino acids and derivatives	Amino acids and derivatives	C5H9NO4	5147-00-2	397427	348258	403108	241265	264557	267811	1.11865	0.0085	0.020793	1.484931802
4-Guanidinobutanoate	Organic acids	Organic acids	C5H11N3O2	463-00-3	460063	406818	461092	299805	243030	284245	1.11078	0.00251	0.010001	1.605615448
Isoguanine	Nucleotides and derivatives	Nucleotides and derivatives	C5H5N5O	3373-53-3	4336591	4166082	4851360	1438265	1644969	1598436	1.15655	0.00274	0.010467	2.852408184
Barbiturate	Nucleotides and derivatives	Nucleotides and derivatives	C4H4N2O3	67-52-7	1E+07	1.1E+07	1.1E+07	1.2E+07	1.1E+07	1.2E+07	0.76173	0.18092	0.226736	0.938695832
Homoproline	Amino acids and derivatives	Amino acids and derivatives	C6H11NO2	535-75-1	731328	704590	674137	628997	528278	581899	1.01334	0.03136	0.051842	1.213251069
CMP	Nucleotides and derivatives	Nucleotides and derivatives	C9H14N3O8P	63-37-6	912350	749516	767107	169373	166569	209553	1.15551	0.00429	0.01359	4.452789544
Isoguanosine	Nucleotides and derivatives	Nucleotides and derivatives	C10H13N5O5	1818-71-9	807942	824939	831936	755176	983295	897949	0.42923	0.48102	0.529561	0.934911328
Isocytosine	Nucleotides and derivatives	Nucleotides and derivatives	C4H5N3O	108-53-2	79514.3	67682.6	66913.5	49495.1	61077	66562.5	0.7948	0.1328	0.174995	1.208744011
L-Valine	Amino acids and derivatives	Amino acids and derivatives	C5H11NO2	72-18-4	3.3E+07	3.2E+07	3.3E+07	1.3E+07	1.5E+07	1.5E+07	1.15962	0.00072	0.005226	2.248827527
Pro-Asp	Amino acids and derivatives	Amino acids and derivatives	C9H14N2O5	85227-98-1	449326	464742	575033	186460	214547	189128	1.14279	0.01357	0.028069	2.523327186
Ile-Asn	Amino acids and derivatives	Amino acids and derivatives	C10H19N3O4	59652-59-4	88427.2	86914.8	104239	19082.9	21071.1	29349.9	1.14134	0.00126	0.00685	4.022521811
O-Acetyl-L-homoserine	Amino acids and derivatives	Amino acids and derivatives	C6H11NO4	250736-84-6	184784	196778	191702	85659.9	140623	122484	1.00885	0.03853	0.061497	1.643687336
2-Methylpyrrolidine-2-carboxylic acid*	Alkaloids	Alkaloids	C6H11NO2	16277-06-8	6.7E+07	6.1E+07	6.4E+07	9539837	9669100	9445386	1.16433	0.00091	0.005911	6.690372909
N,N-Dimethylglycine	Amino acids and derivatives	Amino acids and derivatives	C4H9NO2	1118-68-9	6862695	6886656	5385499	4291834	5080974	5022105	0.95693	0.06654	0.096868	1.329278605
9-Methoxycanthin-6-one N-oxide	Alkaloids	Alkaloids	C15H10N2O3	137739-74-3	49983.4	60619.3	40731.8	76750	72822.7	88395.6	1.01689	0.01897	0.035807	0.63594438
Choline	Alkaloids	Alkaloids	C5H14NO+	62-49-7	561068	421757	458399	494373	441287	479826	0.0787	0.86165	0.882448	1.01818325

5-Methylcytosine	Nucleotides and derivatives	Nucleotides and derivatives	C5H7N3O	554-01-8	165141	191800	185222	78691.7	105521	109344	1.09333	0.00305	0.011153	1.846874832
Turanose	Others	Saccharides	C12H22O11	547-25-1	1.9E+07	1.8E+07	2.1E+07	1.6E+07	1.7E+07	1.8E+07	0.84941	0.13598	0.178429	1.127275994
5-Ribofuranosylnicotinamide	Nucleotides and derivatives	Nucleotides and derivatives	C11H14N2O5	107325-67-7	353528	370793	401414	107434	129987	106761	1.15529	0.00042	0.00388	3.27075791
Betaine	Alkaloids	Alkaloids	C5H11NO2	107-43-7	853327	879737	851740	537525	503049	613976	1.12623	0.00741	0.019178	1.562240216
NG,NG-Dimethyl-L-arginine	Amino acids and derivatives	Amino acids and derivatives	C8H18N4O2	30315-93-6	1421728	1515876	1464800	428515	598262	622830	1.132	0.00127	0.006895	2.668758748
L-Lysine	Amino acids and derivatives	Amino acids and derivatives	C6H14N2O2	56-87-1	1.1E+07	1E+07	9807305	2.3E+07	2.2E+07	2.2E+07	1.16201	1.5E-05	0.000888	0.455653996
2-amino-2-(1-methyl-1H-pyrazol-4-yl)acetic acid	Alkaloids	Alkaloids	C6H9N3O2	195070-68-9	3979777	3977839	3520409	1769913	2957840	1510648	0.98247	0.04724	0.072344	1.839898383
N-Acetylputrescine	Alkaloids	Alkaloids	C6H14N2O	18233-70-0	330906	341007	389828	110315	146421	98025.4	1.13364	0.00068	0.005057	2.992832432
L-Citrulline	Amino acids and derivatives	Amino acids and derivatives	C6H13N3O3	372-75-8	60588.7	72080.2	50860.2	21937.7	46604.9	38464	0.8838	0.05658	0.084146	1.71512034
Pterolactam*	Alkaloids	Pyrrole alkaloids	C5H9NO2	38072-88-7	1.5E+07	1.3E+07	1.4E+07	3741204	4234929	4292596	1.15741	0.00412	0.013261	3.462019139
1-Amino-1-cyclobutane-carboxylic-acid	Amino acids and derivatives	Amino acids and derivatives	C5H9NO2	22264-50-2	2E+07	1.9E+07	2.1E+07	4805522	5430364	5761781	1.1601	6E-05	0.001457	3.725856476
L-Threonine*	Amino acids and derivatives	Amino acids and derivatives	C4H9NO3	72-19-5	1025469	920478	975399	449124	520181	456154	1.15077	0.0003	0.003367	2.049407841
L-Homoserine*	Amino acids and derivatives	Amino acids and derivatives	C4H9NO3	672-15-1	1005145	938475	1097577	444630	524869	507163	1.14429	0.00203	0.008886	2.05950706
Imidazol-1-yl-acetic acid*	Alkaloids	Alkaloids	C5H6N2O2	22884-10-2	966826	820305	940210	1060685	900146	1041452	0.64049	0.24854	0.295916	0.908422155
Bis(2-ethylhexyl)phthalate	Phenolic acids	Phenolic acids	C24H38O4	117-81-7	8039765	7132970	8069275	7815649	6512948	8015872	0.30774	0.62728	0.668263	1.040168358
Lauramide	Lipids	Free fatty acids	C12H25NO	1120-16-7	48675.9	125429	43010	64304.4	89689.5	48328.8	0.03063	0.87732	0.894939	1.073114253
6-Methoxy-2-(2-phenylethyl)chromone	Others	Chromone	C18H16O3	84294-89-3	46932.4	65050.1	46274.4	47516	112088	61784.9	0.52927	0.39764	0.44845	0.714837729
2-Hydroxymethylanthraquinone	Quinones	Anthraquinone	C15H10O3	17241-59-7	1406328	1762676	1992795	1152346	2046881	1267919	0.43691	0.52761	0.574528	1.155502655
L-Canavanine sulfate	Amino acids and derivatives	Amino acids and derivatives	C5H14N4O7S	2219-31-0	296980	277793	273597	290424	263099	283169	0.21132	0.73909	0.77198	1.013957313
9s,13r-12-Oxophytodienoic Acid	Lipids	Free fatty acids	C18H28O3	71606-07-0	60389.4	122097	125906	35793.1	24664.8	27627.7	1.06694	0.07105	0.102649	3.501057089
2',6'-dimethoxy-4,4'-di-hydroxychalcone	Flavonoids	Chalcones	C17H16O5	123316-64-3	104457	67453.3	113608	63693.9	146025	159367	0.34341	0.46453	0.513606	0.773583998
8-Hydroxy-4',5,7-trimethoxyflavon	Flavonoids	Flavones	C18H16O6	21919-71-1	384223	412433	453798	191953	240959	263356	1.09597	0.00322	0.011462	1.795937998
Monopalmitin*	Lipids	Glycerol ester	C19H38O4	542-44-9	231194	192769	275680	361761	355519	247328	0.81753	0.12829	0.170144	0.725311844
Genkwanin*	Flavonoids	Flavones	C16H12O5	437-64-9	1125989	1151570	1334939	96484	109688	94904.9	1.16268	0.0034	0.01192	11.99857742
Hydroxyanthraquinone	Quinones	Anthraquinone	C14H8O3	129-43-1	51638.6	36809.8	46956.8	26494.6	32366.6	28952.9	1.02273	0.05361	0.08046	1.541953498
N-Acetylcadaverine	Alkaloids	Alkaloids	C7H16N2O	32343-73-0	577879	533778	454633	524503	642077	735113	0.7172	0.20556	0.252205	0.823629057
Gly-Tyr*	Amino acids and derivatives	Amino acids and derivatives	C11H14N2O4	658-79-7	428494	474386	436102	254531	288577	295060	1.13304	0.00098	0.006139	1.597511529
Nepetin (5,7,3',4'-Tetrahydroxy-6-	Flavonoids	Flavones	C16H12O7	520-11-6	4194247	4280770	4267532	797834	844868	780461	1.16418	2E-07	0.000172	5.258643468
Tangeretin*	Flavonoids	Flavones	C20H20O7	481-53-8	42253	88549.2	28182.9	181526	48967.5	122235	0.702	0.22983	0.27651	0.450728549
L-Histidine	Amino acids and derivatives	Amino acids and derivatives	C6H9N3O2	71-00-1	5024298	4504438	4306487	3627051	3461736	3408142	1.09703	0.02662	0.045687	1.318025792
Phosphatidylcholine lyso 14	Lipids	LPC	C22H46NO7P	13699-45-1	227529	195017	194400	157625	136299	139263	1.08622	0.0139	0.028506	1.424202526
L-Methionine S-oxide	Amino acids and derivatives	Amino acids and derivatives	C5H11NO3S	3226-65-1	205394	168126	237267	202897	196511	209606	0.03911	0.97928	0.982768	1.0029137
7-(4-Hydroxyphenyl)-1-phenyl-4-hepten-3-one	Others	Ketone compounds	C19H20O2	100667-52-5	464296	517058	579029	343565	916747	519013	0.10997	0.71121	0.745878	0.876952617
Pyridoxal	Others	Vitamin	C8H9NO3	66-72-8	234227	237575	228795	178606	152161	146063	1.1094	0.01316	0.027397	1.469278662
D-Threose	Others	Saccharides	C4H8O4	95-43-2	77362.1	52476.5	54964.5	83820.3	77683.3	75645.3	0.83959	0.14904	0.192541	0.779270146
2-Phenylacetamide	Alkaloids	Alkaloids	C8H9NO	103-81-1	6653488	6668454	6960073	1470117	1733905	1580805	1.16181	4.1E-06	0.000558	4.238819225
dAMP	Nucleotides and derivatives	Nucleotides and derivatives	C10H14N5O6P	653-63-4	1.1E+07	1E+07	1.2E+07	6197052	7239986	8138600	1.07289	0.00718	0.018812	1.527264576
Psicofuranine*	Nucleotides and derivatives	Nucleotides and derivatives	C11H15N5O5	1874-54-0	7527889	6751049	6208234	3782384	4168794	4203682	1.13037	0.01131	0.024735	1.685512873
8-Azaguanine	Nucleotides and derivatives	Nucleotides and derivatives	C4H4N6O	134-58-7	592177	632610	699244	259541	285703	297270	1.15094	0.0036	0.012281	2.283678745
6-Aminohexanoate	Organic acids	Organic acids	C6H13NO2	60-32-2	1E+07	9879763	9190697	4822070	4694375	4632022	1.16037	0.0026	0.010148	2.068037284
Hypoxanthine	Nucleotides and derivatives	Nucleotides and derivatives	C5H4N4O	68-94-0	197019	244170	188265	91126.7	105566	116741	1.11269	0.01487	0.030029	2.008253239
Zarzissine	Alkaloids	Alkaloids	C5H5N5	160568-14-9	2.6E+07	2.3E+07	2.3E+07	1.9E+07	2E+07	1.8E+07	1.09132	0.01017	0.023022	1.258268732
6-Deoxyfagomine	Alkaloids	Piperidine alkaloids	C6H13NO2	197449-09-5	2104663	1930534	1853045	1007776	946549	987023	1.15687	0.00389	0.012833	2.001885892
Pyridoxine	Others	Vitamin	C8H11NO3	65-23-6	708213	758882	813661	239502	254744	253545	1.1607	0.0029	0.010837	3.049991644
L-Norleucine*	Amino acids and derivatives	Amino acids and derivatives	C6H13NO2	327-57-1	1.9E+07	1.8E+07	1.8E+07	8603682	7503321	8221043	1.15819	2.4E-05	0.001082	2.241691962
Andrographidine E	Flavonoids	Flavones	C24H26O11	113963-41-0	711811	717886	567831	267078	329374	340919	1.12027	0.00867	0.021041	2.130991584
Glucan	Others	Saccharides	C18H32O16	9041-22-9	139170	102446	102511	129116	185783	130372	0.73031	0.21661	0.263061	0.772849116
1-Methyladenine	Nucleotides and derivatives	Nucleotides and derivatives	C6H7N5	5142-22-3	200301	199666	231413	71497.7	87353.7	82100.6	1.14981	0.00213	0.009162	2.620354056
Octadecanamide	Alkaloids	Alkaloids	C18H37NO	124-26-5	1834235	1790512	1764702	2317783	2036001	2077408	1.05013	0.05204	0.07856	0.838017234
5,12-Epoxy-14-Hydroxy-1(6),4,7,11-Cadinapentaene-2,3-dione	Terpenoids	Sesquiterpenoids	C15H12O4	24562-65-0	2847318	2806489	2948889	1944151	2001758	2088273	1.15275	0.00014	0.00213	1.425660841

5,7-Dihydroxy-4-Phenylcoumarin	Lignans and Coumarins	Coumarins	C15H10O4	7758-73-8	747359	661199	870154	454148	520727	532641	1.05797	0.03701	0.059408	1.511567214
Wogonin	Flavonoids	Flavones	C16H12O5	632-85-9	2106217	2176796	2401131	270714	253468	323725	1.16096	0.00129	0.006943	7.883108852
5-hydroxy-4a-methyl-4,4a,5,6,7,8-hexahydronaphthalen-2(3h)-one	Terpenoids	Monoterpenoids	C11H16O2	4242-00-6	168573	165181	180333	121023	118570	135625	1.11122	0.00297	0.010958	1.370102394
Spermine	Alkaloids	Alkaloids	C10H26N4	71-44-3	1.2E+07	6754549	6761847	6200243	5031912	5503111	0.80753	0.23662	0.284126	1.533027229
Pectolinarigenin	Flavonoids	Flavones	C17H14O6	520-12-7	169137	114936	111472	210209	269667	209673	1.01655	0.02311	0.041362	0.573629571
3,5-Dimethylcyclohex-3-ene-1-methyl acetate	Others	Others	C11H18O2	67634-25-7	138589	124103	85124	915880	747959	698974	1.1468	0.00681	0.018097	0.147204139
2-Aminohexadecanoic acid	Lipids	Free fatty acids	C16H33NO2	7769-79-1	123645	130579	147889	159750	168100	176776	1.03145	0.02193	0.039784	0.796852574
Imidazole-4-Acetic Acid*	Alkaloids	Alkaloids	C5H6N2O2	645-65-8	592802	552198	600742	806330	1072657	1345919	1.03874	0.08565	0.120879	0.541331221
Isonicotinic acid	Others	Vitamin	C6H5NO2	55-22-1	2286709	2143003	2274194	1120596	1102313	1309265	1.14604	0.00038	0.003775	1.897954577
His-Tyr	Amino acids and derivatives	Amino acids and derivatives	C15H18N4O4	35979-00-1	106515	98392.2	57361.1	36991.4	31281.7	49236.4	1.00104	0.07361	0.105775	2.231891252
Leu-Arg	Amino acids and derivatives	Amino acids and derivatives	C12H25N5O3	26607-15-8	116373	88834.9	51980.9	37641.1	47950.6	51401.2	0.88046	0.15943	0.203844	1.877387433
N-Acetyl-L-Arginine	Amino acids and derivatives	Amino acids and derivatives	C8H16N4O3	155-84-0	400665	333027	346963	43705.4	85406.5	47615.3	1.13366	0.0006	0.004749	6.114827116
N6-Acetyl-L-lysine	Amino acids and derivatives	Amino acids and derivatives	C8H16N2O3	692-04-6	995249	1054793	1102427	422205	505745	467453	1.15188	0.00017	0.00244	2.259180841
L-Glycyl-L-proline	Amino acids and derivatives	Amino acids and derivatives	C7H12N2O3	704-15-4	1796172	1690945	1794464	677515	792122	752556	1.1569	3E-05	0.00117	2.376743197
Methyl 3-aminopropanoate	Amino acids and derivatives	Amino acids and derivatives	C4H9NO2	4138-35-6	5552667	5631474	4859153	3579301	4129257	4812706	0.92312	0.06089	0.089658	1.281284023
N-Ethylglycine*	Amino acids and derivatives	Amino acids and derivatives	C4H9NO2	627-01-0	6836940	7943576	6977474	5014838	5987704	6253784	0.95228	0.04326	0.067486	1.260870337
2-Amino-2-methylpropanoat	Organic acids	Organic acids	C4H9NO2	62-57-7	7048779	7351415	6188882	4614509	5860921	6206013	0.83716	0.10088	0.138716	1.234250226
N(omega)-Methyl-L-	Amino acids and derivatives	Amino acids and derivatives	C7H16N4O2	17035-90-4	1128262	879133	907589	249783	215942	209003	1.15529	0.00953	0.022147	4.32023234
Vicine	Alkaloids	Alkaloids	C10H16N4O7	152-93-2	2477914	2184160	2209604	1257073	1114833	1569915	1.08853	0.00564	0.016074	1.743274787
4-	Organic acids	Organic acids	C4H9NO2	56-12-2	4801489	4559134	4699694	1430197	1345329	1318313	1.16357	4.1E-05	0.001273	3.434506344
Cystathionine	Amino acids and derivatives	Amino acids and derivatives	C7H14N2O4S	61135-95-3	284298	350001	397415	106454	123578	127937	1.13812	0.01755	0.033663	2.882133437
Retronecine	Alkaloids	Pyrrole alkaloids	C8H13NO2	480-85-3	4404578	3507040	3307425	1190691	926681	1673332	1.10117	0.00577	0.016212	2.959619822
3-(Pyrazol-1-yl)-L-alanine	Amino acids and derivatives	Amino acids and derivatives	C6H9N3O2	2734-48-7	4496698	2595894	2430211	1191915	1606926	1022496	1.03029	0.09477	0.131311	2.492008047
Agmatine	Alkaloids	Alkaloids	C5H14N4	306-60-5	286788	277786	298113	91283.3	110427	122391	1.14633	0.00021	0.002662	2.661781779
LL-2,6-Diaminoheptanedioate	Amino acids and derivatives	Amino acids and derivatives	C7H14N2O4	583-93-7	179062	135778	191394	166195	180113	159407	0.04498	0.993	0.993002	1.001028139
2-Hydroxyl emodin-1-methyl ether	Quinones	Anthraquinone	C16H12O6	346434-45-5	1342120	1183075	1317809	327261	335884	301613	1.16159	0.00187	0.008438	3.983388008
Kolavenic acid	Terpenoids	Ditepenoids	C20H32O2	25436-90-2	921184	680034	398790	978068	658834	383215	0.0088	0.97805	0.981853	0.990045883
14-hydroxy-5-methoxy-8,17-dioxatetracyclo[8.7.0.02,?.011,1?]heptadeca-1(10),2,4,6,11,13,15-heptaen-9-	Lignans and Coumarins	Coumarins	C16H10O5	3923-19-1	194492	114388	158231	33947.4	30406.9	32265.7	1.14183	0.03319	0.054325	4.834522213
3-Hydroxymorindone	Quinones	Anthraquinone	C15H10O6	80368-74-7	8089936	7295359	7092867	104783	154353	370223	1.14181	0.00093	0.005941	35.71590165
4-Hydroxy-L-tryptophan*	Amino acids and derivatives	Amino acids and derivatives	C11H12N2O3	25242-90-4	581219	529090	519969	322224	337467	456376	1.0052	0.03935	0.062551	1.460734491
Thiamine	Others	Vitamin	C12H17N4OS+	70-16-6	1.4E+08	1.2E+08	1.3E+08	5.1E+07	4.8E+07	5.2E+07	1.161	0.00064	0.004881	2.606734839
N-[(R)-2-Hydroxy-3,3-dimethyl-4-(β-D-glucopyranosyloxy)butyryl]-β-alanine	Alkaloids	Alkaloids	C15H27NO10	29493-59-2	324523	244572	310435	1227674	1422581	1281942	1.15499	0.00092	0.005941	0.223673747
Methyl linolenate	Lipids	Free fatty acids	C19H32O2	301-00-8	41364.2	39219.7	46291.4	47397.4	37879.2	36005.8	0.28842	0.67805	0.715216	1.046115015
4(15),10(14)-Aromadendradien-1-ol*	Terpenoids	Sesquiterpenoids	C15H22O	782501-65-9	300432	274425	290509	247337	241813	296427	0.6815	0.26303	0.310072	1.101567383
Creatinine	Alkaloids	Alkaloids	C4H7N3O	60-27-5	254777	256868	205397	298721	337072	269661	0.89415	0.0728	0.104807	0.791914039
Camptothecin*	Alkaloids	Quinoline alkaloids	C20H16N2O4	7689-03-4	1.7E+07	1.7E+07	1.7E+07	2.6E+07	2.7E+07	2.8E+07	1.15901	0.00114	0.006581	0.616746207
Zerumbone	Terpenoids	Ditepenoids	C15H22O	471-05-6	3427529	3407688	3570032	69138.4	87888.9	87902.6	1.1636	0.00019	0.002534	42.48256036
3-Oxo-2-(2-entenyl)cyclopentaneoctanoic acid:OPC-8:0	Lipids	Free fatty acids	C18H30O3	204135-86-4	78681.6	69128.5	66280.4	36234.7	59844.8	51419.9	0.90759	0.06441	0.094163	1.451467232
7-Methylguanine	Nucleotides and derivatives	Nucleotides and derivatives	C6H7N5O	578-76-7	384652	388735	313630	124587	75537.5	123333	1.11922	0.00178	0.008226	3.360612879
Glycidyl Linoleate*	Lipids	Glycerol ester	C21H36O3	24305-63-3	39902	41448.2	41601.6	42429.5	43149.6	46958	0.85864	0.13846	0.181072	0.927678561
Myristamine	Others	Others	C16H35NO	3332-27-2	132901	103486	91976.9	68778	190374	97267	0.00446	0.82786	0.852634	0.921287124
3-acetylursolic acid	Terpenoids	Triterpene	C32H50O4	7372-30-7	1111380	1147872	887450	311255	350848	217133	1.12835	0.0041	0.013197	3.57890424
Methyl	Organic acids	Organic acids	C13H20O3	1211-29-6	169941	156774	191689	247189	231951	174234	0.78662	0.16929	0.215025	0.793425993
Diglycol dibenzoate	Others	Others	C18H18O5	120-55-8	173394	184667	198945	160427	150202	167565	0.96666	0.04967	0.075542	1.164810434
Nordihydrocapsaicin	Alkaloids	Phenolamine	C17H27NO3	28789-35-7	117430	100050	102729	39298.1	34699.2	45109.4	1.14633	0.00138	0.007153	2.688425412
Pseudobaptigeni	Flavonoids	Isoflavones	C16H10O5	90-29-9	6119587	6177693	6581026	2228939	2533518	2630461	1.15657	4.5E-05	0.001296	2.553566682
3,6,6-Trimethyl-2,4-Cycloheptadien-1-one	Terpenoids	Monoterpenoids	C10H14O	2767-18-2	80032.7	80213.3	60176.1	677354	547798	375250	1.14158	0.03369	0.054991	0.137729219
Scutellarein 7,4'-dimethyl ether 6-glucoside*	Flavonoids	Flavones	C23H24O11	57498-69-8	8567514	9584222	9022949	785766	860724	945493	1.16305	0.00101	0.006286	10.48413028

Inosine	Nucleotides and derivatives	Nucleotides and derivatives	C10H12N4O5	58-63-9	271151	217446	215501	398049	343763	418291	1.08728	0.00684	0.018154	0.606927024
Myristamide	Lipids	Free fatty acids	C14H29NO	638-58-4	31138.4	21983.1	15770	22325.4	28485.9	26519.5	0.38701	0.60539	0.648065	0.890865891
Farrerol*	Flavonoids	Flavanones	C17H16O5	24211-30-1	53382.4	53353	41423.5	54112.3	41562.3	42461.9	0.33235	0.58761	0.632309	1.072554703
Chrysin	Flavonoids	Flavones	C15H10O4	480-40-0	117213	147197	194773	296461	329952	296621	1.07527	0.0094	0.021906	0.497471658
Monocrotaline N-oxide	Alkaloids	Alkaloids	C16H23NO7	35337-98-5	263313	204573	196957	131078	99350.4	133870	1.06763	0.02369	0.041959	1.824997352
Soranjidiol; 1,6-Dihydroxy-2-methyl-9,10-anthraquinone	Quinones	Anthraquinone	C15H10O4	518-73-0	1180654	1514764	1410413	2613454	2694518	3191112	1.12631	0.00514	0.015137	0.483091096
5-Hydroxy-2-methyl anthraquinone	Quinones	Anthraquinone	C15H10O3	68963-22-4	416465	512715	400642	716765	765986	900119	1.09938	0.00895	0.021264	0.558075679
Adenosine*	Nucleotides and derivatives	Nucleotides and derivatives	C10H13N5O4	58-61-7	6523005	6913992	6552998	2097012	1879112	2040781	1.16253	6.1E-05	0.001457	3.322305527
4-Fumarylacetoacetate	Organic acids	Organic acids	C8H8O6	28613-33-4	358764	304154	417618	143984	157006	185138	1.11665	0.01679	0.032596	2.222738558
Estrane-3,17-diol	Steroids	Steroid	C18H30O2	517-01-1	6982125	5592497	6385842	2410909	2501526	2718173	1.14996	0.00854	0.020844	2.484790852
Octadeca-9,12,15-trienoic	Lipids	Free fatty acids	C18H30O2	28290-79-1	6626095	8262476	8062700	3093236	3250589	3266545	1.14845	0.01242	0.026321	2.388177636
Dimethyl phthalate	Phenolic acids	Phenolic acids	C10H10O4	131-11-3	1955166	1635766	1928686	1737674	1583565	1916098	0.36525	0.53951	0.586251	1.053897686
4-Hydroxy-L-Isoleucine	Amino acids and derivatives	Amino acids and derivatives	C6H13NO3	1219387-79-7	99103.3	74422.1	96135.6	156178	164245	135175	1.07847	0.00619	0.016979	0.5918842
LysoPC 20:0	Lipids	LPC	C28H58NO7P	108341-80-6	105503	139538	142598	112833	117364	109623	0.61126	0.31087	0.359361	1.140718312
1-Desmethylchryso-obtusin	Quinones	Anthraquinone	C18H16O7	90985-58-3	74944.1	76960.6	77086.7	96108.9	139623	139494	1.03387	0.07775	0.111153	0.610275374
28-Hydroxy-20(29)-Lupene-3,7-dione	Terpenoids	Triterpene	C30H46O3	122537-57-9	181826	155798	166800	74026.6	69236.9	74933.4	1.15653	0.00437	0.013689	2.311781858
Erucamide	Others	Others	C22H43NO	112-84-5	1.5E+07	3E+07	2.9E+07	1.4E+07	2.6E+07	2.8E+07	0.12329	0.79857	0.825487	1.080118604
Palmitoylisopropylamide	Lipids	Free fatty acids	C19H39NO	189939-61-5	5029849	4913425	4687993	8247993	7681015	8606760	1.15112	0.00294	0.010926	0.596323972
Damnacanthal	Quinones	Anthraquinone	C16H10O5	477-84-9	492925	446513	593415	144383	133567	160932	1.15002	0.01174	0.025441	3.492634094
Aphanamol I	Terpenoids	Sesquiterpenoids	C15H24O2	91410-61-6	73218.7	46732.5	35201.5	55930	55985	36382	0.0449	0.87106	0.890024	1.046230578
1-aminobenzocyclobutene	Alkaloids	Alkaloids	C8H9N	61599-85-7	5.4E+07	6E+07	5.1E+07	1.6E+07	1.8E+07	1.7E+07	1.15878	0.00384	0.012753	3.236561587
3-Oxo-1,12-Oleanadien-30-Oic Acid	Terpenoids	Triterpene	C30H44O3	51984-82-8	486806	438193	534717	38511.9	41763.3	32074.1	1.1613	0.00354	0.012144	12.99265972
Astraeusin C	Terpenoids	Triterpene	C30H46O2	1946771-11-4	2016023	2120061	2361741	955286	960698	1076067	1.15033	0.00336	0.011833	2.171695866
Mudanpioside G	Terpenoids	Monoterpenoids	C16H24O8	231280-70-9	647653	565843	581013	726899	778155	597093	0.75862	0.18847	0.234826	0.853654845
DL-Histidyl-DL-histidine	Amino acids and derivatives	Amino acids and derivatives	C12H16N6O3	2733-45-1	1453337	1397746	1406055	693958	581115	738334	1.14403	0.00152	0.007514	2.114394551
11-Hydroxycamptothecin	Alkaloids	Quinoline alkaloids	C20H16N2O5	68426-53-9	3.5E+07	3.2E+07	3.3E+07	2.8E+07	2.8E+07	2.8E+07	1.12085	0.02326	0.041514	1.179404219
Quercetin 3,4'-dimethyl ether*	Flavonoids	Flavonols	C17H14O7	33429-83-3	265920	400675	259161	242473	340450	334884	0.00186	0.96479	0.970113	1.008660129
L-Homoglutamic acid	Amino acids and derivatives	Amino acids and derivatives	C6H11NO4	542-32-5	125347	197268	117826	84017.5	244300	227615	0.23656	0.5477	0.59411	0.792254907
Cuscohygrine	Alkaloids	Pyrrole alkaloids	C13H24N2O	454-14-8	1009040	1016594	954173	963504	933959	930202	0.87752	0.1059	0.1444	1.053804772
Triethyl citrate	Organic acids	Organic acids	C12H20O7	77-93-0	50294.6	53617.3	47333.2	40794.5	39985.4	26437.6	0.90655	0.07253	0.104457	1.410638284
Arcapillin	Flavonoids	Flavones	C18H16O8	83162-82-7	215118	225444	176296	94647.1	123528	160775	0.96579	0.03363	0.054915	1.627809564
Octadeca-11E,13E,15Z-trienoic acid	Lipids	Free fatty acids	C18H30O2	25575-00-2	5.2E+07	4.8E+07	4.7E+07	6E+07	5.6E+07	5.6E+07	1.03355	0.01873	0.035422	0.860029979
γ-Glu-Tyr	Amino acids and derivatives	Amino acids and derivatives	C14H18N2O6	7432-23-7	86021.6	87856.5	101633	32366.1	34867.7	47186.2	1.11992	0.00136	0.007153	2.407895927
Phosphotyrosine	Amino acids and derivatives	Amino acids and derivatives	C9H12NO6P	21820-51-9	150457	131614	135025	67670.4	52047.2	53806.9	1.14066	0.0005	0.004356	2.40367395
5,2',6'-Trihydroxy-6,7-dimethoxyflavone 2'-glucoside:2-[2-(beta-D-Glucopyranosyloxy)-6-hydroxyphenyl]-5-hydroxy-6,7-dimethoxy-4H-1-benzopyran-4-one*	Flavonoids	Flavones	C23H24O12	168293-27-4	3526637	3290488	4135648	9445673	9117643	9683544	1.1527	0.00013	0.00213	0.387751884
Ent-14,15-Dinor-3-Cleroden-3-	Terpenoids	Ditepenoids	C18H30O	130466-21-6	191900	170873	219725	148851	178287	151535	0.84141	0.12173	0.16297	1.216902743
Lys-Phe	Amino acids and derivatives	Amino acids and derivatives	C15H23N3O3	6235-35-4	123184	114517	125077	89301	60622.5	81097.8	1.03592	0.02415	0.042567	1.570325486
Diisobutyl phthalate*	Phenolic acids	Phenolic acids	C16H22O4	84-69-5	4.3E+07	4.2E+07	4.9E+07	4.8E+07	4.4E+07	4.4E+07	0.15268	0.82148	0.846902	0.985947011
2,2-dimethylchromene-6-carboxylic	Others	Others	C12H12O3	34818-56-9	1.9E+07	1.9E+07	1.9E+07	1.9E+07	1.7E+07	1.7E+07	0.90863	0.11465	0.154566	1.069732071
5-Hydroxypseudobaptigenin 7-O-glucoside	Flavonoids	Isoflavones	C22H20O11	52663-80-6	1000744	1013988	1052176	1E+07	1.1E+07	1.2E+07	1.16349	0.00359	0.01227	0.090704142
1-Linoleoylglycero	Lipids	Glycerol ester	C21H38O4	2277-28-3	488551	446099	458831	313289	335400	413383	0.9858	0.05197	0.078536	1.312040599
Succinyladenosine	Nucleotides and derivatives	Nucleotides and derivatives	C14H17N5O8	4542-23-8	1.2E+07	1.2E+07	1.2E+07	4555683	5203216	5350837	1.15615	4.7E-05	0.001313	2.345087142
L-Glycyl-L-phenylalanine*	Amino acids and derivatives	Amino acids and derivatives	C11H14N2O3	721-66-4	2266757	2334198	2414199	1026534	1331258	1338820	1.12221	0.00351	0.012142	1.897725454
4-[3-(4,8-Dimethyl-3,7-Nonadienyl)-3-Methyloxiranyl]-2-Butanone	Terpenoids	Ditepenoids	C18H30O2	115028-53-0	1051315	1034594	1098435	423859	411151	486617	1.15584	4.4E-05	0.001296	2.40941256
p-Cumic alcohol	Terpenoids	Monoterpenoids	C10H14O	536-60-7	142652	154041	171055	1349451	1089080	797628	1.14872	0.02828	0.047748	0.144538096

3-Hydroxy-13-Apo-ε-Caroten-13-one	Terpenoids	Sesquiterpenoids	C18H26O2	761409-24-9	333271	312783	321871	332422	345408	420141	0.73187	0.25082	0.298399	0.881558005
12-Oxo phytodienoic	Lipids	Free fatty acids	C18H28O3	67204-66-4	1108995	1147324	1136966	1369707	1369668	1517618	1.11067	0.02332	0.041562	0.797108501
3-Hydroxy-23-Oxo-11,13(18)-Oleanadien-28-Oic Acid	Terpenoids	Triterpene	C30H44O4	1318005-61-6	128388	128675	122636	31727.7	38463.9	22486.1	1.13735	0.00059	0.004749	4.096992433
N-Cyclohexyl-1,3-benzothiazol-2-amine	Alkaloids	Alkaloids	C13H16N2S	28291-75-0	2055346	2195677	1960552	2227017	1986661	2012661	0.02354	0.96396	0.970088	0.997628931
Oleoylethanolamide	Alkaloids	Alkaloids	C20H39NO2	111-58-0	564260	610422	637460	1616813	1707555	1597064	1.16117	5.5E-05	0.001441	0.368214462
(Oxiran-2-yl)methyl octadeca-9,12-dienoate*	Lipids	Glycerol ester	C21H36O3	856386-19-1	35084.1	32128.6	29738	33831.2	46703.2	37822.1	0.78368	0.19289	0.239025	0.819141918
15-Methoxy-8(17)-Labden-19-Oic Acid	Terpenoids	Ditepenoids	C21H36O3	137960-39-5	189852	174779	180855	215162	200483	195184	0.96745	0.04718	0.072326	0.893025599
Capillarisin	Others	Chromone	C16H12O7	56365-38-9	4010122	3755862	3581185	727341	701410	653741	1.16324	0.00123	0.00683	5.448841316
Obtusifolin	Quinones	Anthraquinone	C16H12O5	477-85-0	332877	224884	303190	83626.5	60227.5	83105.3	1.13277	0.01799	0.034326	3.793413022
Leu-Asp	Amino acids and derivatives	Amino acids and derivatives	C10H18N2O5	32949-40-9	994062	913487	875231	299736	361711	347781	1.15448	0.00059	0.004746	2.757336326
L-Tyrosine*	Amino acids and derivatives	Amino acids and derivatives	C9H11NO3	60-18-4	2.7E+07	2.5E+07	2.6E+07	5696500	5815684	5429907	1.16375	0.00073	0.005254	4.594623803
Synephrine	Alkaloids	Alkaloids	C9H13NO2	94-07-5	703062	524568	713734	561065	588106	453803	0.68875	0.21124	0.257622	1.211101232
Gly-Ala-Phe	Amino acids and derivatives	Amino acids and derivatives	C14H19N3O4	17922-87-1	50220.4	38605.4	34820.8	25026.5	17668.8	16181.1	1.05943	0.02398	0.042372	2.100106537
6-Hydroxy-3-Oxo-11,13(18)-Oleanadien-28-Oic Acid*	Terpenoids	Triterpene	C30H44O4	478083-75-9	396374	345567	319392	81587.2	59541.4	87902.9	1.14788	0.00284	0.010661	4.634004771
Laurocapram	Alkaloids	Alkaloids	C18H35NO	59227-89-3	923913	1020494	923722	1194235	1767344	1217204	0.9412	0.14122	0.184212	0.686355081
Ent-16beta-Methoxy-19-Kauranoic Acid	Terpenoids	Ditepenoids	C21H34O3	38308-35-9	47801.4	249313	159057	59510.2	285427	152649	0.0934	0.88266	0.899498	0.9167674
Isosakuranetin	Flavonoids	Flavanones	C16H14O5	480-43-3	41891.7	39660.3	37660.7	19789	12302.8	21424.7	1.07296	0.00764	0.019613	2.22758644
Panduratin H	Others	Others	C20H26O2	1039021-12-9	37049.1	24945.3	23923.7	45990.7	29767.5	20354.6	0.16045	0.71773	0.752205	0.893929967
4',5,7-Trihydroxy-3',6-dimethoxyflavone (Jaceosidin)	Flavonoids	Flavones	C17H14O7	18085-97-7	313001	276780	246684	700727	676359	595490	1.14225	0.00135	0.007095	0.424046761
Ambroxide	Terpenoids	Ditepenoids	C16H28O	6790-58-5	18569.6	19968.9	13195.8	21203.7	20275.3	26528.4	0.78894	0.12884	0.170791	0.760716717
Tectoquinone	Quinones	Anthraquinone	C15H10O2	84-54-8	437633	474244	567448	78198.1	109643	156894	1.11861	0.00259	0.010123	4.29119113
N-Methyl-2-pyrrolidinone	Others	Others	C5H9NO	872-50-4	994180	878407	806891	1045176	907463	793076	0.1233	0.82121	0.846902	0.975876561
2-Amino-4,5-dihydro-1H-imidazole-4-acetic acid	Alkaloids	Alkaloids	C5H9N3O2	69098-41-5	2.5E+07	2.2E+07	2.6E+07	2.4E+07	2.7E+07	2.5E+07	0.34595	0.57191	0.616914	0.965672505
Canthin-6-one N-oxide	Alkaloids	Plumerane	C14H8N2O2	60755-87-5	11676.3	12996	42388.4	33289.5	44781	60870	0.81925	0.13862	0.181195	0.482658303
Deoxylapachol	Quinones	Quinones	C15H14O2	3568-90-9	524037	490829	464061	27352.4	40554.4	43815.4	1.15709	0.00067	0.005014	13.23753906
IMP	Nucleotides and derivatives	Nucleotides and derivatives	C10H13N4O8P	131-99-7	950483	1064721	900197	379309	409510	465768	1.14566	0.00205	0.008905	2.32379316
LysoPC 15:0*	Lipids	LPC	C23H48NO7P	108273-89-8	108917	109381	132284	84425	129127	129517	0.13351	0.89122	0.907025	1.021899268
Thr-Tyr	Amino acids and derivatives	Amino acids and derivatives	C13H18N2O5	145295-02-9	35066.3	30132.6	33547.1	17050.4	34458.3	12322.4	0.75988	0.22232	0.268835	1.546987106
N-Acetyl-D-glucosamine	Others	Saccharides	C8H15NO6	7512-17-6	410792	389179	387384	294670	326339	319014	1.11291	0.00301	0.011083	1.263111131
4-Pentylbenzene-1,2-diol	Others	Others	C11H16O2	2525-11-3	154651	139950	120137	33002.4	42537.1	38942.3	1.14996	0.0065	0.017534	3.622745669
5-	Flavonoids	Flavones	C16H12O3	42079-78-7	115489	136075	167256	145479	122048	74878.2	0.5218	0.38177	0.431814	1.223170775
Neocnidilide*	Others	Lactones	C12H18O2	4567-33-3	93508	92459	74509.2	59376.7	67139.6	69728	0.98631	0.05434	0.08132	1.327306086
Senkyunolide M	Others	Lactones	C16H22O4	146986-60-9	213969	184377	239499	230918	228807	219229	0.46926	0.48252	0.531031	0.939452177
Stigmasta-4,22-Dien-3-One	Steroids	Steroid	C29H46O	20817-72-5	175760	209247	207924	95854.1	91199.9	100959	1.14624	0.00813	0.020265	2.058691659
Prolylproline	Amino acids and derivatives	Amino acids and derivatives	C10H16N2O3	20488-28-2	63495.5	49205.8	61122.5	30184.8	26446.7	40985.4	1.0351	0.01496	0.03014	1.780672029
N-Methyl-Trans-4-Hydroxy-L-Proline	Amino acids and derivatives	Amino acids and derivatives	C6H11NO3	4252-82-8	89076.9	54057.1	68493.3	46757.5	72982.3	53321.9	0.52687	0.37724	0.427617	1.222843343
Zeanoside B*	Alkaloids	Alkaloids	C16H17NO9	113202-67-8	2637194	2242837	2531957	1383243	1497630	1382273	1.14191	0.00747	0.019314	1.738619539
L-Formylkynurenin	Amino acids and derivatives	Amino acids and derivatives	C11H12N2O4	1022-31-7	206845	152482	156225	129048	154628	135151	0.77964	0.19922	0.245496	1.23094633
2-Methylpyridine	Alkaloids	Pyridine alkaloids	C6H7N	109-06-8	1.4E+07	1.2E+07	1.3E+07	1.2E+07	1.1E+07	1.1E+07	0.79426	0.19339	0.239556	1.10813323
Zeatin-O-glucoside	Alkaloids	Alkaloids	C16H23N5O6	71866-93-8	1.4E+07	1.2E+07	1.3E+07	6.2E+07	5.8E+07	7.1E+07	1.15876	0.00518	0.015188	0.206766945
Guanosine*	Nucleotides and derivatives	Nucleotides and derivatives	C10H13N5O5	118-00-3	6083631	6164666	5949122	6242175	5905577	5973393	0.13019	0.84519	0.868454	1.004209123
2,4-Dihydroxypteridine	Nucleotides and derivatives	Nucleotides and derivatives	C6H4N4O2	487-21-8	497870	519384	542580	810809	812207	748244	1.14778	0.00103	0.006291	0.657808261
16-Hydroxytabersonine*	Alkaloids	Plumerane	C21H24N2O3	22149-28-6	109587	125269	120632	432020	453828	477993	1.16181	0.00053	0.004514	0.260651997
Leu-Leu-Glu	Amino acids and derivatives	Amino acids and derivatives	C17H31N3O6	20274-83-3	1107983	864658	818475	828034	841511	704117	0.68017	0.26084	0.307955	1.175869326
N-Methylhistamine	Alkaloids	Alkaloids	C6H11N3	501-75-7	227512	235430	223896	305548	288712	295267	1.14769	0.00064	0.004881	0.772137899
1-Methylpiperidine-2-carboxylic	Alkaloids	Piperidine alkaloids	C7H13NO2	7730-87-2	2.8E+07	2.4E+07	2.3E+07	2.1E+07	2.8E+07	2.3E+07	0.25064	0.71085	0.745757	1.041510186
Thymonin	Flavonoids	Flavones	C18H16O8	76844-67-2	159381	154683	161191	63246.6	112835	90108.5	1.01697	0.03768	0.060299	1.785399921
Eperuol*	Terpenoids	Ditepenoids	C18H30O	87553-46-6	102557	124085	103433	77040.1	83491.7	93089.8	0.98477	0.04702	0.072143	1.301443658
6beta,7beta-Dihydroxykaurenoic acid	Terpenoids	Ditepenoids	C20H30O4	26109-32-0	2727292	3052868	2587072	964359	1082453	965080	1.15621	0.00355	0.012166	2.778065734
3-Hydroxy-11-oxours-12-en-28-oic acid (11-Keto-ursolic	Terpenoids	Triterpene	C30H46O4	105870-59-5	407472	503580	242743	205457	210999	232492	0.89966	0.15626	0.200458	1.777945931

Tuliposide B	Organic acids	Organic acids	C11H18O9	19870-33-8	4124282	4837328	4346468	2564205	2737752	2833246	1.13595	0.00798	0.020007	1.635862921
Menatetrenone (Vitamin K2)	Others	Vitamin	C31H40O2	11032-49-8	456515	343442	466369	593309	393853	452789	0.4278	0.4676	0.516442	0.87942337
Mudanpinoic acid A	Terpenoids	Triterpene	C30H46O3	203511-36-8	6235978	5837875	5864428	2376348	2386406	2480850	1.16312	0.00069	0.005093	2.476430343
LPC(O-	Lipids	LPC	C26H54NO7P	74389-68-7	1645124	1665881	1775136	914800	877005	955969	1.15745	0.00034	0.003513	1.851004264
L-4-Hydroxyphenylglycine	Amino acids and derivatives	Amino acids and derivatives	C8H9NO3	32462-30-9	291088	183967	235037	455329	319742	310815	0.88729	0.09946	0.137062	0.653929125
L-Ornithine	Amino acids and derivatives	Amino acids and derivatives	C5H12N2O2	70-26-8	39818.8	77055.9	61783	37973.1	104072	41578	0.07084	0.94948	0.957824	0.972959629
6F-alpha-D-Galactosylsucros	Others	Saccharides	C18H32O16	470-57-5	84228	73315.1	45639.5	63458	47657.6	51719.1	0.50826	0.36721	0.417478	1.247783788
N-Hydroxypipecolic acid	Alkaloids	Piperidine alkaloids	C6H11NO3	115819-92-6	91040	60565.2	64191	55166.8	57781.4	63324.7	0.67083	0.30237	0.350842	1.224217084
Maltotriose	Others	Saccharides	C18H32O16	1109-28-0	383623	325069	338984	269975	414988	443124	0.18881	0.67441	0.711862	0.928719155
Lys-Thr	Amino acids and derivatives	Amino acids and derivatives	C10H21N3O4	97791-84-9	106819	83876.4	88954.7	60438.4	80031.3	76949.9	0.86686	0.08937	0.125126	1.286225424
N-Acetyl-L-hydroxyproline	Amino acids and derivatives	Amino acids and derivatives	C7H11NO4	33996-33-7	153027	172136	201296	93156.3	128529	122197	0.9993	0.02926	0.049082	1.530921294
Cinchonain Ib*	Flavonoids	Flavanols	C24H20O9	85081-24-9	1457727	1674704	1799635	1027184	999203	1296943	1.03307	0.01775	0.033953	1.484073179
Indole-3-carboxaldehyde	Alkaloids	Plumerane	C9H7NO	487-89-8	155235	168580	130964	124021	127992	87811	0.84691	0.08708	0.122618	1.338279913
N(6),N(6)-Dimethyl-L-	Amino acids and derivatives	Amino acids and derivatives	C8H18N2O2	2259-86-1	3100546	7986535	8236062	4443378	5225020	5511530	0.26572	0.49728	0.544196	1.272940323
5-Hydroxycoumari	Lignans and Coumarins	Coumarins	C9H6O3	6093-67-0	392027	380961	375811	586904	378779	711301	0.78902	0.21092	0.257361	0.685038475
12,13-Dehydrogeranylgeraniol	Terpenoids	Monoterpenoids	C18H30O	352274-18-1	161763	155974	157356	113281	102032	119547	1.1246	0.00674	0.018015	1.418784662
Cinchonain 1a*	Flavonoids	Flavanols	C24H20O9	85022-69-1	1567937	1880994	1808146	1061887	1434548	1343441	0.9667	0.03361	0.054915	1.369074731
3',4',5',5,7-Pentamethoxyflavone*	Flavonoids	Flavones	C20H20O7	53350-26-8	59685.4	75430.5	44315.3	170125	35944.5	88808.7	0.38299	0.42953	0.480047	0.608492007
Choline	Alkaloids	Alkaloids	C5H15NO4P+	107-73-3	1819714	1700975	1726427	1615298	1341642	1707031	0.73498	0.21208	0.258362	1.125031555
LysoPC 16:0	Lipids	LPC	C24H50NO7P	17364-16-8	620660	767313	649301	435646	440200	483328	1.0954	0.02723	0.046459	1.498905623
3,4-Dihydroxyphenylpropanoate	Phenolic acids	Phenolic acids	C9H10O4	1078-61-1	109838	83834.2	118629	191243	145203	151816	0.99773	0.03422	0.055686	0.639618538
Limocitrin-3-O-glucoside*	Flavonoids	Flavonols	C23H24O13	38836-51-0	6416649	5938242	7310888	6696750	9631202	1.2E+07	0.79813	0.19777	0.244001	0.690733611
Phylloquinone	Others	Vitamin	C31H46O2	84-80-0	97298.1	98579.5	101057	272372	242724	266928	1.16128	0.00282	0.010628	0.379701191
6-Pentadecylsalicylic acid	Phenolic acids	Phenolic acids	C22H36O3	16611-84-0	1790256	1884252	1849097	222737	188230	213696	1.16344	5.2E-05	0.00138	8.84253821
LysoPC 18:0	Lipids	LPC	C26H54NO7P	19420-57-6	1E+07	1E+07	1E+07	6235810	6545651	6665773	1.15973	5.9E-05	0.001457	1.594787129
3,7-Dioxo-12-Oleanen-28-Oic Acid*	Terpenoids	Triterpene	C30H44O4	113738-79-7	388219	348153	391440	74588.2	88589	113011	1.14673	0.00012	0.002129	4.083486416
Tryptamine*	Alkaloids	Plumerane	C10H12N2	61-54-1	2.6E+07	2.4E+07	2.3E+07	8.1E+07	8.4E+07	8.3E+07	1.16213	1.6E-06	0.000394	0.294696717
L-Homomethionine	Amino acids and derivatives	Amino acids and derivatives	C6H13NO2S	25148-30-5	458791	386236	363909	272903	231417	290385	1.05858	0.02148	0.039219	1.521240014
Homoarginine	Amino acids and derivatives	Amino acids and derivatives	C7H16N4O2	156-86-5	415987	503432	499539	111555	137669	144713	1.15012	0.00333	0.011778	3.601988036
PAz-PC	Lipids	LPC	C33H64NO10P	117205-52-4	2687389	2748669	2992024	1719702	1878170	1788079	1.14454	0.00255	0.010061	1.56482712
N-p-Coumaroylspermine	Alkaloids	Phenolamine	C19H32N4O2	130210-35-4	111243	71547.7	67600.8	201479	262569	171047	1.0704	0.02413	0.042555	0.394258829
L-Cyclopentylglycine	Amino acids and derivatives	Amino acids and derivatives	C7H13NO2	2521-84-8	589737	714256	804475	245452	274828	314161	1.1294	0.01408	0.028817	2.52680384
Methionine	Amino acids and derivatives	Amino acids and derivatives	C5H11NO2S	59-51-8	517625	514578	527197	361197	370975	360587	1.16203	7.7E-06	0.00069	1.427029083
Lys-Tyr	Amino acids and derivatives	Amino acids and derivatives	C15H23N3O4	35978-98-4	73408.2	103551	83863.5	39165.3	48569.7	52862.2	1.06709	0.02944	0.049301	1.855104413
L-Homocystine	Amino acids and derivatives	Amino acids and derivatives	C8H16N2O4S2	626-72-2	38511.7	57772.7	66893.3	24163.6	16582.8	25030.3	1.05337	0.04942	0.075194	2.480782685
3-Hydroxy-11-oxours-12-en-23-oic acid (11-Keto-beta-boswellic acid)	Terpenoids	Triterpene	C30H46O4	17019-92-0	433194	347737	414782	72439.7	73407.8	69887.8	1.16104	0.00619	0.016979	5.542494054
Tricin	Flavonoids	Flavones	C17H14O7	520-32-1	64634.5	46598.2	52998.2	56480.2	98877.1	82480	0.79211	0.17489	0.220686	0.690518246
Vidarabine	Nucleotides and derivatives	Nucleotides and derivatives	C10H13N5O4	5536-17-4	7286151	6367420	6208935	1639986	1926473	1933128	1.15738	0.00281	0.010604	3.611636007
Phe-Thr	Amino acids and derivatives	Amino acids and derivatives	C13H18N2O4	51352-44-4	340793	356168	342960	149246	128488	149638	1.15703	4.7E-05	0.001313	2.4332971
Histamine	Alkaloids	Alkaloids	C5H9N3	51-45-6	180474	199150	146892	74087.9	90008.7	61557.8	1.10533	0.00968	0.02239	2.333287261
Stigmast-4-ene-3,6-dione	Steroids	Steroid	C29H46O2	23670-94-2	1054960	1088195	1052661	753388	686509	681468	1.14927	0.00093	0.005941	1.506490853
Triacetonamine	Alkaloids	Piperidine alkaloids	C9H17NO	826-36-8	110253	102945	134126	94049.8	162964	130046	0.28731	0.59184	0.636195	0.897335636
1-Hydroxy-2-methylanthraquinone*	Quinones	Anthraquinone	C15H10O3	6268-09-3	585521	549291	648065	191906	252954	221648	1.1441	0.00101	0.006286	2.674956444
Methyldopa anhydrous	Amino acids and derivatives	Amino acids and derivatives	C10H13NO4	555-30-6	1373732	1510216	1454661	1208295	1516724	1224975	0.61699	0.32671	0.375986	1.098383657
Triethylamine	Alkaloids	Alkaloids	C6H15N	121-44-8	54210.9	41095.9	46816.6	55658.1	53817.1	63359.1	0.84528	0.10395	0.142111	0.822309802
Phe-Ser	Amino acids and derivatives	Amino acids and derivatives	C12H16N2O4	16053-39-7	275977	332019	290842	104359	133136	124729	1.14172	0.00256	0.010087	2.481435432
Dimethylfraxetin; 6,7,8-Trimethoxycoumarin*	Lignans and Coumarins	Coumarins	C12H12O5	6035-49-0	63221.9	60103.4	44220.3	2424022	2619577	2659649	1.16247	0.00078	0.0055	0.02175
9-Oxo-10,12-Octadecadienoic Acid	Lipids	Free fatty acids	C18H30O3	54665-32-6	676420	566239	629356	122706	112872	116713	1.16219	0.00372	0.012518	5.313833512
sn-Glycero-3-phosphocholine	Lipids	PC	C8H20NO6P	28319-77-9	435554	387001	378435	304412	328801	418852	0.64481	0.29418	0.342752	1.141555541
Kaempferol-3-O-(6''-malonyl)glucoside*	Flavonoids	Flavonols	C24H22O14	81149-02-2	47853	52393	68614.6	10988.2	14749.4	36353.6	0.9679	0.02644	0.045564	2.71955426

N-Acetylmethionin	Amino acids and derivatives	Amino acids and derivatives	C7H13NO3S	65-82-7	101544	135028	124760	35468.3	27954	37551.9	1.14322	0.00842	0.02067	3.578459203
MG(18:3(9Z,12Z,15Z)/0:0/0:0)	Lipids	Glycerol ester	C21H36O4	129828-41-7	1286764	1377766	1251904	1008118	1067855	1157829	1.037	0.01738	0.033458	1.211092748
Sweroside	Terpenoids	Monoterpenoids	C16H22O9	14215-86-2	5057295	4483833	4642294	2273198	2062638	2833251	1.11884	0.00171	0.008058	1.978413957
Rhododendrin	Others	Others	C16H24O7	497-78-9	1151930	1135010	1188169	900913	1051500	1129908	0.78347	0.18557	0.232006	1.127432565
1-Hydroxy-3-Methoxy-9,10-Anthraquinone	Quinones	Anthraquinone	C15H10O4	20733-99-7	992202	915450	1093033	620361	706386	790951	1.043	0.0145	0.029463	1.416957057
Dihydrositsirikin	Alkaloids	Plumerane	C21H28N2O3	6519-26-2	30018.8	54047.4	83974.8	210885	227585	215493	1.07765	0.0053	0.015406	0.256957779
Sesaminol 2-O-Beta-D-	Lignans and Coumarins	Lignans	C26H28O12	153512-13-1	2519755	2620482	2706739	1298244	1455380	1679188	1.12471	0.00304	0.011153	1.770202795
Axillarin 7-glucoside*	Flavonoids	Flavones	C23H24O13	41749-40-0	1317867	1142561	1070652	534990	464423	656432	1.11397	0.00316	0.011323	2.132493991
Tyr-Gly*	Amino acids and derivatives	Amino acids and derivatives	C11H14N2O4	673-08-5	433114	454483	537231	284466	382060	367933	0.95266	0.04173	0.065627	1.377365013
Nicotine	Alkaloids	Pyridine alkaloids	C10H14N2	54-11-5	86669	116952	99924.8	126293	116632	82778.2	0.22797	0.66867	0.706292	0.931969032
6-O-methylguanine	Nucleotides and derivatives	Nucleotides and derivatives	C6H7N5O	20535-83-5	397801	261751	365740	47103.4	61542.7	51205.6	1.14963	0.01876	0.035463	6.414014477
cis-Zeatin riboside*	Alkaloids	Alkaloids	C15H21N5O5	15896-46-5	780117	794096	915827	2E+07	1.5E+07	2.3E+07	1.16099	0.01332	0.027649	0.043099846
3-Methoxyanthranilate	Phenolic acids	Phenolic acids	C8H9NO3	3177-80-8	151431	159124	147952	123521	162342	138882	0.52124	0.42673	0.47726	1.079488456
N7-Methylguanosine	Nucleotides and derivatives	Nucleotides and derivatives	C11H15N5O5	20244-86-4	5810694	5860385	5144733	3807514	4543418	5168727	0.87727	0.08895	0.124798	1.243804453
6-amino-9-[(2R,3R,4S,5R)-3,4-dihydroxy-5-(hydroxymethyl)oxolan-2-yl]-1-methylpurin-2-one*	Nucleotides and derivatives	Nucleotides and derivatives	C11H15N5O5	70639-65-5	4339876	4397586	4447149	3150480	3590094	3779363	1.05039	0.03813	0.060923	1.253297406
1,3,5-O-Tricaffeoylquinic acid	Phenolic acids	Phenolic acids	C34H30O15	150035-89-5	2310084	2476850	2506128	4337078	6140306	5396944	1.12446	0.03043	0.050658	0.459424932
Marmesinin	Lignans and Coumarins	Coumarins	C20H24O9	495-30-7	33006.9	119059	58937.2	66103	56367.8	53716.8	0.07353	0.69487	0.729975	1.19760422
Kaempferol-4'-O-glucoside*	Flavonoids	Flavonols	C21H20O11	52222-74-9	758411	888907	817369	537918	242264	425081	0.98644	0.02529	0.044087	2.04493719
Isovanillin	Others	Aldehyde compounds	C8H8O3	621-59-0	391864	313693	382735	706608	738957	563191	1.09626	0.01664	0.032497	0.541773806
Blumenol C glucoside; Byzantionoside	Terpenoids	Terpene	C19H32O7	135820-80-3	72179.5	95775.8	50610.6	47086.1	40092.7	37029.8	0.93928	0.13116	0.173278	1.759668845
GMP	Nucleotides and derivatives	Nucleotides and derivatives	C10H14N5O8P	85-32-5	754937	673185	710008	490974	474369	469354	1.14677	0.00667	0.017882	1.490301511
S-Methyl-L-cysteine	Amino acids and derivatives	Amino acids and derivatives	C4H9NO2S	1187-84-4	6401717	6500908	6220178	1362707	1615519	1581776	1.16129	1.9E-06	0.000394	4.193594556
Vanillylamine	Alkaloids	Alkaloids	C8H11NO2	1196-92-5	46559.8	29972.2	53307.6	13956.7	21448.8	21028.2	1.02322	0.05877	0.086997	2.300746005
alpha-(delta)-Guaiene	Terpenoids	Sesquiterpenoids	C15H22	73003-42-6	775326	619926	674298	95752.8	162492	94353.5	1.13937	0.00169	0.008	5.869428845
Lidocaine	Alkaloids	Alkaloids	C14H22N2O	137-58-6	1.4E+07	1.4E+07	1.5E+07	1.5E+07	1.6E+07	1.6E+07	0.67486	0.2298	0.27651	0.938936484
1-Methyltryptopha	Amino acids and derivatives	Amino acids and derivatives	C12H14N2O2	21339-55-9	44386	44023	56840.6	22574.8	68399.5	84609.1	0.07677	0.64418	0.683449	0.82723995
Pseudotropine	Alkaloids	Tropan alkaloids	C8H15NO	135-97-7	1909556	1963170	1866045	1102225	1130906	1115647	1.16264	0.00055	0.004575	1.713691156
7,9(11)-Fernadiene-3,16,19-Triol	Terpenoids	Triterpene	C30H48O3	237391-99-0	466726	455812	366344	98773.1	58536.9	65140.2	1.1428	0.00345	0.012015	5.79402278
Aflatrem	Alkaloids	Alkaloids	C32H39NO4	70553-75-2	341703	340663	373196	154689	181127	175489	1.15036	0.00027	0.003037	2.064448427
5,4'-Dihydroxy-7,2',5'-trimethoxyisoflavone (Derrugenin)	Flavonoids	Isoflavones	C18H16O7	73428-16-7	53556.7	24378.1	38783.8	60974.6	42536.4	42831	0.52616	0.40094	0.451353	0.797574313
(2E)-3-(1-Hydroxy-2,6,6-trimethyl-4-oxo-2-cyclohexen-1-yl)-2-propenoic	Terpenoids	Sesquiterpenoids	C12H16O4	26690-86-8	1676665	1484709	1573644	2164272	2589579	2387496	1.11385	0.01169	0.025375	0.663042823
4-Pyridoxate	Others	Vitamin	C8H9NO4	82-82-6	54182.2	68144.7	26576	43604.4	20945.4	28921.4	0.6148	0.27347	0.320787	1.593035076
[illegible]hydroxy-7-methyl-1-[(2S,3R,4S,5S,6R)-3,4,5-trihydroxy-6-[[(2R,3R,4S,5S,6R)-3,4,5-trihydroxy-6-(hydroxymethyl)oxan-2-yl]oxymethyl]oxan-2-yl]oxy-1,4a,5,6,7,7a-hexahydrocyclopenta[c]pyran-4-	Terpenoids	Monoterpenoids	C23H36O15	1188391-08-3	793801	813511	992212	222765	336783	317303	1.12433	0.00354	0.012144	2.964613958
Dimethyl[1,1'-biphenyl]-4,4'-dicarboxylate	Others	Others	C16H14O4	792-74-5	135743	143325	131857	26841.1	12939.8	12196.1	1.1332	7.4E-05	0.001618	7.90589435
4-Hydroxy-2-quinolone	Alkaloids	Quinoline alkaloids	C9H7NO2	86-95-3	119036	99255	87185.6	125110	115100	138357	0.8403	0.1075	0.146324	0.806926983
Rehderianin I*	Flavonoids	Flavones	C17H14O7	90965-30-3	328059	355185	296354	253015	341349	309107	0.44948	0.46435	0.513589	1.084260752
3-Dihydrocadambine	Alkaloids	Plumerane	C27H34N2O10	54483-84-0	6872109	6534394	7904127	1281931	1272766	1534464	1.1587	0.00381	0.012714	5.211491505
Ganoderal A	Terpenoids	Triterpene	C30H44O2	104700-98-3	5724031	5867103	5478025	2058780	2200091	2451106	1.15699	2.8E-05	0.001169	2.543847682
Blumenol C	Terpenoids	Monoterpenoids	C13H22O2	36151-02-7	173877	170674	224554	240752	145395	152710	0.22843	0.79258	0.820111	1.056132212
Isorhynchophyllic acid	Alkaloids	Plumerane	C21H26N2O4	144525-05-3	523636	408586	361797	714432	679185	627838	1.03847	0.02062	0.038118	0.640142673
Guvacoline	Alkaloids	Piperidine alkaloids	C7H11NO2	495-19-2	1187161	1043876	968623	622345	1005072	1108577	0.50986	0.4147	0.464649	1.169468938
Asphodelin	Quinones	Anthraquinone	C30H18O8	51419-55-7	76753.3	82233.5	94125.1	28831.4	56465.3	34268.8	1.02644	0.01668	0.032524	2.116931813
1-Oleoyl-Sn-Glycerol	Lipids	Glycerol ester	C21H40O4	129784-87-8	197225	202135	164658	131411	112111	143296	1.03854	0.01857	0.035153	1.458096989

Lauryldiethanolamine	Others	Others	C16H35NO2	1541-67-9	7596473	6358037	8115842	5935248	1.1E+07	6138426	0.01976	0.86561	0.885908	0.957747384
L-Alanyl-L-leucine	Amino acids and derivatives	Amino acids and derivatives	C9H18N2O3	3303-34-2	355313	308925	396531	249191	287148	276745	0.98058	0.06485	0.094771	1.304624348
L-Carnitine	Alkaloids	Alkaloids	C7H15NO3	541-15-1	2404089	3919942	3813396	1223744	5220372	2452552	0.34117	0.77009	0.799665	1.139463261
Deoxyguanosine	Nucleotides and derivatives	Nucleotides and derivatives	C10H13N5O4	961-07-9	403186	447099	369966	49758.3	77342.2	104339	1.12715	0.00049	0.004299	5.272438734
N-Acetyl-D-galactosamine	Others	Saccharides	C8H15NO6	1811-31-0	4315264	3364619	3137383	3289931	3297373	2309094	0.63767	0.26007	0.307518	1.215915287
Arg-Gly	Amino acids and derivatives	Amino acids and derivatives	C8H17N5O3	108347-93-9	384437	390265	384635	363664	309408	351729	0.91621	0.11055	0.149956	1.131279727
Ser-Lys	Amino acids and derivatives	Amino acids and derivatives	C9H19N3O4	22677-61-8	204047	220514	209719	147520	140066	141193	1.15307	0.00127	0.006899	1.479268497
D-Glucosamine	Others	Saccharides	C6H13NO5	3416-24-8	127546	114289	101757	122930	110236	120228	0.23917	0.72286	0.756557	0.972264451
Biochanin A 7-O-(6-O-malonyl-beta-D-glucoside)*	Flavonoids	Isoflavones	C25H24O13	34232-17-2	1.7E+07	1.6E+07	1.6E+07	9764861	9643123	1E+07	1.15966	9.1E-05	0.001798	1.631865348
5-Aminopentanoate	Organic acids	Organic acids	C5H11NO2	660-88-8	34422.3	28349.4	27645.5	26885.4	26679.8	23404.5	0.80731	0.16148	0.206297	1.17471047
Catharanthine	Alkaloids	Plumerane	C21H24N2O2	2468-21-5	135653	155724	121592	228176	145619	231814	0.85565	0.13882	0.181314	0.681907524
Andrographidine C	Flavonoids	Flavones	C23H24O10	113963-39-6	1E+07	1.1E+07	1.1E+07	1.5E+07	1.6E+07	1.8E+07	1.13123	0.00308	0.011188	0.660729322
Glycyl-phenylalanine*	Amino acids and derivatives	Amino acids and derivatives	C11H14N2O3	3321-03-7	2007650	2047140	1717531	957859	1057312	1029059	1.14314	0.00866	0.021041	1.896151502
Benzamide	Alkaloids	Alkaloids	C7H7NO	55-21-0	152282	173207	186902	366722	369009	358961	1.15105	0.00131	0.007019	0.468069
Adenosine 2',3'-cyclic	Nucleotides and derivatives	Nucleotides and derivatives	C10H12N5O6P	634-01-5	6574660	7435025	6046393	2390218	2322231	2718208	1.15119	0.00555	0.015868	2.699099208
Pyrrolidin	Alkaloids	Pyrrole alkaloids	C4H9N	123-75-1	274557	396518	344582	140041	168560	132277	1.1107	0.02407	0.042465	2.303720341
p-Coumaric acid methyl ester	Phenolic acids	Phenolic acids	C10H10O3	19367-38-5	256661	212325	189153	181827	165748	138779	0.91458	0.08265	0.117351	1.353210589
Hercynine	Alkaloids	Alkaloids	C9H15N3O2	534-30-5	261829	243661	254765	134153	129292	126104	1.16093	0.00038	0.003781	1.951628867
3,4-Dihydroxy-L-phenylalanine	Amino acids and derivatives	Amino acids and derivatives	C9H11NO4	59-92-7	350737	346140	383175	88229.2	127097	94599.3	1.14494	0.00011	0.001965	3.484872879
AMP	Nucleotides and derivatives	Nucleotides and derivatives	C10H14N5O7P	61-19-8	1.3E+07	1.3E+07	1.4E+07	4071765	6808924	6637146	1.08023	0.0078	0.019812	2.261362717
Epigoitrin	Others	Others	C5H7NOS	1072-93-1	254209	234716	255343	243206	224084	267367	0.14938	0.83576	0.859619	1.01308192
1,10-Decanediol	Others	Alcohol compounds	C10H22O2	112-47-0	100428	108873	70251.6	356622	421689	282350	1.12525	0.01685	0.032653	0.263563808
L-Azetidine-2-carboxylic acid	Alkaloids	Alkaloids	C4H7NO2	2133-34-8	479664	470005	431843	624102	553323	566725	1.07261	0.01348	0.027925	0.792083781
Gln-Tyr	Amino acids and derivatives	Amino acids and derivatives	C14H19N3O5	457662-02-1	2013529	1887697	1913121	1104190	1329679	1408685	1.09486	0.00972	0.022444	1.513146456
2-(Dimethylamino)guanosine*	Nucleotides and derivatives	Nucleotides and derivatives	C12H17N5O5	2140-67-2	834221	910688	911524	624588	565508	666486	1.11178	0.00253	0.010036	1.430819266
L-Prolyl-L-Leucine	Amino acids and derivatives	Amino acids and derivatives	C11H20N2O3	52899-07-7	1252406	1277820	1036521	447068	400506	443423	1.15257	0.00814	0.020265	2.762785849
Cytosine	Nucleotides and derivatives	Nucleotides and derivatives	C4H5N3O	71-30-7	207146	186721	187330	100167	73568.6	83773.3	1.13492	0.00051	0.004436	2.256997387
4-Methylazetidine-2-Carboxylic acid*	Alkaloids	Alkaloids	C5H9NO2	1779725-63-1	7604589	7994200	7848474	3110465	3277453	3636327	1.1573	4.1E-05	0.001273	2.339055123
14,15-Dehydrocrepenynic acid	Lipids	Free fatty acids	C18H28O2	4154-44-3	3042460	3362853	3150603	1677054	1728641	2164373	1.11125	0.00373	0.012535	1.715583405
Daidzein	Flavonoids	Isoflavones	C15H10O4	486-66-8	517664	529039	438263	132088	134766	141865	1.15948	0.00586	0.01642	3.633220942
Ajmalicine;Tetrahydrosepentine;δ-Yohimbine*	Alkaloids	Plumerane	C21H24N2O3	483-04-5	122289	121543	128394	455889	435268	447583	1.16412	7.5E-05	0.00162	0.278042026
Stachydrine	Alkaloids	Pyrrole alkaloids	C7H13NO2	471-87-4	26237.8	24062	11501	23528.1	21617	14316.6	0.00103	0.89319	0.908742	1.039339418
Morin	Flavonoids	Flavonols	C15H10O7	480-16-0	575262	567291	534703	560377	590202	695658	0.64625	0.30191	0.350701	0.908472398
3'-Methoxydaidzein	Flavonoids	Isoflavones	C16H12O5	21913-98-4	1353165	1382873	1556168	214746	218702	325061	1.14962	0.00037	0.003694	5.658738176
Phellamurin	Flavonoids	Flavanonols	C26H30O11	52589-11-4	515206	530417	566067	980161	995948	1159215	1.14622	0.00885	0.021231	0.514042442
1-Hydroxycanthin-6-one*	Alkaloids	Plumerane	C14H8N2O2	80787-59-3	135352	131767	134662	65976.6	65000.4	67211.1	1.16406	7.5E-06	0.00069	2.027270814
11-Hydroxycanthin-6-one*	Alkaloids	Plumerane	C14H8N2O2	75969-83-4	78788.6	89164.1	123919	39433.8	37469.3	45530.2	1.10011	0.04981	0.075679	2.38392312
1-(1H-indol-5-yl)ethanamine*	Alkaloids	Alkaloids	C10H12N2	147591-52-4	1.3E+07	1.1E+07	1.1E+07	3.4E+07	3.1E+07	3.1E+07	1.15304	7.1E-05	0.001605	0.359013216
Spongouridine	Nucleotides and derivatives	Nucleotides and derivatives	C9H12N2O6	3083-77-0	511612	585846	523124	313651	321486	306386	1.14938	0.00841	0.02067	1.721236638
2,3-Dihydroxy-1-(4-hydroxy-3,5-dimethoxyphenyl)propan-1-one	Others	Ketone compounds	C11H14O6	33900-74-2	37968.7	36195	38616.5	85810.7	73904.4	96319.4	1.14317	0.01692	0.032743	0.440488725
Silydianin	Tannins	Tannin	C25H22O10	29782-68-1	308343	329398	339299	1397063	1477389	1514192	1.16351	0.00046	0.004096	0.222629242
stevenin	Lignans and Coumarins	Coumarins	C16H12O5	36286-69-8	1425187	1581829	1711053	304730	251070	292571	1.15971	0.00308	0.011188	5.561329232
Farrerol-7-O-glucoside	Flavonoids	Flavanones	C23H26O10	885044-12-2	2375014	2256872	2322444	146751	123805	116857	1.16345	0.0001	0.001864	17.95068594
3,6-Dihydroxy-P-Menth-1-En-8-Oic Acid	Terpenoids	Monoterpenoids	C10H16O4	1226854-21-2	227033	194740	236217	138828	125714	140531	1.12045	0.01307	0.027283	1.624372936
Lumichrome	Alkaloids	Alkaloids	C12H10N4O2	1086-80-2	57912.4	41251.9	69025.2	105260	53435.4	56284.5	0.44162	0.46693	0.516072	0.782349386
Glu-Phe	Amino acids and derivatives	Amino acids and derivatives	C14H18N2O5	20556-22-3	640304	627761	660486	218584	234745	228948	1.16322	4.5E-05	0.001296	2.826638286
2'-Deoxyinosine 5'-phosphate	Nucleotides and derivatives	Nucleotides and derivatives	C10H13N4O7P	3393-18-8	1.2E+07	1.1E+07	1.1E+07	1.5E+07	1.5E+07	1.6E+07	1.12837	0.00148	0.007427	0.736157195
1,6-Dihydroxy-P-Menthan-9,3-olide 6-O-β-D-Glucopyranoside	Terpenoids	Monoterpenoids	C16H26O9	179072-42-5	393709	385394	348845	880887	871263	818989	1.15799	7.2E-05	0.001608	0.438695936
Glycyl-leucine	Amino acids and derivatives	Amino acids and derivatives	C8H16N2O3	869-19-2	1265591	1028026	1214821	529427	551355	501821	1.14746	0.01018	0.023022	2.216878185
Gallocatechin gallate	Flavonoids	Flavanols	C22H18O11	4233-96-9	122815	108578	140460	114109	155812	106092	0.01226	0.94295	0.95248	0.988937666
Acetylcorynoline	Alkaloids	Isoquinoline alkaloids	C23H23NO6	18797-80-3	4.5E+07	4.5E+07	4.6E+07	7E+07	7.7E+07	7.6E+07	1.15778	0.00352	0.012142	0.607436331

Isorhamnetin-3-O-Galactoside; Cacticin	Flavonoids	Flavonols	C22H22O12	6743-92-6	501450	557247	593349	164234	128264	157860	1.15347	0.00147	0.007425	3.668300168
2-(3,4-dihydroxyphenyl) chromen-4-one	Flavonoids	Flavones	C15H10O4	4143-64-0	518358	333521	325829	105110	142580	107847	1.11502	0.04455	0.06911	3.312472966
Itaconate	Organic acids	Organic acids	C5H6O4	97-65-4	299197	351202	338392	122169	137721	193916	1.09269	0.00373	0.012535	2.178881953
Khusilal	Terpenoids	Monoterpenoids	C14H18O	2221-18-3	734447	788002	857829	103363	166153	163558	1.14433	0.00039	0.003812	5.496242484
Scopolin	Lignans and Coumarins	Coumarins	C16H18O9	531-44-2	5837773	6140092	5944921	1.6E+07	1.8E+07	1.6E+07	1.16129	0.00295	0.010926	0.360765677
4-Aminoindole	Alkaloids	Plumerane	C8H8N2	5192-23-4	75026.5	31422.6	64508.2	91813.3	130599	129737	0.92942	0.03012	0.050226	0.485468268
Alnusonol	Others	Others	C19H20O4	52330-12-8	126120	120047	118408	63381.6	49114.6	56751	1.14318	0.00062	0.004845	2.154093983
4-Vinylguaiacol	Others	Others	C9H10O2	7786-61-0	447731	349786	364809	101473	104978	155887	1.12581	0.00379	0.012658	3.207852003
Indoline	Alkaloids	Plumerane	C8H9N	496-15-1	5.6E+07	5.7E+07	5.5E+07	1.6E+07	1.9E+07	1.9E+07	1.15939	1.3E-05	0.000842	3.057805911
N5-(1-Iminoethyl)-L-ornithine	Amino acids and derivatives	Amino acids and derivatives	C7H15N3O2	36889-13-1	255813	228463	214821	325773	290561	278151	1.01317	0.02631	0.045413	0.781563103
Methylarbutin	Phenolic acids	Phenolic acids	C13H18O7	6032-32-2	20001.9	33534.6	26151.3	30371.5	23382.3	18414.3	0.26596	0.65699	0.69561	1.104198099
5-Hydroxy-2-pyrrolidinone	Alkaloids	Pyrrole alkaloids	C4H7NO2	62312-55-4	944304	854175	917665	924697	1000840	871522	0.32127	0.59287	0.636863	0.971071515
L-Valyl-L-Leucine	Amino acids and derivatives	Amino acids and derivatives	C11H22N2O3	3989-97-7	477653	483278	500365	108944	150982	159409	1.1441	0.00045	0.004065	3.484795335
Lys-Ala	Amino acids and derivatives	Amino acids and derivatives	C9H19N3O3	17043-71-9	436060	488406	447319	459721	438121	456177	0.18521	0.75655	0.788422	1.013120671
Shanzhiside methyl ester	Terpenoids	Monoterpenoids	C17H26O11	64421-28-9	371955	357827	328750	213059	156749	301961	0.91587	0.08223	0.116801	1.575740654
Hirsutine	Alkaloids	Plumerane	C22H28N2O3	7729-23-9	417443	395364	425831	774652	651492	1369202	1.00193	0.14375	0.186727	0.443107302
1(10),8-Aristoladiene	Terpenoids	Sesquiterpenoids	C15H22	212707-06-7	483388	514542	488284	86213.6	105142	74193.8	1.15645	7E-06	0.00068	5.596756516
Komaroveside B	Terpenoids	Sesquiterpenoids	C19H30O9	1373886-91-9	9777054	8303489	1E+07	5458868	5123513	8007788	0.95305	0.05122	0.077591	1.51542232
(-)-Maackiain-3-O-glucosyl-6"-O-malonate	Flavonoids	Isoflavones	C25H24O13	135574-57-1	308753	463594	355099	161583	154291	182765	1.10839	0.04074	0.064324	2.261046141
(E)-2-O-Cinnamoyl-beta-D-glucopyranose	Phenolic acids	Phenolic acids	C15H18O7	94356-16-8	133951	178473	124148	68846.4	70518	62944.9	1.11401	0.04066	0.064274	2.157944651
Baicalein 5,6,7-trimethyl ether	Flavonoids	Flavones	C18H16O5	973-67-1	839945	977374	915108	99940.5	135802	135278	1.15752	0.00125	0.006845	7.364621308
Ixoroside	Terpenoids	Monoterpenoids	C16H24O9	58514-30-0	276757	285930	207354	204103	154704	174688	0.94967	0.06565	0.095754	1.44338904
2-methyl-1-nitronaphthalene	Others	Others	C11H9NO2	881-03-8	254757	288370	215905	109086	153477	158818	1.04549	0.01488	0.030029	1.801300213
L-Praziquanamine	Alkaloids	Isoquinoline alkaloids	C12H14N2O	99746-73-3	67763.3	100375	77170.2	69197.7	94474.1	83119.1	0.04779	0.96963	0.97403	0.993995052
5'-Methylthioadenosine	Nucleotides and derivatives	Nucleotides and derivatives	C11H15N5O3S	2457-80-9	2.3E+07	2.2E+07	2.4E+07	6852736	6238405	6714594	1.16222	0.00094	0.005992	3.510858472
N6-Threonylcarbamoyladenosine	Nucleotides and derivatives	Nucleotides and derivatives	C15H20N6O8	24719-82-2	7274216	6655816	7393536	2613892	2275912	3144802	1.14198	0.00021	0.002675	2.653965499
Apiosylskimmin (Adicardin)	Lignans and Coumarins	Coumarins	C20H24O12	103529-94-8	107706	103277	84332.1	146112	126080	224111	0.9248	0.14801	0.191373	0.595030093
4-Methoxybenzoat	Phenolic acids	Phenolic acids	C8H8O3	100-09-4	142957	136804	131715	448569	482142	535211	1.16003	0.00456	0.014005	0.280694674
Scillascillin	Flavonoids	Other Flavonoids	C17H12O6	52706-07-7	159943	204190	204619	34276	56554.5	66499.9	1.10625	0.00275	0.010486	3.615013638
L-Leucine*	Amino acids and derivatives	Amino acids and derivatives	C6H13NO2	328-39-2	1.6E+07	1.7E+07	1.6E+07	6177421	7208664	7371631	1.15489	0.00034	0.003531	2.376722946
Avicennol	Lignans and Coumarins	Coumarins	C20H22O5	56110-68-0	1.4E+07	1.4E+07	1.5E+07	1.7E+07	1.5E+07	1.4E+07	0.45317	0.45392	0.503309	0.958223286
Tyr-His	Amino acids and derivatives	Amino acids and derivatives	C15H18N4O4	3788-44-1	71087.4	116471	87718.4	82158.6	64808.8	60900.6	0.72245	0.22823	0.275123	1.324285509
Isohomovanillic acid	Phenolic acids	Phenolic acids	C9H10O4	1131-94-8	104878	172291	247424	163856	268899	237297	0.51488	0.40431	0.454148	0.782914063
Africanone	Terpenoids	Sesquiterpenoids	C15H22O	90851-05-1	509259	385772	471814	285726	370203	458863	0.65099	0.25243	0.299976	1.226098777
6-hydroxycoumarin	Lignans and Coumarins	Coumarins	C9H6O3	6093-68-1	2.6E+07	2.9E+07	2.2E+07	5.3E+07	5.9E+07	5.1E+07	1.13495	0.00069	0.005093	0.471461778
Methyl 3-amino-2-phenylpropanoat	Alkaloids	Alkaloids	C10H13NO2	99092-02-1	942942	814194	832680	277747	230431	187831	1.14578	0.00047	0.004171	3.720953461
L-Valyl-L-Phenylalanine	Amino acids and derivatives	Amino acids and derivatives	C14H20N2O3	3918-92-1	173082	179313	215496	29045.8	31569.9	20088	1.15022	0.00428	0.01357	7.036732263
5,7-Dihydroxy-4-methylcoumarin	Lignans and Coumarins	Coumarins	C10H8O4	2107-76-8	496698	438581	416834	316106	456872	406395	0.60221	0.30781	0.356346	1.146468173
3,4-Dehydro-6-hydroxymellein	Lignans and Coumarins	Coumarins	C10H8O4	1204-37-1	135239	180395	126531	186750	146488	160484	0.47859	0.45276	0.502557	0.89557483
2-Amino-2-methylpentanoic acid	Organic acids	Organic acids	C6H13NO2	3275-37-4	1.5E+07	1.5E+07	1.4E+07	7080191	6972685	7344573	1.1617	0.00064	0.004881	2.030561828
6-Acetamidohexanoic acid	Organic acids	Organic acids	C8H15NO3	57-08-9	1422743	1545012	1588314	2040697	1672958	1715571	0.9012	0.11405	0.15386	0.839174842
6,7-Dihydroxy-4-methylcoumarin	Lignans and Coumarins	Coumarins	C10H8O4	529-84-0	871818	1019472	1189906	743569	738655	912589	0.85883	0.11549	0.155565	1.286612583
5,7-Dimethoxychromone	Others	Others	C11H10O4	59887-91-1	3368588	2916973	3329149	2404871	2558423	4088956	0.28491	0.7647	0.795517	1.062134789
5Z,8Z-tetradecadienoic acid	Lipids	Free fatty acids	C14H24O2	39039-37-7	308504	268279	332704	1903460	1954355	1888238	1.16209	5.3E-07	0.000249	0.158280254
Isocrenatoside	Phenolic acids	Phenolic acids	C29H34O15	221895-09-6	98862.9	58988.2	68893.2	91318.5	128606	115105	0.86218	0.09043	0.126416	0.676788594
Fraxidinglucoside*	Lignans and Coumarins	Coumarins	C17H20O10	58970-71-1	852125	723803	810357	933707	882621	937491	0.9456	0.06502	0.094973	0.866536403
Eleutheroside B1*	Lignans and Coumarins	Coumarins	C17H20O10	16845-16-2	845819	931874	781833	1109676	1416918	1202649	1.05389	0.03312	0.054239	0.686339292
H-Leu-Trp-OH	Amino acids and derivatives	Amino acids and derivatives	C17H23N3O3	5156-22-9	147622	106408	140088	64423.1	63836.3	49725.8	1.10477	0.01915	0.035997	2.214327543
3,4'-Dihydroxy-3',5'-dimethoxypropiophenone	Others	Ketone compounds	C11H14O5	136196-47-9	746624	665934	779650	235405	199488	329818	1.11911	0.00083	0.005655	2.866718995
4'-O-Glucosylvitexin	Flavonoids	Flavones	C27H30O15	38950-94-6	779036	602176	828125	314444	306511	333459	1.13185	0.02457	0.043231	2.314862241
Strictosidinic acid*	Alkaloids	Plumerane	C26H32N2O9	150148-81-5	361209	456137	476182	2983383	2995518	3748222	1.15763	0.00711	0.018664	0.132981543

N-(2-Methylbenzoyl)glycine	Amino acids and derivatives	Amino acids and derivatives	C10H11NO3	42013-20-7	36887.8	18372.3	25459.7	50849.7	43676.2	54369.3	0.9742	0.03179	0.052492	0.542124381
swertiajaposide	Others	Others	C17H24O10	853023-30-0	1.2E+08	1.4E+08	1.4E+08	6.9E+07	9E+07	7E+07	1.10124	0.00326	0.011573	1.765366139
Swertiamarin	Terpenoids	Monoterpenoids	C16H22O10	17388-39-5	2526528	2488094	2120717	1414433	1734930	1635302	1.0772	0.01011	0.022921	1.491293418
Secologanic acid	Terpenoids	Monoterpenoids	C16H22O10	60077-46-5	9356549	9659905	7806009	4698174	6729705	6412569	0.98621	0.02504	0.043872	1.503463551
Glycyl-tryptophan	Amino acids and derivatives	Amino acids and derivatives	C13H15N3O3	2390-74-1	708965	589351	578072	448319	401014	371004	1.08367	0.01841	0.034952	1.537598672
10-Hydroxycamptothecin*	Alkaloids	Quinoline alkaloids	C20H16N2O5	19685-09-7	3349950	5000196	4472505	2552171	3196119	2905550	0.94833	0.08933	0.125126	1.481729736
1,2-Methylenedioxy-3,10,11-trimethoxynoraporphine	Alkaloids	Aporphine alkaloids	C20H21NO5	14050-90-9	114243	203479	162152	190227	322154	229333	0.80969	0.14699	0.190386	0.646979141
Dichomine	Alkaloids	Plumerane	C19H24N2O	89647-74-5	5830148	4930779	7339101	1715429	1984059	2630529	1.10774	0.01973	0.036823	2.859396063
3-Methyl-6-prop-1-en-2-ylcyclohex-3-en-	Terpenoids	Monoterpenoids	C10H16O	20019-62-9	397838	366540	347737	976727	1238288	1071235	1.15232	0.00902	0.021308	0.338414815
8-Methoxyapigenin	Flavonoids	Flavones	C16H12O6	57096-02-3	2130277	2340756	2625323	554539	499707	617283	1.15642	0.00437	0.013689	4.245425841
P-Mentha-1,3,5-Triene-2,5-diol 5-O-[β-D-Apiofuranosyl-(1-6)-β-D-Glucopyranoside	Terpenoids	Monoterpenoids	C21H32O11	1380812-13-4	1041353	1317104	1170255	2217920	2534744	2421526	1.13813	0.00064	0.004881	0.491862029
Glycitein	Flavonoids	Isoflavones	C16H12O5	40957-83-3	1085912	1194971	1233580	790473	919662	914584	1.06962	0.00838	0.02067	1.338987088
Villanovane I	Terpenoids	Ditepenoids	C26H40O9	1583263-49-3	438683	395017	401561	233367	321307	403716	0.75704	0.1953	0.241528	1.288891927
Macrophypene B	Terpenoids	Ditepenoids	C18H28O2	1798799-64-0	3291787	3057489	2954790	1782663	1817445	1748561	1.15654	0.00436	0.013689	1.739510595
Punicic acid	Lipids	Free fatty acids	C18H30O2	544-72-9	2209480	2284040	2307108	367327	345931	380352	1.1641	5.7E-05	0.00145	6.218505662
Isoquinoline	Alkaloids	Isoquinoline alkaloids	C9H7N	119-65-3	1314291	1167537	1182879	279830	523514	376455	1.10992	0.00123	0.006806	3.106210489
3,4,5-trihydroxy-6-(hydroxymethyl)oxan-2-yl]oxypropan-2-yl]cyclohexene-1-carbaldehyde	Terpenoids	Monoterpenoids	C16H26O7	1387639-65-7	569139	632603	620646	1364487	1175441	1361386	1.15095	0.00465	0.014192	0.467121465
Indolin-2-one	Alkaloids	Plumerane	C8H7NO	59-48-3	114115	111985	102936	559602	625893	445194	1.15472	0.01418	0.028974	0.201777772
Mitraphylline	Alkaloids	Plumerane	C21H24N2O4	509-80-8	86202.4	36308.6	39699.8	122292	98706.9	108137	0.94408	0.0578	0.085721	0.492838809
Luteolin-3'-O-glucoside	Flavonoids	Flavones	C21H20O11	5154-41-6	2931020	2337432	2229670	48899.9	78010.2	84111.7	1.15841	0.00784	0.019868	35.53245401
Methyl-3-(3-hydroxyphenyl)Propionate	Phenolic acids	Phenolic acids	C10H12O3	61389-68-2	280130	319880	361729	1722079	1274368	1553309	1.15231	0.00988	0.022568	0.211382629
Eleuthoside B	Terpenoids	Ditepenoids	C26H32O14	180692-77-7	34079.3	62127.9	36249.7	176198	130625	224127	1.09867	0.02891	0.048574	0.249471696
Laricitrin-3-O-glucoside	Flavonoids	Flavonols	C22H22O13	39986-90-8	7102676	6441470	6590330	5005473	5770011	6074916	0.94555	0.05369	0.080513	1.194896016
Luteolin-7-O-glucoside (Cynaroside)*	Flavonoids	Flavones	C21H20O11	5373-11-5	920079	689890	756144	376732	267377	526634	1.00823	0.01752	0.033638	2.021036293
Kaempferol 3-O-glucoside*	Flavonoids	Flavonols	C21H20O11	480-10-4	572920	683071	856104	445557	336512	475144	0.99369	0.0547	0.081679	1.6799813
5-Acetylsalicylic acid	Phenolic acids	Phenolic acids	C9H8O4	13110-96-8	172352	147103	123536	203565	156674	156894	0.59763	0.30551	0.354221	0.856628943
L-Alanyl-L-Phenylalanine	Amino acids and derivatives	Amino acids and derivatives	C12H16N2O3	3061-90-3	114921	156970	125950	66458.4	60919.8	60184.9	1.12641	0.02828	0.047748	2.121105972
5,4'-Dihydroxy-7-methoxyflavanon	Flavonoids	Flavanones	C16H14O5	2957-21-3	13542.6	35759.3	33517.4	24335.8	42778.4	45254.5	0.50614	0.36602	0.416427	0.737031901
Sinapaldehyde-4-O-Glucoside	Others	Aldehyde compounds	C17H22O9	154461-65-1	246578	311156	262553	443050	512439	468136	1.11808	0.00203	0.008886	0.576196207
Gramine	Alkaloids	Plumerane	C11H14N2	87-52-5	292978	297961	275533	162535	143534	158547	1.15336	0.00014	0.00213	1.864919819
Cadambine	Alkaloids	Plumerane	C27H32N2O10	54422-49-0	301306	254407	219579	29074.6	36062.8	38301.6	1.15631	0.01021	0.023028	7.495162725
Catechin 7-arabinofuranosid	Flavonoids	Flavanols	C20H22O10	81905-14-8	3242716	2448911	3328146	2279028	3553877	3104627	0.0575	0.95618	0.963017	1.009201773
Hydroxy Camptothecine*	Alkaloids	Quinoline alkaloids	C20H16N2O5	64439-81-2	3810852	3857746	3879906	580441	662492	546010	1.16245	1.8E-06	0.000394	6.455493692
Biotin	Others	Vitamin	C10H16N2O3S	58-85-5	276279	148781	187111	256423	392732	538447	0.88114	0.12787	0.169728	0.515467787
p-Coumaroyl quinic acid	Phenolic acids	Phenolic acids	C16H18O8	1899-30-5	65445	72268.5	30670.1	64014.9	72427.1	82045	0.57719	0.3261	0.375417	0.770680481
Cyclo(L-tyrosyl-D-proline)	Amino acids and derivatives	Amino acids and derivatives	C14H16N2O3	61117-56-4	12698	7162.23	12854.1	71803.7	60150.5	60797.7	1.13675	0.00121	0.00675	0.169722496
6-Methoxykaempferol-3-O-	Flavonoids	Flavonols	C22H22O12	63422-27-5	392146	383567	383701	82902.7	71878.8	132351	1.12813	0.00343	0.011972	4.037902194
L-Homophenylalanine	Amino acids and derivatives	Amino acids and derivatives	C10H13NO2	943-73-7	29977.1	52499.6	40150.6	60557.6	21292.6	26851	0.3162	0.75991	0.790791	1.12811459
2',4',4-Trihydroxy-3',3-dimethoxychalcone 4'-O-glucoside	Flavonoids	Chalcones	C23H26O11	207906-26-1	276213	267632	291098	51380.9	24936.1	40552.4	1.13944	2.2E-05	0.001051	7.144246991
[6]-Shogaol	Phenolic acids	Phenolic acids	C17H24O3	555-66-8	771611	465392	612786	28088	19584	37692.3	1.15139	0.02155	0.039298	21.66933048
Tricyclene	Terpenoids	Monoterpenoids	C10H16	508-32-7	894932	656907	571235	5807180	5002253	4190864	1.14857	0.00929	0.021724	0.141535489
Asp-Lys	Amino acids and derivatives	Amino acids and derivatives	C10H19N3O5	5891-51-0	475985	1025171	623642	245342	283886	373643	0.98691	0.12461	0.165973	2.353379443
beta-Asarone	Others	Others	C12H16O3	2883-98-9	25030.7	48903.1	43079.6	34286.1	44442.1	40165.5	0.13461	0.94137	0.951195	0.984185277
(+)-Didemethylpinoresinol	Lignans and Coumarins	Lignans	C18H18O6	340167-81-9	779220	719792	769641	1234948	1431745	1248858	1.14308	0.00874	0.021126	0.579395296
Coumarin	Lignans and Coumarins	Coumarins	C9H6O2	91-64-5	15232.8	21397.4	20839.4	9457.98	16988	8443.31	0.86227	0.09333	0.129716	1.647198618
Asn-Ile	Amino acids and derivatives	Amino acids and derivatives	C10H19N3O4	438533-59-6	93834.3	33397.9	76689.5	36480.1	29033.5	32465.9	0.82583	0.18694	0.233167	2.081269056
Neopetasane	Terpenoids	Sesquiterpenoids	C15H22O	151282-84-7	2298114	2532556	2459284	77588.8	63422.3	67647.9	1.16396	0.00083	0.00563	34.93716503
Alanylproline	Amino acids and derivatives	Amino acids and derivatives	C8H14N2O3	13485-59-1	109567	172830	155185	99230.4	82714.6	59971	0.96362	0.05302	0.07973	1.808817177
L-Methionine methyl ester	Amino acids and derivatives	Amino acids and derivatives	C6H13NO2S	10332-17-9	76969.7	76451.7	122407	120566	86298.6	119017	0.5008	0.4312	0.48122	0.84640845

4,5-Dihydroxy-1,2-dimethoxy-7-methylanthracene-9,10-dione	Quinones	Anthraquinone	C17H14O6	89701-79-1	601277	629118	507306	42194	43927	64757.7	1.15622	0.00369	0.01247	11.51720595
Methylmethionine*	Amino acids and derivatives	Amino acids and derivatives	C6H13NO2S	4727-40-6	359177	453533	427030	251740	273094	256745	1.10701	0.02719	0.046415	1.586198932
Asp-Glu	Amino acids and derivatives	Amino acids and derivatives	C9H14N2O7	6157-06-8	49657	27719.2	42228	48128.2	57984.1	45729.1	0.66551	0.23922	0.286521	0.787692069
Phe-His	Amino acids and derivatives	Amino acids and derivatives	C15H18N4O3	16874-81-0	54992.8	29783.9	27163.1	26424.2	18460.6	30080.3	0.6693	0.29865	0.347502	1.493226094
N-Isobutyl Decanamide	Alkaloids	Alkaloids	C14H29NO	73785-31-6	73742.6	55878.2	65831.3	102453	115070	81447.4	0.99204	0.05203	0.07856	0.653750941
[6]-Gingerol	Others	Ketone	C17H26O4	23513-14-6	104288	87955.9	87082.4	9382.19	6255.83	12029.2	1.14868	0.0024	0.009833	10.09593084
3,4-Dehydro-DL-proline	Amino acids and derivatives	Amino acids and derivatives	C5H7NO2	3395-35-5	69615.6	73645.2	60607.4	25640.5	56615	56179.7	0.74274	0.15632	0.200458	1.472661677
3-Dehydrosphinganine	Lipids	Sphingolipids	C18H37NO2	16105-69-4	26081.1	22990.1	23611.7	7031.5	7434.78	7286.55	1.16212	0.00272	0.010433	3.341310425
Andrographidin B*	Flavonoids	Flavones	C23H24O12	113963-38-5	7829187	7821220	8800406	1.7E+07	2E+07	2.1E+07	1.14694	0.00911	0.021507	0.420954118
6-MethylCoumarin	Lignans and Coumarins	Coumarins	C10H8O2	92-48-8	51183.2	74490.4	42701.6	249447	269738	234245	1.13701	0.00016	0.002328	0.223478222
cis-Aconitate	Organic acids	Organic acids	C6H6O6	4023-65-8	79053.6	84282	63150.6	95360.7	104657	116819	0.98865	0.02758	0.046894	0.714835525
Thymidine	Nucleotides and derivatives	Nucleotides and derivatives	C10H14N2O5	50-89-5	121119	120835	120535	32439.6	21982	60203.7	1.05317	0.01851	0.035089	3.162373393
1-Phenylethanol	Phenolic acids	Phenolic acids	C8H10O	98-85-1	60791.6	27258.8	57535	464084	465130	285998	1.12083	0.02381	0.042133	0.119802573
L-Homocitrulline	Amino acids and derivatives	Amino acids and derivatives	C7H15N3O3	1190-49-4	394750	291773	305378	368524	425433	292499	0.34065	0.56556	0.610915	0.912968214
1,2,3,7-Tetramethoxyxanthone	Flavonoids	Other Flavonoids	C17H16O6	22804-52-0	59957.5	49794.2	50359.3	18026.3	12548.8	16420.8	1.14249	0.00223	0.009359	3.406905889
Pentoxifylline	Alkaloids	Alkaloids	C13H18N4O3	6493-05-6	45264.6	58431.5	20036	33721.9	36184	32277	0.17812	0.58955	0.634003	1.210889294
9-Methoxycanthin-6-One	Alkaloids	Plumerane	C15H10N2O2	74991-91-6	9548.42	16721.3	7373.18	48686.3	61478	61692.3	1.11496	0.00165	0.007846	0.195761688
Feruloylputrescin	Alkaloids	Phenolamine	C14H20N2O3	501-13-3	145025	217695	155070	40125.5	35130.4	13972.8	1.08643	0.01591	0.03158	5.802951232
9(12)-Capnellene-8,15-	Terpenoids	Sesquiterpenoids	C15H24O2	1016907-50-8	38465	26011.1	12049.1	19247.7	30612.9	19683.6	0.02288	0.80232	0.828807	1.100382216
Pseudosinin M	Others	Others	C15H24O2	911061-18-2	36841.8	23370.8	33848.3	38520.7	24273.1	8865.16	0.48678	0.49113	0.539167	1.312618442
L-Homocysteine	Amino acids and derivatives	Amino acids and derivatives	C4H9NO2S	6027-13-0	146429	82207	111914	47544.7	27981.8	60824.3	0.99799	0.04732	0.072398	2.4976016
LysoPC 20:3	Lipids	LPC	C28H52NO7P	1199257-41-4	31642.5	38292.5	21112.2	21110.6	20235.9	20717.9	0.82764	0.19285	0.239025	1.466979194
Hispanolone	Terpenoids	Ditepenoids	C20H30O3	18676-07-8	85765.8	42683.2	57478.7	44677.3	50575.5	46151.7	0.57784	0.36098	0.411904	1.314865076
Neohesperidin	Flavonoids	Flavanones	C28H34O15	13241-33-3	31700.3	36862.4	40024.9	22514.8	10008.5	10508.2	1.0226	0.01618	0.031918	2.523440249
Phe-Hyp	Amino acids and derivatives	Amino acids and derivatives	C14H18N2O4	90965-82-5	18080.1	20268	42068.4	18397.2	22532.8	16804	0.49678	0.4289	0.479519	1.392879798
Benzaldehyde	Others	Aldehyde compounds	C7H6O	100-52-7	610383	566358	731049	570456	812544	564391	0.05017	0.89802	0.91185	0.979664649
Antiarol	Others	Others	C9H12O4	642-71-7	47510.5	34647.3	30680.8	61788.9	24854.1	81934.8	0.42757	0.38386	0.433856	0.669356332
Apigenin-6,8-di-C-arabinoside*	Flavonoids	Flavones	C25H26O13	73140-47-3	67346.2	94394.7	93942.2	48661.9	64426.3	65957.4	0.88992	0.08475	0.119886	1.428032123
10-Hydroxycanthin-6-One*	Alkaloids	Plumerane	C14H8N2O2	86293-41-6	139516	175970	111688	51891.1	63190.8	36588.2	1.08286	0.02541	0.044232	2.816468534
Leu-Val-Leu	Amino acids and derivatives	Amino acids and derivatives	C17H33N3O4	58337-01-2	216283	154054	224411	89403.1	63381.9	58289.3	1.10252	0.01679	0.032596	2.817717678
8,11,13,15-Abietatetraen-18-Oic Acid	Terpenoids	Ditepenoids	C20H26O2	82276-84-4	7595.48	21217.9	22010.6	36123.2	35002.5	25895.8	0.83264	0.06096	0.08971	0.523841859
Secoisolariciresinol	Lignans and Coumarins	Lignans	C20H26O6	29388-59-8	114788	151268	96304.9	77077.2	98082	103454	0.72461	0.22148	0.268129	1.300586274
Luteolin-7,3'-di-O-glucoside	Flavonoids	Flavones	C27H30O16	52187-80-1	554883	512248	687063	18268.2	47603.5	30332.9	1.14268	0.00769	0.019684	18.23399842
2,2'-Cyclouridine	Nucleotides and derivatives	Nucleotides and derivatives	C9H10N2O5	3736-77-4	37423.8	6579.3	25508.7	18239.1	24932.8	16579.9	0.06489	0.75661	0.788422	1.163343833
Complanatuside	Flavonoids	Flavonols	C28H32O16	116183-66-5	94983.9	94929.5	91520.2	43184.8	63588.8	75399	0.94614	0.07016	0.101521	1.54487433
Lys-Ser-Glu	Amino acids and derivatives	Amino acids and derivatives	C14H26N4O7	115633-61-9	520619	475812	424695	2465714	2705020	3112524	1.15851	0.00589	0.01647	0.171566111
Isorhamnetin-3-O-	Flavonoids	Flavonols	C28H32O16	55033-90-4	46023.6	90754.8	61254.3	49346.6	53591	50215.3	0.56031	0.37255	0.42277	1.293038625
Dihydronortanshinone	Terpenoids	Ditepenoids	C17H14O4	136832-06-9	64738.2	75641.9	30750.9	65619.1	62737.8	83321.3	0.49345	0.43609	0.485622	0.808448839
Geissoschizine methyl ether	Alkaloids	Plumerane	C22H26N2O3	60314-89-8	9990.23	16552.1	27853.9	36820.1	24069.4	54394.5	0.83905	0.13414	0.176313	0.471845958
Phenylethanolamine	Alkaloids	Alkaloids	C8H11NO	7568-93-6	16641.8	34149.4	23490.6	29133	22044.6	30268	0.30652	0.70414	0.739217	0.912042396
Ser-Leu-Leu	Amino acids and derivatives	Amino acids and derivatives	C15H29N3O5	10329-74-5	165136	187018	167888	184427	242105	236375	0.90823	0.10831	0.147239	0.784488145
Mitraphyllic acid	Alkaloids	Plumerane	C20H22N2O4	10126-00-8	233875	241321	206615	717367	834681	794406	1.15824	0.00196	0.008691	0.290570535
Dehydrodiconiferyl alcohol 4-O-beta-D-glucopyranoside	Lignans and Coumarins	Lignans	C26H32O11	107870-88-2	71226.6	85267.5	83085	25118.8	28072.2	25759.4	1.15629	0.00504	0.014954	3.034553119
3,4-Divanillyltetrahydrofuran	Lignans and Coumarins	Lignans	C20H24O5	34730-78-4	184473	192222	248097	808100	720863	772826	1.15226	9.4E-05	0.001821	0.271437801
Crenatoside	Phenolic acids	Phenolic acids	C29H34O15	61276-16-2	13324.7	33213.7	32689.4	98381.8	117688	123660	1.08108	0.00109	0.006485	0.233208968
Gelselegine	Alkaloids	Plumerane	C21H28N2O4	131653-76-4	93936.6	55354.3	93919.2	12947	54947.9	33261	0.8607	0.05548	0.08267	2.404310443
Isorhamnetin-3-O-rutinoside; Narcissoside; Narcissin*	Flavonoids	Flavonols	C28H32O16	604-80-8	40144.9	28648.2	48611.1	48013.1	60425.3	92213.9	0.84838	0.15763	0.201798	0.585112566
Vindolinine*	Alkaloids	Plumerane	C21H24N2O2	5980-02-9	116129	613354	247578	104267	88108	58588.7	0.87411	0.24461	0.292133	3.893239794
Phellodendroside	Flavonoids	Flavanonols	C26H30O11	40451-69-2	295301	335404	386198	630919	563014	665889	1.11548	0.00233	0.009637	0.546774655
Salireposide	Phenolic acids	Phenolic acids	C20H22O9	16955-55-8	22678.9	31780.6	26063.5	4535.79	4535.79	4535.79	1.1575	0.01388	0.028505	5.91761067
Tomentin	Flavonoids	Flavonols	C17H14O8	59171-23-2	40030.6	30075.3	23169.8	30597.6	41573.9	16286.1	0.18045	0.86538	0.885908	1.054468659
3,4-Di-O-caffeoylquinic acid methyl ester	Phenolic acids	Phenolic acids	C26H26O12	114637-83-1	225946	195284	239941	23091.7	23091.7	23091.7	1.16299	0.00444	0.013818	9.544123215
Peucedanol 7-O-glucoside	Lignans and Coumarins	Coumarins	C20H26O10	65853-04-5	214036	136011	152298	107057	151720	98407.4	0.77724	0.17846	0.224284	1.406405124
2-Phenylethy-1-O-β-D-glucoside	Others	Alcohol compounds	C14H20O6	18997-54-1	21694.7	21694.7	21694.7	130567	151142	156510	1.16283	0.00402	0.013056	0.148519317

5-Hydroxyconiferaldehyde	Others	Others	C10H10O4	249647-14-1	53494.3	88834.2	38353.9	316378	334018	339970	1.12172	0.00066	0.004944	0.182440153
2,5-Dimethoxybenzoquinone	Quinones	Quinones	C8H8O4	3117-03-1	5590498	4960499	4806293	1756923	1745013	2105417	1.15191	0.00137	0.007153	2.738777224
Hyperin*	Flavonoids	Flavonols	C21H20O12	482-36-0	116443	172893	96245.1	77581.5	24398.2	72847.8	0.85083	0.07497	0.10758	2.205494191
Myricetin-3-O-glucuronide	Flavonoids	Flavonols	C21H18O14	77363-65-6	40875.9	77754.9	45672.9	9151.55	17692.1	17801.9	1.05986	0.0673	0.097845	3.68017995
Caffeine	Alkaloids	Alkaloids	C8H10N4O2	58-08-2	133202	271057	77739.4	90161.5	67406	45167.5	0.79752	0.24351	0.291159	2.37748165
Bakuchicin	Lignans and Coumarins	Coumarins	C11H6O3	4412-93-5	510040	566562	468874	55336	36080.3	35465.4	1.15618	0.0024	0.009833	12.18044738
3-oxoglycyrrhetinic acid*	Terpenoids	Triterpene	C30H44O4	7020-50-0	151559	89548.6	116644	11003.5	11003.5	11003.5	1.15548	0.02641	0.045528	10.83750931
Met-Phe	Amino acids and derivatives	Amino acids and derivatives	C14H20N2O3S	14492-14-9	28777.4	25398.9	34998.3	13255.6	27929.8	15228.3	0.82995	0.12718	0.168885	1.580724656
3-Ethynylbenzaldehyde	Others	Aldehyde compounds	C9H6O	77123-56-9	2622683	2512050	3178176	250685	267548	338747	1.15853	0.00611	0.016845	9.700240889
3',4',7-Trihydroxyflavon	Flavonoids	Flavones	C15H10O5	2150-11-0	91470.4	66858	91648	87674.3	83156.6	70223.5	0.14017	0.77824	0.806345	1.037012179
S-Methyl GSH	Amino acids and derivatives	Amino acids and derivatives	C11H19N3O6S	2922-56-7	176899	167631	193399	96957.3	68962.9	60990.5	1.10655	0.00231	0.009608	2.370661181
5-Oxo-D-proline	Amino acids and derivatives	Amino acids and derivatives	C5H7NO3	4042-36-8	3.7E+07	3.3E+07	3.5E+07	1200604	1131822	1633951	1.16174	0.0015	0.007449	26.49604063
Caffeic aldehyde	Others	Aldehyde compounds	C9H8O3	141632-15-7	7756.58	7756.58	7756.58	260533	126395	87629.1	1.13742	0.10288	0.140907	0.04903462
L-Pipecolate	Alkaloids	Piperidine alkaloids	C6H11NO2	1723-00-8	2.3E+07	2.2E+07	2.6E+07	689365	611239	741941	1.16367	0.00278	0.010534	35.19623442
L-Arginine	Amino acids and derivatives	Amino acids and derivatives	C6H14N4O2	74-79-3	7611660	7261443	7512409	4288464	4965654	4821064	1.14163	0.00132	0.007025	1.590424318
Asn-Leu	Amino acids and derivatives	Amino acids and derivatives	C10H19N3O4	22601-71-4	28199.4	32749.7	49465.7	15793.8	14470.1	16485.5	1.07475	0.08041	0.114378	2.361843038
S-methyl-L-thiocitrulline	Amino acids and derivatives	Amino acids and derivatives	C7H15N3O2S	209589-59-3	125279	105533	166916	22875.1	4575.01	52759.7	0.94095	0.01136	0.024829	4.958603769
Biochanin A	Flavonoids	Isoflavones	C16H12O5	491-80-5	163414	198806	207115	21973.9	16312.4	12514.6	1.15232	0.00453	0.01396	11.20718577
Dopamine	Alkaloids	Alkaloids	C8H11NO2	51-61-6	31414.7	32894.6	32035.2	3244.09	3244.09	3244.09	1.16466	0.00022	0.002725	9.8995062
O-	Alkaloids	Alkaloids	C9H17NO4	3040-38-8	7180318	5918434	6066486	2721004	4215633	3965249	1.03433	0.01125	0.024605	1.757974568
Sorbifolin	Flavonoids	Flavones	C16H12O6	23130-22-5	1014473	1270640	1065796	118239	118239	118239	1.16255	0.00608	0.01681	9.44672785
4-hydroxy-2-oxo-1,2-dihydroquinoline-3-carboxylic	Alkaloids	Quinoline alkaloids	C10H7NO4	73776-24-6	2738140	2825724	2846281	386166	394505	376232	1.1645	0.00013	0.00213	7.269539178
Alismol	Terpenoids	Sesquiterpenoids	C15H24O	87827-55-2	56810.4	55438	90811.4	7750.25	7750.25	7750.25	1.15138	0.0353	0.057043	8.733470125
Neoabietic acid*	Terpenoids	Ditepenoids	C20H30O2	471-77-2	28797.9	27879.7	17238.3	34730.3	24056.5	14811.2	0.07495	0.98851	0.98979	1.00431941
alpha-Curcumene*	Terpenoids	Sesquiterpenoids	C15H22	4176-17-4	52845.8	49605.2	59798.7	6034.96	6034.96	6034.96	1.16317	0.00389	0.012833	8.961654524
Cyclo(Ala-Gly)	Amino acids and derivatives	Amino acids and derivatives	C5H8N2O2	4526-77-6	674838	614869	660607	767140	853363	632600	0.69799	0.25297	0.300496	0.865611625
N6-methyladenosine	Nucleotides and derivatives	Nucleotides and derivatives	C11H15N5O4	1867-73-8	72058.9	87734.7	93448.5	55336.4	47420.9	62501.8	1.0311	0.02418	0.042593	1.53239456
Costunolide	Terpenoids	Sesquiterpenoids	C15H20O2	553-21-9	51198.7	42139.2	30610.6	28220.8	21552.5	23469.6	0.97221	0.09294	0.12929	1.692294424
5'-Deoxyadenosine*	Nucleotides and derivatives	Nucleotides and derivatives	C10H13N5O3	4754-39-6	60393.1	72427.4	59486.3	30147.4	22359.5	40175.1	1.05108	0.00833	0.020626	2.074909997
6,7-Dehydroartemisinic acid	Terpenoids	Sesquiterpenoids	C15H20O2	120193-24-0	21133.2	16338.9	29584.7	3267.78	3267.78	3267.78	1.14563	0.03878	0.061744	6.840198749
Secologanoside dimethyl ester	Terpenoids	Monoterpenoids	C18H26O11	74713-15-8	1831100	1657797	1735542	180386	180386	180386	1.16444	0.00103	0.006291	9.654180295
Homophthalicaci	Phenolic acids	Phenolic acids	C9H8O4	89-51-0	162808	129568	198580	448669	366152	144818	0.71931	0.22354	0.2701	0.511605469
Dhurrin	Alkaloids	Phenolamine	C14H17NO7	499-20-7	31622	43828	59992.4	10189.1	19328.2	30183.6	0.89431	0.07292	0.104933	2.2686813
Homoplantaginin	Flavonoids	Flavones	C22H22O11	17680-84-1	425116	479130	366919	154349	157842	259356	1.06191	0.00793	0.019937	2.224079188
Corynoxine B	Alkaloids	Plumerane	C22H28N2O4	17391-18-3	45198.3	64080.2	52516.3	49735.9	41650.2	26896.2	0.72783	0.17177	0.217464	1.367870584
Cordycepin*	Nucleotides and derivatives	Nucleotides and derivatives	C10H13N5O3	73-03-0	84036.8	62102.9	106741	7365.58	7365.58	7365.58	1.15485	0.02693	0.046098	11.44425638
Matairesinoside	Lignans and Coumarins	Lignans	C26H32O11	23202-85-9	115629	122955	102648	188117	62221.2	161138	0.13915	0.60441	0.647467	0.829288166
Phe-Ile	Amino acids and derivatives	Amino acids and derivatives	C15H22N2O3	22951-94-6	97758.4	109008	149880	94190.3	54172.6	58276.9	0.92217	0.07246	0.104405	1.725934383
Gentiascabraside A	Terpenoids	Monoterpenoids	C17H24O11	857665-56-6	546942	564898	502943	1495148	1218851	1528872	1.14854	0.01031	0.023202	0.380587336
P-Menth-4-Ene-1,2-diol 1-O-β-D-	Terpenoids	Monoterpenoids	C16H28O7	827038-23-3	19277.1	19277.1	19277.1	96385.7	168259	121483	1.14807	0.0351	0.056751	0.149772782
Tyramine	Alkaloids	Alkaloids	C8H11NO	51-67-2	9464.41	3265.15	8034.3	32604.5	34642.1	41966.9	1.08573	0.00183	0.008411	0.190121746
Biochanin A-beta-D-	Flavonoids	Isoflavones	C22H22O10	5928-26-7	2847455	2890369	2974752	1538692	1697633	1810957	1.14626	0.00102	0.00629	1.726191411
Quinine	Alkaloids	Quinoline alkaloids	C20H24O2N2	130-95-0	19243.7	29548.6	11567.1	52820.5	65443.8	48348.5	1.02863	0.00835	0.020626	0.36227347
Apigenin-6-C-arabinoside-8-C-xyloside*	Flavonoids	Flavones	C25H26O13	677021-30-6	113692	96894.7	65522.2	77593	65951.7	83521.8	0.51682	0.36954	0.419665	1.215981268
(-)-Epicatechin	Flavonoids	Flavanols	C15H14O6	490-46-0	20963.3	31809	45401.5	3445.02	3445.02	3445.02	1.14075	0.05365	0.080487	9.499099879
Apigenin 7-O-beta-D-	Flavonoids	Flavones	C21H20O10	578-74-5	2423698	2310689	2692177	790295	935849	905892	1.15433	0.00189	0.008519	2.821604422
4',6-Dihydroxy-5,7-dimethoxyflavon	Flavonoids	Flavones	C17H14O6	33028-99-8	119126	125621	123245	106424	106432	119872	0.89419	0.10427	0.142491	1.105987099
Carveol*	Terpenoids	Monoterpenoids	C10H16O	99-48-9	457510	488962	456429	676148	771250	747150	1.1414	0.00591	0.016502	0.639266227
6'-O-Sinapoylgeniposide	Terpenoids	Monoterpenoids	C28H34O14	1012306-66-9	185502	133815	153745	146682	123033	155834	0.463	0.43493	0.484677	1.111650067
Acacetin 7-apiosylglucoside	Flavonoids	Flavones	C27H30O14	239106-94-6	259294	162953	155109	77342.3	115412	113434	0.96752	0.10067	0.138611	1.885619745
2,6-Dimethyl-7-Octene-2,3,6-Triol 2-O-β-D-Glucopyranoside	Terpenoids	Monoterpenoids	C16H30O8	288152-88-5	36089.5	40886.6	34868.6	31719.8	70626.7	89791.9	0.67737	0.25688	0.304442	0.582104559
Quercetin-3-O-glucuronide	Flavonoids	Flavonols	C21H18O13	22688-79-5	34469.5	16645.9	45804.8	15206.7	3467.5	7737.39	0.93088	0.09412	0.13058	3.669613078
3,4-Dimethoxypheno	Others	Others	C8H10O3	2033-89-8	741997	898951	873409	1082905	1096624	739926	0.50703	0.37223	0.422563	0.861241632

Pelargonidin 3-O-(6-O-malonyl-beta-D-	Flavonoids	Anthocyanidins	C24H23O13+	165070-68-8	34479.1	15837	14596.1	129938	136250	184141	1.11643	0.00982	0.022547	0.14414396
L-Phenylalanyl-L-phenylalanine	Amino acids and derivatives	Amino acids and derivatives	C18H20N2O3	2577-40-4	66003.3	50207.7	29523.9	22653.7	19107.7	45340.1	0.70465	0.22208	0.268647	1.673162055
Kaempferol-3-O-arabinoside (Juglanin)	Flavonoids	Flavonols	C20H18O10	5041-67-8	18768.4	40456.6	31269.2	19275	12490.8	28056.6	0.63048	0.26337	0.310354	1.5127141
Sophoricoside*	Flavonoids	Isoflavones	C21H20O10	152-95-4	3359769	3473864	3567471	391860	364108	476484	1.16136	1.6E-05	0.000908	8.439361472
Dihydrokaempferide	Flavonoids	Flavanonols	C16H14O6	137225-59-3	239192	237273	455280	13835.6	3772.5	13163.4	1.12408	0.05316	0.079908	30.2794844
Phenylacetylglycine	Amino acids and derivatives	Amino acids and derivatives	C10H11NO3	500-98-1	253287	40460.2	234093	272158	270006	175857	0.48296	0.46347	0.512793	0.735132259
2-(Hydroxymethyl) Cyclohexa-2,5-Diene-1,4-Dione	Others	Others	C7H6O3	644-17-7	853021	814029	722935	77153.5	77153.5	77153.5	1.16388	0.00286	0.01073	10.32567149
2,9-Bornanediol 2-O-[β-D-Apiofuranosyl-(1-6)-β-D-Glucopyranoside	Terpenoids	Monoterpenoids	C21H36O11	217969-26-1	642639	642639	642639	4847636	3968040	3977116	1.16181	0.00642	0.017353	0.150703324
Quercetagetin	Flavonoids	Flavonols	C15H10O8	90-18-6	171471	186874	128469	135301	112195	115937	0.86865	0.12928	0.17131	1.33948772
Gentioflavoside*	Terpenoids	Monoterpenoids	C16H22O10	34239-17-3	379397	439565	369664	41659.9	41659.9	41659.9	1.16344	0.00378	0.012658	9.51056123
Diosmetin-7-O-glucoside*	Flavonoids	Flavones	C22H22O11	20126-59-4	652011	588471	406361	146452	90610.7	118531	1.12801	0.02371	0.041966	4.631257325
9-Oxo-12Z-Octadecenoic	Lipids	Free fatty acids	C18H32O3	112543-32-5	29290.8	11688.8	16447.2	2337.77	2337.77	2337.77	1.12674	0.0855	0.120724	8.188274091
Protochiisanogenin	Terpenoids	Triterpene	C30H46O5	193206-51-8	147296	117171	125219	9497.8	9497.8	9497.8	1.16332	0.00555	0.015868	13.67636637
Olibanumol C	Terpenoids	Monoterpenoids	C10H18O2	366802-85-9	13684.5	13684.5	13684.5	159507	107176	114013	1.15741	0.0204	0.037779	0.107837688
Wogonin-7-O-glucuronide*	Flavonoids	Flavones	C22H22O10	51059-44-0	1.3E+07	1.3E+07	1.5E+07	501704	579519	540443	1.1639	0.00177	0.008205	24.96485129
Oroxin A	Flavonoids	Flavones	C21H20O10	57396-78-8	1666786	1582175	1818454	221382	221382	221382	1.16373	0.00221	0.009303	7.629982883
4,6-Dihydroxyquinoline	Alkaloids	Quinoline alkaloids	C9H7NO2	3517-61-1	101983	52355.5	52626.7	7020.86	7020.86	7020.86	1.14317	0.06413	0.093809	9.826219823
Vitexin -4"-O-glucoside	Flavonoids	Flavones	C27H30O15	178468-00-3	1272486	965554	1331307	305108	80887.3	74704.7	1.0769	0.00274	0.010461	7.747662097
Saponarin	Flavonoids	Flavones	C27H30O15	20310-89-8	1952875	2137573	2087854	57709.3	800989	11541.9	0.92439	0.01675	0.032567	7.099539996
5-Formylbenzofura	Others	Others	C9H6O2	10035-16-2	153484	153484	153484	1327105	1232403	1326137	1.16441	0.00076	0.005359	0.118501006
5-O-Methylvisammioside	Others	Chromone	C22H28O10	84272-85-5	181224	218763	256269	21352.7	21352.7	21352.7	1.1601	0.01183	0.025541	10.24469403
Glycitin*	Flavonoids	Isoflavones	C22H22O10	40246-10-4	11035.5	11035.5	11035.5	67979.6	58146.4	55177.4	1.16154	0.00608	0.01681	0.182602403
6-Hydroxynicotinat	Alkaloids	Pyridine alkaloids	C6H5NO3	5006-66-6	18676.2	21192.4	47344.4	34935.5	37681	22243	0.25622	0.82094	0.846902	0.919391437
Niloticin*	Terpenoids	Triterpene	C30H48O3	115404-57-4	237273	211137	178340	20061.3	20061.3	20061.3	1.16211	0.00805	0.020134	10.4139063
1-Methylnicotinamide	Alkaloids	Pyridine alkaloids	C7H9N2O	1005-24-9	5392.09	5392.09	5392.09	40706.2	56665.7	37316.6	1.15652	0.02205	0.039953	0.120101469
4-Nitrophenol	Others	Others	C6H5NO3	100-02-7	2279668	1905127	1958601	2601192	2732583	2444776	1.02348	0.02306	0.04129	0.7897866
Afformosin	Flavonoids	Isoflavones	C17H14O5	550-79-8	988988	896819	914960	89521.6	89521.6	89521.6	1.16446	0.00111	0.006549	10.42864735
Mosloflavone; 5-Hydroxy-6,7-Dimethoxyflavon	Flavonoids	Flavones	C17H14O5	740-33-0	15990.5	15990.5	15990.5	182513	153350	140553	1.16211	0.00747	0.019314	0.100692718
Tyr-Ala	Amino acids and derivatives	Amino acids and derivatives	C12H16N2O4	730-08-5	25821.6	39072.9	18868	6218.5	9456.67	10804.2	1.05284	0.07756	0.110933	3.163315868
Naringenin-4',7-dimethyl ether	Flavonoids	Flavanones	C17H16O5	29424-96-2	2420.92	2420.92	2420.92	12104.6	23547.6	19134.8	1.14282	0.04151	0.065379	0.132563339
Isooccidol 2	Terpenoids	Sesquiterpenoids	C15H22O	87797-89-5	8472.61	8472.61	8472.61	42363.1	42805.3	48752.5	1.16301	0.00323	0.011497	0.189797315
1,11-Dihydroxy-3-Eudesmen-12,6-olide 1-O-β-D-	Terpenoids	Sesquiterpenoids	C21H32O9	1438883-37-4	251731	234072	402943	117619	101151	109636	1.09386	0.07226	0.104219	2.706246476
2-(4-hydroxy-2-methoxyphenyl)-6-methoxy-1-benzofuran-3-carbaldehyde	Lignans and Coumarins	Lignans	C17H14O5	877932-11-1	37835.9	37835.9	37835.9	399132	396709	433776	1.1644	0.00103	0.006293	0.092311424
Rhamnocitrin	Flavonoids	Flavonols	C16H12O6	569-92-6	59108.1	61165.5	54075.6	157208	166248	134862	1.15172	0.00722	0.018854	0.380411144
2-Ethyl-2,6,6-trimethylpiperidin-4-one	Alkaloids	Piperidine alkaloids	C10H19NO	133568-79-3	45079.1	51084.9	33320.2	28453.1	30741.3	24626.3	0.96724	0.08751	0.123112	1.544774213
N-p-Coumaroyltyrosine	Amino acids and derivatives	Amino acids and derivatives	C18H17NO5	77201-66-2	3500.5	3500.5	3500.5	17502.5	32150.4	35926.9	1.13825	0.04689	0.072081	0.122710074
4-Hydroxyhippurat	Amino acids and derivatives	Amino acids and derivatives	C9H9NO4	2482-25-9	25592.7	25592.7	25592.7	192478	181849	213206	1.16349	0.00291	0.010855	0.130679147
7,8-Dihydroxy-4-phenylcoumarin	Lignans and Coumarins	Coumarins	C15H10O4	842-01-3	583180	606455	601302	77312.5	77312.5	77312.5	1.16465	0.00018	0.002534	7.721644889
5,6-Dihydroxy-7,4'-dimethoxyflavone (Ladanein)	Flavonoids	Flavones	C17H14O6	10176-71-3	111960	71792.8	70553.9	4513.34	4513.34	4513.34	1.15888	0.02754	0.046858	18.78185015
4-Acetamidobutanoate	Organic acids	Organic acids	C6H11NO3	3025-96-5	3582963	3139789	3230444	334365	334365	334365	1.16415	0.00205	0.008905	9.922486188
Scopolamine	Alkaloids	Tropan alkaloids	C17H21NO4	51-34-3	14587.1	14811.3	18428.9	13613.9	27956.9	15369.6	0.30941	0.57549	0.620345	0.839952654
2-Oxo-3,4-dihydro-1H-quinoline-3-carboxylic acid	Alkaloids	Quinoline alkaloids	C10H9NO3	246867-17-4	36910.2	36910.2	36910.2	324225	393244	353356	1.16343	0.00388	0.012833	0.103406929
Epigallocatechin-(4β→8)-epigallocatechin-3-O-gallate	Flavonoids	Flavanols	C37H30O18	86588-88-7	7999	14927.8	18840	16947.9	24196.5	35682.8	0.79729	0.15489	0.199192	0.543646079
Deoxyadenosine*	Nucleotides and derivatives	Nucleotides and derivatives	C10H13N5O3	958-09-8	141221	149250	148432	12032.7	12032.7	12032.7	1.1646	0.00036	0.003654	12.1586304
H-Pro-Ile-OH	Amino acids and derivatives	Amino acids and derivatives	C11H20N2O3	51926-51-3	181919	227655	163888	20252.8	20252.8	20252.8	1.1605	0.01211	0.025852	9.438372862

5,8,11,13-Abietatetraen-18-Oic Acid	Terpenoids	Ditepenoids	C20H26O2	344363-33-3	8543.17	15082.1	21725.4	23391.2	16624.9	12642.2	0.32635	0.64816	0.68697	0.861225808
Isorhamnetin-7-O-glucoside (Brassicin)	Flavonoids	Flavonols	C22H22O12	6743-96-0	209511	167275	259866	116343	161881	166348	0.83724	0.12481	0.16616	1.432053597
Apigenin 7-(6''-malonylglucoside	Flavonoids	Flavones	C24H22O13	86546-87-4	10576.8	10576.8	10576.8	52884.1	60959	80787.6	1.15374	0.02252	0.040638	0.163028912
p-Coumaroylputrescine	Alkaloids	Phenolamine	C13H18N2O2	34136-53-3	64702.4	94703.7	101923	9649.28	9649.28	9649.28	1.1547	0.02097	0.038456	9.02760003
Gaultherin	Phenolic acids	Phenolic acids	C19H26O12	490-67-5	22547	40149.5	73348.8	81413.6	118182	32526.9	0.52717	0.34298	0.392661	0.586091842
Forsythide	Terpenoids	Monoterpenoids	C16H22O11	43043-09-0	100337	100337	100337	501685	750104	732775	1.15376	0.01976	0.036853	0.15167607
Purpureoside*	Terpenoids	Monoterpenoids	C16H22O10	205127-53-3	452759	419269	412551	45187.8	45187.8	45187.8	1.16447	0.00105	0.006356	9.475855533
Phlorizin	Flavonoids	Chalcones	C21H22O10	4547-85-7	81683.8	75432.2	65238.8	19835.1	31609.4	36677.2	1.07711	0.00296	0.010955	2.52326936
16R-Sitsirikine	Alkaloids	Plumerane	C21H26N2O3	1245-00-7	23403.7	23403.7	23403.7	240936	174927	160350	1.15657	0.02093	0.03842	0.121849211
Paederoside	Terpenoids	Monoterpenoids	C18H22O11S	20547-45-9	13392.1	13392.1	13392.1	153046	149048	160297	1.16456	0.00055	0.004575	0.086887905
Luteolin-4'-O-glucoside*	Flavonoids	Flavones	C21H20O11	6920-38-3	523513	498616	699634	325925	385882	378018	1.02862	0.06928	0.10034	1.579851794
Phenylethyl alcohol	Others	Alcohol compounds	C8H10O	60-12-8	352203	352203	352203	1847697	2005039	1761015	1.16363	0.0022	0.009287	0.188218031
9-(4-methoxyphenyl)phenalen-1-one	Quinones	Quinones	C20H14O2	65874-43-3	431402	426312	408046	547189	651867	715224	1.08367	0.04516	0.069824	0.661219679
10-Hydroxy-2-Pinen-4-one 10-O-[β-D-Apiofuranosyl-(1-6)-β-D-Glucopyranoside	Terpenoids	Monoterpenoids	C21H32O11	1403967-09-8	527633	527633	527633	4122730	3559542	4706825	1.16106	0.00835	0.020626	0.1277655
Prunetin-4'-O-glucoside(Prunitrin)*	Flavonoids	Isoflavones	C22H22O10	154-36-9	1156968	1312604	1004261	858471	801915	780842	1.05354	0.05291	0.079602	1.422985372
Diosmetin-7-O-Neohesperidoside (Neodiosmin)	Flavonoids	Flavones	C28H32O15	38665-01-9	61088.9	75893.8	60280.2	12056	12056	12056	1.16025	0.00881	0.021231	5.45405278
Quinidine	Alkaloids	Quinoline alkaloids	C20H24N2O2	56-54-2	60312.5	213892	43988.7	182229	267717	321322	0.85499	0.09454	0.131048	0.412557968
4-Ethynylbenzaldehyde	Others	Aldehyde compounds	C9H6O	63697-96-1	3091402	2949023	2451861	925943	1022394	978284	1.15329	0.0096	0.022302	2.901737608
Tinocordiside	Terpenoids	Sesquiterpenoids	C21H32O7	191613-38-4	302208	234310	242358	18286.7	18286.7	18286.7	1.16283	0.00778	0.019798	14.19746892
16-hydroxy-17-[7-hydroxy-5-(1-hydroxypropan-2-yl)hept-5-en-2-yl]-10,13-dimethyl-3-[3,4,5-trihydroxy-6-(hydroxymethyl)oxan-2-yl]oxy-1,2,3,4,5,12,14,15,16,17-decahydrocyclopenta[a]phenanthr	Steroids	Steroidal saponins	C35H54O10	1501940-12-0	239700	210520	265043	26616	26616	26616	1.1625	0.00549	0.015765	8.957797761
16-Hydroxytrichodiene	Terpenoids	Sesquiterpenoids	C15H24O	185054-67-5	48302.4	23697.7	47690.4	4739.55	4739.55	4739.55	1.13607	0.04921	0.074921	8.417862159
Met-Tyr	Amino acids and derivatives	Amino acids and derivatives	C14H20N2O4S	13589-04-3	24837	18078.5	24939.9	8776.98	8655.77	21524.4	0.82535	0.13898	0.18144	1.74179726
Delta-	Others	Lactones	C6H10O2	823-22-3	7812.67	7812.67	7812.67	39063.4	70023.2	77511.1	1.14006	0.04377	0.068173	0.125607262
Scopine	Alkaloids	Tropan alkaloids	C8H13NO2	498-45-3	299982	249850	153132	203743	304656	208933	0.09663	0.93407	0.944745	0.979969074
1(10),15-Rosadiene-2,12-dione	Terpenoids	Ditepenoids	C20H28O2	913059-57-1	1167.46	1167.46	1167.46	11209.8	5837.29	6467.33	1.13778	0.05896	0.087222	0.148946011
Isocupressic acid	Terpenoids	Ditepenoids	C20H32O3	1909-91-7	28164.9	16762.9	24804.6	2922.06	2922.06	2922.06	1.15162	0.02661	0.045687	7.954701296
Nootkatone	Terpenoids	Sesquiterpenoids	C15H22O	4674-50-4	38628.9	48443	31588.3	5178.24	5178.24	5178.24	1.15634	0.01962	0.036686	7.638395158
2',6'-Dihydroxyacetophenone	Others	Ketone compounds	C8H8O3	699-83-2	37208.1	16551.2	22069.3	55668.2	35545.6	24937.5	0.63123	0.29403	0.342698	0.652843311
Asn-Arg	Amino acids and derivatives	Amino acids and derivatives	C10H20N6O4	2478-01-5	41540.3	31572	35041.9	5342.03	5342.03	5342.03	1.16087	0.00893	0.021264	6.748631307
Luteolin 5-glucoside	Flavonoids	Flavones	C21H20O11	20344-46-1	141996	55796.7	140979	11159.3	11159.3	11159.3	1.12216	0.07055	0.102044	10.11922141
2-Aminopurine	Nucleotides and derivatives	Nucleotides and derivatives	C5H5N5	452-06-2	95191.4	265236	208056	122324	62564.2	82781.1	0.8078	0.1733	0.219043	2.123824194
Cyclopenta[c]pyran-3(5H)-one	Others	Others	C8H6O2	880161-56-8	3625.83	3625.83	3625.83	28470.9	25249.9	20193.7	1.15832	0.01289	0.027017	0.147163067
5,7,3',5'-Tetrahydroxy-3,6,8,4'-tetramethoxyflavone 3'-glucoside	Flavonoids	Flavonols	C25H28O15	101021-27-6	6381.91	6381.91	6381.91	61792.7	54073.6	31909.5	1.14193	0.04097	0.064657	0.129559229
Robinetin	Flavonoids	Flavonols	C15H10O7	490-31-3	186322	142432	172448	98688.8	121197	157533	0.79867	0.1325	0.174677	1.32797479
Apigenin-5-O-glucoside	Flavonoids	Flavones	C21H20O10	28757-27-9	9693.85	9693.85	9693.85	48469.2	72779.4	78322.8	1.15086	0.02505	0.043872	0.145719975
6-Methoxymellein	Lignans and Coumarins	Coumarins	C11H12O4	13410-15-6	77543.1	115000	94552.6	12910.3	12910.3	12910.3	1.15699	0.01668	0.032524	7.412578435
3,3',5,7-Tetrahydroxy-4',6-Dimethoxyflavon	Flavonoids	Flavonols	C17H14O8	74161-28-7	5567.88	5567.88	5567.88	29501.6	52771.4	27839.4	1.13751	0.06078	0.08954	0.151696393
1,4,8-Trihydroxynaphthalene-1-O-glucoside	Quinones	Quinones	C16H18O8	39015-63-9	2578706	2396784	2198303	1391637	1680458	1466597	1.11067	0.00392	0.012878	1.580585608
Maytensifolone	Terpenoids	Triterpene	C30H44O5	1581689-19-1	55720	61231.4	74333.1	4145.78	4145.78	4145.78	1.16227	0.00846	0.020738	15.3798749
Cirsimaritin*	Flavonoids	Flavones	C17H14O6	6601-62-3	43464.3	50093.1	59957.1	4413.25	4413.25	4413.25	1.1611	0.01034	0.023209	11.59497126
Farnesyl acetone	Terpenoids	Ditepenoids	C18H30O	1117-52-8	32453.9	32996.6	34390.7	2980.57	2980.57	2980.57	1.16459	0.00036	0.003654	11.16576574
Methyl	Phenolic acids	Phenolic acids	C9H10O4	3187-58-4	30009.6	30009.6	30009.6	237876	281275	223312	1.16238	0.00635	0.017233	0.121256828
N-p-Coumaroyloctopamine	Alkaloids	Phenolamine	C17H17NO4	66648-45-1	548168	401559	720492	482753	795227	516681	0.18875	0.77446	0.803235	0.930659395

4-hydroxy-tryptamine	Alkaloids	Plumerane	C10H12N2O	570-14-9	204623	283243	134098	26819.6	26819.6	26819.6	1.13883	0.05251	0.079117	7.730231925
Sinensetin	Flavonoids	Flavones	C20H20O7	2306-27-6	1038.53	1038.53	1038.53	10076.2	6673.08	5192.65	1.14157	0.04919	0.074921	0.141992764
8-Hydroxycoumari	Lignans and Coumarins	Coumarins	C9H6O3	2442-31-1	605423	441327	403268	864552	471882	762134	0.72282	0.20144	0.247838	0.690956001
(+)-Perillyl alcohol*	Terpenoids	Monoterpenoids	C10H16O	57717-97-2	690246	634578	675092	126916	126916	126916	1.16425	0.00095	0.006021	5.252614641
Icariside B2	Terpenoids	Sesquiterpenoids	C19H30O8	108906-51-0	85576.3	85576.3	85576.3	464403	427881	596973	1.15706	0.01528	0.030633	0.172387162
(9E)-Octadecenoic	Lipids	Free fatty acids	C18H34O2	112-79-8	3644803	3845708	3538796	1332521	1617854	1598335	1.15153	7.4E-05	0.001618	2.424711501
(6Z)-Octadecenoic	Lipids	Free fatty acids	C18H34O2	593-39-5	1234263	1309227	1197942	452646	511990	543435	1.15483	8.1E-05	0.001679	2.480938085
D-Erythrose 4-phosphate	Others	Saccharides	C4H9O7P	585-18-2	164904	181677	213729	111328	108694	126820	1.08921	0.02575	0.04469	1.615468267
Sorbitol 6-phosphate	Others	Saccharides	C6H15O9P	20479-58-7	35782.4	31837.2	27528	33466	43107.9	31823.1	0.54139	0.36494	0.415355	0.877769364
Pinen-10-yl vicianoside	Terpenoids	Terpene	C21H34O10	88623-94-3	786738	779973	753321	3334204	4203301	3698633	1.16048	0.00704	0.018522	0.206479529
D-Threonate	Others	Saccharides	C4H8O5	3909-12-4	355600	381613	359293	123258	131770	130060	1.16277	0.00045	0.004048	2.847423966
3',5'-Cyclic AMP	Nucleotides and derivatives	Nucleotides and derivatives	C10H12N5O6P	60-92-4	1051564	878610	872352	315360	307753	328038	1.15708	0.00842	0.02067	2.946457245
Nepetariaside	Others	Others	C16H28O8	110344-61-1	731528	789565	954182	1008953	781932	941594	0.4825	0.41674	0.46659	0.905871578
(-)-Pinoresinol glucoside	Lignans and Coumarins	Lignans	C26H32O11	41607-20-9	104188	110057	99494.1	253416	203062	265445	1.13903	0.01717	0.033134	0.434587123
(+)-Syringaresinol O-beta-D-glucoside	Lignans and Coumarins	Lignans	C28H36O13	7374-79-0	3332300	3397356	3404464	888230	986015	852804	1.16204	7.8E-06	0.00069	3.716147316
2,3,6-Trihydroxyurs-12-en-28-oic acid (Madasiatic	Terpenoids	Triterpene	C30H48O5	26532-66-1	3627743	3061102	3273027	8382280	7967926	8546799	1.15656	3.2E-05	0.001173	0.400123277
DL-2-hydroxystearic acid*	Lipids	Free fatty acids	C18H36O3	629-22-1	629857	610676	585973	85022.3	98287.4	97733.6	1.16301	0.00018	0.002464	6.499018957
5'-Methoxymataires inoside*	Lignans and Coumarins	Lignans	C27H34O12	1691201-82-7	332854	437849	405180	175681	190081	204289	1.12742	0.01743	0.03352	2.062770855
2-Hydroxyhexadec anoic acid*	Lipids	Free fatty acids	C16H32O3	764-67-0	2643990	2517960	2977734	608951	626980	647758	1.16153	0.00409	0.013197	4.321140113
3-Hydroxy-24-methylene-9,19-cyclolanostan-26-oic acid (Ambolic acid)*	Terpenoids	Triterpene	C31H50O3	13878-93-8	91581.6	97473.4	144889	43659.6	51669.8	75121	0.98177	0.06336	0.092802	1.959185891
Methyl palmitate	Lipids	Free fatty acids	C17H34O2	112-39-0	871592	783047	540539	399971	308418	464346	0.99865	0.057	0.084654	1.871844341
Isochlorogenic acid b	Phenolic acids	Phenolic acids	C25H24O12	14534-61-3	4338598	4340463	4712433	1.4E+07	1.5E+07	1.2E+07	1.15841	0.00485	0.014585	0.331858202
Rosmarinyl glucoside	Phenolic acids	Phenolic acids	C24H26O13	910028-78-3	152538	142735	149908	203273	202108	204233	1.1552	0.00201	0.008839	0.73026549
Momordin Ic*	Terpenoids	Triterpene	C41H64O13	96990-18-0	287088	294205	268234	160657	226822	221294	0.96965	0.04978	0.075674	1.395473397
(7S,8S)-DiHODE*	Lipids	Free fatty acids	C18H32O4	143288-65-7	46363.4	48550.6	43385.6	54959.8	52967.5	53478.7	1.06371	0.02351	0.041745	0.85684369
Kaempferol-7-O-glucoside*	Flavonoids	Flavonols	C21H20O11	16290-07-6	188710	201578	227575	65045.6	62206.6	57474	1.15756	0.00489	0.014667	3.34474856
Nortrachelogenin-4-O-glucoside	Lignans and Coumarins	Lignans	C26H32O12	33464-78-7	99033.6	109315	90616.2	42215.9	57360.7	46898.5	1.11785	0.0022	0.009283	2.041061527
Genistin	Flavonoids	Isoflavones	C21H20O10	529-59-9	195520	183536	179282	125723	106015	123871	1.12636	0.00137	0.007153	1.570088893
Emodin 8-glucoside*	Quinones	Anthraquinone	C21H20O10	23313-21-5	1799557	1948737	1888854	2184770	2425209	2538678	1.07285	0.02672	0.045839	0.788560324
Naringenin 7-O-beta-D-glucoside	Flavonoids	Flavanones	C21H22O10	529-55-5	1560288	1627800	1495356	883368	1115131	1033267	1.10524	0.00493	0.014728	1.544790725
Astringin	Others	Stilbene	C20H22O9	29884-49-9	61296.3	44819.5	53954.2	163434	187724	220844	1.14249	0.00985	0.022547	0.27984159
Deacetyl asperulosidic acid methyl ester	Terpenoids	Monoterpenoids	C17H24O11	52613-28-2	603641	838286	925046	932814	1020499	898458	0.70034	0.22856	0.275298	0.830001046
6'-O-α-D-galactosylshanzhi side methyl ester	Terpenoids	Monoterpenoids	C23H36O16	927707-47-9	91612.3	98136.8	151693	366254	444886	460293	1.12837	0.00169	0.008	0.268548882
Oroxin B	Flavonoids	Flavones	C27H30O15	114482-86-9	6E+07	5.7E+07	4.9E+07	2.7E+07	2.7E+07	3.2E+07	1.13409	0.00528	0.015388	1.928705
4-O-(3'-O-alpha-D-Glucopyranosyl)c affeoylquinic	Phenolic acids	Phenolic acids	C22H28O14	1401352-63-3	696485	571050	606276	1551651	1476691	1553940	1.15368	9.4E-05	0.001821	0.408925304
Quercetin-4'-O-glucoside (Spiraeoside)*	Flavonoids	Flavonols	C21H20O12	20229-56-5	2943655	3245363	3235059	1660307	1786485	2458816	1.03388	0.02872	0.048336	1.595784332
Quercimeritrin	Flavonoids	Flavonols	C21H20O12	491-50-9	3161570	2599401	2971821	1294143	2012501	2084420	0.97457	0.02714	0.046349	1.619863966
1,4-Dihydroxy-2-methylanthraquin one*	Quinones	Anthraquinone	C15H10O4	2589-39-1	668265	649177	631348	554573	499603	418751	1.00619	0.04837	0.073775	1.323073658
Desmethylagrimo nolide-6-O-glucoside*	Lignans and Coumarins	Coumarins	C23H26O10	1257408-55-1	155196	177847	184176	47588.1	63596.4	80038.4	1.11083	0.00109	0.006484	2.704794564
3-(Methoxycarbony l)propyl-beta-D-glucopyranoside	Organic acids	Organic acids	C11H20O8	218607-09-1	443967	361347	399478	370883	388604	455215	0.0514	0.92948	0.940719	0.991841736
Aloe emodin-1-O-glucoside*	Quinones	Anthraquinone	C21H20O10	266997-58-4	660306	613357	540468	135764	195476	155592	1.14571	0.00158	0.007676	3.726400924
Aloeemodin-8-O-glucoside*	Quinones	Anthraquinone	C21H20O10	33037-46-6	567537	472622	642835	142321	122803	185559	1.13591	0.00756	0.019457	3.734324401
3-Methoxyapigenin	Flavonoids	Flavonols	C16H12O6	1592-70-7	4391022	4536089	4701134	1591820	1757452	1933270	1.15555	3.3E-05	0.001173	2.579864985
Lamalbid	Terpenoids	Monoterpenoids	C17H26O12	52212-87-0	1248243	1465980	1395359	1005074	1126931	1091338	1.04716	0.02527	0.044087	1.274944293
Anthragallol 2-Methyl Ether; 1,3-Dihydroxy-2-methoxyanthraqu inone	Quinones	Anthraquinone	C15H10O5	10383-63-8	593930	558056	567598	81852	82741.9	90083.8	1.16402	0.00025	0.002941	6.752001922
7β-Hydroxydarutige nol	Terpenoids	Ditepenoids	C20H34O4	1188281-99-3	13462.6	12895.6	11449.4	13551.5	16995.8	11392.5	0.40282	0.49565	0.542908	0.901473342

Medicagenic acid 3-O-triglucoside	Terpenoids	Triterpene Saponin	C48H76O21	37838-43-0	2272807	2133781	2646010	782720	1127501	842404	1.12359	0.00241	0.009837	2.562135672
Embelin	Quinones	Quinones	C17H26O4	550-24-3	336121	679631	323787	373509	386076	390516	0.21543	0.64254	0.682171	1.164714257
8,15-Dihydroxy-5,9,11,13-eicosatetraenoic acid	Lipids	Free fatty acids	C20H32O4	77667-08-4	457627	437631	460068	813101	878161	911933	1.15729	0.0032	0.011439	0.520639957
3,7-Di-O-methylquercetin	Flavonoids	Flavonols	C17H14O7	2068-02-2	30000.1	25836.1	20946	24005.6	42215.1	51204	0.71253	0.22687	0.273692	0.653884893
Morindaparvin A	Quinones	Anthraquinone	C15H8O4	41621-32-3	1128586	1115219	1196914	892584	965966	917410	1.11575	0.00287	0.010772	1.239470524
3,19-Epoxy-3,22-dihydroxydammara-20,24-dien-26-oic acid δ-lactone (Semialactone)	Terpenoids	Triterpene	C30H44O4	366450-46-6	146070	152555	158795	167661	168621	147507	0.56247	0.34062	0.390253	0.945495138
N-Acetyl-D-glucosamine 1-phosphate	Others	Saccharides	C8H16NO9P	6866-69-9	37514	37454.7	35899.4	14138.9	17634.3	10595.9	1.11492	0.00527	0.015388	2.616720951
Emodin-6-O-glucoside*	Quinones	Anthraquinone	C21H20O10	34298-85-6	1786478	1918666	1956059	1916180	2301137	2266962	0.83615	0.14265	0.185691	0.873066017
Medicarpin	Flavonoids	Isoflavones	C16H14O4	32383-76-9	4095909	3801299	3867637	2026516	2238299	2136257	1.15653	0.00018	0.002464	1.837949114
Azelaic acid	Lipids	Free fatty acids	C9H16O4	123-99-9	1133536	1252930	1049425	910395	1225561	1087507	0.37564	0.5557	0.601733	1.065900637
2,6-Dimethyl-7-octene-2,3,6-triol	Terpenoids	Monoterpenoids	C10H20O3	73815-21-1	178434	175627	196565	141484	164995	151477	0.99131	0.03097	0.051369	1.202357328
Apigenin 7-O-neohesperidoside	Flavonoids	Flavones	C27H30O14	17306-46-6	1694780	1944207	1804741	1258426	1473608	1601112	0.95345	0.045	0.069704	1.256299055
Cimidahurinine	Phenolic acids	Phenolic acids	C14H20O8	142542-89-0	361198	368502	391854	64412.5	109730	79701.8	1.13996	0.00013	0.00213	4.418283694
Agrimonolide-6-O-glucoside	Lignans and Coumarins	Coumarins	C24H28O10	126223-29-8	80459.7	92946.9	92744.1	19434.3	18248.8	17179.4	1.16127	0.00283	0.01064	4.851223637
Caffeate	Phenolic acids	Phenolic acids	C9H8O4	331-39-5	874631	1041484	898697	1269575	1068691	919950	0.63659	0.28551	0.333528	0.863911837
D-Ribulose 5-phosphate	Others	Saccharides	C5H11O8P	4151-19-3	93061.4	111862	131039	118776	106850	104703	0.0446	0.88504	0.901626	1.0170574
(R,R)-Tartaric acid	Organic acids	Organic acids	C4H6O6	87-69-4	58040.5	62433	79198.1	65571.1	47997.5	58748.6	0.56744	0.33296	0.382323	1.158743701
Asiatic acid*	Terpenoids	Triterpene	C30H48O5	464-92-6	3052371	2672302	2903077	7348570	7547189	7956806	1.15965	9.3E-05	0.001821	0.377539726
4-p-Coumaroylquinic acid	Phenolic acids	Phenolic acids	C16H18O8	93451-44-6	2855162	2901299	2777802	3608582	3948745	3997047	1.13527	0.00969	0.022391	0.738617569
sn-Glycero-3-phospho-1-	Others	Saccharides	C9H19O11P	129830-95-1	69068.4	97566.6	62142.4	100172	65613.6	98902.8	0.40885	0.48754	0.535597	0.864327757
N-Propionylglycine	Amino acids and derivatives	Amino acids and derivatives	C5H9NO3	21709-90-0	78847.2	78171.7	61227.1	47986.7	37904.9	32270.7	1.06008	0.01187	0.02558	1.847000822
Sedoheptulose 7-phosphate	Others	Saccharides	C7H15O10P	2646-35-7	284547	228670	289222	207005	254246	308605	0.20369	0.77535	0.803894	1.042323792
UDP-D-xylose	Nucleotides and derivatives	Nucleotides and derivatives	C14H22N2O16P2	3616-06-6	50868.4	43715.8	45610	42804	47394	36618.3	0.59431	0.31182	0.360188	1.105489396
2-Deoxy-D-ribose 5-	Nucleotides and derivatives	Nucleotides and derivatives	C5H11O7P	102916-66-5	18301.9	18163.7	16138.1	6049.45	10265.5	11516.1	0.99756	0.02436	0.042872	1.890107081
Kaempferol-3-O-galactoside (Trifolin)*	Flavonoids	Flavonols	C21H20O11	23627-87-4	514145	618069	649002	280066	353627	386940	1.07034	0.00929	0.021724	1.745207824
2α,3α-Dihydroxyolean-12-en-28-oic acid	Terpenoids	Triterpene	C30H48O4	26563-68-8	234736	234966	226247	92219.7	97149.6	110516	1.15504	0.00021	0.002662	2.320719202
Linolelaidic acid ethyl ester*	Lipids	Free fatty acids	C20H36O2	544-35-4	171960	165347	141418	58344	58400.2	59444.4	1.15683	0.00828	0.020528	2.717118269
9(10)-EpOME	Lipids	Free fatty acids	C18H32O3	16833-56-0	37043.9	29877.9	26557.3	7812.74	5494.85	5272.51	1.14379	0.01095	0.024102	5.031145936
Phosphoenolpyruvate	Organic acids	Organic acids	C3H5O6P	138-08-9	65051.8	89879.4	62391.6	24373.8	33454.6	23093.4	1.10559	0.02401	0.04238	2.685589253
Ethyl ferulate	Phenolic acids	Phenolic acids	C12H14O4	4046-02-0	83332.1	94921.3	87728.5	42694.3	43445.7	44001.1	1.15773	0.00507	0.015014	2.043796066
Isochlorogenic acid C	Phenolic acids	Phenolic acids	C25H24O12	57378-72-0	4676585	4880263	5215315	1.2E+07	1.4E+07	1.3E+07	1.15955	0.00147	0.007425	0.379764775
Succinic acid*	Organic acids	Organic acids	C4H6O4	110-15-6	1.7E+07	1.7E+07	1.3E+07	4458924	6397111	7510193	1.09688	0.00363	0.012331	2.580515561
3,4-Dimethoxycinnamic acid	Phenolic acids	Phenolic acids	C11H12O4	2316-26-9	171622	185779	181137	64865.3	58682.3	59342.6	1.16164	0.00019	0.002534	2.944598646
Glycyrrhetinate	Terpenoids	Triterpene	C30H46O4	471-53-4	35058.9	29325.7	27458.7	16417.9	16517.7	12770.6	1.10997	0.009	0.021308	2.009424025
Iminodiacetate*	Amino acids and derivatives	Amino acids and derivatives	C4H7NO4	142-73-4	26984.3	25127.6	28097.8	74275.8	76599.8	64662.5	1.156	0.00457	0.014036	0.372137127
Gnetifolin B*	Flavonoids	Flavonols	C16H12O6	140671-06-3	4683702	5057811	4974525	1900326	2149335	2313109	1.15211	7.4E-05	0.001618	2.312835238
Aracarpene 1*	Flavonoids	Isoflavones	C16H12O6	1261245-78-6	2885549	2985286	2917278	1118311	1251579	1089297	1.1595	4.2E-05	0.001277	2.540513669
alpha-D-Glucosamine 1-phosphate	Others	Saccharides	C6H14NO8P	2152-75-2	15556.8	9977.61	16335.5	8951.87	12225.4	10166.3	0.70289	0.21605	0.262794	1.335836984
Gingerglycolipid A	Lipids	Glycerol ester	C33H56O14	145937-22-0	178574	179107	180250	133591	144345	134761	1.14482	0.00576	0.016212	1.303456326
(+)-Pinoresinol*	Lignans and Coumarins	Lignans	C20H22O6	487-36-5	19729.1	13742.8	10474.5	135494	144674	155703	1.14929	0.00038	0.00378	0.100824411
Epipinoresinol*	Lignans and Coumarins	Lignans	C20H22O6	18779-41-4	19396.3	15189.1	16469.7	114036	127931	147137	1.15865	0.00646	0.017435	0.131212039
D-Arabinono-1,4-lactone	Others	Saccharides	C5H8O5	2782-09-4	201395	260441	187270	183454	170123	146104	0.84856	0.14278	0.185773	1.29903921
Undecanedioic acid	Lipids	Free fatty acids	C11H20O4	1852-04-6	45834.5	52124.3	50363.8	72068.4	55854	62005.3	0.96063	0.08389	0.118992	0.780941995
3-Hydroxyoctadecanoic Acid	Lipids	Free fatty acids	C18H36O3	45261-96-9	462757	424534	394539	1026907	786185	597204	1.02618	0.09062	0.126625	0.531814176
Prunetin (5,4'-Dihydroxy-7-methoxyisoflavone)	Flavonoids	Isoflavones	C16H12O5	552-59-0	1.9E+07	2E+07	2.1E+07	5930570	6353256	6793262	1.16077	0.00015	0.002276	3.117775392
Strophanthobiose	Others	Others	C13H24O9	7724-04-1	114130	103360	92288.8	224846	229712	230347	1.15042	0.00138	0.007153	0.452293647
Chlorogenate*	Phenolic acids	Phenolic acids	C16H18O9	327-97-9	3.2E+07	2.9E+07	3.8E+07	6E+07	5.3E+07	6.1E+07	1.11364	0.00242	0.009848	0.568343019
2,4-Dihydroxybenzoic acid	Phenolic acids	Phenolic acids	C7H6O4	89-86-1	666442	899925	1042038	595554	759478	945037	0.37017	0.5283	0.57508	1.134055133
2-Hydroxy-2-methylpropanoate*	Organic acids	Organic acids	C4H8O3	594-61-6	373051	338456	396897	96563.8	103271	92324.1	1.1604	0.00298	0.010992	3.793834459
Orotate	Others	Vitamin	C5H4N2O4	65-86-1	964542	827331	1033340	693529	576814	584517	1.0733	0.0158	0.03145	1.523140282
Uracil	Nucleotides and derivatives	Nucleotides and derivatives	C4H4N2O2	66-22-8	1404010	1594780	1509983	940771	918177	1085437	1.11575	0.0024	0.009833	1.531312533
(R)-2-Hydroxystearate*	Lipids	Free fatty acids	C18H36O3	26633-48-7	608423	602237	520370	83835.6	96549.9	96102.2	1.16165	0.00289	0.010802	6.260781862

Sinapoyl	Phenolic acids	Phenolic acids	C11H12O4	4206-58-0	80546.3	93537.8	113972	261338	362580	392771	1.12943	0.02075	0.038267	0.283328165
D-Maltose*	Others	Saccharides	C12H22O11	69-79-4	3609439	3632230	5036809	2386736	1923294	3040643	0.96956	0.05259	0.079208	1.670388288
Galactitol*	Others	Saccharides	C6H14O6	608-66-2	40909.5	54012.1	47418.3	36495.1	48860.8	43368.3	0.46113	0.43266	0.482328	1.105774102
D-Glucono-1,5-lactone*	Others	Saccharides	C6H10O6	90-80-2	97106	85403.9	73029	77578.7	80145.5	81442.6	0.39489	0.51593	0.562405	1.068454583
16,23:16,30-Diepoxydammar-24-ene-3,20-diol (Jujubogenin)*	Terpenoids	Triterpene	C30H48O4	54815-36-0	3.3E+07	3.4E+07	3.3E+07	1.3E+07	1.3E+07	1.5E+07	1.1601	1.8E-05	0.000937	2.375643021
(9Z,12Z,15Z)-Octadecatrienoic acid*	Lipids	Free fatty acids	C18H30O2	463-40-1	2.2E+07	2E+07	2.2E+07	9315461	8964684	9665559	1.16059	0.00087	0.00579	2.274534795
Benzyl β-primeveroside*	Others	Others	C18H26O10	130622-31-0	5776396	5397910	5221304	813378	1080668	1160060	1.15461	7.6E-05	0.00162	5.368382598
2-hydroxy-3-hydroxymethyl-anthraquinone	Quinones	Anthraquinone	C15H10O4	68243-30-1	308102	286759	305250	163323	166210	176391	1.15752	0.00028	0.003172	1.779145825
(-)-5'-Methoxyisolariciresinol 3alpha-O-beta-glucopyranoside	Lignans and Coumarins	Lignans	C27H36O12	143236-03-7	174081	137871	159024	53660.4	86700.6	76748.4	1.08006	0.00421	0.013452	2.169300479
confusarin	Quinones	PhenAnthraquinones	C17H16O5	108909-02-0	50969.9	37431.9	19477.7	24031.7	25948.1	26862.6	0.49463	0.37426	0.424554	1.403907364
Apigenin-4'-O-glucoside	Flavonoids	Flavones	C21H20O10	20486-34-4	5.4E+07	4.8E+07	4.9E+07	2.8E+07	3E+07	3.3E+07	1.13144	0.00191	0.008549	1.645361435
1,14-Tetradecanedioic Acid	Lipids	Free fatty acids	C14H26O4	821-38-5	49437.4	49369.4	44069	71140.3	60240.4	60942.9	1.06305	0.02569	0.044641	0.74289298
2-Hydroxy-3-(4-Hydroxyphenyl)Propanoic Acid	Phenolic acids	Phenolic acids	C9H10O4	23508-35-2	108665	120754	135087	147968	137406	136442	0.86329	0.11321	0.153023	0.864137305
3-Furoic acid	Organic acids	Organic acids	C5H4O3	488-93-7	71234.6	59270.5	73337.2	91340.5	64204.5	104933	0.67473	0.25134	0.29891	0.782569197
Persicoside*	Flavonoids	Flavanones	C23H26O11	28978-03-2	892093	990387	923365	257821	366512	324245	1.14251	0.00014	0.00217	2.957944535
Esculin*	Lignans and Coumarins	Coumarins	C15H16O9	531-75-9	23175.7	25416.1	22300.1	70080.7	86122.4	70354.9	1.15438	0.0087	0.021062	0.312908651
Cichoriin*	Lignans and Coumarins	Coumarins	C15H16O9	531-58-8	45751	16670.7	19333.4	62352.2	40455	53380.1	0.85469	0.10093	0.138716	0.523442334
Oleoside 11-methyl ester	Terpenoids	Monoterpenoids	C17H24O11	60539-23-3	162369	98764.3	126253	480286	619330	527629	1.13666	0.00362	0.012331	0.238063037
(+)-Mediresinol Di-O-beta-D-glucopyranoside	Lignans and Coumarins	Lignans	C33H44O17	88142-63-6	290788	217508	262577	49343	39622.7	36893.5	1.15455	0.00815	0.020271	6.124879055
Monomethyl succinate*	Organic acids	Organic acids	C5H8O4	3878-55-5	127584	124275	126306	115415	126916	112078	0.76099	0.21643	0.263011	1.067034213
Sesamoside	Terpenoids	Monoterpenoids	C17H24O12	117479-87-5	127007	172703	160370	701818	922890	665172	1.14856	0.01491	0.030055	0.200919094
2,4-Dihydroxybenzaldehyde	Others	Aldehyde compounds	C7H6O3	95-01-2	23545.1	36717.8	25741.8	63532	54921.7	57452.4	1.08577	0.0059	0.016502	0.488923683
Purpurin 1-methyl ether	Quinones	Anthraquinone	C15H10O5	94099-66-8	178115	143945	159152	82448	95197.1	78272.8	1.12485	0.00669	0.017913	1.880336031
(6Z,9Z,12Z)-Octadecatrienoic acid*	Lipids	Free fatty acids	C18H30O2	506-26-3	4.8E+07	4.7E+07	5.1E+07	2.1E+07	2.1E+07	2.1E+07	1.16222	0.00168	0.007968	2.336401321
Crepenynic acid	Lipids	Free fatty acids	C18H30O2	2277-31-8	137952	128104	141812	53494.7	53763.9	56220.6	1.16146	0.00173	0.008085	2.494920822
Rumenic acid	Lipids	Free fatty acids	C18H32O2	2540-56-9	27657.9	23743.4	25051.2	16454.9	12505.1	11225.9	1.0916	0.00458	0.014048	1.902468977
Secologanoside	Terpenoids	Monoterpenoids	C16H22O10	59472-23-0	1908530	2013995	2040786	1286153	1342382	1312553	1.15659	0.00117	0.006662	1.513112788
Ferulic acid methyl ester	Phenolic acids	Phenolic acids	C11H12O4	22329-76-6	433958	437957	451743	136983	162308	159982	1.1582	2.6E-05	0.001112	2.882070507
Xanthurenic Acid 8-O-	Alkaloids	Quinoline alkaloids	C16H17NO9	97451-32-6	510545	529219	587879	274764	321362	295635	1.14008	0.00214	0.009168	1.825200399
4-Hydroxybenzaldehyde	Others	Aldehyde compounds	C7H6O2	123-08-0	1859187	1811226	1780877	838129	935630	954137	1.15625	0.0001	0.001864	1.998349543
3-Hydroxyurs-12-en-28-oic acid (3-Epiursolic	Terpenoids	Triterpene	C30H48O3	989-30-0	160987	150890	158427	58296.3	43937.6	44758.6	1.15044	0.00012	0.002129	3.199513313
Urolignoside*	Lignans and Coumarins	Lignans	C26H34O11	131723-83-6	150820	145831	166384	155015	187906	227982	0.7503	0.22526	0.271966	0.811058027
Verminoside*	Terpenoids	Monoterpenoids	C24H28O13	50932-19-9	1539385	1559783	1738460	2209090	2213147	2086595	1.11978	0.00315	0.011309	0.743240625
2-Phenylethyl beta-primeveroside	Others	Alcohol compounds	C19H28O10	129932-48-5	870644	619655	809306	164141	185607	221050	1.14361	0.01351	0.027985	4.028757534
Icariside E5*	Lignans and Coumarins	Lignans	C26H34O11	126176-79-2	109199	184058	133403	201101	171817	190666	0.79966	0.16368	0.20868	0.757047187
Quercetin-4'-O-glucuronide*	Flavonoids	Flavonols	C21H18O13	201463-36-7	532971	439285	421182	305270	310387	400698	0.94826	0.05457	0.081593	1.371015282
Gardenoside	Terpenoids	Monoterpenoids	C17H24O11	24512-62-7	80101.8	85985.1	74606.9	109765	80599.7	100542	0.78171	0.18199	0.227982	0.827389489
Quinizarin; 1,4-Dihydroxyanthraquinone*	Quinones	Anthraquinone	C14H8O4	81-64-1	2037594	1817282	1980660	1627214	1837073	1660652	0.91692	0.06301	0.09243	1.138654561
5-O-Caffeoylshikimic acid	Phenolic acids	Phenolic acids	C16H16O8	73263-62-4	1962218	1743296	1766684	1601071	2061391	2157956	0.30703	0.58105	0.625653	0.940172695
23-Hydroxy-3-oxoolean-12-en-28-oic acid (Hederagonic acid)	Terpenoids	Triterpene	C30H46O4	466-01-3	13320.9	10312	14048.2	5046.35	7530.09	9364.9	0.94194	0.03679	0.05909	1.717359091
3',5'-Cyclic GMP	Nucleotides and derivatives	Nucleotides and derivatives	C10H12N5O7P	7665-99-8	556134	680037	696483	194856	214169	265946	1.13694	0.00398	0.013022	2.863312119
Phlorizin	Flavonoids	Chalcones	C21H24O10	60-81-1	13561.7	11590.2	13019.8	7893.15	10850.5	9575.73	0.96205	0.04041	0.063966	1.347898586
Pantetheine	Alkaloids	Alkaloids	C11H22N2O4S	496-65-1	21149.6	26321.4	22467.5	10409.3	8110.01	12165.7	1.10835	0.00328	0.011599	2.279243402
4-tert-	Others	Others	C14H22O	140-66-9	23008.5	19642.4	23803.3	22221.1	19756.4	21078.4	0.40227	0.4923	0.540257	1.053893036
10-Hydroxydecanoic acid	Lipids	Free fatty acids	C10H20O3	1679-53-4	8774.91	7181.28	4677.31	27152.1	34040.3	30194.8	1.1267	0.00139	0.007186	0.225781028
3-Dehydroshikimat	Organic acids	Organic acids	C7H8O5	2922-42-1	166825	149210	200422	364135	270717	292560	1.07309	0.02258	0.040675	0.556880182
myo-Inositol	Others	Saccharides	C6H12O6	87-89-8	762813	666994	830716	1520211	1417292	1286163	1.12867	0.00215	0.009176	0.535204107
Chrysophanic acid 9-anthrone	Quinones	Anthraquinone	C15H12O3	491-58-7	884488	808689	918281	1005259	795534	861109	0.11577	0.82533	0.850589	0.981049662
N-Acetylornithine	Amino acids and derivatives	Amino acids and derivatives	C7H14N2O3	6205-08-9	42583.8	33408.4	43677.3	19291.5	15608	29173.6	0.98987	0.02529	0.044087	1.867702177

Aloesone	Phenolic acids	Phenolic acids	C13H12O4	40738-40-7	31881.9	22615.2	27050	7217.19	11359.7	8666.55	1.11597	0.01061	0.023579	2.993273684
1-O-Gentisoyl-β-D-glucoside*	Phenolic acids	Phenolic acids	C13H16O9	23445-11-6	1E+07	9628575	9623774	3488047	3713529	3234073	1.16018	0.00012	0.002073	2.836532739
1-Decanol*	Others	Alcohol compounds	C10H22O	112-30-1	2771387	2409700	2382358	2477790	2633885	2202039	0.26988	0.66423	0.702318	1.034145607
Sucrose 6-phosphate	Others	Saccharides	C12H23O14P	22372-29-8	60759.9	65164.6	61716.6	29753.4	50260.8	48593.6	0.88894	0.09039	0.126416	1.459017928
2-Decanol*	Others	Alcohol compounds	C10H22O	1120-06-5	5015632	4318928	4044439	4454066	4523922	4065981	0.18194	0.75241	0.784572	1.025684567
Morroniside	Terpenoids	Monoterpenoids	C17H26O11	25406-64-8	2513601	2402098	2415707	886319	976645	778657	1.15481	8.6E-05	0.001732	2.775343438
Glutathione disulfide	Amino acids and derivatives	Amino acids and derivatives	C20H32N6O12S2	27025-41-8	114250	153593	140949	32523.5	57573.4	40200.2	1.10727	0.00443	0.013801	3.137383506
Abietate*	Terpenoids	Ditepenoids	C20H30O2	514-10-3	112388	110142	87320.8	250964	176388	118893	0.87624	0.17081	0.216516	0.567238906
D-Glucose 6-phosphate*	Others	Saccharides	C6H13O9P	56-73-5	3314676	3202549	2725012	3543822	2671747	2808394	0.15613	0.83565	0.859619	1.024188385
alpha-D-Glucose 1,6-bisphosphate	Others	Saccharides	C6H14O12P2	10139-18-1	168789	116528	127539	70881.6	91194.2	99905	0.96844	0.06696	0.097396	1.575900368
1-O-Sinapoyl-β-D-glucose*	Phenolic acids	Phenolic acids	C17H22O10	78185-48-5	2217004	2604446	2173468	1032085	1543088	1319167	1.06662	0.00696	0.018368	1.796175633
Roseoside	Terpenoids	Sesquiterpenoids	C19H30O8	54835-70-0	6170255	6138938	6697826	2430156	4094134	3937660	1.02132	0.02341	0.041664	1.816775796
Benzyl 6-O-beta-D-apiofuranosyl-beta-D-	Others	Alcohol compounds	C18H26O10	115009-57-9	2E+07	1.4E+07	1.5E+07	4125353	6414677	4966507	1.11772	0.01818	0.034589	3.149682612
Xanthosine	Nucleotides and derivatives	Nucleotides and derivatives	C10H12N4O6	146-80-5	95145.3	92751.9	68312.2	90635.3	91067.8	71367.3	0.036	0.92751	0.939335	1.012403993
trans-2-Hydroxycinnamate*	Phenolic acids	Phenolic acids	C9H8O3	583-17-5	1647073	1677164	1504244	2019313	2653733	2801935	1.04226	0.06052	0.0892	0.645952327
Loganate	Terpenoids	Monoterpenoids	C16H24O10	22255-40-9	4.1E+07	3.8E+07	4.1E+07	2.4E+07	3.1E+07	3.2E+07	1.01475	0.034	0.055355	1.380398872
Mussaenosidic acid*	Terpenoids	Monoterpenoids	C16H24O10	82451-22-7	3E+07	2.7E+07	2.7E+07	1.6E+07	2.1E+07	1.7E+07	1.07692	0.00891	0.021264	1.534168651
5-Nitrobenzimidazole	Alkaloids	Alkaloids	C7H5N3O2	94-52-0	320353	317103	332462	628290	671984	767338	1.15015	0.01155	0.025155	0.469100619
Sinapate	Phenolic acids	Phenolic acids	C11H12O5	530-59-6	212659	124164	126461	5381.46	21767.8	44730.9	0.99875	0.033	0.054071	6.445232063
D-Galactose*	Others	Saccharides	C6H12O6	59-23-4	1694364	1877348	1759586	1560832	1724478	2002081	0.09306	0.9238	0.935884	1.008303986
Malate	Organic acids	Organic acids	C4H6O5	636-61-3	6.1E+07	7.8E+07	7.8E+07	3.3E+07	3E+07	3.7E+07	1.12521	0.01188	0.02558	2.168779262
(-)-Jasmonic acid	Organic acids	Organic acids	C12H18O3	77026-92-7	313329	290809	321650	264646	301012	266390	0.83921	0.10991	0.149209	1.112662209
3,4-Dihydroxyphenyl acetate*	Phenolic acids	Phenolic acids	C8H8O4	102-32-9	2724021	2228563	2482025	265311	177577	203179	1.15836	0.00312	0.011262	11.50750019
2-Propylglutaric acid	Organic acids	Organic acids	C8H14O4	32806-62-5	186103	192562	176312	252218	341526	330818	1.08298	0.04507	0.06974	0.600260097
UDP-glucose	Nucleotides and derivatives	Nucleotides and derivatives	C15H24N2O17P2	133-89-1	625448	533883	581116	513999	529227	471059	0.89573	0.08597	0.121283	1.149351812
dGMP	Nucleotides and derivatives	Nucleotides and derivatives	C10H14N5O7P	902-04-5	190807	169281	189571	41641.7	59962.8	104262	1.03663	0.01588	0.031558	2.669986055
9,12,13-TriHOME	Lipids	Free fatty acids	C18H34O5	97134-11-7	534221	484985	482928	292840	283772	300256	1.1545	0.00389	0.012833	1.713066272
Isolariciresinol-9'-O-glucoside*	Lignans and Coumarins	Lignans	C26H34O11	63358-12-3	985950	906259	838245	232813	193808	178495	1.15662	0.00133	0.007025	4.51227964
2-Methyl-1,3,6-trihydroxy-9,10-anthraquinone	Quinones	Anthraquinone	C15H10O5	87686-86-0	285232	272523	275717	273836	252646	244608	0.85816	0.12529	0.166657	1.08090107
6-O-Acetylarbutin*	Others	Others	C14H18O8	10338-88-2	2187786	1853261	1428202	215118	302391	384308	1.13543	0.01651	0.032323	6.064699724
1-O-Caffeoyl-β-D-glucose*	Phenolic acids	Phenolic acids	C15H18O9	14364-08-0	183225	145860	162120	93274.5	73855.7	114557	1.04134	0.01213	0.025862	1.743793628
Koaburaside	Others	Others	C14H20O9	41653-73-0	704081	675321	679554	657383	605278	785982	0.08242	0.95513	0.962273	1.005033732
demethylregelin	Terpenoids	Triterpene	C30H46O4	173991-81-6	2370926	1753318	1886388	637363	435596	460238	1.13863	0.0094	0.021906	3.920326138
Methyl oleanolate*	Terpenoids	Triterpene	C31H50O3	1724-17-0	90286.2	111793	136112	51235.8	62579.1	53496.7	1.08578	0.04244	0.066541	2.021325152
(15Z)-Tetracosenoic	Lipids	Free fatty acids	C24H46O2	506-37-6	26463.5	29507.9	26001.1	22471.7	27652.4	30075.5	0.16677	0.82865	0.85316	1.022105754
Ethanolamine phosphate	Alkaloids	Alkaloids	C2H8NO4P	1071-23-4	39585.4	25214.6	34279.3	26273.3	31222.2	18971.7	0.64474	0.24428	0.291859	1.2957096
1-Octadecanol	Lipids	Free fatty acids	C18H38O	112-92-5	714993	726772	689597	787393	817908	848227	1.0894	0.01049	0.023395	0.868692906
4-Hydroxy-3-methoxy-benzaldehyde	Others	Aldehyde compounds	C8H8O3	121-33-5	147162	217100	190085	282026	370710	392391	1.0509	0.02128	0.038914	0.530411232
Methyl 4-hydroxybenzoate	Phenolic acids	Phenolic acids	C8H8O3	99-76-3	42582.5	29567.4	55608.6	95090.1	116189	97581.3	1.07284	0.00404	0.013098	0.413644524
Dehydroabietic acid*	Terpenoids	Ditepenoids	C20H28O2	1740-19-8	542716	452885	376170	1043940	811553	632035	0.9918	0.07348	0.10564	0.551459203
Sarcosine	Amino acids and derivatives	Amino acids and derivatives	C3H7NO2	107-97-1	52304.5	67007	71308	51517.5	51681.4	51420.3	0.84452	0.17229	0.217947	1.232832729
2-Hydroxymyristic acid	Lipids	Free fatty acids	C14H28O3	2507-55-3	25817.5	31822.7	32533.9	48899.3	44718.2	43313.9	1.08637	0.00534	0.015489	0.658534005
UDP	Nucleotides and derivatives	Nucleotides and derivatives	C9H14N2O12P2	27821-45-0	123226	95324	90750.3	56710.3	49985.3	64703.5	1.07865	0.03103	0.051401	1.804560213
Umbelliferone	Lignans and Coumarins	Coumarins	C9H6O3	93-35-6	3.3E+07	4E+07	3.9E+07	7E+07	8.1E+07	8.8E+07	1.13171	0.00637	0.017239	0.467131223
cis,cis-Muconate	Organic acids	Organic acids	C6H6O4	1119-72-8	289328	366670	270936	371986	296006	360471	0.487	0.42192	0.472217	0.901279881
Uridine	Nucleotides and derivatives	Nucleotides and derivatives	C9H12N2O6	58-96-8	104145	107039	125595	104446	97510.5	93730.4	0.80506	0.16734	0.212816	1.138966393
Salicortin	Phenolic acids	Phenolic acids	C20H24O10	29836-41-7	35018.6	37349.3	27299	35941.5	39173.1	48283.5	0.74092	0.17617	0.221784	0.807686354
Piperonylic acid	Phenolic acids	Phenolic acids	C8H6O4	94-53-1	147010	175082	197194	126078	137870	152334	0.84891	0.1266	0.16818	1.247437356
D-Glucuronate*	Others	Saccharides	C6H10O7	528-16-5	165035	130553	209580	228991	213881	263556	0.8857	0.08006	0.113982	0.715103276
Androsin	Others	Ketone	C15H20O8	531-28-2	659769	719370	713470	417117	405742	411772	1.15763	0.00352	0.012142	1.694927573
Scandoside methyl ester	Others	Others	C17H24O11	27530-67-2	354754	466780	322546	458738	625610	445073	0.79626	0.15725	0.201391	0.74804845
10,16-Dihydroxyhexadecanoic acid	Lipids	Free fatty acids	C16H32O4	3233-90-7	47236.3	42432.2	39038.7	40704.3	29705.2	36248.9	0.78202	0.14456	0.187614	1.206724078
3β,6β,19α,24-Tetrahydroxyurs-12-en-28-oic	Terpenoids	Triterpene	C30H48O6	91095-51-1	58393	52564.2	59355.4	468985	437569	438963	1.16368	0.00043	0.003925	0.126577874
NADP+	Nucleotides and derivatives	Nucleotides and derivatives	C21H28N7O17P3	53-59-8	142908	179060	175978	170935	164919	96162.4	0.45278	0.47196	0.520888	1.152606597
Eucalyptin (5-Hydroxy-7,4'-dimethoxy-6,8-dimethylflavone)	Flavonoids	Flavones	C19H18O5	3122-88-1	191474	116787	138154	6678.45	5946.67	8837.06	1.15632	0.02357	0.041814	20.80005825

Poncirin*	Flavonoids	Flavanones	C28H34O14	14941-08-3	91997.4	106739	77691.5	66684.8	66887.7	66102.2	1.00874	0.09261	0.128948	1.384389419
3-Methylbenzaldehyde	Others	Aldehyde compounds	C8H8O	620-23-5	172847	194090	188209	173856	222983	193551	0.39678	0.51087	0.557277	0.940303666
Regaloside G	Phenolic acids	Phenolic acids	C19H26O11	120601-64-1	3347036	3040477	2896023	352805	377593	358620	1.16375	0.0023	0.009585	8.524683217
Dehydrololiolide	Terpenoids	Monoterpenoids	C11H14O3	19355-58-9	33427.5	25370.7	32328.1	44281.1	59747.4	60884.9	1.05958	0.02806	0.047481	0.552570968
4-Feruloyl-5-O-Caffeoylquinic Acid	Phenolic acids	Phenolic acids	C26H26O12	188742-80-5	227752	214938	230583	436355	646624	611379	1.12359	0.03399	0.055355	0.397360935
9,10,11-Trihydroxy-12-octadecenoic	Lipids	Free fatty acids	C18H34O5	61911-67-9	248860	261621	237231	294161	436038	472121	0.975	0.10568	0.144168	0.621890892
Eicosadienoic acid*	Lipids	Free fatty acids	C20H36O2	5598-38-9	494018	467453	410786	178613	179558	186239	1.15664	0.00738	0.019124	2.520630849
3-Oxolup-20(29)-en-28-oic acid (Betulonic	Terpenoids	Triterpene	C30H46O3	4481-62-3	218437	211389	197649	16085.9	14021.2	15894	1.16395	0.00088	0.00581	13.64041537
Salicylic acid-2-O-glucoside	Phenolic acids	Phenolic acids	C13H16O8	10366-91-3	1222057	1306242	1355430	1237225	1105311	1241139	0.74359	0.16791	0.213362	1.083728294
3-Oxo-9,19-cyclolanost-24-en-26-oic acid (Mangiferonic acid)*	Terpenoids	Triterpene	C30H46O3	13878-90-5	212857	224353	189499	17660.5	14251.1	15716	1.16272	0.00261	0.010169	13.15851584
Glehlinoside C	Lignans and Coumarins	Lignans	C26H32O13	402565-04-2	150073	187380	210125	72332.2	55555.5	77441.3	1.11875	0.01382	0.02841	2.666832324
Arbutin	Others	Others	C12H16O7	497-76-7	1124872	1118758	1273517	1448716	1495556	1503806	1.09318	0.01731	0.033346	0.790711521
Sakuranin	Flavonoids	Flavanones	C22H24O10	529-39-5	364082	393601	356521	220521	194009	207277	1.1498	0.00056	0.00459	1.791878837
Glucosyringic acid*	Phenolic acids	Phenolic acids	C15H20O10	33228-65-8	3844490	4011205	4021965	1E+07	8745996	8183430	1.15126	0.01143	0.024946	0.439934321
Pterocarpine	Flavonoids	Isoflavones	C17H14O5	524-97-0	333959	345953	364785	15995.1	19690.9	20956.7	1.16265	0.00055	0.004585	18.44364048
(+)-Pimaric acid*	Terpenoids	Ditepenoids	C20H30O2	127-27-5	271438	237639	181723	499372	376143	231477	0.75884	0.20816	0.25439	0.6240323
(10E,12Z,15Z)-9-oxooctadeca-10,12,15-trienoic acid	Lipids	Free fatty acids	C18H28O3	125559-74-2	94476	79816.8	79825.5	27163.3	27926.6	28049.2	1.15901	0.00712	0.018681	3.056546348
picrorhizin	Phenolic acids	Phenolic acids	C20H28O14	60824-10-4	212387	196789	240186	80643.9	88041.4	74226.4	1.1506	0.00503	0.01495	2.673240527
Methyl cumalate	Others	Lactones	C7H6O4	6018-41-3	142118	159393	156811	203342	235602	232409	1.11329	0.00855	0.020845	0.682683277
N-Acetyl-Tryptophan	Amino acids and derivatives	Amino acids and derivatives	C13H14N2O3	1218-34-4	2202475	2396249	2400572	1638615	1769858	1830759	1.11503	0.00265	0.010249	1.335939147
4'-Hydroxyacetophenone	Others	Ketone compounds	C8H8O2	99-93-4	109860	147621	129178	197287	250762	226629	1.08469	0.0094	0.021906	0.573100624
AICAR phosphate (Acadesine	Nucleotides and derivatives	Nucleotides and derivatives	C9H17N4O9P	681006-28-0	29614.3	29453.2	21314.3	27917.7	23775.5	27398	0.03945	0.89664	0.911301	1.016318436
4-O-Feruloylquinic acid	Phenolic acids	Phenolic acids	C17H20O9	2613-86-7	845690	854524	988680	692053	922056	975929	0.22017	0.75986	0.790791	1.03816765
N-Acetyl-L-glutamate	Amino acids and derivatives	Amino acids and derivatives	C7H11NO5	1188-37-0	49984.9	56084.3	57308.9	24102.2	35650.2	29112.2	1.08431	0.00535	0.015516	1.838506195
Oleanolic acid*	Terpenoids	Triterpene	C30H48O3	508-02-1	3399562	3021130	3250013	1564383	1605320	1610261	1.15897	0.004	0.013043	2.023175413
8-Hydroxykaempferol	Flavonoids	Flavonols	C15H10O7	527-95-7	832748	902194	1081140	490838	554453	575216	1.10456	0.02319	0.041437	1.737778921
Vicianose	Others	Saccharides	C11H20O10	14116-69-9	57165.3	68572.3	115065	42604.6	100346	35107.3	0.49806	0.48532	0.533545	1.352384352
Tracheloside*	Lignans and Coumarins	Lignans	C27H34O12	33464-71-0	202532	240539	247454	230476	230182	327783	0.4863	0.43048	0.480762	0.875811699
Tuberonic acid glucoside*	Organic acids	Organic acids	C18H28O9	120399-24-8	363415	374905	460569	304367	283883	400065	0.71189	0.2123	0.258536	1.213062916
(R)-2-Hydroxyisocaproate	Organic acids	Organic acids	C6H12O3	20312-37-2	724015	671231	616332	97326.9	70893.5	74562.3	1.15898	0.00159	0.007685	8.285510848
Aromadendrin-7-O-glucoside	Flavonoids	Flavanonols	C21H22O11	28189-90-4	131671	140662	160953	34012.7	34352.2	16700	1.11719	0.00074	0.005321	5.093593401
Borneol 7-O-[β-D-apiofuranosyl-(1→6)]-β-D-glucopyranoside*	Terpenoids	Monoterpenoids	C21H36O10	88700-35-0	753428	645950	740848	1856163	2229760	2406915	1.14929	0.00985	0.022547	0.32962879
3,8-Dimethylherbacetin	Flavonoids	Flavonols	C17H14O7	14965-09-4	32099.1	38623.1	36735.5	27373.2	32904	28709.2	0.89535	0.07508	0.107691	1.207574096
Secoisolariciresinol diglucoside	Lignans and Coumarins	Lignans	C32H46O16	158932-33-3	12997.4	16794.5	19241.1	45271.4	52143.8	38922.5	1.1234	0.0073	0.018974	0.359644003
Cantleyine	Alkaloids	Alkaloids	C11H13NO3	30333-81-4	1479796	1707685	1709711	409359	455827	568268	1.14718	0.00059	0.00474	3.416358325
N-Acetyl-L-phenylalanine	Amino acids and derivatives	Amino acids and derivatives	C11H13NO3	2018-61-3	1300868	1133112	1207566	323518	404939	390135	1.15422	0.0006	0.004749	3.255471011
3-Aminosalicylic acid	Phenolic acids	Phenolic acids	C7H7NO3	570-23-0	409089	452769	386895	216997	172004	147980	1.12128	0.00107	0.0064	2.325506956
Isogenistein 7-O-glucoside:5,7,2'-Trihydroxyisoflavone 7-O-	Flavonoids	Isoflavones	C21H20O10	70943-69-0	1571112	1384828	1407839	3394613	3657817	3928503	1.15547	0.00192	0.008581	0.397395975
2,4-Di-Tert-Butylphenol	Phenolic acids	Phenolic acids	C14H22O	96-76-4	4064393	3944812	4555220	4464087	4231738	4354357	0.45885	0.48479	0.533153	0.962777679
Undecanoic acid	Lipids	Free fatty acids	C11H22O2	112-37-8	588252	573170	480053	484720	523047	411194	0.71053	0.19076	0.237152	1.156814422
2-hydroxy-3-methyl-anthraquinone	Quinones	Anthraquinone	C15H10O3	17241-40-6	90077.8	86191.9	90590.3	55162	48508.2	36788.6	1.09312	0.01176	0.02545	1.899916112
13-Hydroperoxy-9Z,11E-octadecadienoic acid*	Lipids	Free fatty acids	C18H32O4	23017-93-8	46628.3	48519.7	40198.7	51931.4	55367.5	55445.5	0.97354	0.05046	0.076561	0.8316524
[(2R)-2-(8-carboxyoctanoyloxy)-3-hexadecanoyloxypropyl] 2-(trimethylazaniumyl)ethyl	Lipids	LPC	C33H64NO10P	117746-89-1	79852	60721.4	66923.8	66091.1	46932	46373.8	0.80863	0.13671	0.179151	1.301764742

Erythro-Guaiacylglycerol-β-O-4'-dehydrodisinapyl Ether	Lignans and Coumarins	Lignans	C31H36O11	613684-55-2	25199.9	24026.6	21525	24683.9	29006.3	34114.8	0.82369	0.1613	0.206155	0.805780581
2,3-Dihydroxyoleana-11,13(18)-dien-28-oic acid (Camaldulenic acid)	Terpenoids	Triterpene	C30H46O4	71850-15-2	465992	481605	469929	163192	191863	235612	1.13235	0.00419	0.013424	2.399874607
Kaempferide*	Flavonoids	Flavonols	C16H12O6	491-54-3	135332	158715	163700	20767.6	41298.5	30194.1	1.12848	0.00064	0.004881	4.961480533
3,4-methylenedioxy cinnamyl alcohol	Others	Alcohol compounds	C10H10O3	17581-86-1	51141.6	39924	42906.8	57204.4	55284.4	56947.1	0.98896	0.06767	0.098338	0.790697091
Carthamone*	Flavonoids	Chalcones	C21H20O11	86579-00-2	302653	301190	227968	53444	78838.2	73429.1	1.13859	0.00868	0.021041	4.043580282
Scopoletin	Lignans and Coumarins	Coumarins	C10H8O4	92-61-5	144906	168355	159338	270599	300284	304743	1.14545	0.00103	0.006291	0.539725379
Deoxyarbutin	Phenolic acids	Phenolic acids	C11H14O3	53936-56-4	13923.3	17604.5	16905.2	13166.9	8789.52	17244.4	0.58709	0.34037	0.390107	1.235507266
Dodecanoic acid	Lipids	Free fatty acids	C12H24O2	143-07-7	1137747	871629	728192	1184016	911164	683507	0.01717	0.94548	0.954417	0.985201906
Methyl sinapate	Phenolic acids	Phenolic acids	C12H14O5	20733-94-2	825616	827815	827194	424530	540636	523020	1.11739	0.0117	0.025375	1.666877518
Homogentisate*	Phenolic acids	Phenolic acids	C8H8O4	451-13-8	2246927	2398559	1972727	146384	181926	163339	1.1623	0.00352	0.012142	13.461271
Anthraquinone-2-carboxylic acid	Quinones	Anthraquinone	C15H8O4	117-78-2	446425	455839	466495	325574	358452	352051	1.13443	0.00187	0.008438	1.321097354
Dambonitol	Others	Saccharides	C8H16O6	523-94-4	1302117	1173301	1425521	769963	759888	581278	1.09983	0.00364	0.012339	1.84779679
9(S)-HPOT	Lipids	Free fatty acids	C18H30O4	111004-08-1	34101.9	39098	35018.6	26678.8	21517.4	28796.5	1.01719	0.02088	0.038397	1.40556934
Pseudopurpurin	Quinones	Quinones	C15H8O7	476-41-5	28070.2	15120.5	15354.6	10988.9	9936.91	8088.28	0.96986	0.14333	0.186258	2.017824514
1-Eicosanol	Lipids	Free fatty acids	C20H42O	629-96-9	18374.2	20375.3	25552.2	18971.3	21409.3	19158.8	0.35939	0.54444	0.591191	1.079985092
Allitol	Others	Saccharides	C6H14O6	488-44-8	53108.5	49117.6	46436.6	19524.4	25303.7	27287.1	1.11681	0.00125	0.006845	2.06145999
Rubiadin-1-Methyl Ether	Quinones	Anthraquinone	C16H12O4	7460-43-7	45533.3	40340.8	56124.1	261665	224709	207171	1.15233	0.00463	0.014164	0.204742786
Eucommin A*	Lignans and Coumarins	Lignans	C27H34O12	99633-12-2	228892	199527	210568	183739	260457	231389	0.24404	0.65083	0.689563	0.945827274
Psoralenoside*	Phenolic acids	Phenolic acids	C17H18O9	905954-17-8	356071	350335	255546	47072.8	125033	150542	0.98541	0.00916	0.021566	2.981432692
2-methoxy-9,10-dihydrophenanthrene-4,5-diol	Quinones	PhenAnthraquinones	C15H14O3	70205-50-4	60757.9	50136.7	63050.4	15278.4	19698.5	19648.1	1.14406	0.00495	0.014771	3.184344589
4-Methylhippuric Acid	Amino acids and derivatives	Amino acids and derivatives	C10H11NO3	27115-50-0	2099528	2250561	2414136	734042	947749	889131	1.1461	0.00043	0.003909	2.631050069
Ferulate*	Phenolic acids	Phenolic acids	C10H10O4	537-98-4	814256	846168	698605	172961	185242	218487	1.15548	0.00314	0.011297	4.090637756
Isoferulic Acid*	Phenolic acids	Phenolic acids	C10H10O4	25522-33-2	1111240	1152712	1246503	215166	293446	286922	1.15297	0.00015	0.002263	4.412697338
Methyl 3-O-glucosyl-4-hydroxybenzoate	Phenolic acids	Phenolic acids	C14H18O9	155112-92-8	34107.6	41814.5	34377.1	17248.8	18862	34476.2	0.85518	0.12239	0.163639	1.562598656
Pseudouridine	Nucleotides and derivatives	Nucleotides and derivatives	C9H12N2O6	1445-07-4	21665	27526.2	28768.3	8391.73	15519.6	15274.3	0.99103	0.01579	0.031449	1.989490817
ASPERULOSIDIC ACID METHYL ESTER	Terpenoids	Monoterpenoids	C19H26O12	14260-99-2	407006	359984	424568	476342	645985	595192	1.01028	0.05861	0.086839	0.693767026
(2R,3S)-3-Isopropylmalate*	Organic acids	Organic acids	C7H12O5	921-28-8	151808	158821	191211	42315.9	47423.6	42288.9	1.15591	0.00854	0.020844	3.801000689
L-threo-3-Methylaspartate	Amino acids and derivatives	Amino acids and derivatives	C5H9NO4	6061-13-8	167686	173237	159672	206329	331396	271129	0.99787	0.10281	0.140866	0.618893511
Cryptochlorogenic acid (4-O-Caffeoylquinic acid)*	Phenolic acids	Phenolic acids	C16H18O9	905-99-7	3.5E+07	3.4E+07	3.8E+07	5.2E+07	6.3E+07	5.7E+07	1.12667	0.0161	0.031848	0.620922183
D-Glucarate	Others	Saccharides	C6H10O8	87-73-0	854358	700332	868044	440706	442610	548193	1.08931	0.00999	0.022746	1.692432488
myo-Inositol 4-phosphate	Others	Saccharides	C6H13O9P	142760-33-6	1333226	1374118	1169719	1791955	1883401	1660679	1.08685	0.00567	0.016112	0.726581151
cis-Vaccenic	Lipids	Free fatty acids	C18H34O2	506-17-2	910163	981529	839754	372519	435343	410250	1.15045	0.00216	0.009199	2.242360257
Levopimaric	Terpenoids	Ditepenoids	C20H30O2	79-54-9	55869.7	59600.2	30832.3	149837	96761.8	68361.5	0.90005	0.13016	0.172243	0.464509802
ADP	Nucleotides and derivatives	Nucleotides and derivatives	C10H15N5O10P2	58-64-0	151024	79864.3	149541	86342.7	90231.8	92766.8	0.68046	0.25498	0.302541	1.412444766
Xanthurenic acid	Alkaloids	Quinoline alkaloids	C10H7NO4	59-00-7	66130.1	74600.4	56186.8	51680.4	56853.7	32103.6	0.78949	0.11963	0.160573	1.400174216
Vanillic acid glucoside	Phenolic acids	Phenolic acids	C14H18O9	32142-31-7	6073045	4635317	5143974	1814489	1782145	2225753	1.13986	0.00942	0.021919	2.72265244
2,2-Dimethylsuccinic acid	Organic acids	Organic acids	C6H10O4	597-43-3	13643.5	9475.51	6202.98	22919.7	17971.6	11594	0.81419	0.13145	0.173591	0.558669789
9,10,13-TriHOME	Lipids	Free fatty acids	C18H34O5	29907-57-1	339560	332356	300388	170530	207980	183440	1.12969	0.00114	0.006581	1.730232491
Fraxin	Lignans and Coumarins	Coumarins	C16H18O10	524-30-1	1987424	1927599	1762292	720086	650542	789787	1.15431	0.00041	0.003813	2.627881112
Traumatic acid	Lipids	Free fatty acids	C12H20O4	6402-36-4	138896	137926	151389	71198.2	70082.9	88618.1	1.12722	0.00133	0.007025	1.862603662
7-Deoxyloganate	Terpenoids	Monoterpenoids	C16H24O9	22487-36-1	3093860	3463223	3845572	3744257	3049171	2804023	0.42661	0.49483	0.542262	1.083897751
8-Epideoxyloganic	Terpenoids	Monoterpenoids	C16H24O9	88668-99-9	3485275	3472915	4472203	3546198	3599006	4082434	0.07529	0.8676	0.88745	1.018058559
2-Oxoglutarate	Organic acids	Organic acids	C5H6O5	328-50-7	1078755	1066294	1484486	333884	422006	312200	1.1337	0.02002	0.037253	3.398155358
Hesperetin-5-O-glucoside	Flavonoids	Flavanones	C22H24O11	69651-80-5	3387595	2600139	2329712	1817584	1907981	1902012	1.00359	0.10453	0.142717	1.477979923
Cannabiscitrin	Flavonoids	Flavonols	C21H20O13	520-14-9	365984	399416	367125	138441	146203	172386	1.15055	0.00012	0.002074	2.47800869
Isoquercitrin	Flavonoids	Flavonols	C21H20O12	482-35-9	3383847	4296515	4011714	1862809	2426489	2486682	1.07108	0.00999	0.022746	1.72551804
Phthalate	Phenolic acids	Phenolic acids	C8H6O4	88-99-3	645118	702530	677410	147255	159468	175042	1.16118	0.00013	0.00213	4.203413976
2,5-Dihydroxybenzoic acid; Gentisic Acid*	Phenolic acids	Phenolic acids	C7H6O4	490-79-9	176040	138098	168236	187628	238501	218995	0.95316	0.04793	0.073178	0.747722639
Propyl 4-hydroxybenzoate	Phenolic acids	Phenolic acids	C10H12O3	94-13-3	25713.1	18658.2	13702.9	19933.4	35094.6	17171	0.38781	0.52034	0.566817	0.804362815
2-Hydroxybutyric	Organic acids	Organic acids	C4H8O3	3347-90-8	370316	331691	356583	72967.6	102740	83791.6	1.15208	7.7E-05	0.001628	4.079359447
3-Carboxy-4-Methyl-5-Propyl-2-Furanpropionic Acid	Organic acids	Organic acids	C12H16O5	86879-39-2	107896	104963	72037.2	70624.9	95285.9	91533.6	0.32804	0.54906	0.595381	1.106633405
Pinoresinol-4,4'-O-diglucoside	Lignans and Coumarins	Lignans	C32H42O16	63902-38-5	144359	156713	171749	93234.8	118433	115630	1.04401	0.01249	0.026384	1.44461674
Glyceraldehyde 3-phosphate	Organic acids	Organic acids	C3H7O6P	591-59-3	262964	264281	328319	181712	260390	250201	0.72983	0.17366	0.219411	1.235823873
Rubiadin*	Quinones	Anthraquinone	C15H10O4	117-02-2	611596	593408	574448	498418	457619	416894	1.07534	0.0161	0.031848	1.296097004

ampelopsin E	Others	Stilbene	C42H32O9	149496-36-6	536835	523054	644550	487208	659236	525153	0.11807	0.87471	0.893165	1.019647137
Medicagenic acid-3-O-glucuronide-28-O-xylosyl(1,4)-rhamnosyl(1,2)-arabinoside	Terpenoids	Triterpene Saponin	C52H80O24	128192-15-4	120506	91164.4	127711	195735	161506	156161	0.99917	0.0259	0.044875	0.661043444
Isocitrate*	Organic acids	Organic acids	C6H8O7	320-77-4	7.6E+07	7.4E+07	8.2E+07	4.5E+07	5.9E+07	4.7E+07	1.07508	0.01082	0.023878	1.527537153
Emodin	Quinones	Anthraquinone	C15H12O4	491-60-1	908135	905284	924371	456570	450768	467763	1.16395	7.2E-07	0.000249	1.99097284
Chrysophanol	Quinones	Anthraquinone	C15H10O4	481-74-3	2.1E+07	2.2E+07	2.2E+07	5.4E+07	5.5E+07	5.8E+07	1.16323	0.0006	0.004749	0.387525039
alpha-Isopropylmalate	Organic acids	Organic acids	C7H12O5	49601-06-1	214995	168050	176743	43405.8	54912.6	51071	1.15117	0.00819	0.020343	3.74717856
Phenyl acetate	Others	Others	C8H8O2	122-79-2	150464	151675	162262	242295	220395	191491	1.07129	0.04285	0.066985	0.709896214
2,4-	Others	Others	C6H4N2O5	51-28-5	16641.8	23542.9	16736.9	19258.3	18136.8	19380.2	0.05264	0.98503	0.987939	1.002577877
Soyasapogenol B	Terpenoids	Triterpene	C30H50O3	595-15-3	33589.7	32878.4	33179.1	31237.4	32088.4	34550.9	0.34097	0.61547	0.656769	1.018091224
(1alpha,3R,4alpha,5R)-3,4,5-Trihydroxy-1-[[3-(4-hydroxy-3-methoxyphenyl)-1-oxo-2-propen-1-yl]oxy]cyclohexa	Phenolic acids	Phenolic acids	C17H20O9	53505-93-4	4880027	5415301	4427192	5071209	5735627	5573142	0.71985	0.19639	0.242596	0.898811975
D-Arabinonate*	Others	Saccharides	C5H10O6	32609-14-6	974657	1144473	1074558	657563	924691	722829	0.96673	0.04503	0.069711	1.385498374
(9Z,11E)-(13S)-13-Hydroperoxyoctadeca-9,11-dienoic acid	Lipids	Free fatty acids	C18H32O4	33964-75-9	1.9E+07	1.8E+07	1.9E+07	5408054	5072779	5257790	1.16413	4.5E-05	0.001296	3.561675456
Damnacanthol	Quinones	Anthraquinone	C16H12O5	477-83-8	5882289	6322591	6575645	1.5E+07	1.9E+07	1.8E+07	1.1543	0.00729	0.018974	0.357152403
Tribuloside	Flavonoids	Flavonols	C30H26O13	20316-62-5	73508.9	109983	77281.7	50257.5	59172.8	81118.5	0.7479	0.19191	0.238293	1.368541815
D-Glucose-1-phosphate*	Others	Saccharides	C6H13O9P	59-56-3	2522629	2905633	3194806	3383648	3246254	3336379	0.85369	0.14306	0.185984	0.8652243
3-Deoxy-D-glycero-D-galacto-non-2-ulopyranosonate	Lipids	Free fatty acids	C9H16O9	153666-19-4	207101	175443	173054	186298	180151	182037	0.09876	0.85003	0.872427	1.012968257
3-Ureidopropionate	Amino acids and derivatives	Amino acids and derivatives	C4H8N2O3	462-88-4	9482.25	13809.3	4832.29	6182.19	6849.88	3684.05	0.63217	0.27772	0.325536	1.682437343
1-(9Z,12Z-octadecadienoyl)-sn-glycero-3-phosphocholine	Lipids	LPC	C26H50NO7P	22252-07-9	7236177	6967591	6792157	4709409	4993778	4952944	1.15305	0.00036	0.003654	1.432569539
D-Sorbitol	Others	Saccharides	C6H14O6	50-70-4	37266.1	25937.1	40549.8	12113.5	19009.6	34730.8	0.72283	0.20119	0.247637	1.57550337
Gentisate aldehyde	Others	Aldehyde compounds	C7H6O3	1194-98-5	83361.9	118036	95204.2	198232	195193	216961	1.11854	0.00184	0.008438	0.485925142
D-Fructose*	Others	Saccharides	C6H12O6	57-48-7	1775825	1578379	1621390	1194763	1533206	1500543	0.80617	0.13298	0.175157	1.176677152
L-Glucose*	Others	Saccharides	C6H12O6	921-60-8	1680930	1811806	1935923	1404665	1164004	1410174	1.04925	0.01184	0.025547	1.364380879
Syringaldehyde; 4-Hydroxy-3,5-Dimethoxybenzaldehyde	Others	Aldehyde compounds	C9H10O4	134-96-3	50683.5	57921.2	68746.4	94693.9	65847	67384.2	0.746	0.21043	0.256861	0.778111539
Morolic acid*	Terpenoids	Triterpene	C30H48O3	559-68-2	3358171	3168961	3078954	1629535	1540387	1562052	1.16076	0.00111	0.006549	2.030037707
Chrysophanol-1-O-β-D-	Quinones	Anthraquinone	C21H20O9	4839-60-5	1485726	1285953	1306470	3156643	3493670	3296964	1.15601	0.0002	0.00263	0.409976415
Soyasapogenol	Terpenoids	Triterpene	C30H48O3	6750-59-0	3225638	3038653	3041600	1564082	1502889	1594368	1.16167	0.00033	0.003509	1.996398601
2-Carboxy-3-hydroxy-A(1)-norlupan-20(29)-en-28-oic acid (Ceanothic acid)	Terpenoids	Triterpene	C30H46O5	21302-79-4	18052	16269.4	12917.8	3826.09	5688.37	5234.5	1.12565	0.01099	0.024154	3.202884008
Ricinoleic acid	Lipids	Free fatty acids	C18H34O3	141-22-0	29892.6	32402.3	28714	34633.6	35159.5	32620.6	0.94879	0.0524	0.079035	0.888640323
Suberic acid	Lipids	Free fatty acids	C8H14O4	505-48-6	38739.8	48339.9	25038.7	29306.4	31413.8	35901.3	0.32486	0.52965	0.575939	1.160386907
Rubiatriol	Terpenoids	Triterpene	C30H50O3	107694-02-0	182663	164576	152412	78813.2	79080.3	68128.4	1.14649	0.00391	0.01286	2.21063376
Lawsone	Quinones	Quinones	C10H6O3	83-72-7	8218604	8065043	7263204	614056	1095055	1230952	1.14079	0.00013	0.00213	8.008960287
Didymin (Isosakuranetin-7-O-rutinoside)*	Flavonoids	Flavanones	C28H34O14	14259-47-3	297294	346867	346188	179160	254441	254938	0.96014	0.03598	0.057971	1.438335336
13(S)-HOT	Lipids	Free fatty acids	C18H30O3	87984-82-5	397214	411727	404122	249059	247241	268128	1.15637	0.00015	0.00229	1.586891674
Tridecanedioic acid	Lipids	Free fatty acids	C13H24O4	505-52-2	910832	966337	908886	1045143	1005683	956358	0.88431	0.08759	0.123179	0.926466446
1,4-Dihydroxy-2-hydroxymethyl anthraquinone	Quinones	Anthraquinone	C15H10O5	22296-59-9	1878762	1714918	1722292	310806	299641	344547	1.16285	0.00074	0.005321	5.566494859
Betulinic acid*	Terpenoids	Triterpene	C30H48O3	472-15-1	2934495	2729775	2803547	1385626	1360645	1473764	1.16006	0.00017	0.00244	2.006575435
Sedoheptulose	Others	Saccharides	C7H14O7	3019-74-7	402737	448862	483406	217248	276053	282337	1.09397	0.00414	0.013284	1.721170469
Glycerone phosphate	Others	Ketone compounds	C3H7O6P	57-04-5	299767	226609	223748	194778	608110	205093	0.23355	0.59429	0.638162	0.74418436
L-Aspartic Acid*	Amino acids and derivatives	Amino acids and derivatives	C4H7NO4	56-84-8	127717	182642	214560	402717	553723	333037	1.05392	0.04517	0.069824	0.407079315
L-Fucitol	Others	Saccharides	C6H14O5	13074-06-1	299786	266823	252229	161693	190238	213169	1.03962	0.0146	0.029617	1.449014826
Chrysophanol 8-O-beta-D-glucoside*	Quinones	Anthraquinone	C21H20O9	13241-28-6	1848303	1807723	1931727	4912198	5205451	5211502	1.16316	0.00025	0.002941	0.364518127
alpha-Hydroxylinoleic acid*	Lipids	Free fatty acids	C18H32O3	57818-44-7	803172	781963	821187	282061	282788	296374	1.16372	8E-05	0.001654	2.79407343
3-[(1-Carboxyvinyl)oxy]benzoate	Phenolic acids	Phenolic acids	C10H8O5	16929-37-6	2738185	2777432	2813921	9758137	1.2E+07	1.3E+07	1.15744	0.01038	0.023261	0.244072227
Alizarin*	Quinones	Anthraquinone	C14H8O4	72-48-0	1240906	1194855	1261617	1032562	1019692	1009016	1.14523	0.00448	0.013898	1.207792306
Rutinose	Others	Saccharides	C12H22O10	90-74-4	91114.3	44054.7	96702.5	38868.9	36892	54861.3	0.79981	0.17155	0.217273	1.775129689
Neosakuranin	Flavonoids	Chalcones	C22H24O10	31187-54-9	229172	168710	225869	54138.2	71490.5	59164.2	1.13906	0.01304	0.027255	3.375407432
Plantaginin	Flavonoids	Flavones	C21H20O11	26046-94-6	1098929	1033399	1180700	280700	236063	281865	1.15921	0.00098	0.006139	4.148396526
2-Methylglutaric acid*	Organic acids	Organic acids	C6H10O4	617-62-9	216954	296354	240247	168651	173838	88664.4	0.90827	0.04268	0.066879	1.7477623
alpha,alpha-Trehalose*	Others	Saccharides	C12H22O11	99-20-7	2367497	1585214	2370493	1301940	803973	769881	1.02634	0.02742	0.046707	2.198767922
Sucrose*	Others	Saccharides	C12H22O11	57-50-1	2004649	2411159	2880664	1106582	1209799	1374561	1.09403	0.03246	0.053439	1.976858775
L-Gulono-1,4-Lactone*	Others	Saccharides	C6H10O6	1128-23-0	72978.9	62414.9	54101.6	41323.3	65901.7	45875.6	0.65743	0.26914	0.316556	1.237718148
1,6-anhydro-β-D-glucose	Others	Saccharides	C6H10O5	498-07-7	70984.2	61933.6	84409.5	47745.9	38404.9	34461.1	1.06385	0.02016	0.037469	1.80187183

4-ketopinoresinol	Lignans and Coumarins	Lignans	C20H20O7	66288-89-9	27691.9	36572.7	38552.6	32431.7	34846.5	38706.1	0.19285	0.79901	0.825662	0.970117941
2,19-Dihydroxy-3-oxours-12-en-28-oic acid	Terpenoids	Triterpene	C30H46O5	176983-21-4	231273	205494	256483	186036	193956	195871	0.94144	0.11175	0.151374	1.203842358
Shikimate	Organic acids	Organic acids	C7H10O5	138-59-0	61993	111775	66656.8	84143.2	55192.8	46940	0.51428	0.4117	0.461785	1.290692267
Trifolirhizin*	Flavonoids	Isoflavones	C22H22O10	6807-83-6	1230744	1161081	1155444	141057	125273	103841	1.16105	6.7E-05	0.001561	9.58275557
Isopimaric acid*	Terpenoids	Ditepenoids	C20H30O2	5835-26-7	67211.7	65917	37710.6	150242	119304	86032.5	0.96503	0.06	0.088557	0.480453629
Formononetin	Flavonoids	Isoflavones	C16H12O4	485-72-3	638972	645102	626491	571827	572692	580217	1.14447	0.00235	0.009685	1.107744031
L-Xylose*	Others	Saccharides	C5H10O5	25990-60-7	163261	94822.1	114401	55012.8	57069.7	84081.2	0.97349	0.08427	0.11943	1.898838958
Hexadecanedioat	Lipids	Free fatty acids	C16H30O4	505-54-4	58555.7	59343	64864.4	73204.1	71553.3	72608.9	1.09128	0.02315	0.041409	0.840807081
FMN	Nucleotides and derivatives	Nucleotides and derivatives	C17H21N4O9P	6184-17-4	1175371	1232491	972970	612482	616119	647607	1.12947	0.02198	0.039838	1.801948644
2-Aldehydo-A(1)-norlup-20(29)-en-27,28-dioic acid	Terpenoids	Triterpene	C30H44O5	1167421-91-1	457468	485613	501231	337678	329701	234794	1.04188	0.02071	0.038237	1.600927037
Genipin	Terpenoids	Monoterpenoids	C11H14O5	6902-77-8	352953	372724	362202	228792	300184	252386	1.04905	0.03239	0.053375	1.392286414
Resveratrol-4'-O-β-D-(6''-O-galloyl)-	Others	Stilbene	C27H26O12	64898-03-9	400269	363682	320014	142003	288467	222365	0.91048	0.05637	0.083919	1.660395086
12,13-DHOME	Lipids	Free fatty acids	C18H34O4	263399-35-5	43715.4	33723	42660.1	45423.9	43458.4	39777.3	0.43642	0.48288	0.531241	0.933459526
Octadecanoic	Lipids	Free fatty acids	C18H36O2	57-11-4	3.7E+07	3.7E+07	3.5E+07	3.7E+07	4.3E+07	3.9E+07	0.72464	0.2431	0.29078	0.923109905
D-Fructose 6-phosphate*	Others	Saccharides	C6H13O9P	643-13-0	2004837	1469980	2090952	1607358	2047278	2555185	0.33286	0.56088	0.606495	0.896284889
Allantoin	Alkaloids	Alkaloids	C4H6N4O3	97-59-6	109510	73535.5	61707.8	105883	145096	113859	0.8522	0.10132	0.139128	0.670854608
alpha,alpha'-Trehalose 6-phosphate	Others	Saccharides	C12H23O14P	4484-88-2	44146.7	28617.1	31593.2	29823.2	33044.3	29666.9	0.41795	0.49729	0.544196	1.127763758
D-Xylonate*	Others	Saccharides	C5H10O6	526-91-0	1754085	1696767	1256061	946463	1184154	1367875	0.81873	0.11741	0.157946	1.345411862
3-Methylmalic acid*	Organic acids	Organic acids	C5H8O5	152204-30-3	1014188	1025928	1091213	613665	654552	615132	1.15572	0.00049	0.004274	1.662638389
Adipic Acid*	Organic acids	Organic acids	C6H10O4	124-04-9	153144	153964	163550	128554	136798	111447	1.0111	0.03617	0.058235	1.249093828
3,23-Dihydroxyolean-12-en-28-oic acid (Hederagenin)	Terpenoids	Triterpene	C30H48O4	465-99-6	13113.7	16284.4	13473.5	3918.43	6710.01	6259.3	1.08762	0.00304	0.011151	2.538619489
Citrate	Organic acids	Organic acids	C6H8O7	77-92-9	2.6E+07	3.2E+07	3.3E+07	3.3E+07	2.9E+07	2.7E+07	0.04127	0.93453	0.944895	1.00785655
D-Pinitol*	Others	Saccharides	C7H14O6	10284-63-6	135525	221808	122788	174860	183990	221663	0.5673	0.40483	0.454577	0.827062595
Aloesaponarin	Quinones	Anthraquinone	C15H10O4	53254-94-7	671930	606512	604685	547378	452069	383996	0.96358	0.05402	0.080882	1.36118832
D-Gluconic acid	Others	Saccharides	C6H12O7	526-95-4	1195196	1327865	1346738	884776	709362	944533	1.06539	0.00919	0.0216	1.524340586
Gingerglycolipid B	Lipids	Glycerol ester	C33H58O14	88168-90-5	206179	188803	200792	113929	111468	142976	1.10168	0.00709	0.018619	1.617311104
Norarjunolic acid	Terpenoids	Triterpene	C29H44O5	60393-90-0	907413	882022	973693	258890	231563	271590	1.16044	0.00036	0.003654	3.625944017
Fumarate	Organic acids	Organic acids	C4H4O4	110-17-8	2186276	2122433	2299058	1093680	1058392	1188723	1.15611	0.00012	0.002091	1.977902436
Protocatechuic acid-4-O-glucoside*	Phenolic acids	Phenolic acids	C13H16O9	7361-59-3	1.9E+07	2E+07	2.1E+07	5172765	4712876	5781699	1.15953	3.8E-05	0.001212	3.802345549
(7Z)-Hexadecenoic	Lipids	Free fatty acids	C16H30O2	2416-19-5	5550.1	5934.52	6164.43	10666.9	8176.68	7441.84	1.0083	0.09371	0.130072	0.671437217
3-Phospho-D-glycerate	Organic acids	Organic acids	C3H7O7P	820-11-1	422362	208564	227536	170612	139043	142553	0.92058	0.18365	0.229876	1.898383236
Quinate	Organic acids	Organic acids	C7H12O6	77-95-2	507996	326155	241321	389455	136776	257772	0.50493	0.41666	0.46659	1.371769945
5-Carboxymellein	Lignans and Coumarins	Coumarins	C11H10O5	69135-42-8	65262.4	63573.7	47217.5	302013	259052	282579	1.15243	0.00071	0.005185	0.208682215
2,3-Dihydroxyolean-12-en-28-oic acid (2-Hydroxyoleanolic acid)*	Terpenoids	Triterpene	C30H48O4	26707-60-8	2303655	2198342	2086284	4474573	4644911	4553607	1.16045	1.3E-05	0.000842	0.481842881
3,19-Dihydroxyurs-12-en-28-oic acid (Pomolic acid)*	Terpenoids	Triterpene	C30H48O4	13849-91-7	3.7E+07	3.5E+07	3.6E+07	1.6E+07	1.6E+07	1.6E+07	1.1635	0.00121	0.00675	2.24841725
Citpressine I	Alkaloids	Quinoline alkaloids	C16H15NO5	81525-58-8	15534.5	12871.1	11500.3	5673.79	7325.88	7085.45	1.10104	0.01812	0.034516	1.986836446
Methyl caffeate	Phenolic acids	Phenolic acids	C10H10O4	3843-74-1	22804.2	18418.1	19234.6	34136.6	53030.1	48274.4	1.09045	0.04156	0.065423	0.446370604
mudanoside B*	Phenolic acids	Phenolic acids	C18H24O14	203511-37-9	97985	64937.3	63629.8	19994.3	18448.8	18493.8	1.14058	0.03709	0.0595	3.979009183
10-Hydroxy-2-Decenoic Acid	Lipids	Free fatty acids	C10H18O3	14113-05-4	30063.2	23306.2	18546.6	50478	52082.6	47853.2	1.08954	0.00902	0.021308	0.478121248
Pentadecanoic acid	Lipids	Free fatty acids	C15H30O2	1002-84-2	68601.3	59515.9	64685.3	122408	101007	93822.6	1.09465	0.03134	0.05184	0.607753289
L-Ascorbic acid-2-glucoside	Others	Vitamin	C12H18O11	129499-78-1	148567	167977	126875	90964.6	70837	99677.9	1.04681	0.01741	0.033506	1.695805935
3-Hydroxy-3-methylglutarate	Organic acids	Organic acids	C6H10O5	503-49-1	3515444	1750225	3155251	881012	1164566	1091896	1.04632	0.07871	0.11232	2.683980474
Coniferyl alcohol	Others	Alcohol compounds	C10H12O3	458-35-5	41176.5	36845.2	29503.2	12867.1	26090.2	33235.7	0.71047	0.17985	0.225757	1.489408099
13-methylmyristic	Lipids	Free fatty acids	C15H30O2	2485-71-4	32472.8	39446.5	38564.5	78755.7	80064.2	75558.3	1.14905	0.0003	0.003296	0.471391603
Solatriose	Others	Saccharides	C18H32O15	528-40-5	57076.4	21581.4	24560	16597.3	15466.7	22000.3	0.76447	0.28475	0.332886	1.909165993
6-Methylaloe emodin	Quinones	Anthraquinone	C16H12O5	873431-39-1	414176	338036	336786	187055	171852	187891	1.13913	0.01635	0.032126	1.991587436
Formylanthranilate	Phenolic acids	Phenolic acids	C8H7NO3	3342-77-6	156468	154332	177949	159116	169143	168328	0.2077	0.77295	0.801943	0.98421471
3,23-Dihydroxy-30-noroleana-12,20(29)-dien-28-oic acid (30-Norhederagenin)	Terpenoids	Triterpene	C29H44O4	117654-06-5	459301	434533	467519	100807	113840	98037.4	1.16186	8.7E-05	0.001732	4.353768922
Indolelactate	Alkaloids	Plumerane	C11H11NO3	1821-52-9	50868.8	40055.8	38650.3	20064.8	12306.9	12052.9	1.10243	0.00552	0.015822	2.916741087
Hexadecanal	Lipids	Free fatty acids	C16H32O	629-80-1	1.3E+07	1.3E+07	1.4E+07	1.3E+07	1.2E+07	1.1E+07	0.79346	0.14141	0.184375	1.086901743
p-Coumaroyl-D-glucose	Phenolic acids	Phenolic acids	C15H18O8	7139-64-2	78930.9	76346	54620.1	37117.4	58237.6	43847.5	0.88809	0.07904	0.112736	1.50785356
Lucidin	Quinones	Anthraquinone	C15H10O5	478-08-0	2E+07	2E+07	2.1E+07	1.8E+07	1.9E+07	1.8E+07	1.07298	0.00879	0.021201	1.123296054
(R)-2,3-Dihydroxy-3-methylpentanoate	Organic acids	Organic acids	C6H12O4	562-43-6	140005	104685	109218	119730	97879.8	77250.4	0.60819	0.30041	0.34909	1.200258374
D-Galactarate	Others	Saccharides	C6H10O8	526-99-8	667311	695823	408599	370367	481694	305674	0.80899	0.14152	0.18445	1.530343895
D-Galacturonate*	Others	Saccharides	C6H10O7	685-73-4	198951	198595	198882	211369	218014	188933	0.42675	0.49418	0.541931	0.964601555
Coniferin	Others	Alcohol compounds	C16H22O8	531-29-3	380507	344528	413067	244579	236980	187812	1.09263	0.00435	0.013682	1.700254746

Eriodictyol	Flavonoids	Flavanones	C15H12O6	552-58-9	58896.1	68837	65218	15596.7	21916.1	16820.6	1.14651	0.00042	0.00388	3.55123847
13(S)-HODE*	Lipids	Free fatty acids	C18H32O3	10219-69-9	603497	606663	582425	177183	199084	169016	1.16057	5E-06	0.0006	3.287437025
Kaur-16-en-18-oic acid*	Terpenoids	Ditepenoids	C20H30O2	6730-83-2	243112	219975	173296	415426	318424	209010	0.73402	0.22274	0.269236	0.674950792
9(S)-HODE*	Lipids	Free fatty acids	C18H32O3	15514-85-9	800679	782599	799918	297599	266453	301504	1.1613	2.9E-05	0.00117	2.753369931
(9S)-Hydroxyoctadecadienoic acid	Lipids	Free fatty acids	C18H32O3	73543-67-6	3630996	3454938	3290255	1208593	1252042	1365699	1.16004	0.00036	0.003654	2.711783548
Cirsilineol	Flavonoids	Flavones	C18H16O7	41365-32-6	13108.3	8723.78	10709	4549.47	7732.41	8360.38	0.84719	0.08415	0.119306	1.5764287
1-(4-Hydroxy-3-methoxyphenyl)-2-(2-methoxyphenoxy)propane-1,3-diol	Lignans and Coumarins	Lignans	C17H20O6	7382-59-4	102349	89598.3	95130.9	38652	50005.4	40672.6	1.13778	0.0005	0.004359	2.219734677
1-O-Salicyloyl-β-D-glucose*	Phenolic acids	Phenolic acids	C13H16O8	60517-74-0	8269173	6601812	6975635	75979.8	99643.9	129625	1.16125	0.00488	0.014664	71.56982647
Cmp-nana	Nucleotides and derivatives	Nucleotides and derivatives	C20H31N4O16P	3063-71-6	1585769	1551032	1439941	1325073	1611685	1404342	0.457	0.47378	0.52235	1.054281454
5,7,2'-Trihydroxy-8-methoxyflavone*	Flavonoids	Flavones	C16H12O6	80713-32-2	1437515	1611138	1583112	843443	1198900	1072276	1.02376	0.0229	0.041077	1.487104288
D-Glucose*	Others	Saccharides	C6H12O6	50-99-7	1832900	1480920	1796254	1510234	1526388	1515715	0.73678	0.23785	0.285159	1.122516631
D-Mannose*	Others	Saccharides	C6H12O6	3458-28-4	2139081	2039333	2279659	1873550	1406072	1777077	0.93329	0.06272	0.092082	1.277132166
D-Arabinose*	Others	Saccharides	C5H10O5	10323-20-3	125594	116759	128584	97344.4	70840	90296.4	1.02443	0.02687	0.046009	1.435062981
alpha-D-Galactosyl-(1->3)-1D-myo-	Others	Saccharides	C12H22O11	3687-64-7	5031780	5008661	3970101	2045861	3091136	2289179	1.06112	0.00987	0.022567	1.886642497
2(3H)-Benzothiazolone	Alkaloids	Alkaloids	C7H5NOS	934-34-9	149971	108464	65208.5	96996.6	61338.8	42128.1	0.67246	0.24395	0.291573	1.614476665
Geniposidic acid	Terpenoids	Monoterpenoids	C16H22O10	27741-01-1	329731	345913	322028	2.9E+07	3.1E+07	3.2E+07	1.16461	0.0009	0.005901	0.010880974
1,2,4-Trihydroxynaphthalene-1,4-di-glucoside (Lawsoniaside)	Others	Others	C22H28O13	116964-02-4	42812.2	57057.5	52556.3	67088.9	59435.8	57829.3	0.82828	0.11376	0.153559	0.826811296
6'-O-Feruloyl-D-sucrose*	Phenolic acids	Phenolic acids	C22H30O14	118230-77-6	150968	155865	164251	73521	81373.1	90804.9	1.14159	0.0004	0.003813	1.917318851
Sibiricose A5*	Phenolic acids	Phenolic acids	C22H30O14	107912-97-0	173794	237686	206688	71942.1	102065	123745	1.04548	0.01196	0.025689	2.076116348
4-	Phenolic acids	Phenolic acids	C7H7NO3	65-49-6	135346	121990	119860	46487.8	44800.3	35462.3	1.14858	0.00028	0.003175	2.97588901
(S)-2-Hydroxyglutarate	Organic acids	Organic acids	C5H8O5	13095-48-2	1160439	934085	1055405	561781	607862	634789	1.1274	0.01372	0.028346	1.745662263
Terephthalate	Phenolic acids	Phenolic acids	C8H6O4	100-21-0	5818686	5375502	5500323	1598527	2059599	2001906	1.15118	5.1E-05	0.001368	2.949543729
Salicylaldehyde	Others	Aldehyde compounds	C7H6O2	90-02-8	348203	232945	310318	51559.6	97287	110377	1.07562	0.01155	0.025155	3.438983119
gentiananoside D	Terpenoids	Monoterpenoids	C18H26O11	2454150-75-3	2748315	2170501	2489173	637630	797311	1199922	1.09225	0.00254	0.01004	2.811526762
Vanillate	Phenolic acids	Phenolic acids	C8H8O4	121-34-6	371801	216889	315233	200046	166220	140262	0.968	0.08498	0.120098	1.784546211
2-Oxopimelate*	Organic acids	Organic acids	C7H10O5	17126-90-8	136958	152061	113806	130296	154418	136807	0.28433	0.66654	0.704282	0.95564633
Chlorogenic acid methyl ester	Phenolic acids	Phenolic acids	C17H20O9	29708-87-0	4190302	4030914	2984143	4441598	4242156	5246641	0.77498	0.13873	0.181272	0.804382027
4-O-(4'-O-alpha-D-Glucopyranosyl)caffeoylquinic	Phenolic acids	Phenolic acids	C22H28O14	1401352-62-2	4608351	3703894	3911082	7582820	8006295	7517481	1.1385	0.00111	0.006549	0.528997313
methyl 4-O-galloylchlorogenate	Phenolic acids	Phenolic acids	C24H24O13	574747-58-3	1061806	1029716	1128433	701689	909096	763204	1.03146	0.02813	0.047567	1.35634768
Dihydroconiferyl alcohol	Phenolic acids	Phenolic acids	C10H14O3	2305-13-7	46587.2	43003.8	55537.7	65646.8	72857.1	68669.8	1.06305	0.01508	0.030292	0.700516658
4-Hydroxy-2,5-dimethylfuran-3(2H)-one	Others	Ketone compounds	C6H8O3	3658-77-3	38913.1	36144.1	50233.2	69168.4	55436.3	67840.7	1.01564	0.02186	0.039691	0.651043674
1-O-Feruloyl-β-D-glucose	Phenolic acids	Phenolic acids	C16H20O9	7196-71-6	1.5E+07	1.3E+07	1.6E+07	1.9E+07	2E+07	2.3E+07	1.07848	0.01653	0.032323	0.702402818
Bergenin	Lignans and Coumarins	Coumarins	C14H16O9	477-90-7	725256	598047	678854	390882	384534	337713	1.13032	0.0069	0.018237	1.79867448
(S)-Mandelate	Phenolic acids	Phenolic acids	C8H8O3	90-64-2	576573	354917	806893	170132	289991	392891	0.84063	0.13808	0.180803	2.037930252
12-Hydroxyjasmonic Acid Glucoside	Organic acids	Organic acids	C18H28O9	124649-25-8	165513	174444	209950	145226	203933	173167	0.25654	0.6948	0.729975	1.052802947
2-Methylsuccinic acid*	Organic acids	Organic acids	C5H8O4	498-21-5	2059537	1857455	1910579	1837625	1666715	2008213	0.49223	0.42497	0.475459	1.057145459
Oxalate	Organic acids	Organic acids	C2H2O4	144-62-7	411014	466767	411804	56756.3	73075.7	57643.5	1.15937	0.0013	0.006996	6.878685289
Chrysoeriol-7-O-glucoside*	Flavonoids	Flavones	C22H22O11	19993-32-9	2064327	2213555	2188268	751485	1106538	890913	1.12591	0.00233	0.009634	2.352237356
Obtusifolin 2-glucoside	Quinones	Anthraquinone	C22H22O10	120163-18-0	1932053	2072874	1960132	314105	394036	324989	1.16052	3.5E-05	0.001173	5.773772237
Methyl 14-methylpentadecanoate	Lipids	Free fatty acids	C17H34O2	5129-60-2	875494	896744	904956	787753	898839	899146	0.43567	0.50021	0.547192	1.035369455
4-Hydroxybenzoate	Phenolic acids	Phenolic acids	C7H6O3	99-96-7	464233	445009	556022	764916	928814	608676	0.98539	0.08028	0.11424	0.636405701
Phloretate	Phenolic acids	Phenolic acids	C9H10O3	501-97-3	33431.3	35954.3	29021.8	21017.4	19922.5	24100.7	1.07882	0.01462	0.029624	1.513014301
(S)-2-Acetolactate*	Organic acids	Organic acids	C5H8O4	71698-08-3	151733	142792	153237	95731.9	121256	134623	0.89936	0.09716	0.134375	1.273459109
Pratensein 7-O-glucopyranoside*	Flavonoids	Isoflavones	C22H22O11	36191-03-4	2047457	2217283	2404146	591405	922955	812317	1.12589	0.00053	0.004495	2.866270008
Grevilloside F	Phenolic acids	Phenolic acids	C15H18O9	1094004-50-8	2108368	2100180	2504878	1104682	1383492	1295573	1.11209	0.00612	0.016856	1.774280229
Diosmetin*	Flavonoids	Flavones	C16H12O6	520-34-3	1508497	1608872	1572407	877890	1056781	1129755	1.09507	0.01031	0.023202	1.530393301
UMP	Nucleotides and derivatives	Nucleotides and derivatives	C9H13N2O9P	58-97-9	177872	155622	222491	60615.3	49836.7	61857.5	1.13863	0.01969	0.0368	3.226666498
Dimethylmalonic acid*	Organic acids	Organic acids	C5H8O4	595-46-0	248826	271051	242215	183821	158878	170060	1.12424	0.00209	0.009068	1.486256806
5,3'-Dihydroxy-7,4'-Dimethoxyflavon	Flavonoids	Flavones	C17H14O6	32174-62-2	10398.4	12306.8	9690.86	42039.1	47978.2	40483.2	1.1557	0.00225	0.00942	0.248244101
Syringin	Others	Alcohol compounds	C17H24O9	118-34-3	2236567	2155816	2170045	1404117	1300400	1203322	1.14716	0.00131	0.007005	1.679298306
Chicoric acid	Phenolic acids	Phenolic acids	C22H18O12	70831-56-0	21964.8	19232.9	29937.6	22516.3	32265.2	38943.3	0.63448	0.2696	0.316899	0.758981289
Chrysoeriol*	Flavonoids	Flavones	C16H12O6	491-71-4	122998	148808	117300	29103.8	23402	30235.1	1.15384	0.00679	0.01809	4.702701406
1,3-Dihydroxyanthraquinone (Xanthopurpurin)	Quinones	Anthraquinone	C14H8O4	518-83-2	415277	412370	400688	71067.6	94625.6	70034.6	1.15648	3.4E-05	0.001173	5.210820171

2-Deoxy-D-ribose 1-	Nucleotides and derivatives	Nucleotides and derivatives	C5H11O7P	17210-42-3	34464.5	72276.6	127153	32691.8	53359.4	36402.3	0.63766	0.2998	0.348511	1.910063205
3-Hydroxyglutaric	Organic acids	Organic acids	C5H8O5	638-18-6	302637	222730	272777	167055	123825	166825	1.05042	0.02081	0.038309	1.743797293
6-Oxo-7(9)-dehydro-6,7-dihydrogeranyl acetate	Terpenoids	Monoterpenoids	C12H18O3	101622-74-6	58617.3	81368.9	79569.7	55909	70662.2	78277.7	0.26356	0.64412	0.683449	1.071794652
Caffeic acid 4-O-glucoside	Phenolic acids	Phenolic acids	C15H18O9	166735-99-5	32617	33833.5	34517	27143.1	26502.8	39024.9	0.43531	0.56811	0.613247	1.089526832
Lariciresinol 4-O-glucoside	Lignans and Coumarins	Lignans	C26H34O11	143663-00-7	1462968	1516943	1567270	2802664	2773937	3031602	1.15822	0.00139	0.007185	0.528238157
Anhydroglycinol	Flavonoids	Isoflavones	C15H10O4	67685-22-7	573984	509635	656501	180775	168086	191152	1.15323	0.00973	0.022449	3.22236119
Isoxanthopterin	Nucleotides and derivatives	Nucleotides and derivatives	C6H5N5O2	529-69-1	11854.4	9619.79	12642.3	7149.64	10685.2	7862.85	0.82026	0.11933	0.160239	1.327609346
6-	Flavonoids	Flavones	C15H10O7	18003-33-3	1190137	1082493	1338087	559821	678767	697486	1.11619	0.00593	0.016531	1.864967967
Norathyriol	Flavonoids	Flavones	C13H8O6	3542-72-1	74321.6	70433.8	90598.8	180643	182504	197611	1.14538	0.00021	0.002662	0.419706868
1,5-Anhydro-D-glucitol	Others	Saccharides	C6H12O5	154-58-5	36196	66033.3	78614.9	34821.8	39655.2	46924.6	0.67075	0.25211	0.299709	1.489635914
6'-O-α-D-galactosylbarlerin	Terpenoids	Monoterpenoids	C25H38O17	927691-89-2	140294	110701	77519.5	114264	135356	83215.9	0.05564	0.95439	0.961841	0.987016046
3,4-Dihydroxybenzoate*	Phenolic acids	Phenolic acids	C7H6O4	99-50-3	270966	387676	213781	355769	375459	578802	0.77307	0.17905	0.224843	0.665957004
Gardoside	Terpenoids	Monoterpenoids	C16H22O10	54835-76-6	52671.1	16543.1	22428.7	1752703	1735218	1545988	1.14802	0.00124	0.006845	0.018205118
Isofraxidin-7-O-glucoside	Lignans and Coumarins	Coumarins	C17H20O10	483-91-0	58320.3	77887.5	63826.3	37327.6	46519	90868.4	0.39537	0.66921	0.706619	1.14491628
D-Lactose*	Others	Saccharides	C12H22O11	63-42-3	4916538	4865718	5413988	3216738	1883547	4969661	0.76044	0.19229	0.238573	1.509069021
Melibiose	Others	Saccharides	C12H22O11	585-99-9	1932326	1656751	1312953	735502	1093440	854313	1.02813	0.03352	0.054821	1.826896753
Isopsoralenoside	Phenolic acids	Phenolic acids	C17H18O9	905954-18-9	307892	258400	270664	39632.3	115797	130202	0.98093	0.00993	0.022634	2.930188402
mudanpioside E	Terpenoids	Monoterpenoids	C24H30O13	172705-25-8	88048.1	99084.1	96708	70518.9	62706.8	95068.6	0.77934	0.1899	0.236359	1.243308517
3-O-	Phenolic acids	Phenolic acids	C8H8O5	3934-84-7	21633.7	16774.2	24404.8	5151.2	11258.5	18635.4	0.77801	0.12614	0.16765	1.792340814
1-(4-Methoxyphenyl)-1-propanol	Others	Alcohol compounds	C10H14O2	5349-60-0	30284	31242.1	22433	27448.9	24934.8	22236.1	0.48476	0.39699	0.447875	1.12515967
Oxamate	Amino acids and derivatives	Amino acids and derivatives	C2H3NO3	471-47-6	19395.8	44431.1	39434.7	19812.3	23777	9558.12	0.77051	0.14849	0.191916	1.942925786
Aminomalonate	Amino acids and derivatives	Amino acids and derivatives	C3H5NO4	1068-84-4	129787	527169	392704	109406	106916	149624	0.8354	0.18851	0.234826	2.868346186
D-Cellobiose	Others	Saccharides	C12H22O11	528-50-7	56742.9	56250.5	45852.5	37766.1	46578.8	30096	0.88776	0.07244	0.104405	1.388017201
4-[4-(4-hydroxy-3-methoxyphenyl)-2,3-dimethylbutyl]-2-methoxyphenol	Lignans and Coumarins	Lignans	C20H26O4	36469-60-0	323176	272221	282645	10460.7	12120.3	16380.3	1.1601	0.00278	0.010534	22.53624962
2-(1,3-Benzodioxol-5-yl)-5-hydroxy-6,8-dimethoxy-4H-1-	Flavonoids	Flavones	C18H14O7	220841-90-7	73955.6	75115.7	74134.3	2768.52	2703.01	4161.25	1.16028	8.1E-08	0.000172	23.17147592
Maltitol	Others	Saccharides	C12H24O11	585-88-6	408114	431791	372641	251184	309408	304380	1.05535	0.01041	0.023285	1.401834035
Danthron	Quinones	Anthraquinone	C14H8O4	117-10-2	417340	307428	301124	56879.4	33536.2	47153	1.14589	0.01366	0.028229	7.45730775
Phenyllactate	Phenolic acids	Phenolic acids	C9H10O3	7326-19-4	51053.5	55890.9	63620.4	5968.49	10702.6	10885.5	1.13615	0.00191	0.008549	6.189631197
4-Hydroxyphenylethanol	Others	Alcohol compounds	C8H10O2	501-94-0	11644.1	16548.1	13297.3	49437.5	36119.7	32595.2	1.11233	0.0304	0.050637	0.351152501
2,3-Dihydroxyurs-12-en-28-oic acid	Terpenoids	Triterpene	C30H48O4	4547-24-4	2598948	2647091	2468669	4253370	4772613	5054443	1.14564	0.00924	0.021679	0.547903018
FAD	Nucleotides and derivatives	Nucleotides and derivatives	C27H33N9O15P2	146-14-5	1258923	1217354	1251363	700227	962077	942726	1.0156	0.04434	0.068889	1.430939583
Diacetoxy-[6]-gingerdiol	Others	Alcohol compounds	C21H32O6	143615-75-2	5880.69	7826.09	6399.52	4320.66	1896.59	2735.95	1.00426	0.01673	0.032561	2.24571103
Pyrocatechol monoglucoside	Others	Others	C12H16O7	2400-71-7	27485.6	13259.3	26083.2	16799.5	14360.2	11259.8	0.72607	0.20694	0.253487	1.575409331
Erythorbic acid	Others	Vitamin	C6H8O6	89-65-6	87728.1	77765.1	100904	30917.8	47396.5	30324.5	1.09537	0.00423	0.013472	2.452135833
trans-3-Hydroxycinnamate*	Phenolic acids	Phenolic acids	C9H8O3	14755-02-3	818295	632980	622057	1299784	1858235	2042958	1.09549	0.03479	0.056305	0.398642758
3,19,23-Trihydroxyurs-12-en-28-oic acid (Rutundic acid)	Terpenoids	Triterpene	C30H48O5	20137-37-5	27042	20278.6	28895.9	33691.5	33363.6	39502.9	0.95047	0.04073	0.064324	0.71525769
9-(Arabinosyl)hypoxanthine	Nucleotides and derivatives	Nucleotides and derivatives	C10H12N4O5	7013-16-3	22016.4	14984.2	16653.6	40430.6	43998	35750.4	1.10954	0.00238	0.009789	0.446452216
3-hydroxy-5-methoxybenzaldehyde	Phenolic acids	Phenolic acids	C8H8O3	57179-35-8	28809.5	27035.9	41560	20462.6	9812.82	10561.9	1.00164	0.03356	0.054861	2.38520507
Xanthorin	Quinones	Anthraquinone	C16H12O6	17526-15-7	469271	536604	551769	31294.9	38562.7	72542.6	1.13985	0.00052	0.004495	10.93849928
3,4-Dimethoxyphenyl acetic acid	Phenolic acids	Phenolic acids	C10H12O4	93-40-3	65448	37711.2	44241.4	19087.5	16051.3	10779.4	1.08071	0.04691	0.072081	3.210069533
Nectandrin B	Lignans and Coumarins	Lignans	C20H24O5	74683-16-2	12834.7	9507.65	10905.7	1320.89	1320.89	1320.89	1.16105	0.00962	0.022311	8.390335047
Erythro-Guaiacylglycerol-β-threo-syringylglycerol Ether	Lignans and Coumarins	Lignans	C21H28O10	1313434-74-0	7809.5	7458.02	7875.35	20869.4	13178.2	21680.2	1.08954	0.05663	0.084189	0.415283981
Methylmalonate*	Organic acids	Organic acids	C4H6O4	516-05-2	4562464	1.7E+07	1.7E+07	7454679	8281530	8882751	0.36417	0.37802	0.428351	1.570207123
9-OxoODE*	Lipids	Free fatty acids	C18H30O3	54232-59-6	21496.7	20622.5	20089.2	5083.24	3838.91	4167.17	1.1581	8.5E-06	0.000711	4.752603822
9(S)-HPODE	Lipids	Free fatty acids	C18H32O4	5502-91-0	3561412	3798750	3903041	1215169	1135535	1172102	1.16271	0.00095	0.006057	3.197225186
Salicin	Phenolic acids	Phenolic acids	C13H18O7	138-52-3	14409.9	23872	19041.5	9192.94	9197.66	13812.8	0.95649	0.07173	0.103506	1.780041684
N-Acetylisatin	Alkaloids	Plumerane	C10H7NO3	574-17-4	29975.8	24079	19469.1	2849.52	2849.52	2849.52	1.15736	0.01914	0.035997	8.600749227
Cyrtominetin	Flavonoids	Flavones	C17H16O6	95272-99-4	23620.9	15763.6	22773.8	5003.08	11023.6	5384.12	1.0383	0.01431	0.029156	2.903131159
4-O-galactopyranosyl xylose	Others	Saccharides	C11H20O10	14087-31-1	82748.8	94787.4	122529	77457.8	78852	66778.2	0.86927	0.15164	0.195536	1.345053646
(2R,3S)-Dihydrodehydroconiferyl alcohol	Lignans and Coumarins	Lignans	C20H24O6	126253-41-6	16670.1	10240.5	13300.5	6984.82	13701.2	16051.3	0.25422	0.74487	0.777235	1.094558807

Pimelate	Lipids	Free fatty acids	C7H12O4	111-16-0	15626.1	13551.9	13904.5	10320.7	17788.6	9217.32	0.47228	0.55338	0.599635	1.154202785
Protocatechuic Acid Methyl	Phenolic acids	Phenolic acids	C8H8O4	2150-43-8	2290.34	2290.34	2290.34	26342.8	24686.6	24973.9	1.16457	0.00049	0.004299	0.090404058
3-Oxours-12-en-28-oic acid (Ursonic acid)*	Terpenoids	Triterpene	C30H46O3	6246-46-4	184270	195950	186078	16280.1	16280.1	16280.1	1.16461	0.00044	0.004009	11.59490351
2,3,23-Trihydroxyolean-12-ene-28,29-dioic acid 29-methyl ester (Phytolaccagenin	Terpenoids	Triterpene	C31H48O7	1802-12-6	33985.6	31552.1	33030.5	6286.63	6286.63	6286.63	1.1644	0.00071	0.005169	5.226346651
2-Phosphoglycolate	Organic acids	Organic acids	C2H5O6P	13147-57-4	47845.5	73590.4	41126	30857.1	57023.9	36614.7	0.54817	0.37623	0.426633	1.305763187
Salicin 6'-Acetate	Phenolic acids	Phenolic acids	C15H20O8	19764-02-4	21516	22614.6	19089.3	6445.8	12169.6	10612.5	1.04301	0.00835	0.020626	2.163007989
Isovitexin	Flavonoids	Flavones	C21H20O10	38953-85-4	17486.9	24336.4	24992.5	16394.4	5025.95	7796.7	0.91979	0.04604	0.070989	2.286876706
2-Propylsuccinic acid	Organic acids	Organic acids	C7H12O4	618-57-5	13698.6	14836.6	18899.4	19032	16088.8	10780	0.14817	0.86925	0.888468	1.033413597
Citrusin C; Eugenol Glucoside	Phenolic acids	Phenolic acids	C16H22O7	18604-50-7	4081.08	4021.39	7824.59	20783.3	44388.8	31296.3	1.10263	0.05472	0.081679	0.165101394
Aspidinol	Others	Others	C12H16O4	519-40-4	4846.43	8250.86	10134.1	4758.64	5818.53	1614.06	0.73004	0.14203	0.18503	1.905584748
2-Hydroxy-4-methylpentanoic acid	Organic acids	Organic acids	C6H12O3	498-36-2	79602.7	65754	49564.7	13078.7	17898.9	16054.4	1.13452	0.02706	0.046234	4.1444395
3-Hydroxycycloarta-24-ene-26-oic acid (Isomangiferolic	Terpenoids	Triterpene	C30H48O3	13878-92-7	694878	648167	687473	85258.6	87570.2	95916.3	1.16379	0.00035	0.003637	7.555553156
UDP-N-acetyl-alpha-D-glucosamine	Nucleotides and derivatives	Nucleotides and derivatives	C17H27N3O17P2	528-04-1	11862.2	11516.4	10364.7	5764.86	4689.14	3126.33	1.08457	0.00364	0.012349	2.484725516
helminthosporin	Quinones	Anthraquinone	C15H10O5	518-80-9	252526	193005	268360	95997.2	127606	95121	1.09986	0.01592	0.03158	2.239842009
Syringic acid	Phenolic acids	Phenolic acids	C9H10O5	530-57-4	102620	78091.1	106400	87139.4	132243	157332	0.61907	0.28251	0.3304	0.762142889
D-Glycerate	Organic acids	Organic acids	C3H6O4	473-81-4	42326.9	51477.1	43060.2	8465.37	8465.37	8465.37	1.16151	0.00618	0.016979	5.389174332
2,3-Dihydroxybenzoate*	Phenolic acids	Phenolic acids	C7H6O4	303-38-8	307153	361728	302856	389780	602659	528872	0.97801	0.08845	0.124158	0.638749494
13S-Hydroperoxy-6Z,9Z,11E-octadecatrienoic	Lipids	Free fatty acids	C18H30O4	121107-97-9	22431.2	19580.7	20800.5	9567.24	7920.98	14530.9	1.03392	0.02266	0.04079	1.96171745
N-Acetyl-L-Glutamine	Amino acids and derivatives	Amino acids and derivatives	C7H12N2O4	2490-97-3	71630.3	57539	77819.1	30923.4	31254.3	40808.5	1.0937	0.01385	0.028446	2.009865234
Sennoside C	Quinones	Anthraquinone	C42H40O19	37271-16-2	334867	334165	341603	2571.42	3213.91	4386.73	1.16213	2.3E-05	0.001051	99.35401863
Wedelolactone	Lignans and Coumarins	Coumarins	C16H10O7	524-12-9	18822.1	15630.6	12929.9	22370.8	31082.5	31200.2	1.02702	0.03108	0.051432	0.559723354
3-O-Methylquercetin*	Flavonoids	Flavonols	C16H12O7	1486-70-0	33414.7	16832.3	21798.9	12219.6	11235.5	6356.78	0.97706	0.08938	0.125126	2.416681714
Quercetin-3-O-robinobioside	Flavonoids	Flavonols	C27H30O16	52525-35-6	1658.43	1658.43	1658.43	11297.9	11778.2	8292.13	1.15641	0.01503	0.030251	0.158609021
Phenylpyruvate	Phenolic acids	Phenolic acids	C9H8O3	156-06-9	123961	82328.5	133061	43155	31704	35912.7	1.111	0.03465	0.056139	3.06351026
1-Hydroxypinoresinol-1-O-	Lignans and Coumarins	Lignans	C26H32O12	81495-71-8	100387	132101	133281	251381	254130	222033	1.11794	0.00127	0.006901	0.502744772
(R)-Mevalonate	Organic acids	Organic acids	C6H12O4	150-97-0	52997.9	102966	70006.9	30781.5	38324.2	54615.5	0.88256	0.13112	0.173278	1.826449604
Citreorosein	Quinones	Anthraquinone	C15H10O6	481-73-2	2965.54	2965.54	2965.54	19418.1	15661.9	14827.7	1.15936	0.0105	0.023395	0.178261575
1,2,3,19-Tetrahydroxyurs-12-en-28-oic acid	Terpenoids	Triterpene	C30H48O6	120211-98-5	1607.69	1607.69	1607.69	37076	35955.7	43275.3	1.16397	0.00373	0.012535	0.041468535
2-Hydroxy-3-oxopropanoate	Organic acids	Organic acids	C3H4O4	2480-77-5	72939.3	48034.9	51679.7	4926.88	4926.88	4926.88	1.15864	0.02109	0.038637	11.68107523
Picein	Phenolic acids	Phenolic acids	C14H18O7	530-14-3	3592.05	3592.05	3592.05	17960.2	23138.7	20534.8	1.16079	0.00769	0.01968	0.1748416
5,4'-Dihydroxy-3,7-dimethoxyflavone(Kumatakenin)*	Flavonoids	Flavonols	C17H14O6	3301-49-3	19550.6	20659.4	16710.3	2040.3	2040.3	2040.3	1.16302	0.00479	0.014506	9.29933502
Azaleatin	Flavonoids	Flavonols	C16H12O7	529-51-1	16076	22488.6	21792.6	8634.51	5528.93	14067	0.95112	0.03097	0.051369	2.138019135
Azukisaponin VI	Terpenoids	Triterpene	C54H86O25	82801-39-6	473252	535113	598039	49165.7	49165.7	49165.7	1.16267	0.00544	0.015685	10.89109702
6-Ethoxygeniposid	Terpenoids	Monoterpenoids	C19H28O11	1264496-61-8	7587.23	7587.23	7587.23	79448.7	77423.5	71649.9	1.16434	0.00116	0.006626	0.099603908
Hellicoside	Phenolic acids	Phenolic acids	C29H36O17	132278-04-7	15442.4	4515.12	14769.4	13542.3	13712.2	22864.3	0.55222	0.33638	0.385683	0.692892174
Indole-5-carboxylic acid*	Alkaloids	Plumerane	C9H7NO2	1670-81-1	16287.7	27141.6	17878	1683.61	1683.61	1683.61	1.15537	0.03106	0.051429	12.13804884
2-Hydroxyethylphosphonate	Organic acids	Organic acids	C2H7O4P	22987-21-9	18577	22907.4	13288.2	24404.2	23654.5	13640.1	0.25323	0.63227	0.672189	0.887739288
Epicatechin 3-glucoside	Flavonoids	Flavanols	C21H24O11	103303-00-0	46135.5	53171.1	61548.4	5643.55	5643.55	5643.55	1.16128	0.00851	0.020799	9.500823102
(Indol-3-yl)acetyl-L-	Alkaloids	Plumerane	C14H14N2O5	2456-73-7	109411	119449	118613	15187.9	15187.9	15187.9	1.16422	0.00102	0.00629	7.626119558
Narirutin	Flavonoids	Flavanones	C27H32O14	14259-46-2	45511.9	56074.8	73902.1	2273	2273	2273	1.16004	0.02103	0.03855	25.73526308
Gambiriin A1	Tannins	Tannin	C30H28O12	76250-49-2	8482381	8038125	9880393	472657	472657	472657	1.1636	0.00441	0.013769	18.61877974
Procyanidin A1	Tannins	Proanthocyanidin	C30H24O12	103883-03-0	25625.5	31525.9	18861.9	1137.27	1137.27	1137.27	1.15955	0.0221	0.040003	22.27953005
3-Oxoolean-12-en-28-oic Acid (Oleanonic	Terpenoids	Triterpene	C30H46O3	17990-42-0	170729	182634	166661	13190.1	13190.1	13190.1	1.16451	0.00089	0.005882	13.14180014
Ascorbate	Others	Vitamin	C6H8O6	50-81-7	34908.2	40215.5	29587.4	9115.09	27474	24975.5	0.76251	0.11283	0.152571	1.700832369
3-Hydroxymandelate	Phenolic acids	Phenolic acids	C8H8O4	17119-15-2	1381.95	1381.95	1381.95	16405	18941.7	16729.1	1.16408	0.00248	0.009973	0.079611576
Tuberonic Acid	Organic acids	Organic acids	C12H18O4	124649-26-9	2425.88	2425.88	2425.88	21876.4	18970.4	35807.3	1.14883	0.04696	0.072126	0.094941427
Robustaside A;[6'-p-Coumarylarbutin	Phenolic acids	Phenolic acids	C21H22O9	148810-39-3	33155.1	31085.6	21954.1	2375.9	2375.9	2375.9	1.15895	0.01662	0.03249	12.09292525
2,3-Dihydro-1,4-naphthoquinone	Quinones	Quinones	C10H8O2	21545-31-3	27512.2	20851.8	23371.7	2076.13	2076.13	2076.13	1.16238	0.00781	0.019825	11.51757957
Sideretin	Lignans and Coumarins	Coumarins	C10H8O6	2177290-23-0	616469	654407	557937	78106.6	103311	99536.7	1.15831	0.00161	0.007765	6.509293683
2-Hydroxyphenylacetate	Phenolic acids	Phenolic acids	C8H8O3	614-75-5	29334.7	33835.7	33656	2445.44	2445.44	2445.44	1.16387	0.00242	0.009859	13.19821989

Tricalysioside O	Terpenoids	Ditepenoids	C28H46O12	874332-47-5	1976305	1615125	1747830	194448	194448	194448	1.16324	0.0044	0.013748	9.152839944
Indole-3-carboxylate*	Alkaloids	Plumerane	C9H7NO2	771-50-6	23188.9	20413	38310	6779.69	7122.44	10815.7	1.07679	0.06874	0.099701	3.313873453
3-Hydroxyurs-12-en-28-oic acid (Ursolic acid)*	Terpenoids	Triterpene	C30H48O3	77-52-1	697517	648601	673981	92468.3	86263	71427.2	1.1614	7.8E-05	0.001638	8.075279857
Agrimonolide	Lignans and Coumarins	Coumarins	C18H18O5	21499-24-1	101045	89965.5	96354.6	9813.77	9813.77	9813.77	1.1643	0.00139	0.007186	9.760619095
D-Mannitol*	Others	Saccharides	C6H14O6	69-65-8	12785.8	15184.5	12234.8	8565.92	7538.4	4378.17	0.99475	0.01631	0.032058	1.962898041
Pedalitin*	Flavonoids	Flavones	C16H12O7	22384-63-0	19981	29001.8	31164.5	7298.19	14210.8	4537.84	1.00183	0.01665	0.032507	3.077046216
Lucidin 1,3-dimethyl ether	Quinones	Anthraquinone	C17H14O5	61434-48-8	46779.5	56630.3	57356.8	6386.49	8647.22	6345.18	1.15605	0.00384	0.012753	7.519875395
4-Hydroxyquinolin	Alkaloids	Quinoline alkaloids	C9H7NO	611-36-9	30397.7	24358.9	24470.3	2336.59	2336.59	2336.59	1.16284	0.0068	0.018097	11.30236396
Velutin (5,4'-dihydroxy-7,3'-dimethoxyflavone)*	Flavonoids	Flavones	C17H14O6	25739-41-7	23801.1	26841.4	26581.7	3241.37	3241.37	3241.37	1.16383	0.00187	0.008438	7.941504121
Norartocarpetin	Flavonoids	Flavones	C15H10O6	520-30-9	101019	73206.5	80612.9	98632.2	106642	121734	0.86829	0.09107	0.127087	0.779302218
Choerospondin	Flavonoids	Flavanones	C21H22O10	81202-36-0	17847.4	9670	16303.3	94431.6	75258.8	100575	1.13537	0.00574	0.016201	0.162139616
Verbascoside	Phenolic acids	Phenolic acids	C29H36O15	61276-17-3	48413.8	30098.6	23704	65306.8	84386.6	63116.5	0.99954	0.02155	0.039298	0.480317655
4-Hydroxy-2-oxopentanoate*	Organic acids	Organic acids	C5H8O4	3318-73-8	91490.9	93889.6	96630.5	64391.3	81670.6	87005	0.84912	0.13363	0.175784	1.210000151
Kaempferol-3,7-O-diglucoside	Flavonoids	Flavonols	C27H30O16	25615-14-9	7801.2	14713.8	24369.2	7627.45	3624.35	10718.2	0.73422	0.2198	0.266304	2.134012719
Olivil-4'-O-glucoside	Lignans and Coumarins	Lignans	C26H34O12	76880-93-8	45051.4	56839.5	82200.6	34563.3	18297.7	33266.2	0.95168	0.07827	0.11185	2.137435583
Deacetyl asperuloside	Terpenoids	Monoterpenoids	C16H20O10	18843-01-1	9467.36	9467.36	9467.36	47336.8	99478.3	103184	1.13288	0.05473	0.081679	0.113608765
3,3'-Di-O-methylellagic acid 4'-glucoside	Tannins	Tannin	C22H20O13	51803-68-0	14686.4	5604.51	10065.1	12944.1	4283.85	6858.8	0.34176	0.59951	0.64333	1.260273338
Lactate	Organic acids	Organic acids	C3H6O3	113-21-3	40298.2	32003.8	26736.2	3233.98	3233.98	3233.98	1.15904	0.01712	0.033069	10.20807122
Hibiscus acid	Organic acids	Organic acids	C6H8O8	6205-14-7	10236.2	5793.16	17766.3	1158.63	1158.63	1158.63	1.11572	0.10165	0.13946	9.72288608
3,5-Digalloylshikimic acid	Phenolic acids	Phenolic acids	C23H26O11	95753-52-9	23483.1	27609.5	38577.7	927.445	927.445	927.445	1.16028	0.02334	0.041563	32.22842283
Sanguiin H7	Tannins	Tannin	C34H26O23	98917-86-3	7926.58	6483.24	8868.72	9569.39	10283.4	7037.19	0.49755	0.38011	0.43025	0.865696773
3-nitro-L-tyrosine	Amino acids and derivatives	Amino acids and derivatives	C9H10N2O5	621-44-3	64405.1	64862.9	64836.3	4820.1	4820.1	4820.1	1.16475	6.1E-06	0.000656	13.42325193
Benzoylmalic	Phenolic acids	Phenolic acids	C11H10O6	22138-51-8	20429	17523.2	13362.4	2016.52	2016.52	2016.52	1.15732	0.01798	0.034326	8.48235824
3'-AMP	Nucleotides and derivatives	Nucleotides and derivatives	C10H14N5O7P	84-21-9	7574.65	8147.56	11646.6	1466.42	1466.42	1466.42	1.15186	0.02653	0.045641	6.221245299
Alizarin 1-methyl ether	Quinones	Anthraquinone	C15H10O4	6170-06-5	154127	144149	139231	44953.1	53018	46596.2	1.15842	0.00022	0.002725	3.026318998
Curcurbitacin IIA	Terpenoids	Triterpene	C32H50O8	58546-34-2	9885.88	10779.4	9105.98	1186.73	1186.73	1186.73	1.16361	0.00305	0.011153	8.362237016
Glyceraldehyde	Others	Aldehyde compounds	C3H6O3	56-82-6	23857.2	24366	35122.9	2598.4	2598.4	2598.4	1.15782	0.02062	0.038118	10.69198035
Rhamnose	Others	Saccharides	C6H12O5	3615-41-6	51262	29087.2	22783.9	21557.8	27534	29324.1	0.46509	0.44353	0.492668	1.315206623
2,4,6-Tri-O-galloyl-D-	Phenolic acids	Phenolic acids	C27H24O18	108043-99-8	11235.9	7206.61	14291.3	1441.32	1441.32	1441.32	1.14143	0.04387	0.068192	7.570323504
Hydroxytyrosol	Others	Alcohol compounds	C8H10O3	10597-60-1	2297.7	2297.7	2297.7	22737.2	26582.7	15123	1.15164	0.02946	0.049301	0.106964236
Piceid	Others	Stilbene	C20H22O8	27208-80-6	20014.3	18556	15463.2	2287.99	2287.99	2287.99	1.16182	0.0072	0.018826	7.872072324
Acacetin-7-O-glucoside (Tilianin)*	Flavonoids	Flavones	C22H22O10	4291-60-5	1971334	2161210	2219203	124048	417588	372701	1.08949	0.00013	0.00213	6.94683271
12-Hydroxydodecanoic acid	Lipids	Free fatty acids	C12H24O3	505-95-3	1402.42	1402.42	1402.42	11694.2	13062.5	10560.7	1.1629	0.00483	0.014557	0.119126607
6-O-Feruloyl-glucose	Phenolic acids	Phenolic acids	C16H20O9	137887-25-3	1232331	1248866	1221268	629447	721689	722726	1.15014	0.00194	0.008635	1.785298303
2-Benzylsuccinic Acid	Organic acids	Organic acids	C11H12O4	884-33-3	38436.5	35593.7	37147.2	3830.44	3830.44	3830.44	1.16457	0.00061	0.004781	9.674910708
2-Methylmaleate	Organic acids	Organic acids	C5H6O4	498-23-7	419225	446640	497862	204799	110500	305740	0.95799	0.03384	0.05517	2.195881963
7-hydroxy-3-(2-hydroxy-4-methoxyphenyl)chromen-2-one	Lignans and Coumarins	Coumarins	C16H12O5	54300-95-7	127463	102071	111776	14878.6	14878.6	14878.6	1.16256	0.00555	0.015868	7.646550901

Table S10. Hormone profiling data of *OpAVT1-KO1* hairy root line.

Compounds	Class	KO-1	KO-2	KO-3	EV-1	EV-2	EV-3	P-value	FDR	Fold_Change
N6-isopentenyladenosine	CK	2.18707343	2.10093215	2.07903742	3.83017402	4.17474593	3.92020464	0.001436667	0.00431	0.533918363
Tryptamine	Auxin	1701.95003	1648.4617	1581.36132	5951.31552	6318.80081	5849.31786	0.000582779	0.002507794	0.272181405
L-tryptophan	Auxin	4647.11558	6387.60697	6947.59356	2235.06319	2714.60874	3211.20953	0.028486127	0.030161782	2.203477185
Indole-3-acetyl glutamic acid	Auxin	208.152803	166.136155	144.202271	0.879650922	0.917568598	0.710247404	0.011690394	0.014028473	206.7788907
trans-Zeatin-9-glucoside	CK	281.36299	256.008048	264.618771	1062.36534	1086.18648	1047.09326	2.69614E-06	9.70609E-05	0.250963354
Gibberellin A29	GA	309.252488	249.618225	237.486992	326.916304	324.123476	298.457544	0.134806272	0.134806272	0.83871506
Gibberellin A20	GA	60.6789559	55.5702425	42.9377627	80.6018231	102.145325	84.1165241	0.015037566	0.016917262	0.59651042
1-Aminocyclopropanecarboxylic acid	ETH	43.4366748	48.7894764	47.1217731	38.2092915	34.5355691	35.3250356	0.007734811	0.011602216	1.28942406
2-Methylthio-cis-zeatin riboside	CK	0.643169815	0.730839955	0.74698319	0.547462192	0.378531504	0.434934331	0.017788617	0.019405764	1.558490176
trans-Zeatin riboside	CK	14.3825157	13.9240271	13.4855663	320.98612	327.868394	334.203828	0.000138708	0.002000713	0.042512339
N6-Isopentenyl-adenine-9-glucoside	CK	19.5769856	19.8408975	18.3351511	4.12150404	4.09697663	4.21487477	0.000886616	0.003191818	4.645007897
N6-Isopentenyl-adenine-7-glucoside	CK	48.7248629	42.6520477	42.5222634	8.28778227	7.23968496	7.59244553	0.002556732	0.005113463	5.791508618
para-Topolin	CK	2.2773055	2.46981714	2.00942065	42.772167	47.0368394	41.1789859	0.00168716	0.00457958	0.051581394
trans-Zeatin-O-glucoside	CK	170.367154	180.82773	146.508155	1114.02527	1241.6565	1293.48402	0.001908158	0.00457958	0.136388169
9-Ribosyl-trans-zeatin 5'-monophosphate	CK	26.5188909	32.9923085	32.1157695	284.162005	288.211382	307.529016	0.000330376	0.002000713	0.104133105
Dihydrozeatin-7-glucoside	CK	2.56253809	2.50440098	2.9431659	15.2980889	18.0949187	18.0159336	0.003405556	0.006130002	0.155811514
Methyl indole-3-acetate	Auxin	2.58551696	2.19975295	2.38131129	0.51036358	0.375754573	0.392924048	0.001138195	0.003725002	5.603084241
Indole-3-acetyl-L-aspartic acid	Auxin	16734.6892	15837.8667	16117.0702	18.1864253	15.0977388	18.0538078	0.000266933	0.002000713	948.4135095
N-6-iso-pentenyladenosine-5'-monophosphate	CK	19.3476793	19.1093877	17.3601911	22.6755231	22.3714431	21.3788434	0.013376333	0.015533806	0.840294735
L-Phenylalanine	SA	3317.15925	3550.52975	3087.62758	1032.69888	871.859756	1176.8708	0.000333452	0.002000713	3.230746245
cis(+)-12-Oxophytodienoic acid	JA	262.8961	289.305725	294.053932	326.110938	331.379573	301.343922	0.048948065	0.050346581	0.882587992
3-oxo-2-(2-(Z)-Pentenyl) cyclopentane-1-butyric acid	JA	9.54222527	10.8357529	13.4329848	0	0	0	0.010146647	0.013045689	Inf
Dihydrozeatin ribonucleoside	CK	0	0	0	6.10959188	5.98445122	5.26440643	0.00206527	0.004646858	0
Gibberellin A12 aldehyde	GA	4.85184339	4.91732885	3.65154092	0	0	0	0.008353264	0.011912533	Inf
Salicylic acid	SA	0	0	0	62.5875285	47.2009654	58.2691916	0.006623666	0.010367478	0
Jasmonic acid	JA	0	0	0	20.498498	22.8776931	23.7208053	0.001855623	0.00457958	0
Indole-3-carboxaldehyde	Auxin	0	0	0	10.5030298	8.43902439	11.0397831	0.006233384	0.010200082	0
Salicylic acid 2-O-β-glucoside	SA	10888.2287	10047.2188	14192.2153	0	0	0	0.011471241	0.014028473	Inf
Jasmonoyl-L-isoleucine	JA	0	0	0	4.21900249	4.92317073	4.88711566	0.002388336	0.005057653	0
Dihydrozeatin-O-glucoside riboside	CK	0	0	0	1.65969028	1.58392022	1.59102779	0.000224667	0.002000713	0
N6-isopentenyladenine	CK	0.12448964	0.135256214	0.151660997	0	0	0	0.003301982	0.006130002	Inf
Abscisic acid	ABA	0	0	0	3.65345971	3.640625	3.92794237	0.000626948	0.002507794	0
N6-Benzyladenine-7-glucoside	CK	6.71018688	7.4140434	9.14746348	0	0	0	0.008603496	0.011912533	Inf
Dihydrozeatin	CK	0	0	0	0.972995649	0.765914634	0.957763185	0.005450161	0.009343134	0
3-Indole acetamide	Auxin	2.84686675	2.72720558	2.61194217	0	0	0	0.000617194	0.002507794	Inf
N-(3-Indolylacetyl)-L-phenylalanine	Auxin	0.262192769	0.204482478	0.191119672	0	0	0	0.009747608	0.012996811	Inf

Table S11. Primer used in this study.

Primer	Sequence (5'-3')	Purpose
Op05g01165-F	ATGAATGTTCTAGCAAACACACTA	gene cloning
Op05g01165-R	TCAATGATGGCCCTCCATGGA	gene cloning
Op11g00940-F	ATGGCCATCACTCATCATTCAG	gene cloning
Op11g00940-R	TTAATTGTAGGCATCAGGATTAG	gene cloning
Op10g00760-F	ATGGCTGCTTGGAAGAGAAGC	gene cloning
Op10g00760-R	TTAGGCTTTTACTCGGCCTTC	gene cloning
Op09g01504-F	ATGGACTTAGCAGCCATTTTCAC	gene cloning
Op09g01504-R	CTAGTTACAAAGTTCACGAGCAA	gene cloning
Op01g02324-F	ATGGCCTCCCACCTCATGAA	gene cloning
Op01g02324-R	TTAGACGTCACTATCATAAGGAA	gene cloning
Op06g00213-F	ATGGTGCTCAATTCTCAACTCAAT	gene cloning
Op06g00213-R	TTAGTCAAGAGACTTGGATATCCA	gene cloning
OpG10H-F	ATGGATTACCTCACTATTGCTCTAG	gene cloning
OpG10H-R	TCAAAGAGAAATTGGTACAGCTAGC	gene cloning
OpSLS-F	ATGCAAACATCCTACGGTTTGAC	gene cloning
OpSLS-R	TTACAGTTTGTGCAAAATCAAGTG	gene cloning
OpLAMT-F	ATGGCCCCAACCATGGACAACAA	gene cloning
OpLAMT-R	TTAATTGATTTTGCGTTTTAGGA	gene cloning
OpSTR1-F	ATGCATAGTTCAGAAGCCATGG	gene cloning
OpSTR1-R	TCAGAAAGAAGAAAATTCCTTG	gene cloning
OpTDC-F	ATGGGCAGCATTAGTGAAAATTG	gene cloning
OpTDC-R	TTACTCAATGATATTGGTTTTCG	gene cloning
OpTDC3-F	ATGGGCAGCATTAATGAAAATT	gene cloning
OpTDC3-R	ATCAATGAATTGCTTTTCCTTG	gene cloning
OpTDC4-F	ATGGGCAGCATTGATGCAAAT	gene cloning
OpTDC4-R	AATATCAATGCACTGATGATTTTC	gene cloning
OpTDC5-F	ATGGGAAGCATTGATACAAATAAT	gene cloning
OpTDC5-R	GTGATCATTTTCCTTAAGTAAAG	gene cloning
OpSTR2-F	ATGGCAAAAACTGAGCTAGTTC	gene cloning
OpSTR2-R	ACCTAGCCTTGGAGGCCGGTATA	gene cloning
OpAVT1-F	ATGGATCAGGGACCAAAAAATTG	gene cloning
OpAVT1-R	TTAGCTTACAAATTGCCTTATTAAC	gene cloning
AtAAP6-F1	ATGGAGAAGAAGAAGAGCATGTT	gene cloning
AtAAP6-R	CTAAGGAGCCTGGAAAGGCTT	gene cloning
pKW89-Op05g01165-Eco32I-F	cggccgccagatatcATGAATGTTCTAGCAAACACACTA	ISH
pKW89-Op05g01165-KpnI-R	tctcactgaggtaccATGATGGCCCTCCATGG	ISH
pKW89-Op11g00940-Eco32I-F	cggccgccagatatcATGGCCATCACTCATCATTCAG	ISH
pKW89-Op11g00940-KpnI-R	tctcactgaggtaccATTGTAGGCATCAGGAT	ISH
pKW89-Op10g00760-Eco32I-F	cggccgccagatatcATGGCTGCTTGGAAGAGAAGC	ISH
pKW89-Op10g00760-KpnI-R	tctcactgaggtaccGGCTTTTACTCGGCCTTC	ISH
pKW89-Op09g01504-Eco32I-F	cggccgccagatatcATGGACTTAGCAGCCATTTTCAC	ISH
pKW89-Op09g01504-KpnI-R	tctcactgaggtaccGTTACAAAGTTCACGAG	ISH
pKW89-Op01g02324-Eco32I-F	cggccgccagatatcATGGCCTCCCACCTCAT	ISH
pKW89-Op01g02324-KpnI-R	tctcactgaggtaccGACGTCACTATCATAAG	ISH

pKW89-Op06g00213-Eco32I-F	cggccgccagatatcATGGTGCTCAATTCTCAACTCAAT	ISH
pKW89-Op06g00213-KpnI-R	tctcactgaggtaccGTCAAGAGACTTGGATA	ISH
pKW89-OpG10H-Eco32I-F	cggccgccagatatcATGGATTACCTCACTATTGCTCT	ISH
pKW89-OpG10H-KpnI-R	tctcactgaggtaccAAGAGAAATTGGTACAGCTAGC	ISH
pKW89-OpSLS-Eco32I-F	cggccgccagatatcATGCAAACATCCTACGGTTTGAC	ISH
pKW89-OpSLS-KpnI-R	tctcactgaggtaccCAGTTTGTGCAAAATCA	ISH
pKW89-OpLAMT-Eco32I-F	cggccgccagatatcATGGCCCCAACCATGGACAACAA	ISH
pKW89-OpLAMT-KpnI-R	tctcactgaggtaccATTGATTTTGCGTTTTAG	ISH
pKW89-OpSTR1-Eco32I-F	cggccgccagatatcATGCATAGTTCAGAAGCCATGG	ISH
pKW89-OpSTR1-KpnI-R	tctcactgaggtaccGAAAGAAGAAAATTCCT	ISH
pKW89-OpTDC-Eco32I-F	cggccgccagatatcATGGGCAGCATTAGTGAAAATTG	ISH
pKW89-OpTDC-KpnI-R	tctcactgaggtaccCTCAATGATATTGGTTTT	ISH
pKW89-OpTDC3-Eco32I-F	cggccgccagatatcATGGGCAGCATTAATGAAAATT	ISH
pKW89-OpTDC3-KpnI-R	tctcactgaggtaccATCAATGAATTGCTTTTCCTTG	ISH
pKW89-OpTDC4-Eco32I-F	cggccgccagatatcATGGGCAGCATTGATGCAAAT	ISH
pKW89-OpTDC4-KpnI-R	tctcactgaggtaccAATATCAATGCACTGATGATTTTC	ISH
pKW89-OpTDC5-Eco32I-F	cggccgccagatatcATGGGAAGCATTGATACAAATAAT	ISH
pKW89-OpTDC5-KpnI-R	tctcactgaggtaccGTGATCATTTTCCTTAAGTAAAG	ISH
pKW89-OpSTR2-Eco32I-F	cggccgccagatatcATGGCAATTATCCTCACTCTCATC	ISH
pKW89-OpSTR2-KpnI-R	tctcactgaggtaccATATGGAGAAGAGGGTTTTTCC	ISH
pCold-OpTDC3-EcoRI-F	ctcggtaccctcgagggatccATGGGCAGCATTAATGAAAATT	catalysis
pCold-OpTDC3-BamHI-R	caggtcgacaagcttgaattcATCAATGAATTGCTTTTCCTTG	catalysis
pCold-OpTDC4-EcoRI-F	ctcggtaccctcgagggatccATGGGCAGCATTGATGCAAAT	catalysis
pCold-OpTDC4-BamHI-R	caggtcgacaagcttgaattcAATATCAATGCACTGATGATTTTC	catalysis
pCold-OpTDC5-EcoRI-F	ctcggtaccctcgagggatccATGGGAAGCATTGATACAAATAAT	catalysis
pCold-OpTDC5-BamHI-R	caggtcgacaagcttgaattcGTGATCATTTTCCTTAAGTAAAG	catalysis
pCold-OpSTR1-EcoRI-F	ctcggtaccctcgagggatccATGCATAGTTCAGAAGCCATGG	catalysis
pCold-OpSTR1-BamHI-R	caggtcgacaagcttgaattcGAAAGAAGAAAATTCCTTG	catalysis

pCold-OpSTR2-EcoRI-F	ctcggtaccctcgagggatccATGGCAATTATCCTCACTCTCATC	catalysis
pCold-OpSTR2-BamHI-R	caggtcgacaagcttgaattcATATGGAGAAGAGGGTTTTTCC	catalysis
pESC-Ura-OpAVT1-EcoRI-F	aatttttgaaaattcgaattcATGGATCAGGGACCAAAAAATTG	complementation analysis
pESC-Ura-OpAVT1-SpeI-R	ttgtaatccatcgatactagtGCTTACAAATTGCCTTATTAACTCC	complementation analysis
pESC-Ura-AtAAP6-XhoI-F	atttccgaagaagacctcgagATGGAGAAGAAGAAGAGCATGTT	complementation analysis
pESC-Ura-AtAAP6-HindIII-R	gctagccgcggtaccaagcttAGGAGCCTGGAAA	complementation analysis
